# Visible-light-induced indole synthesis *via* intramolecular C–N bond formation: desulfonylative C(sp^2^)–H functionalization†

**DOI:** 10.1039/d2sc02822k

**Published:** 2022-09-16

**Authors:** Quanzhe Li, Xintao Gu, Yin Wei, Min Shi

**Affiliations:** State Key Laboratory of Organometallic Chemistry, Center for Excellence in Molecular Synthesis, Shanghai Institute of Organic Chemistry, University of Chinese Academy of Science, Chinese Academy of Sciences 345 Lingling Road Shanghai 200032 China weiyin@sioc.ac.cn; Key Laboratory for Advanced Materials and Institute of Fine Chemicals, School of Chemistry & Molecular Engineering, East China University of Science and Technology 130 Meilong Road Shanghai 200237 China mshi@mail.sioc.ac.cn

## Abstract

Despite significant advances made on the synthesis of indole derivatives through photochemical strategies during the past several years, the requirement of equivalent amounts of oxidants, bases or other additional additives has limited their practical applications in the synthesis of natural products and pharmaceuticals as environment-friendly processes. Herein, we report LED visible-light-induced redox neutral desulfonylative C(sp^2^)–H functionalization for the synthesis of *N*-substituted indoles with a broad scope through γ-fragmentation under mild conditions in the absence of any additional additive. The reaction mechanism paradigm has been investigated on the basis of deuterium labeling experiments, kinetic analysis, Hammett plotting analysis and DFT calculations.

## Abstract 2

LED visible-light-induced redox neutral desulfonylative C(sp^2^)–H functionalization for the synthesis of N-substituted indoles in the absence of any additional additive has been established on the basis of KIE, Hammett plotting and DFT calculations.

## Graphical abstract

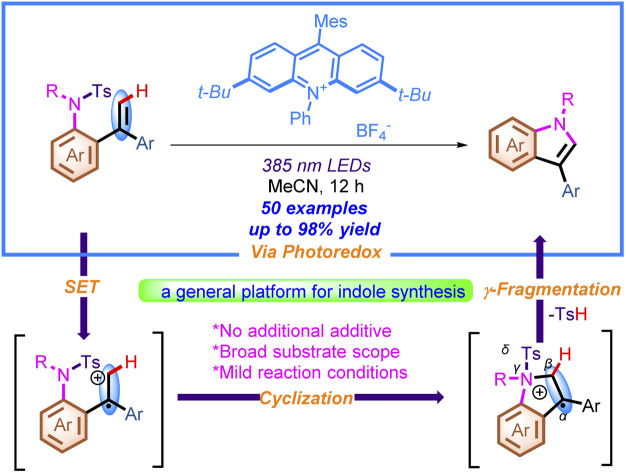

## Introduction

**Scheme 1:**
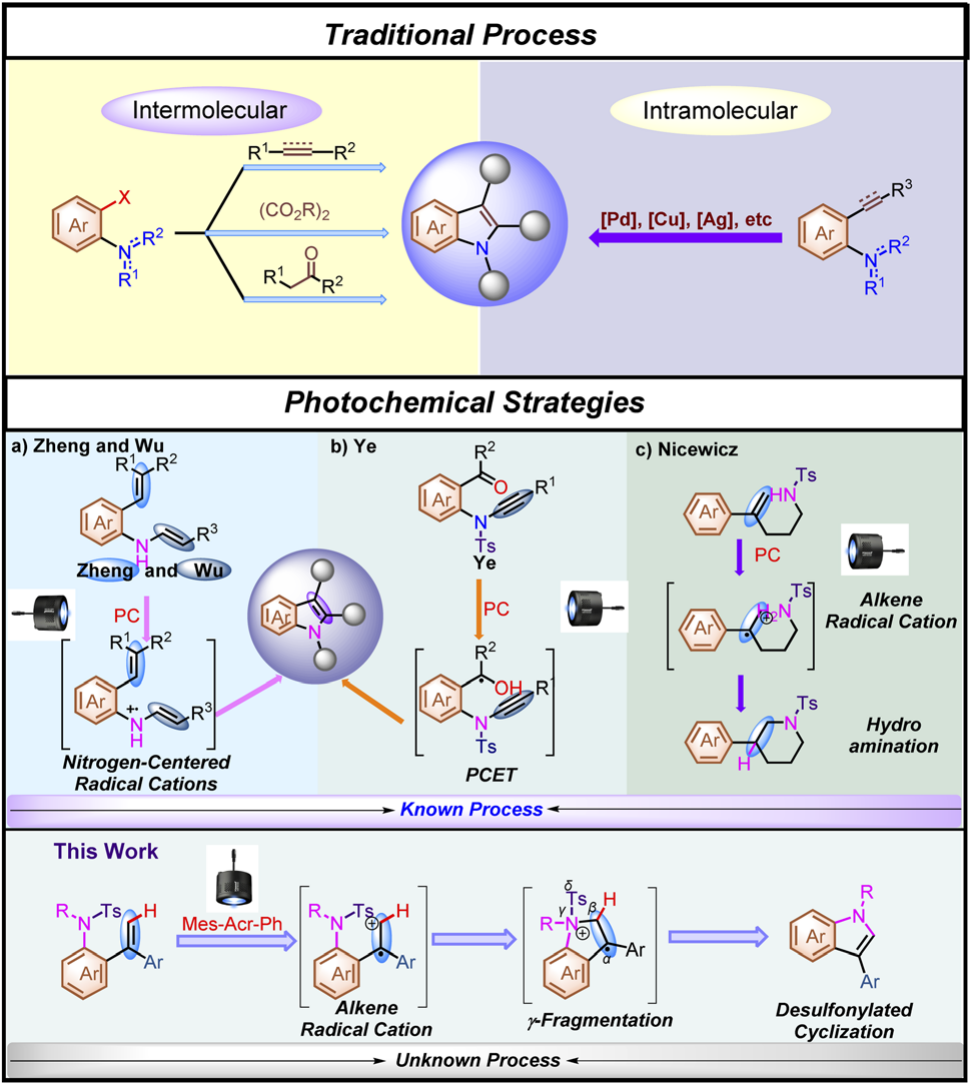
Previous studies on the preparation of indole motifs and this work.

Indole motifs are privileged scaffolds found in many natural products^1^ and biologically active compounds,^2^ and therefore they have been a target of numerous methodology developments.^3^ However, many of these developed methodologies are facing the lack of starting material availability and functional-group tolerance, resulting in restricted practicability or the requirement of high costs of starting materials. In general, starting from *ortho*-halogenated aniline derivatives, most of the synthetic methodologies had to use stoichiometric amounts of oxidants, bases or others as additional additives in the reaction under harsh reaction conditions, leading to the generation of undesired wastes^3*b*,*g*^ (Scheme 1, Traditional Process). Furthermore, as for traditional intramolecular indole synthetic methodologies, although metal-catalyzed processes have been well developed,^3*g*,*h*,4^ the use of prefunctionalized aniline derivatives is still required in most of the cyclization processes (Scheme 1, Traditional Process). Thus, the further development of new types of practical synthetic methods for the rapid construction of indole motifs is highly desired.^5^

Visible-light activated photoredox catalysis for the synthesis of indole derivatives has recently emerged as a novel reaction mode and an alternative to traditional processes for the synthesis of indole derivatives.^6^ For example, Zheng's group^7^ and Wu's group^8^ delicately reported an intramolecular indole synthesis initiated by an efficient single electron oxidation of the nitrogen atom using styryl aniline derivatives and *N*-aryl enamines, respectively under visible-light-irradiation (Scheme 1a, Photochemical Strategy). In addition, sulfonamides, as robust protecting groups, are easy to purify and are very stable under various reaction conditions.^9^ Recently, desulfonylation of tosyl amides through catalytic photoredox cleavage processes has attracted much attention from organic chemists.^10^ In 2020, Ye and co-workers^11^ reported an outstanding study of ynamide Smiles rearrangement triggered by visible-light-mediated regioselective ketyl–ynamide coupling, which enables facile access to a variety of valuable 2-benzhydrylindoles or isoquinolines with a broad substrate scope in generally good yields under mild reaction conditions (Scheme 1b, Photochemical Strategy). On the other hand, Fukuzumi acridinium salts (*E_1/2red_ > +1.60 V *vs.* SCE),^12^ as excellent photo-oxidation catalysts in the excited state, have been extensively reported to oxidize alkenes through single-electron oxidation. In this respect, Nicewicz,^13^ Glorius^14^ and others^15^ have demonstrated that alkene radical cations are susceptible to highly regioselective nucleophilic attack, and because this step does not involve a discrete nucleophile–catalyst interaction, they are relatively agnostic toward the identity of the nucleophilic reaction partner (Scheme 1c, Photochemical Strategy). On the basis of these research backgrounds, we envisaged that if an appropriate organic radical intermediate and desulfonylated elimination could be merged together, a redox neutral process might be able to be established as a brand-new alternative photochemical platform for indole synthesis through γ-fragmentation. Herein, we wish to disclose how this hypothesis was translated into experimental reality, enabling a desulfonylated process as an additional additive-free and step-economic strategy for the synthesis of indole (Scheme 1, this work).

**Scheme 2:**
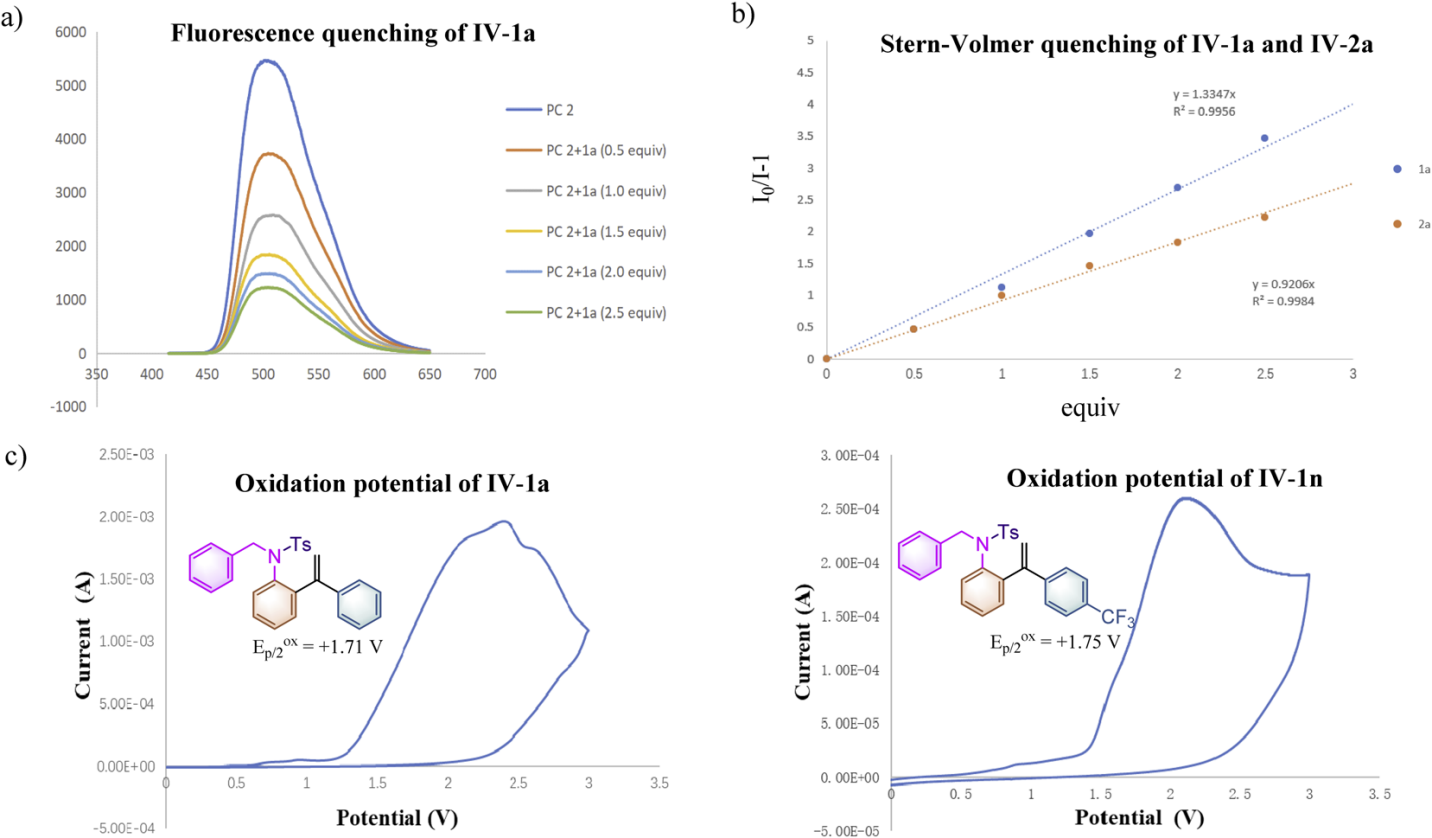
Mechanistic studies. (a) Solution of PC 2 (5.0 × 10^−3^ M) was added to 1a (1.0 M, 0.5 equiv. as gradient) in dry MeCN upon excitation at 415 nm. (b) Solution of PC 2 (5.0 × 10^−3^ M) was added to 1a and 2a (1.0 M, 0.5 equiv. as gradient), respectively in dry MeCN upon excitation at 415 nm. (c) A solution of the substrate 1 in MeCN (0.2 M) was tested with 0.2 M Bu_4_NPF_6_ as the supporting electrolyte, using glassy carbon as the working electrode, Pt as the counter electrode, and a saturated calomel electrode reference electrode. Scan rate = 0.1 V s^−1^, 2 sweep segments, a sample interval of 0.001 V.

To verify our working hypothesis, we initiated our study by using tosylated biphenylalkene derivative 1a (0.1 mmol, 1.0 equiv.) as a substrate, xanthone (5.0 mol%) as a photosensitizer and CH_3_CN as the solvent under an argon atmosphere upon 12 W 365 nm LED light irradiation for 12 hours. To our delight, the desired indole product 2a was produced in 15% yield (Table 1, entry 1). Using thioxanthone and *fac*-Ir(ppy)_3_ as photocatalysts did not afford 2a at all (Table 1, entries 2 & 3). Since the acridinium derivative had a strong absorption band in the visible region (*λ* = 430 nm) along with the high oxidation potential in the excited state, acridinium PC 1 (*E*_1/2red_* = +2.06 V *vs.* SCE)^12^ was used as the photocatalyst in this reaction, giving 2a in 79% yield (Table 1, entry 4). This uplifting result demonstrated that it was feasible to initiate the reaction through oxidation of the alkene moiety in 1a (*E*^ox^_1/2_ = +1.71 V *vs.* SCE) (for details see Scheme 2c or ESI, at page S43†) upon SET with the excited state of MesAcr^+^*, resulting in the formation of the alkene radical cation to trigger the intramolecular nucleophilic attack of the tosylated amino moiety, giving the desired indole product 2a. Furthermore, using PC 2 (*E*_1/2red_* = +2.08 V *vs.* SCE)^12^ as a photocatalyst provided 2a in 88% (Table 1, entry 5). However, PC 3 (*E*_1/2red_* = +1.65 V *vs.* SCE)^12^ and PC 4 (*E*_1/2red_* = +1.90 V *vs.* SCE),^12^ which had lower redox potentials, were not suited to this transformation of 1a (Table 1, entries 6 & 7). Screening of LED light sources revealed that 385 nm LED light was the best one for the production of 2a (Table 1, entries 8 & 9). Subsequently, the examination of photocatalyst loading indicated that the use of 2.0 mol% PC 2 is the most suitable condition, delivering 2a in the highest yield of 91% along with 90% yield of the isolated products (Table 1, entries 10–13). As for solvent effect studies, it was found that THF and DMF were not productive in this transformation (Table 1, entries 14 & 15) and using DCM as the solvent gave 2a in 51% yield (Table 1, entry 16). Moreover, we also found that the yield of 2a significantly decreased in the presence of sacrificial reducing agents such as Hantzsch ester and DIPEA (Table 1, entries 17 & 18). The control experiments illustrated that both the photocatalyst and light irradiation were requisite for the generation of indole product 2a (Table 1, entries 19 & 20) (for more information, see Tables S1–S3 in the ESI†). A scale-up reaction could be performed with 1.0 mmol of 1a, affording 2a in 82% yield, *i.e.*, 232 mg (Table 1, entry 21) (see ESI at page S30†).

**Table 1:** Optimization of reaction conditions^a^^b^

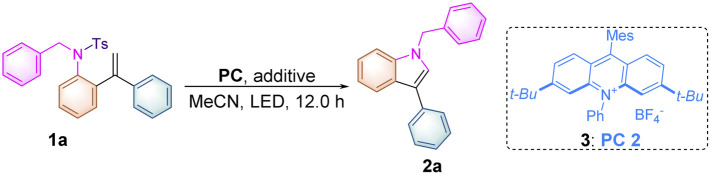						
Entry^a^	PC	PC (mol%)	Additive	Solvent	LED	Yield^b^ (%)
1	Xanthone	5.0	—	MeCN	365 nm	15
2	Thioxanthone	5.0	—	MeCN	365 nm	—
3	*fac*-Ir(ppy)_3_	5.0	—	MeCN	365 nm	—
4	PC 1	5.0	—	MeCN	365 nm	79
5	PC 2	5.0	—	MeCN	365 nm	88
6	PC 3	5.0	—	MeCN	365 nm	—
7	PC 4	5.0	—	MeCN	365 nm	—
8	PC 2	5.0	—	MeCN	385 nm	90
9	PC 2	5.0	—	MeCN	Blue LED (100 W)	88
10	PC 2	1.0	—	MeCN	385 nm	83
**11**	PC 2	**2.0**	—	**MeCN**	**385 nm**	**91 (90)** c
12	PC 2	3.0	—	MeCN	385 nm	81
13	PC 2	4.0	—	MeCN	385 nm	83
14	PC 2	2.0	—	THF	385 nm	<5
15	PC 2	2.0	—	DMF	385 nm	—
16	PC 2	2.0	—	DCM	385 nm	51
17^d^	PC 2	5.0	DIPEA	MeCN	385 nm	29
18^d^	PC 2	5.0	Hantzsch ester	MeCN	385 nm	—
19	—	—	—	MeCN	385 nm	—
20^e^	PC 2	2.0	—	MeCN	385 nm	—
21^f^	PC 2	2.0	—	MeCN	385 nm	82^c^
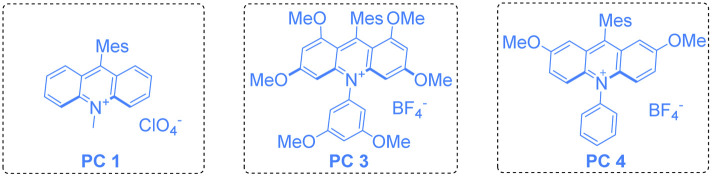						

a Reaction conditions: 1a (0.1 mmol) and PC (*x* mol%) were added to degassed MeCN (2.0 mL) under an Ar atmosphere for 12.0 h, in a sealed tube using LED light irradiation.

b NMR yield using 1,3,5-trimethoxybenzene as an internal standard.

c Yield of the isolated products.

d Additive (0.2 mmol) was used.

e Under dark conditions.

f A scale-up reaction with 1.0 mmol of 1a.

With the optimized conditions identified, the substrate scope of this reaction has been examined with a wide range of substrates 1 and it was found that most of them underwent this transformation successfully, providing the desired products in good yields (Table 2). As for introducing substituents onto the benzene ring connected both to the alkene and sulfonated moieties, we found that substrates 1c–g bearing electron-withdrawing or -donating substituents, such as halogen atoms, fluorine or methyl or *tert*-butyl groups, turned out to be applicable under the optimal reaction conditions, affording the desired products 2c–g in good to excellent yields ranging from 75% to 88%. Surprisingly, in the case of substrate 1b having a strongly electron-donating methoxyl group in the benzene ring, no reaction occurred, which is quite different from the Stephenson's observation,^16^ in which the electron-rich phenyl ring, with decreasing oxidation potential of styrene, would promote the reaction. For substrate 1b, the BET (back electron transfer) process^17^ may easily occur because of the lower barrier of the SET process in the initial state in this reaction, indicating that no reaction could take place along with complete recovery of the starting materials. Replacing the benzene ring by a naphthalene unit gave the corresponding product 2h in 79% yield. Subsequently, we expanded the substrate scope of the substituted benzene ring connected only to the alkene moiety and found that introducing substituents such as methyl or ethyl groups, halogen atoms, fluorine groups or trifluoromethyl groups at 2′-, 3′-, and 4′-positions of the benzene ring turned out to be compatible, furnishing the desired products 2i–p in 76–85% yields. The structure of 2k had been unambiguously determined by X-ray diffraction as shown in Table 2 and the corresponding CIF data are presented in the ESI.† As for substrate 1q having an electron-rich aromatic ring and substrate 1r bearing a heteroaromatic ring, no reactions occurred as well similar to that of 1b along with complete recovery of the starting materials. The tethered benzyl group in substrates 1s–x has also been examined and we found that when introducing cyano, methyl or trifluoromethyl groups as well as fluorine atoms onto its benzene ring, the reactions proceeded smoothly, delivering the desired products 2s–x in 69–89% yields. The benzene ring could be replaced by a naphthyl group, giving 2y in 76% yield. Besides the benzyl group, R^6^ could be a methyl group (substrate 1z, R^6^ = Me) or an isopropyl group (substrate 1aa, R^6^ = *i*-Pr), affording the desired products 2z and 2aa in 89% and 78% yields, respectively. As the nucleophilicity of the tosylated nitrogen atom is also an essential factor for this photo-induced cyclization, the tethered alkyl effect based on the length of the carbon chain along with its electronic effect has also been investigated. Substrates 1ab–af, in which R^7^ = OMe, C_2_H_5_, CO_2_Me, Br, or Ph bearing three –CH_2_-moieties, were tolerated under the optimal reaction conditions, affording the desired products 2ab–af in moderate to good yields ranging from 54% to 84%. However, in the case of substrate 1ag (R^7^ = CN), we failed to get the desired product 2ag after several attempts under different reaction conditions, presumably due to that the aliphatic cyano group may interact with the excited photocatalyst to impair the reaction proceeding or the electronic nature of the aliphatic cyano group may enhance its oxidation potential, disturbing the SET process between the excited photocatalyst and 1ag. Furthermore, for substrates 1ah–aj, in which R^8^ = OTBS, Br, or CF_3_ bearing two –CH_2_- moieties, the reactions became less facile under the standard conditions, affording the corresponding products 2ah–aj in 32–77% yields. It should be noted that substrates 1ak (R^9^ = COOMe) and 1al (R^9^ = CF_3_) bearing one –CH_2_- moiety did not undergo the desired process to give the indole product, indicating that the inductive effect plays a role in the nucleophilicity of the tosylated nitrogen atom and the electron-withdrawing CO_2_Me or CF_3_ at the β position of the nitrogen atom can decrease its nucleophilicity. In the meantime, for substrate 1am having a menthol skeleton and substrate 1an bearing a modified sugar group, the reactions were also tolerated, delivering the corresponding products 2am and 2an in 46% and 75% yields, respectively. Furthermore, using substrates 1ao (*Z*/*E* = 1/0.9) and 1ap (*Z*/*E* = 1/0.4) in the reaction showed that only the *E* configuration was reactive along with full recovery of unreactive substrates with the *Z*-configuration because after the reaction completion, the remaining substrates 1ao and 1ap were determined to have the *Z*-configuration upon NOE spectroscopic analysis (see ESI at page S58†). The *Z*–*E* isomerization of anethole reported in Stephenson’s work,^16^ in which the initial rate of (*Z*)-anethole isomerization was much faster than that of (*E*)-anethole isomerization at the photostationary state, could not be identified in this reaction process. Unfortunately, styrene-type substrates 1aq–av were incompatible in this transformation.

**Table 2:** Substrate scope of 1^a^^b^

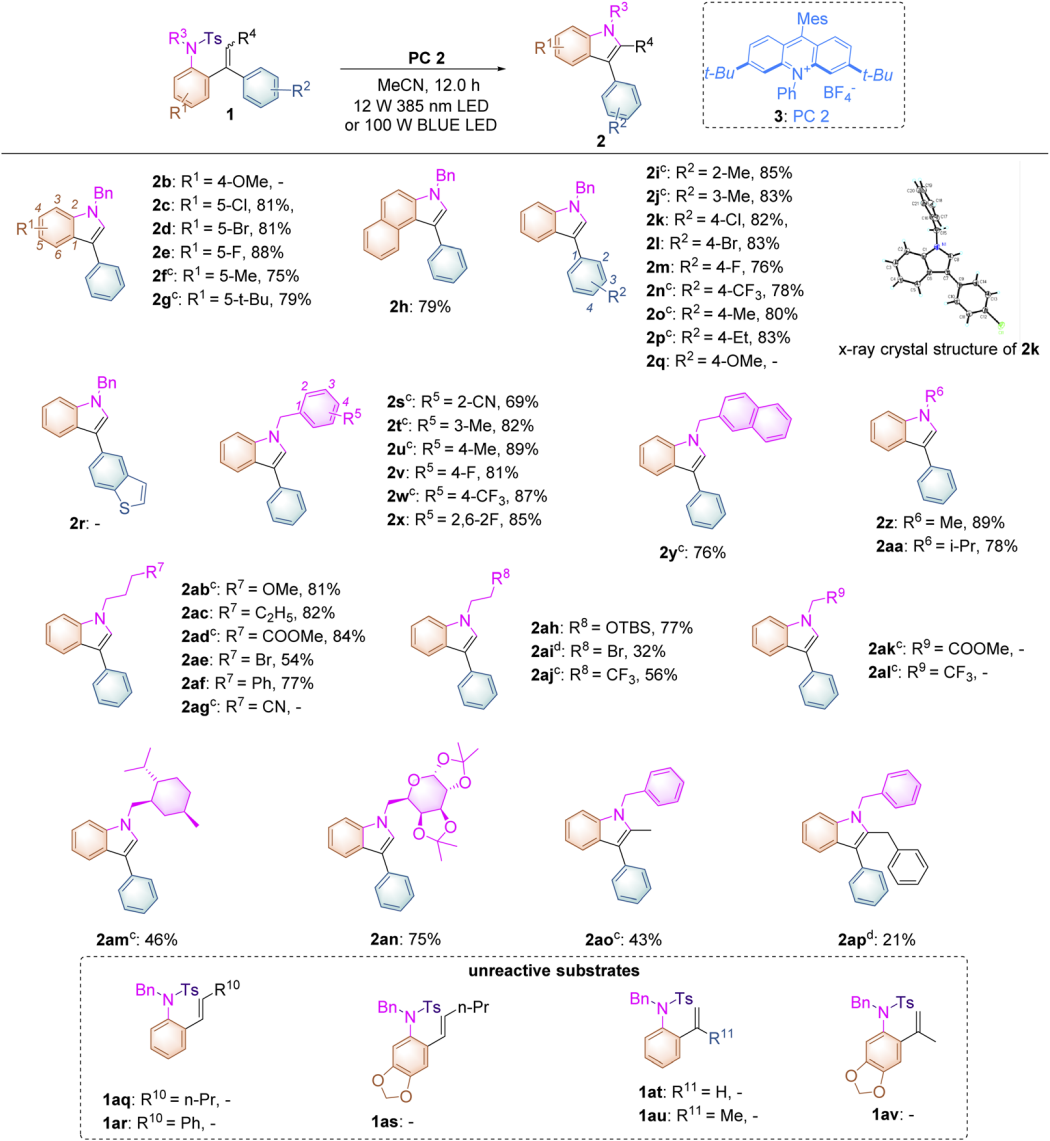

a Reaction conditions: 1 (0.1 mmol) and PC 2 (2.0 mol%) were added to degassed MeCN (2.0 mL) under an Ar atmosphere for 12.0 h, in a sealed tube using 385 nm LED light irradiation.

b Yield of the isolated products.

c Reaction conditions: 1a (0.1 mmol) and PC 2 (2.0 mol%) were added to degassed MeCN (2.0 mL) under an Ar atmosphere for 24.0 h, in a sealed tube using 100 W blue light irradiation.

d Reaction conditions: 1 (0.1 mmol) and PC 2 (2.0 mol%) were added to degassed DCM (2.0 mL) under an Ar atmosphere for 24.0 h, in a sealed tube using 100 W blue light irradiation.

To further broaden the substrate scope on this newly developed visible-light-induced photochemical synthetic methodology and to carry out mechanistic investigations, substrates 1aw–aak, having different *N*-protecting R groups, were studied in this transformation (Table 3). When substrate 1aw (R^1^ = H) was employed, the desired product 2a was obtained in 82% yield. Substrates 1ax–az, in which the fluorine atom was introduced at the 2-, 3-, and 4-positions of the benzene ring, afforded 2a in increased yields of 37%, 64% and 98% respectively perhaps due to the steric and electronic effects under the standard conditions. For substrates 1aaa–aaw, in which R^1^ = 4-Cl, 4-OMe, 4-CF_3_, 4-NO_2_, 4-CN, the desired product 2a could also be obtained in 35–95% yields. Replacing the benzene ring by a naphthalene moiety or a thiophene moiety provided 2a in 85% and 71% yields, respectively. Moreover, substrates 1aah (R = Ms) and 1aai (R = Tf) were also compatible in this methodology, affording 2a in 85% and 60% yields. However, substrates 1aaj (R = Ac), 1aak (R = CF_3_C(O)), 1aal (R = Boc) and 1aam (R = H) were not applicable in this process, indicating that the use of a sulfonyl group as a *N*-protecting group is essential in this photochemical transformation.

**Table 3:** The study of the leaving sulfonyl group and the other *N*-protecting groups in 1aw–aak^a^^b^

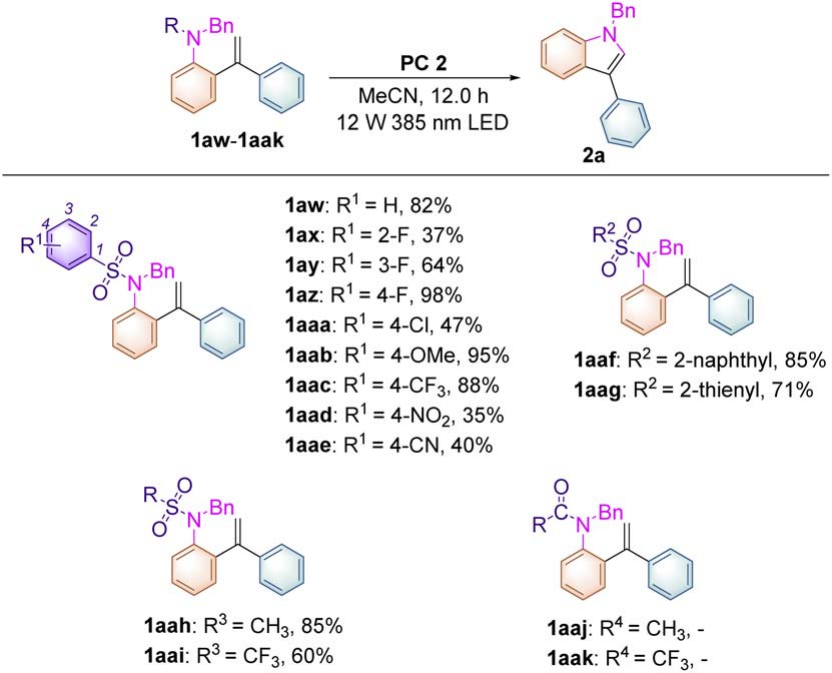

a Reaction conditions: 1aw–aak (0.1 mmol) and PC 2 (2.0 mol%) were added to degassed MeCN (2.0 mL) under an Ar atmosphere for 12.0 h, in a sealed tube using 385 nm LED light irradiation.

b NMR yield using 1,3,5-trimethoxybenzene as an internal standard.

To further clarify the interaction details between the photocatalyst and substrate 1a, the fluorescence quenching experiment of PC 2 (5.0 × 10^−3^ M) with 1a (1.0 M, 0.5 equiv. as a gradient) was performed and the emission of PC 2 was effectively quenched by 1a as shown in Scheme 2a. The Stern–Volmer analytical results of PC 2 with substrate 1a and product 2a are depicted in Scheme 2b, suggesting that 1a can more efficiently quench the emission of PC 2 than that of 2a. To get more accurate results, Stern–Volmer experiments of compounds 1a and 2a were repeated at a lower concentration using PC 2 (5.0 × 10^−5^ M) and similar results were obtained (for more details, see ESI at pages S38–S42†). The oxidation potentials of 1a and 1n were measured as *E*^ox^_p/2_ = +1.71 V *vs.* SCE and *E*^ox^_p/2_ = +1.75 V *vs.* SCE as shown in Scheme 2c, suggesting that the inductive effect on the benzene ring could slightly increase the oxidation potential of substrate 1 (for more details, see ESI, at page S43†). It is noteworthy that the reaction rate of 1n having a CF_3_ group at the *para*-position of the benzene ring was similar to that of 1o having a Me group, while both of them are much slower than that of substrate 1a (see ESI at page S52 and S55† for more details). As measured above, the reaction rate of 1n is more sluggish than that of 1a under identical conditions probably owing to its higher oxidation potential. As for substrate 1o, electronic distribution on the styrene moiety in the transition state may be the key point resulting in the deceleration of the reaction rate of 1o. The relatively high reaction rate of substrate 1a may imply the presence of a significant chain process. To further verify our hypothesis, the quantum yields of the reactions of 1a, 1n and 1o under the standard conditions were measured as 1.77, 0.18 and 0.17, respectively (see ESI, at page S35† for more details). The results indicate that the photochemical chain process indeed exists in the reaction of 1a; a possible radical chain process cannot be excluded in the reactions of 1n and 1o, since it can be a consequence of a highly inefficient initiation process or a short-lived chain propagation process due to the substituent effect.^18^ Overall, this reaction may involve an inefficient radical chain process.

**Scheme 3:**
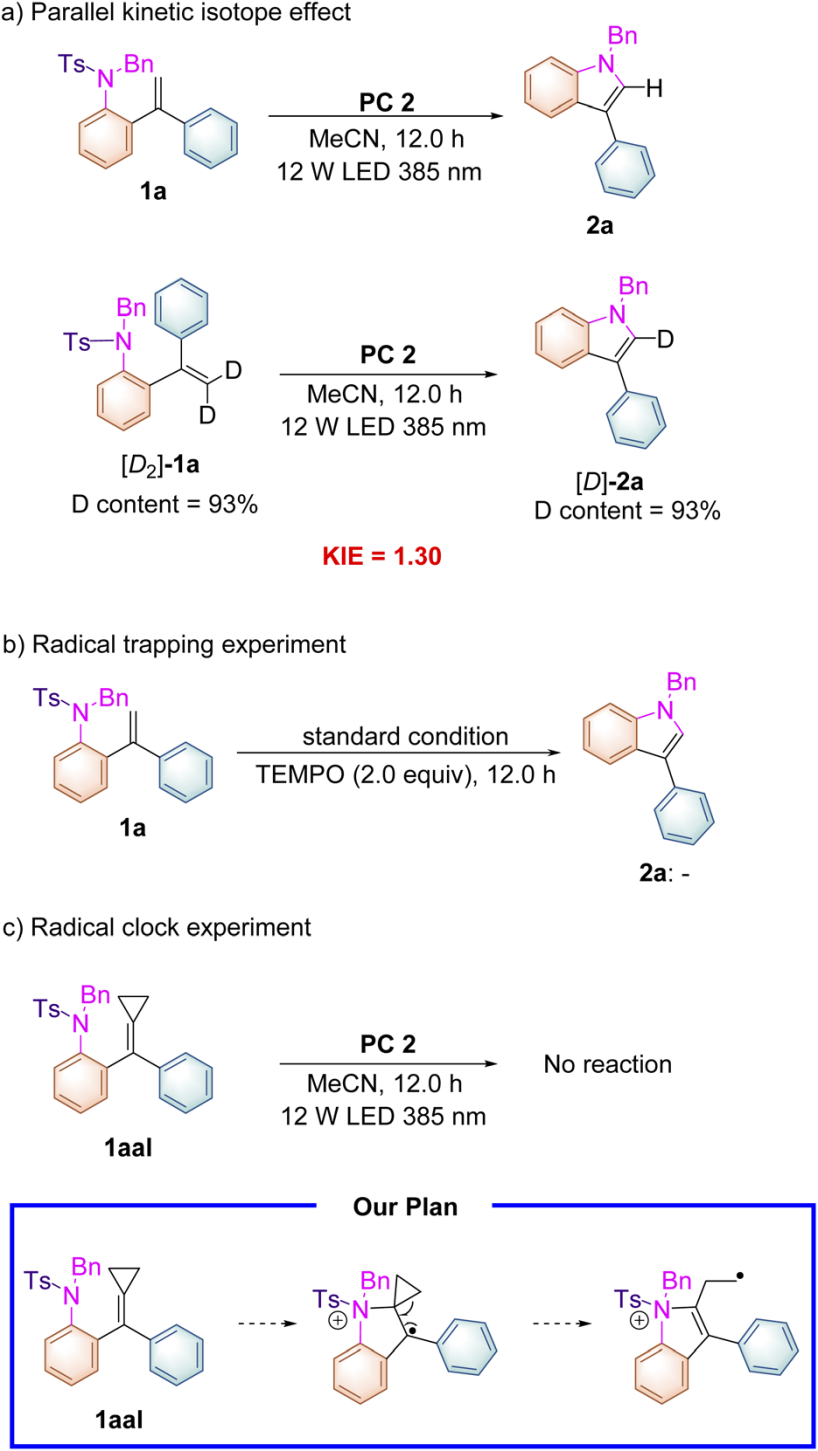
(a) Parallel kinetic isotope effects, (b) radical trapping experiments, and (c) radical clock experiments.

As shown in Scheme 3, the deuterium labeled kinetic experiment was conducted to gain information on the rate-determining step. The kinetic isotope effect of this reaction was measured on the basis of parallel experiments, and a *k*_H_/*k*_D_ value of 1.30 was obtained (Scheme 3a), which suggested that the C–H cleavage might not be involved in the turnover-limiting process. Adding radical scavenger 2,2,6,6-tetramethyl-1-piperidinyloxy (TEMPO) (2.0 equiv.) into the reaction system completely suppressed the reaction proceeding and none of the cyclized products was detected under the standard conditions (Scheme 3b). Subsequently, a radical clock experiment with a cyclopropane-modified alkene 1aal as a substrate was carried out under the standard conditions. Unfortunately, we found that no reaction occurred presumably due to the steric effect (Scheme 3c). This substrate was designed on the basis of our proposed reaction mechanism shown in Scheme 5, in which a radical intermediate 5a was involved as a key radical species.

**Scheme 4:**
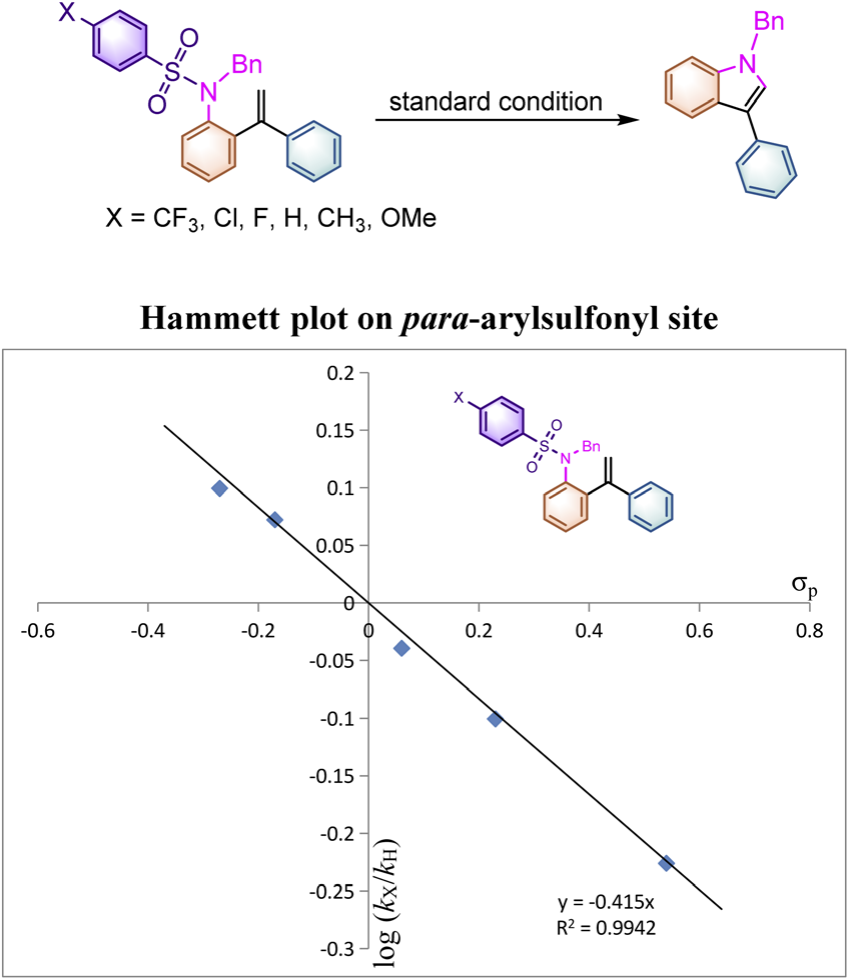
Parallel substituent effect on the *para*-arylsulfonyl site.

**Scheme 5:**
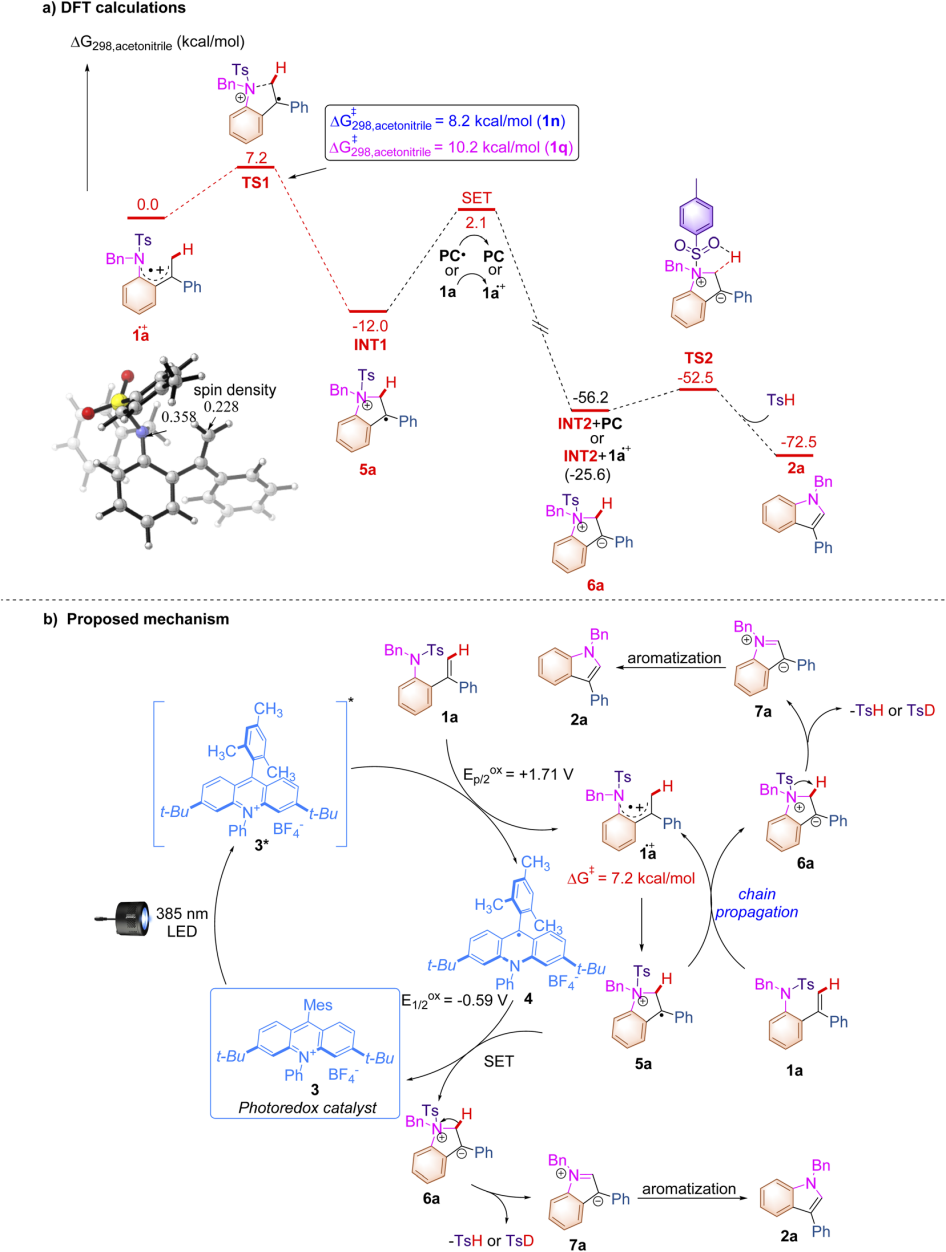
(a) DFT calculations and (b) proposed reaction mechanism.

To better indicate the influence of the electronic characteristics of the arylsulfonyl moiety on this reaction, the Hammett effect was examined in this system. A set of parallel reactions of 1aab, 1a, 1aw, 1az, 1aaa and 1aac bearing various substituents on the benzene ring were carried out under identical conditions. As listed in Table 4, the reaction rates of substrates 1aab and 1a with electron-donating substituents on the *para*-phenylsulfonyl site are found to be faster than those of 1az, 1aaa and 1aac, where the electron-withdrawing substituents decelerated the reaction rate. Plotting log(*k*_X_/*k*_H_) *versus σ*_p_ gave an excellent linear plot with *ρ*_p_ being −0.415, indicating that a positively charged intermediate should be generated during the reaction process (Scheme 4). This result suggested that the reaction initiates from the nucleophilic attack of the nitrogen atom to the alkene moiety to generate a cationic species. As for the rate constants of 4′-substituted substrates 1a, 1l, 1n and 1o measured (see ESI at page S50† for more details), even if the spin-delocalization effect of the substituents was taken into account using the dual parameter equation log(*k*_X_/*k*_H_) = *ρ*^X^*σ*^X^ + *ρ*˙*σ*˙, neither Jiang's^19^*σ*_mb_ and *σ*_JJ_˙ scales nor Creary's^20^ radical scale *σ*_c_˙ gave a linear correlation presumably due to the discrepancy of electronic distribution on the alkene moiety in the transition state, implying that the radical cationic intermediate may be involved in this transformation rather than a simple radical process.

**Table 4:** Rate constant *K*_X_, relative rates log(*k*_X_/*k*_H_) with the *σ*_p_ scale on the *para*-arylsulfonyl site

Subtituent	*k*_X_	*k*_X_/*k*_H_	Log(*k*_X_/*k*_H_)	*σ*_p_
OMe	1.388	1.2572	0.0994	−0.27
CH_3_	1.304	1.1812	0.0723	−0.17
H	1.104	—	—	—
F	1.008	0.9130	−0.0395	0.06
Cl	0.876	0.7935	−0.1005	0.23
CF_3_	0.656	0.5942	−0.2261	0.54

The DFT calculations were conducted to further clarify the plausible reaction pathways. All calculations have been performed at the SMD/B3LYP/6-311+G(d,p)//B3LYP/6-31G(d) level with the Gaussian 16 program.^21^ The solvation Gibbs free energy profile in acetonitrile for the suggested reaction pathway is shown in Scheme 5a (see ESI at page S268† for the details). The excited state of PC 2 (*E*^red^_p/2_ = +2.08 V *vs.* SCE) can facilitate the conversion of 1a (*E*^ox^_p/2_ = +1.71 V *vs.* SCE) to generate 1a^.+^*via* SET. The analysis of spin density shows that some amounts of spin densities are delocalized across nitrogen and carbon centers (0.358 and 0.228)^22^ and the rest of the spin densities are delocalized on aryl groups. Subsequently, 1a^.+^ undergoes cyclization *via*TS1 with an energy barrier of 7.2 kcal mol^−1^ to form a radical cation intermediate INT1, which is an exothermic process (Δ*G* = −12.0 kcal mol^−1^). The energy barriers for cyclization with respect to substrates 1n and 1q are 8.2 kcal mol^−1^ and 10.2 kcal mol^−1^ (for details, see ESI at page S277†), which are in line with the experimental results that show that the reaction rate of 1n is slower than that of 1a and 1q cannot undergo this reaction. The intermediate INT2 is generated through a SET process with an energy barrier of 14.1 kcal mol^−1^ between INT1 and PC^.+^ or between INT1 and 1a, which is a highly exothermic process and indicates that the radical chain process shown in Scheme 5b is thermodynamically favorable and has a possibility to take place. The intermediate INT2 exists as a close ion pair. The Ts^−^ anion moiety in INT2 promotes deprotonation to give product 2a and release TsH through a low activation barrier of 3.7 kcal mol^−1^, which agrees with the result of the KIE experiment, indicating that the C–H cleavage is not involved in the turnover-limiting process. The experimentally observed KIE (1.3) suggested the potential normal secondary KIE effect, which usually implies a reaction from the sp^3^–sp^2^ ground-state to a transition-state in the rate-determining step. However, this photochemical reaction may not follow the normal rule since it involves the photoexcitation and SET processes which require higher energies than other steps. Based on the aforementioned experimental results and the detailed mechanistic investigations, we proposed a plausible reaction mechanism paradigm for this photochemical transformation (Scheme 5b). First, photoexcitation of acridinium salt 3 affords a highly oxidizing excited state MesAcrPh^+^* (*E_1/2red_ = +2.06 V *vs.* SCE), which is reductively quenched by substrate 1a, furnishing radical cationic species 1a^.+^ and acridine radical 4. The intramolecular nucleophilic attack of the nitrogen atom to the alkene moiety in 1a^.+^ affords cyclized radical cationic intermediate 5a with an energy barrier of 7.2 kcal mol^−1^, which undergoes a SET process with acridine radical 4 to deliver a zwitterionic intermediate 6a along with the regeneration of acridinium salt 3 in the closed catalytic loop. The elimination of TsH (or TsD) from 6a provides another zwitterionic intermediate 7a, which undergoes a subsequent aromatization to furnish the desired indole product 2a. Given the fact that the quantum yield of this reaction has been detected as 1.77, a chain process, in which 5a^.+^ acts as an oxidant for another molecule of 1a to produce 6a and 1a^.+^, takes place to afford 2a as well through the same intermediate 7a. The formation of TsH (or TsD) has been confirmed by Mass spectroscopy (see ESI at page S57†). As for the multistep process, it should be emphasized here that the kinetics of this reaction is probably related to both cyclization (1a^.+^ to 5a) and SET (5a to 6a) processes which are defined as rate determining steps.^23^

**Scheme 6:**
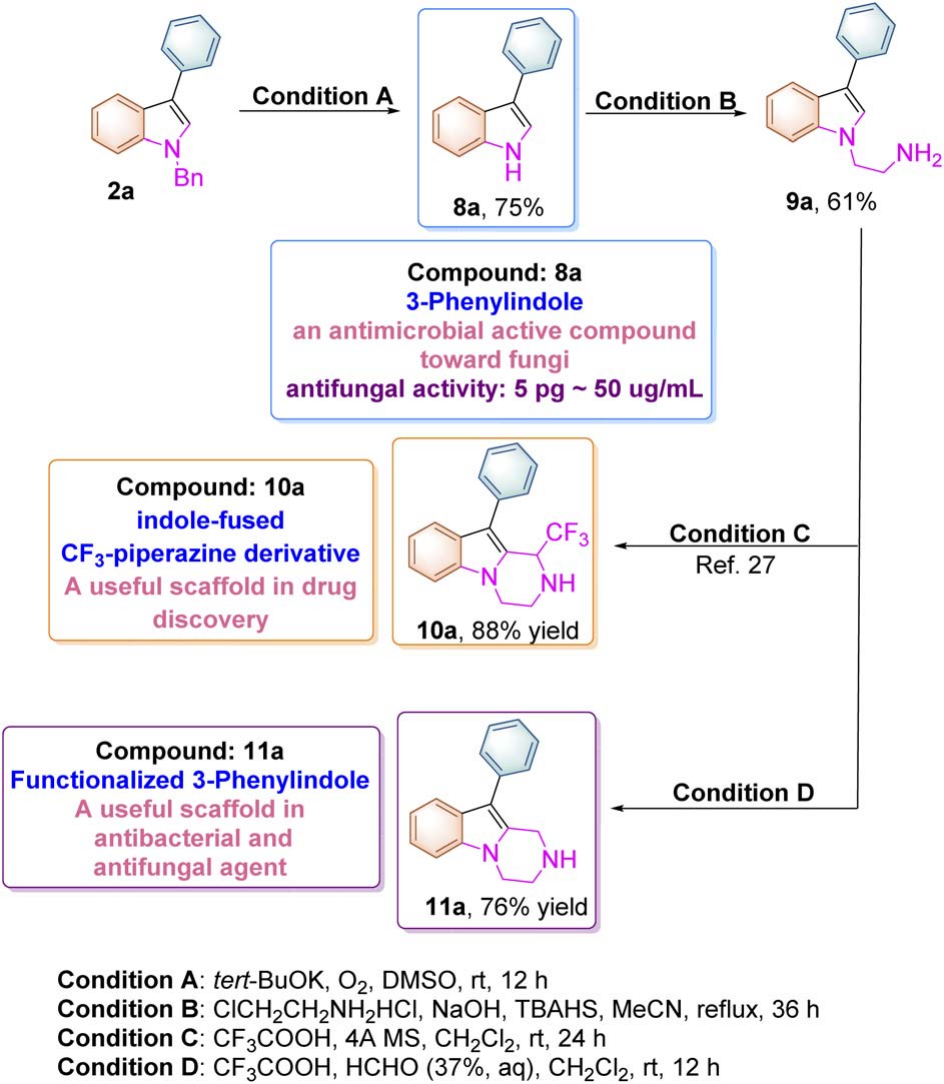
Synthetic routes to bioactive indole compounds.

The *N*-benzyl substituted indole product 2a can be easily transformed to the corresponding unsubstituted indole upon treatment with *t*-BuOK/DMSO and molecular oxygen at room temperature according to the previous literature (Scheme 6).^24^ The compound 8a itself (3-phenylindole) is a biologically active substance, which can be used as an antimicrobial active compound toward many fungi and Gram-positive bacteria. For instance, it inhibits the growth of *Aspergillus niger* at the 5 pg mL^−1^ level and can also suppress spore germination at 50 μg mL^−1^.^25^ Moreover, 8a can be transformed to 9a upon treatment with 2-chloroethylamine hydrochloride under basic conditions, which can be further converted into indole-fused CF_3_-piperazine derivative 10a through a Pictet–Spengler reaction according to the previous literature.^26,27^ In addition, a new pyrazino-indole derivative 11a was also synthesized in 76% yield in this paper as shown in Scheme 6. These pyrazino-indole derivatives have been recognized as antibacterial and antifungal agents in drug discovery.^28^

## Conclusions

In conclusion, we have explored a novel visible-light-induced photocatalytic synthetic methodology for the production of *N*-substituted indole products 2 through γ-fragmentation using acridinium salt PC 2 as the photocatalyst and *N*-sulfonated arylalkene derivatives 1 as substrates under mild conditions without any additional additive. The investigation on the substituent effects with regard to the sulfonyl leaving groups disclosed that the desulfonylative reaction initiates from the intramolecular nucleophilic attack of the nitrogen atom to the alkene moiety of the radical cationic species of 1. DFT calculations and kinetic experiments revealed that both cyclization (1a^.+^ to 5a) and SET (5a to 6a) processes are rate determining steps. A radical chain process was also involved in this reaction considering that the quantum yield is 1.77 and a light source is necessary. All of these results represent a robust and potentially useful strategy for the synthesis of indoles and will inspire further development in the synthesis of nitrogen atom-containing heterocycles under redox neutral conditions along with exploitation of the versatility of photoredox catalytic processes. The exploration of this photoinduced synthetic strategy in the synthesis of natural products or crucial pharmaceutical molecules and other complicated systems is currently under investigation.

## Conflicts of interest

There are no conflicts to declare.

## Data availability

Experimental and DFT computational data are available free of charge in ESI† section of this article.

## Author contributions

Shi, M. directed the project and revised the manuscript. Li, Q. Z. and Gu, X. T. wrote the manuscript and carried out the reactions. Wei, Y. checked the spectroscopic data and carried out the DFT calculations.

## Supplementary Material

SC-013-D2SC02822K-s001

SC-013-D2SC02822K-s002
