# Supplementary material for: Visible-light-induced indole synthesis via intramolecular C–N bond formation: desulfonylative C(sp2)–H functionalization
Source: Chem Sci. 2022 Sep 16;13(39):11623–32. doi: 10.1039/d2sc02822k (PMC9555724; doi:10.1039/d2sc02822k)
Supplement: SC-013-D2SC02822K-s001 [file SC-013-D2SC02822K-s001.pdf]

Electronic Supplementary Material (ESI) for Chemical Science.
This journal is © The Royal Society of Chemistry 2022

Supporting Information

Visible-Light-Induced Indole Synthesis via Intramolecular C-N Bond Formation: Desulfonylative C(sp^2)-H Functionalization

Quanzhe Li,[a] Xintao Gu,[a] Yin Wei*[a] and Min Shi*[a,b]

[a] *State Key Laboratory of Organometallic Chemistry, Center for Excellence in Molecular Synthesis, Shanghai Institute of Organic Chemistry, University of Chinese Academy of Science, Chinese Academy of Sciences, 345 Lingling Road, Shanghai 200032 China. E-mail: weiyin@sioc.ac.cn, mshi@mail.sioc.ac.cn.*

[b] *Key Laboratory for Advanced Materials and Institute of Fine Chemicals, School of Chemistry & Molecular Engineering, East China University of Science and Technology, 130 Meilong Road, Shanghai 200237, China. E-mail: mshi@mail.sioc.ac.cn.*

CONTENTS

1. General Remarks..S3

2. Optimization of Reaction Conditions...S4

3. Reaction Setup..S7

4. General Procedure for the Synthesis of Substrates..S8

5. General Procedure for the Synthesis of Products...S29

6. Mechanistic Investigations...S34

7. KIE Experiment...S60

8. Characterization Data of Substrates..S62

9. Characterization Data of Products...S192

10. Computational Studies..S268

11. X-ray Data...S281

12. References...S282

1. General Remarks

^{1}H NMR spectra were recorded on a Varian Mercury-400 spectrometer for solution in $CDCl_3$ with tetramethylsilane (TMS) as an internal standard; coupling constants *J* are given in Hz. ^{13}C NMR spectra were recorded on a Varian Mercury-400 spectrophotometers (101 MHz) with complete proton decoupling spectrophotometers ($CDCl_3$: 77.0 ppm). Mass 1gand HRMS spectra were recorded by ESI, EI, DART or FI method. Organic solvents used were dried by standard methods when necessary. Infrared spectra were recorded on a Perkin-Elmer PE-983 spectrometer with absorption in cm^{-1}. Melting points were determined on a digital melting point apparatus and temperatures were uncorrected. The reactions were carried out in oil bath. Commercially obtained reagents were used without further purification. All these reactions were monitored by TLC with silica gel coated plates. Flash column chromatography was carried out using silica gel at increased pressure.

2. Optimization of Reaction Conditions

Table S1: Optimization of the catalysts for the production of **2a**

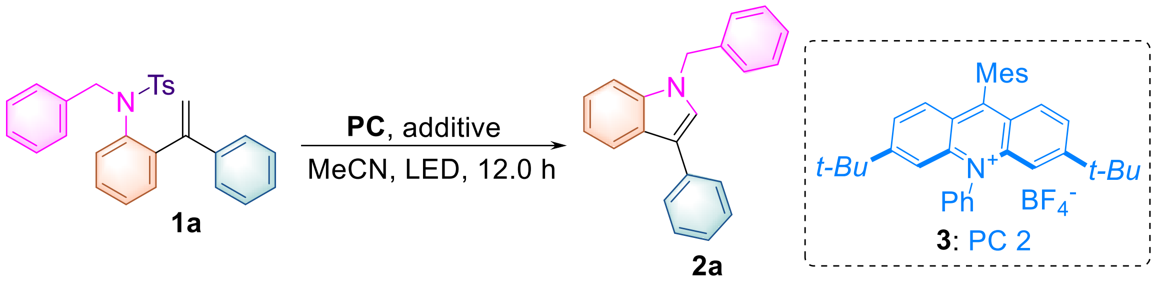

entry[a]	PC (5.0 mol%)	additive	LED	yield[b] (%)
1	(Ir[dFppy]$_2$(dtbpy))PF$_6$	-	365 nm	-
2	4CZIPN	-	365 nm	-
3	xanthone	-	365 nm	15
4	thioxanthone	-	365 nm	-
5	*fac*-Ir(ppy)$_3$	-	365 nm	-
6[c]	xanthone	Hantzsch ester	365 nm	-
7[c]	xanthone	DIPEA	365 nm	-
8	xanthone (dark)	-	365 nm	-
9	acetone	-	365 nm	<5
10	PC 1	-	365 nm	79
**11**	**PC 2**	**-**	**365 nm**	**88**
12	PC 3	-	365 nm	-
13	PC 4	-	365 nm	-
14	[Ir(dF(CF$_3$ppy)$_2$)(5,5′-CF$_3$-bpy)]PF$_6$	-	385 nm	39
15[c]	PC 2	Hantzsch ester	365 nm	-
16[c]	PC 2	DIPEA	365 nm	29

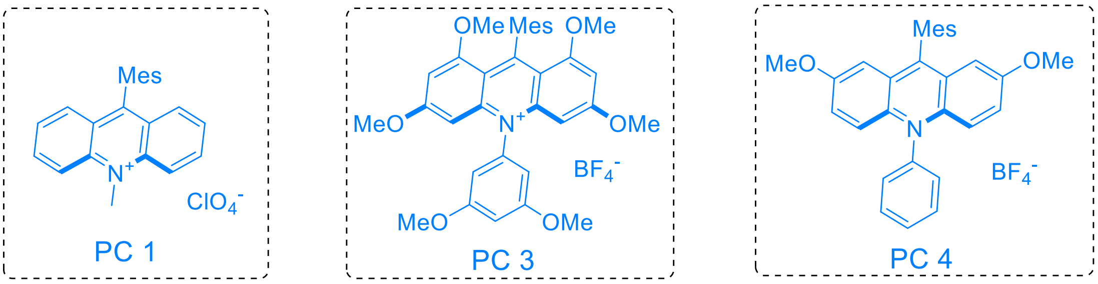

[a] Reaction conditions: **1a** (0.1 mmol) and **PC** (5.0 mol%) were added in degassed MeCN (2.0 mL) under Ar atmosphere for 12.0 h, in a sealed tube using 365 nm or 385 nm LED light irradiation. [b] NMR yield using 1,3,5-trimethoxybenzene as an internal standard. [c] Additive (0.2 mmol) was used.

Table S2: Optimization of the amount of **PC 2** and optimal light source for the production of **2a**

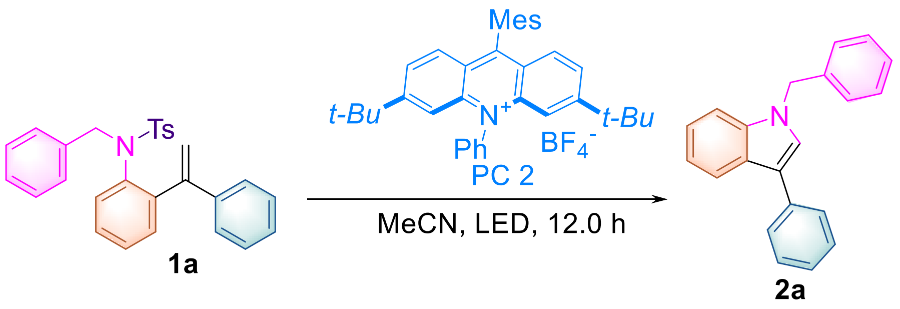

entry[a]	PC 2 (mol%)	LED	yield[b] (%)
1	5.0	365 nm	88
2	5.0	385 nm	90
3	5.0	blue (100 W)	88
4	5.0	blue (12 W)	-
5	1.0	385 nm	83
**6**	**2.0**	**385 nm**	**91 (90)[c]**
7	3.0	385 nm	81
8	4.0	385 nm	83
9	-	385 nm	-
10[d]	2.0	-	-

[a] Reaction conditions: **1a** (0.1 mmol) and **PC 2** were added in degassed MeCN (2.0 mL) under Ar atmosphere for 12.0 h, in a sealed tube using LED light irradiation. [b] NMR yield using 1,3,5-trimethoxybenzene as an internal standard. [c] Yield of the isolated products. [d] Under dark condition.

Table S3: Optimization of the solvents for the production of **2a**

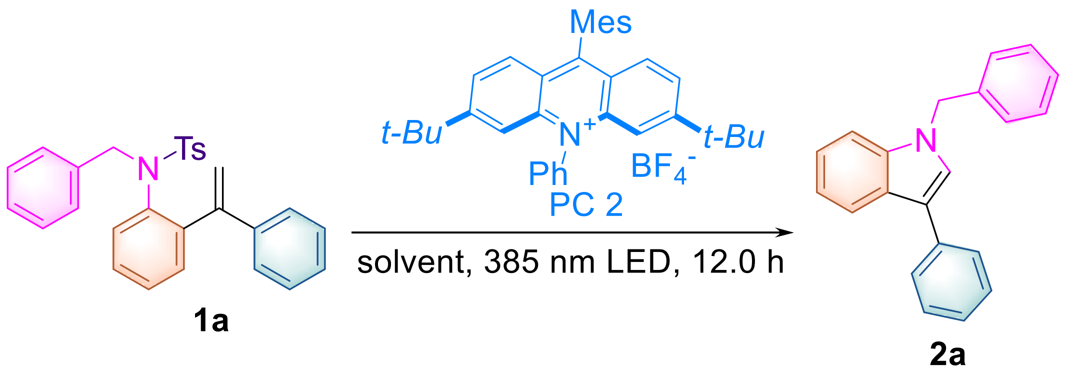

entry[a]	LED	yield[b] (%)
1	DCM	51
2	DCE	66
3	THF	<5
4	DMF	-
**5**	**MeCN**	**91 (90)[c]**

[a] Reaction conditions: **1a** (0.1 mmol) and **PC 2** (2.0 mol%) were added in degassed solvent (2.0 mL) under Ar atmosphere for 12.0 h, in a sealed tube using 385 nm LED light irradiation. [b] NMR yield using 1,3,5-trimethoxybenzene as an internal standard. [c] Yield of the isolated products.

3. Reaction Setup

Photoreactor scaled as 12 W 385 nm LED.

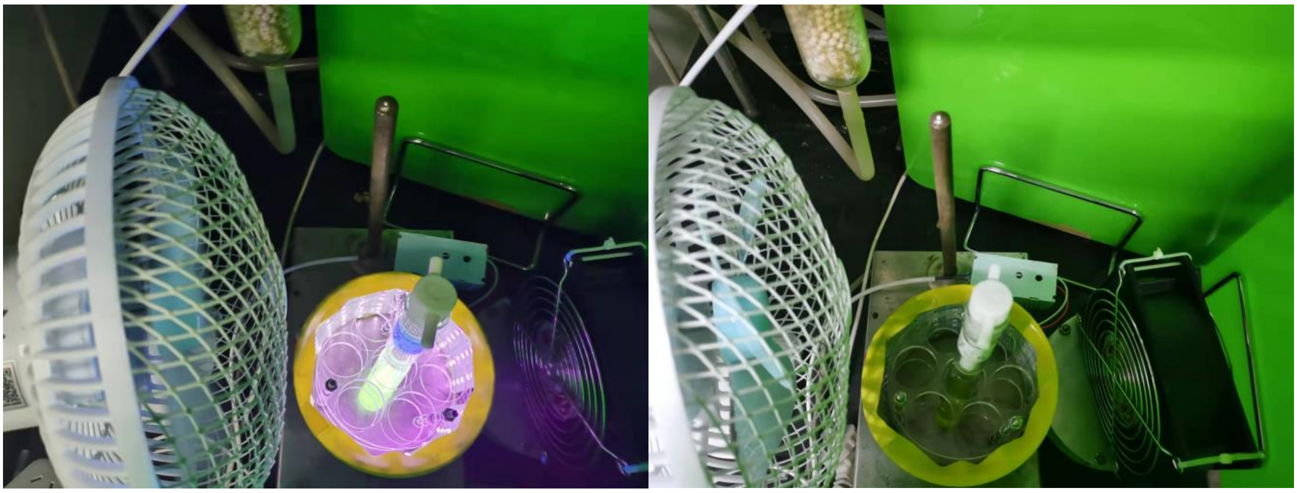

Photoreactor scaled as 100 W BLUE LED.

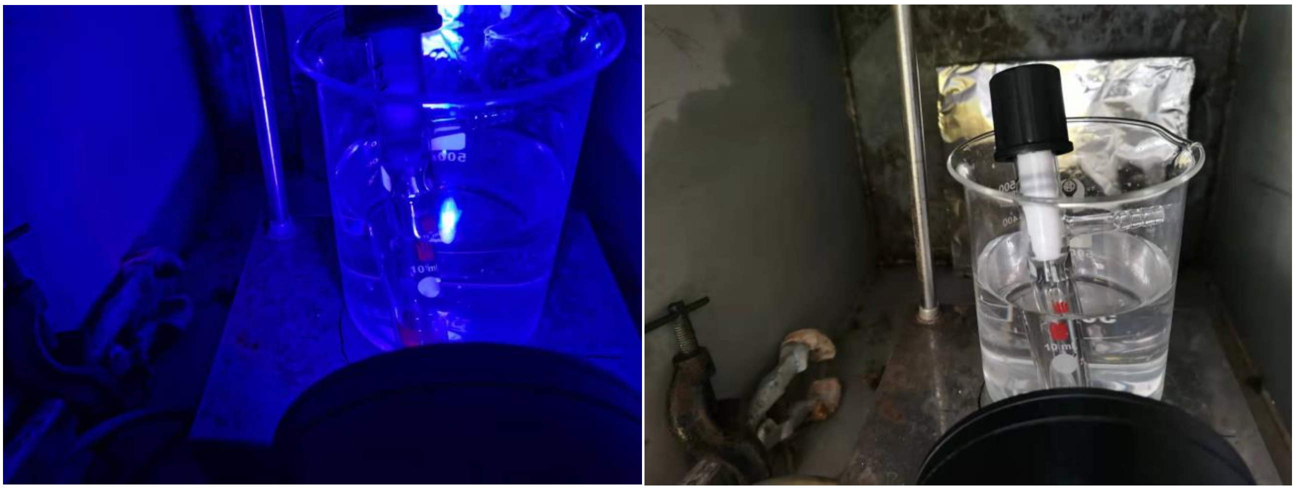

4. General Procedure for the Synthesis of Substrates

General procedure for the synthesis of compounds 1a-1m and 1t-1y

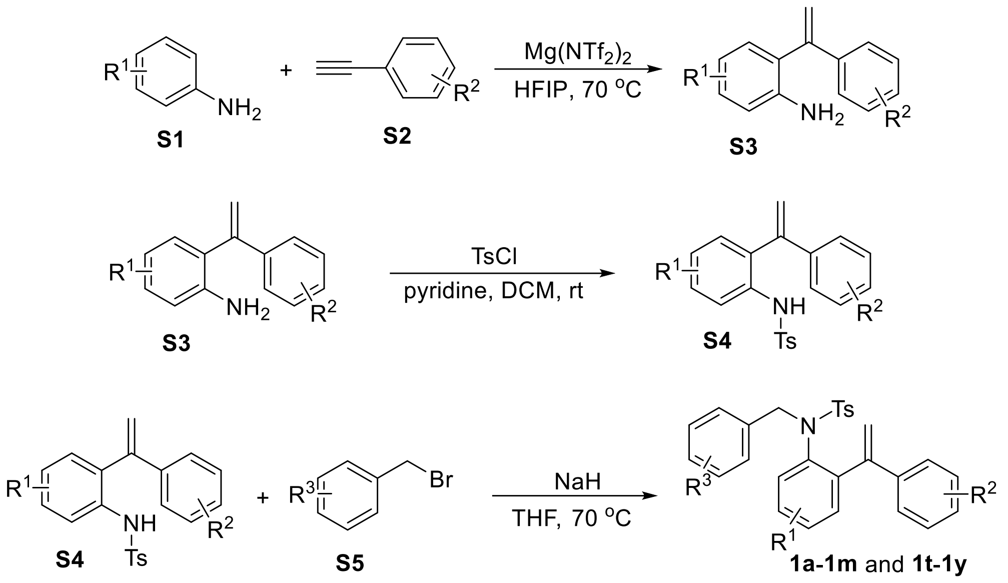

The procedure of preparing substrates **S3** was slightly modified according to the previous literature.[1]

To a stirred solution of **S1** (10.0 mmol, 1.0 equiv) and **S2** (12.0 mmol, 1.2 equiv) in HFIP (20.0 mL) was added $Mg(NTf_2)_2$ (5.0 mol%). The resulted mixture was stirred at 70 °C for 24.0 h. After the filtration and the removal of solvent under reduced pressure, the residue was purified by a column chromatography on silica gel (petroleum ether / ethyl acetate = 10 / 1) to afford the corresponding compounds **S3**.

To a solution of **S3** (1.0 equiv) in 20.0 mL of freshly distilled anhydrous DCM was added TsCl (1.2 equiv) and pyridine (1.2 equiv) at room temperature and the reaction mixture was stirred for 12.0 h. After the reaction completion monitored by TLC analysis, the residue was purified by a silica gel flash chromatography (eluent: petroleum ether / EtOAc = 10 / 1) to afford compounds **S4**.

To a solution of **S4** (1.0 equiv) in 20.0 mL of freshly distilled anhydrous THF was added NaH (1.2 equiv) at room temperature and the resulting mixture was stirred for 1.0 h and then was warmed up to 70 °C. The resulted mixture was added with **S5** benzyl bromide (1.5 equiv) and stirred for 12.0 h. After the reaction completion monitored by TLC analysis, the residue was purified by a silica gel flash chromatography (eluent: petroleum ether / EtOAc = 10 / 1) to afford compounds **1a-1m** and **1t-1y** in good yields ranging from 69% to 93%.

General procedure for the synthesis of compounds 1n-1r

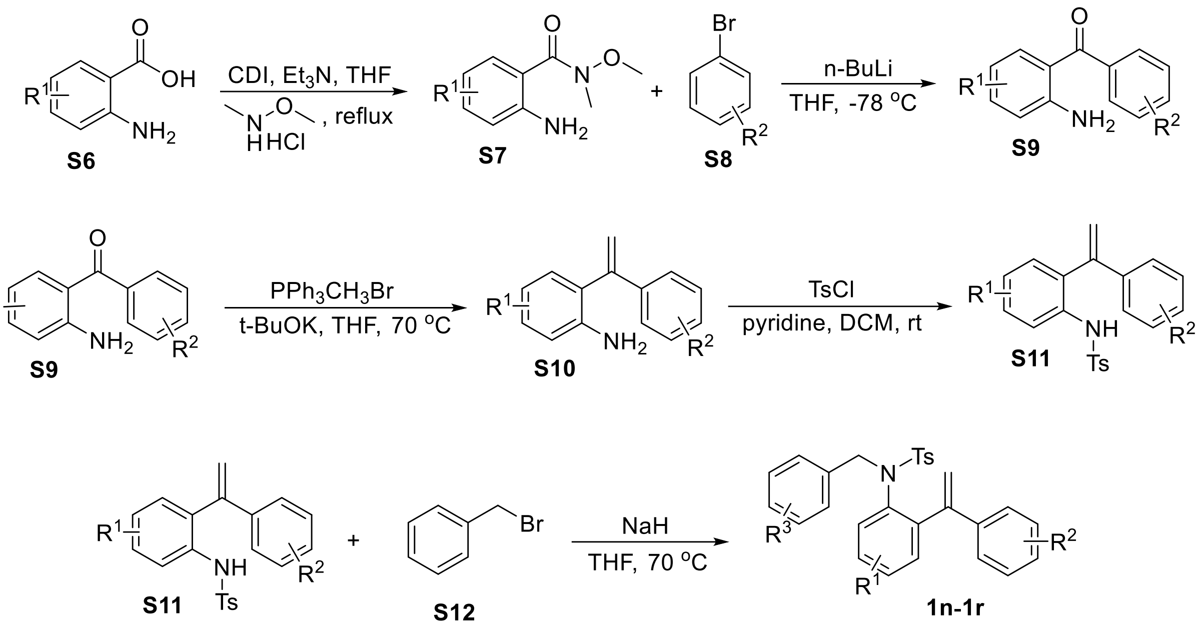

Compounds **S9** was prepared according to the previous literature.[2]

To a solution of **S6** (20.0 mmol, 1.0 equiv) and CDI (20.0 mmol, 1.0 equiv) in THF (extra dry, 20.0 mL) was stirred at room temperature for 2.0 h, and then a solution of *N*,*O*-dimethylhydroxylamine hydrochloride (20.0 mmol, 1.0 equiv) and NEt_3 (30.0 mmol, 1.5 equiv) in THF (extra dry, 20.0 mL) was added. The reaction mixture was stirred at 70 °C for another 22.0 h. After completion of the reaction, the mixture was poured onto an equal volume of ice and saturated Na_2CO_3 (50.0 mL). Next, the mixture was extracted with EA (3 x 30.0 mL), and the combined extracts were washed with water and brine, dried over anhydrous Na_2SO_4. After the solution was filtered and the solvent was evaporated under vacuum, the residue was purified by a flash column chromatograph on silica gel (eluent: petroleum ether / EtOAc = 1 / 1) to yield the products **S7**.

n-BuLi (2.0 equiv) was added slowly to a mixture of **S7** (1.0 equiv) and ArBr **S8** (1.0 equiv) in 20.0 mL extra dry THF over 1.0 h in a flamed-dried flask at -78 °C under the protection of Ar atmosphere upon stirring, and then 1.0 N HCl (32.0 mL, 2.0 equiv) was added carefully at -78 °C, then the mixture was extracted with EA (3 x 20.0 mL), and the combined extracts were washed with saturated Na_2CO_3, dried over anhydrous Na_2SO_4. After the solution was filtered and the solvent was evaporated under vacuum, the residue was purified by a flash column chromatograph on silica gel (eluent: petroleum ether / EtOAc = 10 / 1) to yield the products **S9**.

A solution of PPh_3CH_3Br (1.2 equiv) and t-BuOK (1.2 equiv) in THF (10.0 mL) was stirred at 70 ^oC under argon atmosphere for 0.5 h. Afterwards, compounds **S9** (1.0 equiv) in THF (30.0 mL) was added and the reaction solution was stirred at 70 ^oC for another 12.0 h. Upon completion, the reaction was cooled to room temperature and the mixture was filtered through a celite. The filtrate was concentrated under reduced pressure and the residue was purified by a silica gel flash chromatography (eluent: petroleum ether / EtOAc = 20 / 1) to afford compounds **S10**.

To a solution of **S10** (1.0 equiv) in 20.0 mL of freshly distilled anhydrous DCM was added TsCl (1.2 equiv) and pyridine (1.2 equiv) at room temperature and the resulting mixture was stirred for 12.0 h. After the reaction completion monitored by TLC analysis, the solvent was removed under reduced pressure and the residue was purified by a silica gel flash chromatography (eluent: petroleum ether / EtOAc = 10 / 1) to afford compounds **S11**.

To a solution of **S11** (1.0 equiv) in 20.0 mL of freshly distilled anhydrous THF was added NaH (1.2 equiv) at room temperature and the resulting mixture was stirred for 1.0 h and then warmed up to 70 ^oC. The resulted mixture was added with **S12** BnBr (1.5 equiv) for 12.0 h. After the reaction completion monitored by TLC analysis, the solvent was removed under reduced pressure and the residue was purified by a silica gel flash chromatography (eluent: petroleum ether / EtOAc = 10 / 1) to afford compounds **1n**-**1r** in good yields ranging from 80% to 91%.

General procedure for the synthesis of compounds 1s, 1z-1ai and 1ak

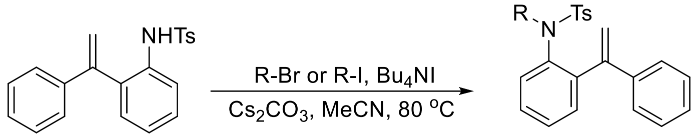

S13 **1s**, **1z-1ai** and **1ak**

To a solution of **S13** (3.0 mmol, 1.0 equiv) in 20.0 mL of freshly distilled anhydrous MeCN was added R-Br or R-I (6.0 mmol, 2.0 equiv), Cs_2CO_3 (6.0 mmol, 2.0 equiv) and Bu_4NI (0.3 mmol, 0.1 equiv) and the resulting mixture was stirred at 80 °C for 12.0 h. After the reaction completion monitored by TLC analysis, the solvent was removed under reduced pressure and the residue was purified by a silica gel flash chromatography (eluent: petroleum ether / EtOAc = 10 / 1) to afford compounds **1s**, **1z-1ai** and **1ak** in good yields ranging from 71% to 94%.

General procedure for the synthesis of compounds 1aj and 1al

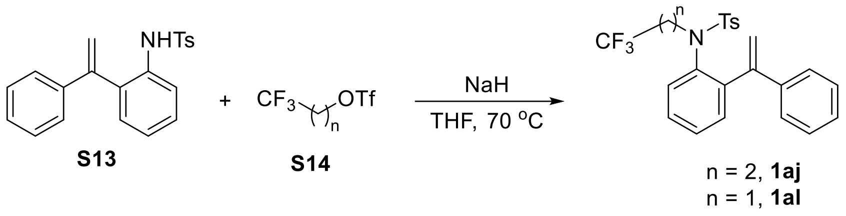

To a solution of **S13** (3.0 mmol, 1.0 equiv) in 20.0 mL of freshly distilled anhydrous THF was added NaH (3.6 mmol, 1.2 equiv) at room temperature for 1.0 h and the resulting mixture was warmed up to 70 °C. The resulted mixture was added with **S14** (4.5 mmol, 1.5 equiv) for 12.0 h. After the reaction completion monitored by TLC analysis, the solvent was removed under reduced pressure and the residue was purified by a silica gel flash chromatography (eluent: petroleum ether / EtOAc = 10 / 1) to afford compounds **1aj** and **1al** in 77% and 84% yields, respectively.

General procedure for the synthesis of compound 1am

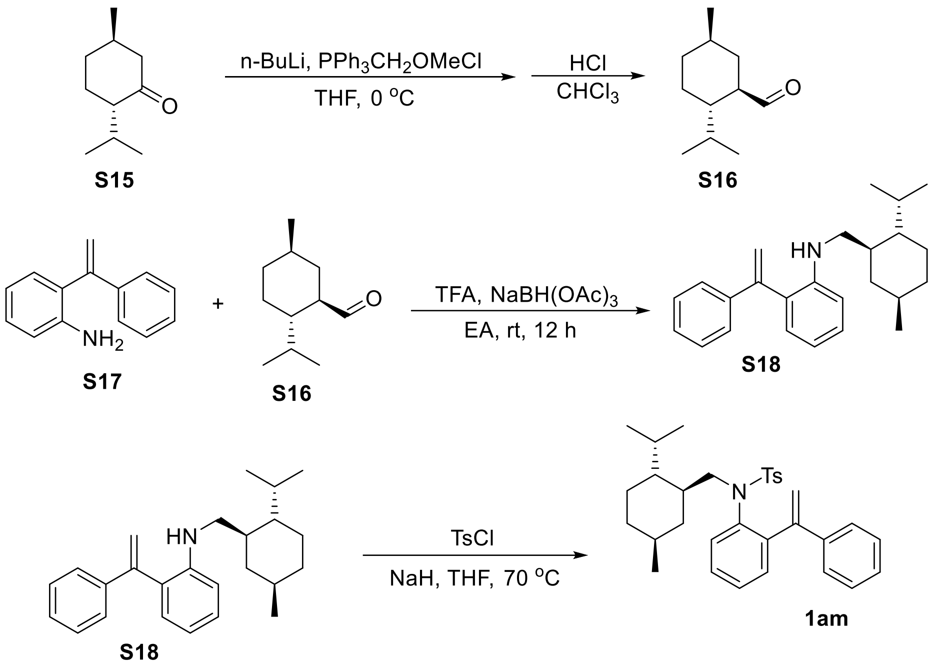

The procedure of preparing substrate **S18** was slightly modified according to the previous literature.[6,7]

A solution of PPh_3CH_2OMeCl (12.0 mmol 1.2 equiv) in THF (20.0 mL) was added n-BuLi (12.0 mmol, 1.2 equiv) dropwise at 0 °C under argon atmosphere for 0.5 h and the resulting mixtures was stirred for another 1.0 h. Afterwards compound **S15** (10.0 mmol, 1.0 equiv) in THF (10.0 mL) was added and the reaction solution was stirred at room temperature for overnight. The reaction mixture was neutralized by adding ice cold 1.0 N hydrochloric acid (35.0 mL) at 0 °C and the aqueous phase was extracted with ether (3 x 15.0 mL). The combined organic extracts were washed with water, brine, dried over $MgSO_4$ and concentrated to approximately 10.0 mL. A white solid (triphenylphosphine oxide) precipitated, and these solids were filtered and washed with hexanes. The filtrate was concentrated in vacuo, and the residue was re-dissolved in chloroform (20.0 mL) and HCl (12.0 N, 3.0 mL) was added dropwise at 0 °C. The resulting mixture was stirred at room temperature for 4.0 h (monitored by TLC). The chloroform was evaporated from the mixture, and the residue was diluted with water (15.0 mL) and extracted with diethyl ether (3 x 15.0 mL). The combined organic extracts were washed with brine (20.0 mL), dried over $MgSO_4$, filtered and concentrated under reduced pressure. The crude compound was purified by a silica gel flash

column chromatography (eluent: petroleum ether / EtOAc = 40 / 1) to afford compound **S16** in 74% yield.

The 2-aminostyrene **S17** (1.0 equiv) was dissolved in ethyl acetate (0.25 M). The aldehyde **S16** (1.5 equiv) was added and followed by adding trifluoroacetic acid (2.0 equiv). The resulting mixture was stirred for 0.5 h and then sodium triacetoxyborohydride (2.0 equiv) was added. The mixture was stirred for 2.0 h and then the reduction was quenched with 4.0 M NaOH aqueous solution. The reaction mixture was diluted with ethyl acetate and washed twice with brine. The organic layer was dried over anhydrous magnesium sulfate, filtered, and concentrated under reduced pressure. The residue was purified by a flash column chromatography (eluent: petroleum ether / EtOAc = 10 / 1) to afford compound **S18** in 55% yield.

To a solution of **S18** (1.0 equiv) in 20.0 mL of freshly distilled anhydrous THF was added NaH (1.2 equiv) at room temperature for 1.0 h upon stirring and then was warmed up to 70 ^{o}C. The resulted mixture was added with TsCl and the resulting mixture was stirred for 12.0 h. After the reaction completion monitored by TLC analysis, the solvent was removed under reduced pressure and the residue was purified by a silica gel flash chromatography (eluent: petroleum ether / EtOAc = 10 / 1) to afford compound **1am** in 70% yield.

General procedure for the synthesis of compound 1an

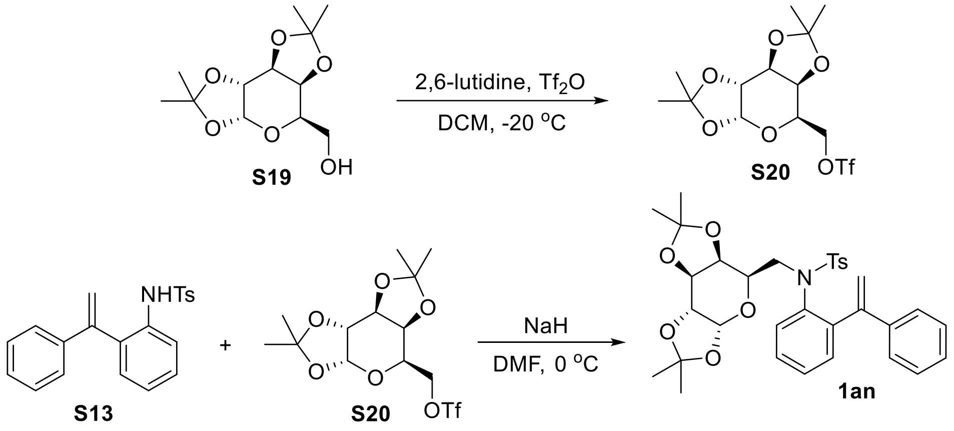

The procedure of preparing substrate **S20** was slightly modified according to the previous literature.[8]

Under argon atmosphere, to a solution of 2,6-lutidine (15.0 mmol, 1.5 equiv) in dry dichloromethane (35.0 mL) was added trifluoromethanesulfonic anhydride (15.0 mmol, 1.5 equiv) at -20 °C. After 5.0 min, a solution of 1,2:3,4-di-*O*-isopropylidene-α-d-galactopyranose **S19** (10.0 mmol, 1.0 equiv) in dichloromethane (40.0 mL) was added dropwise to the solution at -20 °C. The resulting solution was stirred at -20 °C for 5.0 min, and then at 0 °C for another 30.0 min. The mixture was poured into ice-cooled water and extracted with dichloromethane. The solvent was removed under reduced pressure and the residue was purified on a silica gel column chromatography (eluent: petroleum ether / EtOAc = 10 / 1) to give compound **S20** as a colorless oil in 94% yield.

To a solution of **S13** (1.0 equiv) in 20.0 mL of anhydrous DMF was added NaH (1.2 equiv) at 0 °C for 1.0 h. Afterwards compound **S20** (1.0 equiv) in DMF (10.0 mL) was added dropwise at 0 °C for 1.0 h. The resulted mixture was stirred for overnight at 0 °C. After the reaction completion monitored by TLC analysis, the mixture was filtered and washed with DCM. The filtrate was concentrated in vacuo and extracted with DCM (3 x 15.0 mL). After above operation, the residue was purified by a silica gel flash chromatography (eluent: petroleum ether / EtOAc = 5 / 1) to afford compound **1an** in 51% yield.

General procedure for the synthesis of compound 1ao

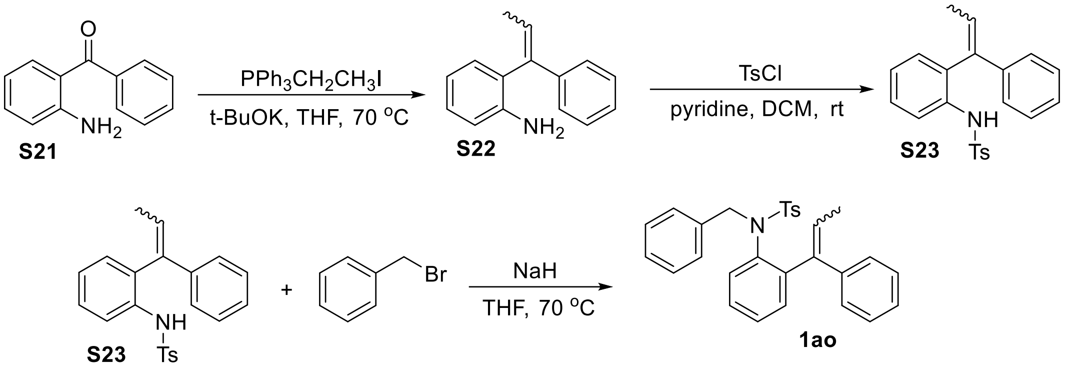

A solution of $PPh_3CH_2CH_3I$ (10.0 mmol, 1.0 equiv) and t-BuOK (12.0 mmol, 1.2 equiv) in THF (10.0 mL) was stirred at 70 °C under argon atmosphere for 0.5 h. Afterwards compound **S21** (10.0 mmol, 1.0 equiv) in THF (10.0 mL) was added and the reaction solution was stirred at 70 °C for another 1.0 h. Upon completion, the reaction was cooled to room temperature and the mixture was filtered through a celite. The filtrate was concentrated under reduced pressure and the residue was purified by a silica gel flash chromatography (eluent: petroleum ether / EtOAc = 10 / 1) to afford compound **S22** in 65% yield.

To a solution of **S22** (1.0 equiv) in 20.0 mL of freshly distilled anhydrous DCM was added TsCl (1.2 equiv) and pyridine (1.2 equiv) at room temperature and the resulting mixture was stirred for 12.0 h. After the reaction completion monitored by TLC analysis, the solvent was removed under reduced pressure and the residue was purified by a silica gel flash chromatography (eluent: petroleum ether / EtOAc = 10 / 1) to afford compound **S23** in 93% yield.

To a solution of **S23** (1.0 equiv) in 20.0 mL of freshly distilled anhydrous THF was added NaH (1.2 equiv) at room temperature for 1.0 h and the mixture was warmed up to 70 °C. The resulted mixture was added with BnBr (1.5 equiv) and was stirred for 12.0 h. After the reaction completion monitored by TLC analysis, the solvent was removed under reduced pressure and the residue was purified by a silica gel flash chromatography (eluent: petroleum ether / EtOAc = 10 / 1) to afford compound **1ao** in 81% yield.

General procedure for the synthesis of compound 1ap

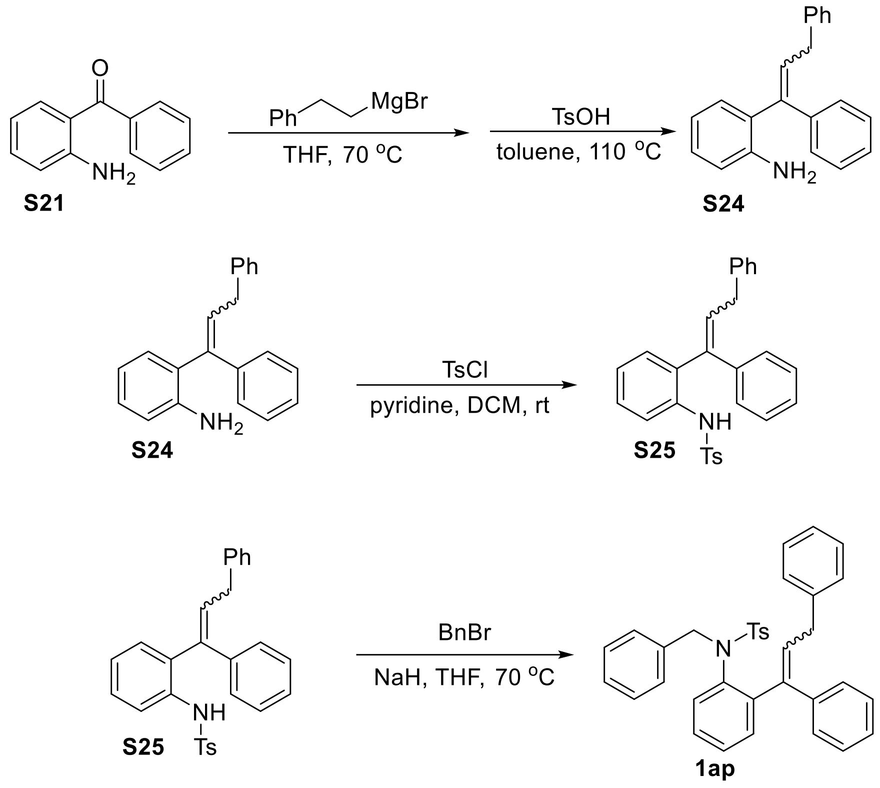

Compound **S24** was prepared according to the previous literature.[3]

To a solution of **S21** (10.0 mmol, 1.0 equiv) in 30.0 mL of freshly distilled anhydrous THF was added dropwise $Ph(CH_2)_2MgBr$ (30.0 mmol, 3.0 equiv) at room temperature over 1.0 h. The mixture was stirred at 70 °C for 24.0 h. After the reaction completion monitored by TLC analysis, the residue was added with 1.0 N HCl (30.0 mL), and then the resulting mixture was stirred for 1.0 h and then was extracted with EA (3 x 20.0 mL), and the combined extracts were washed with saturated Na_2CO_3, dried over anhydrous Na_2SO_4. A reaction mixture of the above crude tertiary alcohol and *p*-TsOH (2.0 mmol, 0.2 equiv) in toluene (30.0 mL) was refluxed for 24.0 h. After cooling to room temperature, it was washed with H_2O (3 × 15.0 mL) and brine (2 × 15.0 mL) in sequence, and then extracted with EtOAc (3 × 15.0 mL). The combined organic layer was dried over anhydrous Na_2SO_4, concentrated, and purified by a silica gel flash chromatography (eluent: petroleum ether / EtOAc = 10 / 1) to afford compound **S24** in 52% yield.

To a solution of **S24** (1.0 equiv) in 20.0 mL of freshly distilled anhydrous DCM was added TsCl (1.2 equiv) and pyridine (1.2 equiv) at room temperature for 12.0 h upon stirring. After the reaction completion monitored by TLC analysis, the solvent was removed under reduced pressure

and the residue was purified by a silica gel flash chromatography (eluent: petroleum ether / EtOAc = 10 / 1) to afford compound **S25** in 85% yield.

To a solution of **S25** (1.0 equiv) in 20.0 mL of freshly distilled anhydrous THF was added NaH (1.2 equiv) at room temperature and was stirred for 1.0 h and then was warmed up to 70 ^{o}C. The resulted mixture was added with BnBr (1.5 equiv) and was stirred for 12.0 h. After the reaction completion monitored by TLC analysis, the solvent was removed under reduced pressure and the residue was purified by a silica gel flash chromatography (eluent: petroleum ether / EtOAc = 10 / 1) to afford compound **1ap** in 79% yield.

General procedure for the synthesis of compounds 1aw-1aag

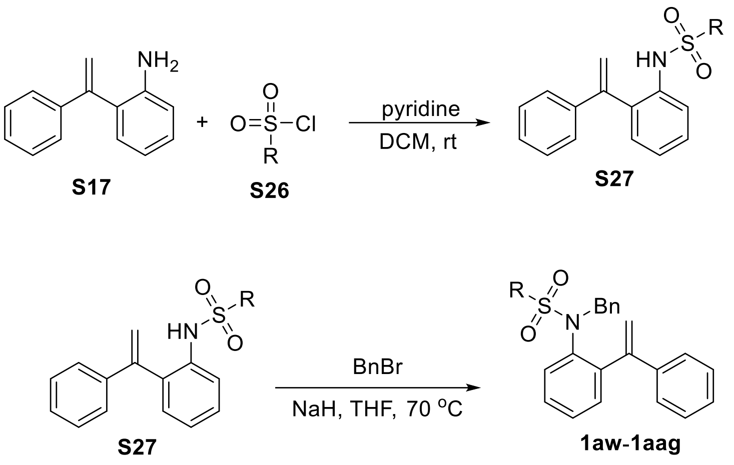

To a solution of **S17** (10.0 mmol, 1.0 equiv) in 20.0 mL of freshly distilled anhydrous DCM was added **S26** (12.0 mmol, 1.2 equiv) and pyridine (12.0 mmol, 1.2 equiv) at room temperature and the resulting mixture was stirred for 12.0 h. After the reaction completion monitored by TLC analysis, the solvent was removed under reduced pressure and the residue was purified by a silica gel flash chromatography (eluent: petroleum ether / EtOAc = 10 / 1) to afford compounds **S27**.

To a solution of **S27** (1.0 equiv) in 20.0 mL of freshly distilled anhydrous THF was added NaH (1.2 equiv) at room temperature for 1.0 h and the mixture was warmed up to 70 ^{o}C. The resulted mixture was added with BnBr (1.5 equiv) and was stirred for 12.0 h. After the reaction completion monitored by TLC analysis, the solvent was removed under reduced pressure and the residue was purified by a silica gel flash chromatography (eluent: petroleum ether / EtOAc = 10 / 1) to afford compounds **1aw-1aag** in good yields ranging from 60% to 90%.

General procedure for the synthesis of compounds 1aah-1aak

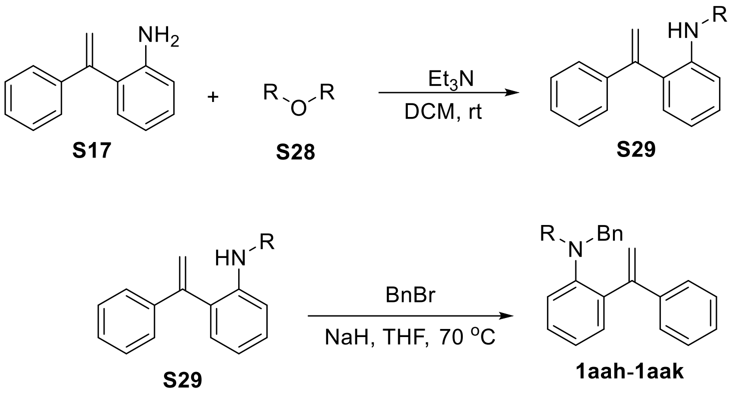

To a solution of **S17** (5.0 mmol, 1.0 equiv) and Et_3N (7.5 mmol, 1.5 equiv) in 20.0 mL of freshly distilled anhydrous DCM was added **S28** (7.5 mmol, 1.5 equiv) dropwise at room temperature for 20.0 min. The mixture was stirred for another 12.0 h. After the reaction completion monitored by TLC analysis, the solvent was removed under reduced pressure and the residue was purified by a silica gel flash chromatography (eluent: petroleum ether / EtOAc = 10 / 1) to afford compounds **S29**.

To a solution of **S29** (1.0 equiv) in 20.0 mL of freshly distilled anhydrous THF was added NaH (1.2 equiv) at room temperature for 1.0 h and the mixture was warmed up to 70 ^{o}C. The resulted mixture was added with BnBr (1.5 equiv) and was stirred for 12.0 h. After the reaction completion monitored by TLC analysis, the solvent was removed under reduced pressure and the residue was purified by a silica gel flash chromatography (eluent: petroleum ether / EtOAc = 10 / 1) to afford compounds **1aah-1aak** in good yields ranging from 67% to 90%.

General procedure for the synthesis of compounds 1aq and 1as

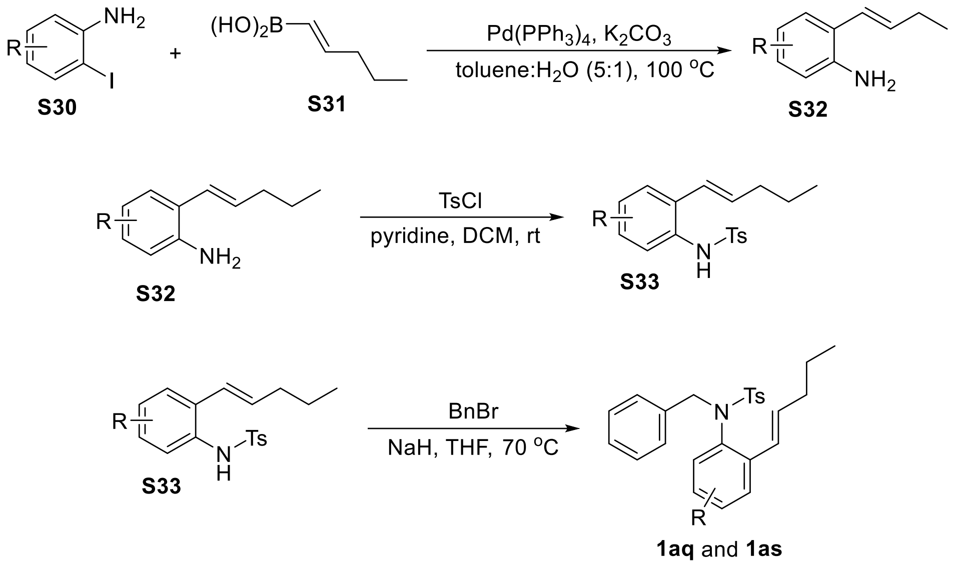

The procedure of preparing substrates **S32** was slightly modified according to the previous literature.[5]

Compounds **S30** (10.0 mmol, 1.0 equiv), **S31** (15.0 mmol, 1.5 equiv), $Pd(PPh_3)_4$ (10.0 mol%) and K_2CO_3 (30.0 mmol, 3.0 equiv) were mixed with toluene (50.0 mL) and H_2O (10.0 mL). The resulting reaction mixture was stirred for 24.0 h at 100 °C. After completion of the reaction, the solution was concentrated in vacuo and the residue was purified by a column chromatography on silica gel (eluent: petroleum ether / EtOAc = 8 / 1) to give the desired products **S32**.

To a solution of **S32** (1.0 equiv) in 20.0 mL of freshly distilled anhydrous DCM was added TsCl (1.2 equiv) and pyridine (1.2 equiv) at room temperature and the mixture was stirred for 12.0 h. After the reaction completion monitored by TLC analysis, the solvent was removed under reduced pressure and the residue was purified by a silica gel flash chromatography (eluent: petroleum ether / EtOAc = 10 / 1) to afford compounds **S33**.

To a solution of **S33** (1.0 equiv) in 20.0 mL of freshly distilled anhydrous THF was added NaH (1.2 equiv) at room temperature and the resulting mixture was stirred for 1.0 h and then was warmed up to 70 °C. The resulted mixture was added with BnBr (1.5 equiv) and was stirred for 12.0 h. After the reaction completion monitored by TLC analysis, the solvent was removed under reduced pressure and the residue was purified by a silica gel flash chromatography (eluent: petroleum ether / EtOAc = 5 / 1) to afford compounds **1aq** and **1as** in 92% and 90% yields, respectively.

General procedure for the synthesis of compound 1ar

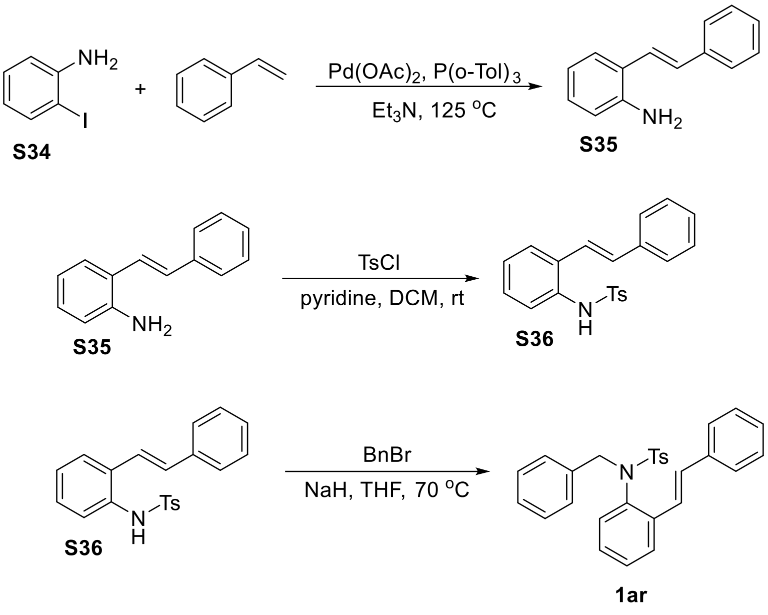

The procedure of preparing substrate **S35** was slightly modified according to the previous literature.[9]

The reaction mixture of compound **S34** (10.0 mmol, 1.0 equiv), styrene (15.0 mmol, 1.5 equiv), $Pd(OAc)_2$ (10.0 mol%), P(*o*-Tol)$_3$ (20.0 mol%) and Et_3N (50.0 mL) was stirred for 24.0 h at 125 °C. After completion of the reaction, the solution was concentrated in vacuo and the residue was purified by a column chromatography on silica gel (eluent: petroleum ether / EtOAc = 8 / 1) to give the desired product **S35** in 66% yield.

To a solution of **S35** (1.0 equiv) in 20.0 mL of freshly distilled anhydrous DCM was added TsCl (1.2 equiv) and pyridine (1.2 equiv) at room temperature and the resulting mixture was stirred for 12.0 h. After the reaction completion monitored by TLC analysis, the solvent was removed under reduced pressure and the residue was purified by a silica gel flash chromatography (eluent: petroleum ether / EtOAc = 10 / 1) to afford compound **S36** in 87% yield.

To a solution of **S36** (1.0 equiv) in 20.0 mL of freshly distilled anhydrous THF was added NaH (1.2 equiv) at room temperature for 1.0 h and the mixture was warmed up to 70 ^{o}C. The resulted mixture was added with BnBr (1.5 equiv) and was stirred for 12.0 h. After the reaction completion monitored by TLC analysis, the solvent was removed under reduced pressure and the residue was purified by a silica gel flash chromatography (eluent: petroleum ether / EtOAc = 5 / 1) to afford compound **1ar** in 81% yield.

General procedure for the synthesis of compounds 1at and 1au

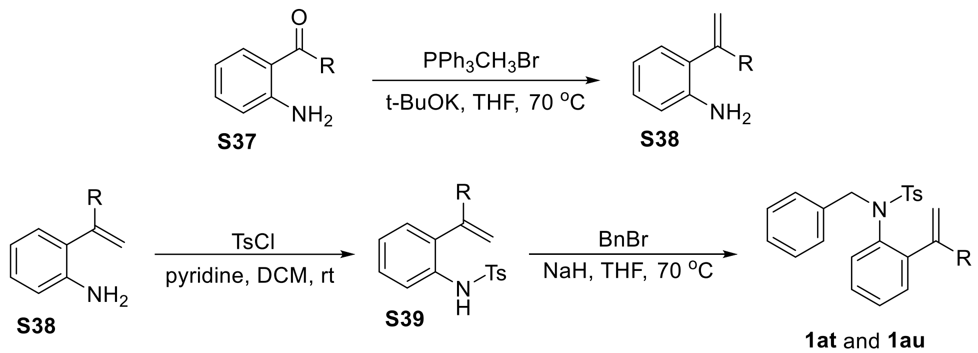

1at and **1au**

A solution of PPh_3CH_3Br (12.0 mmol, 1.2 equiv) and t-BuOK (12.0 mmol, 1.2 equiv) in THF (10.0 mL) was stirred at 70 ^oC under argon atmosphere for 0.5 h. Afterwards compounds **S37** (10.0 mmol, 1.0 equiv) in THF (10.0 mL) was added and the reaction solution was stirred at 70 ^oC for another 1.0 h. Upon completion, the reaction was cooled to room temperature and the mixture was filtered through a celite. The filtrate was concentrated under reduced pressure and the residue was purified by a silica gel flash chromatography (eluent: petroleum ether / EtOAc = 10 / 1) to afford compounds **S38**.

To a solution of **S38** (1.0 equiv) in 20.0 mL of freshly distilled anhydrous DCM was added TsCl (1.2 equiv) and pyridine (1.2 equiv) at room temperature and the resulting mixture was stirred for 12.0 h. After the reaction completion monitored by TLC analysis, the solvent was removed under reduced pressure and the residue was purified by a silica gel flash chromatography (eluent: petroleum ether / EtOAc = 5 / 1) to afford compounds **S39**.

To a solution of **S39** (1.0 equiv) in 20.0 mL of freshly distilled anhydrous THF was added NaH (1.2 equiv) at room temperature for 1.0 h and the mixture was warmed up to 70 ^oC. The resulted mixture was added with BnBr (1.5 equiv) and was stirred for 12.0 h. After the reaction completion monitored by TLC analysis, the solvent was removed under reduced pressure and the residue was purified by a silica gel flash chromatography (eluent: petroleum ether / EtOAc = 10 / 1) to afford compounds **1at** and **1au** in 77% and 90% yields, respectively.

General procedure for the synthesis of compound 1av

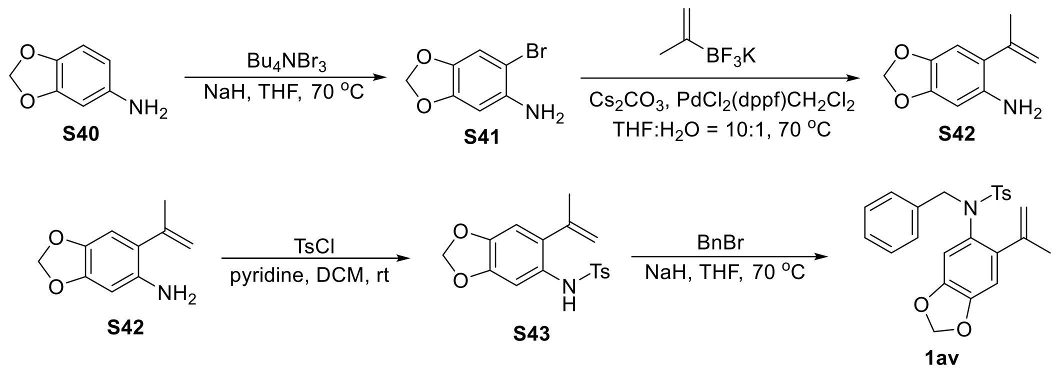

The procedure of preparing substrate **S42** was slightly modified according to the previous literature.[4]

Compound **S40** (10.0 mmol, 1.0 equiv), Bu_4NBr_3 (30.0 mmol, 3.0 equiv) and NaH (30.0 mmol, 3.0 equiv) were added in THF (40.0 mL). The reaction mixture was stirred for 24.0 h at 70 °C. After completion of the reaction, the solution was concentrated in vacuo and purified by a column chromatography on silica gel (eluent: petroleum ether / DCM / EtOAc = 30 / 1 / 1) to give the desired product **S41** in 50% yield.

Compound **S41** (1.0 equiv), potassium isopropenyltrifluoroborate (1.1 equiv), and $PdCl_2(dppf)CH_2Cl_2$ (10.0 mol%), Cs_2CO_3 (3.0 equiv) were mixed with THF (20.0 mL) and H_2O (2.0 mL) solvent. The reaction mixture was stirred for 24.0 h at 70 °C. After completion of the reaction, the organic phase was extracted with EtOAc (3 × 15.0 mL) and then the solution was concentrated in vacuo and purified by a column chromatography on silica gel (eluent: petroleum ether / EtOAc = 8 / 1) to give the desired product **S42** in 71% yield.

To a solution of **S42** (1.0 equiv) in 20.0 mL of freshly distilled anhydrous DCM was added TsCl (1.2 equiv) and pyridine (1.2 equiv) at room temperature and the resulting mixture was stirred for 12.0 h. After the reaction completion monitored by TLC analysis, the solvent was removed under reduced pressure and the residue was purified by a silica gel flash chromatography (eluent: petroleum ether / EtOAc = 5 / 1) to afford compound **S43** in 90% yield.

To a solution of **S43** (1.0 equiv) in 20.0 mL of freshly distilled anhydrous THF was added NaH (1.2 equiv) at room temperature for 1.0 h and then was warmed up to 70 ^{o}C. The resulted mixture was added with BnBr (1.5 equiv) and was stirred for 12.0 h. After the reaction completion

monitored by TLC analysis, the solvent was removed under reduced pressure and the residue was purified by a silica gel flash chromatography (eluent: petroleum ether / EtOAc = 5 / 1) to afford compound **1av** in 80% yield.

General procedure for the synthesis of compound [D_2]-1a

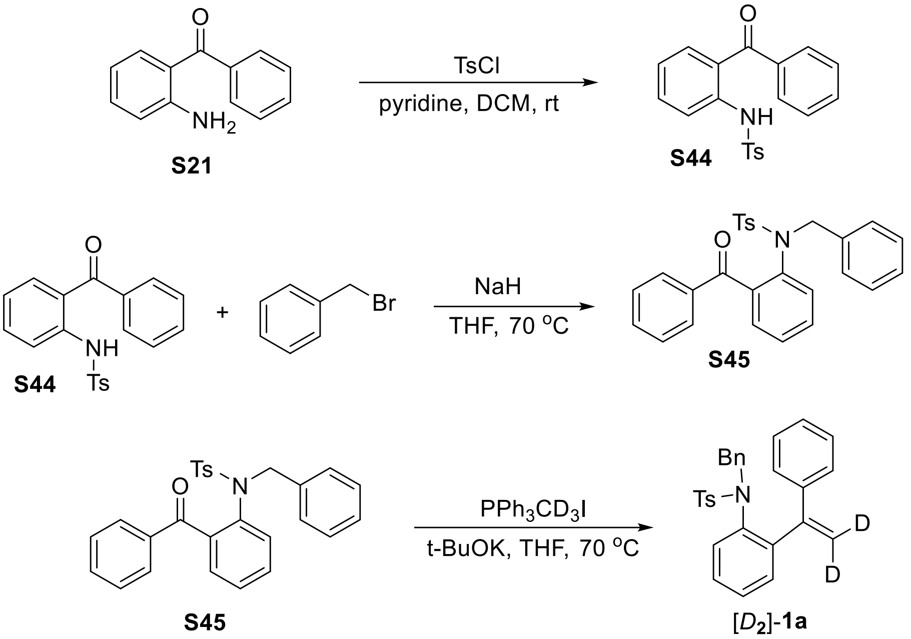

To a solution of **S21** (10.0 mmol, 1.0 equiv) in 20.0 mL of freshly distilled anhydrous DCM was added TsCl (12.0 mmol, 1.2 equiv) and pyridine (12.0 mmol, 1.2 equiv) at room temperature and the resulting mixture was stirred for 12.0 h. After the reaction completion monitored by TLC analysis, the solvent was removed under reduced pressure and the residue was purified by a silica gel flash chromatography (eluent: petroleum ether / EtOAc = 8 / 1) to afford compound **S44** in 75% yield.

To a solution of **S44** (1.0 equiv) in 20.0 mL of freshly distilled anhydrous THF was added NaH (1.2 equiv) at room temperature and the resulting mixture was stirred for 1.0 h and then was warmed up to 70 ^oC. The resulted mixture was added with BnBr (1.5 equiv) and was stirred for 12.0 h. After the reaction completion monitored by TLC analysis, the solvent was removed under reduced pressure and the residue was purified by a silica gel flash chromatography (eluent: petroleum ether / EtOAc = 8 / 1) to afford compound **S45** in 92% yield.

A solution of PPh_3CD_3I (1.2 equiv) and t-BuOK (1.2 equiv) in THF (20.0 mL) was stirred at 70 ^oC under argon atmosphere for 0.5 h. Afterwards, compound **S45** (1.0 equiv) in THF (20.0 mL) was added and the reaction solution was stirred at 70 ^oC for another 1.0 h. Upon completion, the reaction was cooled to room temperature and the mixture was filtered through a celite. The filtrate was concentrated under reduced pressure and the residue was purified by a silica gel flash

chromatography (eluent: petroleum ether / EtOAc = 10 / 1) to afford compound [D_2]-**1a** in 75% yield and the D containing content was 93%.

General procedure for the synthesis of compounds 1aal

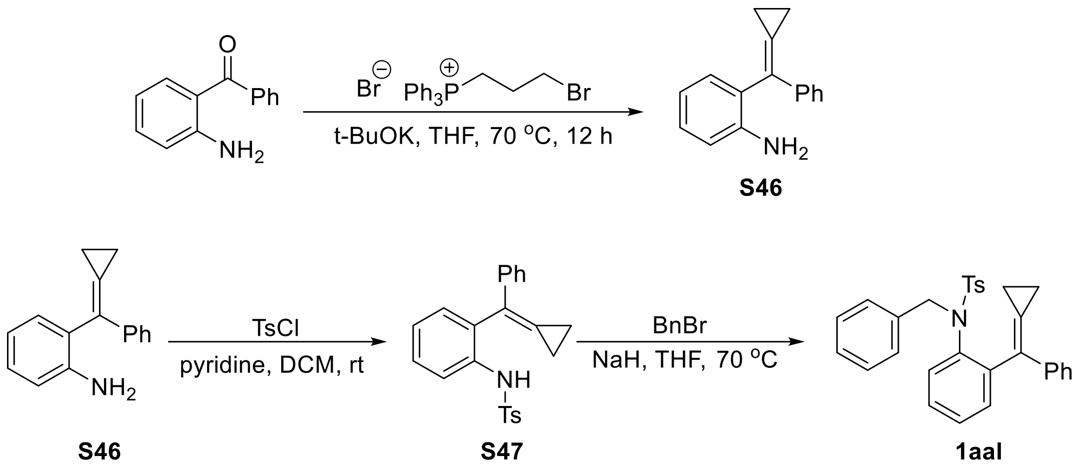

A solution of (3-bromopropyl)triphenylphosphinium bromide (12.0 mmol, 1.2 equiv) and *tert*-BuOK (12.0 mmol, 1.2 equiv) in THF (10.0 mL) was stirred at 70 °C under argon atmosphere for 0.5 h. Afterwards compound (2-aminophenyl)(phenyl)methanon (10.0 mmol, 1.0 equiv) in THF (10.0 mL) was added and the reaction solution was stirred at 70 °C for another 12.0 h. Upon completion, the reaction was cooled to room temperature and the mixture was filtered through a celite. The filtrate was concentrated under reduced pressure and the residue was purified by a silica gel flash chromatography (eluent: petroleum ether / EtOAc = 20 / 1) to afford compounds **S46** in 58% yield.

To a solution of **S46** (5.0 mmol, 1.0 equiv) in 20.0 mL of freshly distilled anhydrous DCM was added TsCl (6.0 mmol, 1.2 equiv) and pyridine (6.0 mmol, 1.2 equiv) at room temperature and the resulting mixture was stirred for 12.0 h. After the reaction completion monitored by TLC analysis, the solvent was removed under reduced pressure and the residue was purified by a silica gel flash chromatography (eluent: petroleum ether / EtOAc = 10 / 1) to afford compounds **S47** in 90% yield.

To a solution of **S47** (2.0 mmol, 1.0 equiv) in 20.0 mL of freshly distilled anhydrous THF was added NaH (2.4 mmol, 1.2 equiv) at room temperature for 1.0 h and the mixture was warmed up to 70 °C. The resulted mixture was added with BnBr (3.0 mmol, 1.5 equiv) and was stirred for 12.0 h. After the reaction completion monitored by TLC analysis, the solvent was removed under reduced pressure and the residue was purified by a silica gel flash chromatography (eluent: petroleum ether / EtOAc = 10 / 1) to afford compound **1aal** in 88% yield with 818 mg.

5. General Procedure for the Synthesis of Products

General procedure for the synthesis of compounds 2a-2ah, 2aj-2ao

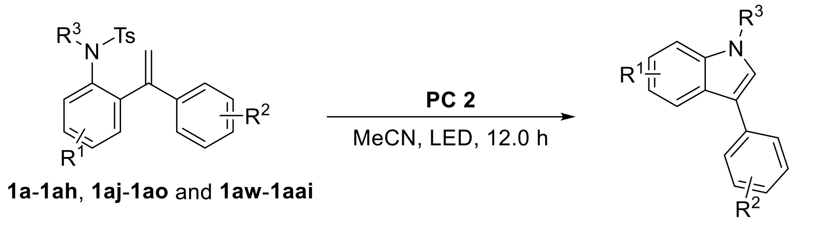

To a stirred solution of **1a-1ah**, **1aj-1ao** and **1aw-1aai** (0.1 mmol, 1.0 equiv) was added **PC 2** (2.0 mol%) in degassed MeCN (2.0 mL) under argon atmosphere and the mixture was irradiated with 385 nm LED or 100 W BLUE LED for 12.0-24.0 h. After the removal of solvent under reduced pressure, the residue was purified by a column chromatography on silica gel (petroleum ether / ethyl acetate = 20 / 1) to afford the corresponding compounds **2a-2ah** and **2aj-2ao** in the yields ranging from 37% to 98%.

General procedure for the synthesis of compounds 2ai and 2ap and the scale-up reaction of 2a

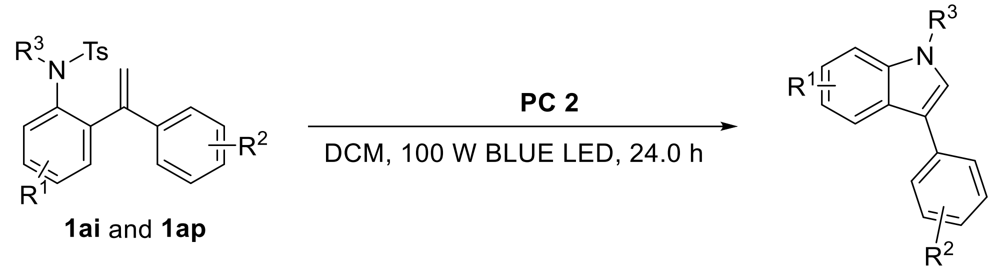

1ai and **1ap** **2ai** and **2ap**

To a stirred solution of **1ai** and **1ap** (0.1 mmol, 1.0 equiv) was added **PC 2** (2.0 mol%) in degassed DCM (2.0 mL) under argon atmosphere and the mixture was irradiated with 100 W BLUE LED for 24.0 h. After the removal of solvent under reduced pressure, the residue was purified by a column chromatography on silica gel (petroleum ether / ethyl acetate = 20 / 1) to afford the corresponding compounds **2ai** and **2ap** in 32% and 21% yields, respectively.

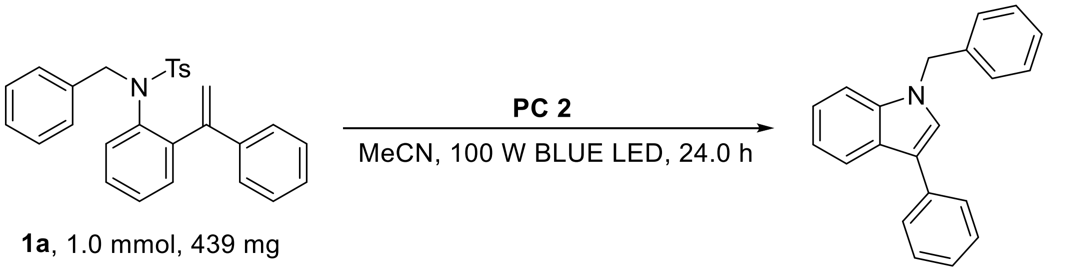

1a, 1.0 mmol, 439 mg

2a, 232 mg, 82% yield

To a stirred solution of **1a** (439 mg, 1.0 mmol) was added **PC 2** (2.0 mol%) in degassed MeCN (20.0 mL) under argon atmosphere and the mixture was irradiated with 100 W BLUE LED for 24.0 h. After the removal of solvent under reduced pressure, the residue was purified by a column chromatography on silica gel (petroleum ether / ethyl acetate = 20 / 1) to afford the corresponding compound **2a** in 82% yield with 232 mg.

General procedure for the synthesis of compound [*D*]-2a

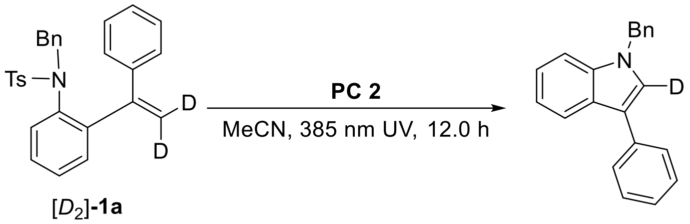

[D_2]-**1a**

[*D*]-**2a**

To a stirred solution of [D_2]-**1a** (0.1 mmol, 1.0 equiv) was added **PC 2** (2.0 mol%) in degassed MeCN (2.0 mL) under argon atmosphere and the mixture was irradiated with 385 nm LED light for 12.0 h. After the removal of solvent under reduced pressure, the residue was purified by a column chromatography on silica gel (petroleum ether / ethyl acetate = 20 / 1) to afford the corresponding compound [*D*]-**2a** in 83% D containing 93%.

General procedure for the synthesis of compound 11a

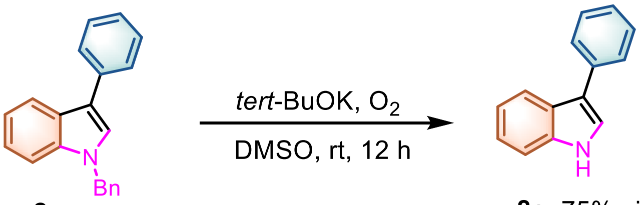

2a **8a**, 75% yield

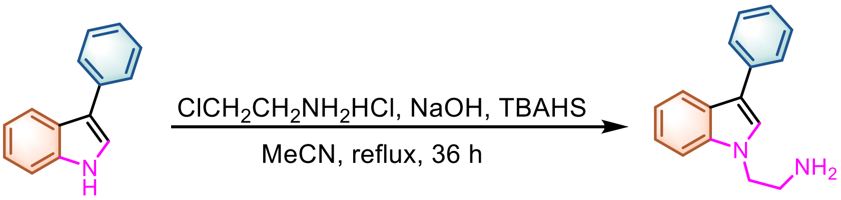

8a **9a**, 61% yield

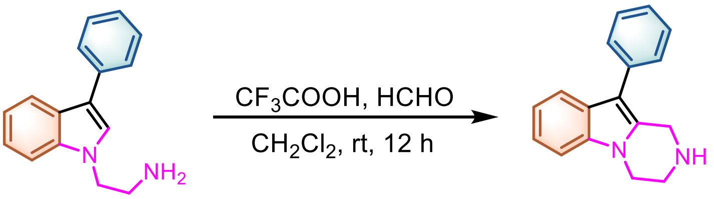

9a **11a**, 76% yield

Compound **2a** (2.0 mmol) was dissolved in DMSO (4 mL). While the solution was stirred at room temperature, KOt-Bu (10 mL of a 1 M solution in THF, 10.0 mmol) was added. Oxygen was then bubbled into the resulting solution for 12 h. Upon completion (determined by TLC), the reaction was quenched with saturated aqueous NH_4Cl (20 mL). The aqueous phase was extracted with EtOAc (3 × 10 mL), and the combined organic layers were dried over anhydrous Na_2SO_4 and concentrated under reduced pressure. The remaining residue was purified by a flash column chromatography on silica gel (petroleum ether / ethyl acetate = 10 / 1) to afford **8a** (290 mg, 75% yield) as a yellow solid.

To a solution of the compound **8a** (1.0 mmol) in MeCN (3 mL) was added sodium hydroxide (200 mg, 5.0 mmol) and tetrabutylammonium hydrogen sulfate (17 mg, 0.05 mmol). After the solution was stirred at room temperature for 30 min, 2-chloroethylamine hydrochloride (139 mg, 1.2 mmol) was added. Then the reaction mixture was refluxed for 36 h. The resulting mixture was poured into water (10 mL), extracted with diethyl ether, dried over anhydrous $MgSO_4$, and concentrated under reduced pressure to give a crude product. The crude was then purified by a flash column chromatography on silica gel (petroleum ether / acetone = 1 / 1) to give the product **9a** (144

mg, 61% yield) as a yellow oil.

A solution of the **9a** (0.2 mmol) in dichloromethane (2 mL) was treated with formaldehyde (37% aqueous solution, 328 μL, 0.4 mmol). Trifluoroacetic acid (60 μL, 0.8 mmol) was then added at room temperature, and the resulting reaction mixture was stirred for 12 h. Saturated aqueous sodium hydrogen carbonate was added, and the organic phase was separated. The aqueous phase was extracted with dichloromethane (DCM), the organic extracts were combined together, washed with saturated aqueous sodium chloride, and dried over anhydrous Na_2SO_4. Removal of the solvent under reduced pressure left a residue which was purified by a column chromatography on silica gel (petroleum ether / ethyl acetate = 2 / 1) to afford **11a** (38 mg, 76% yield) as a light yellow solid.

6. Mechanistic Investigations

6.1 Radical Trapping Experiment

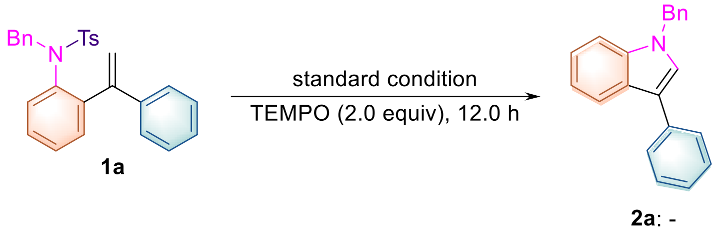

To a stirred solution of **1a** (0.1 mmol, 1.0 equiv) was added **PC 2** (2.0 mol%) and TEMPO (0.2 mmol, 2.0 equiv) in degassed MeCN (2.0 mL) under argon atmosphere and the mixture was irradiated with 385 nm LED for 12.0 h. When the reaction finished, the mixture was concentrated in *vacuo*. The yield of product **2a** was determined by ^{1}H NMR spectroscopy using 1,3,5-trimethoxybenzene as an internal standard.

6.2 Quantum Yield

To further investigate whether the chain process is involved upon light irradiation, we measured the quantum yields of the reaction of **1a**, **1n** and **1o** to **2a**, **2n** and **2o**.

$$\phi = \frac{n_x}{n_p} = \frac{n_x}{\frac{\Delta E \times S \times t}{N_A h v}} = \frac{n_x \times N_A \times h \times c}{\Delta E \times S \times t \times \lambda}$$

n_x is the amount of photochemical or photophysical events x occurred during irradiation, n_p is the number of photons absorbed by the reactant. E is the radiant power. S is the irradiated area. t is the irradiated time. N_A is the Avogadro constant. h is the Planck constant. v is the frequency of incident light. n_x was analyzed by ^{1}H NMR, *DE* was measured by ILT1400 Portable Radiometer/Photometer.[10]

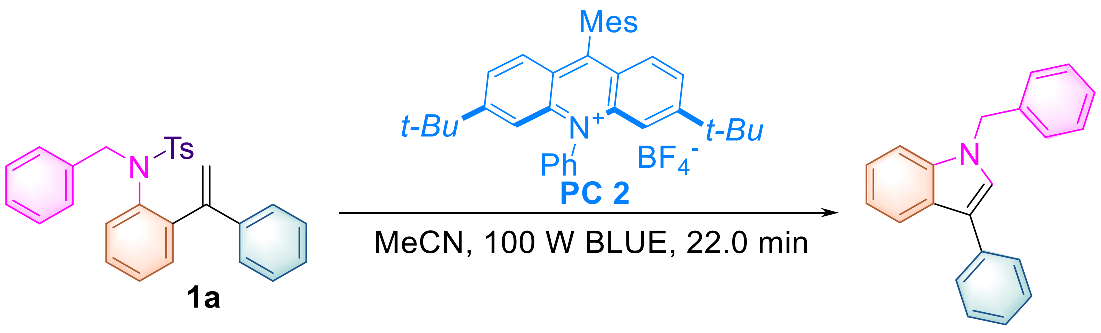

A cuvette equipped with a magnetic stir bar was added substrate **1a** (0.1 mmol, 1.0 equiv) and degassed acetonitrile (2.0 mL). Record the value of blank sample after which **PC 2** (2.0 mol%) was added at room temperature. The heterogeneous mixture was placed at a distance (app. 10.0 cm) from 100 W Blue LED irradiation for 22.0 min, The reaction mixture was concentrated in *vacuo* and analyzed by ^{1}H NMR spectrum using 1,3,5-trimethoxybenzene as an internal standard. The quantum yield is calculated to be 1.77.

$$\Phi = \frac{0.019 \times 10^{-3}\,mol \times 6.022 \times 10^{23} \times 6.626 \times 10^{-34}\,J{\cdot}s \times 2.998 \times 10^{8}\,m{\cdot}s^{-1}}{(11.7 \times 10^{-4}\,W{\cdot}cm^{-2} \times 2\,cm^{2}) \times 1320\,s \times 415 \times 10^{-9}\,m} = 1.77$$

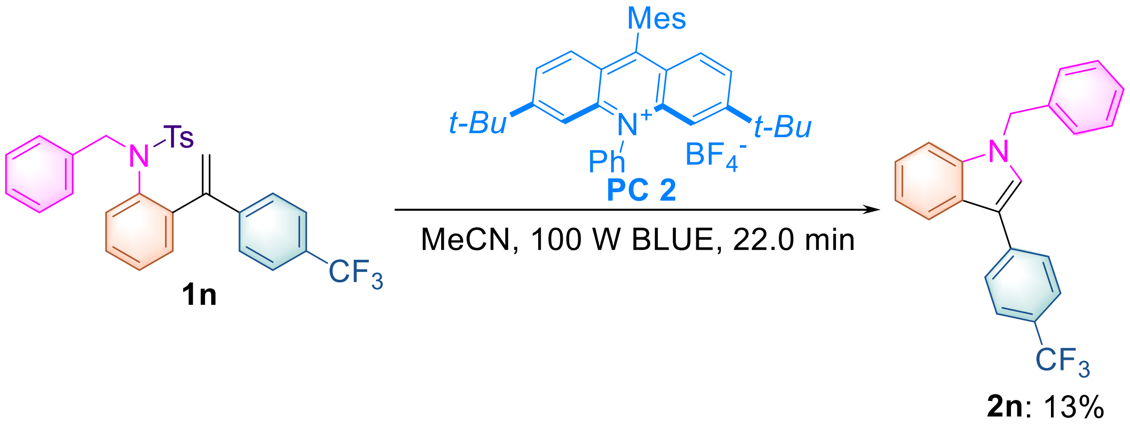

A cuvette equipped with a magnetic stir bar was added substrate **1n** (0.1 mmol, 1.0 equiv) and degassed acetonitrile (2.0 mL). Record the value of blank sample after which **PC 2** (2.0 mol%) was added at room temperature. The heterogeneous mixture was placed at a distance (app. 10.0 cm) from 100 W Blue LED irradiation for 22.0 min, The reaction mixture was concentrated in *vacuo* and analyzed by ^{1}H NMR spectrum using 1,3,5-trimethoxybenzene as an internal standard. The quantum yield is calculated to be 0.17.

$$\Phi = \frac{0.013 \times 10^{-3} mol \times 6.022 \times 10^{23} \times 6.626 \times 10^{-34} J \cdot s \times 2.998 \times 10^{8} m \cdot s^{-1}}{(8.2 \times 10^{-3} W \cdot cm^{-2} \times 2cm^{2}) \times 1320s \times 415 \times 10^{-9} m} = 0.17$$

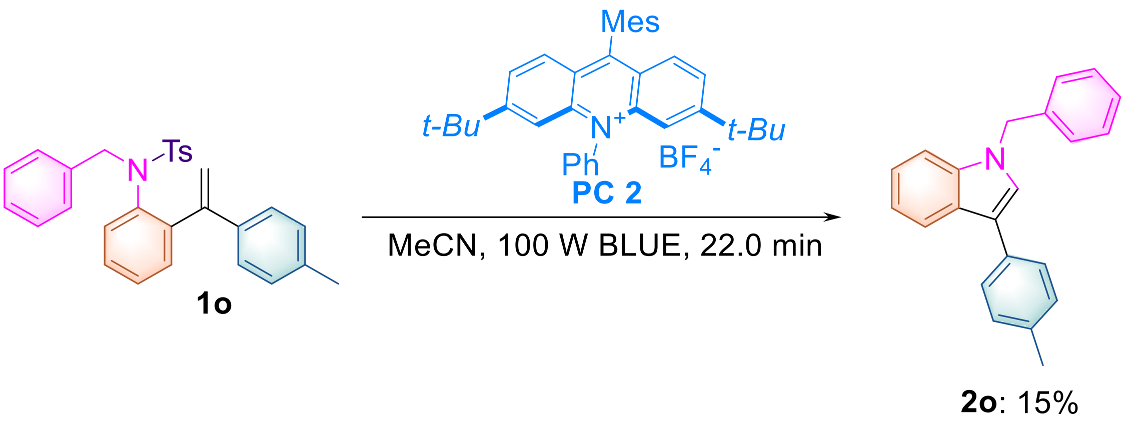

A cuvette equipped with a magnetic stir bar was added substrate **1o** (0.1 mmol, 1.0 equiv) and degassed acetonitrile (2.0 mL). Record the value of blank sample after which **PC 2** (2.0 mol%) was added at room temperature. The heterogeneous mixture was placed at a distance (app. 10.0 cm) from 100 W Blue LED irradiation for 22.0 min, The reaction mixture was concentrated in *vacuo* and analyzed by ^{1}H NMR spectrum using 1,3,5-trimethoxybenzene as an internal standard. The quantum yield is calculated to be 0.18.

$$\Phi = \frac{0.015 \times 10^{-3} mol \times 6.022 \times 10^{23} \times 6.626 \times 10^{-34} J{\cdot}s \times 2.998 \times 10^{8} m{\cdot}s^{-1}}{(8.9 \times 10^{-3} W{\cdot}cm^{-2} \times 2cm^{2}) \times 1320s \times 415 \times 10^{-9} m} = 0.18$$

6.3 Emission Quenching Studies

All the emission intensities were recorded by Varian Cary Eclipse spectrometer. Solutions of **PC 2** (5.0×10^{-3} M) was added with **1a** (1.0 M) or **2a** (1.0 M) or **2m** (1.0 M) in dry MeCN upon excitation at 415 nm and the emission intensity was collected at 503 - 508 nm. Solution was introduced to a 1.0 cm path length quartz cuvette equipped with a Teflon® septum.

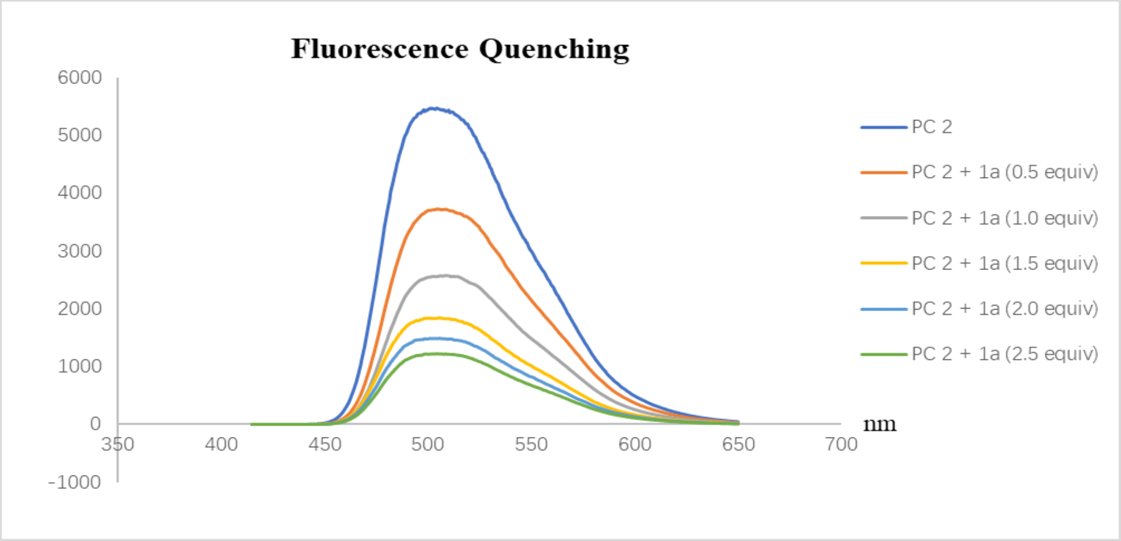

Figure S1. Fluorescence quenching experiment of **1a**

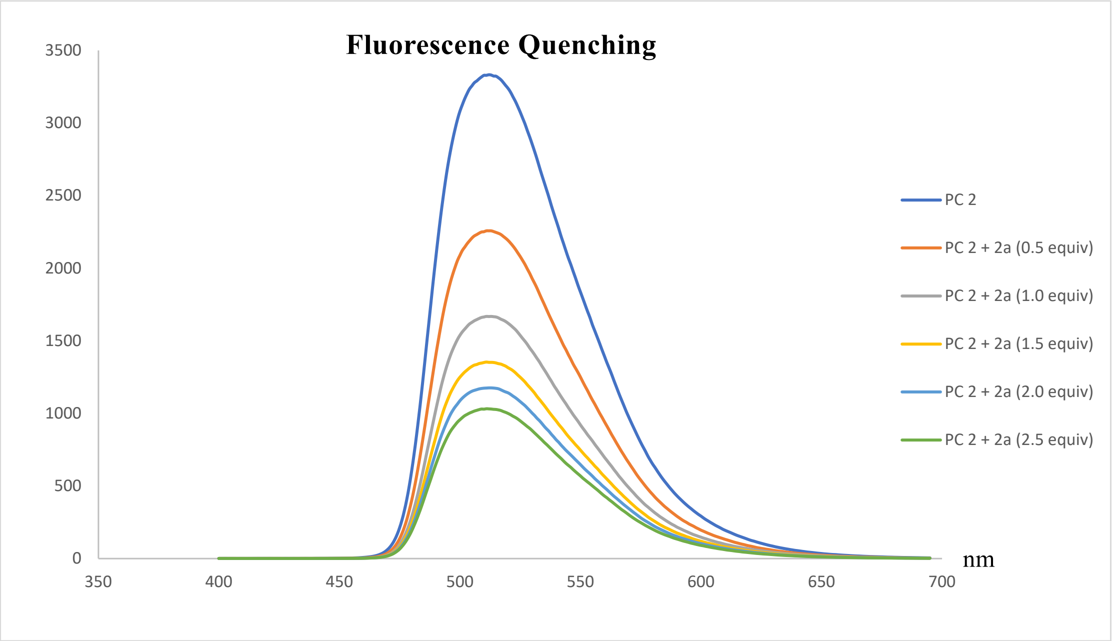

Figure S2. Fluorescence quenching experiment of **2a**

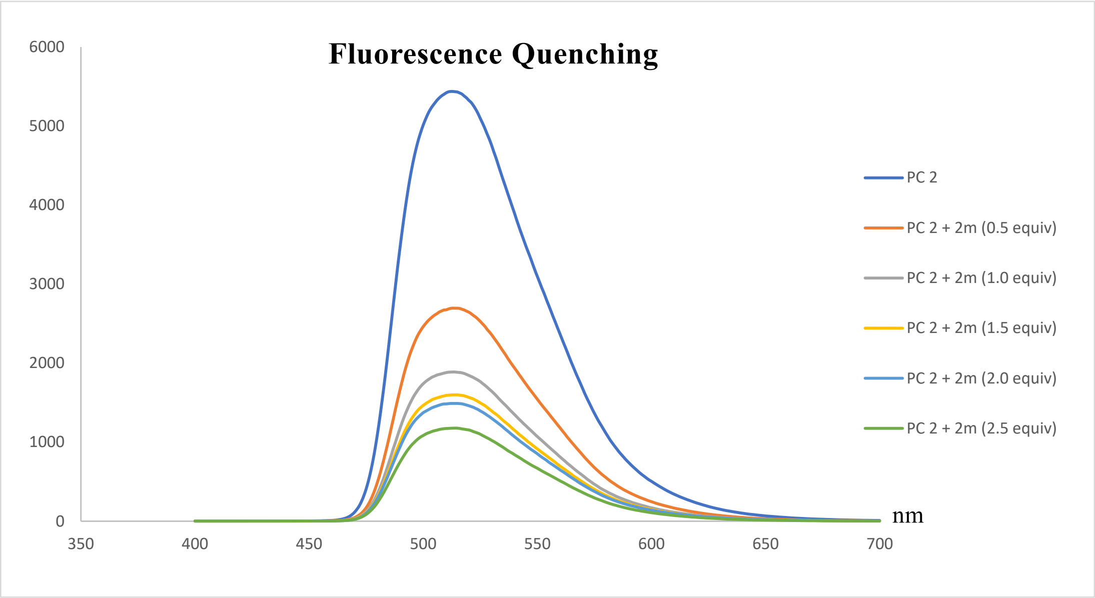

Figure S3. Fluorescence quenching experiment of **2m**

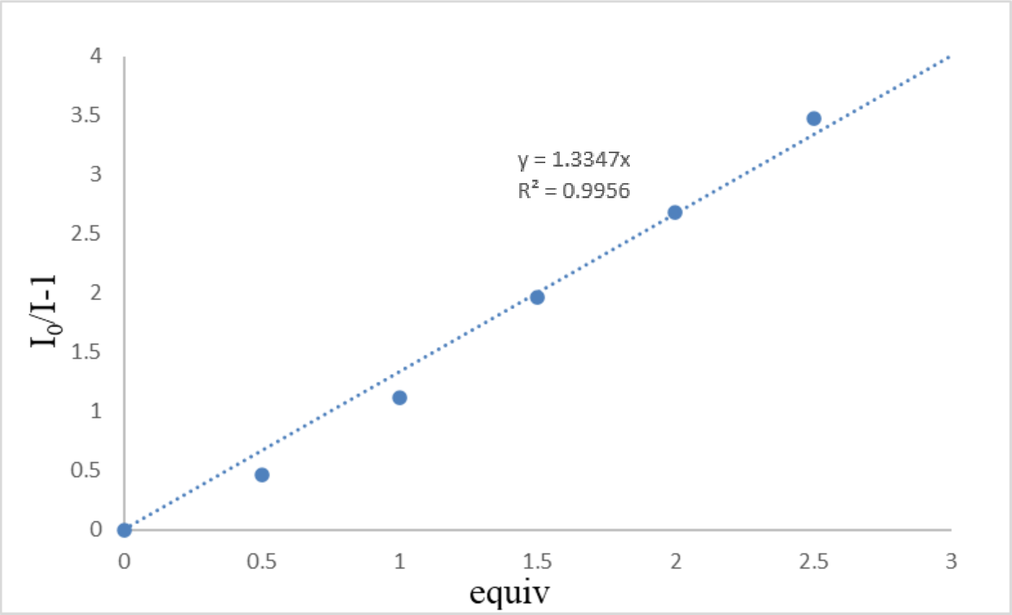

Figure S4. Stern-Volmer experiments of **1a**

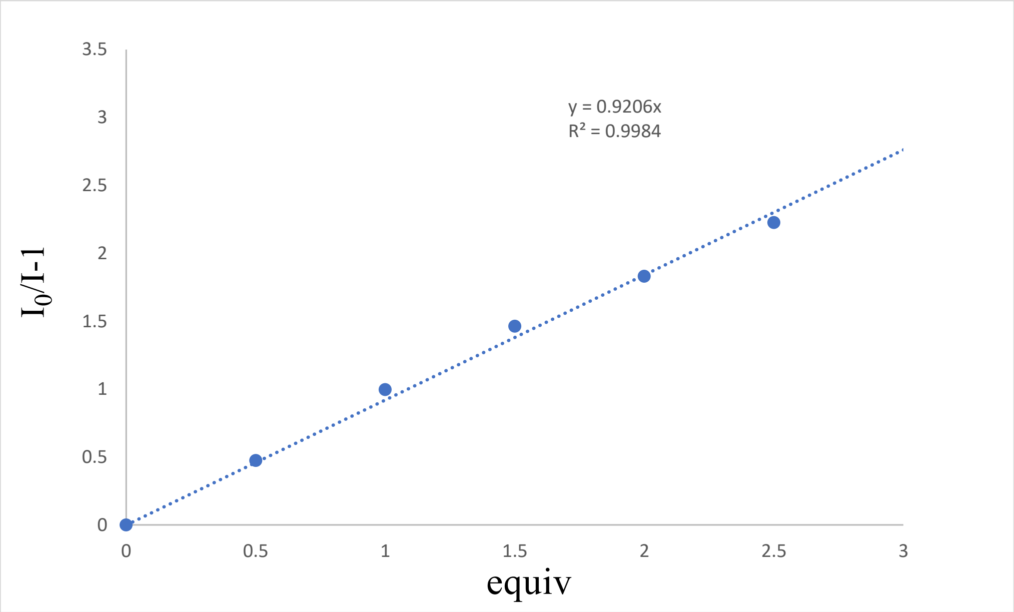

Figure S5. Stern-Volmer experiments of **2a**

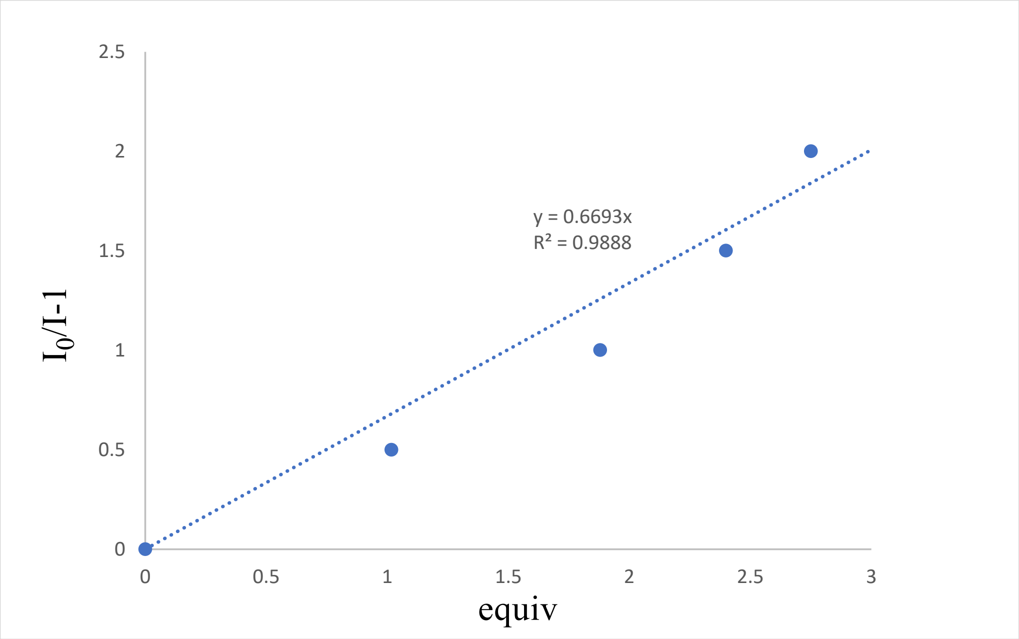

Figure S6. Stern-Volmer experiments of **2m**

To get a more accurate result, Stern-Volmer experiments were repeated at a lower concentration. Solutions of **PC 2** (5.0×10^{-5} M) was added with **1a** (1.0 M) or **2a** (1.0 M) in dry MeCN upon excitation at 415 nm and the emission intensity was collected at 503 - 508 nm. Solution was introduced to a 1.0 cm path length quartz cuvette equipped with a Teflon® septum. Similar results as mentioned above were obtained.

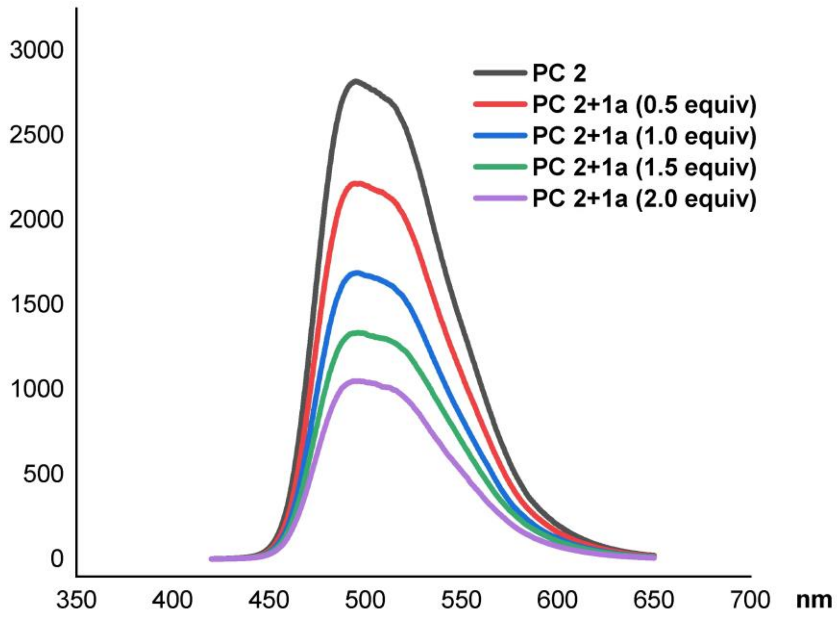

Figure S7. Fluorescence quenching experiment of **1a**

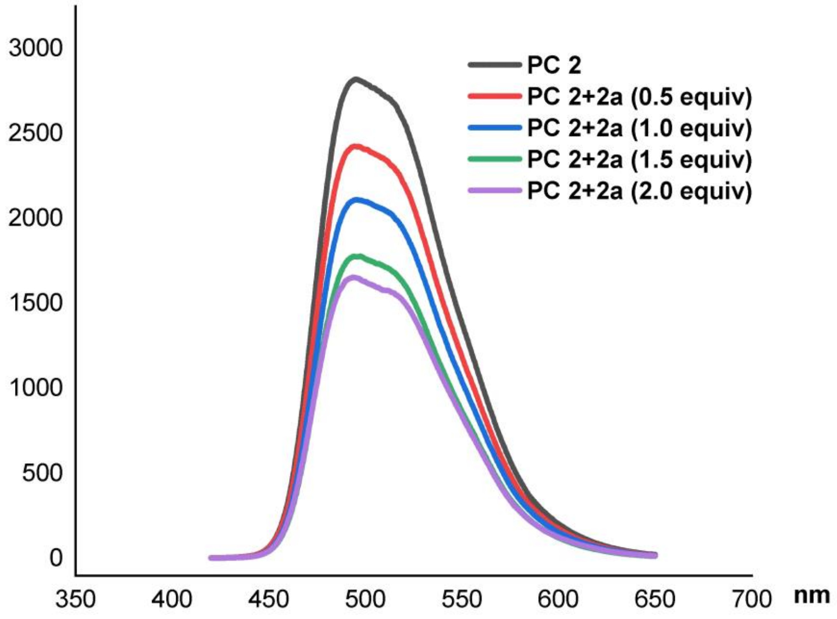

Figure S8. Fluorescence quenching experiment of **2a**

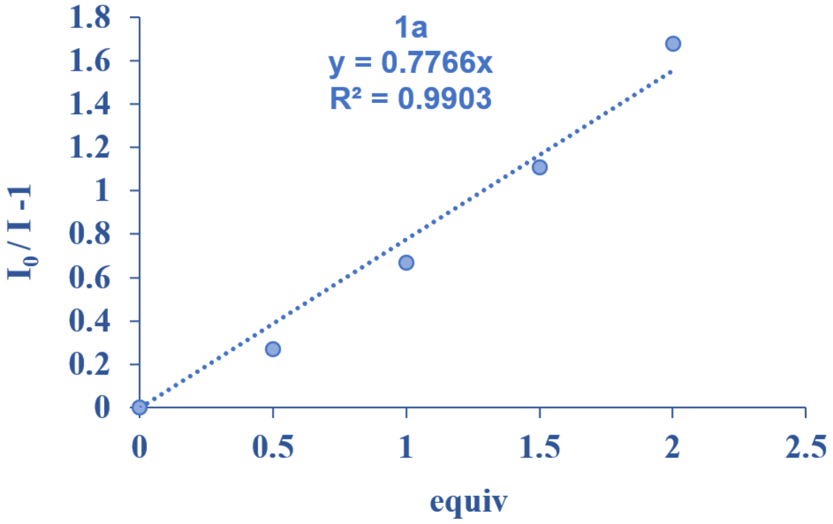

Figure S9. Stern-Volmer experiments of **1a**

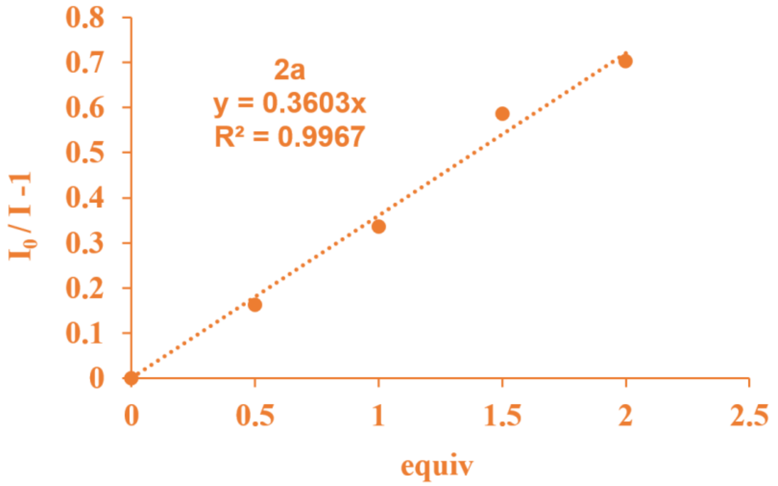

Figure S10. Stern-Volmer experiments of **2a**

6.4 Cyclic Voltammetry Experiments

Cyclic Voltammetry was performed on a CH Instruments Electrochemical Workstation model CS350H. A solution of the substrates **1a** in MeCN (0.2 M) was tested with 0.2 M Bu_4NPF_6 as the supporting electrolyte, using a glassy carbon as the working electrode, a Pt as the counter electrode, and a saturated calomel electrode reference electrode. Ar was bubbled into the system for 20.0 min to degas the solution. Scan rate = 0.1 V/s, 2 sweep segments, a sample interval of 0.001 V.

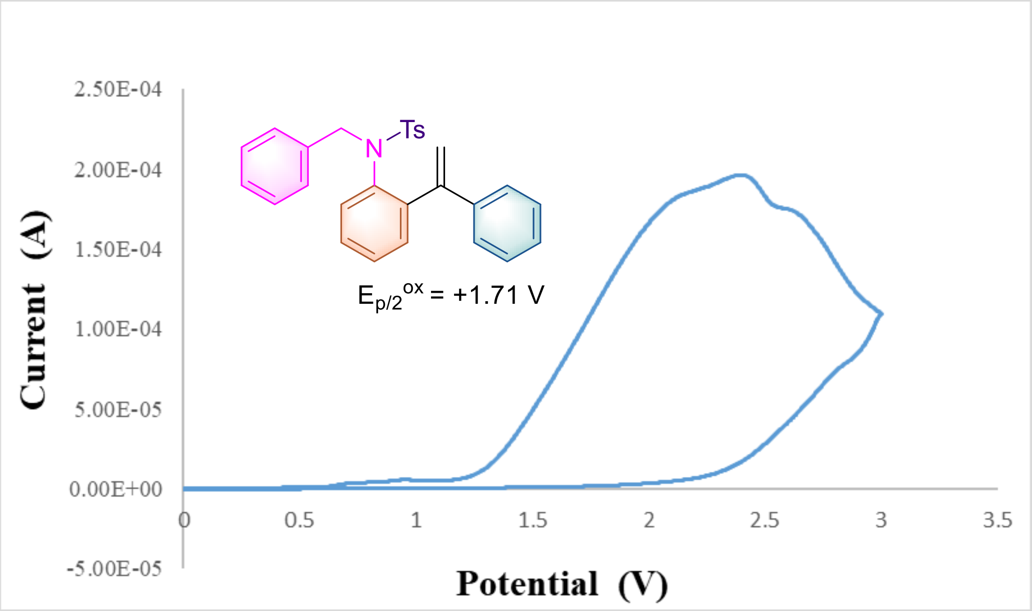

Figure S11. Oxidative potential of **1a**

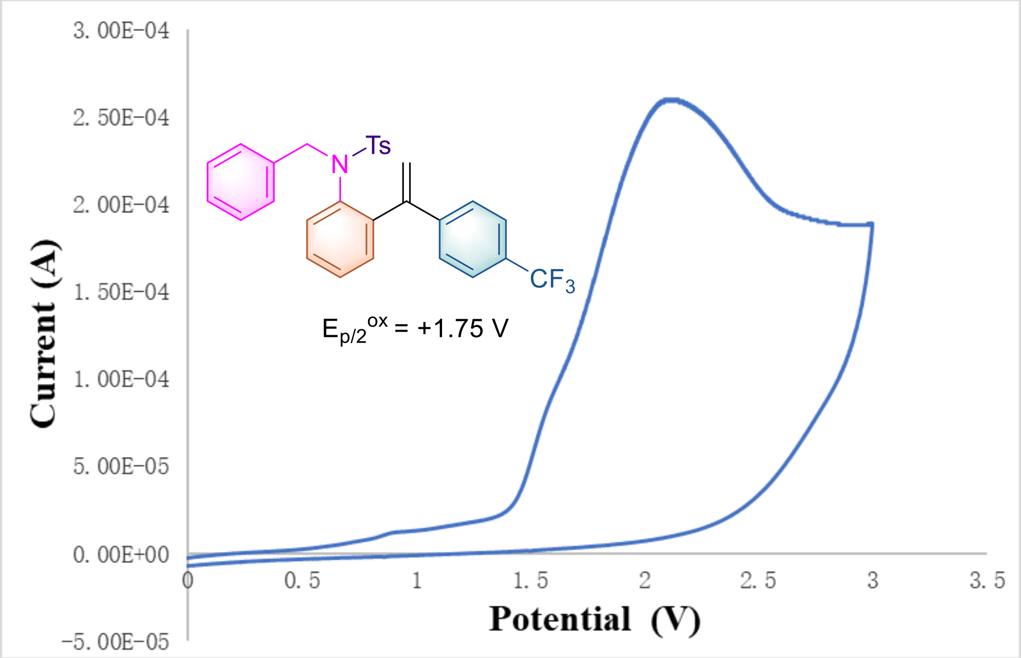

Figure S12. Oxidative potential of **1n**

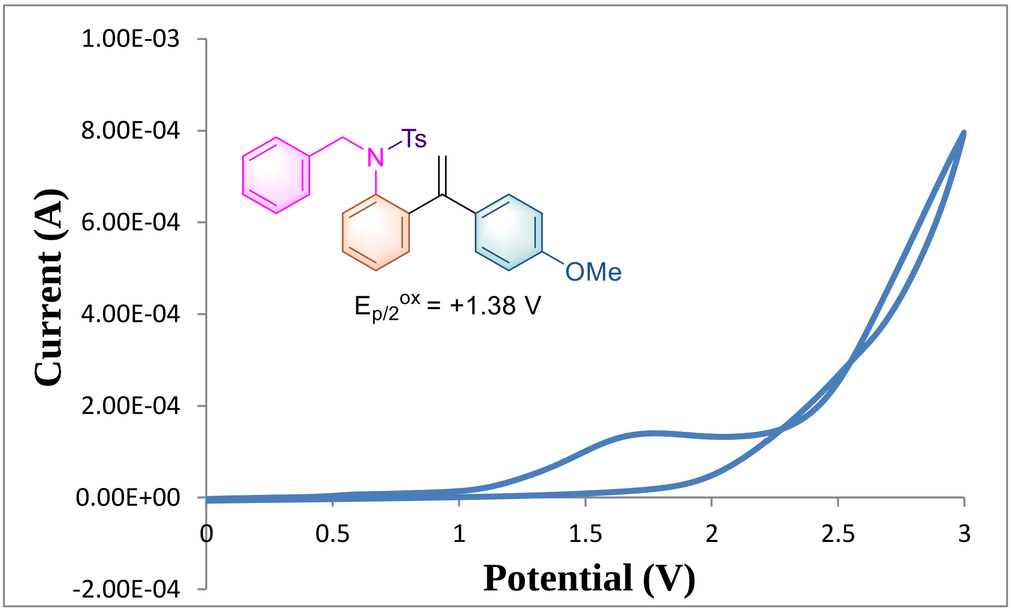

Figure S13. Oxidative potential of **1q**

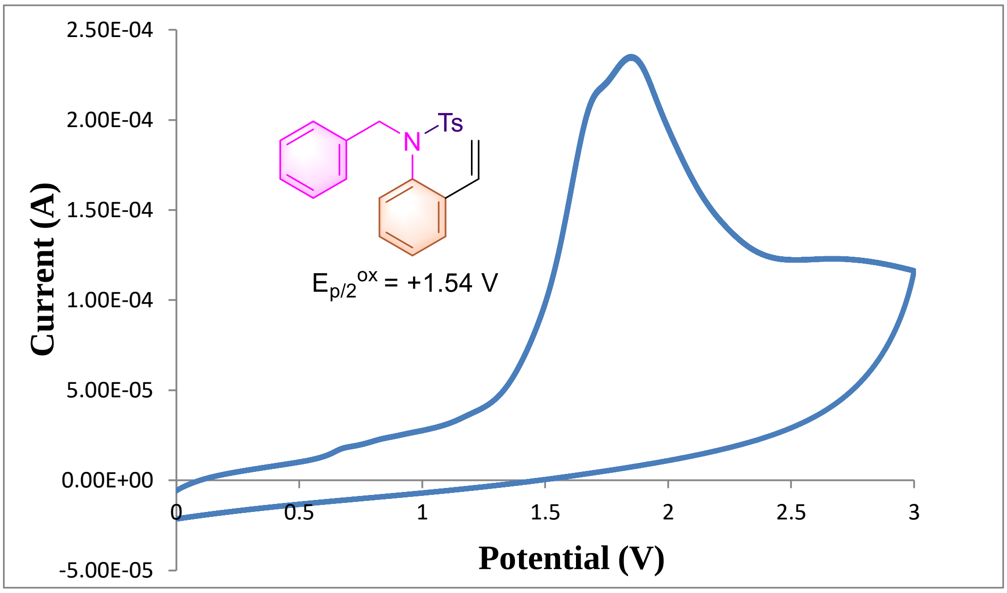

Figure S14. Oxidative potential of **1at**

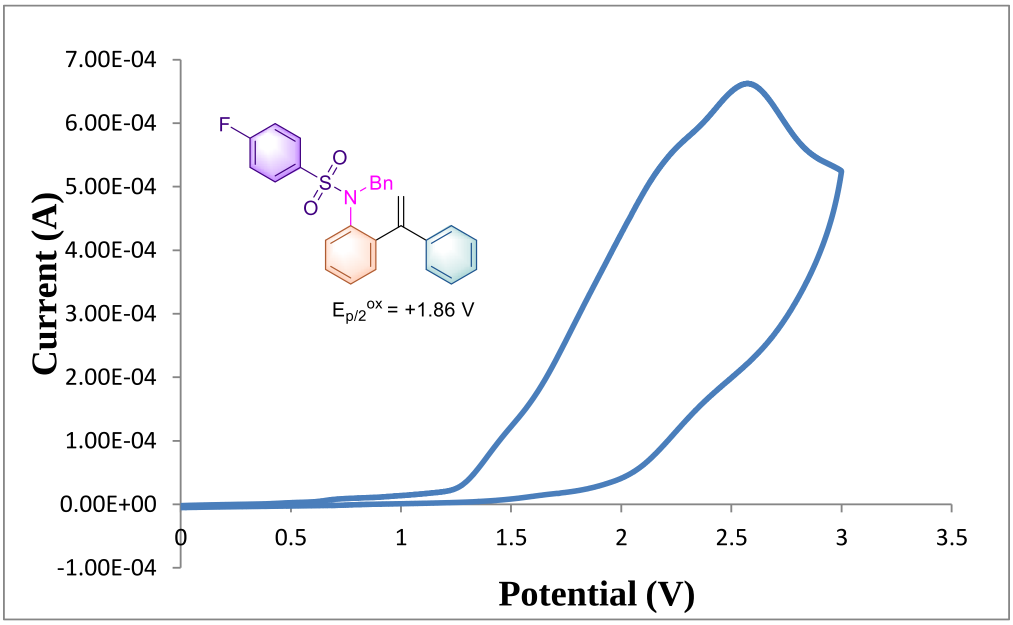

Figure S15. Oxidative potential of **1az**

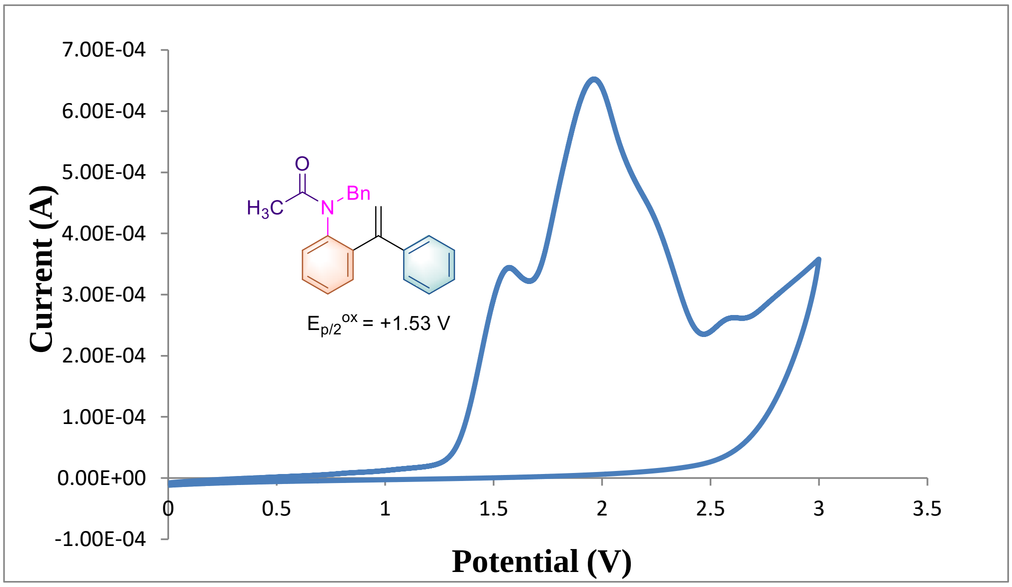

Figure S16. Oxidative potential of **1aaj**

6.5 Kinetic Experiment and Hammett plot

To a stirred solution of **1** (0.1 mmol, 1.0 equiv) was added **PC 2** (2.0 mol%) in degassed MeCN (2.0 mL) under argon atmosphere and the mixture was irradiated with 385 nm LED for 15.0 min, 20.0 min and 25.0 min. After the removal of solvent under reduced pressure, the yields of products **2** were measured by NMR spectroscopy using 1,3,5-trimethoxybenzene as an internal standard.

**Time (min)**	**0**	**15**	**20**	**25**
**Yield (%)**	0	9	13	17

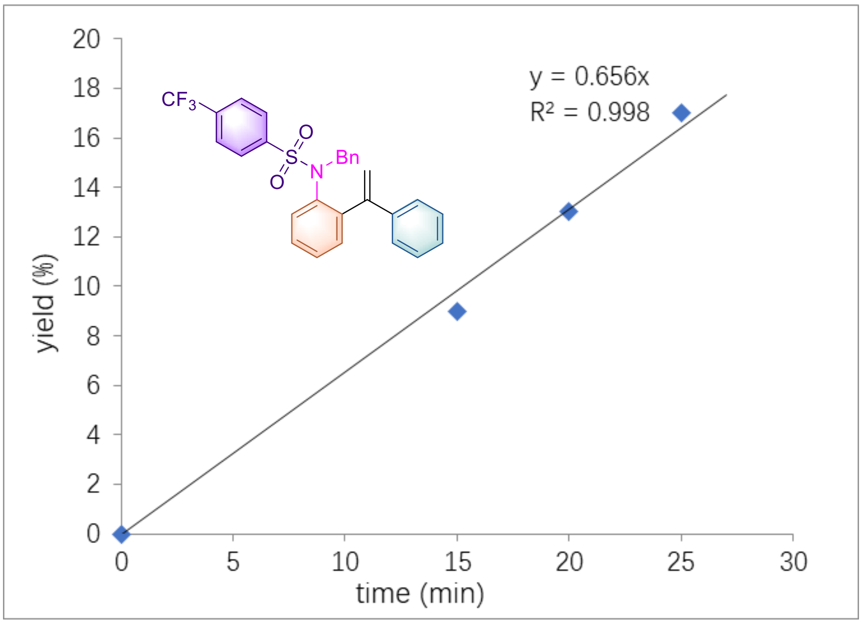

Figure S17. Rate constant of **1aac**

Time (min)	0	15	20	25
Yield (%)	0	14	18	21

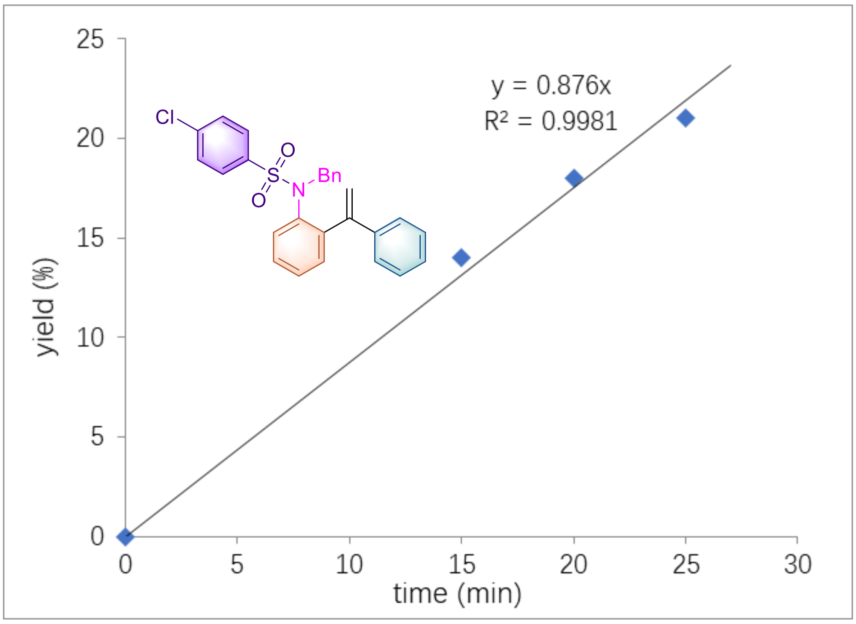

Figure S18. Rate constant of **1aaa**

Time (min)	0	15	20	25
Yield (%)	0	16	21	24

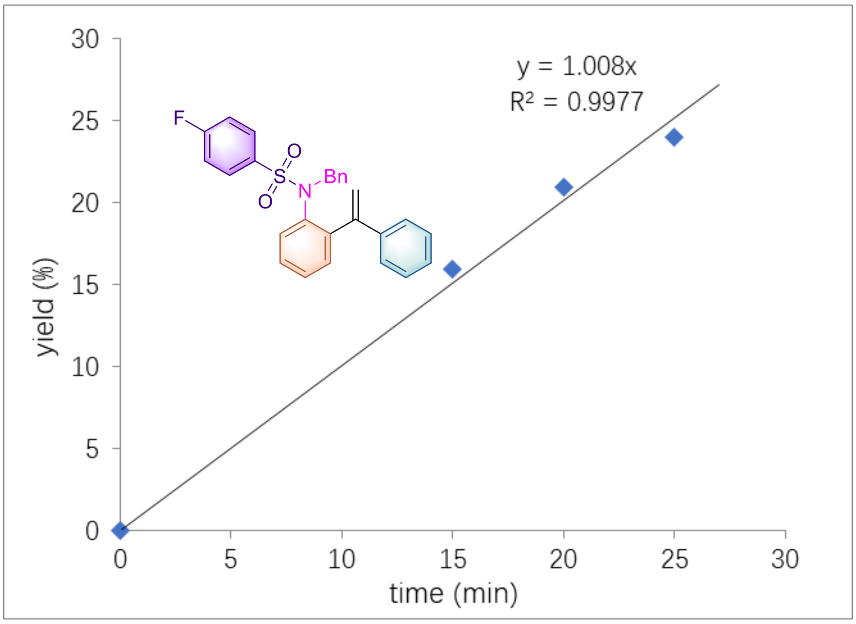

Figure S19. Rate constant of **1az**

Time (min)	0	15	20	25
Yield (%)	0	18	23	26

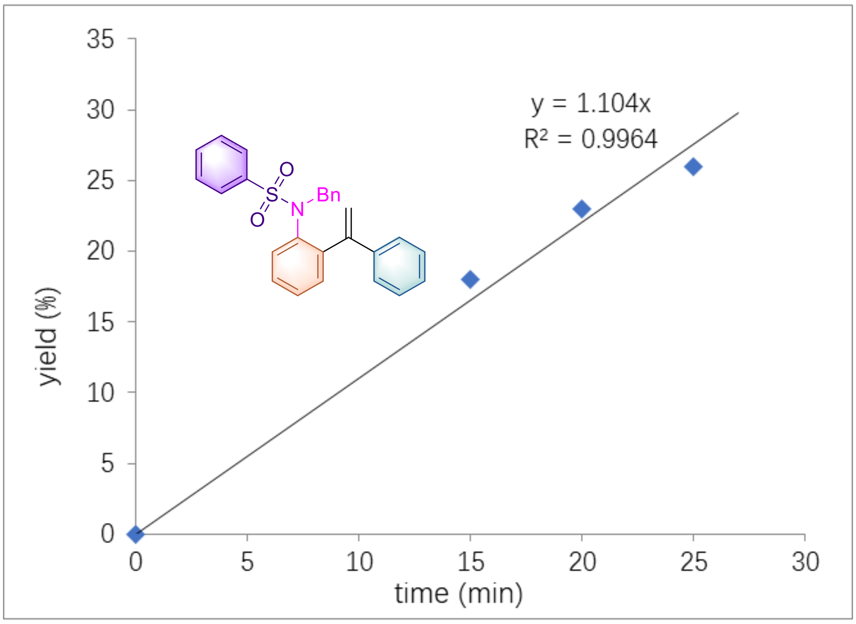

Figure S20. Rate constant of **1aw**

Time (min)	0	15	20	25
**Yield (%)**	0	21	27	31

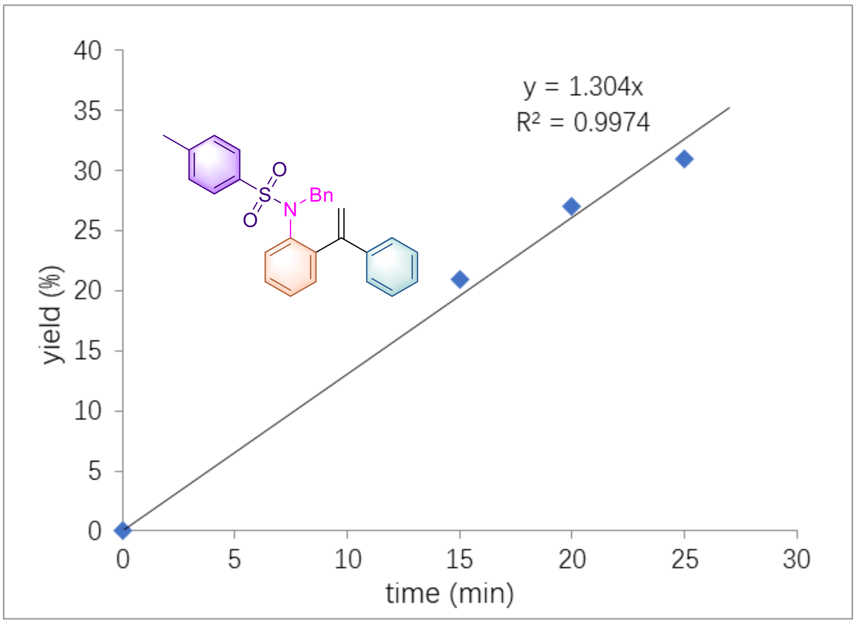

Figure S21. Rate constant of **1a**

Time (min)	0	15	20	25
Yield (%)	0	22	29	33

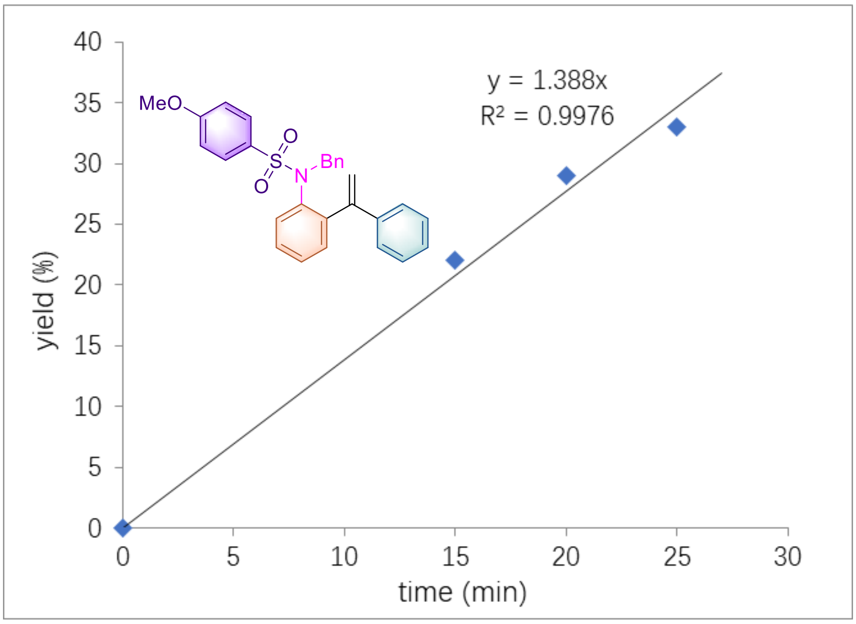

Figure S22. Rate constant of **1aab**

Time (min)	0	15	20	25
Yield (%)	0	5	6	8

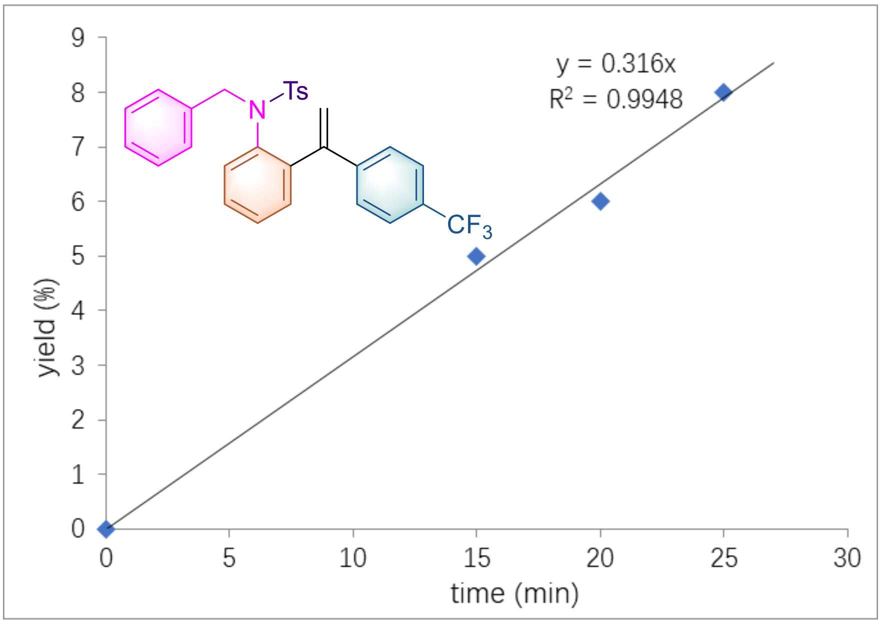

Figure S23. Rate constant of **1n**

Time (min)	0	15	20	25
Yield (%)	0	18	22	27

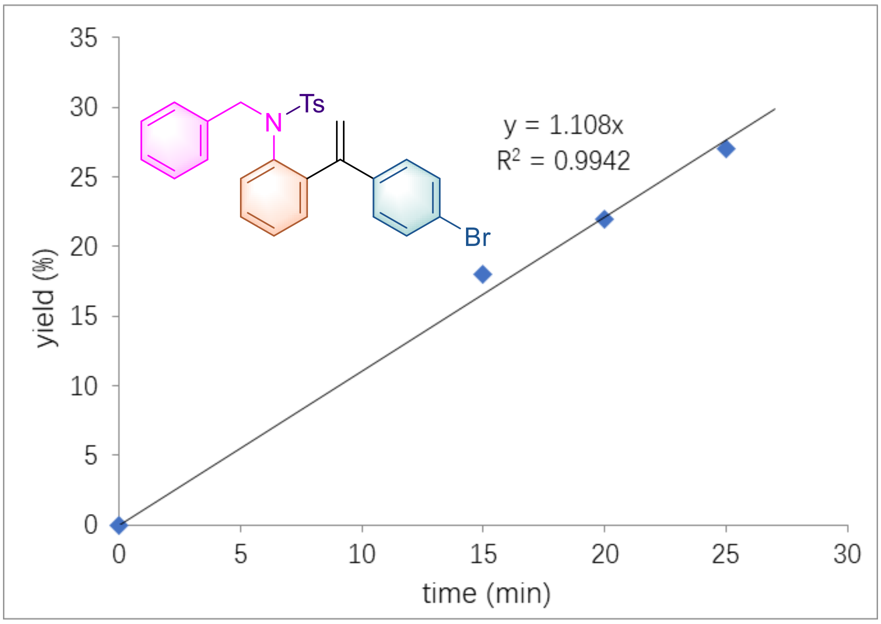

Figure S24. Rate constant of **1l**

Time (min)	0	15	20	25
Yield (%)	0	20	24	28

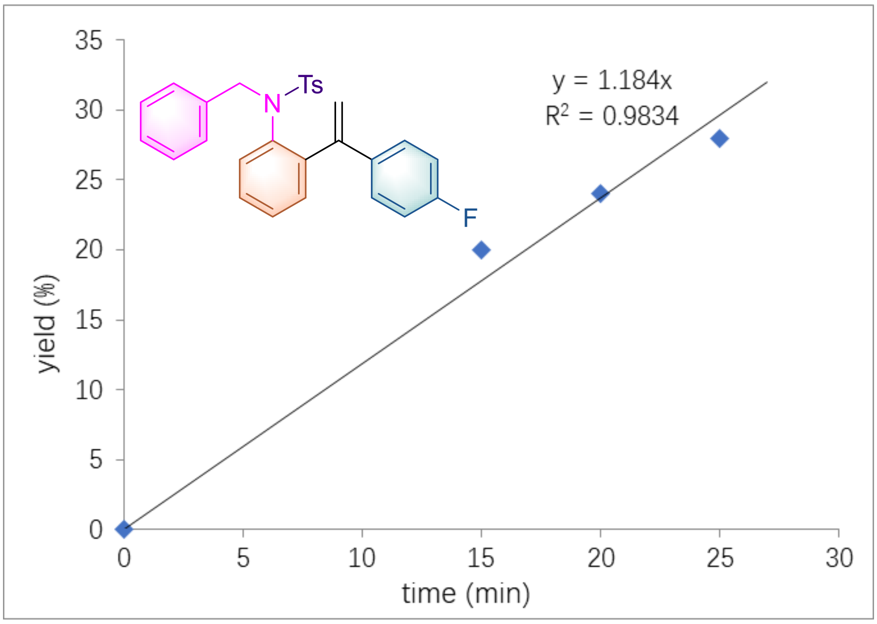

Figure S25. Rate constant of **1m**

Time (min)	0	15	20	25
Yield (%)	0	5	7	8

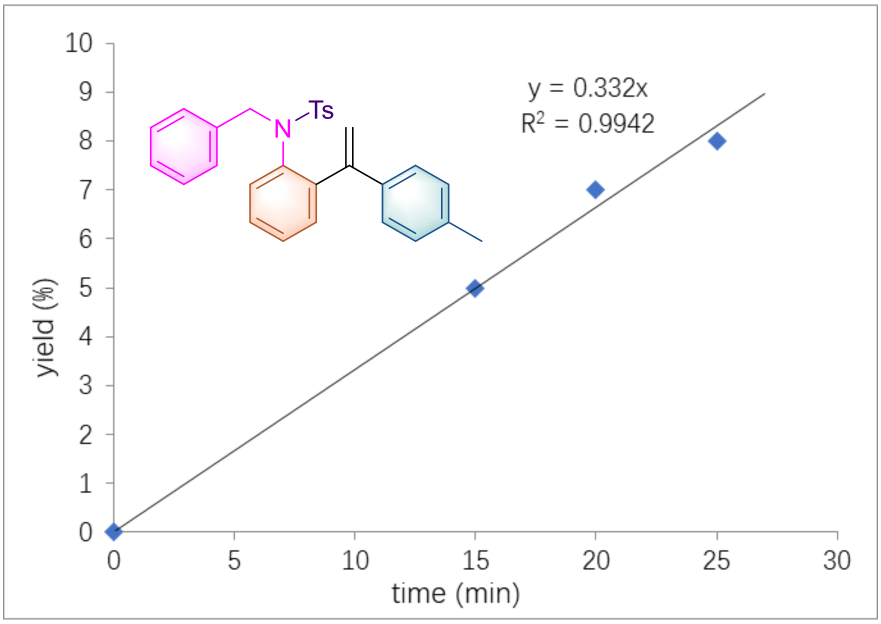

Figure S26. Rate constant of **1o**

substituent	k_X	k_X / k_H	log (k_X / k_H)	σ_p
OMe	1.388	1.2572	0.0994	-0.27
CH_3	1.304	1.1812	0.0723	-0.17
H	1.104	-	-	-
F	1.008	0.9130	-0.0395	0.06
Cl	0.876	0.7935	-0.1005	0.23
CF_3	0.656	0.5942	-0.2261	0.54

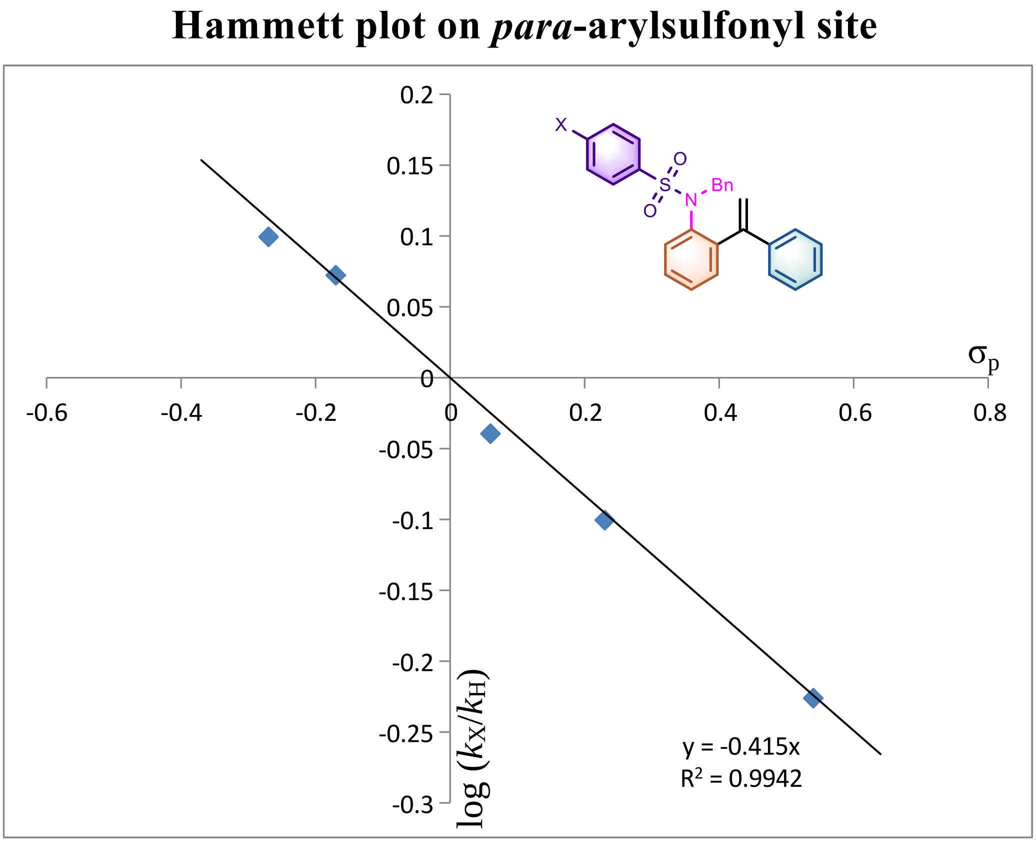

Figure S27. Hammett plot study on the *para*-arylsulfonyl site

6.6 The MS spectra of TsH and TsD

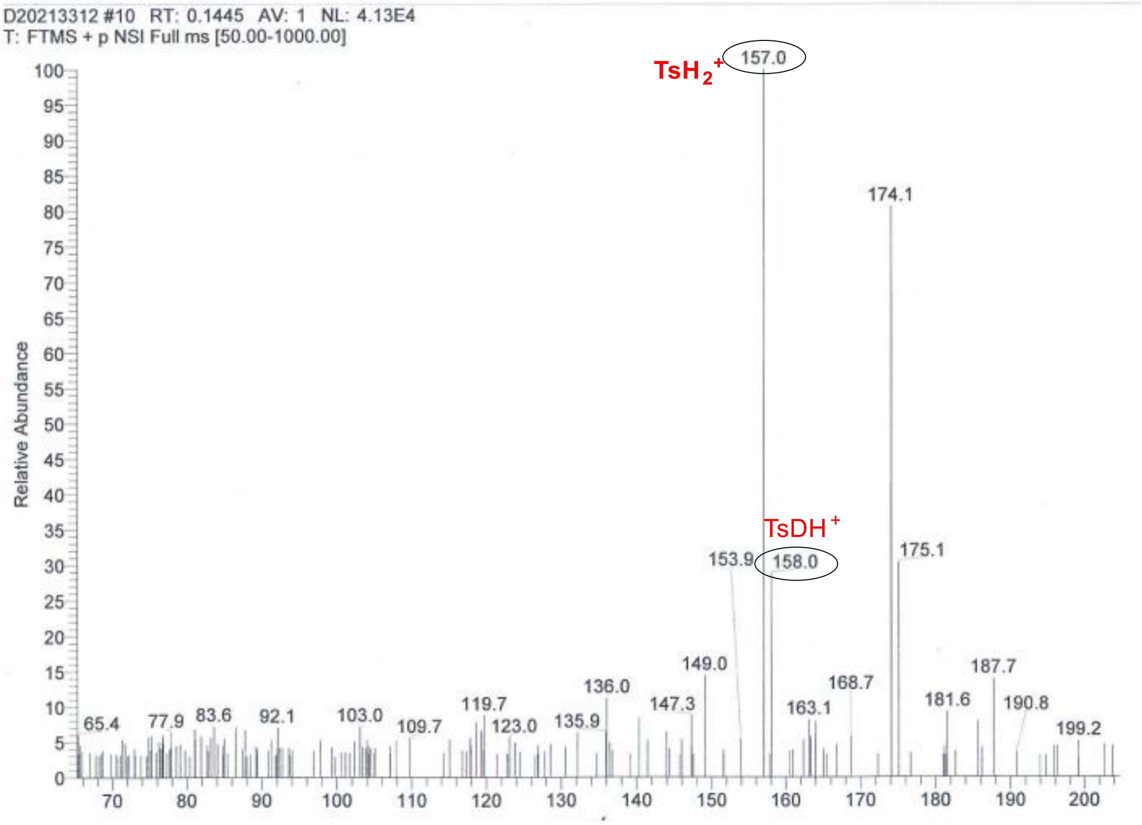

Figure S28.The MS spectra of TsH and TsD

6.7 NOE spectrum of remained 1ao after the reaction

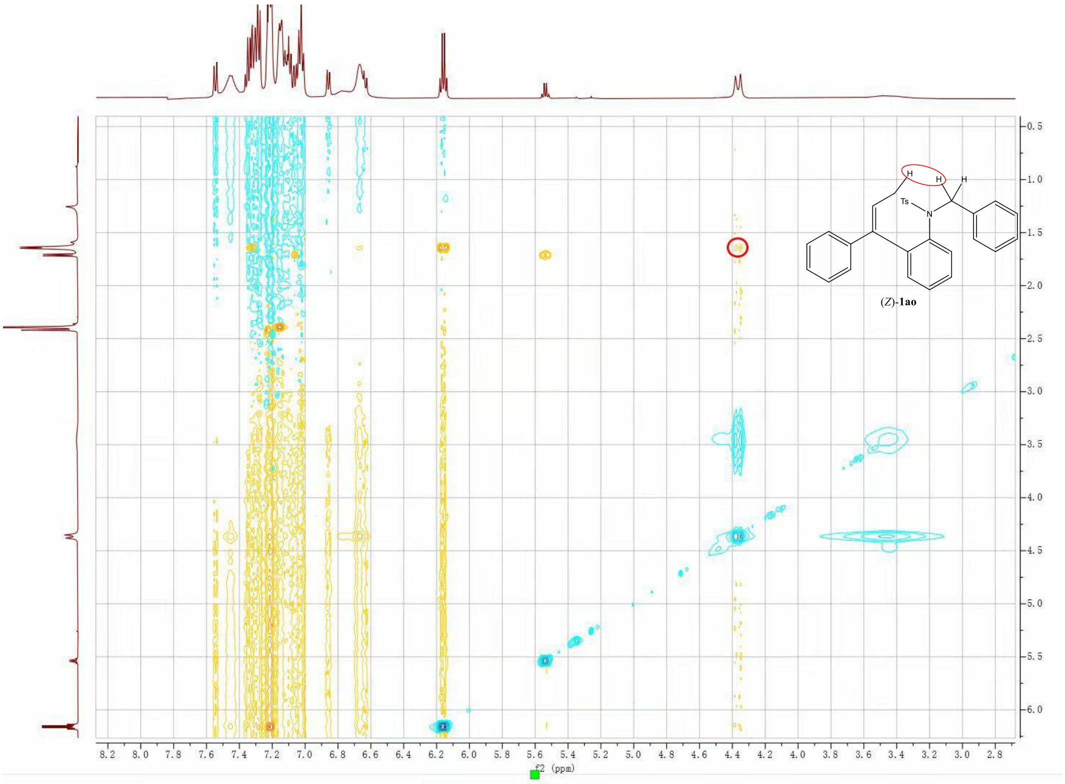

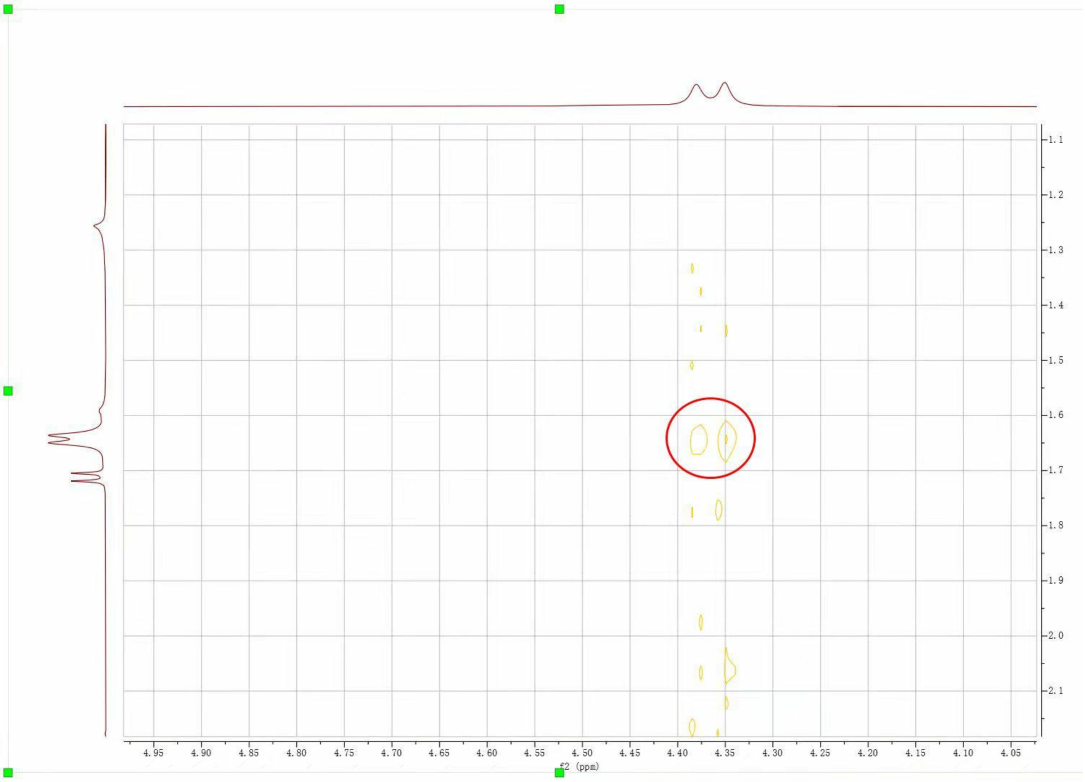

Figure S29. The NOE spectrum of **1ao** after the reaction

6.8 Radical clock experiment

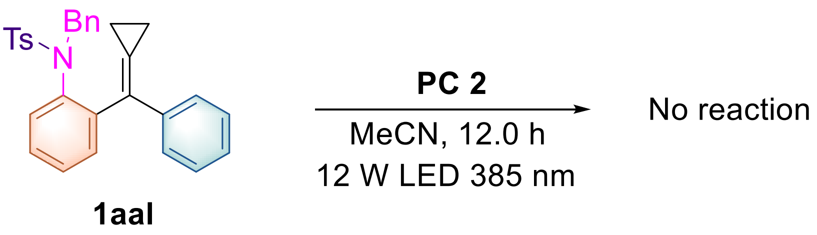

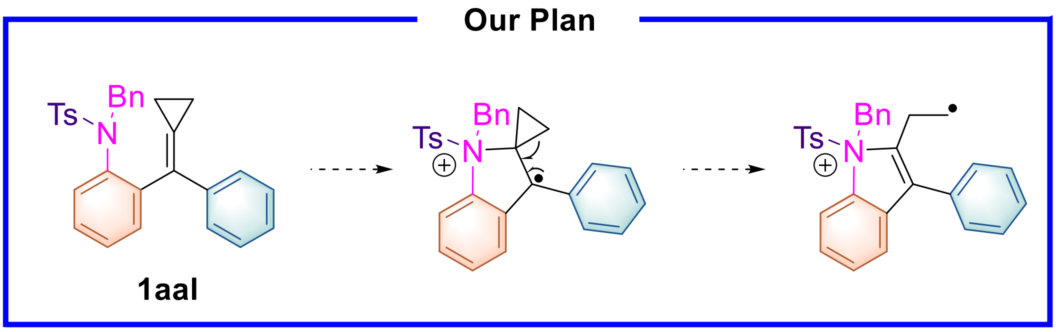

To a stirred solution of **1aal** (0.1 mmol, 1.0 equiv) was added **PC 2** (2.0 mol%) in degassed MeCN (2.0 mL) under argon atmosphere and the reaction mixture was irradiated with 385 nm LED 12. h. After the removal of solvent under reduced pressure, the residue was purified by a column chromatography on silica gel (petroleum ether / ethyl acetate = 10 / 1). This result showed that the substrate **1aal** did not undergo the expected reaction under the standard conditions, presumably due to the steric effect.

7. KIE Experiment

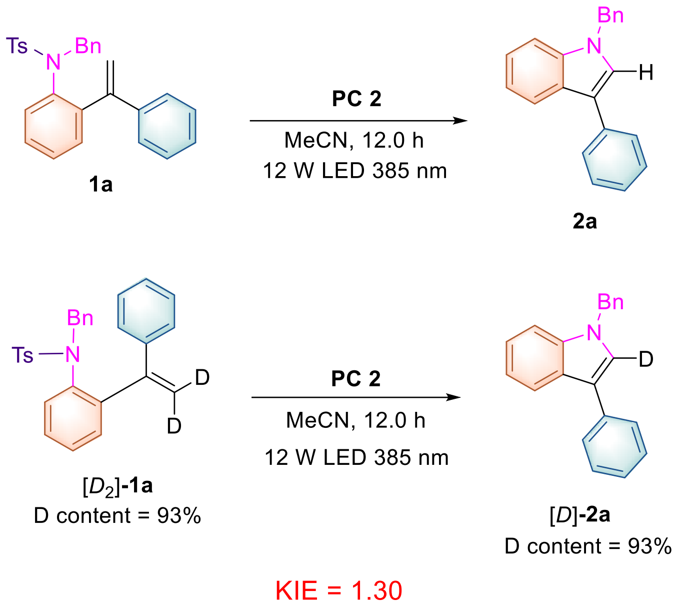

To a stirred solution of **1a** (0.1 mmol, 1.0 equiv) was added **PC 2** (2.0 mol%) in degassed MeCN (2.0 mL) under argon atmosphere in one sealed tube and [D_2]-**1a** (1.0 mmol, 1.0 equiv) was added **PC 2** (2.0 mol%) in degassed MeCN (2.0 mL) under argon atmosphere in another sealed tube and then the mixture was irradiated with 385 nm LED for 10.0 min, 15.0 min, 30.0 min and 40.0 min, respectively. After the removal of solvent under reduced pressure, the yields of products **2a** and [*D*]-**2a** were measured by NMR spectroscopy using 1,3,5-trimethoxybenzene as an internal standard. The corresponding kinetic isotope effect outcomes were shown in Fig. S24.

Time (min)	10	15	30	40
Yield (%)	18	23	46	62
	14	21	36	46

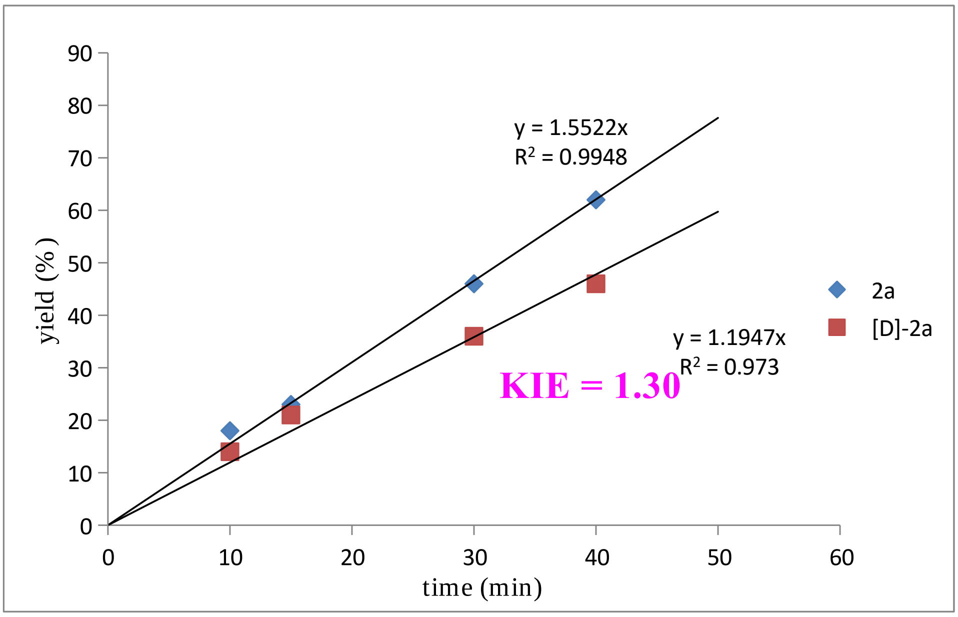

Figure S30. The experiments on the kinetic isotope effect from **1a** to **2a**

8. Characterization Data of Substrates 1

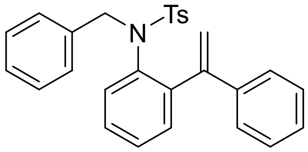

Compound 1a: Yield: 1186 mg, 90%; a yellow oil; Eluent: PE/EA = 10/1. ^{1}H NMR (400 MHz, $CDCl_3$, TMS) δ 2.40 (s, 3H), 4.00-4.60 (m, 2H), 5.10 (s, 1H), 5.64 (s, 1H), 6.85 (d, J = 6.4 Hz, 2H), 6.94 (d, J = 7.6 Hz, 1H), 7.03-7.35 (m, 13H), 7.50 (d, J = 7.6 Hz, 2H); ^{13}C{^{1}H}-NMR (101 MHz, $CDCl_3$, TMS) δ_C 21.5, 54.6, 117.2, 127.0, 127.6, 127.8, 127.9, 128.0, 128.06, 128.14, 129.2, 129.4, 130.8, 132.0, 135.5, 137.3, 137.4, 140.8, 142.3, 143.2, 145.9; IR (neat): ν 3060, 3028, 2921, 1596, 1493, 1342, 1154, 1089, 909, 813, 697 cm^{-1}; HRMS (ESI-TOF) Calcd for $C_{28}H_{25}NO_2NaS$ [M+Na]$^+$: 462.1498, found: 462.1508.

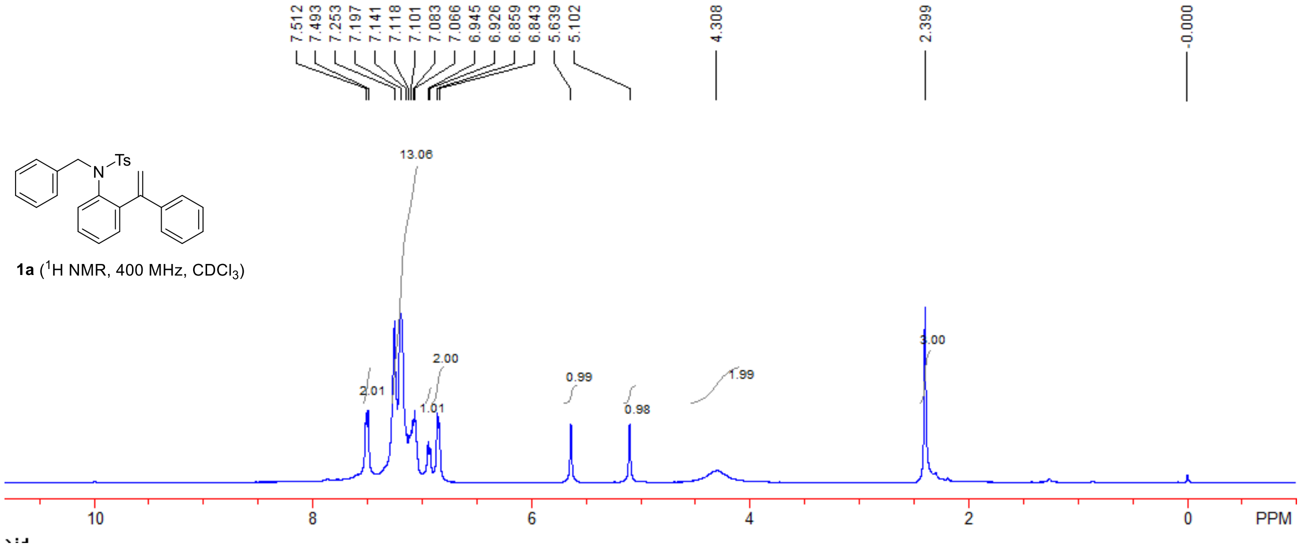

1a (^{1}H NMR, 400 MHz, $CDCl_3$)

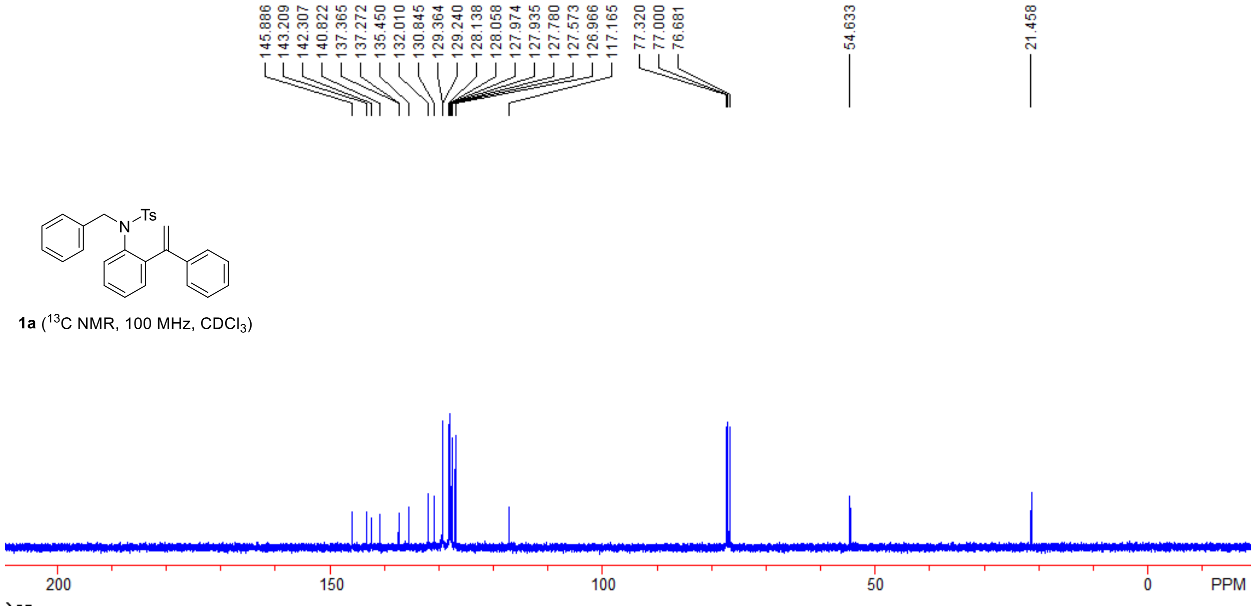

1a (^{13}C NMR, 100 MHz, $CDCl_3$)

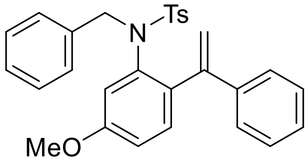

Compound 1b: Yield: 1338 mg, 80%; a yellow oil; Eluent: PE/EA = 5/1. ^{1}H NMR (400 MHz, $CDCl_3$, TMS) δ 2.33 (s, 3H), 3.52 (s, 3H), 4.00-4.60 (m, 2H), 5.12 (s, 1H), 5.58 (s, 1H), 6.42-6.47 (m, 1H), 6.77 (d, *J* = 8.0 Hz, 1H), 6.89 (d, *J* = 6.4 Hz, 2H), 7.02-7.12 (m, 4H), 7.13-7.27 (m, 7H), 7.51 (d, *J* = 8.0 Hz, 2H); $^{13}C\{^{1}H\}$-NMR (101 MHz, $CDCl_3$, TMS) δ_C 21.2, 54.5, 54.9, 113.8, 115.9, 116.7, 126.9, 127.3, 127.4, 127.84, 127.88, 127.91, 129.1, 129.2, 132.3, 134.3, 135.3, 137.2, 138.0, 141.1, 143.1, 145.5, 158.4; IR (neat): ν 3029, 2928, 2835, 1602, 1493, 1341, 1235, 1193, 1028, 908, 813, 694 cm^{-1}; HRMS (ESI-TOF) Calcd for $C_{29}H_{27}NO_3NaS$ $[M+Na]^+$: 492.1604, found: 492.1596.

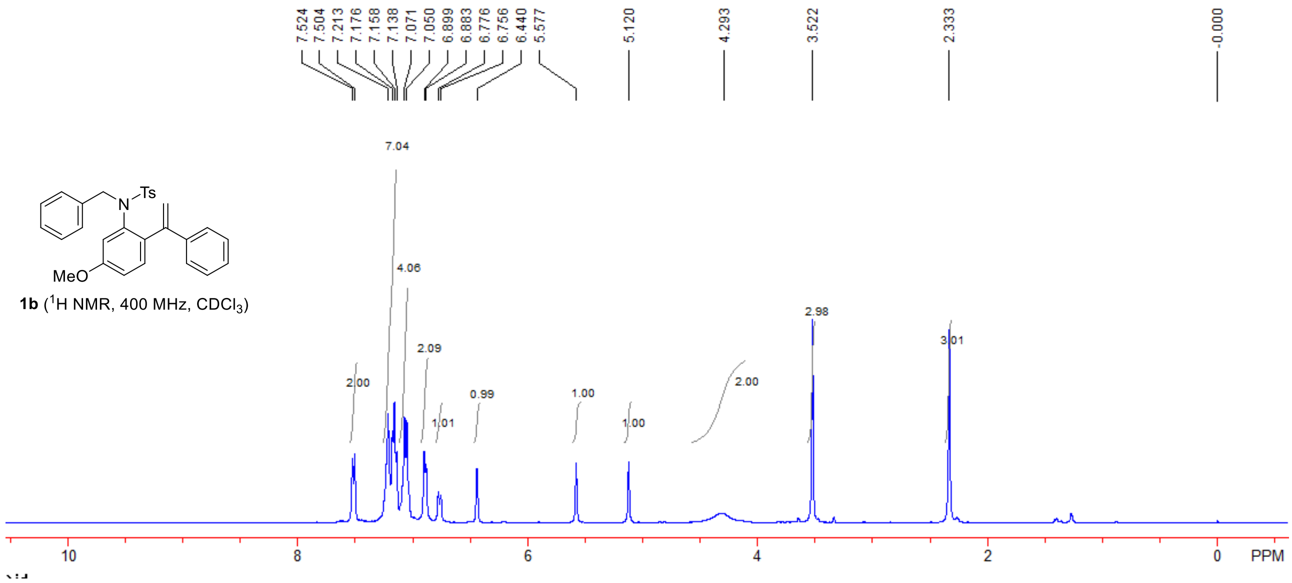

1b (^{1}H NMR, 400 MHz, $CDCl_3$)

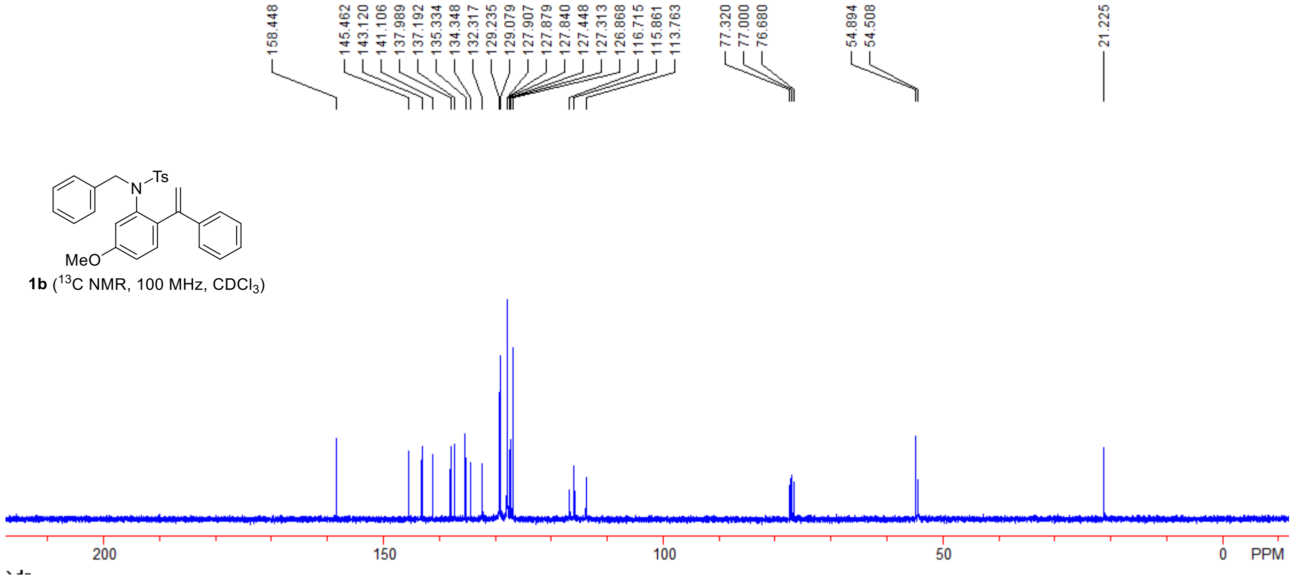

1b (^{13}C NMR, 100 MHz, $CDCl_3$)

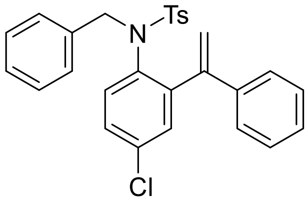

Compound 1c: Yield: 1812 mg, 88%; a yellow oil; Eluent: PE/EA = 10/1. ^{1}H NMR (400 MHz, $CDCl_3$, TMS) δ 2.39 (s, 3H), 4.00-4.60 (m, 2H), 5.07 (s, 1H), 5.62 (s, 1H), 6.81-6.87 (m, 3H), 7.04-7.22 (m, 9H), 7.24-7.31 (m, 3H), 7.50 (d, *J* = 8.0 Hz, 2H); ^{13}C{^{1}H}-NMR (101 MHz, $CDCl_3$, TMS) δ_C 21.4, 54.4, 117.8, 126.9, 127.7, 127.79, 127.83, 127.96, 128.04, 128.2, 129.3, 131.6, 132.2, 133.6, 135.0, 135.7, 136.9, 140.1, 143.4, 144.1, 144.8; IR (neat): ν 3028, 2973, 1596, 1493, 1342, 1154, 1088, 1027, 845, 813, 695 cm^{-1}; HRMS (ESI-TOF) Calcd for $C_{28}H_{24}NO_2NaSCl$ [M+Na]$^{+}$: 496.1109, found: 496.1117.

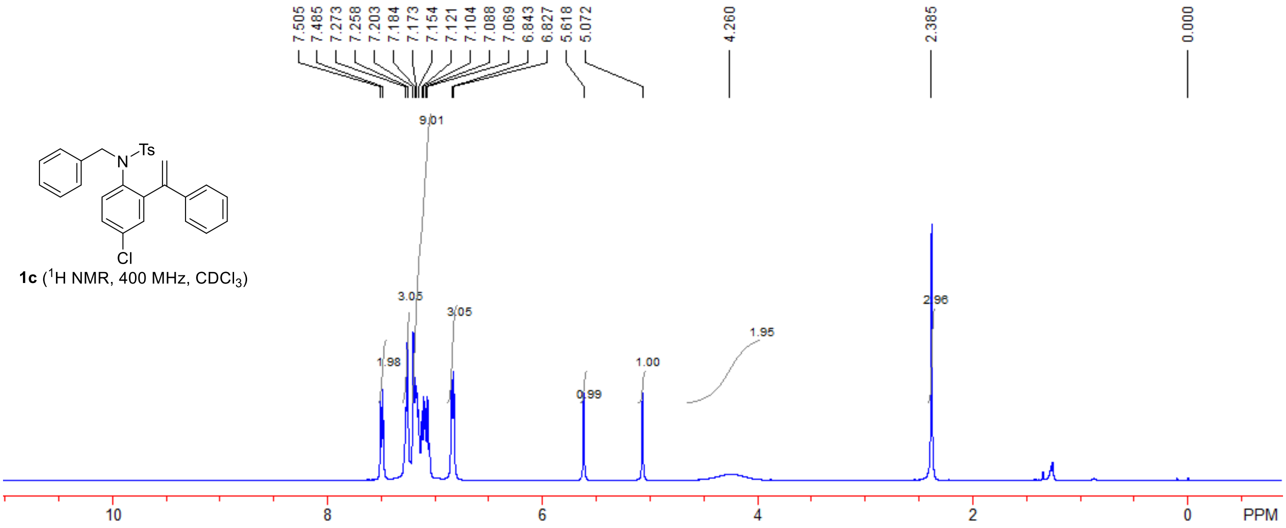
7.505
7.485
7.273
7.258
7.203
7.184
7.173
7.154
7.121
7.104
7.088
7.069
6.843
6.827
5.618
5.072
4.260
2.385
0.000
Ts
N
Cl
1c (1H NMR, 400 MHz, CDCl3)
9.01
3.05
3.05
1.98
1.95
1.00
0.99
2.96
10
8
6
4
2
0
PPM

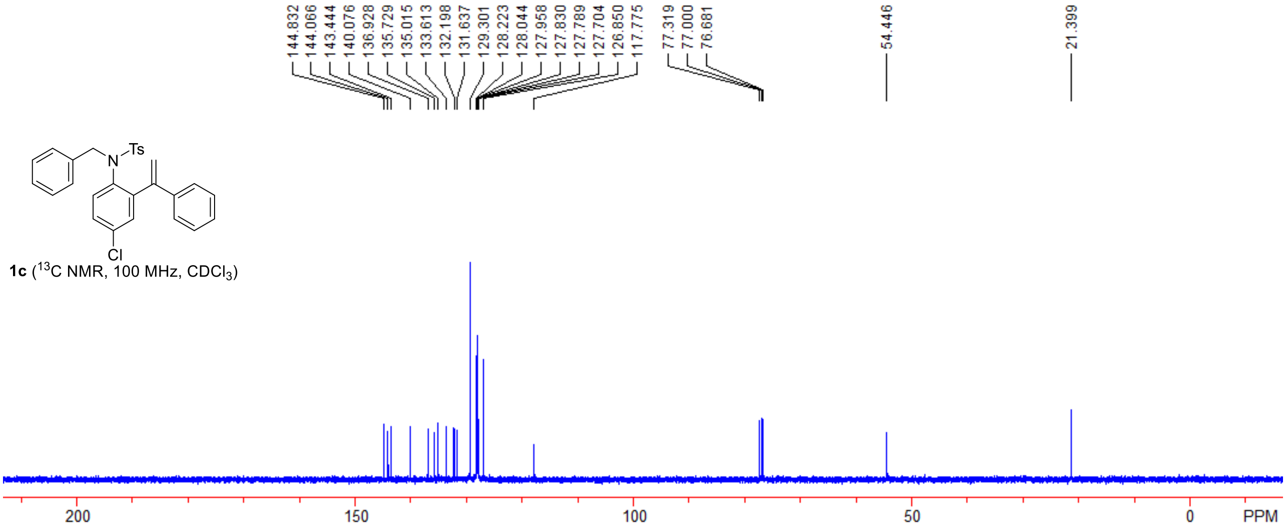
144.832
144.066
143.444
140.076
136.928
135.729
135.015
133.613
132.198
131.637
129.301
128.223
128.044
127.958
127.830
127.789
127.704
126.850
117.775
77.319
77.000
76.681
54.446
21.399
Ts
N
Cl
1c (13C NMR, 100 MHz, CDCl3)
200
150
100
50
0
PPM

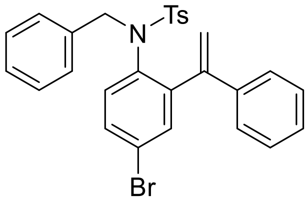

Compound 1d: Yield: 1506 mg, 85%; a yellow oil; Eluent: PE/EA = 10/1. ^{1}H NMR (400 MHz, $CDCl_3$, TMS) δ 2.35 (s, 3H), 4.00-4.60 (m, 2H), 5.07 (s, 1H), 5.61 (s, 1H), 6.79 (d, *J* = 8.8 Hz, 1H), 6.83 (d, *J* = 6.8 Hz, 2H), 7.02-7.12 (m, 3H), 7.13-7.19 (m, 4H), 7.21-7.28 (m, 4H), 7.34-7.36 (m, 1H), 7.49 (d, *J* = 8.0 Hz, 2H); ^{13}C{^{1}H}-NMR (101 MHz, $CDCl_3$, TMS) δ_C 21.3, 54.3, 117.7, 121.7, 126.7, 127.6, 127.7, 127.8, 127.9, 128.1, 129.18, 129.22, 130.7, 132.4, 134.4, 134.9, 136.2, 136.8, 139.9, 143.3, 144.3, 144.6; IR (neat): ν 3059, 3029, 2922, 1596, 1493, 1343, 1154, 1089, 1027, 813, 694 cm^{-1}; HRMS (ESI-TOF) Calcd for $C_{28}H_{24}NO_2NaSBr$ $[M+Na]^+$: 540.0603, found: 540.0600.

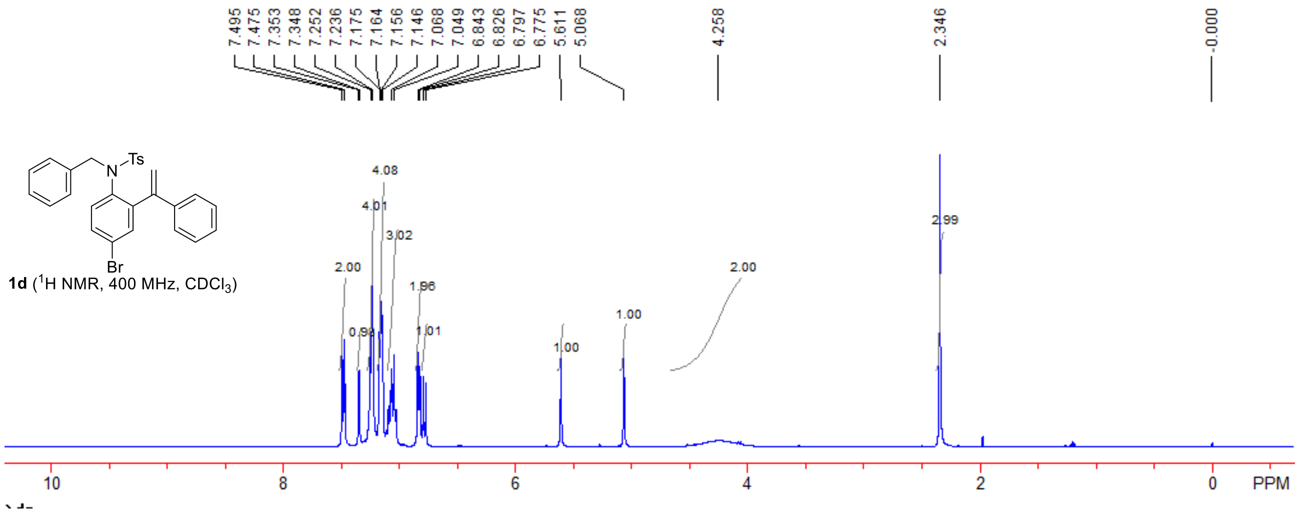

1d (^{1}H NMR, 400 MHz, $CDCl_3$)

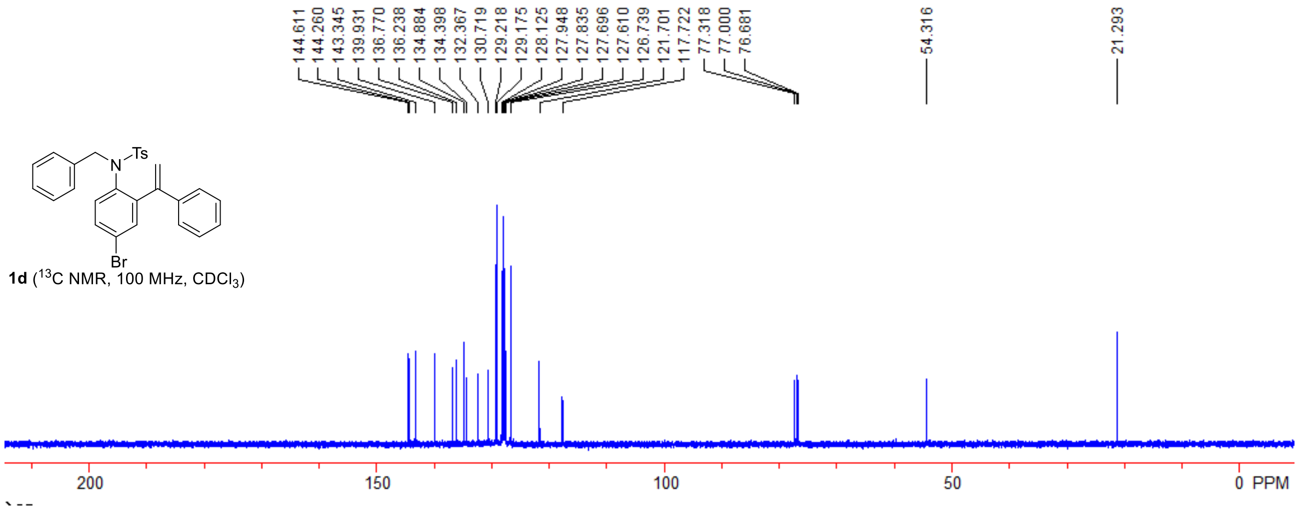

1d (^{13}C NMR, 100 MHz, $CDCl_3$)

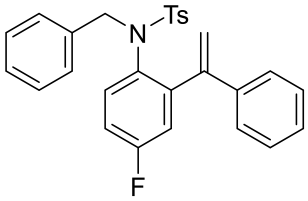

Compound 1e: Yield: 1581 mg, 93%; a yellow oil; Eluent: PE/EA = 10/1. ^{1}H NMR (400 MHz, $CDCl_3$, TMS) δ 2.34 (s, 3H), 3.90-4.60 (m, 2H), 5.10 (s, 1H), 5.60 (s, 1H), 6.75-6.91 (m, 5H), 7.02-7.10 (m, 3H), 7.13-7.19 (m, 4H), 7.20-7.28 (m, 3H), 7.51 (d, *J* = 8.0 Hz, 2H); ^{13}C{^{1}H}-NMR (101 MHz, $CDCl_3$, TMS) δ_C 21.2, 54.5, 114.4 (d, *J* = 21.9 Hz), 117.5, 118.3 (d, *J* = 22.1 Hz), 126.8, 127.56, 127.62, 127.8, 127.9, 128.1, 129.17, 129.25, 132.5 (d, *J* = 9.0 Hz), 133.0 (d, *J* = 2.9 Hz), 135.0, 136.9, 140.1, 143.3, 144.6 (d, *J* = 8.3 Hz), 144.8 (d, *J* = 0.9 Hz), 161.2 (d, *J* = 247.8 Hz); ^{19}F NMR (376 MHz, $CDCl_3$) δ -112.6; IR (neat): ν 3062, 3029, 2924, 1597, 1485, 1343, 1183, 1155, 1089, 939, 813, 698 cm^{-1}; HRMS (ESI-TOF) Calcd for $C_{28}H_{24}NO_2FNaS$ $[M+Na]^+$: 480.1404 found: 480.1404.

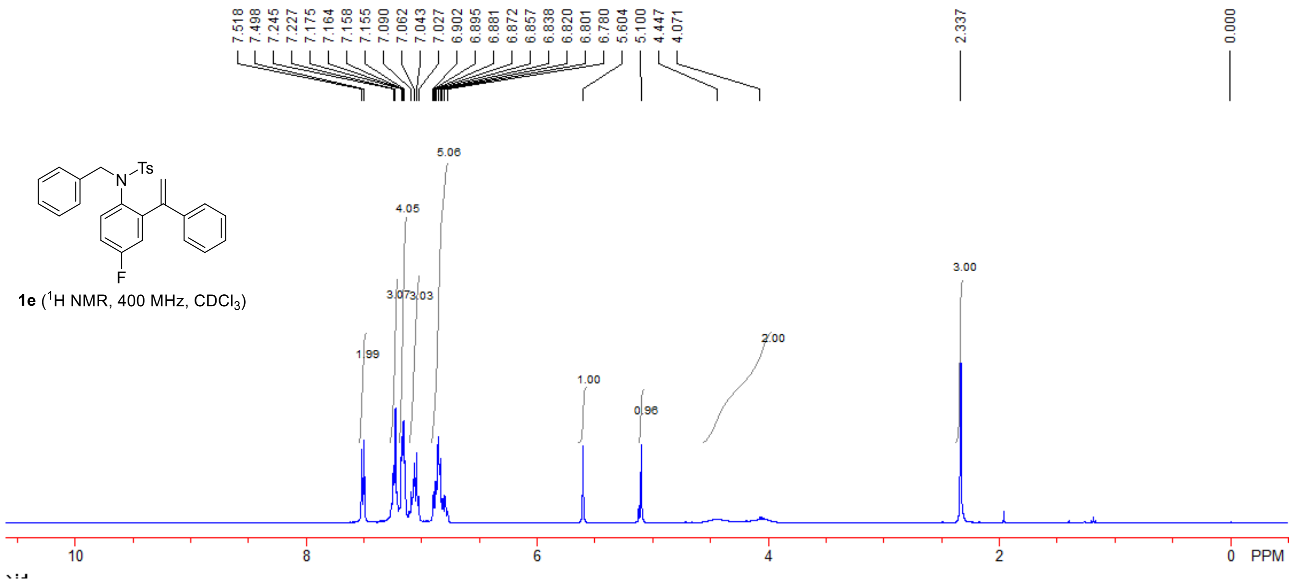

1e (¹H NMR, 400 MHz, CDCl₃)
PPM

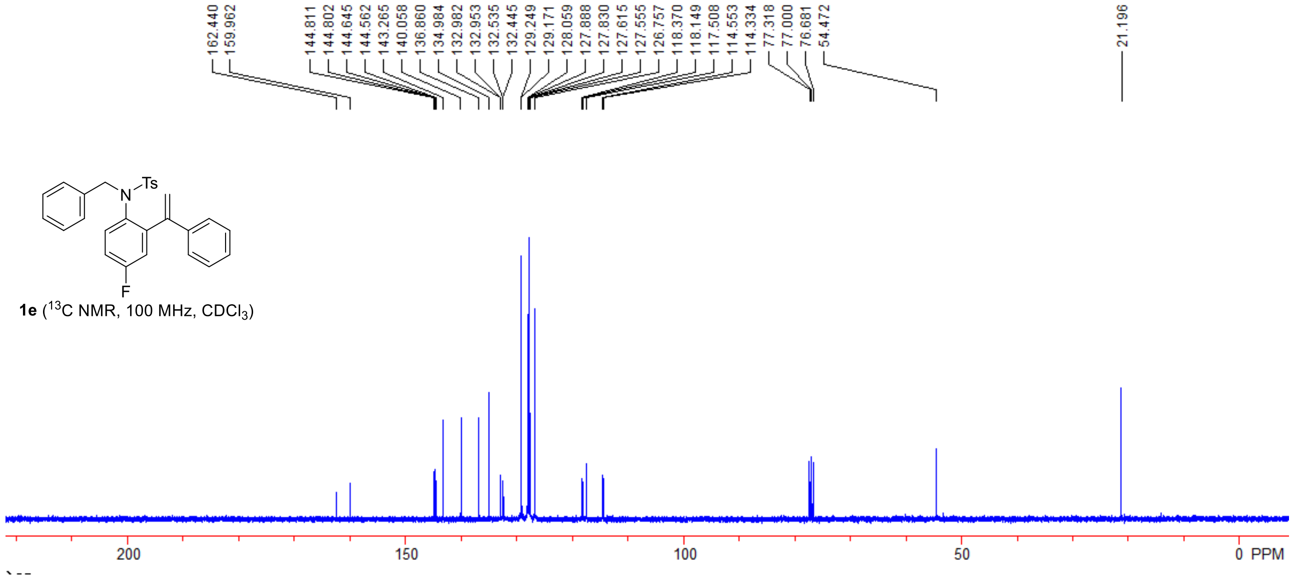

1e (¹³C NMR, 100 MHz, CDCl₃)
PPM

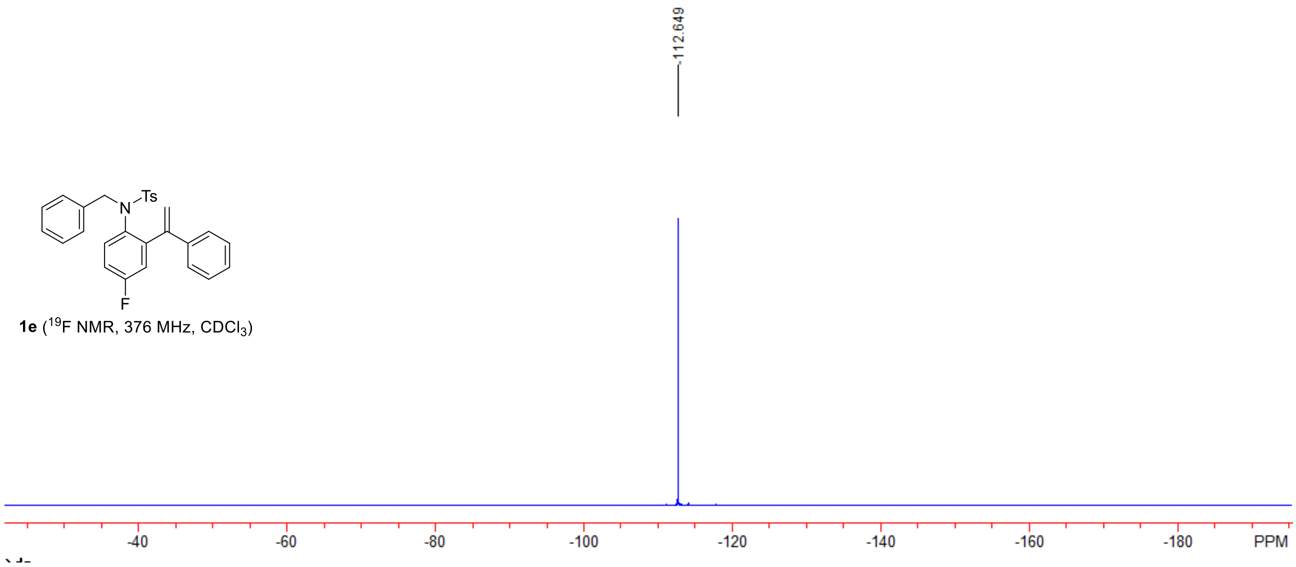

-112.649
1e (¹⁹F NMR, 376 MHz, CDCl₃)
PPM

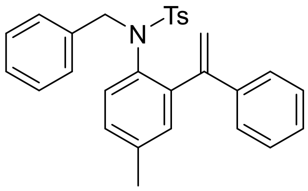

Compound 1f: Yield: 1668 mg, 90%; a yellow oil; Eluent: PE/EA = 10/1. ^{1}H NMR (400 MHz, $CDCl_3$, TMS) δ 2.23 (s, 3H), 2.35 (s, 3H), 4.00-4.60 (m, 2H), 5.09 (s, 1H), 5.61 (s, 1H), 6.81 (d, *J* = 8.0 Hz, 1H), 6.86 (d, *J* = 8.8 Hz, 2H), 6.93 (d, *J* = 8.4 Hz, 1H), 6.97-7.00 (m, 1H), 7.01-7.10 (m, 3H), 7.12-7.26 (m, 7H), 7.51 (d, *J* = 7.6 Hz, 2H); ^{13}C{^{1}H}-NMR (101 MHz, $CDCl_3$, TMS) δ_C 20.8, 21.3, 54.5, 116.8, 126.8, 127.37, 127.40, 127.82, 127.88, 128.0, 128.4, 129.1, 129.2, 130.4, 132.3, 134.4, 135.4, 137.3, 137.6, 140.8, 141.9, 143.0, 145.8; IR (neat): ν 3028, 2919, 2864, 1597, 1492, 1444, 1304, 1154, 1090, 906, 813, 698 cm^{-1}; HRMS (ESI-TOF) Calcd for $C_{29}H_{27}NO_2NaS$ $[M+Na]^+$: 476.1655, found: 476.1673.

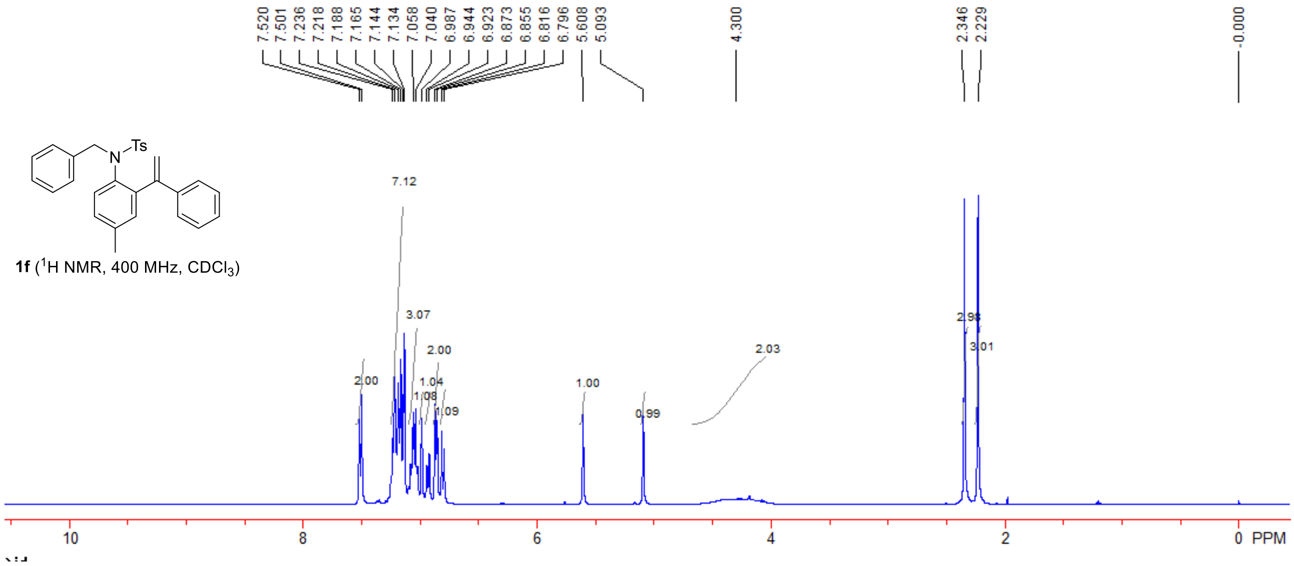

1f (^{1}H NMR, 400 MHz, $CDCl_3$)

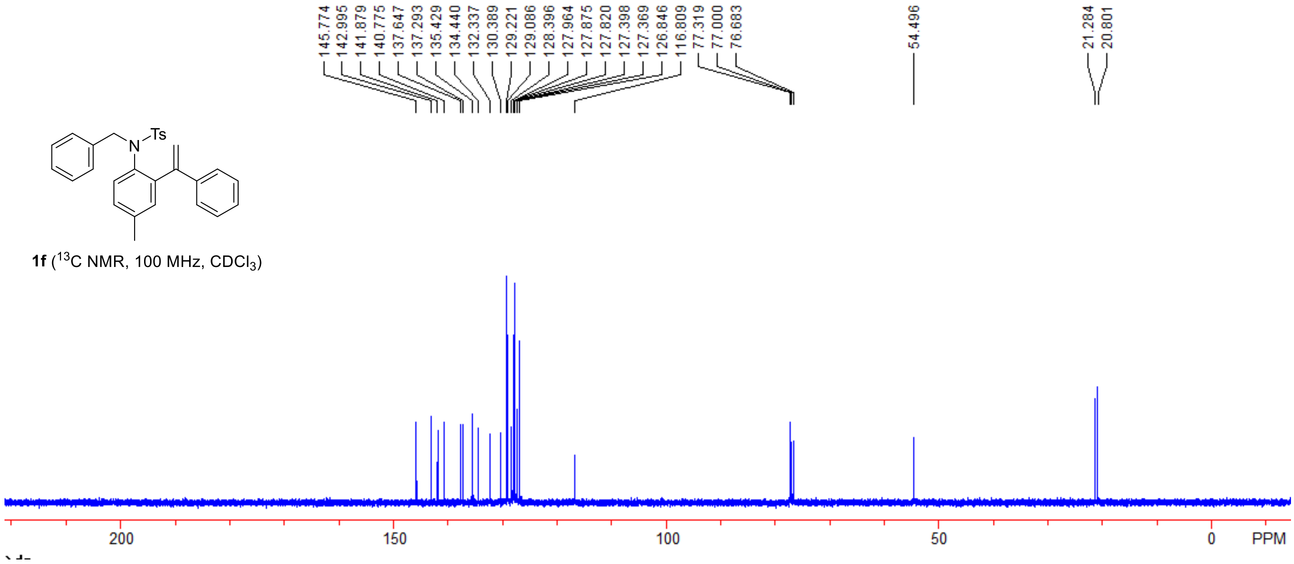

1f (^{13}C NMR, 100 MHz, $CDCl_3$)

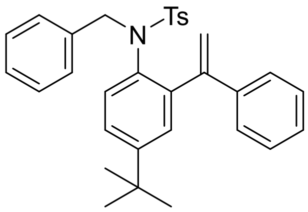

Compound 1g: Yield: 2142 mg, 88%; a yellow oil; Eluent: PE/EA = 10/1. ^{1}H NMR (400 MHz, $CDCl_3$, TMS) δ 1.24 (s, 9H), 2.32 (s, 3H), 4.00-4.60 (m, 2H), 5.15 (s, 1H), 5.66 (s, 1H), 6.84-6.94 (m, 3H), 6.99-7.08 (m, 3H), 7.12 (d, *J* = 8.0 Hz, 2H), 7.15-7.26 (m, 7H), 7.48 (d, *J* = 8.0 Hz, 2H); ^{13}C{^{1}H}-NMR (101 MHz, $CDCl_3$, TMS) δ_C 21.2, 30.9, 34.2, 54.6, 116.6, 124.5, 126.7, 127.26, 127.32, 127.7, 127.8, 127.9, 128.8, 128.97, 129.04, 130.0, 134.6, 135.5, 137.4, 140.6, 141.3, 142.8, 146.1, 150.5; IR (neat): ν 3029, 2960, 2867, 1597, 1493, 1454, 1343, 1155, 1090, 913, 853, 697 cm^{-1}; HRMS (ESI-TOF) Calcd for $C_{32}H_{33}NO_2NaS$ $[M+Na]^+$: 518.2124, found: 518.2120.

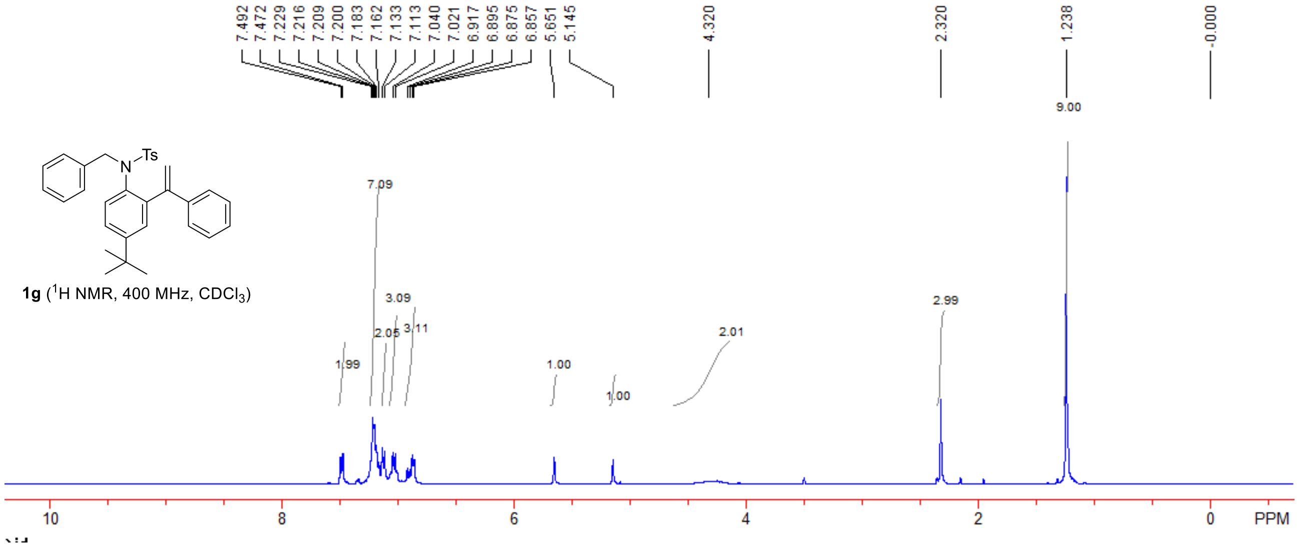

1g (^{1}H NMR, 400 MHz, $CDCl_3$)

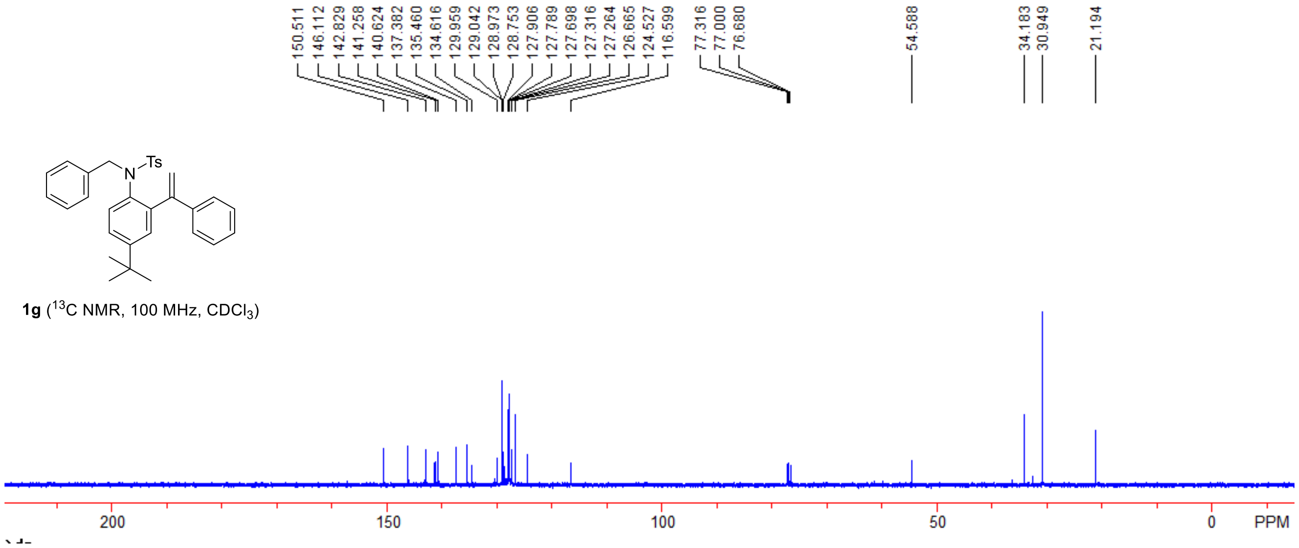

1g (^{13}C NMR, 100 MHz, $CDCl_3$)

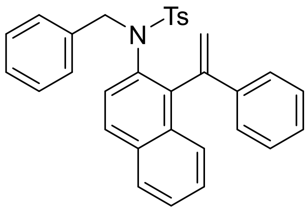

Compound 1h: Yield: 1866 mg, 79% as a pair of rotamers with 1:1 ratio because it has two rotation axles; a dark yellow solid; Mp: 144-146 °C; Eluent: PE/EA = 10/1. ^{1}H NMR (400 MHz, $CDCl_3$, TMS) δ 2.35 (s, 6H), 3.76-4.60 (m, 1.5H), 4.71 (s, 1H), 4.74 (s, 1H), 5.14-5.30 (m, 1.5H), 6.00-6.16 (m, 2H), 6.66-6.86 (m, 3H), 6.94-7.27 (m, 24H), 7.31 (t, *J* = 7.6 Hz, 3H), 7.40 (t, *J* = 7.2 Hz, 3H), 7.57-7.67 (m, 4H), 7.75 (d, *J* = 8.0 Hz, 2H), 7.85 (d, *J* = 8.4 Hz, 2H); ^{13}C{^{1}H}-NMR (101 MHz, $CDCl_3$, TMS) δ_C 21.4, 53.9, 119.0, 126.25, 126.28, 126.4, 127.0, 127.47, 127.59, 127.61, 127.7, 127.8, 127.9, 128.3, 128.6, 129.2, 129.7, 129.9, 130.6, 132.7, 133.3, 134.3, 135.5, 139.7, 143.1; IR (neat): ν 3061, 3029, 2931, 1596, 1493, 1359, 1279, 1217, 1150, 1066, 960, 817, 698 cm^{-1}; HRMS (ESI-TOF) Calcd for $C_{32}H_{27}NO_2NaS$ $[M+Na]^+$: 512.1655, found: 512.1660.

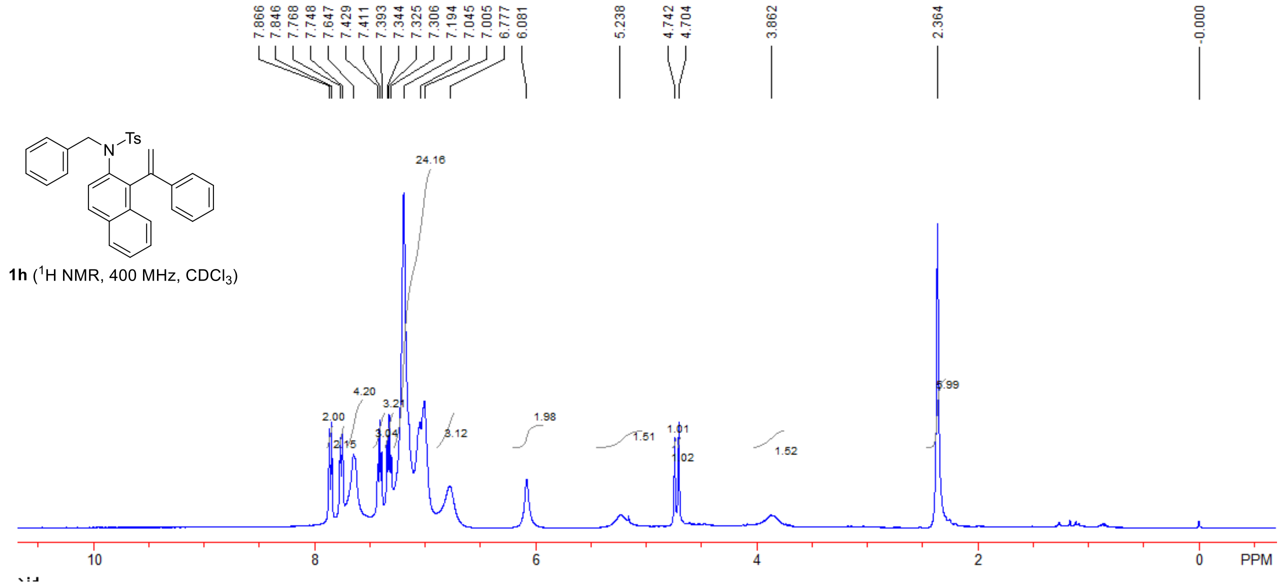

1h (^{1}H NMR, 400 MHz, $CDCl_3$)

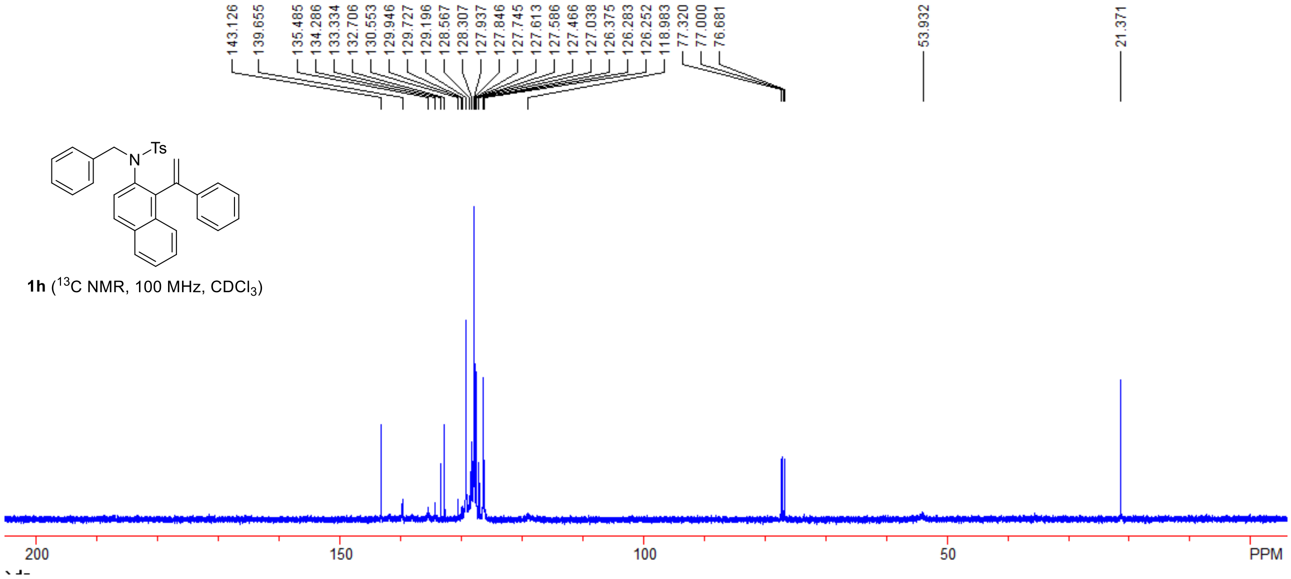

1h (^{13}C NMR, 100 MHz, $CDCl_3$)

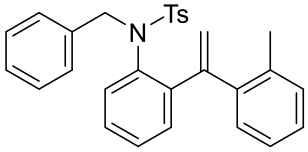

Compound 1i: Yield: 1226 mg, 81%; a light yellow solid; Mp: 96-98 °C; Eluent: PE/EA = 10/1. ^{1}H NMR (400 MHz, $CDCl_3$, TMS) δ 2.13 (s, 3H), 2.38 (s, 3H), 3.50-3.67 (m, 1H), 4.37-4.50 (m, 1H), 5.30 (s, 1H), 5.49 (s, 1H), 6.51 (d, *J* = 7.6 Hz, 1H), 6.82 (d, *J* = 7.6 Hz, 2H), 6.99-7.19 (m, 10H), 7.23 (t, *J* = 7.6 Hz, 1H), 7.32 (d, *J* = 7.6 Hz, 1H), 7.45 (d, *J* = 7.6 Hz, 2H); ^{13}C{^{1}H}-NMR (101 MHz, $CDCl_3$, TMS) δ_C 20.7, 21.2, 54.0, 120.7, 125.5, 127.16, 127.24, 127.5, 127.8, 128.0, 128.2, 129.0, 129.78, 129.84, 130.2, 130.9, 132.0, 135.2, 135.5, 136.3, 136.9, 141.5, 143.0, 143.6, 147.6; IR (neat): ν 3063, 3031, 1597, 1485, 1369, 1155, 1032, 949, 823, 771, 699 cm^{-1}; HRMS (ESI-TOF) Calcd for $C_{29}H_{27}NO_2NaS$ [M+Na]$^+$: 476.1655, found: 476.1658.

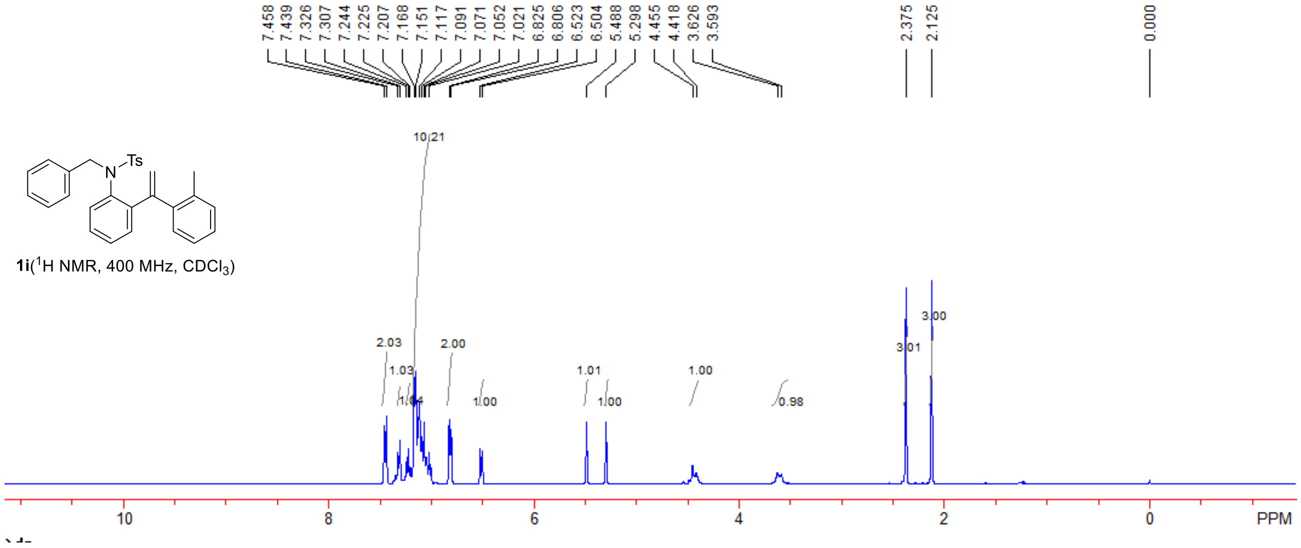

1i(^{1}H NMR, 400 MHz, $CDCl_3$)

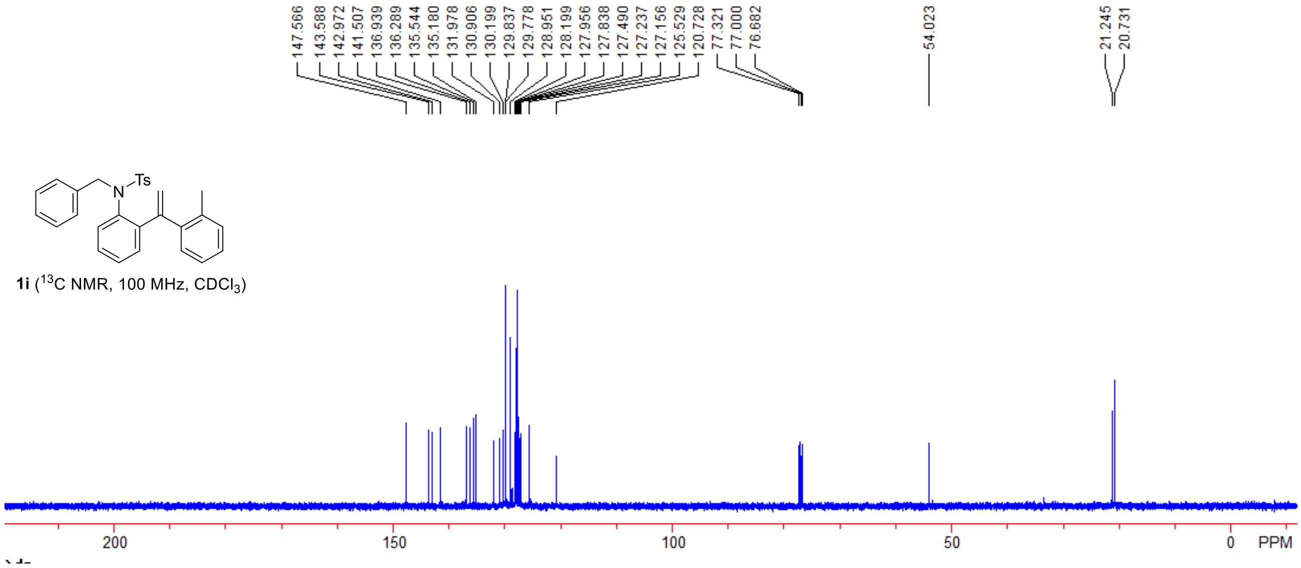

1i (^{13}C NMR, 100 MHz, $CDCl_3$)

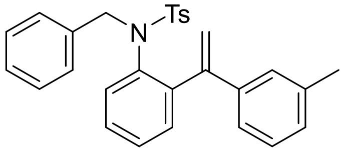

Compound 1j: Yield: 1546 mg, 69%; a yellow oil; Eluent: PE/EA = 10/1. ^{1}H NMR (400 MHz, $CDCl_3$, TMS) δ 2.27 (s, 3H), 2.40 (s, 3H), 4.00-4.60 (m, 2H), 5.10 (s, 1H), 5.63 (s, 1H), 6.85 (d, *J* = 7.6 Hz, 2H), 6.92-6.97 (m, 2H), 6.99 (d, *J* = 7.6 Hz, 1H), 7.04-7.27 (m, 10H), 7.51 (d, *J* = 7.2 Hz, 2H); ^{13}C{^{1}H}-NMR (101 MHz, $CDCl_3$, TMS) δ_C 21.38, 21.44, 54.5, 117.0, 124.1, 127.5, 127.67, 127.72, 127.89, 127.93, 128.0, 128.4, 129.2, 129.3, 131.0, 132.0, 135.5, 137.1, 137.5, 137.6, 140.7, 142.3, 143.2, 146.0; IR (neat): ν 3029, 2920, 1597, 1485, 1445, 1400, 1341, 1304, 1155, 1040, 908, 767, 698 cm^{-1}; HRMS (ESI-TOF) Calcd for $C_{29}H_{27}NO_2NaS$ $[M+Na]^+$: 476.1655, found: 476.1646.

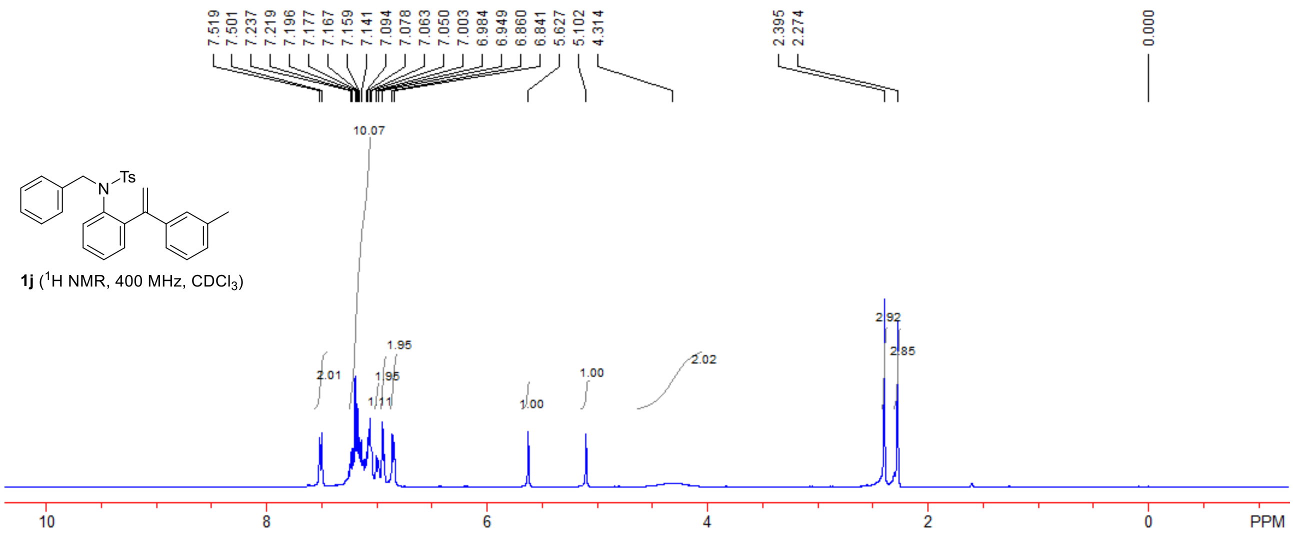

1j (^{1}H NMR, 400 MHz, $CDCl_3$)

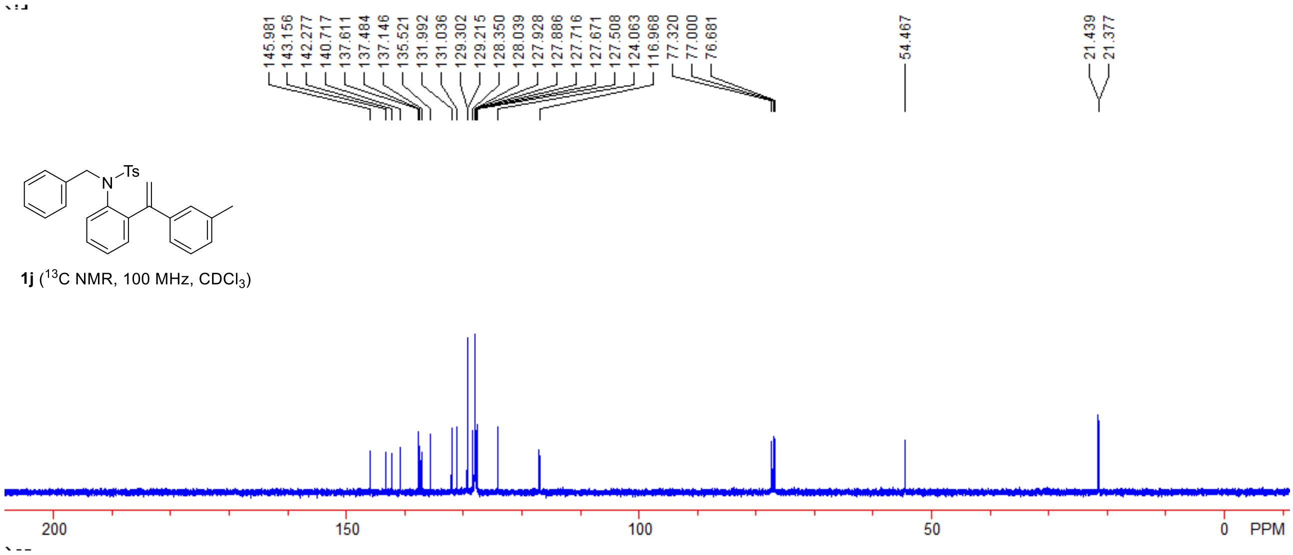

1j (^{13}C NMR, 100 MHz, $CDCl_3$)

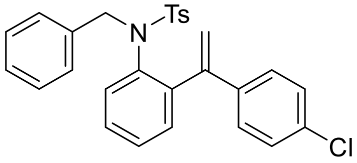

Compound 1k: Yield: 1920 mg, 80%; a light yellow solid; Mp: 113-115 °C; Eluent: PE/EA = 10/1. ^{1}H NMR (400 MHz, $CDCl_3$, TMS) δ 2.38 (s, 3H), 4.00-4.60 (m, 2H), 5.13 (s, 1H), 5.62 (s, 1H), 6.86 (d, *J* = 7.2 Hz, 2H), 6.95 (d, *J* = 7.2 Hz, 1H), 7.02-7.12 (m, 5H), 7.12-7.24 (m, 7H), 7.51 (d, *J* = 8.0 Hz, 2H); ^{13}C{^{1}H}-NMR (101 MHz, $CDCl_3$, TMS) δ_C 21.4, 55.0, 117.7, 127.6, 127.92, 127.93, 127.98, 128.0, 128.15, 128.23, 129.3, 130.0, 131.8, 133.1, 135.0, 136.6, 137.4, 139.3, 141.9, 143.3, 144.6; IR (neat): ν 3066, 3029, 2902, 1593, 1455, 1395, 1309, 1258, 1148, 1066, 1016, 957, 877, 775 cm^{-1}; HRMS (ESI-TOF) Calcd for $C_{28}H_{24}NO_2NaSCl$ [M+Na]$^+$: 496.1109, found: 496.1114.

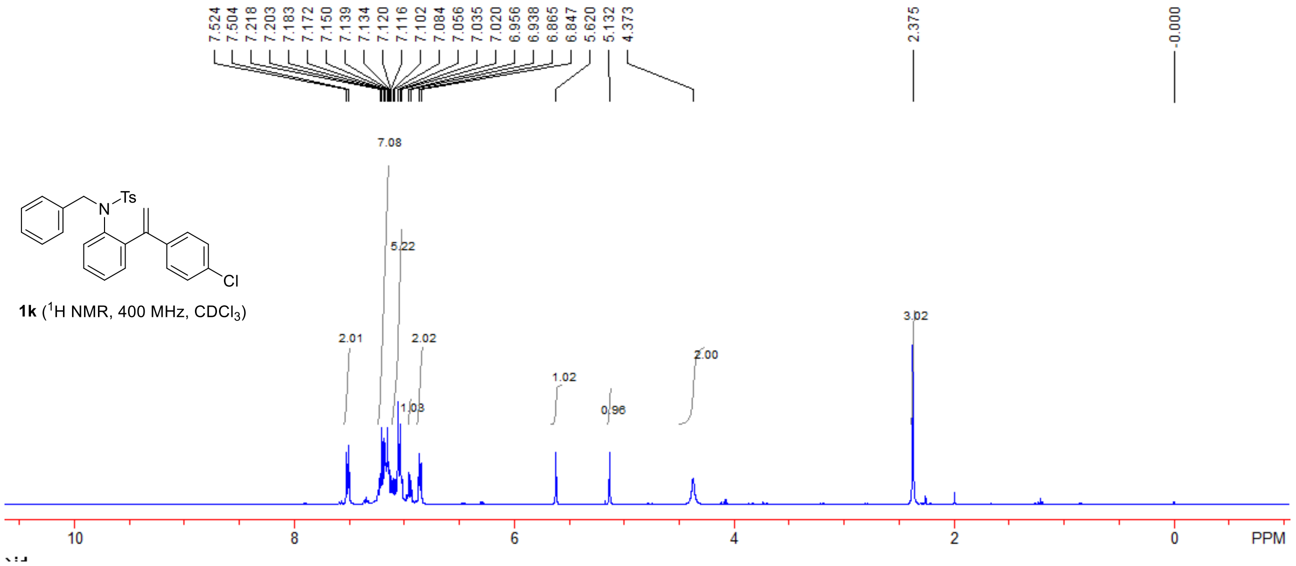
7.524 7.504 7.218 7.203 7.183 7.172 7.150 7.139 7.134 7.120 7.116 7.102 7.084 7.056 7.035 7.020 6.956 6.938 6.865 6.847 5.620 5.132 4.373
2.375
-0.000
7.08
5.22
2.01
2.02
1.03
1.02
0.96
2.00
3.02
Ts
N
Cl
1k (1H NMR, 400 MHz, CDCl3)
10
8
6
4
2
0
PPM

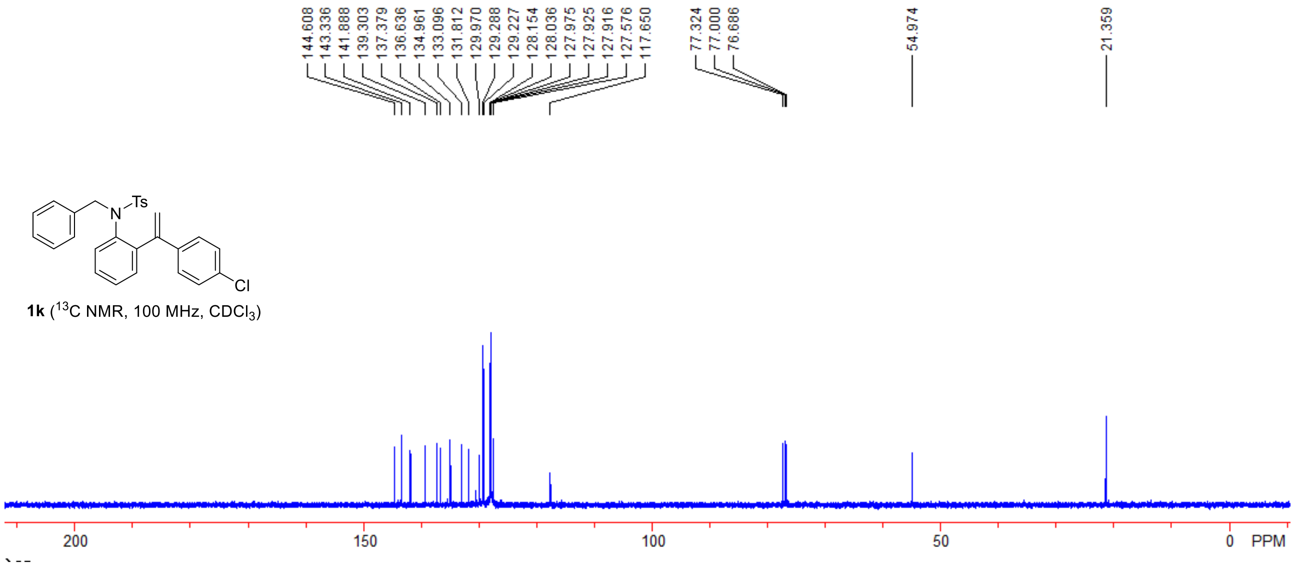
144.608 143.336 141.888 139.303 137.379 136.636 134.961 133.096 131.812 129.970 129.288 129.227 128.154 128.036 127.975 127.925 127.916 127.576 117.650
77.324 77.000 76.686
54.974
21.359
Ts
N
Cl
1k (13C NMR, 100 MHz, CDCl3)
200
150
100
50
0
PPM

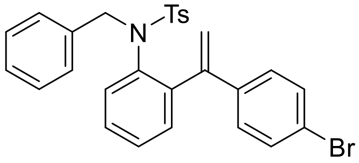

Compound 1l: Yield: 2214 mg, 85%; a light yellow solid; Mp: 108-110 °C; Eluent: PE/EA = 10/1. ^{1}H NMR (400 MHz, $CDCl_3$, TMS) δ 2.39 (s, 3H), 4.00-4.60 (m, 2H), 5.14 (s, 1H), 5.63 (s, 1H), 6.85 (d, *J* = 7.2 Hz, 2H), 6.92-7.02 (m, 3H), 7.04 (t, *J* = 7.6 Hz, 2H), 7.08-7.26 (m, 6H), 7.32 (d, *J* = 8.4 Hz, 2H), 7.51 (d, *J* = 8.0 Hz, 2H); ^{13}C{^{1}H}-NMR (101 MHz, $CDCl_3$, TMS) δ_C 21.4, 55.0, 117.8, 121.4, 127.6, 127.93, 127.96, 128.02, 128.5, 129.26, 129.33, 130.0, 131.0, 131.9, 135.0, 136.6, 137.4, 139.8, 141.8, 143.4, 144.7; IR (neat): ν 2987, 2901, 1593, 1454, 1365, 1290, 1149, 1112, 1076, 1030, 927, 876, 774, 702 cm^{-1}; HRMS (ESI-TOF) Calcd for $C_{28}H_{24}NO_2NaSBr$ [M+Na]$^+$: 540.0603, found: 540.0609.

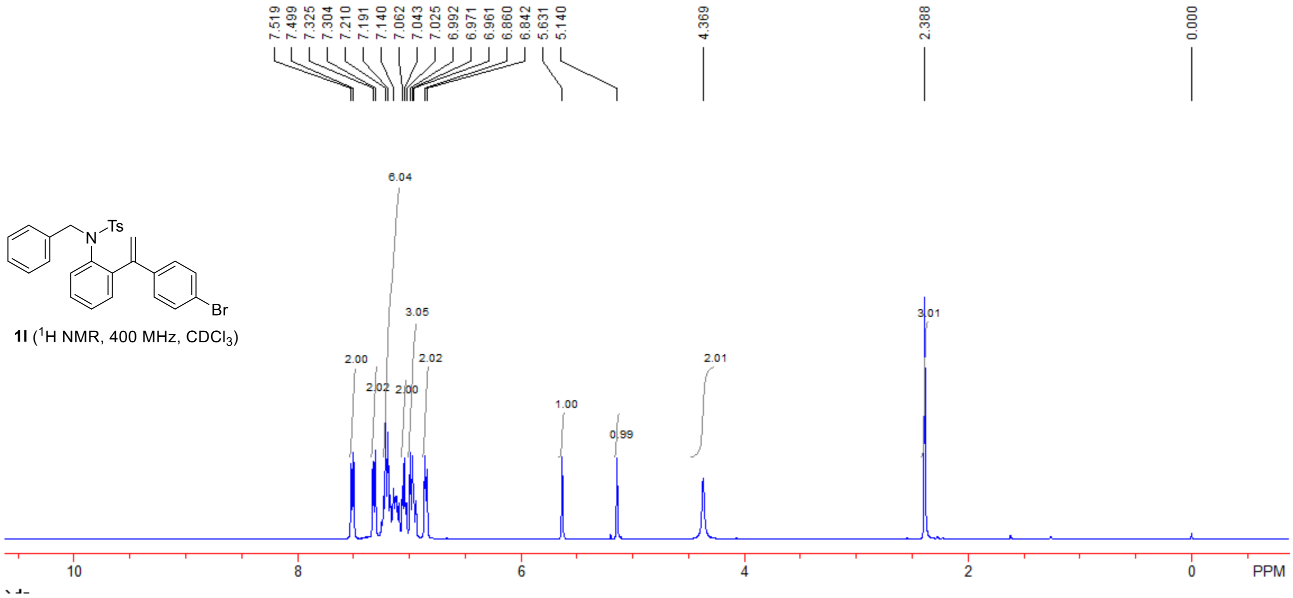

1l (^{1}H NMR, 400 MHz, $CDCl_3$)

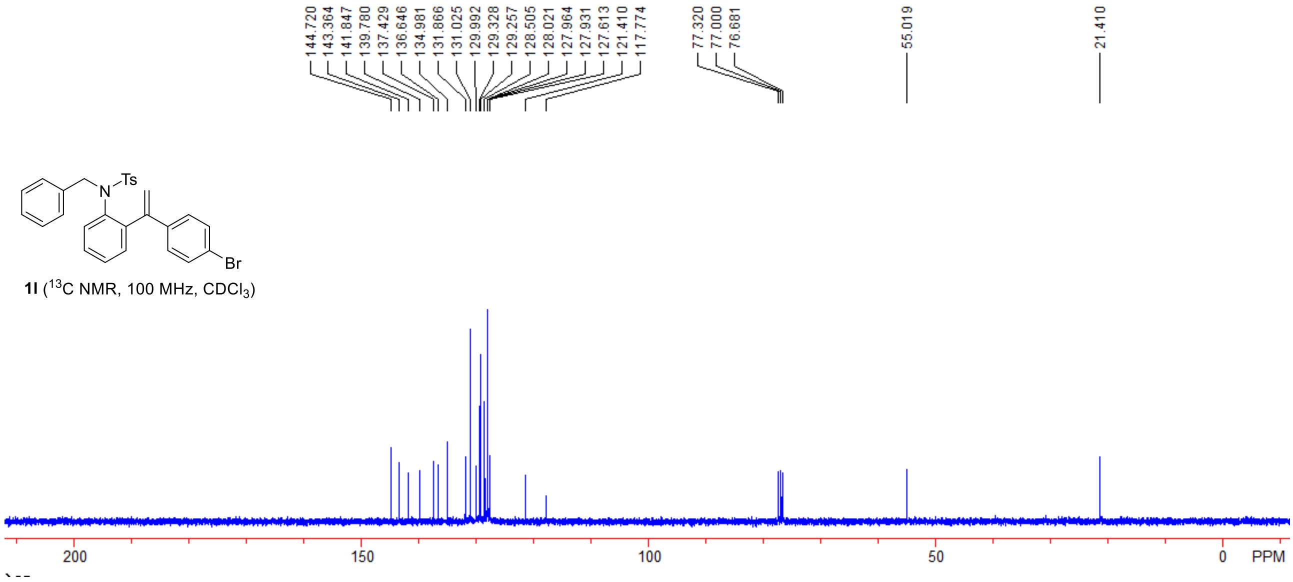

1l (^{13}C NMR, 100 MHz, $CDCl_3$)

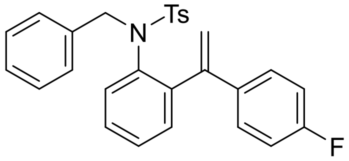

Compound 1m: Yield: 1628 mg, 93%; a light yellow solid; Mp: 104-106 °C; Eluent: PE/EA = 10/1. ^{1}H NMR (400 MHz, $CDCl_3$, TMS) δ 2.39 (s, 3H), 4.20-4.60 (m, 2H), 5.09 (s, 1H), 5.58 (s, 1H), 6.84-6.96 (m, 5H), 7.02-7.26 (m, 10H), 7.53 (d, *J* = 8.0 Hz, 2H); ^{13}C{^{1}H}-NMR (101 MHz, $CDCl_3$, TMS) δ_C 21.4, 54.9, 114.8 (d, *J* = 21.6 Hz), 117.0, 127.6, 127.88, 127.95, 127.98, 128.02, 128.6 (d, *J* = 7.5 Hz), 129.27, 129.34, 130.2, 131.9, 135.1, 136.9, 137.0 (d, *J* = 3.3 Hz), 137.4, 142.2, 143.4, 144.8, 162.2 (d, *J* = 243.7 Hz); ^{19}F NMR (376 MHz, $CDCl_3$) δ -114.7; IR (neat): ν 2986, 2903, 1597, 1485, 1400, 1337, 1289, 1154, 1086, 1030, 953, 924, 868, 775, 705 cm^{-1}; HRMS (ESI-TOF) Calcd for $C_{28}H_{24}NO_2FNaS$ [M+Na]$^+$: 480.1404, found: 480.1410.

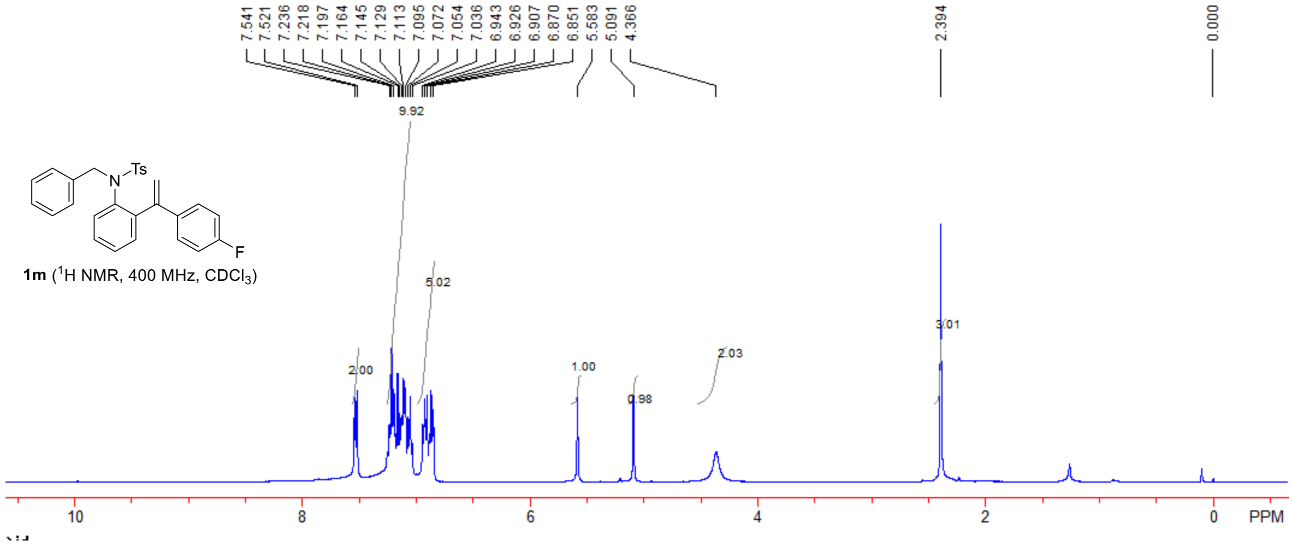

1m (^{1}H NMR, 400 MHz, $CDCl_3$)

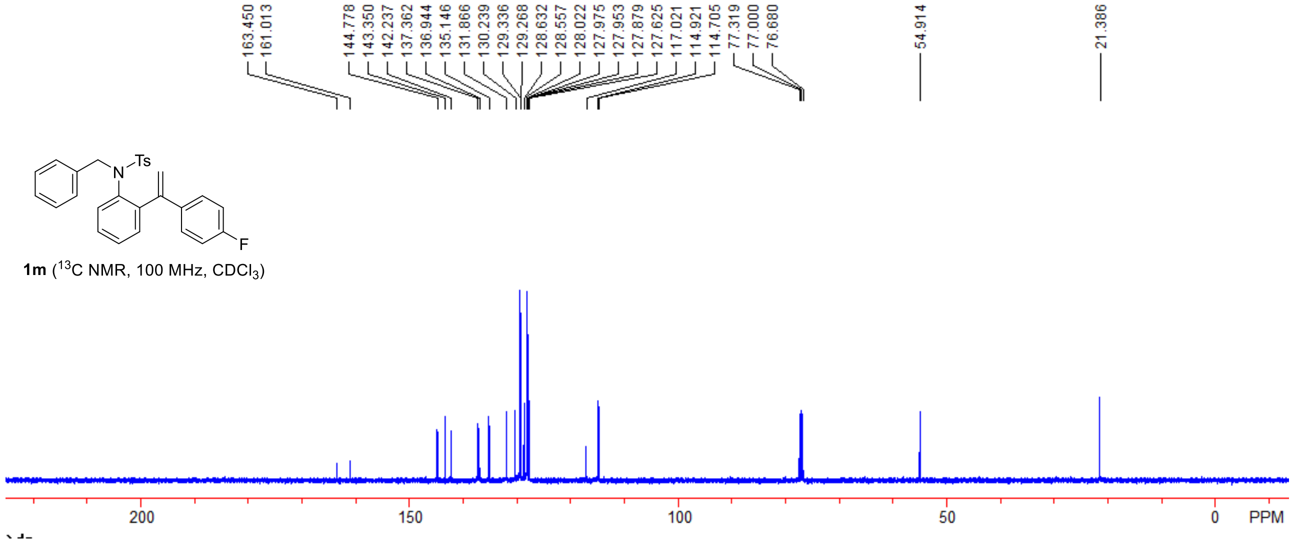

1m (^{13}C NMR, 100 MHz, $CDCl_3$)

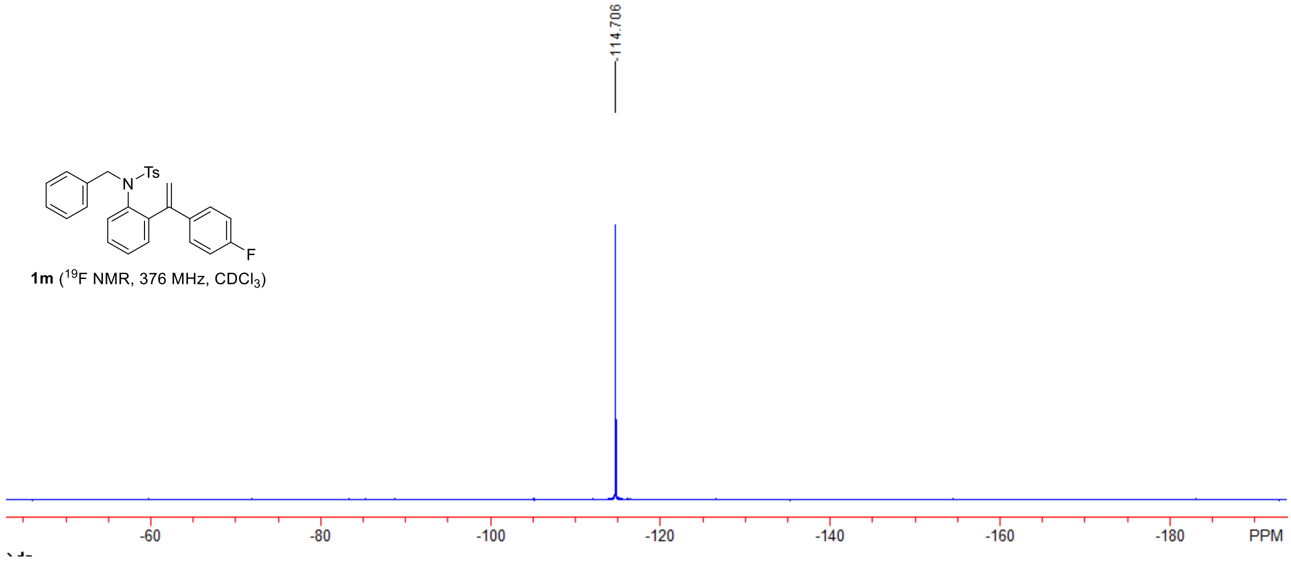

1m (^{19}F NMR, 376 MHz, $CDCl_3$)

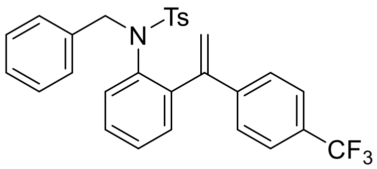

Compound 1n: Yield: 1338 mg, 91%; a yellow oil; Eluent: PE/EA = 10/1. ^{1}H NMR (400 MHz, $CDCl_3$, TMS) δ 2.35 (s, 3H), 4.35-4.45 (m, 2H), 5.25 (s, 1H), 5.72 (s, 1H), 6.85 (d, *J* = 7.2 Hz, 2H), 6.95-7.04 (m, 3H), 7.05-7.14 (m, 2H), 7.16-7.26 (m, 6H), 7.43 (d, *J* = 8.0 Hz, 2H), 7.52 (d, *J* = 8.0 Hz, 2H); ^{13}C{^{1}H}-NMR (101 MHz, $CDCl_3$, TMS) δ_C 21.2, 55.3, 119.3, 124.1 (q, *J* = 270.3 Hz), 124.8 (q, *J* =3.8 Hz), 127.1, 127.7, 127.91, 127.97, 128.03, 128.1, 128.9 (q, *J* = 32.0 Hz), 129.2, 129.3, 129.5, 131.8, 134.8, 136.3, 137.6, 141.7, 143.5, 144.4 (q, *J* =1.2 Hz), 144.5; ^{19}F NMR (376 MHz, $CDCl_3$) δ -62.1; IR (neat): ν 3030, 2924, 1615, 1597, 1487, 1321, 1157, 1114, 1063, 913, 849, 726, 698 cm^{-1}; HRMS (ESI-TOF) Calcd for $C_{29}H_{24}NO_2F_3NaS$ $[M+Na]^+$: 530.1372, found: 530.1366.

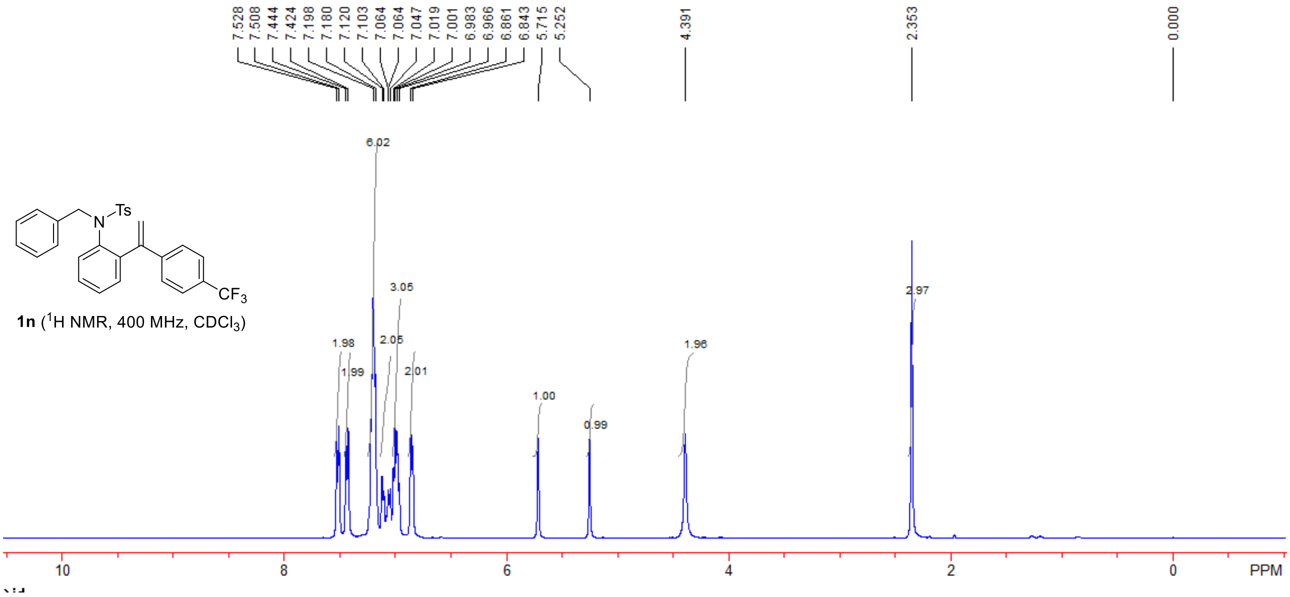

1n (^{1}H NMR, 400 MHz, $CDCl_3$)

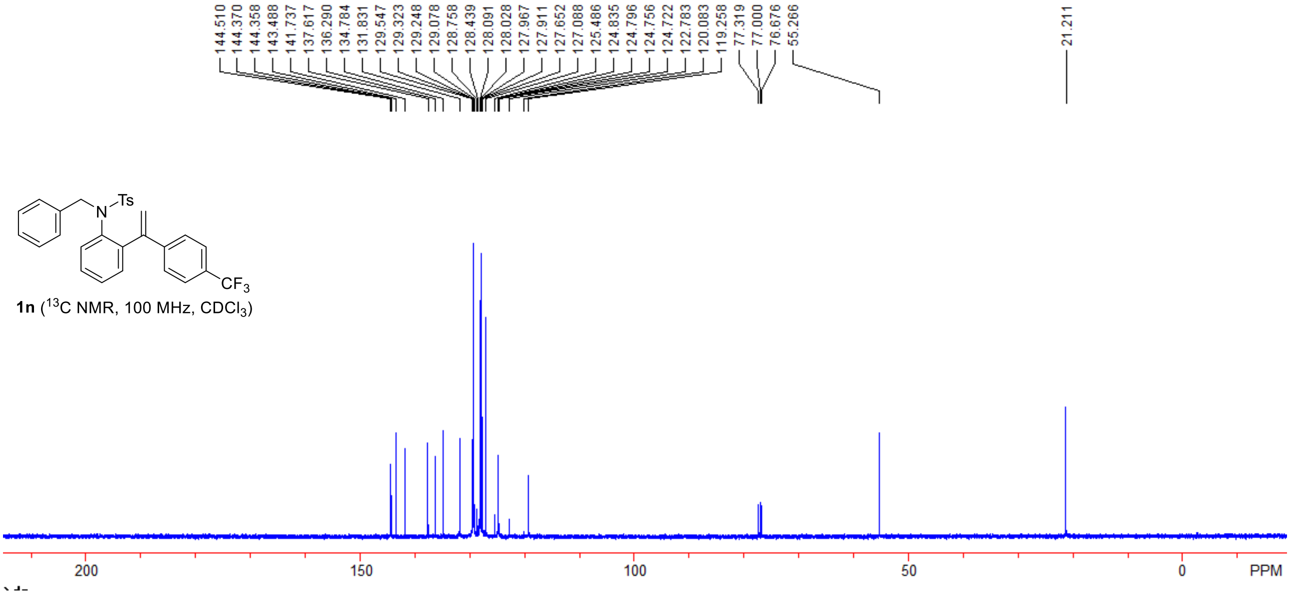

1n (^{13}C NMR, 100 MHz, $CDCl_3$)

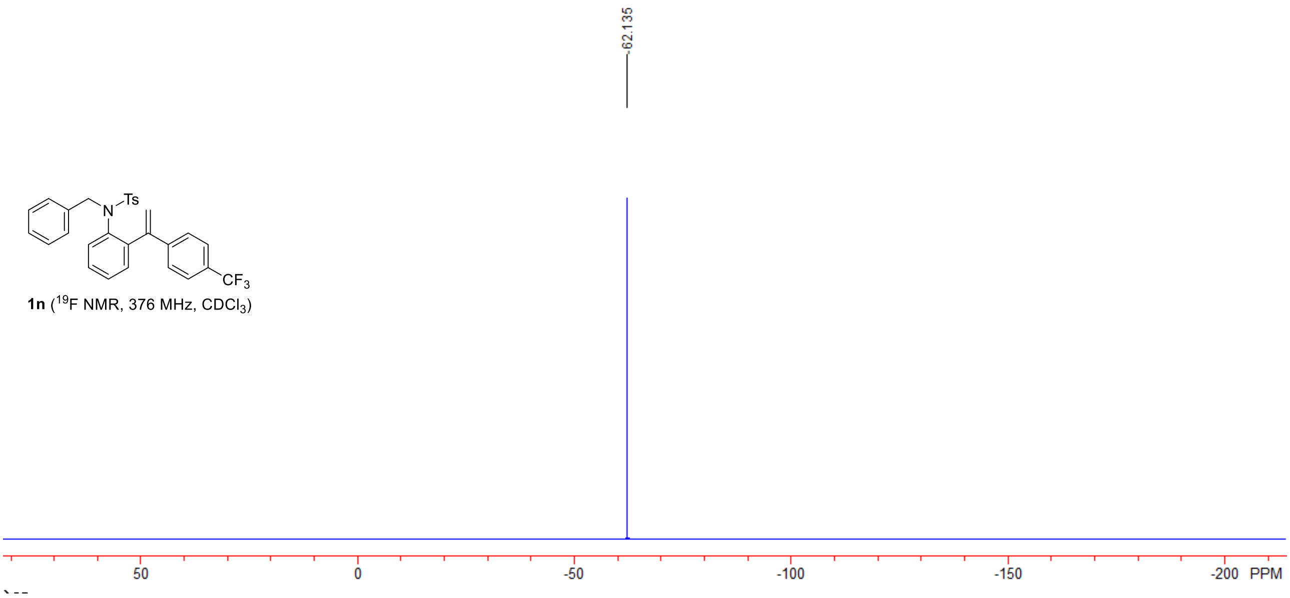

1n (^{19}F NMR, 376 MHz, $CDCl_3$)

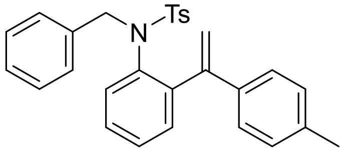

Compound 1o: Yield: 1642 mg, 80%; a light yellow solid; Mp: 102-104 ^{o}C; PE/EA = 10/1. ^{1}H NMR (400 MHz, $CDCl_3$, TMS) δ 2.30 (s, 3H), 2.36 (s, 3H), 4.00-4.60 (m, 2H), 5.05 (s, 1H), 5.61 (s, 1H), 6.87 (d, *J* = 6.8 Hz, 2H), 6.95 (d, *J* = 8.0 Hz, 1H), 7.02-7.24 (m, 12H), 7.50 (d, *J* = 8.4 Hz, 2H); ^{13}C{^{1}H}-NMR (101 MHz, $CDCl_3$, TMS) δ_C 21.0, 21.3, 54.5, 116.1, 126.7, 127.4, 127.6, 127.78, 127.87, 127.95, 128.7, 129.1, 129.3, 130.8, 131.9, 135.5, 137.20, 137.24, 137.3, 137.9, 142.3, 143.1, 145.6; IR (neat): ν 3064, 3011, 2984, 2920, 1597, 1486, 1442, 1369, 1272, 1210, 1198, 1159, 991, 880, 771, 726, 700 cm^{-1}; HRMS (ESI-TOF) Calcd for $C_{29}H_{27}NO_2NaS$ $[M+Na]^+$: 476.1655, found: 476.1662.

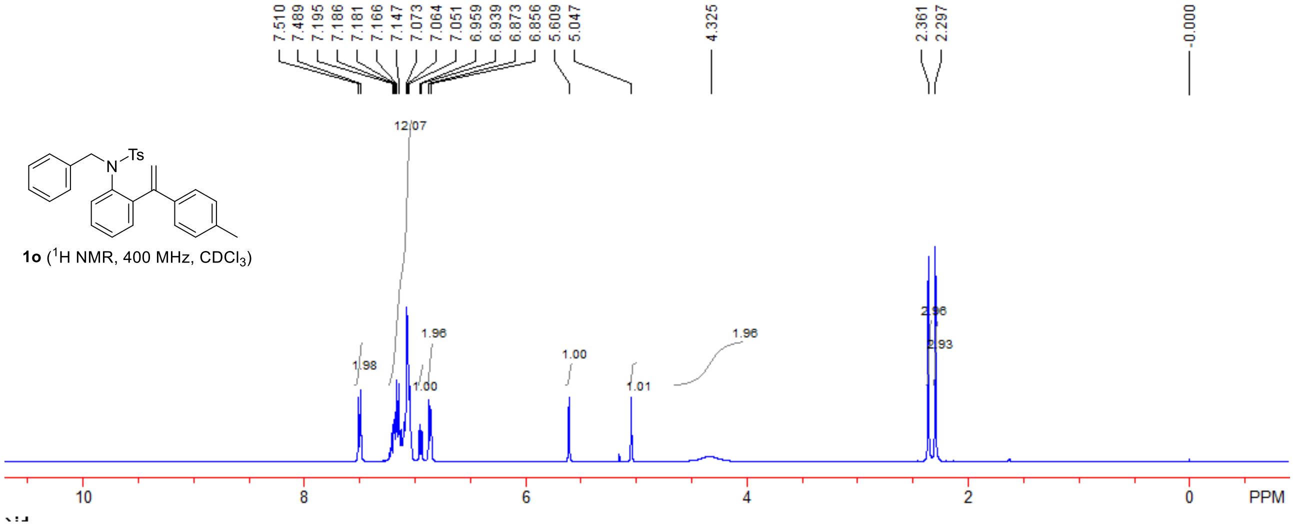
7.510 7.489 7.195 7.186 7.181 7.166 7.147 7.073 7.064 7.051 6.959 6.939 6.873 6.856 5.609 5.047
4.325
2.361 2.297
-0.000
Ts
N
1o (1H NMR, 400 MHz, CDCl3)
12.07
1.98
1.96
1.00
1.00
1.01
1.96
2.96
2.93
10
8
6
4
2
0
PPM

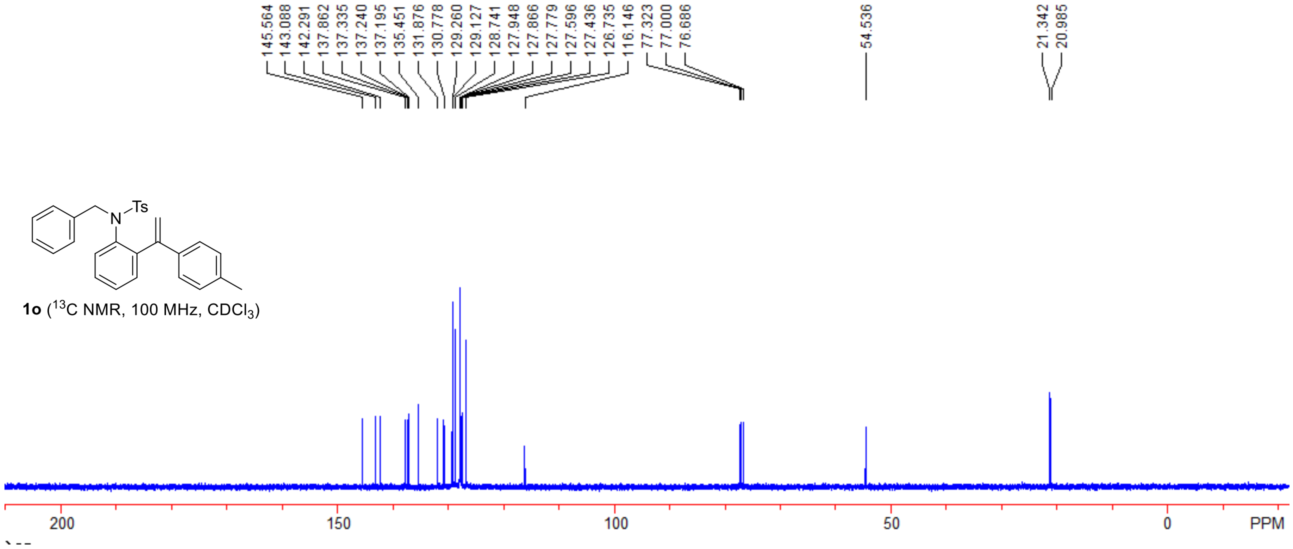
145.564 143.088 142.291 137.862 137.335 137.240 137.195 135.451 131.876 130.778 129.260 129.127 128.741 127.948 127.866 127.779 127.596 127.436 126.735 116.146 77.323 77.000 76.686
54.536
21.342 20.985
Ts
N
1o (13C NMR, 100 MHz, CDCl3)
200
150
100
50
0
PPM

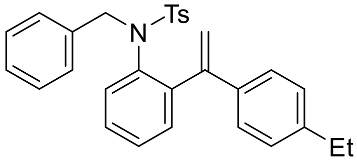

Compound 1p: Yield: 1906 mg, 89%; a yellow oil; Eluent: PE/EA = 10/1. ^{1}H NMR (400 MHz, $CDCl_3$, TMS) δ 1.20 (t, *J* = 7.6 Hz, 3H), 2.36 (s, 3H), 2.60 (q, *J* = 7.6 Hz, 2H), 4.00-4.60 (m, 2H), 5.06 (s, 1H), 5.62 (s, 1H), 6.86 (d, *J* = 6.8 Hz, 2H), 6.96 (d, *J* = 7.6 Hz, 1H), 7.02-7.25 (m, 12H), 7.50 (d, *J* = 8.0 Hz, 2H); ^{13}C{^{1}H}-NMR (101 MHz, $CDCl_3$, TMS) δ_C 15.4, 21.3, 28.3, 54.5, 116.1, 126.8, 127.4, 127.52, 127.58, 127.7, 127.8, 127.9, 129.1, 129.2, 130.7, 131.9, 135.4, 137.2, 137.3, 138.1, 142.3, 143.1, 143.6, 145.5; IR (neat): ν 3062, 3028, 2962, 2868, 1597, 1509, 1343, 1156, 1090, 863, 767 cm^{-1}; HRMS (ESI-TOF) Calcd for $C_{25}H_{27}NO_2NaS$ $[M+Na]^+$: 428.1655, found: 428.1652.

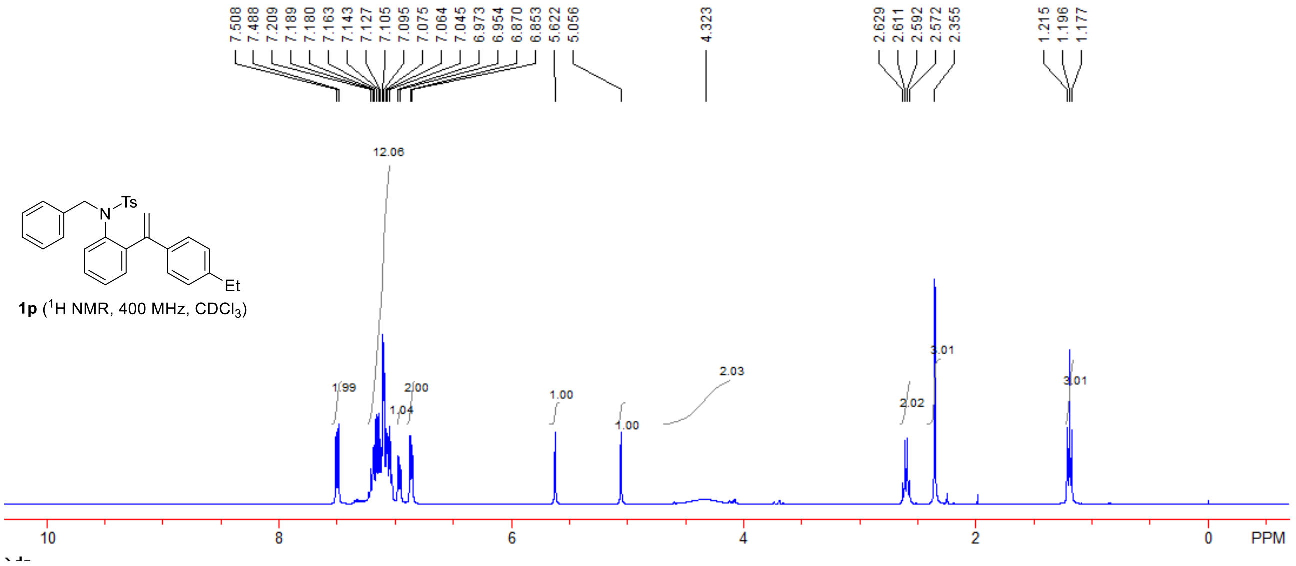

1p (^{1}H NMR, 400 MHz, $CDCl_3$)

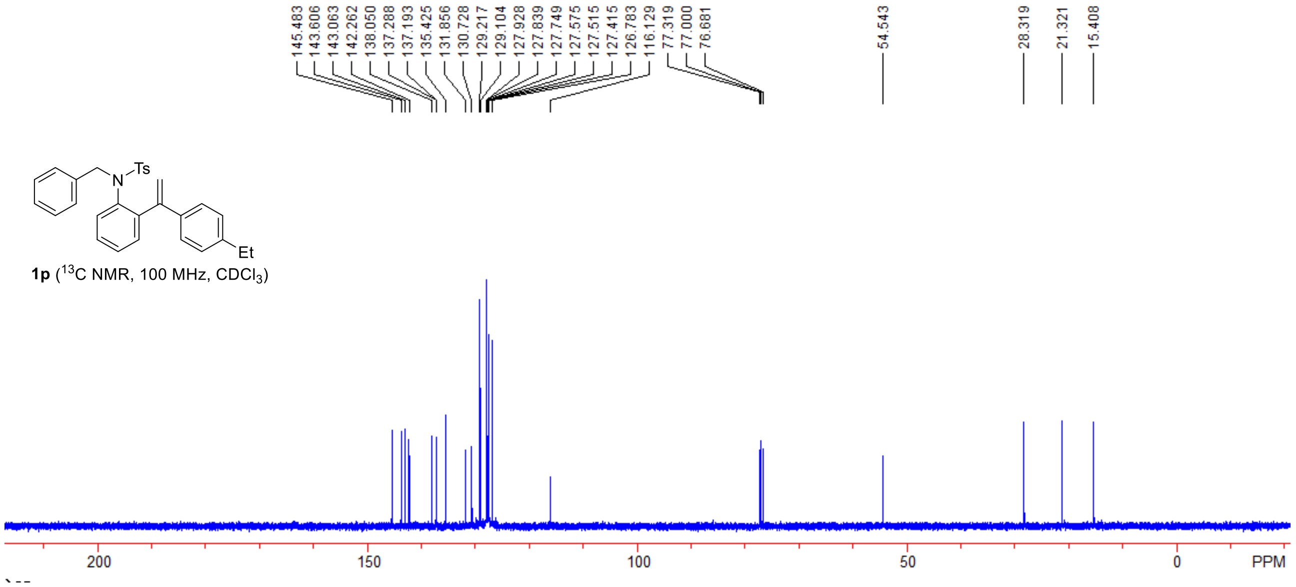

1p (^{13}C NMR, 100 MHz, $CDCl_3$)

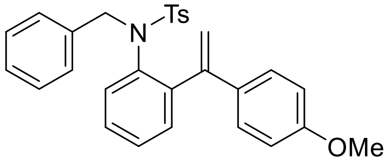

Compound 1q: Yield: 2014 mg, 88%; a yellow oil; Eluent: PE/EA = 5/1. ^{1}H NMR (400 MHz, $CDCl_3$, TMS) δ 2.39 (s, 3H), 3.75 (s, 3H), 4.00-4.70 (m, 2H), 4.99 (s, 1H), 5.56 (s, 1H), 6.79 (d, *J* = 8.8 Hz, 2H), 6.87 (d, *J* = 7.2 Hz, 2H), 6.95 (d, *J* = 7.6 Hz, 1H), 7.04-7.27 (m, 10H), 7.52 (d, *J* = 8.4 Hz, 2H); ^{13}C{^{1}H}-NMR (101 MHz, $CDCl_3$, TMS) δ_C 21.3, 54.5, 55.0, 113.3, 115.2, 127.4, 127.6, 127.8, 127.86, 127.90, 128.0, 129.1, 129.2, 130.7, 131.8, 133.3, 135.4, 137.1, 137.2, 142.3, 143.1, 145.0, 159.1; IR (neat): ν 3029, 2931, 2835, 1598, 1508, 1340, 1247, 1153, 1027, 835, 768, 697 cm^{-1}; HRMS (ESI-TOF) Calcd for $C_{29}H_{27}NO_3NaS$ $[M+Na]^+$: 492.1604, found: 492.1609.

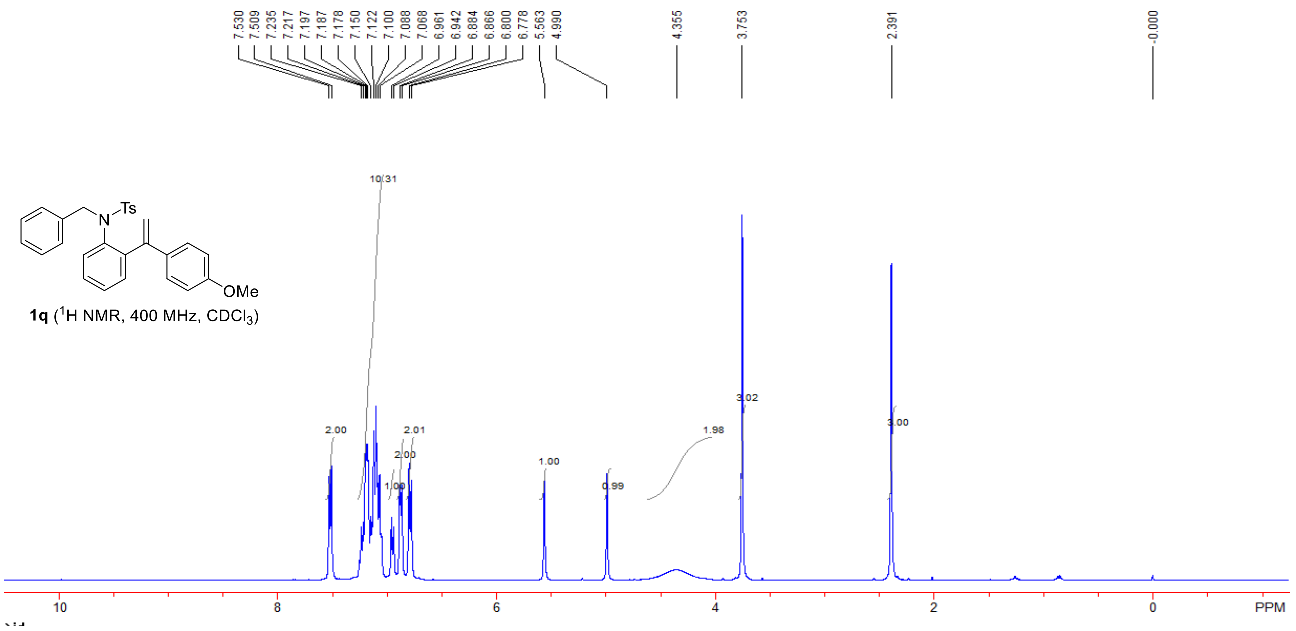

Ts
N
OMe
1q (^{1}H NMR, 400 MHz, $CDCl_3$)
PPM

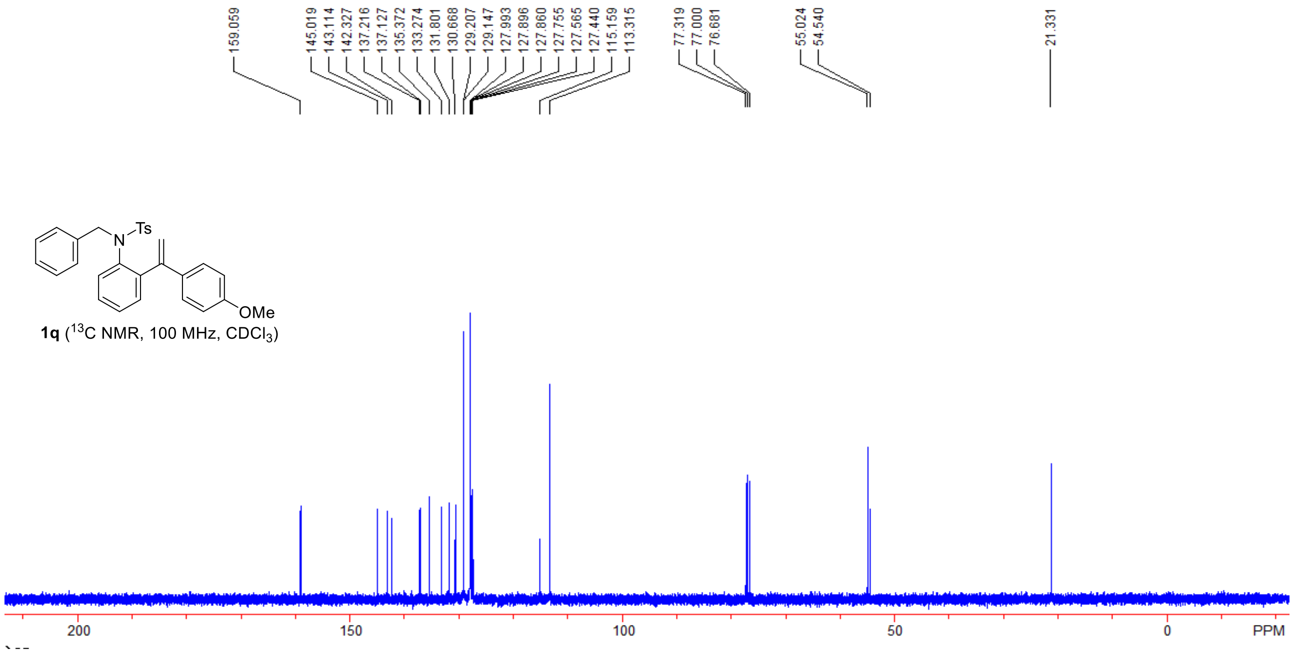

Ts
N
OMe
1q (^{13}C NMR, 100 MHz, $CDCl_3$)
PPM

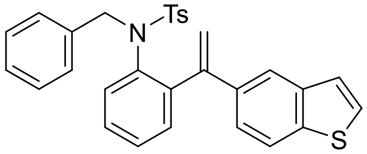

Compound 1r: Yield: 1632 mg, 80%; a yellow oil; Eluent: PE/EA = 5/1. ^{1}H NMR (400 MHz, $CDCl_3$, TMS) δ 2.31 (s, 3H), 4.10-4.60 (m, 2H), 5.21 (s, 1H), 5.71 (s, 1H), 6.84 (d, *J* = 6.4 Hz, 2H), 6.94-7.04 (m, 4H), 7.08 (d, *J* = 8.0 Hz, 2H), 7.13-7.25 (m, 5H), 7.31 (d, *J* = 5.6 Hz, 1H), 7.45 (t, *J* = 8.0 Hz, 3H), 7.70 (d, *J* = 8.0 Hz, 1H); ^{13}C{^{1}H}-NMR (101 MHz, $CDCl_3$, TMS) δ_C 21.3, 54.6, 117.1, 121.8, 122.0, 123.1, 123.9, 126.6, 127.4, 127.72, 127.76, 127.79, 127.9, 129.07, 129.14, 130.5, 131.9, 135.1, 136.9, 137.1, 137.2, 138.8, 139.5, 142.2, 143.1, 145.7; IR (neat): ν 3062, 3029, 2924, 2864, 1596, 1486, 1339, 1184, 1155, 1045, 1027, 832, 754, 696 cm^{-1}; HRMS (ESI-TOF) Calcd for $C_{30}H_{25}NO_2NaS_2$ $[M+Na]^+$: 518.1219, found: 518.1214.

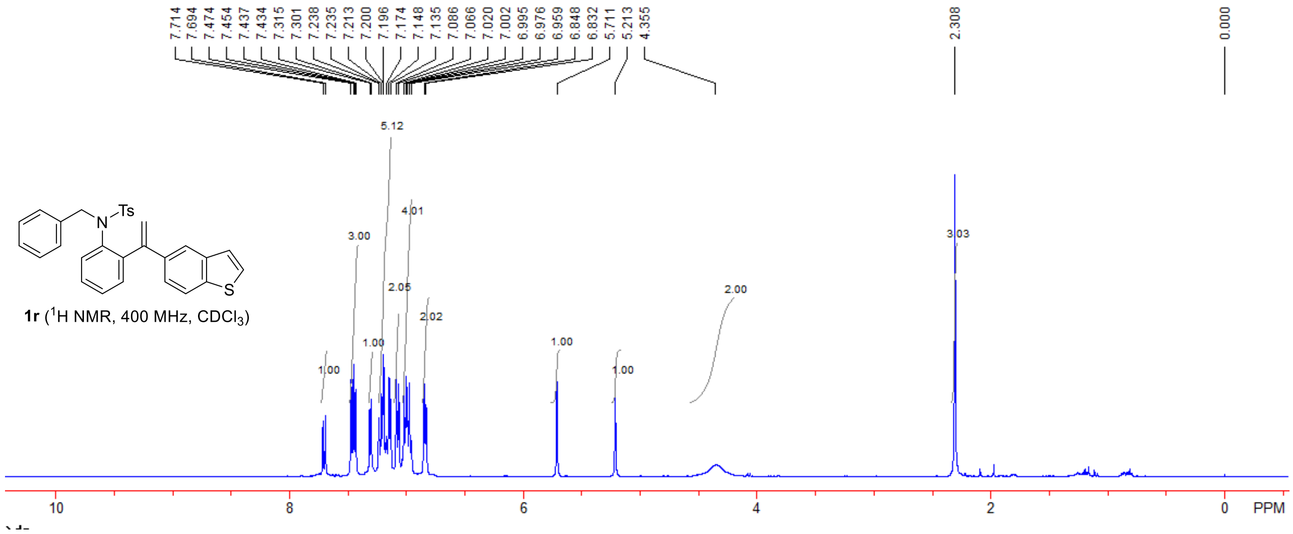

Ts
N
S
1r (1H NMR, 400 MHz, CDCl3)

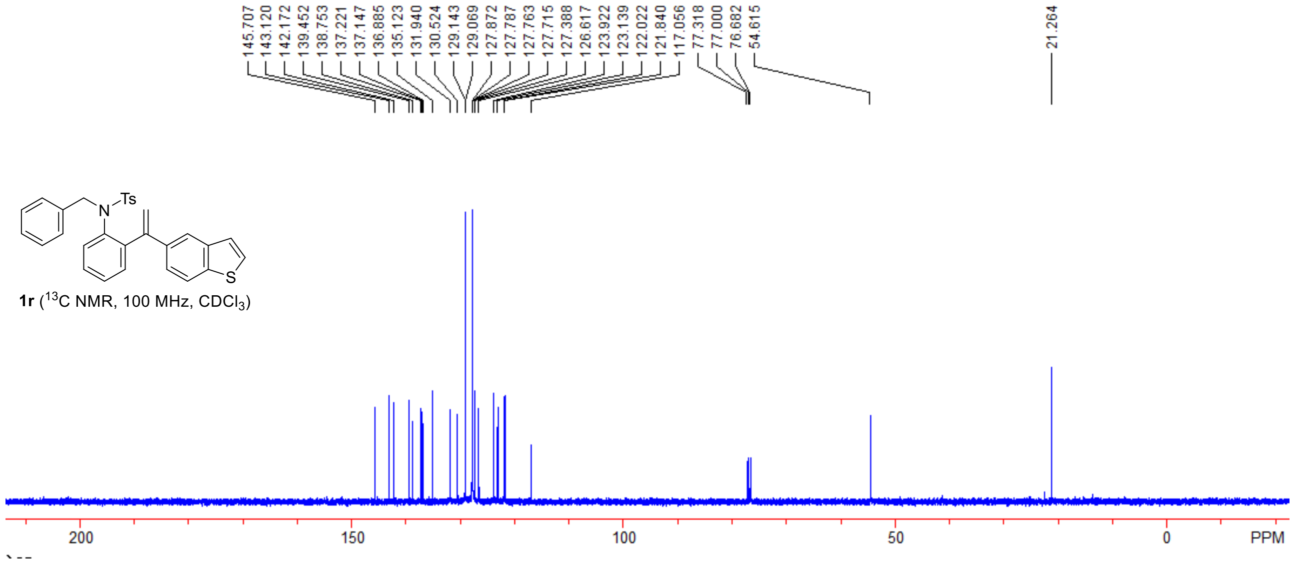

Ts
N
S
1r (13C NMR, 100 MHz, CDCl3)

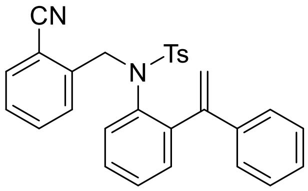

Compound 1s: Yield: 1030 mg, 74%; a light yellow solid; Mp: 107-109 ^{o}C; Eluent: PE/EA = 10/1. ^{1}H NMR (400 MHz, $CDCl_3$, TMS) δ 2.40 (s, 3H), 4.62 (s, 2H), 5.20 (s, 1H), 5.67 (s, 1H), 7.02-7.09 (m, 3H), 7.10-7.31 (m, 10H), 7.35 (t, *J* = 7.6 Hz, 2H), 7.48 (t, *J* = 8.0 Hz, 2H); ^{13}C{^{1}H}-NMR (101 MHz, $CDCl_3$, TMS) δ_C 21.4, 53.3, 111.9, 116.9, 117.4, 126.5, 127.3, 127.9, 128.0, 128.1, 128.3, 129.3, 129.5, 130.7, 132.1, 132.3, 135.3, 137.6, 139.0, 140.3, 142.0, 143.8, 145.4; IR (neat): ν 3061, 1596, 1488, 1445, 1345, 1184, 1156, 1051, 911, 868, 779, 709 cm^{-1}; HRMS (ESI-TOF) Calcd for $C_{29}H_{24}N_2O_2NaS[M+Na]^+$: 487.1451, found: 487.1452.

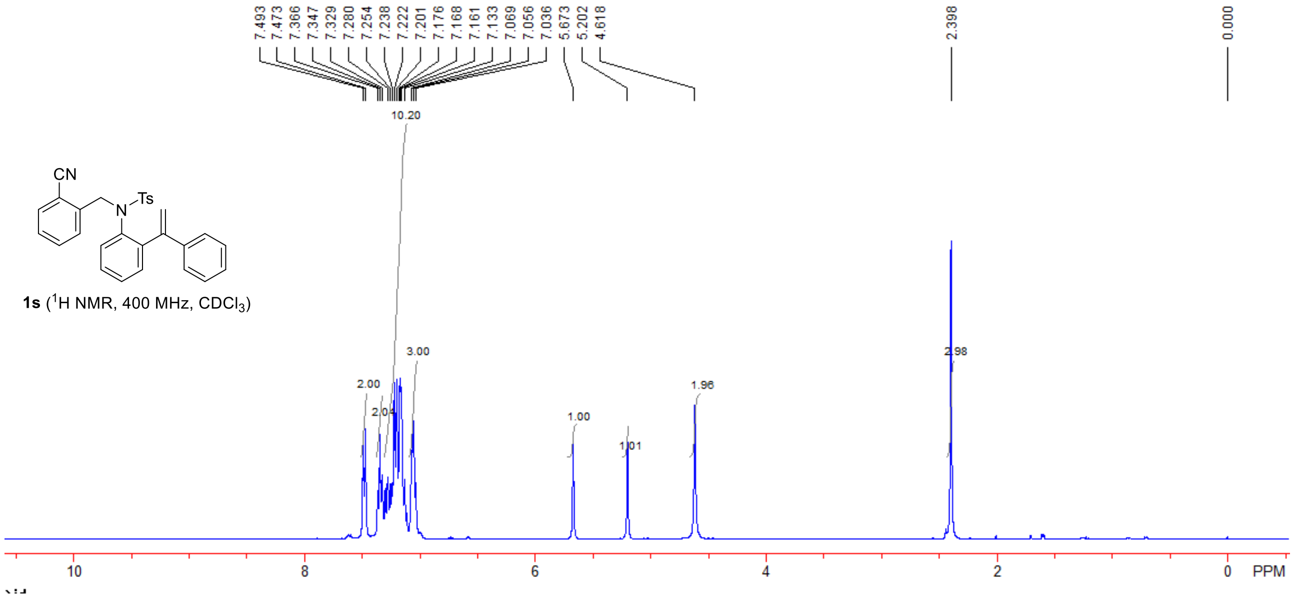

1s (^{1}H NMR, 400 MHz, $CDCl_3$)

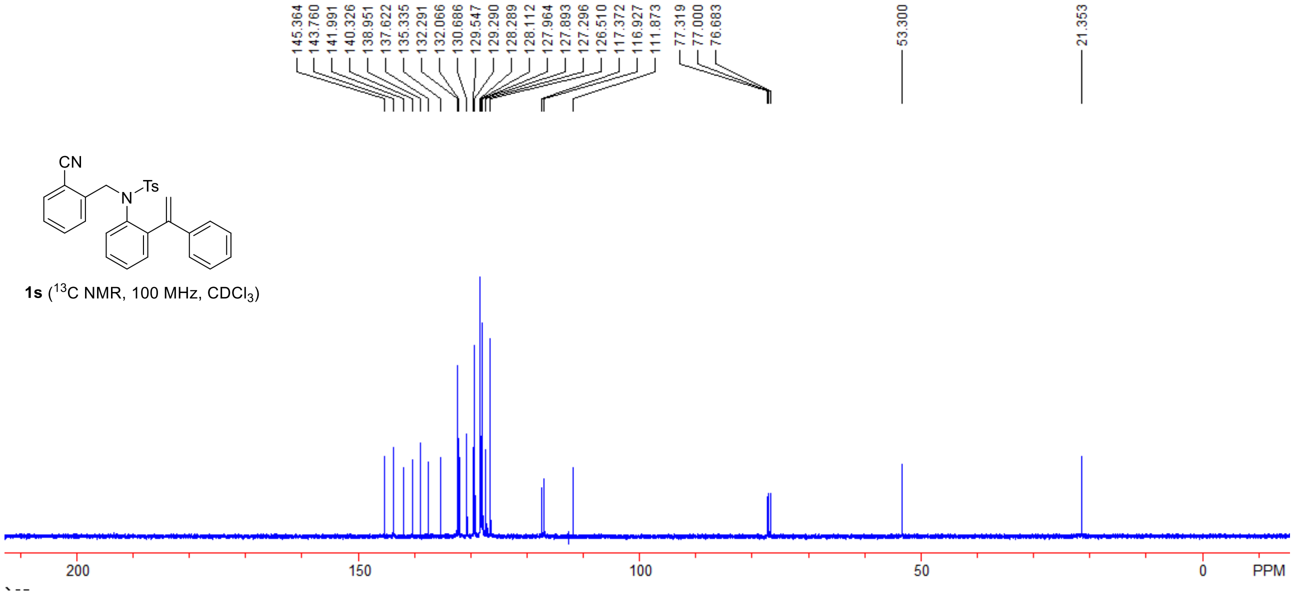

1s (^{13}C NMR, 100 MHz, $CDCl_3$)

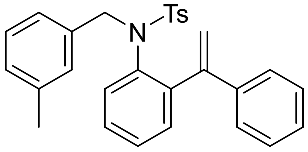

Compound 1t: Yield: 1237 mg, 91%; a yellow oil; Eluent: PE/EA = 10/1. ^{1}H NMR (400 MHz, $CDCl_3$, TMS) δ 2.07 (s, 3H), 2.36 (s, 3H), 4.00-4.60 (m, 2H), 5.20 (d, *J* = 0.8 Hz, 1H), 5.68 (d, *J* = 0.8 Hz, 1H), 6.58-6.64 (m, 2H), 6.85-6.96 (m, 3H), 7.10-7.25 (m, 10H), 7.52 (d, *J* = 8.0 Hz, 2H); ^{13}C{^{1}H}-NMR (101 MHz, $CDCl_3$, TMS) δ_C 20.9, 21.3, 54.7, 117.0, 126.2, 126.9, 127.4, 127.67, 127.68, 127.8, 127.97, 128.0, 128.2, 129.1, 130.0, 130.5, 131.9, 135.1, 137.39, 137.42, 137.44, 140.7, 142.3, 143.1, 145.7; IR (neat): ν 3025, 2919, 1596, 1486, 1379, 1184, 1089, 860, 765, 702 cm^{-1}; HRMS (ESI-TOF) Calcd for $C_{29}H_{27}NO_2NaS$ $[M+Na]^+$: 476.1655, found: 476.1661.

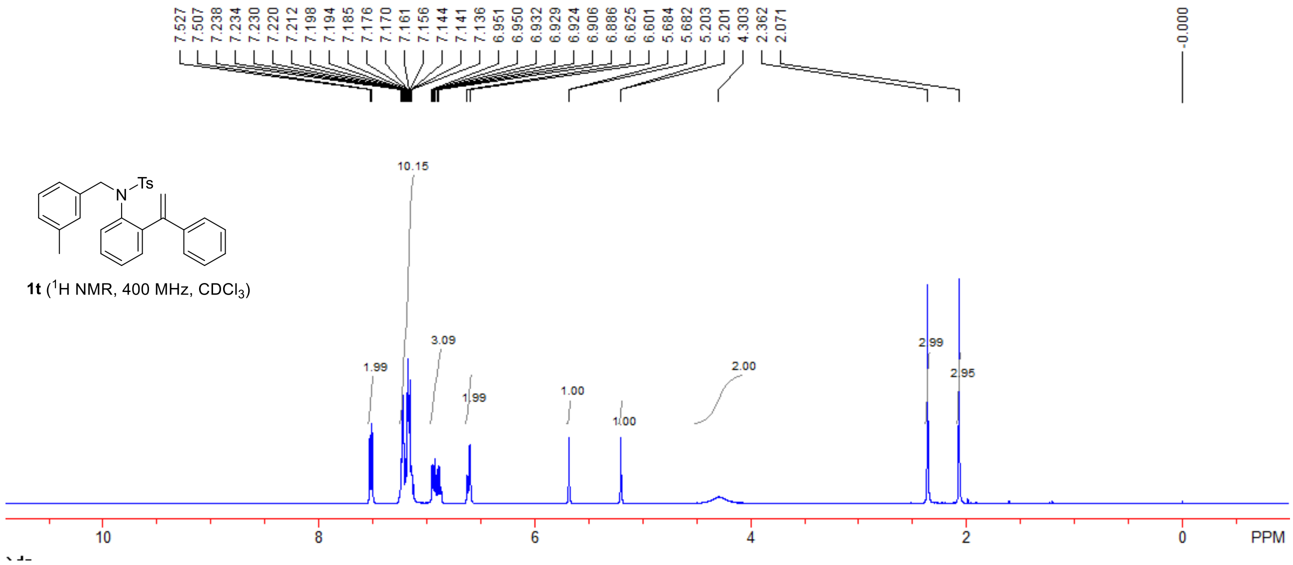
7.527 7.507 7.238 7.234 7.230 7.220 7.212 7.198 7.194 7.185 7.176 7.170 7.161 7.156 7.144 7.141 7.136 6.951 6.950 6.932 6.929 6.924 6.906 6.886 6.625 6.601 5.684 5.682 5.203 5.201 4.303 2.362 2.071
-0.000
Ts
N
1t (^{1}H NMR, 400 MHz, CDCl$_3$)
10.15
1.99
3.09
1.99
1.00
1.00
2.00
2.99
2.95
10
8
6
4
2
0
PPM

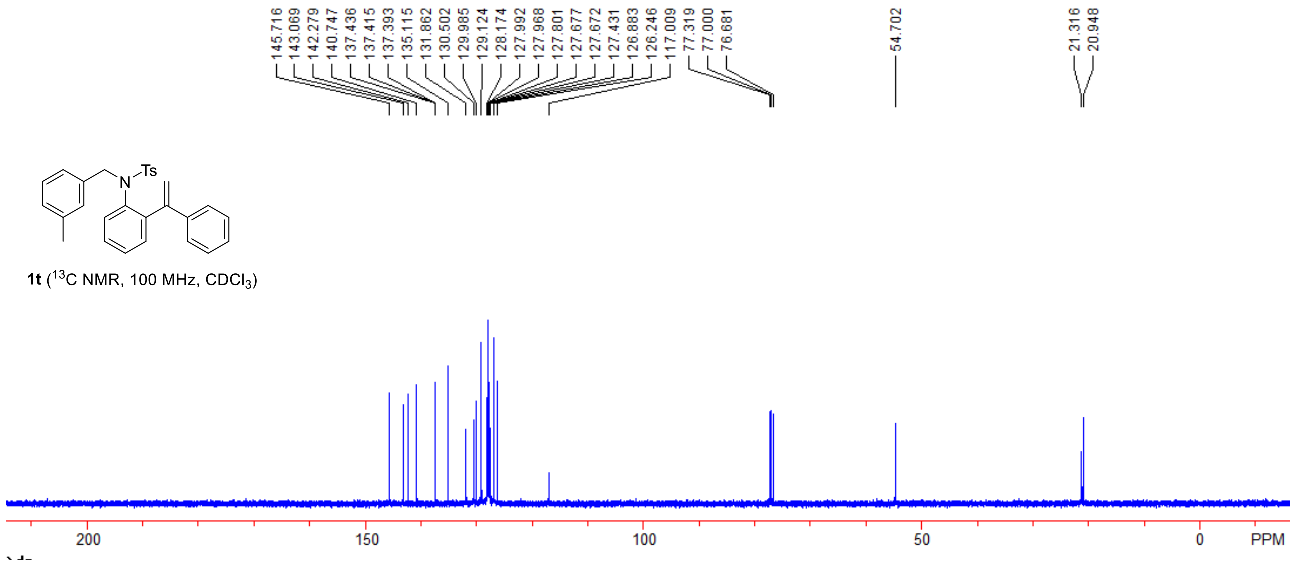
145.716 143.069 142.279 140.747 137.436 137.415 137.393 135.115 131.862 130.502 129.985 129.124 128.174 127.992 127.968 127.801 127.677 127.672 127.431 126.883 126.246 117.009 77.319 77.000 76.681
54.702
21.316 20.948
Ts
N
1t (^{13}C NMR, 100 MHz, CDCl$_3$)
200
150
100
50
0
PPM

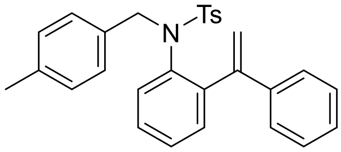

Compound 1u: Yield: 1210 mg, 89%; a yellow oil; Eluent: PE/EA = 10/1. ^{1}H NMR (400 MHz, $CDCl_3$, TMS) δ 2.17 (s, 3H), 2.36 (s, 3H), 4.00-4.60 (m, 2H), 5.17 (s, 1H), 5.65 (s, 1H), 6.72 (d, *J* = 6.4 Hz, 2H), 6.83 (d, *J* = 6.4 Hz, 2H), 6.93 (d, *J* = 7.6 Hz, 1H), 7.08-7.25 (m, 10H), 7.52 (t, *J* = 8.0 Hz, 2H); ^{13}C{^{1}H}-NMR (101 MHz, $CDCl_3$, TMS) δ_C 20.9, 21.3, 54.4, 117.0, 126.9, 127.3, 127.6, 127.7, 128.0, 128.5, 129.1, 129.2, 130.6, 131.8, 132.1, 137.1, 137.2, 140.8, 142.3, 143.1, 145.7; IR (neat): ν 3051, 2920, 2862, 1596, 1486, 1341, 1155, 1089, 865, 781, 705 cm^{-1}; HRMS (ESI-TOF) Calcd for $C_{29}H_{27}NO_2NaS$ [M+Na]$^+$: 476.1655, found: 476.1656.

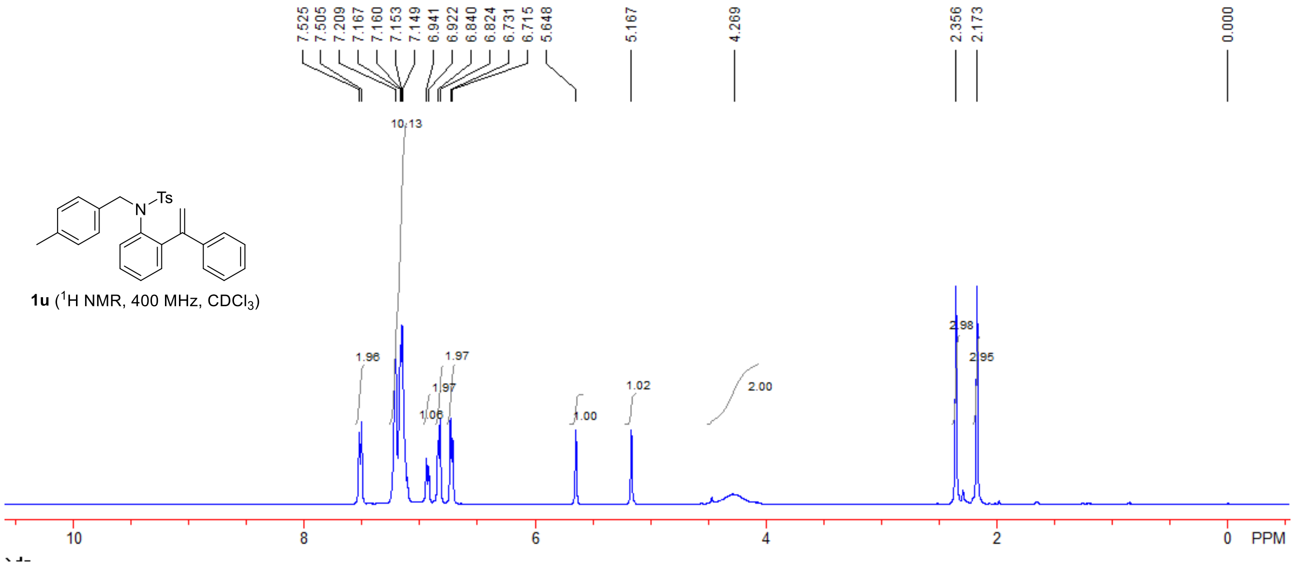

1u (^{1}H NMR, 400 MHz, $CDCl_3$)

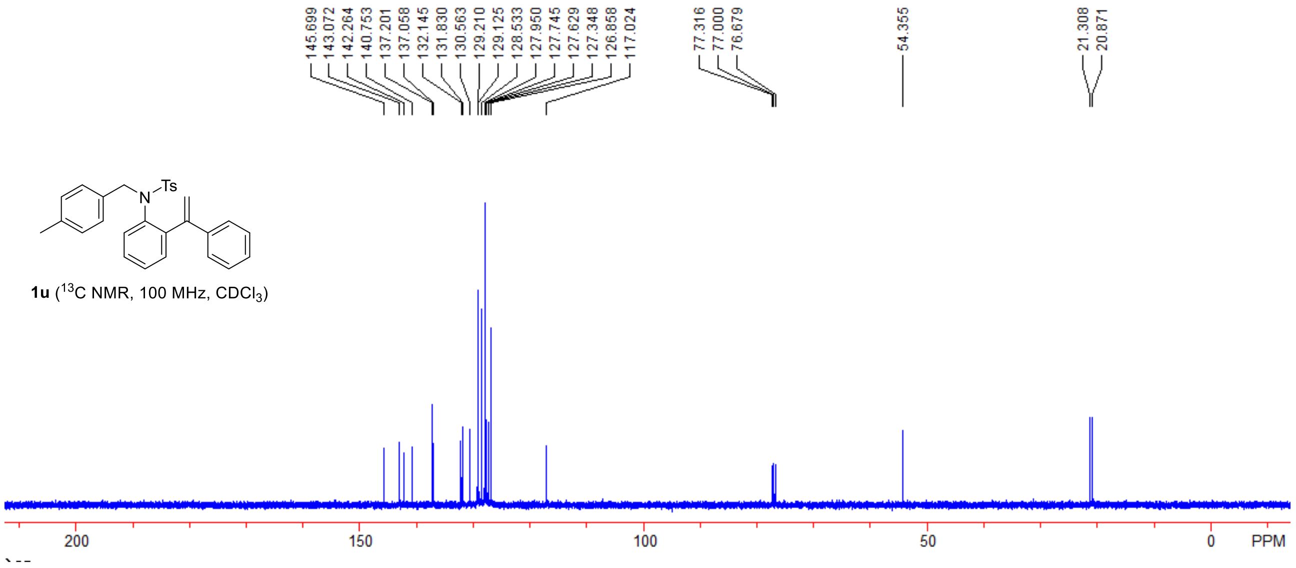

1u (^{13}C NMR, 100 MHz, $CDCl_3$)

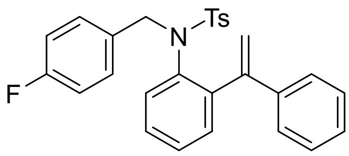

Compound 1v: Yield: 1234 mg, 90%; a yellow oil; Eluent: PE/EA = 10/1. ^{1}H NMR (400 MHz, $CDCl_3$, TMS) δ 2.38 (s, 3H), 4.00-4.60 (m, 2H), 5.14 (s, 1H), 5.65 (s, 1H), 6.71 (t, *J* = 8.8 Hz, 2H), 6.78-6.84 (m, 2H), 6.93 (d, *J* = 8.0 Hz, 1H), 7.12-7.27 (m, 10H), 7.50 (d, *J* = 8.0 Hz, 2H); ^{13}C{^{1}H}-NMR (101 MHz, $CDCl_3$, TMS) δ_C 21.3, 53.9, 114.7 (d, *J* = 21.2 Hz), 117.1, 126.7, 127.5, 127.8, 128.0, 128.1, 129.2, 130.5, 131.0 (d, *J* = 7.9 Hz), 131.1 (d, *J* = 3.3 Hz), 132.0, 136.9, 137.1, 140.6, 142.2, 143.3, 145.8, 162.1 (d, *J* = 244.9 Hz); ^{19}F NMR (376 MHz, $CDCl_3$) δ -114.3; IR (neat): ν 3055, 2922, 1599, 1508, 1444, 1342, 1220, 1089, 851, 814, 764, 705 cm^{-1}; HRMS (ESI-TOF) Calcd for $C_{28}H_{24}NO_2FNaS$ $[M+Na]^+$: 480.1404, found: 480.1413.

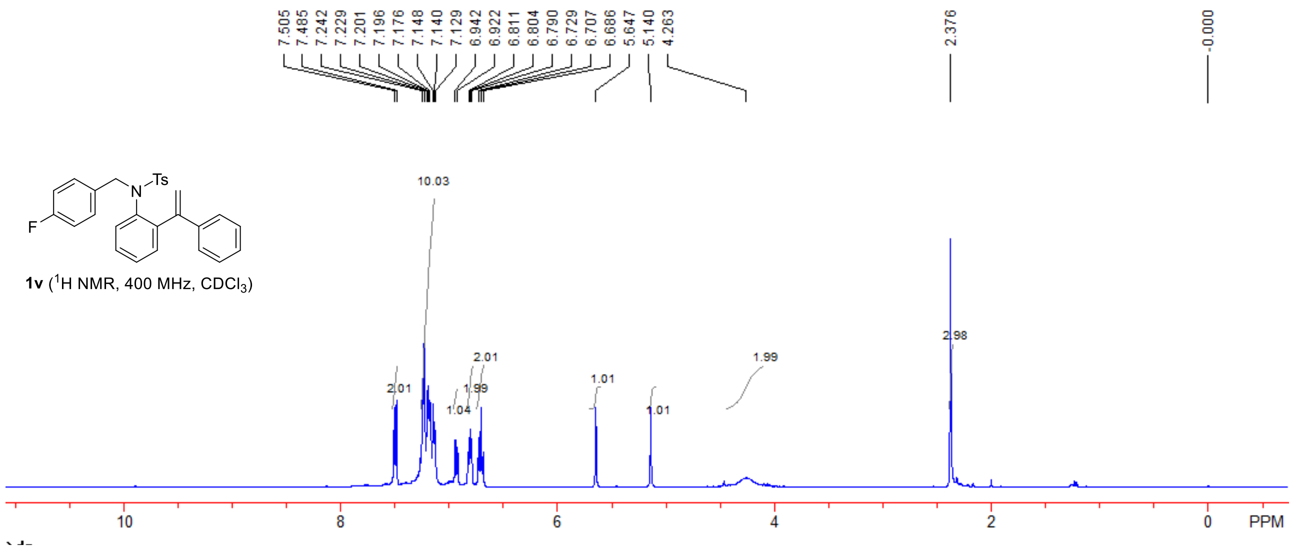

1v (^{1}H NMR, 400 MHz, $CDCl_3$)

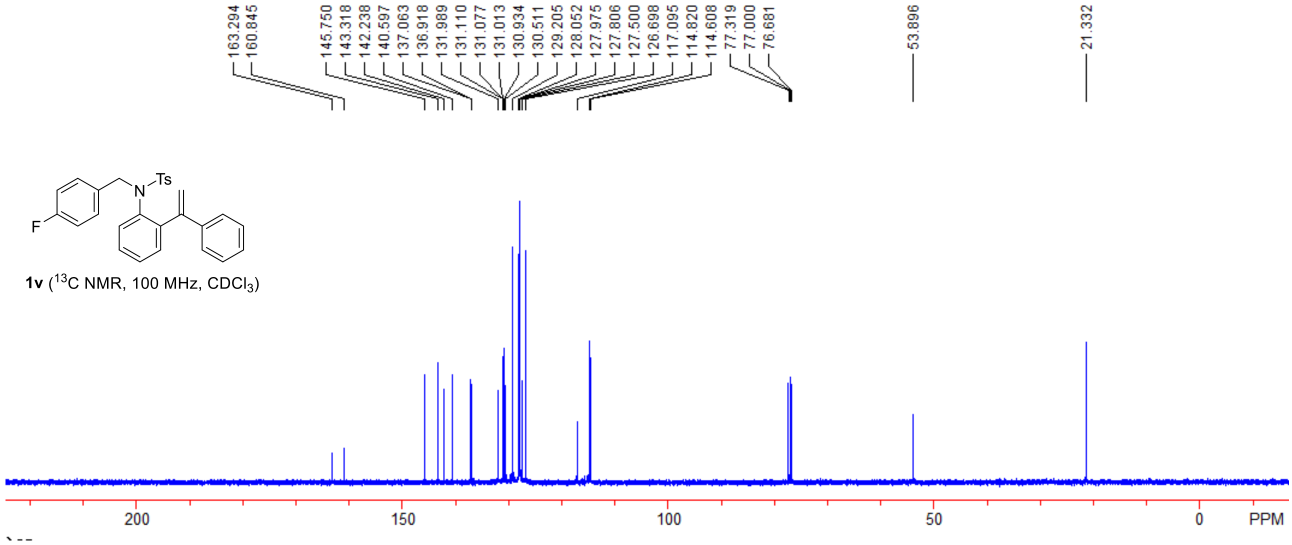

1v (^{13}C NMR, 100 MHz, $CDCl_3$)

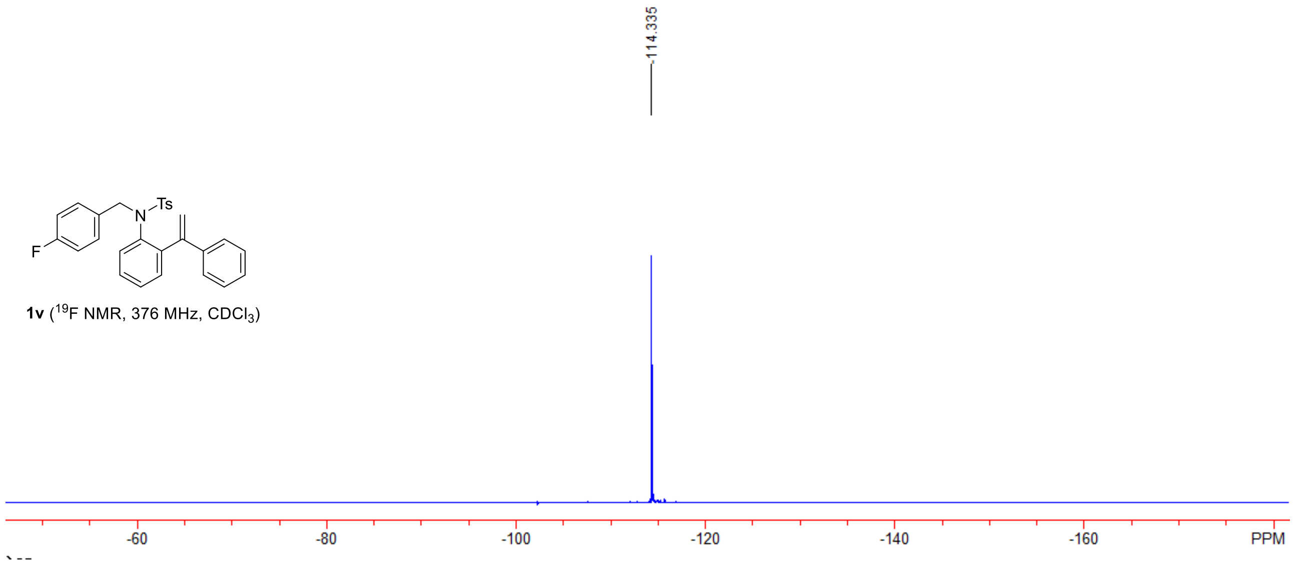

1v (^{19}F NMR, 376 MHz, $CDCl_3$)

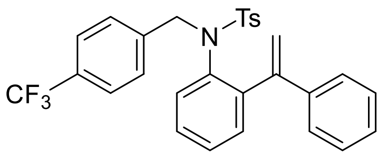

Compound 1w: Yield: 1126 mg, 74%; a yellow oil; Eluent: PE/EA = 10/1. ^{1}H NMR (400 MHz, $CDCl_3$, TMS) δ 2.39 (s, 3H), 4.30-4.45 (m, 2H), 5.18 (s, 1H), 5.70 (s, 1H), 6.95 (d, *J* = 8.0 Hz, 2H), 7.03 (d, *J* = 8.4 Hz, 1H), 7.10-7.33 (m, 12H), 7.48 (d, *J* = 8.0 Hz, 2H); ^{13}C{^{1}H}-NMR (101 MHz, $CDCl_3$, TMS) δ_C 21.4, 54.5, 117.3, 123.9 (q, *J* = 270.6 Hz), 124.8 (q, *J* = 3.7 Hz), 126.6, 127.7, 128.1, 128.2, 129.3, 129.4, 129.6 (q, *J* = 32.5 Hz), 130.3, 132.2, 136.8, 137.5, 139.5, 140.5, 142.1, 143.6, 145.8; ^{19}F NMR (376 MHz, $CDCl_3$) δ -62.5; IR (neat): ν 2923, 1596, 1493, 1419, 1321, 1157, 1113, 1089, 1018, 908, 828, 766, 706 cm^{-1}; HRMS (ESI-TOF) Calcd for $C_{29}H_{27}NO_2NaS$ $[M+Na]^+$: 476.1655, found: 476.1661.

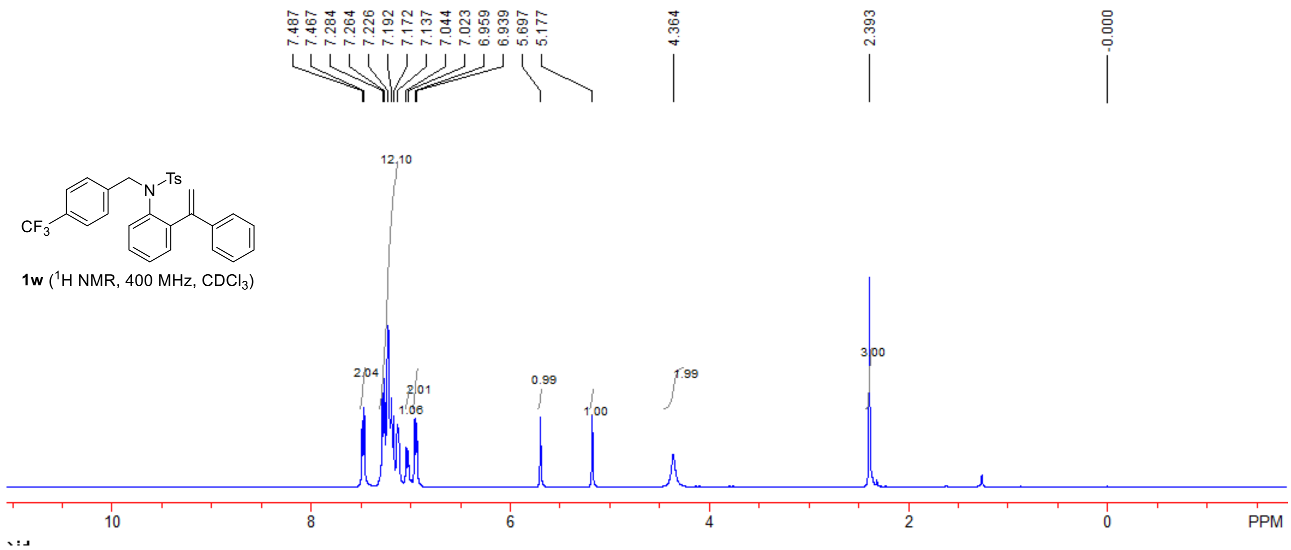
7.487
7.467
7.284
7.264
7.226
7.192
7.172
7.137
7.044
7.023
6.959
6.939
5.697
5.177
4.364
2.393
-0.000
12.10
2.04
2.01
1.06
0.99
1.00
1.99
3.00
CF3
N
Ts
1w (1H NMR, 400 MHz, CDCl3)
10
8
6
4
2
0
PPM

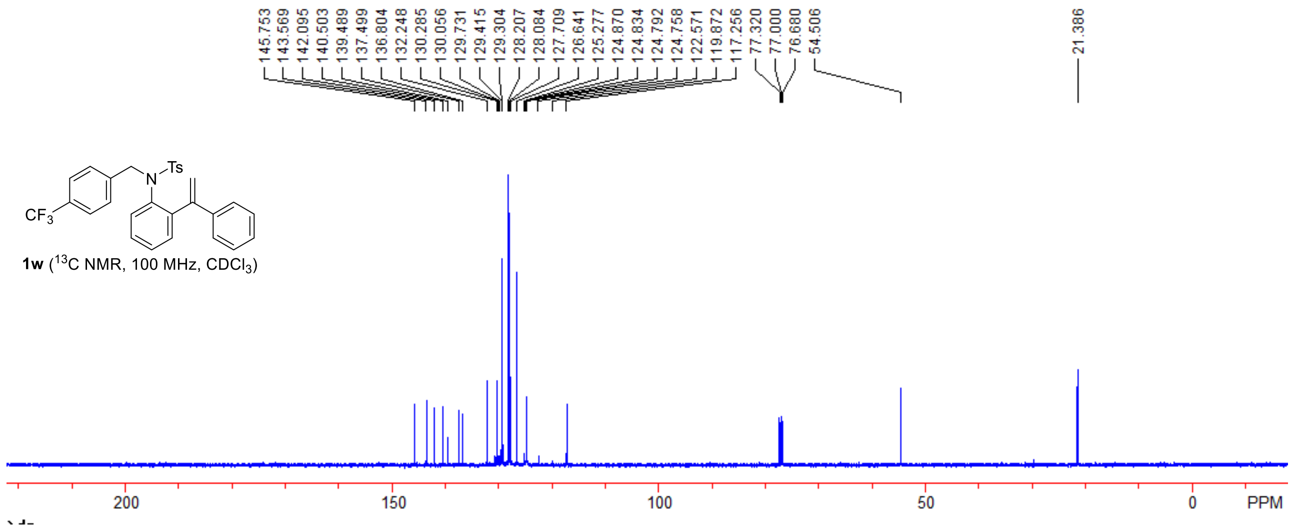
145.753
143.569
142.095
140.503
139.489
137.499
136.804
132.248
130.285
130.056
129.731
129.415
129.304
128.207
128.084
127.709
126.641
125.277
124.870
124.834
124.792
124.758
122.571
119.872
117.256
77.320
77.000
76.680
54.506
21.386
CF3
N
Ts
1w (13C NMR, 100 MHz, CDCl3)
200
150
100
50
0
PPM

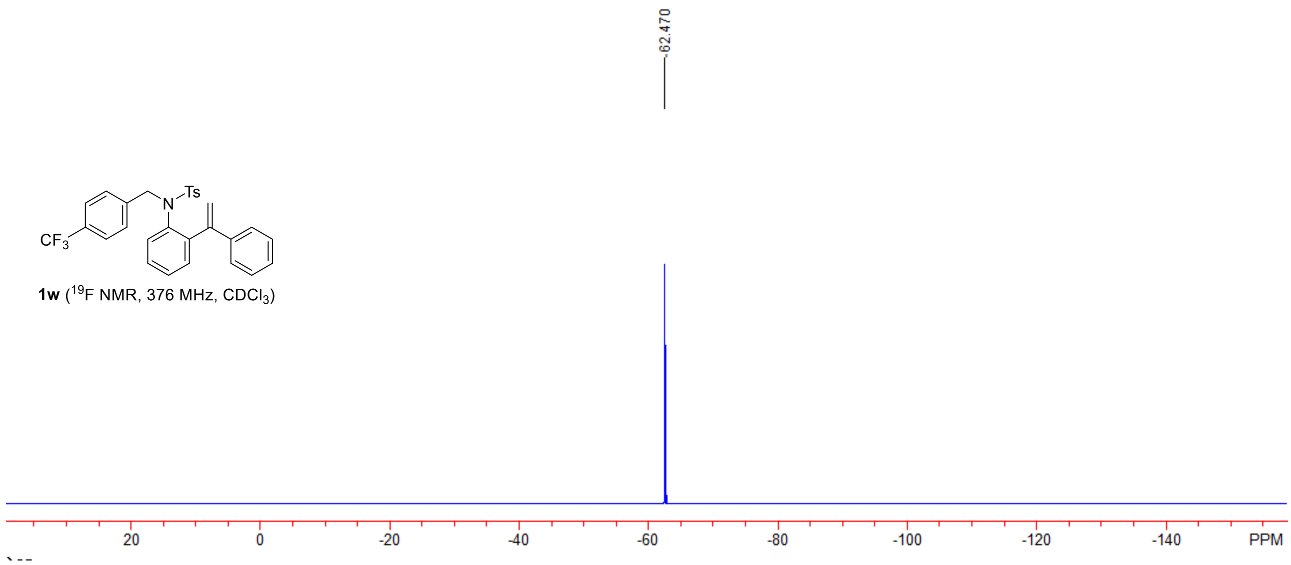
-62.470
CF3
N
Ts
1w (19F NMR, 376 MHz, CDCl3)
20
0
-20
-40
-60
-80
-100
-120
-140
PPM

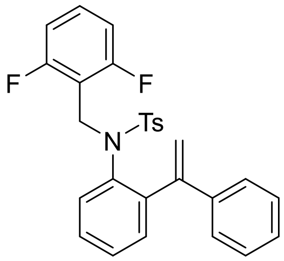

Compound 1x: Yield: 1212 mg, 85%; a light yellow solid; Mp: 118-120 ^{o}C; Eluent: PE/EA = 10/1. ^{1}H NMR (400 MHz, $CDCl_3$, TMS) δ 2.36 (s, 3H), 4.30-4.60 (m, 2H), 5.11 (s, 1H), 5.62 (s, 1H), 6.53-6.63 (m, 2H), 7.00-7.09 (m, 1H), 7.12-7.31 (m, 11H), 7.41 (d, J = 8.0 Hz, 2H); $^{13}C\{^{1}H\}$-NMR (101 MHz, $CDCl_3$, TMS) δ_C 21.3, 42.7, 110.9 (dd, J_1 = 19.0 Hz, J_2 = 6.4 Hz), 111.1 (t, J = 19.2 Hz), 117.1, 126.9, 127.3, 127.86, 127.90, 128.1, 128.4, 129.0, 129.6, 130.1 (t, J = 10.3 Hz), 132.0, 135.9, 137.2, 140.7, 142.5, 143.4, 145.9, 161.7 (dd, J_1 = 250.1 Hz, J_2 =7.2 Hz); ^{19}F NMR (376 MHz, $CDCl_3$) δ -111.9; IR (neat): ν 3051, 2942, 1594, 1487, 1320, 1267, 1196, 1156, 1088, 947, 907, 814, 726 cm^{-1}; HRMS (ESI-TOF) Calcd for $C_{28}H_{23}NO_2F_2NaS$ [M+Na]$^+$: 498.1310, found: 498.1315.

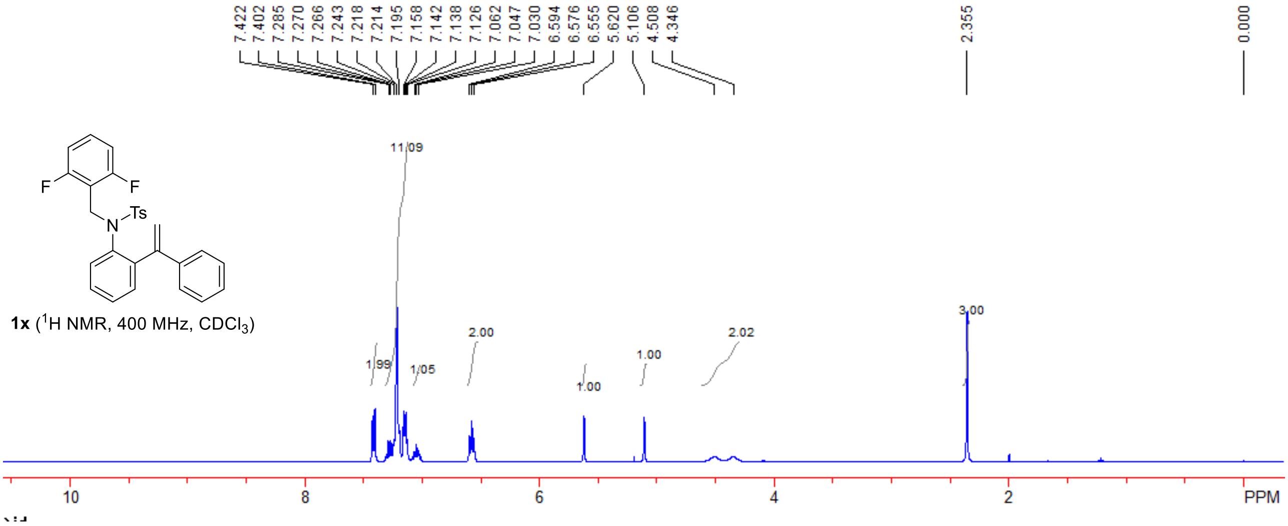

1x (^{1}H NMR, 400 MHz, $CDCl_3$)

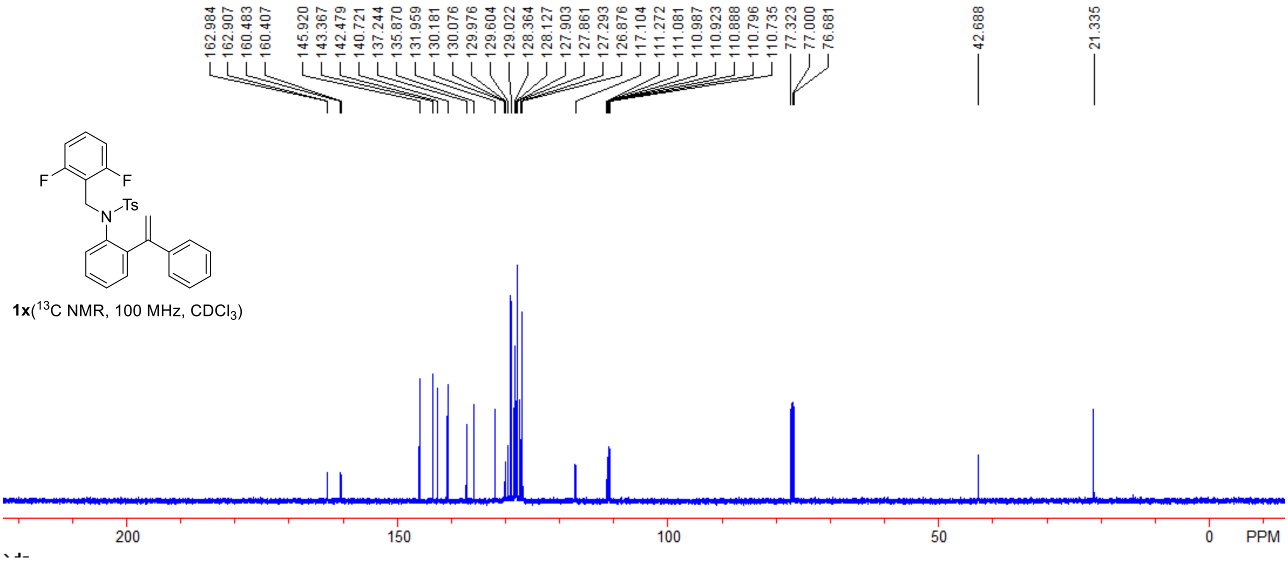

1x(^{13}C NMR, 100 MHz, $CDCl_3$)

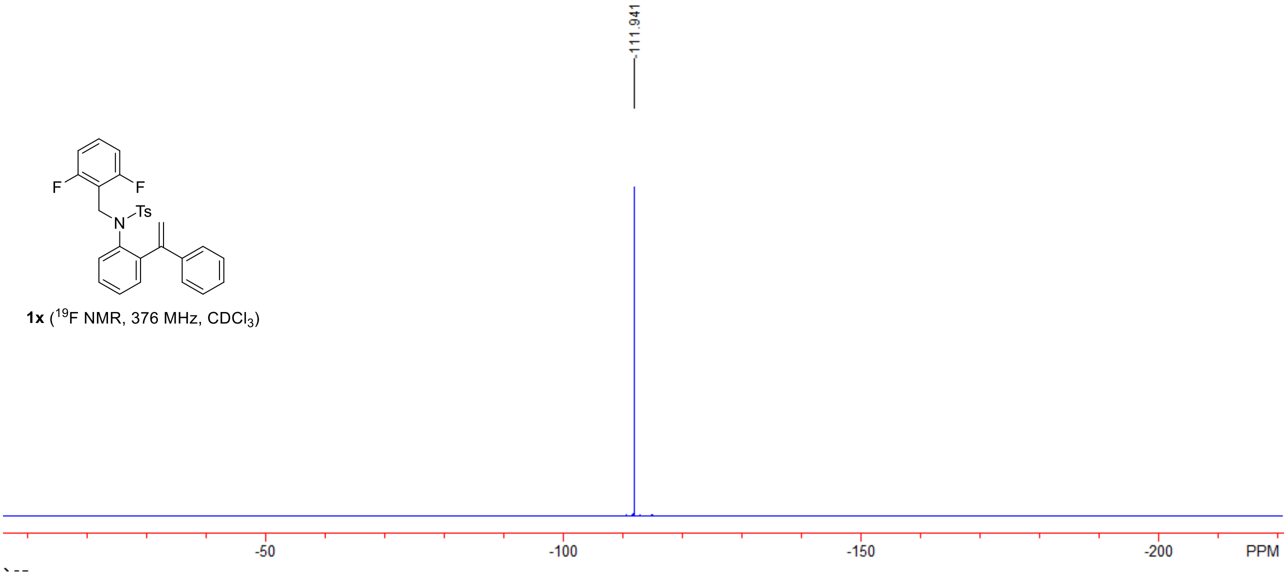

1x (^{19}F NMR, 376 MHz, $CDCl_3$)

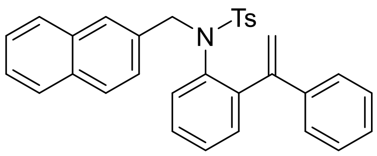

Compound 1y: Yield: 1174 mg, 80%; a light yellow solid; Mp: 123-125 ºC; Eluent: PE/EA = 10/1. ^{1}H NMR (400 MHz, $CDCl_3$, TMS) δ 2.28 (s, 3H), 4.30-4.70 (m, 2H), 5.22 (s, 1H), 5.68 (s, 1H), 7.01 (d, J = 7.6 Hz, 2H), 7.06-7.18 (m, 11H), 7.29-7.34 (m, 2H), 7.44-7.56 (m, 4H), 7.59-7.64 (m, 1H); $^{13}C\{^{1}H\}$-NMR (101 MHz, $CDCl_3$, TMS) δ_C 21.4, 55.0, 117.2, 125.77, 125.80, 126.8, 126.9, 127.4, 127.51, 127.60, 127.66, 127.8, 127.9, 128.01, 128.05, 128.3, 129.2, 130.5, 132.0, 132.6, 132.74, 132.78, 137.3, 137.4, 140.6, 142.2, 143.2, 145.6; IR (neat): ν 3053, 2921, 1596, 1489, 1441, 1361, 1305, 1156, 1037, 928, 816, 765, 705 cm^{-1}; HRMS (ESI-TOF) Calcd for $C_{32}H_{27}NO_2NaS$ $[M+Na]^+$: 512.1655, found: 512.1653.

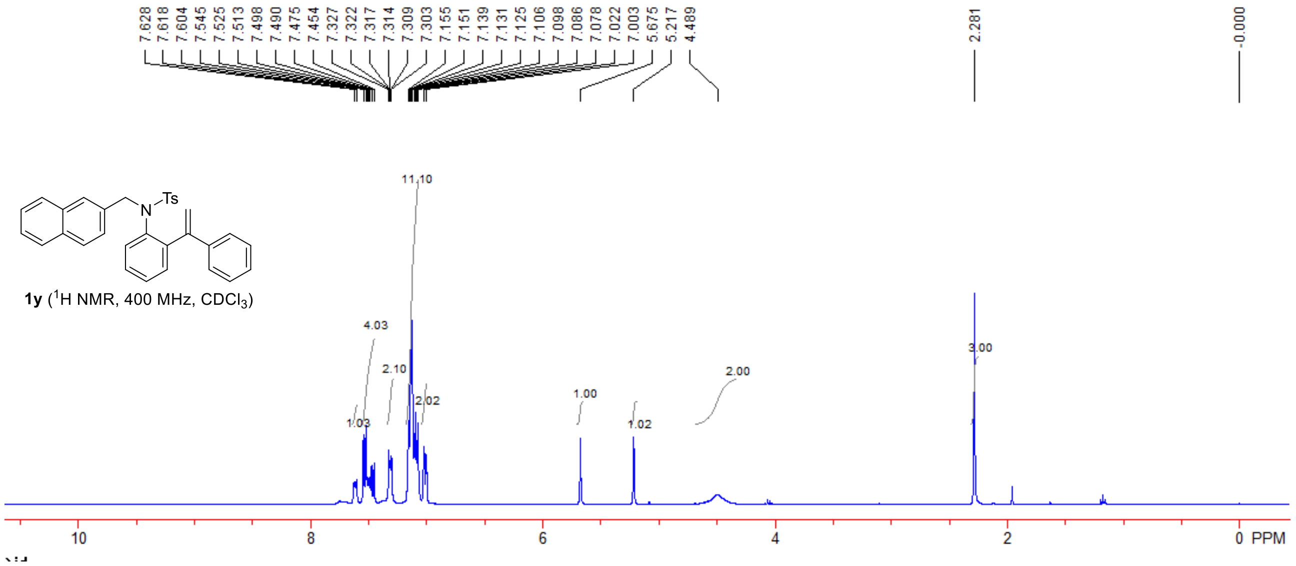

1y (^{1}H NMR, 400 MHz, $CDCl_3$)

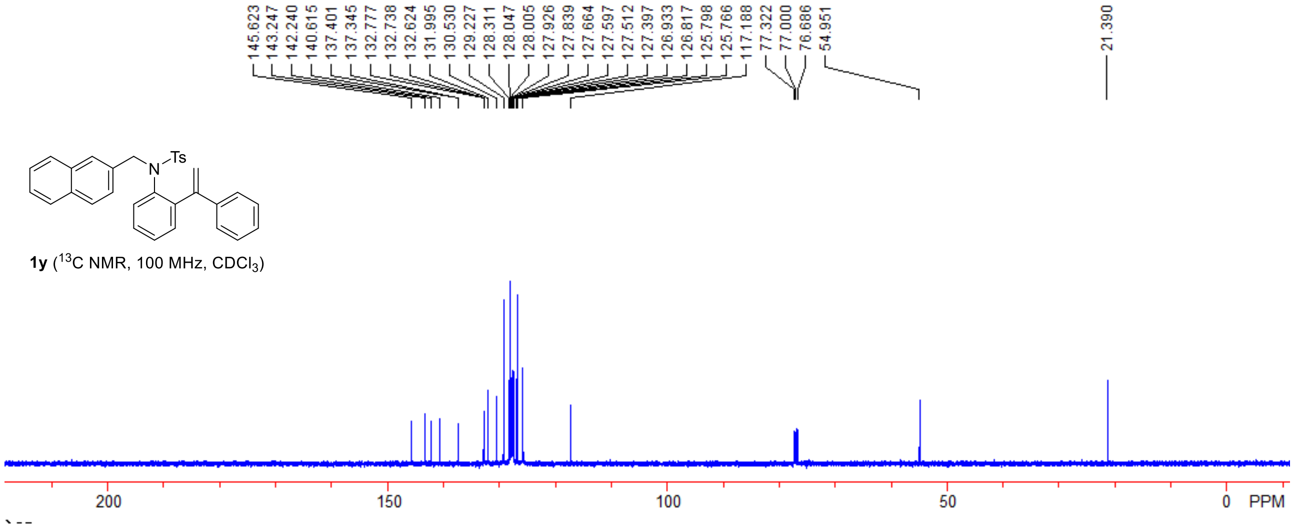

1y (^{13}C NMR, 100 MHz, $CDCl_3$)

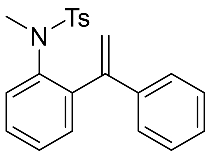

Compound 1z: Yield: 1024 mg, 94%; a light yellow solid; Mp: 124-126 ^{o}C; Eluent: PE/EA = 10/1. ^{1}H NMR (400 MHz, $CDCl_3$, TMS) δ 2.35 (s, 3H), 2.58 (s, 3H), 5.38 (s, 1H), 5.66 (s, 1H), 6.79 (d, *J* = 7.6 Hz, 1H), 7.16-7.32 (m, 9H), 7.37 (d, *J* = 7.6 Hz, 1H), 7.48 (d, *J* = 7.6 Hz, 2H); ^{13}C{^{1}H}-NMR (101 MHz, $CDCl_3$, TMS) δ_C 21.2, 38.1, 116.5, 126.7, 127.1, 127.2, 127.7, 128.0, 128.2, 129.1, 131.3, 134.9, 139.6, 141.3, 142.9, 143.1, 147.4; IR (neat): ν 3062, 3025, 2919, 1596, 1493, 1344, 1270, 1108, 1029, 900, 774, 708 cm^{-1}; HRMS (ESI-TOF) Calcd for $C_{23}H_{23}NO_2NaS$ $[M+Na]^+$: 400.1342, found: 400.1340.

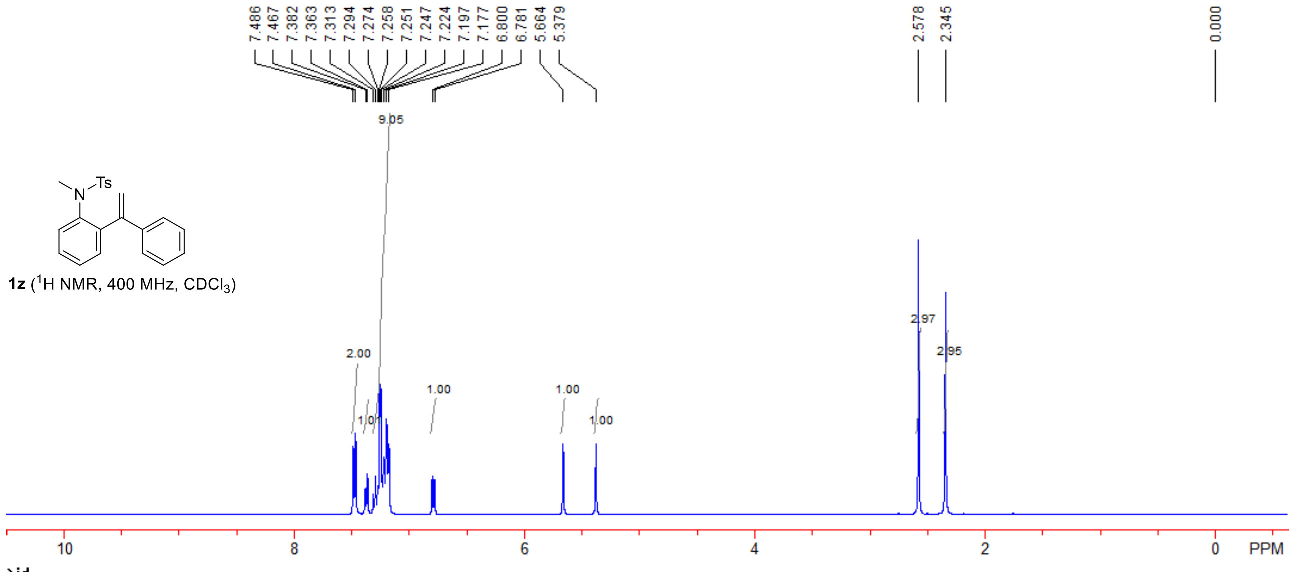

1z (^{1}H NMR, 400 MHz, $CDCl_3$)

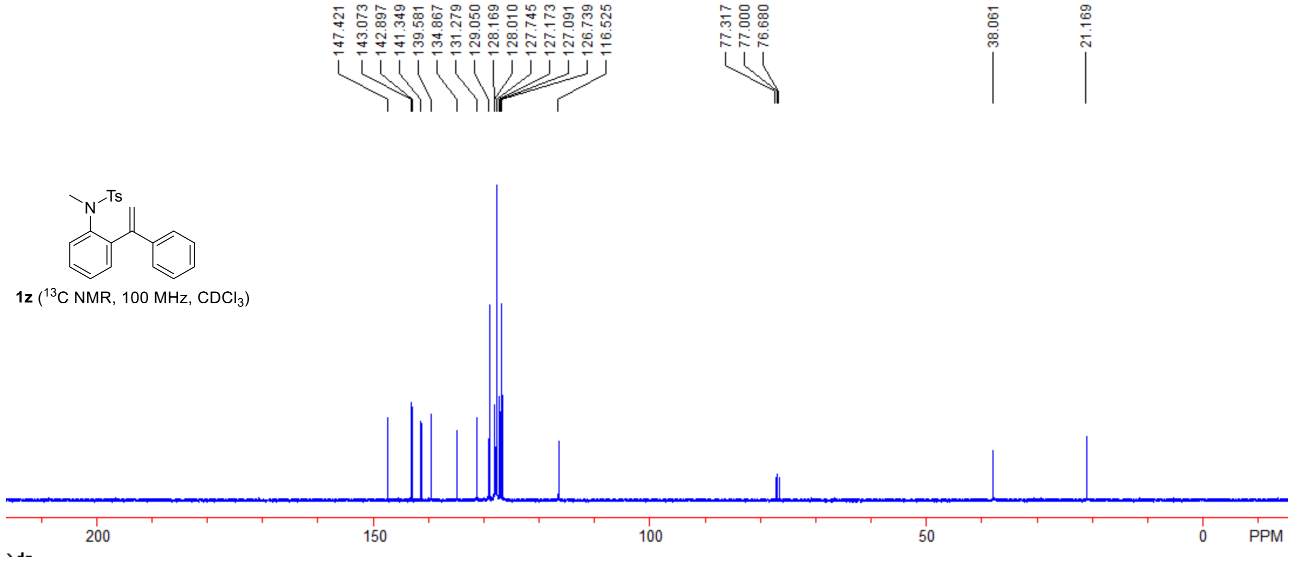

1z (^{13}C NMR, 100 MHz, $CDCl_3$)

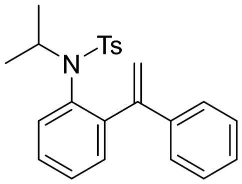

Compound 1aa: Yield: 904 mg, 77%; a light yellow solid; Mp: 119-121 ^{o}C; Eluent: PE/EA = 10/1. ^{1}H NMR (400 MHz, $CDCl_3$, TMS) δ 0.83 (d, *J* = 6.0 Hz, 3H), 0.94 (d, *J* = 5.6 Hz, 3H), 2.40 (s, 3H), 3.95-4.05 (m, 1H), 5.71 (s, 1H), 5.86 (s, 1H), 7.13 (d, *J* = 7.6 Hz, 1H), 7.20-7.34 (m, 10H), 7.56 (d, *J* = 8.0 Hz, 2H); $^{13}C\{^{1}H\}$-NMR (101 MHz, $CDCl_3$, TMS) δ_C 20.7, 21.4, 22.6, 53.4, 118.2, 127.2, 127.4, 127.5, 128.0, 128.1, 129.3, 131.8, 132.6, 135.3, 138.0, 141.2, 143.1, 143.5, 145.9; IR (neat): ν 3023, 2986, 2970, 2934, 1595, 1485, 1386, 1333, 1269, 1180, 1125, 1025, 922, 866, 767, 706 cm^{-1}; HRMS (ESI-TOF) Calcd for $C_{24}H_{25}NO_2NaS$ $[M+Na]^+$: 414.1498, found: 414.1501.

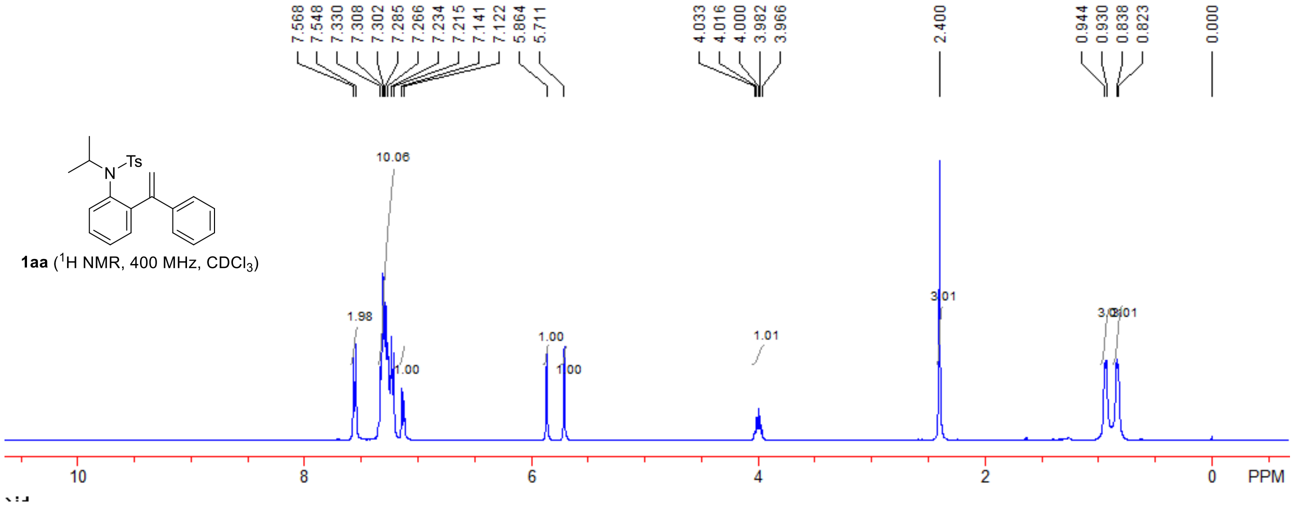

1aa (^{1}H NMR, 400 MHz, $CDCl_3$)

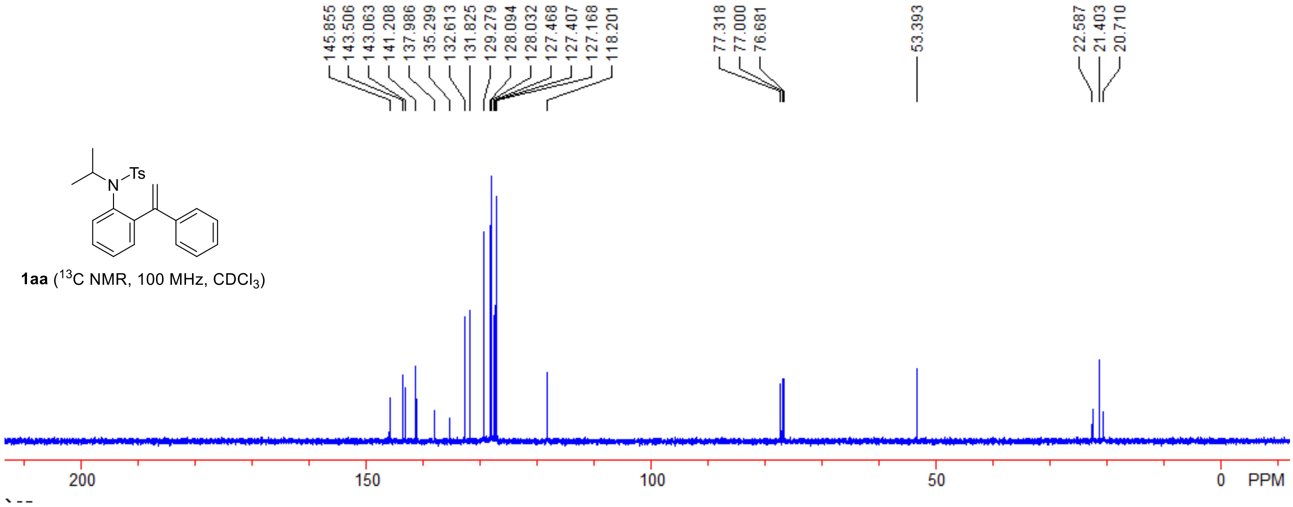

1aa (^{13}C NMR, 100 MHz, $CDCl_3$)

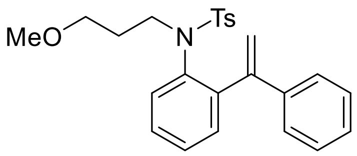

Compound 1ab: Yield: 1150 mg, 91%; a yellow oil; Eluent: PE/EA = 10/1. ^{1}H NMR (400 MHz, $CDCl_3$, TMS) δ 1.28-1.38 (m, 2H), 2.36 (s, 3H), 3.00 (t, *J* = 5.6 Hz, 2H), 3.07 (s, 3H), 3.26 (t, *J* = 8.0 Hz, 2H), 5.40 (s, 1H), 5.74 (s, 1H), 7.02 (d, *J* = 7.6 Hz, 1H), 7.17-7.32 (m, 10H), 7.52 (d, *J* = 8.0 Hz, 2H); $^{13}C\{^{1}H\}$-NMR (101 MHz, $CDCl_3$, TMS) δ_C 21.2, 27.6, 48.4, 58.0, 69.5, 117.2, 126.8, 127.3, 127.7, 127.85, 127.88, 127.91, 129.0, 129.1, 131.9, 136.2, 137.7, 140.9, 142.4, 143.0, 146.3; IR (neat): ν 3056, 2924, 2871, 1596, 1486, 1444, 1385, 1186, 1110, 910, 781, 706 cm^{-1}; HRMS (ESI-TOF) Calcd for $C_{25}H_{27}NO_3NaS$ $[M+Na]^+$: 444.1604, found: 444.1610.

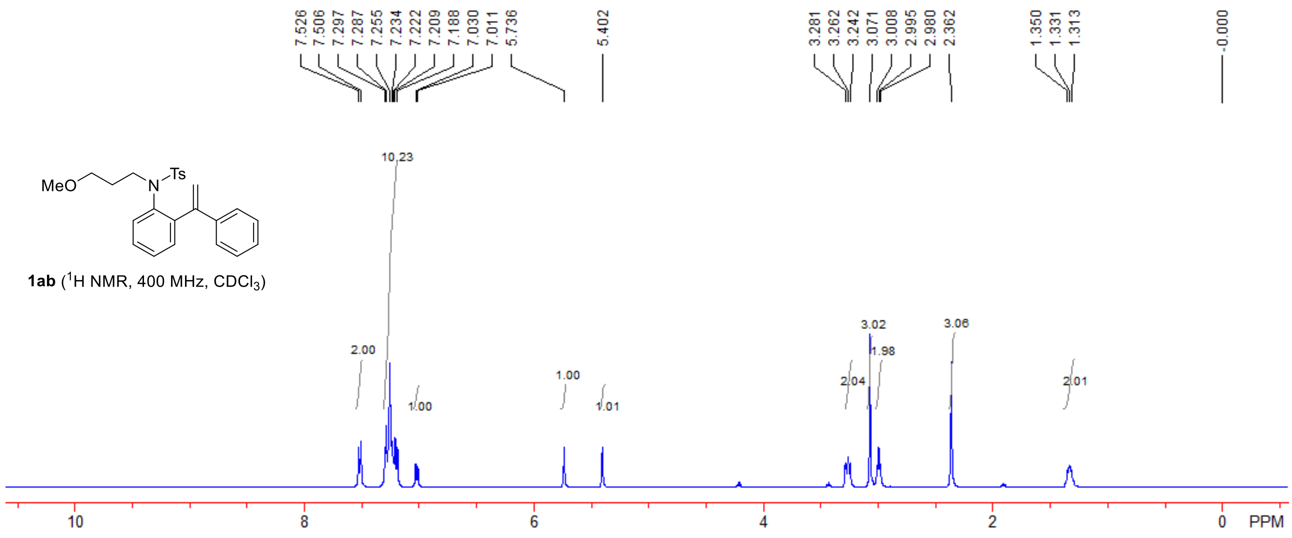

1ab (^{1}H NMR, 400 MHz, $CDCl_3$)

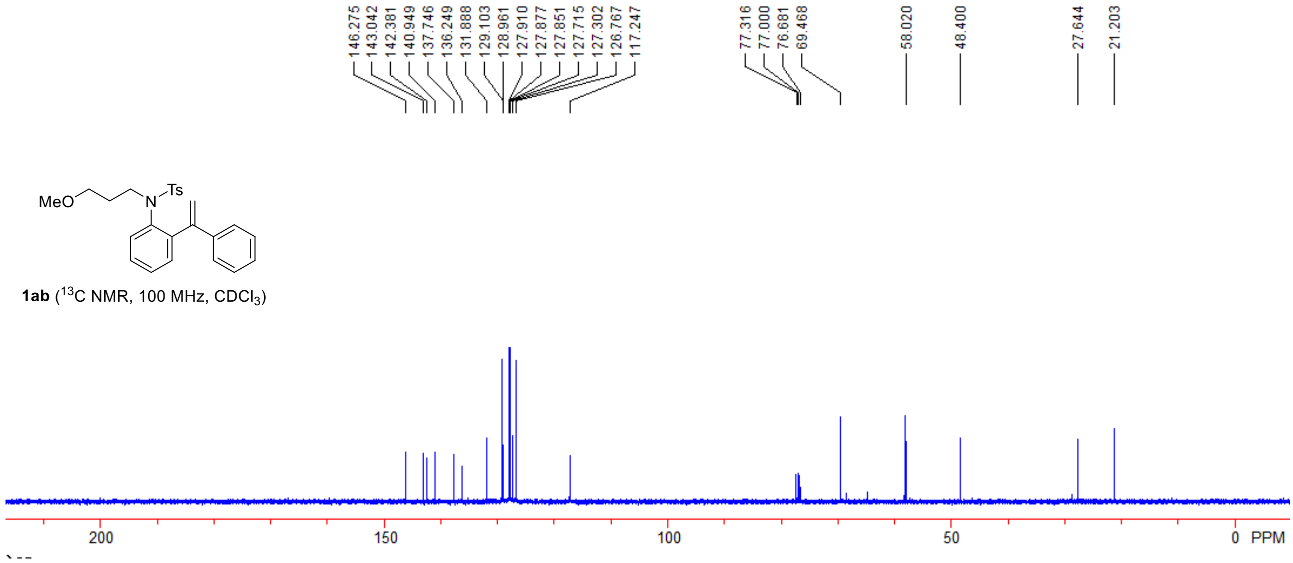

1ab (^{13}C NMR, 100 MHz, $CDCl_3$)

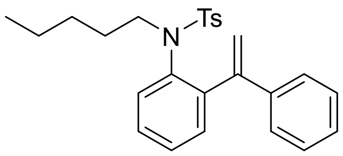

Compound 1ac: Yield: 1157 mg, 92%; a light yellow solid; Mp: 84-86 °C; Eluent: PE/EA = 10/1. ^{1}H NMR (400 MHz, $CDCl_3$, TMS) δ 0.70 (t, *J* = 7.2 Hz, 3H), 0.83-0.92 (m, 2H), 0.96-1.08 (m, 4H), 2.41 (s, 3H), 3.05-3.12 (m, 2H), 5.38 (s, 1H), 5.73 (s, 1H), 7.02 (d, *J* = 7.6 Hz, 1H), 7.21-7.35 (m, 10H), 7.35 (d, *J* = 8.0 Hz, 2H); ^{13}C{^{1}H}-NMR (101 MHz, $CDCl_3$, TMS) δ_C 13.8, 21.5, 22.0, 27.3, 28.8, 51.2, 117.5, 127.0, 127.5, 127.87, 127.96, 128.03, 128.08, 129.3, 132.1, 137.9, 141.2, 142.7, 143.1, 146.5; IR (neat): ν 3320, 3061, 2921, 1597, 1450, 1330, 1264, 1164, 1090, 1027, 873, 784, 703 cm^{-1}; HRMS (ESI-TOF) Calcd for $C_{26}H_{29}NO_2NaS$ [M+Na]$^+$: 442.1811, found: 442.1811.

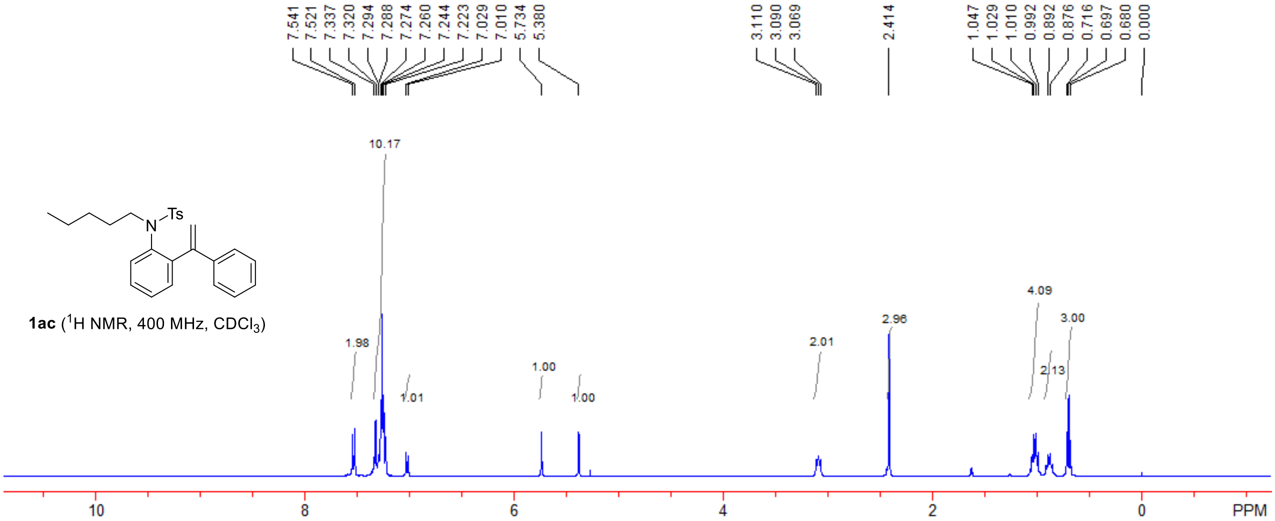

1ac (^{1}H NMR, 400 MHz, $CDCl_3$)

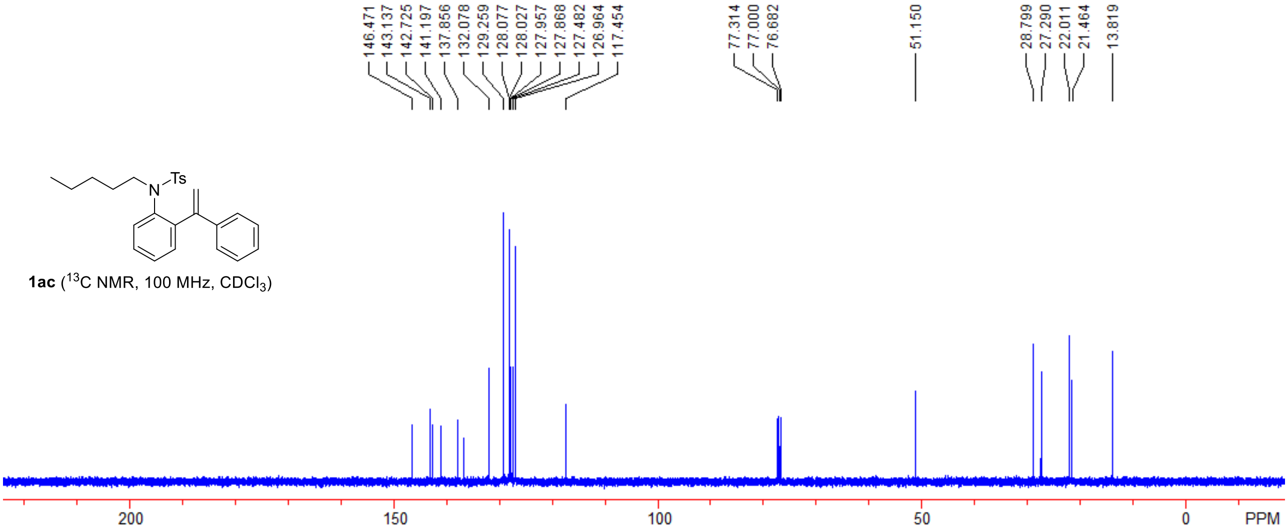

1ac (^{13}C NMR, 100 MHz, $CDCl_3$)

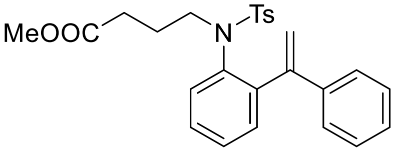

Compound 1ad: Yield: 1199 mg, 89%; a yellow oil; Eluent: PE/EA = 10/1. ^{1}H NMR (400 MHz, $CDCl_3$, TMS) δ 1.34-1.44 (m, 2H), 1.96 (t, *J* = 6.4 Hz, 2H), 2.40 (s, 3H), 3.18 (t, *J* = 6.8 Hz, 2H), 3.54 (s, 3H), 5.38 (s, 1H), 5.75 (s, 1H), 7.03 (d, *J* = 7.6 Hz, 1H), 7.20-7.35 (m, 10H), 7.51 (d, *J* = 8.4 Hz, 2H); $^{13}C\{^{1}H\}$-NMR (101 MHz, $CDCl_3$, TMS) δ_C 21.4, 22.8, 30.7, 50.3, 51.3, 117.4, 126.8, 127.4, 128.0, 128.1, 129.2, 129.3, 132.1, 136.3, 137.6, 140.9, 142.5, 143.3, 146.2, 172.8; IR (neat): ν 2950, 1733, 1596, 1486, 1438, 1344, 1304, 1154, 1088, 911, 815, 732 cm^{-1}; HRMS (ESI-TOF) Calcd for $C_{26}H_{27}NO_4NaS$ $[M+Na]^+$: 472.1553, found: 472.1553.

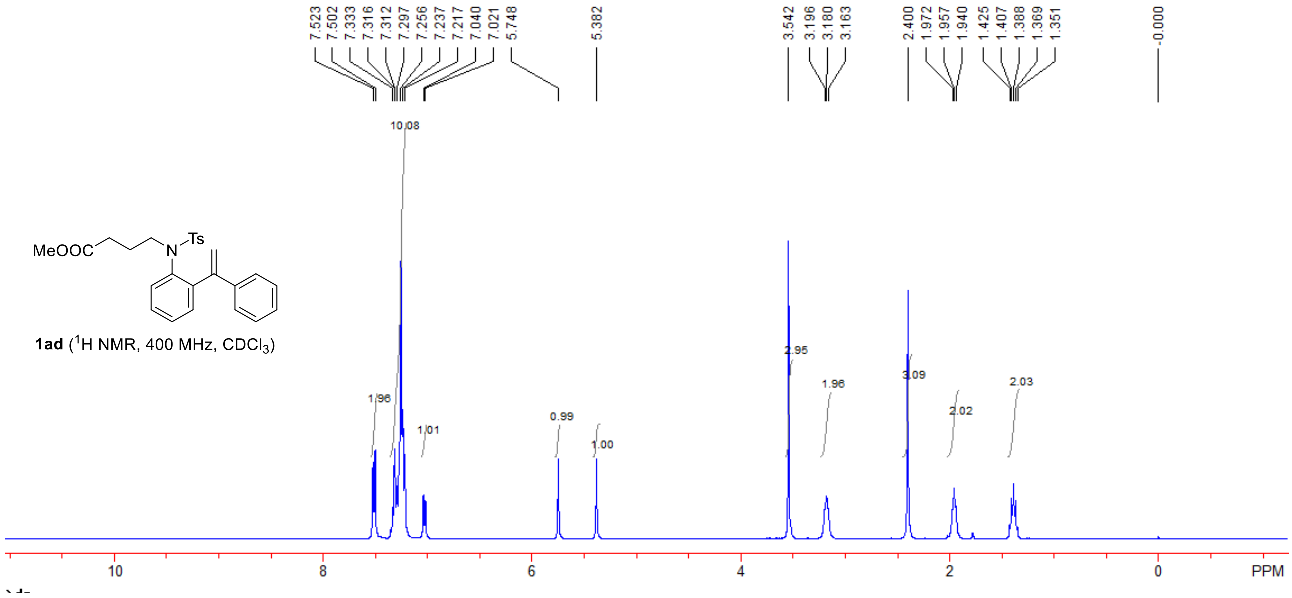

1ad (^{1}H NMR, 400 MHz, $CDCl_3$)

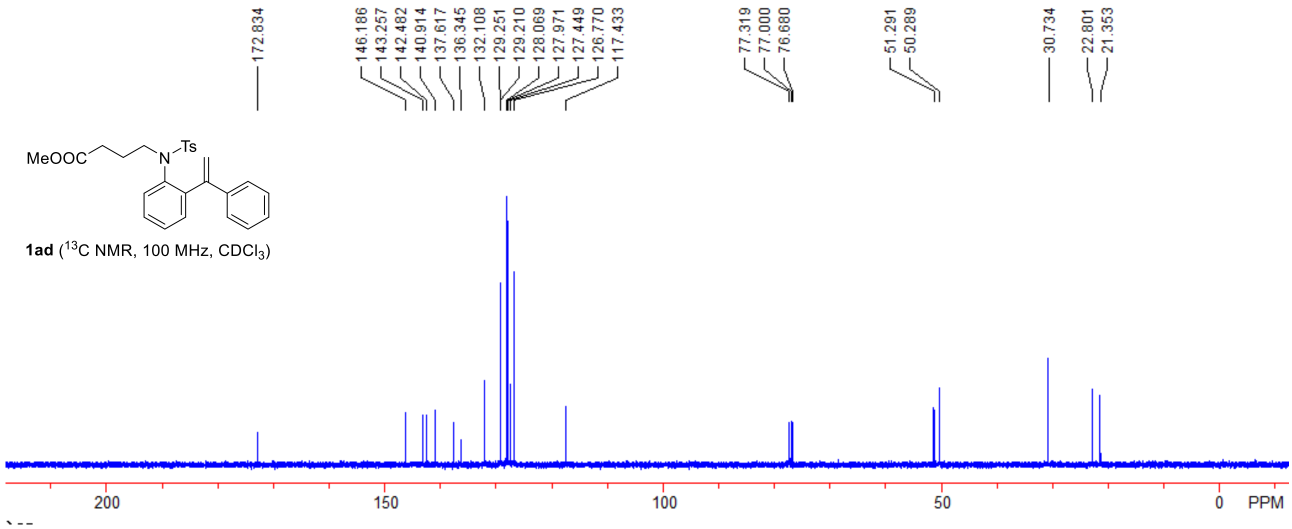

1ad (^{13}C NMR, 100 MHz, $CDCl_3$)

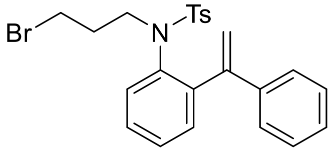

Compound 1ae: Yield: 999 mg, 71%; a yellow oil; Eluent: PE/EA = 10/1. ^{1}H NMR (400 MHz, $CDCl_3$, TMS) δ 1.45-1.65 (m, 2H), 2.42 (s, 3H), 3.00 (t, *J* = 6.0 Hz, 2H), 3.20-3.30 (m, 2H), 5.37 (s, 1H), 5.75 (s, 1H), 7.00 (d, *J* = 7.2 Hz, 1H), 7.21-7.30 (m, 8H), 7.32-7.37 (m, 2H), 7.50 (d, *J* = 8.0 Hz, 2H); ^{13}C{^{1}H}-NMR (101 MHz, $CDCl_3$, TMS) δ_C 21.5, 30.5, 30.6, 50.1, 117.7, 126.8, 127.6, 128.19, 128.25, 128.27, 128.9, 129.4, 132.3, 135.8, 138.0, 141.1, 142.6, 143.5, 146.5; IR (neat): ν 3056, 2969, 1596, 1485, 1444, 1379, 1290, 1153, 1088, 1055, 906, 814, 765, 705 cm^{-1}; HRMS (ESI-TOF) Calcd for $C_{24}H_{24}NO_2NaSBr$ [M+Na]$^+$: 492.0603, found: 492.0611.

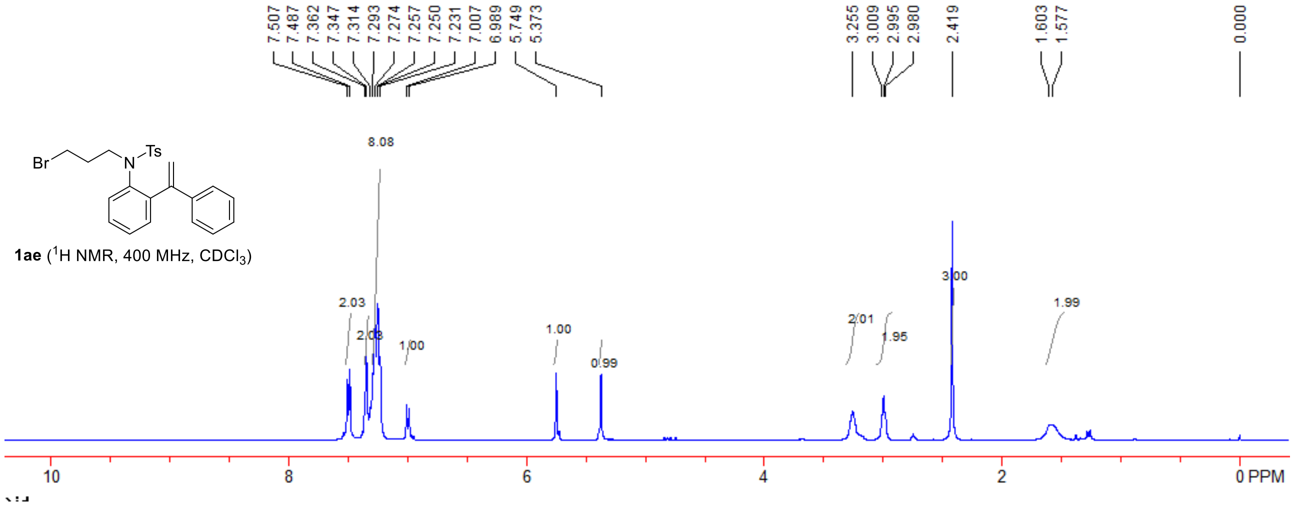

1ae (^{1}H NMR, 400 MHz, $CDCl_3$)

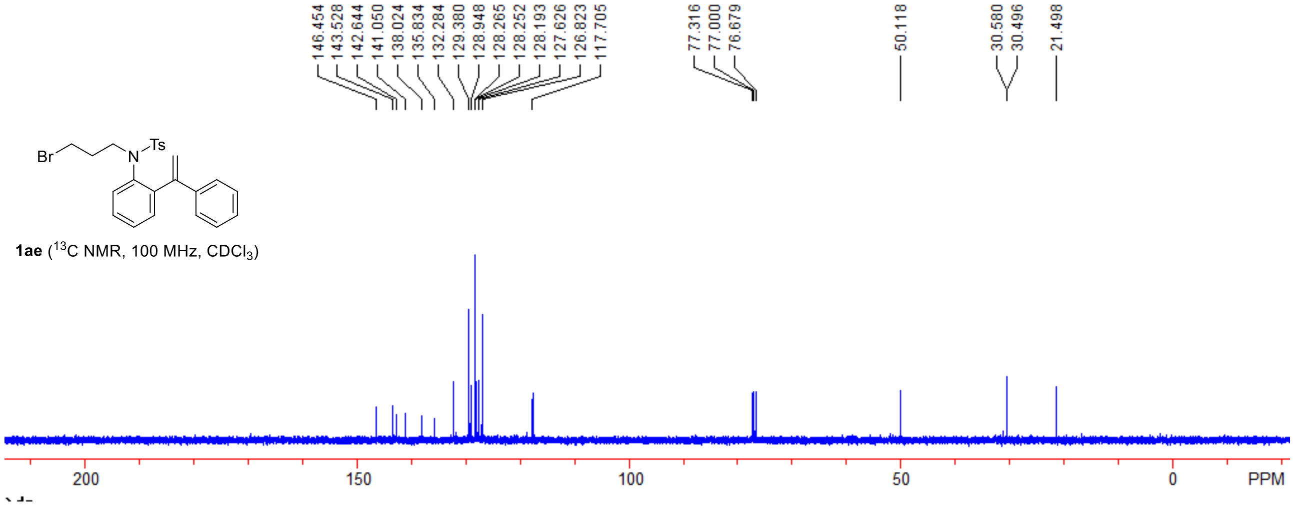

1ae (^{13}C NMR, 100 MHz, $CDCl_3$)

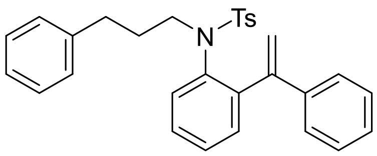

Compound 1af: Yield: 1177 mg, 84%; a yellow oil; Eluent: PE/EA = 10/1. ^{1}H NMR (400 MHz, $CDCl_3$, TMS) δ 1.30-1.45 (m, 2H), 2.22 (t, *J* = 8.0 Hz, 2H), 2.34 (s, 3H), 3.14 (t, *J* = 8.0 Hz, 2H), 5.36 (s, 1H), 5.71 (s, 1H), 6.87 (d, *J* = 7.2 Hz, 2H), 7.02 (d, *J* = 8.0 Hz, 1H), 7.07-7.30 (m, 13H), 7.46 (d, *J* = 8.4 Hz, 2H); ^{13}C{^{1}H}-NMR (101 MHz, $CDCl_3$, TMS) δ_C 21.3, 28.9, 32.6, 50.5, 117.3, 125.6, 126.7, 127.4, 127.8, 127.90, 127.98, 127.99, 129.11, 129.15, 131.9, 136.4, 137.7, 140.7, 140.9, 142.4, 143.0, 146.3; IR (neat): ν 3058, 3025, 2923, 2859, 1596, 1493, 1444, 1378, 1153, 1089, 1028, 909, 814, 767, 732 cm^{-1}; HRMS (ESI-TOF) Calcd for $C_{30}H_{29}NO_2NaS$ $[M+Na]^+$: 490.1811, found: 490.1817.

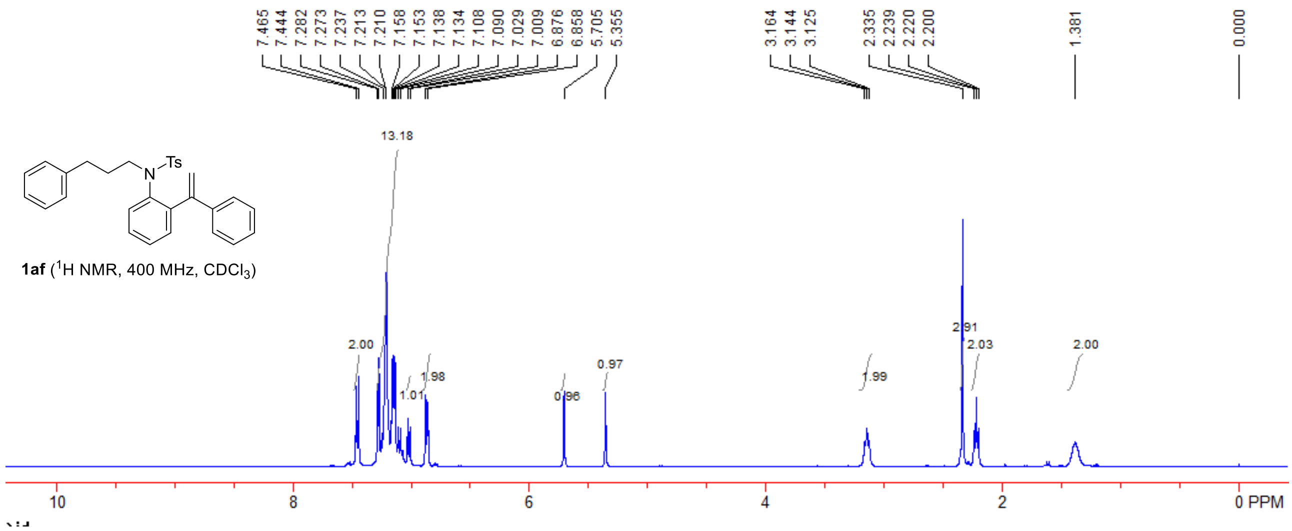
7.465 7.444 7.282 7.273 7.237 7.213 7.210 7.158 7.153 7.138 7.134 7.108 7.090 7.029 7.009 6.876 6.858 5.705 5.355
3.164 3.144 3.125 2.335 2.239 2.220 2.200 1.381 0.000
13.18
2.00 1.01 1.98 0.96 0.97 1.99 2.91 2.03 2.00
N
Ts
1af (1H NMR, 400 MHz, CDCl3)
10 8 6 4 2 0 PPM

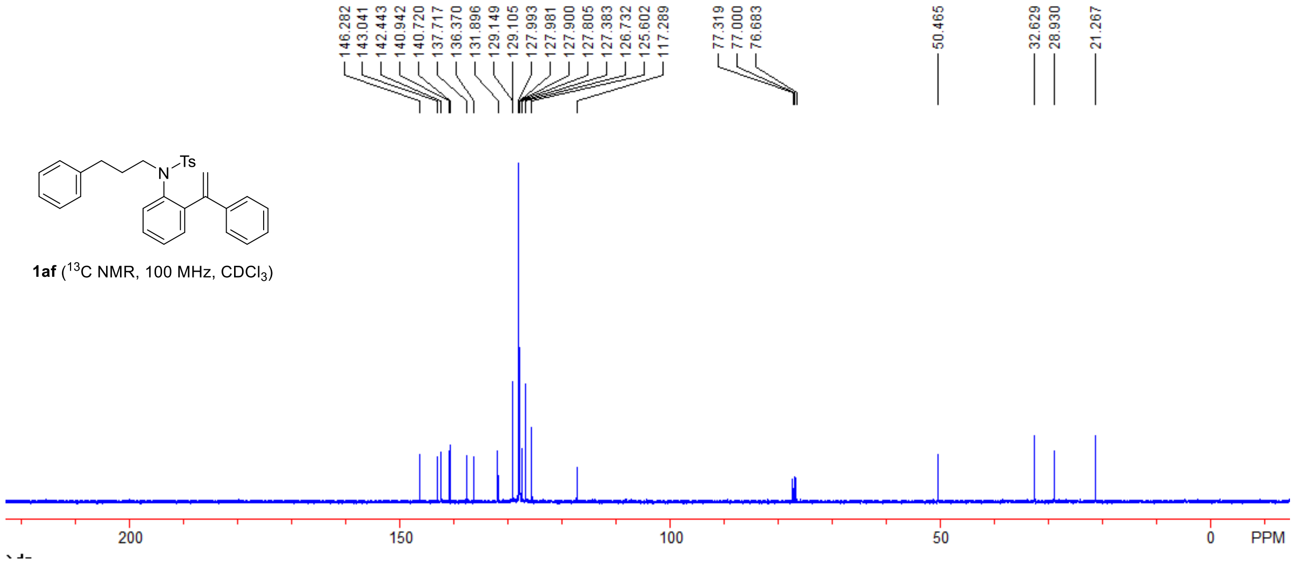
146.282 143.041 142.443 140.942 140.720 137.717 136.370 131.896 129.149 129.105 127.993 127.981 127.900 127.805 127.383 126.732 125.602 117.289
77.319 77.000 76.683 50.465 32.629 28.930 21.267
N
Ts
1af (13C NMR, 100 MHz, CDCl3)
200 150 100 50 0 PPM

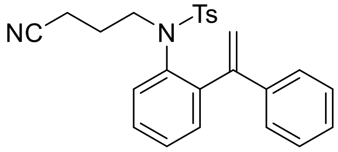

Compound 1ag: Yield: 1124 mg, 90%; a yellow oil; Eluent: PE/EA = 5/1. ^{1}H NMR (400 MHz, $CDCl_3$, TMS) δ 1.15-1.45 (m, 2H), 1.80-2.00 (m, 2H), 2.38 (s, 3H), 3.00-3.40 (m, 2H), 5.37 (s, 1H), 5.75 (s, 1H), 6.93 (d, J = 7.6 Hz, 1H), 7.19-7.30 (m, 8H), 7.31-7.37 (m, 2H), 7.47 (d, J = 8.0 Hz, 2H); ^{13}C{^{1}H}-NMR (101 MHz, $CDCl_3$, TMS) δ_C 14.1, 21.2, 23.6, 50.0, 117.5, 118.6, 126.4, 127.5, 127.9, 128.08, 128.11, 128.16, 128.4, 129.2, 132.1, 135.1, 137.7, 140.7, 142.4, 143.5, 146.1; IR (neat): ν 3055, 2924, 1596, 1486, 1444, 1346, 1156, 1089, 912, 815, 732 cm^{-1}; HRMS (ESI-TOF) Calcd for $C_{25}H_{24}N_2O_2NaS$ $[M+Na]^+$: 439.1451, found: 439.1443.

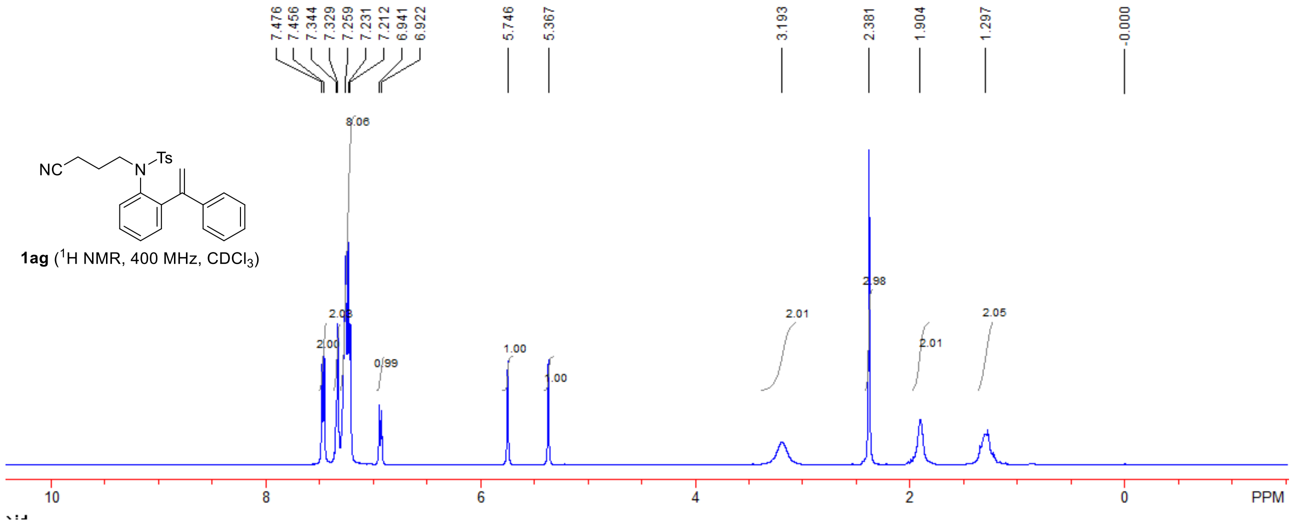

1ag (^{1}H NMR, 400 MHz, $CDCl_3$)

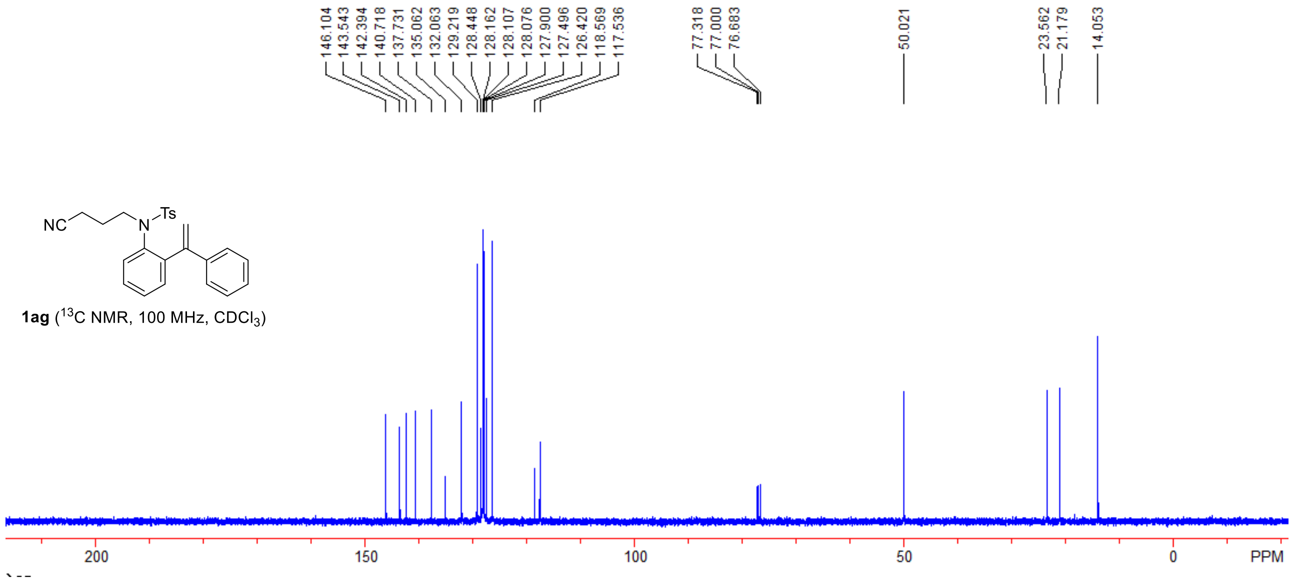

1ag (^{13}C NMR, 100 MHz, $CDCl_3$)

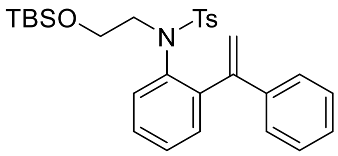

Compound 1ah: Yield: 1248 mg, 82%; a light yellow solid; Mp: 107-109 ^{o}C; Eluent: PE/EA = 10/1. ^{1}H NMR (400 MHz, $CDCl_3$, TMS) δ -0.15 (s, 6H), 0.74 (s, 9H), 2.39 (s, 3H), 3.00-3.40 (m, 4H), 5.41 (s, 1H), 5.72 (s, 1H), 7.03 (d, *J* = 7.2 Hz, 1H), 7.22 (d, *J* = 8.0 Hz, 2H), 7.23-7.30 (m, 6H), 7.31 (d, *J* = 8.0 Hz, 2H), 7.54 (d, *J* = 8.0 Hz, 2H); ^{13}C{^{1}H}-NMR (101 MHz, $CDCl_3$, TMS) δ_C -5.6, 17.9, 21.3, 25.6, 52.6, 60.1, 117.7, 126.9, 127.5, 127.92, 127.97, 128.0, 128.1, 129.19, 129.21, 131.9, 136.4, 138.1, 141.2, 142.5, 143.2, 146.5; IR (neat): ν 2954, 2925, 2882, 2852, 1596, 1492, 1443, 1303, 1273, 1213, 1166, 1071, 1036, 950, 832, 774, 705 cm^{-1}; HRMS (ESI-TOF) Calcd for $C_{29}H_{37}NO_3NaSiS$ $[M+Na]^+$: 530.2156, found: 530.2151.

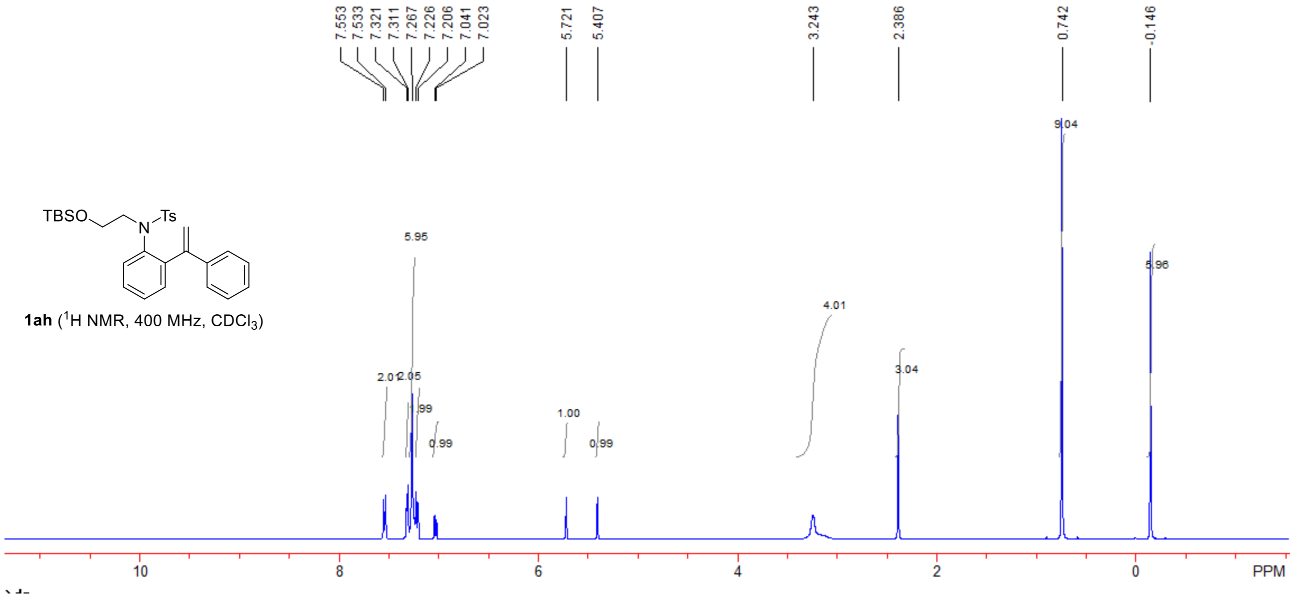
7.553
7.533
7.321
7.311
7.267
7.226
7.206
7.041
7.023
5.721
5.407
3.243
2.386
0.742
-0.146
TBSO
N
Ts
1ah (¹H NMR, 400 MHz, CDCl₃)
5.95
2.01
2.05
1.99
0.99
1.00
0.99
4.01
3.04
9.04
5.96
10
8
6
4
2
0
PPM

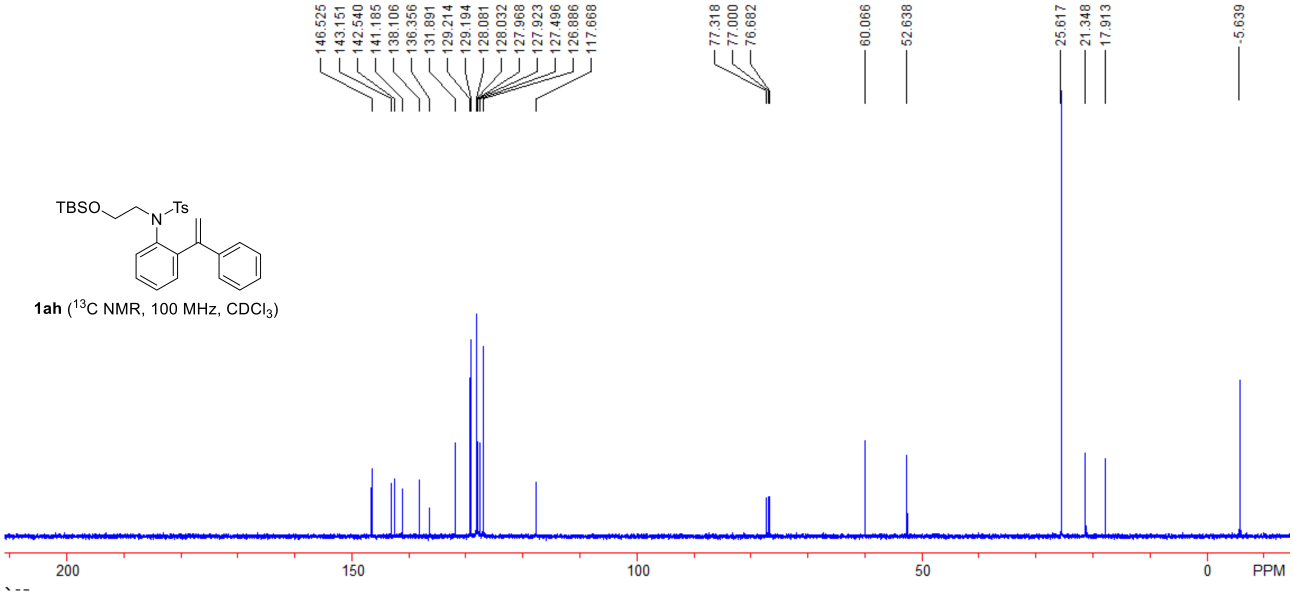
146.525
143.151
142.540
141.185
138.106
136.356
131.891
129.214
129.194
128.081
128.032
127.968
127.923
127.496
126.886
117.668
77.318
77.000
76.682
60.066
52.638
25.617
21.348
17.913
-5.639
TBSO
N
Ts
1ah (¹³C NMR, 100 MHz, CDCl₃)
200
150
100
50
0
PPM

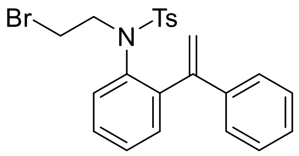

Compound 1ai: Yield: 1038 mg, 76%; a light yellow solid; Mp: 114-116 °C; Eluent: PE/EA = 10/1. ^{1}H NMR (400 MHz, $CDCl_3$, TMS) δ 2.38 (s, 3H), 2.50-3.20 (m, 2H), 3.32-3.48 (m, 2H), 5.32 (s, 1H), 5.69 (s, 1H), 6.90 (d, *J* = 7.6 Hz, 1H), 7.18-7.30 (m, 8H), 7.31-7.37 (m, 2H), 7.47 (d, *J* = 8.0 Hz, 2H); ^{13}C{^{1}H}-NMR (101 MHz, $CDCl_3$, TMS) δ_C 21.4, 27.7, 52.8, 117.6, 126.6, 127.6, 128.0, 128.2, 128.3, 128.4, 128.9, 129.3, 132.1, 135.1, 137.9, 141.1, 142.6, 143.7, 146.6; IR (neat): ν 3063, 2971, 2917, 1595, 1492, 1444, 1355, 1304, 1233, 1156, 1083, 972, 876, 773, 707 cm^{-1}; HRMS (EI-TOF) Calcd for $C_{23}H_{22}NO_2SBr$ [M]$^+$: 455.0549, found: 455.0553.

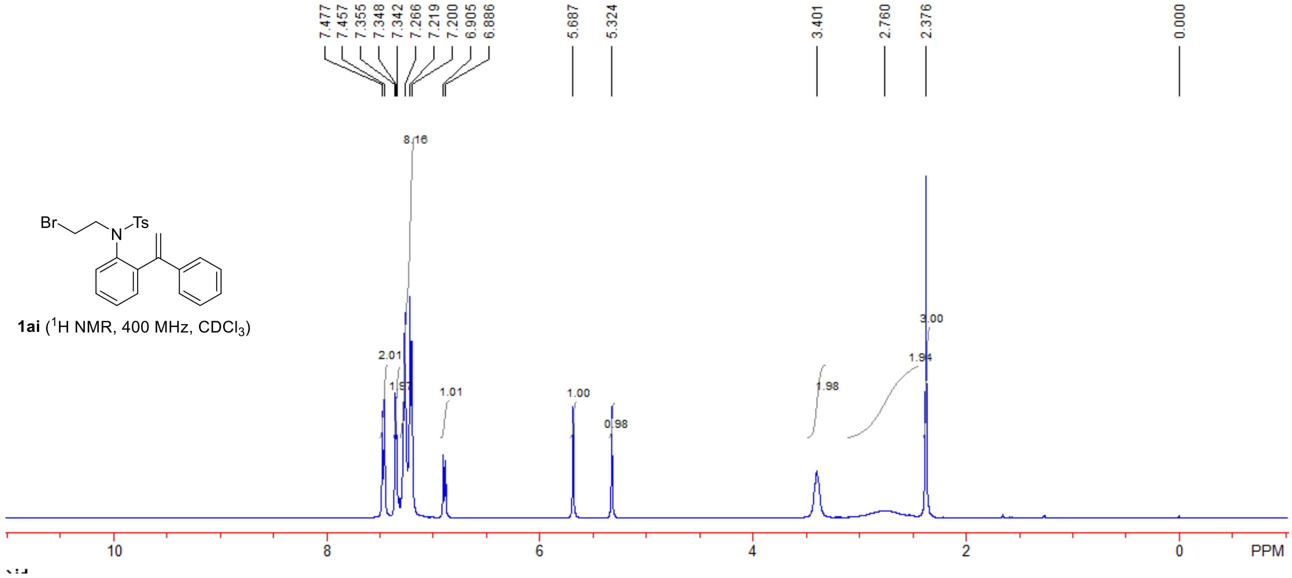

1ai (^{1}H NMR, 400 MHz, $CDCl_3$)

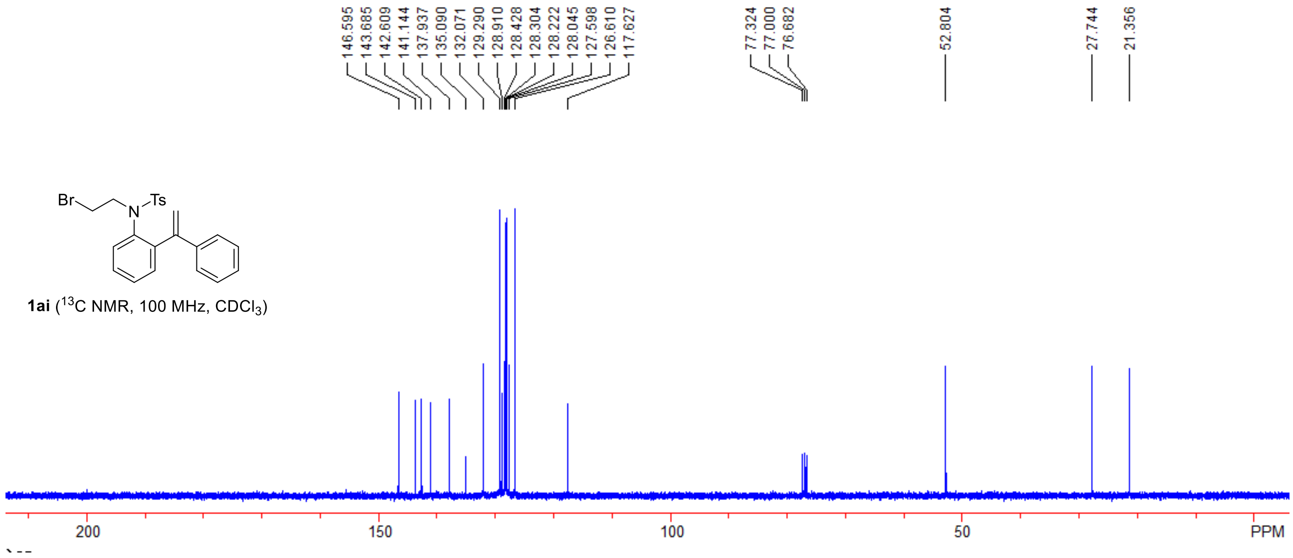

1ai (^{13}C NMR, 100 MHz, $CDCl_3$)

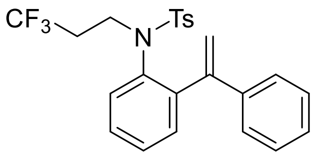

Compound 1aj: Yield: 1028 mg, 77%; a light yellow solid; Mp: 104-106 °C; Eluent: PE/EA = 10/1. ^{1}H NMR (400 MHz, $CDCl_3$, TMS) δ 1.30-2.20 (m, 2H), 2.39 (s, 3H), 3.00-3.50 (m, 2H), 5.35 (s, 1H), 5.71 (s, 1H), 6.83 (d, *J* = 8.0 Hz, 1H), 7.20-7.32 (m, 8H), 7.35-7.49 (m, 4H); ^{13}C{^{1}H}-NMR (101 MHz, $CDCl_3$, TMS) δ_C 21.4, 32.3 (q, *J* = 27.6 Hz), 45.0 (q, *J* = 4.5 Hz), 117.7, 125.4 (q, *J* = 274.7 Hz), 126.7, 127.6, 128.2, 128.33, 128.38, 128.45, 128.6, 129.4, 132.2, 134.5, 138.3, 141.5, 143.0, 143.9, 147.0; ^{19}F NMR (376 MHz, $CDCl_3$) δ -65.9; IR (neat): ν 3094, 3001, 2941, 1597, 1487, 1445, 1392, 1306, 1259, 1238, 1184, 1115, 1074, 981, 787, 711 cm^{-1}; HRMS (ESI-TOF) Calcd for $C_{24}H_{22}NO_2F_3NaS$ [M+Na]$^+$: 468.1216, found: 468.1221.

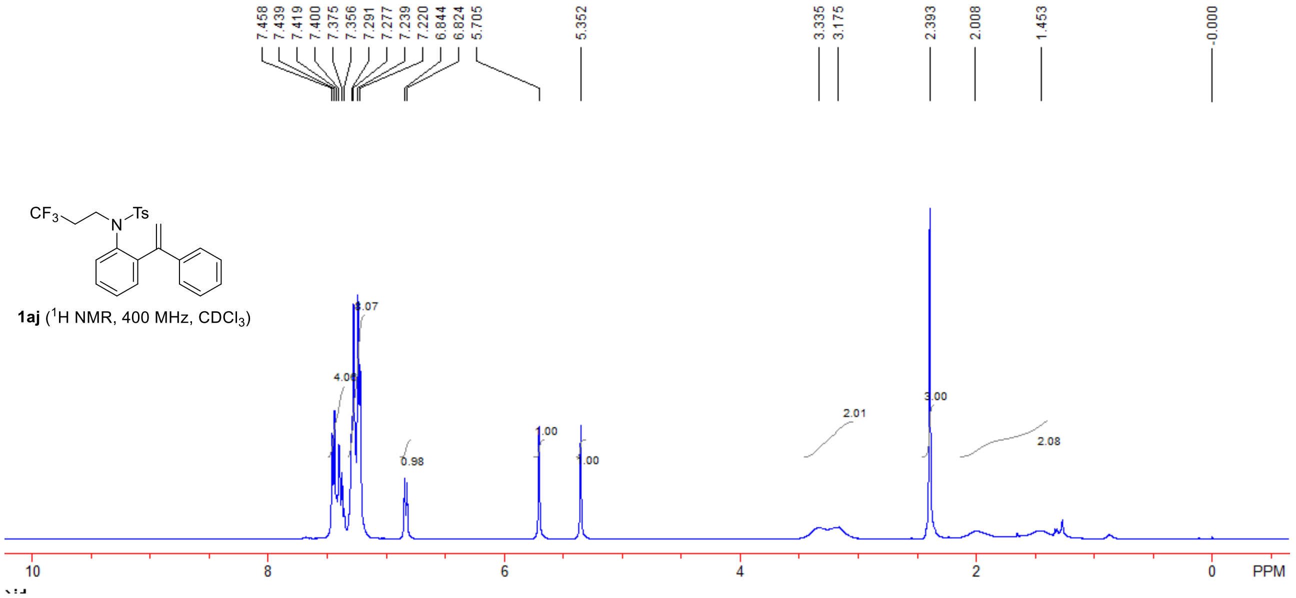
7.458
7.439
7.419
7.400
7.375
7.356
7.291
7.277
7.239
7.220
6.844
6.824
5.705
5.352
3.335
3.175
2.393
2.008
1.453
-0.000
CF3
N
Ts
1aj (1H NMR, 400 MHz, CDCl3)
4.06
3.07
0.98
1.00
1.00
2.01
3.00
2.08
10
8
6
4
2
0
PPM

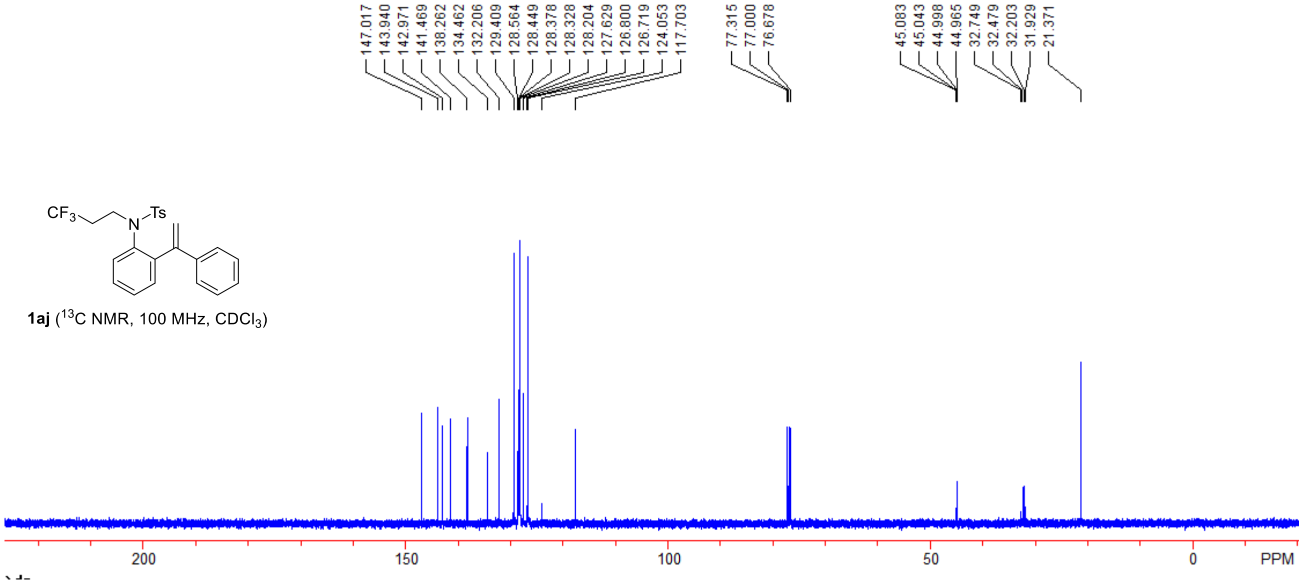
147.017
143.940
142.971
141.469
138.262
134.462
132.206
129.409
128.564
128.449
128.378
128.328
128.204
127.629
126.800
126.719
124.053
117.703
77.315
77.000
76.678
45.083
45.043
44.998
44.965
32.749
32.479
32.203
31.929
21.371
CF3
N
Ts
1aj (13C NMR, 100 MHz, CDCl3)
200
150
100
50
0
PPM

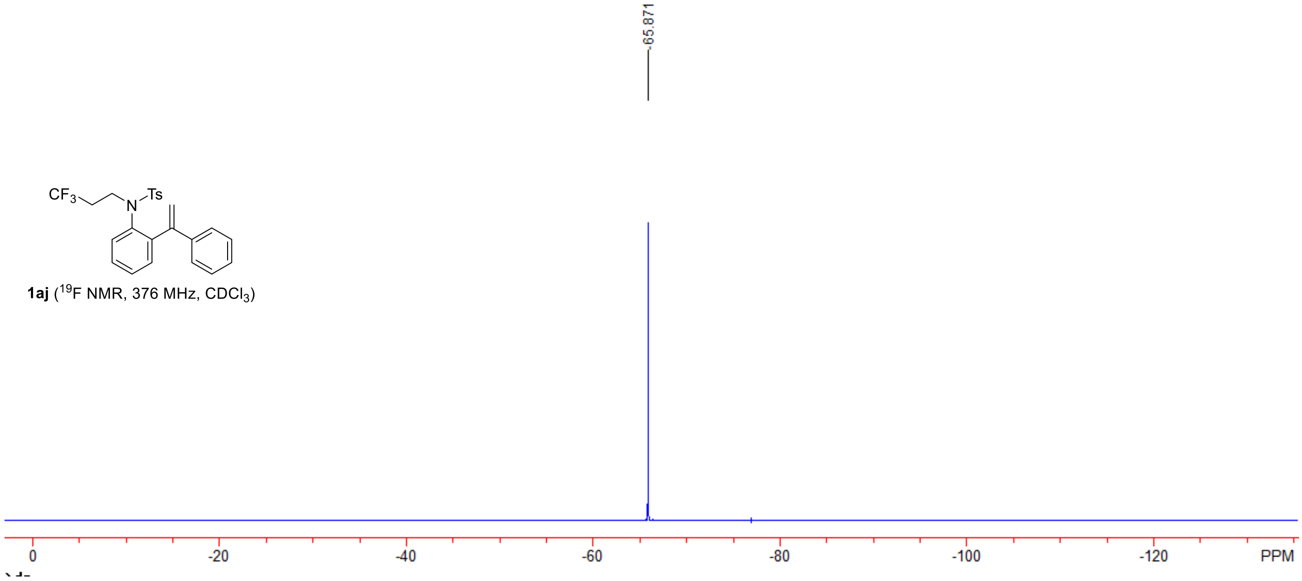
-65.871
CF3
N
Ts
1aj (19F NMR, 376 MHz, CDCl3)
0
-20
-40
-60
-80
-100
-120
PPM

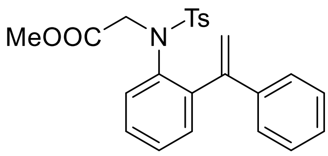

Compound 1ak: Yield: 1150 mg, 91%; a light yellow solid; Mp: 96-98 °C; Eluent: PE/EA = 5/1. ^{1}H NMR (400 MHz, $CDCl_3$, TMS) δ 2.41 (s, 3H), 3.48 (s, 3H), 3.75-3.95 (m, 2H), 5.25 (s, 1H), 5.62 (s, 1H), 7.15-7.40 (m, 10H), 7.48 (d, *J* = 7.6 Hz, 1H), 7.57 (d, *J* = 8.4 Hz, 2H); $^{13}C\{^{1}H\}$-NMR (101 MHz, $CDCl_3$, TMS) δ_C 21.5, 51.5, 51.8, 117.5, 126.9, 127.8, 127.9, 128.3, 128.6, 129.1, 131.3, 131.7, 137.5, 137.6, 140.8, 142.1, 143.3, 146.3, 169.4; IR (neat): ν 2983, 2953, 2928, 1750, 1594, 1445, 1346, 1238, 1163, 1089, 1023, 909, 786, 701 cm^{-1}; HRMS (ESI-TOF) Calcd for $C_{24}H_{23}NO_4NaS$ $[M+Na]^+$: 444.1240, found: 444.1239.

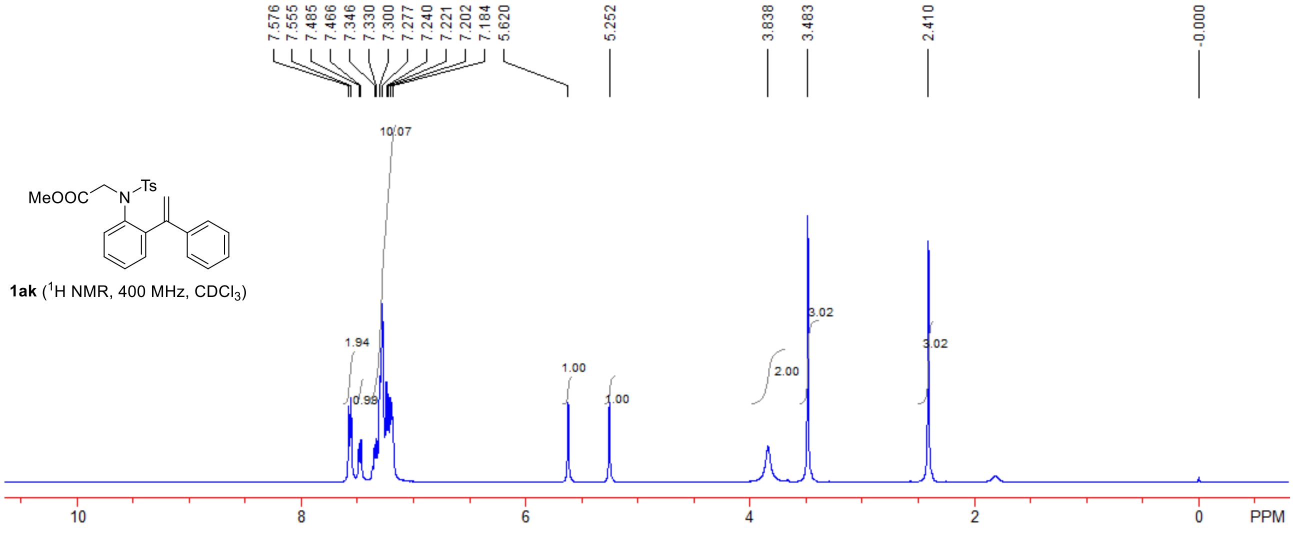
7.576
7.555
7.485
7.466
7.346
7.330
7.300
7.277
7.240
7.221
7.202
7.184
5.620
5.252
3.838
3.483
2.410
-0.000
10.07
1.94
0.98
1.00
1.00
2.00
3.02
3.02
MeOOC
N
Ts
1ak (1H NMR, 400 MHz, CDCl3)
10
8
6
4
2
0
PPM

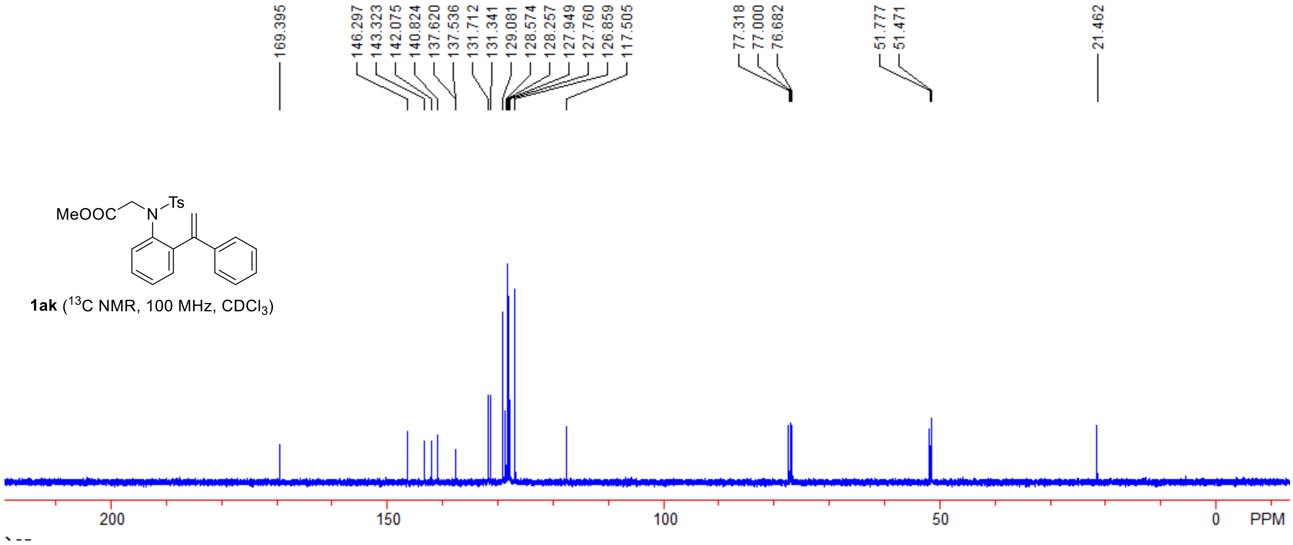
169.395
146.297
143.323
142.075
140.824
137.620
137.536
131.712
131.341
129.081
128.574
128.257
127.949
127.760
126.859
117.505
77.318
77.000
76.682
51.777
51.471
21.462
MeOOC
N
Ts
1ak (13C NMR, 100 MHz, CDCl3)
200
150
100
50
0
PPM

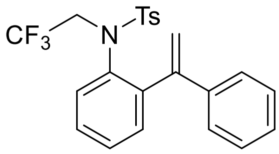

Compound 1al: Yield: 1086 mg, 84%; a light yellow solid; Mp: 90-92 °C; Eluent: PE/EA = 10/1. ^{1}H NMR (400 MHz, $CDCl_3$, TMS) δ 2.39 (s, 3H), 3.30-3.50 (m, 1H), 4.25-4.45 (m, 1H), 5.33 (s, 1H), 5.74 (s, 1H), 7.18-7.25 (m, 8H), 7.27-7.35 (m, 3H), 7.63 (d, *J* = 8.0 Hz, 2H); $^{13}C\{^{1}H\}$-NMR (101 MHz, $CDCl_3$, TMS) δ_C 21.4, 50.1 (q, *J* = 33.8 Hz), 117.6, 123.9 (q, *J* =279.5 Hz), 126.7, 127.93, 127.98, 128.2, 128.4, 128.7, 129.4, 130.80, 130.85, 132.1, 136.4, 137.4, 140.2, 141.0, 143.9, 145.0; ^{19}F NMR (376 MHz, $CDCl_3$) δ -68.5; IR (neat): ν 3094, 3001, 2941, 1620, 1597, 1445, 1353, 1259, 1184, 1115, 1087, 1040, 981, 827, 768, 711 cm^{-1}; HRMS (ESI-TOF) Calcd for $C_{23}H_{20}NO_2F_3NaS$ $[M+Na]^+$: 454.1059, found: 454.1065.

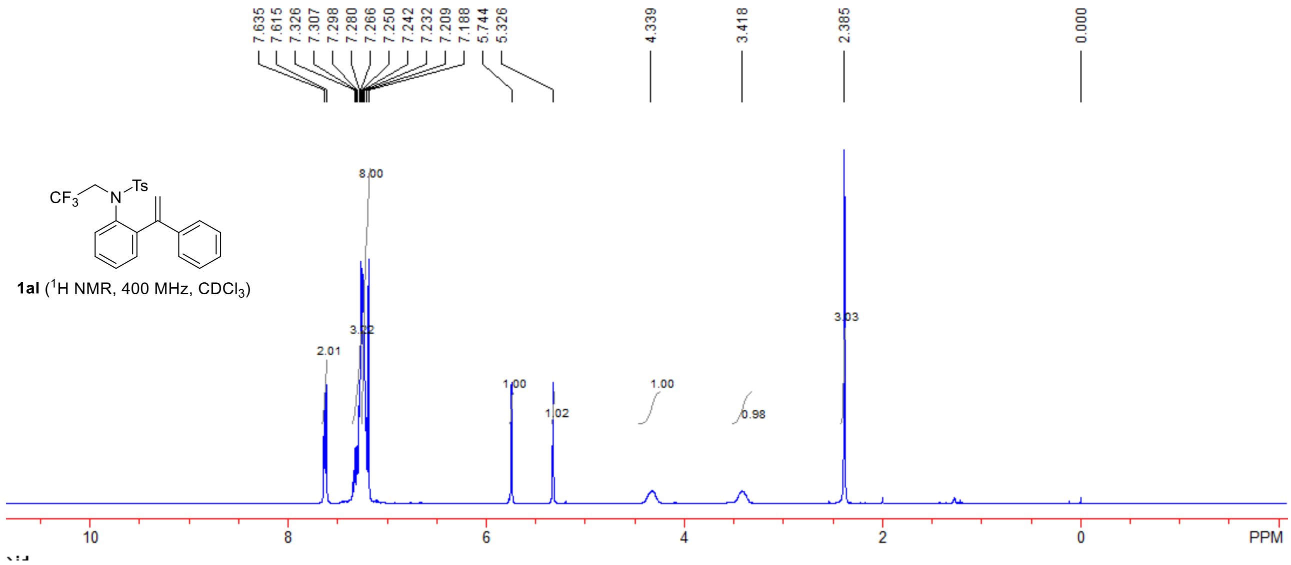
7.635
7.615
7.326
7.307
7.298
7.280
7.266
7.250
7.242
7.232
7.209
7.188
5.744
5.326
4.339
3.418
2.385
0.000
CF3
N
Ts
8.00
3.22
2.01
1.00
1.02
1.00
0.98
3.03
10
8
6
4
2
0
PPM
1al (1H NMR, 400 MHz, CDCl3)

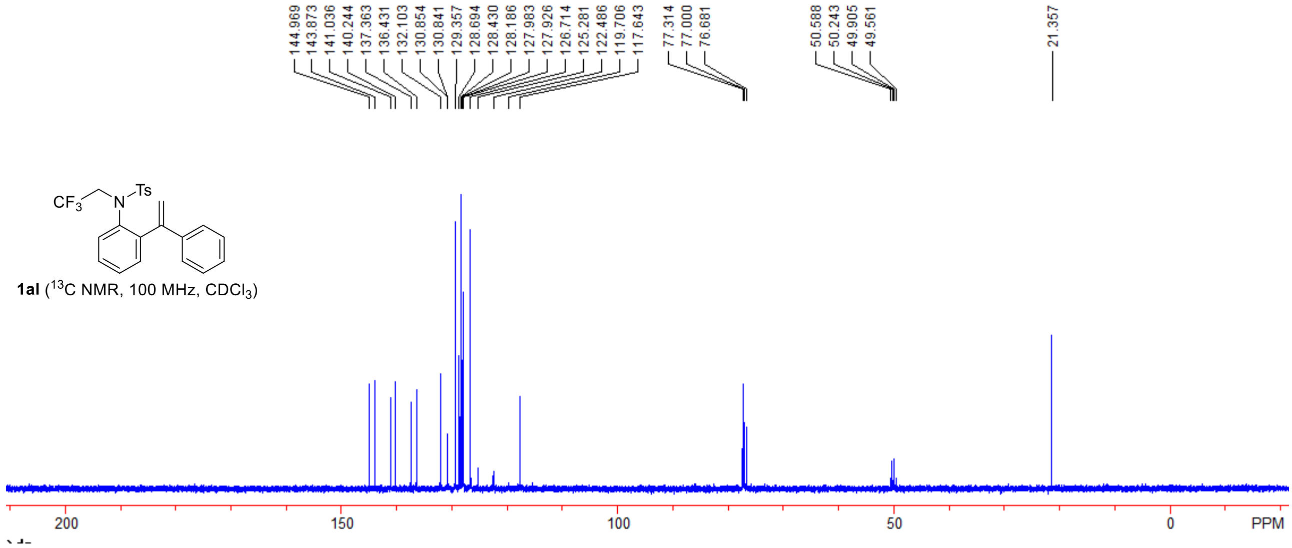
144.969
143.873
141.036
140.244
137.363
136.431
132.103
130.854
130.841
129.357
128.694
128.430
128.186
127.983
127.926
126.714
125.281
122.486
119.706
117.643
77.314
77.000
76.681
50.588
50.243
49.905
49.561
21.357
CF3
N
Ts
200
150
100
50
0
PPM
1al (13C NMR, 100 MHz, CDCl3)

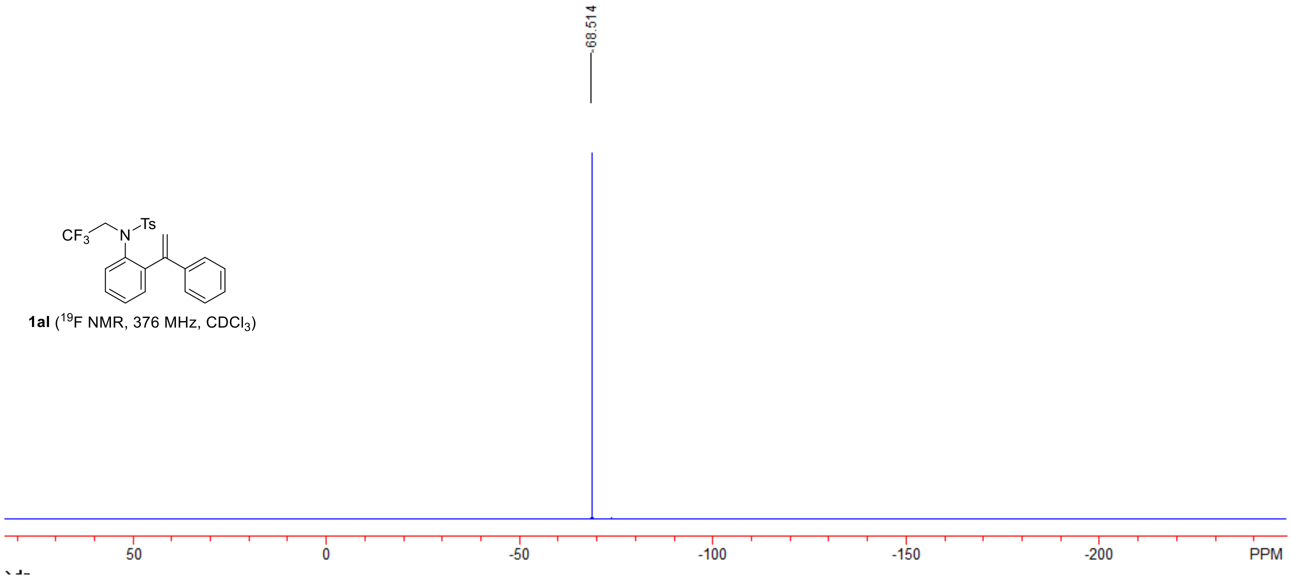
-68.514
CF3
N
Ts
50
0
-50
-100
-150
-200
PPM
1al (19F NMR, 376 MHz, CDCl3)

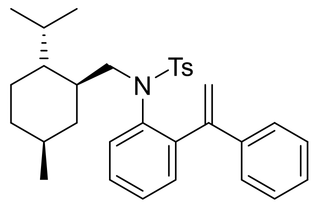

Compound 1am: Yield: 1428 mg, 70%; a light yellow solid; Mp: 147-149 ^{o}C; Eluent: PE/EA = 10/1. ^{1}H NMR (400 MHz, $CDCl_3$, TMS) δ 0.05-0.20 (m, 1.33H), 0.25-0.45 (m, 5.33H), 0.52-0.76 (m, 11.97H), 0.85-1.00 (m, 0.33H), 1.14-1.19 (m, 1.33H), 1.32-1.55 (m, 3.33H), 1.56-1.70 (m, 1.00H), 1.84 (d, *J* = 12.4 Hz, 1.00H), 2.35-2.46 (m, 3.99H), 2.90-3.06 (m, 1.33H), 3.32-3.45 (m, 1.33H), 5.40 (s, 0.33H), 5.69 (s, 1.00H), 5.74 (s, 0.33H), 6.02 (s, 1.00H), 7.11 (d, *J* = 7.6 Hz, 1.00H), 7.20-7.40 (m, 13.33H), 7.50 (d, *J* = 7.6 Hz, 2.00H), 7.61 (d, *J* = 7.6 Hz, 1.00H); ^{13}C{^{1}H}-NMR (101 MHz, $CDCl_3$, TMS) δ_C 14.6, 15.4, 19.9, 21.1, 21.3, 21.4, 22.3, 22.4, 23.77, 23.84, 25.9, 26.2, 31.1, 31.6, 34.61, 34.67, 36.7, 36.8, 39.89, 39.98, 44.6, 45.4, 54.9, 57.1, 117.8, 118.1, 126.7, 126.8, 127.44, 127.49, 127.7, 127.88, 127.95, 128.1, 128.2, 128.5, 128.6, 129.07, 129.13, 129.17, 130.3, 132.5, 135.9, 139.5, 140.4, 140.6, 142.0, 142.5, 143.0, 143.3, 144.9; IR (neat): ν 2952, 2918, 2867, 1596, 1488, 1444, 1384, 1333, 1186, 1163, 1114, 983, 853, 715 cm^{-1}; HRMS (ESI-TOF) Calcd for $C_{32}H_{39}NO_2NaS$ $[M+Na]^+$: 524.2594, found: 524.2593.

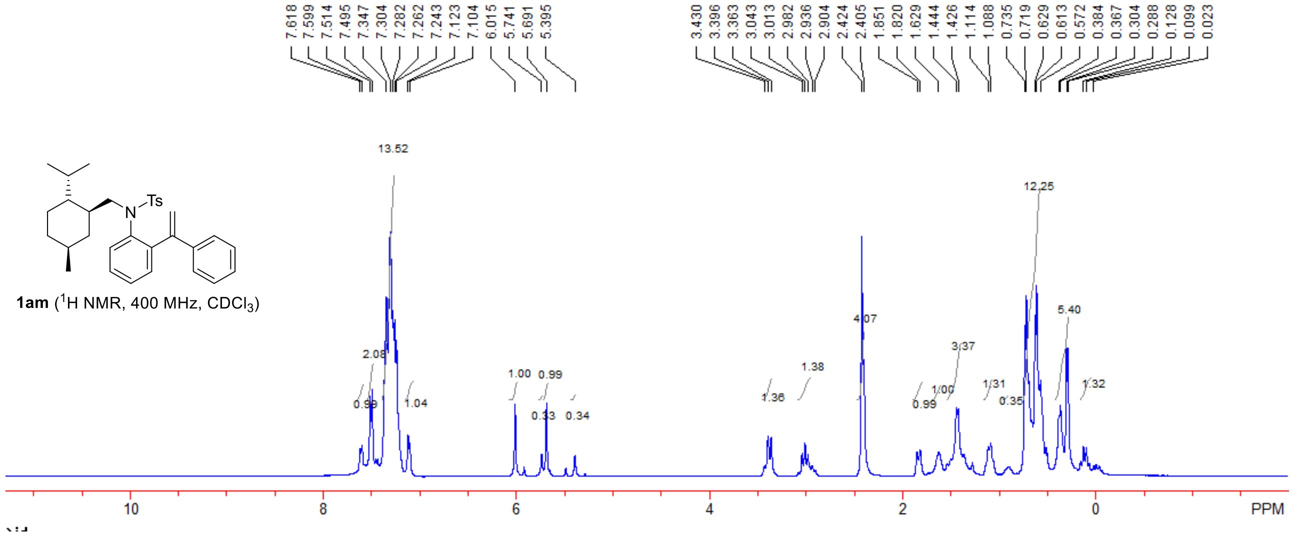
1am (¹H NMR, 400 MHz, CDCl₃)
PPM

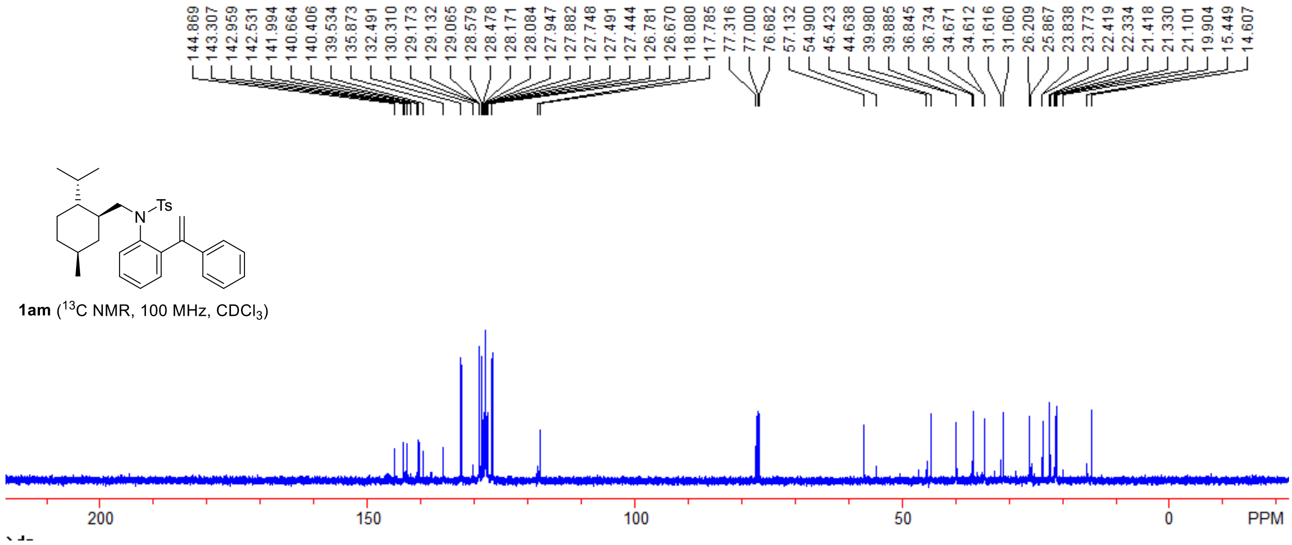
1am (¹³C NMR, 100 MHz, CDCl₃)
PPM

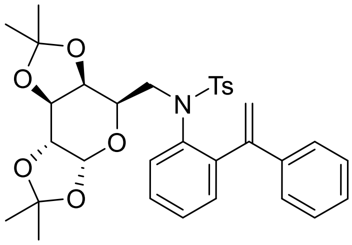

Compound 1an: Yield: 2834 mg, 51%; a yellow oil; Eluent: PE/EA = 5/1. ^{1}H NMR (400 MHz, $CDCl_3$, TMS) δ 1.02 (s, 3.00H), 1.15-1.30 (m, 11.20H), 1.36 (s, 3.00H), 2.40 (s, 4.20H), 3.22-3.34 (m, 1.40H), 3.38-3.50 (m, 2.00H), 3.73-4.00 (m, 2.40H), 4.10-4.20 (m, 1.40H), 4.34 (d, *J* = 7.2 Hz, 1.00H), 4.45 (d, *J* = 7.2 Hz, 0.40H), 5.14 (d, *J* = 5.2 Hz, 1.00H), 5.29-5.34 (m, 0.80H), 5.48 (s, 1.00H), 5.65 (s, 0.40H), 5.81 (s, 1.00H), 7.15-7.35 (m, 15.40H), 7.60 (d, *J* = 7.6 Hz, 0.80H), 7.67 (d, *J* = 8.0 Hz, 2.00H); ^{13}C{^{1}H}-NMR (101 MHz, $CDCl_3$, TMS) δ_C 21.4, 24.3, 24.7, 24.9, 25.4, 25.6, 25.8, 25.9, 49.5, 50.6, 64.6, 66.7, 69.98, 70.03, 70.5, 71.0, 71.4, 95.9, 96.2, 108.1, 108.4, 108.96, 109.04, 117.0, 117.5, 126.8, 127.2, 127.4, 127.61, 127.64, 127.70, 127.73, 128.00, 128.10, 128.15, 128.18, 128.3, 128.8, 129.3, 130.8, 131.4, 131.7, 136.6, 137.4, 137.8, 140.6, 141.0, 141.3, 141.4, 142.7, 143.1, 145.4, 145.7; IR (neat): ν 3055, 2985, 2933, 1597, 1487, 1444, 1381, 1305, 1254, 1162, 1089, 1002, 961, 861, 782, 704 cm^{-1}; HRMS (ESI-TOF) Calcd for $C_{33}H_{37}NO_7NaS$ $[M+Na]^+$: 614.2183, found: 614.2181.

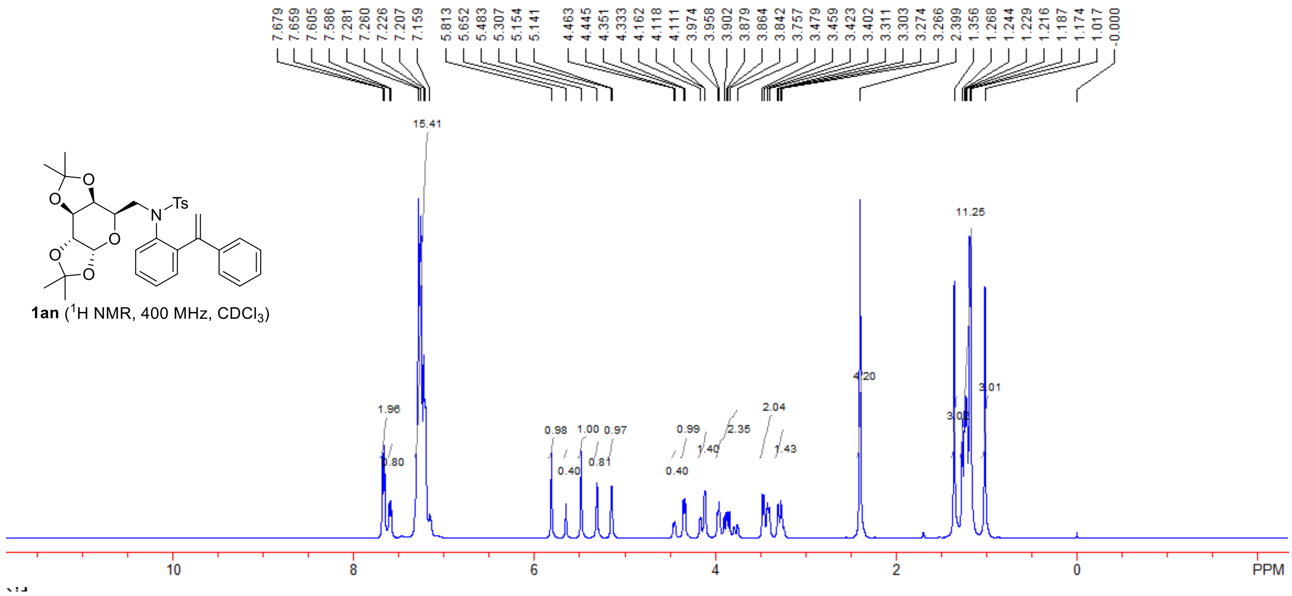

1an (1H NMR, 400 MHz, CDCl3)
PPM

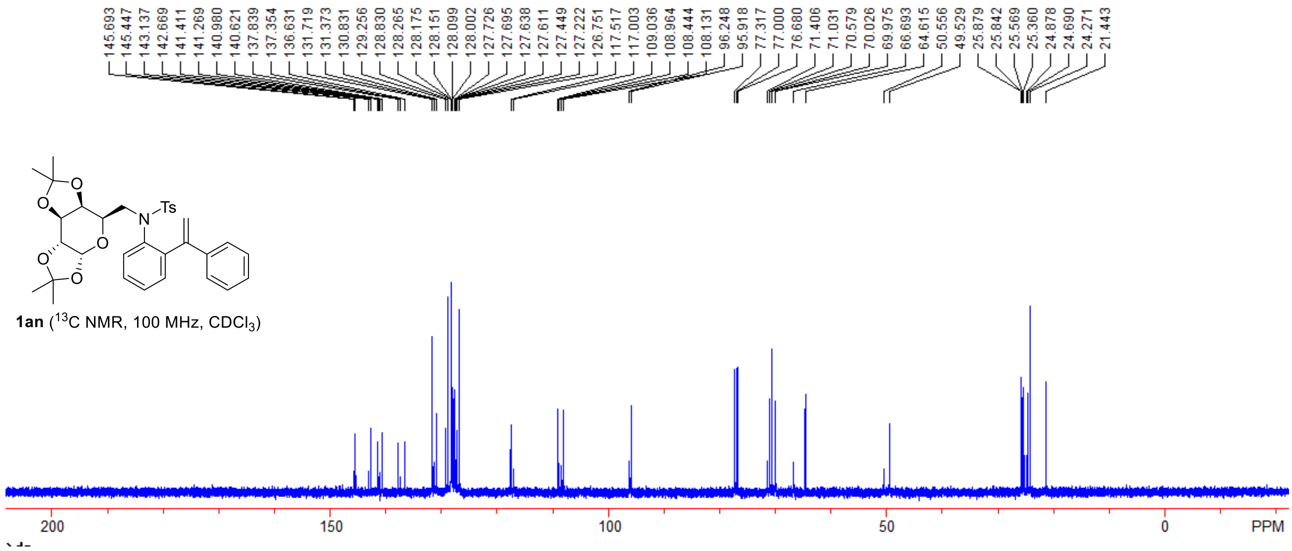

1an (13C NMR, 100 MHz, CDCl3)
PPM

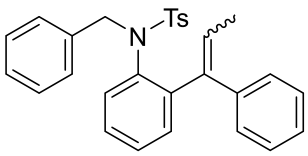

Compound 1ao: Yield: 2219 mg, 81%; a light yellow solid; Mp: 116-118 °C; Eluent: PE/EA = 10/1. ^{1}H NMR (400 MHz, $CDCl_3$, TMS) δ 1.64 (d, *J* = 6.4 Hz, 2.70H), 1.70 (d, *J* = 6.8 Hz, 3.00H), 2.33 (s, 2.70H), 2.36 (s, 3.00H), 3.20-3.80 (m, 1.90H), 4.30-4.65 (m, 1.90H), 5.54 (q, *J* = 7.2 Hz, 0.90H), 6.14 (q, *J* = 6.8 Hz, 1.00H), 6.62-6.70 (m, 2.90H), 6.87 (d, *J* = 6.8 Hz, 2.00H), 6.96-7.03 (m, 3.00H), 7.04-7.13 (m, 8.60H), 7.16-7.23 (m, 7.90H), 7.24-7.32 (m, 6.20H), 7.40-7.57 (m, 3.80H); ^{13}C{^{1}H}-NMR (101 MHz, $CDCl_3$, TMS) δ_C 15.3, 15.9, 21.25, 21.30, 53.7, 53.9, 126.6, 126.7, 127.0, 127.3, 127.5, 127.67, 127.71, 127.81, 127.83, 128.1, 128.99, 129.05, 129.4, 129.52, 129.59, 131.7, 132.0, 132.6, 135.3, 135.7, 136.3, 137.6, 137.7, 139.2, 139.6, 140.0, 142.2, 142.8, 142.9, 145.1; IR (neat): ν 3059, 3025, 2977, 2910, 1597, 1493, 1444, 1306, 1181, 1155, 1028, 917, 816, 727 cm^{-1}; HRMS (ESI-TOF) Calcd for $C_{29}H_{27}NO_2NaS$ [M+Na]$^+$: 476.1655, found: 476.1655.

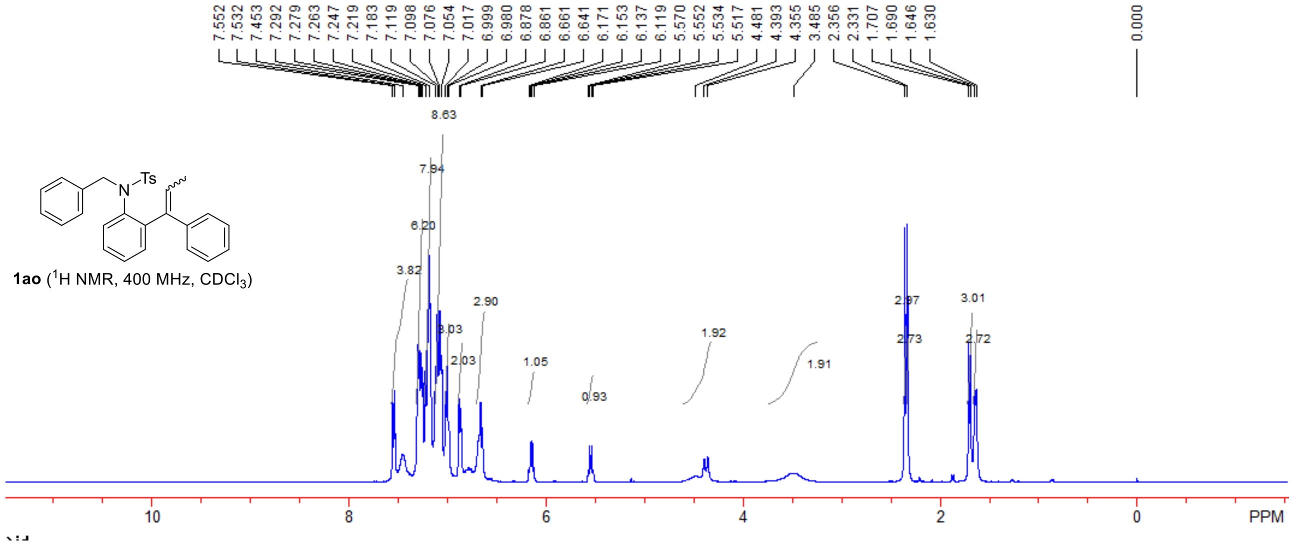

1ao (^{1}H NMR, 400 MHz, $CDCl_3$)

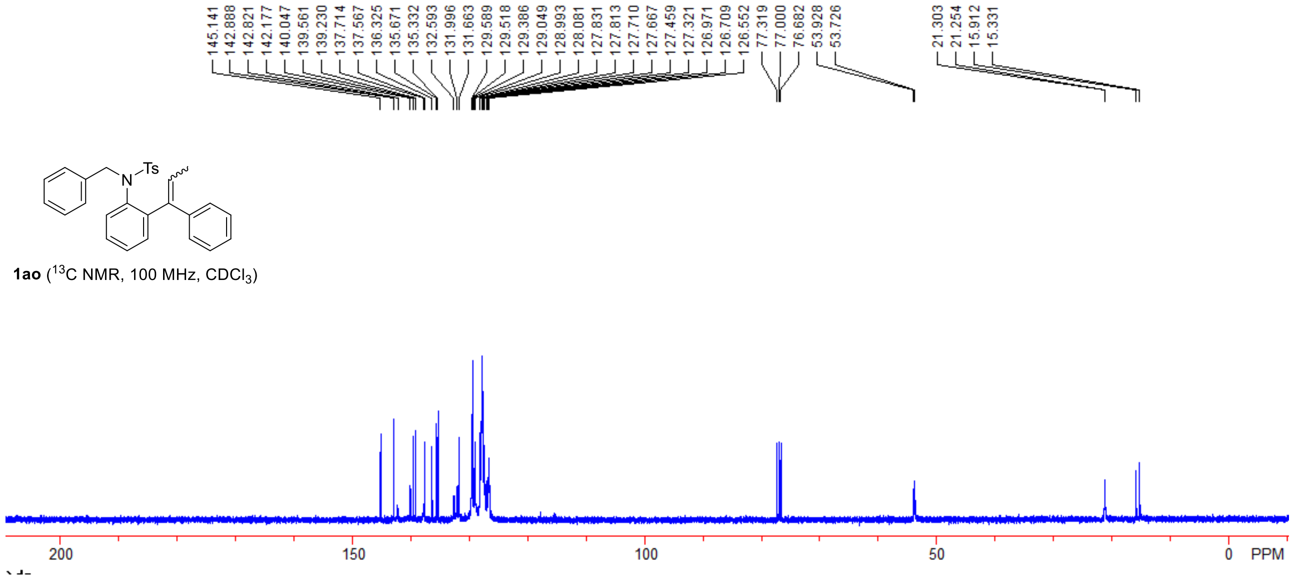

1ao (^{13}C NMR, 100 MHz, $CDCl_3$)

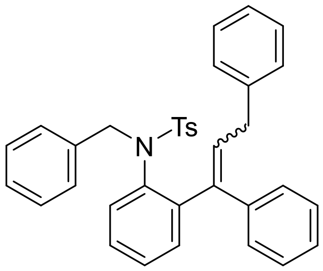

Compound 1ap: Yield: 1848 mg, 79%; a light yellow solid; Mp: 179-181 °C; Eluent: PE/EA = 10/1. ^{1}H NMR (400 MHz, $CDCl_3$, TMS) δ 2.36 (s, 4.20H), 3.30-3.70 (m, 4.20H), 4.25-4.60 (m, 1.40H), 5.64 (t, *J* = 7.6 Hz, 0.40H), 6.31 (d, *J* = 7.2 Hz, 1.00H), 6.50-6.82 (m, 4.20H), 6.94-7.30 (m, 25.20H), 7.38-7.58 (m, 4.20H); ^{13}C{^{1}H}-NMR (101 MHz, $CDCl_3$, TMS) δ_C 21.4, 35.6, 36.3, 53.9, 54.2, 125.76, 125.85, 126.92, 127.0, 127.1, 127.2, 127.4, 127.6, 127.8, 127.90, 127.97, 128.00, 128.04, 128.16, 128.24, 128.31, 128.33, 128.35, 128.42, 128.44, 129.1, 129.5, 129.7, 129.8, 131.38, 131.45, 132.0, 132.2, 132.3, 135.4, 135.6, 136.5, 137.4, 138.0, 139.6, 139.7, 140.0, 140.7, 141.0, 142.1, 143.0, 145.0; IR (neat): ν 3060, 3023, 1595, 1492, 1447, 1359, 1302, 1218, 1090, 1029, 994, 919, 878, 776, 703 cm^{-1}; HRMS (ESI-TOF) Calcd for $C_{35}H_{31}NO_2NaS$ $[M+Na]^+$: 552.1968, found: 552.1974.

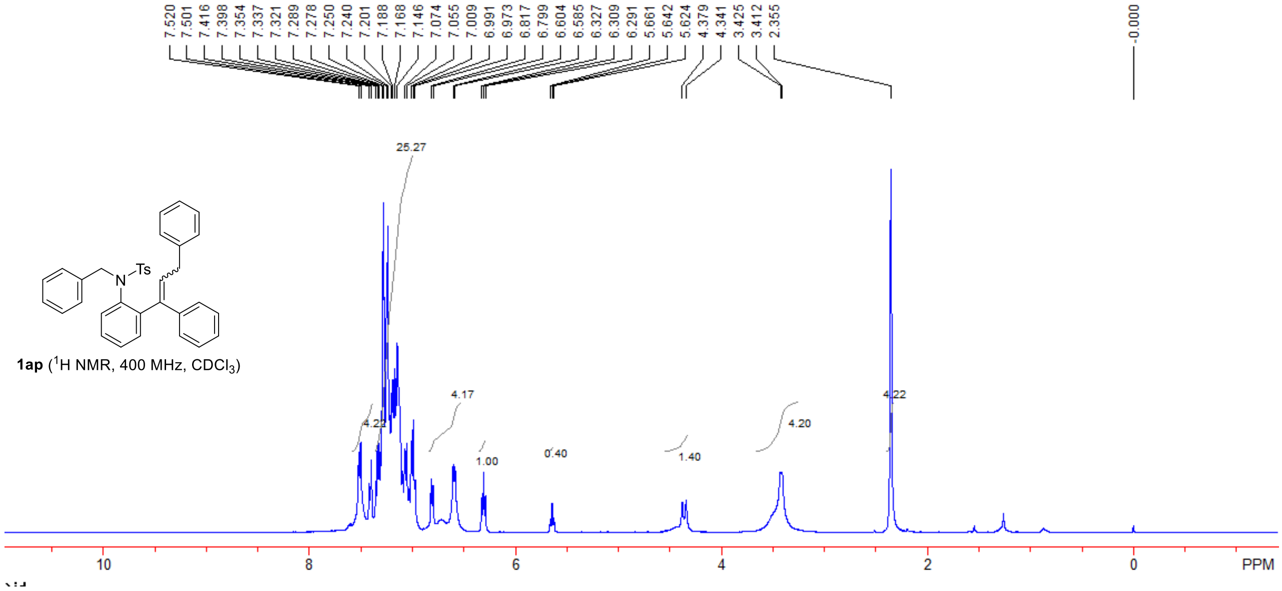
7.520 7.501 7.416 7.398 7.354 7.337 7.321 7.289 7.278 7.250 7.240 7.201 7.188 7.168 7.146 7.074 7.055 7.009 6.991 6.973 6.817 6.799 6.604 6.585 6.327 6.309 6.291 5.661 5.642 5.624 4.379 4.341 3.425 3.412 2.355
-0.000
25.27
4.22
4.17
1.00
0.40
1.40
4.20
4.22
Ts
N
1ap (^{1}H NMR, 400 MHz, $CDCl_3$)
10
8
6
4
2
0
PPM

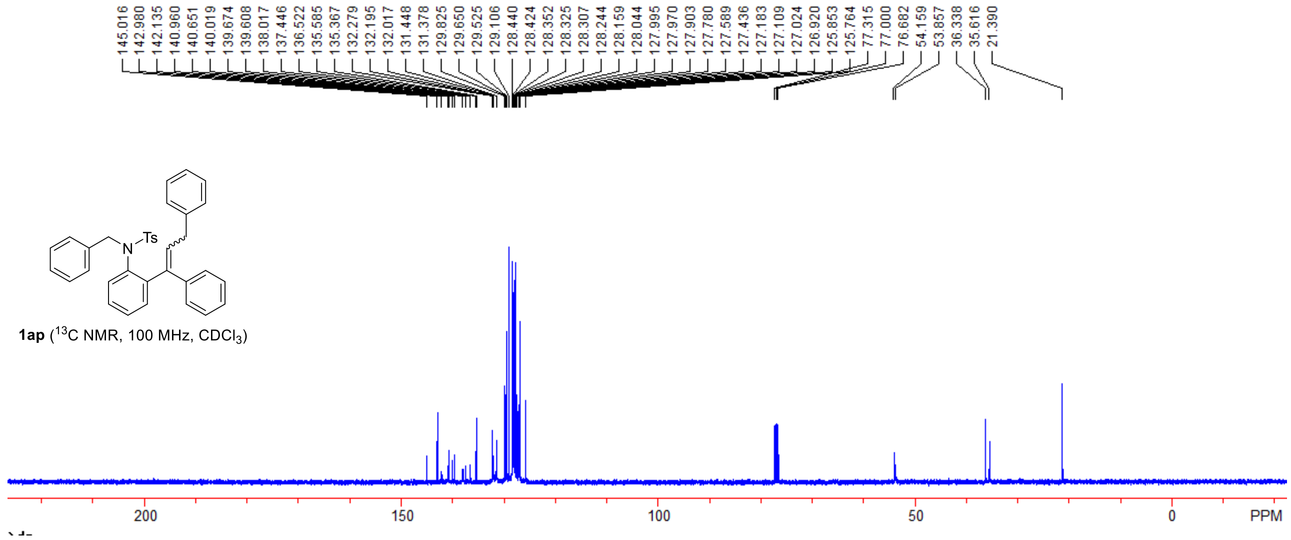
145.016 142.980 142.135 140.960 140.651 140.019 139.674 139.608 138.017 137.446 136.522 135.585 135.367 132.279 132.195 132.017 131.448 131.378 129.825 129.650 129.525 129.106 128.440 128.424 128.352 128.325 128.307 128.244 128.159 128.044 127.995 127.970 127.903 127.780 127.589 127.436 127.183 127.109 127.024 126.920 125.853 125.764 77.315 77.000 76.682 54.159 53.857 36.338 35.616 21.390
Ts
N
1ap (^{13}C NMR, 100 MHz, $CDCl_3$)
200
150
100
50
0
PPM

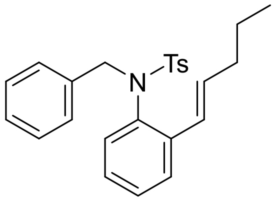

Compound 1aq: Yield: 2362 mg, 92%; a light yellow solid; Mp: 100-102 °C; Eluent: PE/EA = 5/1. ^{1}H NMR (400 MHz, $CDCl_3$, TMS) δ 0.89 (t, *J* = 7.2 Hz, 3H), 1.28-1.40 (m, 2H), 1.99 (q, *J* = 6.8 Hz, 2H), 2.37 (s, 3H), 4.48 (d, *J* = 13.2 Hz, 1H), 4.79 (d, *J* = 13.2 Hz, 1H), 5.92-6.03 (m, 1H), 6.30 (d, *J* = 16.4 Hz, 1H), 6.67 (d, *J* = 8.0 Hz, 1H), 6.97 (t, *J* = 7.6 Hz, 1H), 7.03-7.16 (m, 6H), 7.22 (d, *J* = 8.0 Hz, 2H), 7.39 (d, *J* = 8.0 Hz, 1H), 7.59 (d, *J* = 7.6 Hz, 2H); $^{13}C\{^{1}H\}$-NMR (101 MHz, $CDCl_3$, TMS) δ_C 13.6, 21.3, 22.0, 35.0, 55.6, 125.3, 125.8, 126.7, 127.47, 127.53, 127.9, 128.2, 129.0, 129.3, 129.6, 132.3, 135.4, 135.6, 136.4, 138.3, 143.2; IR (neat): ν 3063, 3033, 2958, 2923, 2870, 1596, 1483, 1453, 1338, 1181, 1155, 1117, 1088, 1027, 964, 861, 765, 714 cm^{-1}; HRMS (ESI-TOF) Calcd for $C_{25}H_{27}NO_2NaS$ $[M+Na]^+$: 428.1655, found: 428.1652.

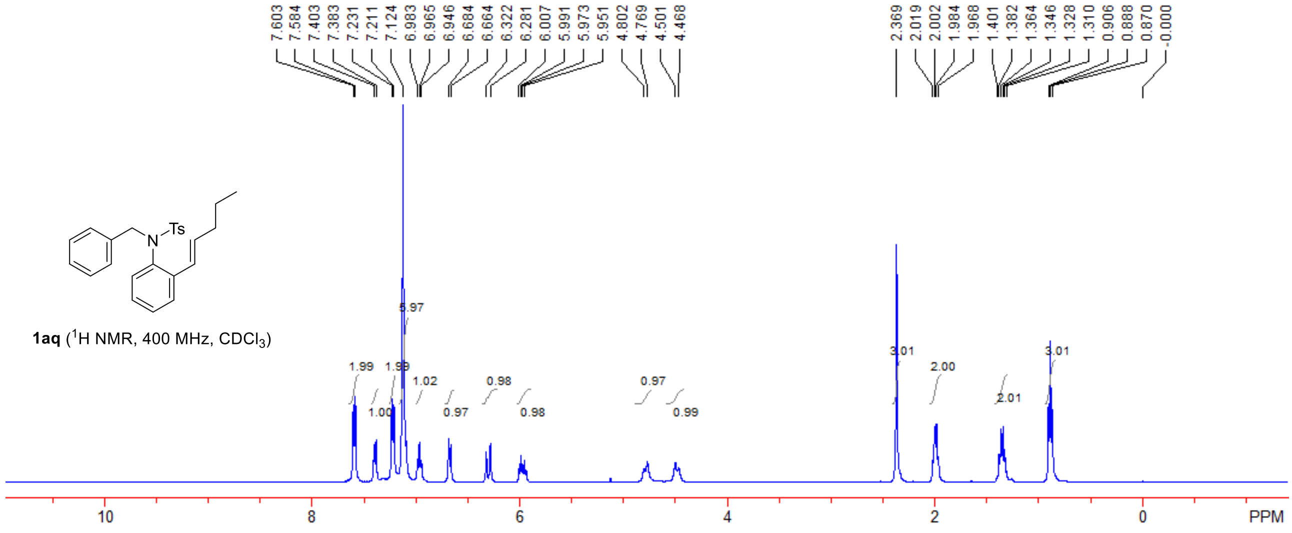

1aq (^{1}H NMR, 400 MHz, $CDCl_3$)

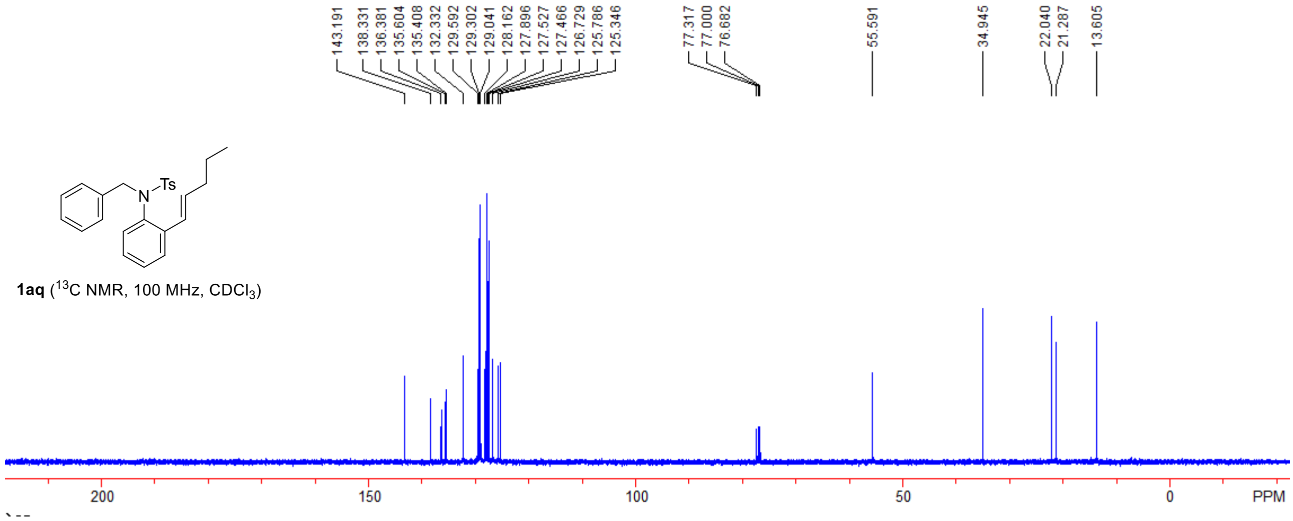

1aq (^{13}C NMR, 100 MHz, $CDCl_3$)

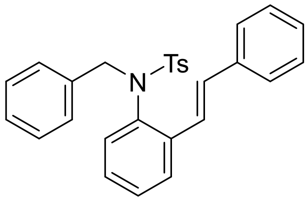

Compound 1ar: Yield: 2043 mg, 81%; a light yellow solid; Mp: 120-122 °C; Eluent: PE/EA = 5/1. ^{1}H NMR (400 MHz, $CDCl_3$, TMS) δ 2.31 (s, 3H), 4.54 (d, *J* = 13.6 Hz, 1H), 4.84 (d, *J* = 14.0 Hz, 1H), 6.74-6.86 (m, 2H), 6.90 (d, *J* = 16.0 Hz, 1H), 7.04-7.32 (m, 14H), 7.55 (d, *J* = 8.0 Hz, 1H), 7.63 (d, *J* = 8.0 Hz, 2H); ^{13}C{^{1}H}-NMR (101 MHz, $CDCl_3$, TMS) δ_C 21.4, 55.9, 124.1, 125.8, 126.6, 127.55, 127.57, 127.70, 127.71, 128.1, 128.3, 128.5, 129.1, 129.5, 129.9, 130.4, 135.4, 136.5, 136.6, 137.0, 137.7, 143.5; IR (neat): ν 3057, 3026, 2918, 1596, 1494, 1452, 1400, 1369, 1306, 1257, 1186, 1120, 1026, 980, 860, 776, 714cm^{-1}; HRMS (ESI-TOF) Calcd for $C_{28}H_{25}NO_2NaS$ $[M+Na]^+$: 462.1498, found: 462.1494.

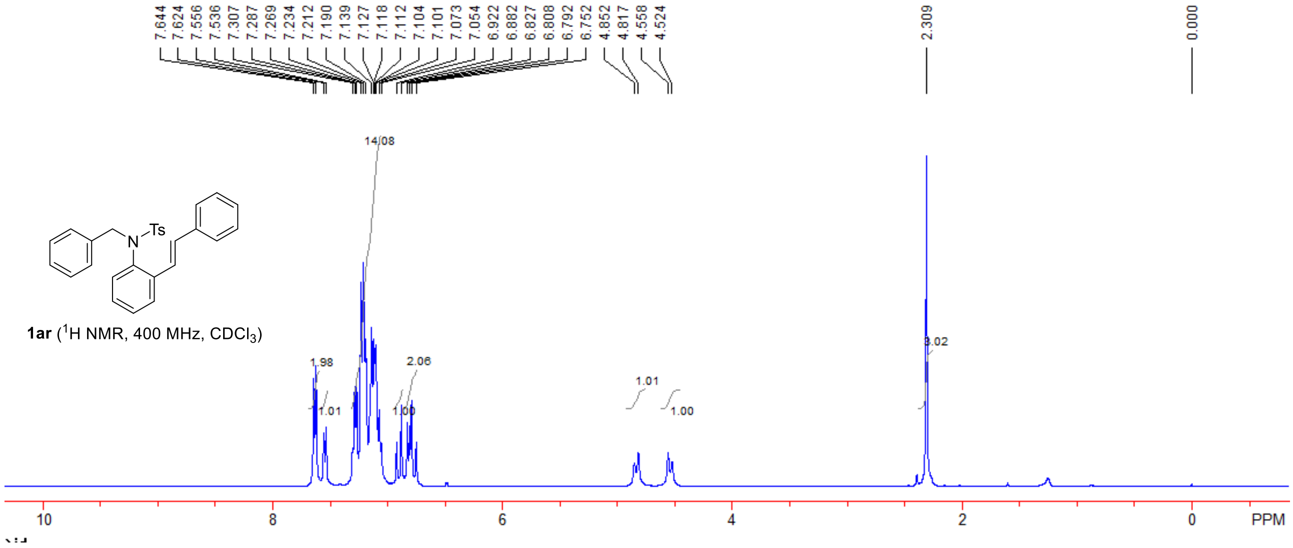
7.644 7.624 7.556 7.536 7.307 7.287 7.269 7.234 7.212 7.190 7.139 7.127 7.118 7.112 7.104 7.101 7.073 7.054 6.922 6.882 6.827 6.808 6.792 6.752 4.852 4.817 4.558 4.524
2.309
0.000
14.08
1.98
1.01
2.06
1.00
1.01
1.00
3.02
N
Ts
1ar (1H NMR, 400 MHz, CDCl3)
10
8
6
4
2
0
PPM

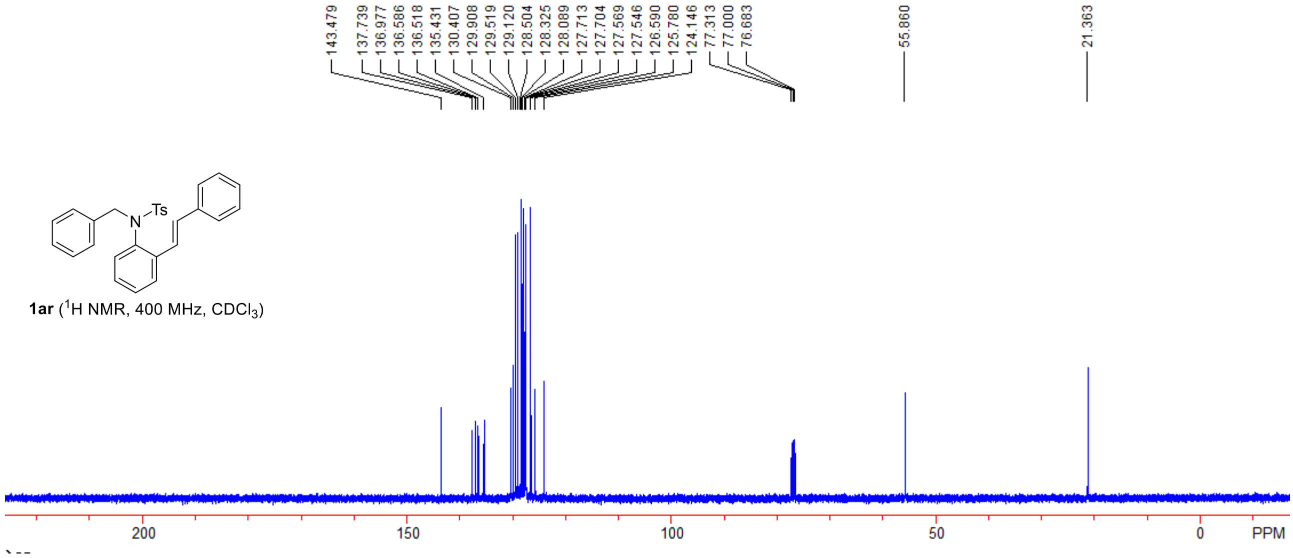
143.479 137.739 136.977 136.586 136.518 135.431 130.407 129.908 129.519 129.120 128.504 128.325 128.089 127.713 127.704 127.569 127.546 126.590 125.780 124.146 77.313 77.000 76.683
55.860
21.363
N
Ts
1ar (1H NMR, 400 MHz, CDCl3)
200
150
100
50
0
PPM

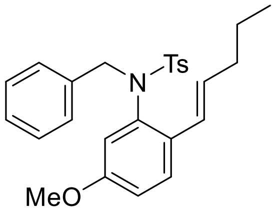

Compound 1as: Yield: 2482 mg, 90%; a yellow oil; Eluent: PE/EA = 5/1. ^{1}H NMR (400 MHz, $CDCl_3$, TMS) δ 0.89 (t, J = 7.6 Hz, 3H), 1.28-1.40 (m, 2H), 1.96 (q, J = 7.2 Hz, 2H), 2.39 (s, 3H), 3.52 (s, 3H), 4.45-4.85 (m, 2H), 5.81-5.92 (m, 1H), 6.10-6.25 (m, 2H), 6.72 (dd, J_1 = 8.8 Hz, J_2 = 2.4 Hz, 1H), 7.10-7.20 (m, 5H), 7.25 (d, J = 8.0 Hz, 2H), 7.32 (d, J = 8.8 Hz, 1H), 7.63 (d, J = 8.0 Hz, 2H); ^{13}C{^{1}H}-NMR (101 MHz, $CDCl_3$, TMS) δ_C 13.7, 21.4, 22.3, 35.0, 55.0, 55.6, 114.6, 114.9, 124.8, 126.6, 127.6, 127.7, 128.0, 129.2, 129.4, 130.4, 131.0, 135.5, 136.4, 136.6, 143.3, 158.1; IR (neat): ν 3030, 2956, 2926, 2869, 2836, 1605, 1568, 1494, 1455, 1345, 1303, 1248, 1159, 1091, 1035, 966, 812, 754, 700 cm^{-1}; HRMS (ESI-TOF) Calcd for $C_{26}H_{29}NO_3NaS$ $[M+Na]^+$: 458.1760, found: 458.1758.

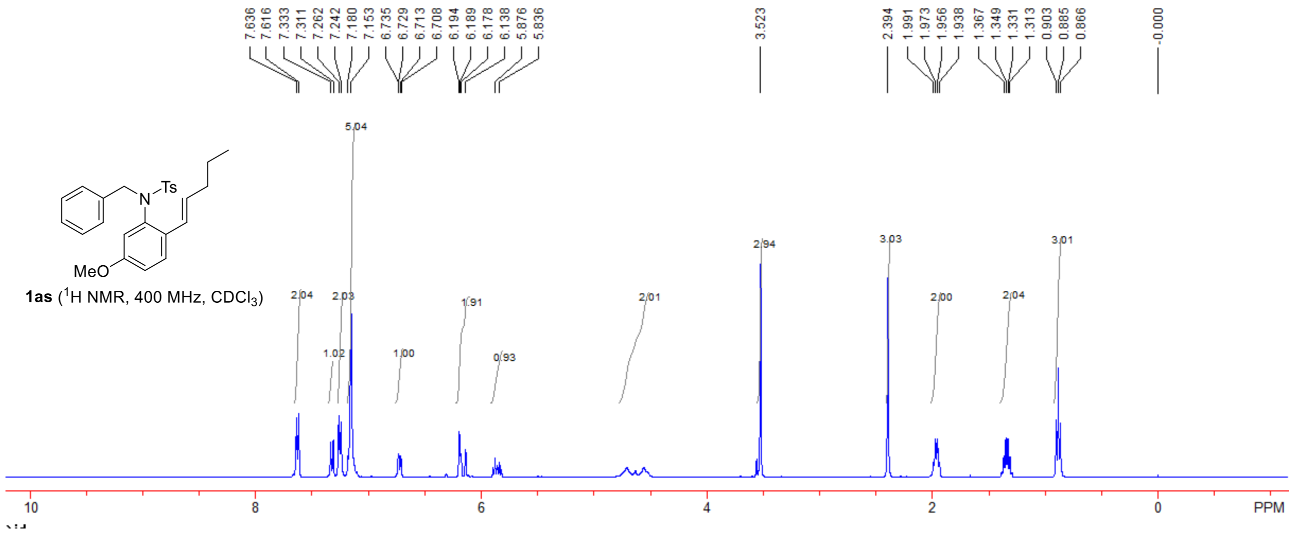

7.636 7.616 7.333 7.311 7.262 7.242 7.180 7.153 6.735 6.729 6.713 6.708 6.194 6.189 6.178 6.138 5.876 5.836
3.523
2.394 1.991 1.973 1.956 1.938 1.367 1.349 1.331 1.313 0.903 0.885 0.866
-0.000
Ts
N
MeO
1as (1H NMR, 400 MHz, CDCl3)
5.04
2.04 2.03 1.02 1.00 1.91 0.93 2.01 2.94 3.03 2.00 2.04 3.01
10 8 6 4 2 0 PPM

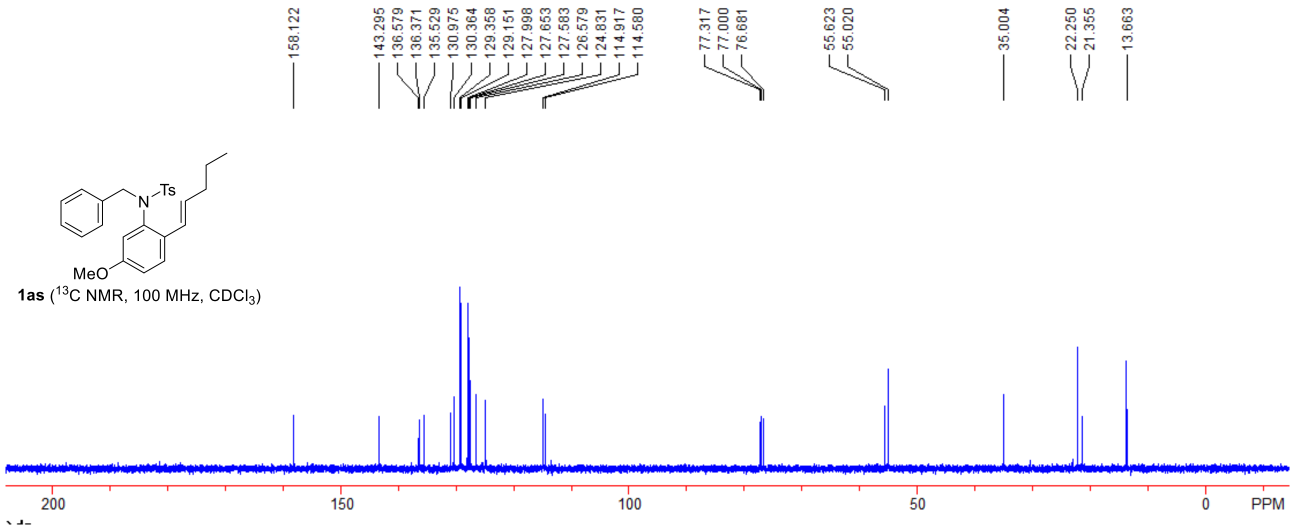

158.122
143.295 136.579 136.371 135.529 130.975 130.364 129.358 129.151 127.998 127.653 127.583 126.579 124.831 114.917 114.580
77.317 77.000 76.681
55.623 55.020
35.004
22.250 21.355
13.663
Ts
N
MeO
1as (13C NMR, 100 MHz, CDCl3)
200 150 100 50 0 PPM

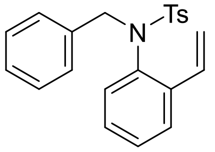

Compound 1at: Yield: 1826 mg, 77%; a light red solid; Mp: 73-75 °C; Eluent: PE/EA = 10/1. ^{1}H NMR (400 MHz, $CDCl_3$, TMS) δ 2.34 (s, 3H), 4.37 (d, *J* = 12.4 Hz, 1H), 4.88 (d, *J* = 12.8 Hz, 1H), 5.06 (d, *J* = 10.8 Hz, 1H), 5.49 (d, *J* = 17.6 Hz, 1H), 6.65 (d, *J* = 8.0 Hz, 1H), 6.76-6.86 (m, 1H), 7.02 (t, *J* = 7.6 Hz, 1H), 7.08-7.16 (m, 6H), 7.21 (d, *J* = 8.0 Hz, 2H), 7.44 (d, *J* = 7.6 Hz, 1H), 7.57 (d, *J* = 8.0 Hz, 2H); ^{13}C{^{1}H}-NMR (101 MHz, $CDCl_3$, TMS) δ_C 21.2, 55.6, 115.0, 125.6, 127.45, 127.49, 127.7, 127.9, 128.1, 128.7, 128.9, 129.3, 132.3, 135.0, 135.7, 136.2, 138.2, 143.3; IR (neat): ν 3029, 1628, 1597, 1484, 1411, 1373, 1289, 1186, 1107, 1052, 995, 915, 813, 770, 709 cm^{-1}; HRMS (ESI-TOF) Calcd for $C_{22}H_{21}NO_2NaS$ [M+Na]$^+$: 386.1185, found: 386.1188.

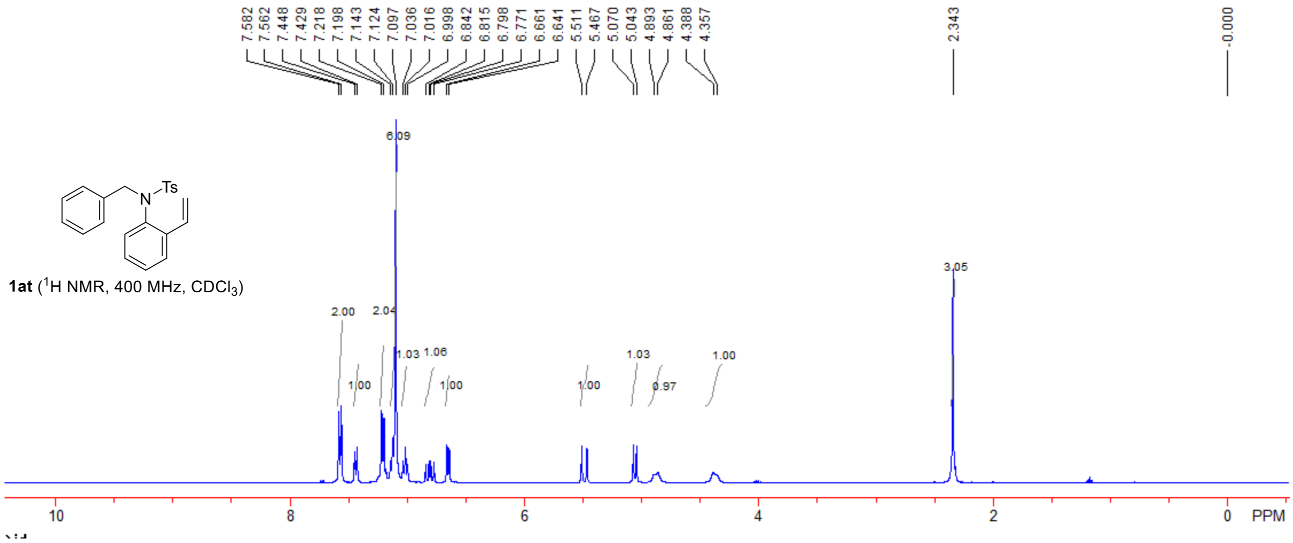

1at (^{1}H NMR, 400 MHz, $CDCl_3$)

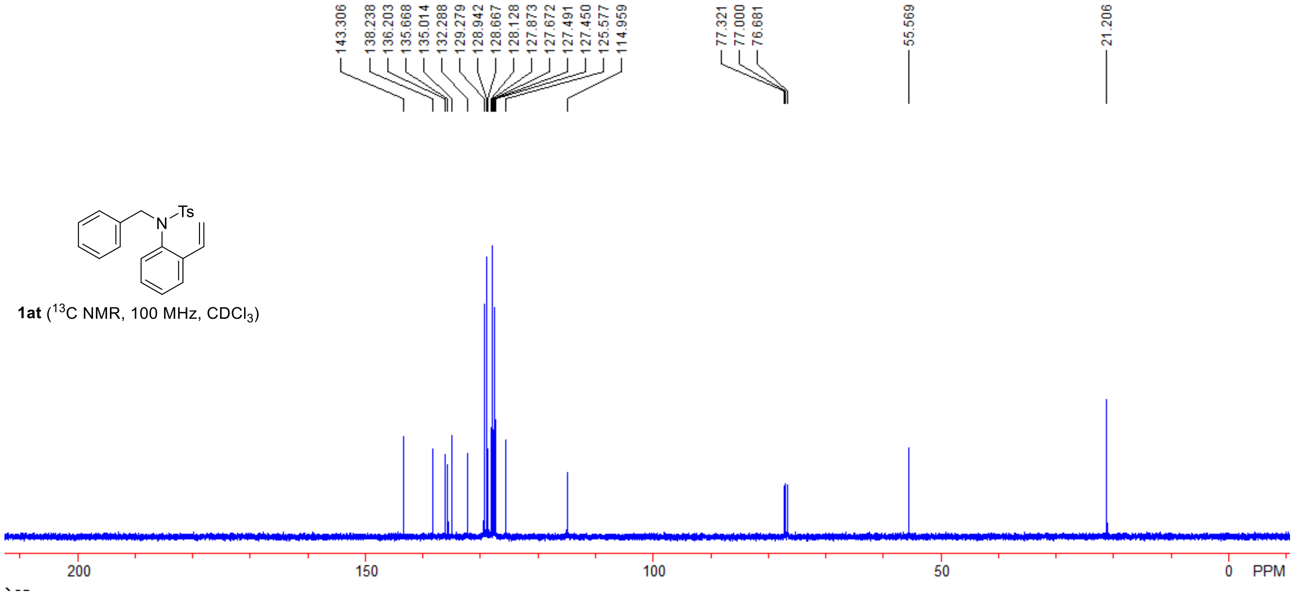

1at (^{13}C NMR, 100 MHz, $CDCl_3$)

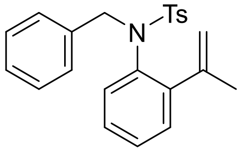

Compound 1au: Yield: 1992 mg, 90%; a light yellow solid; Mp: 80-82 °C; Eluent: PE/EA = 10/1. ^{1}H NMR (400 MHz, $CDCl_3$, TMS) δ 1.87 (s, 3H), 2.35 (s, 3H), 4.30-4.90 (m, 3H), 5.02 (s, 1H), 6.77 (d, *J* = 7.6 Hz, 1H), 6.98 (d, *J* = 7.6 Hz, 2H), 7.02-7.14 (m, 6H), 7.22 (d, *J* = 7.6 Hz, 2H), 7.63 (d, *J* = 7.6 Hz, 2H); ^{13}C{^{1}H}-NMR (101 MHz, $CDCl_3$, TMS) δ_C 21.2, 23.9, 55.4, 116.2, 127.0, 127.5, 127.78, 127.82, 128.4, 129.2, 129.4, 129.6, 134.9, 136.1, 136.5, 142.5, 143.2, 144.6; IR (neat): ν 2978, 1642, 1597, 1489, 1438, 1366, 1343, 1200, 1182, 1157, 1089, 952, 858, 782, 703 cm^{-1}; HRMS (ESI-TOF) Calcd for $C_{23}H_{23}NO_2NaS$ [M+Na]$^+$: 400.1342, found: 400.1340.

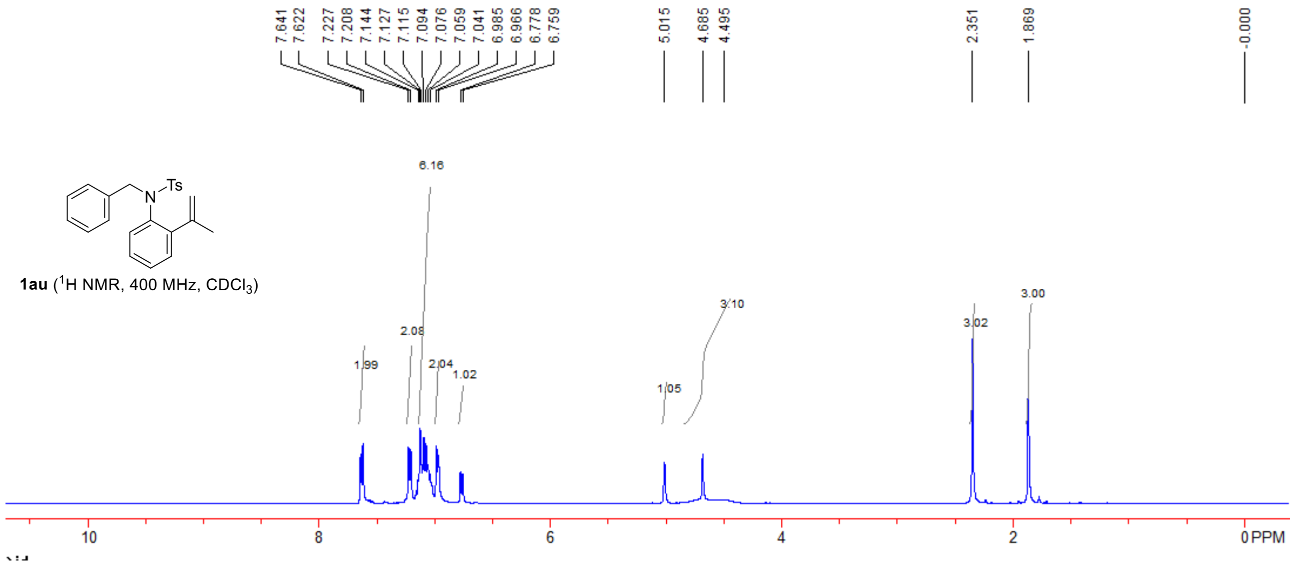

1au (^{1}H NMR, 400 MHz, $CDCl_3$)

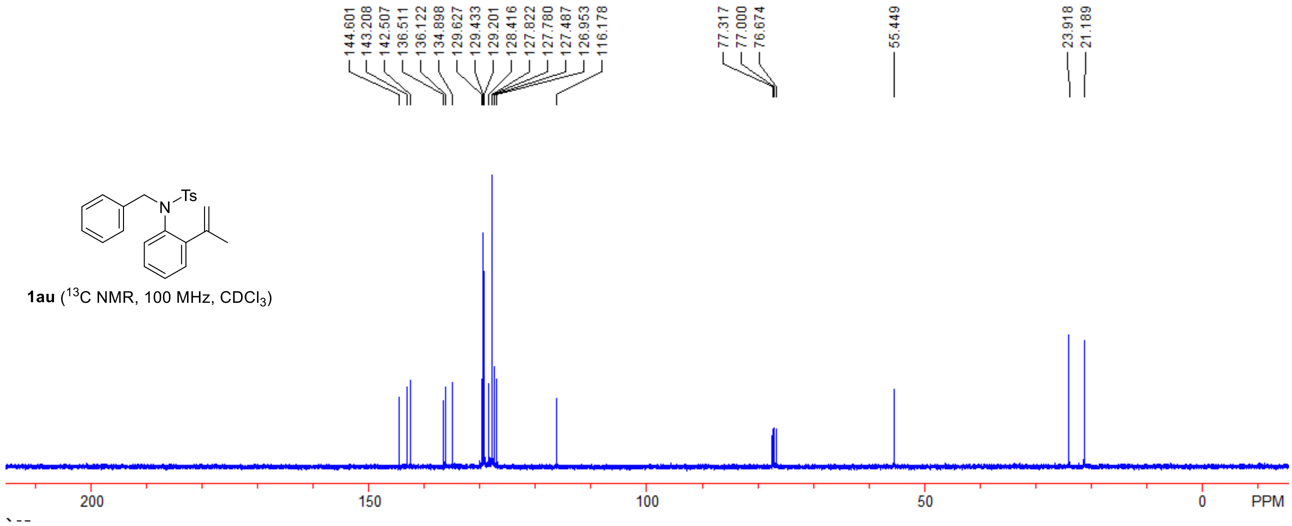

1au (^{13}C NMR, 100 MHz, $CDCl_3$)

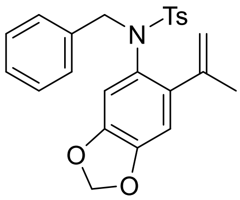

Compound 1av: Yield: 1076 mg, 80%; a light yellow solid; Mp: 158-160 ^{o}C; Eluent: PE/EA = 5/1. ^{1}H NMR (400 MHz, $CDCl_3$, TMS) δ 1.80 (s, 3H), 2.42 (s, 3H), 4.38 (d, *J* = 12.8 Hz, 1H), 4.65 (s, 1H), 4.71 (d, *J* = 13.2 Hz, 1H), 4.98 (s, 1H), 5.88 (s, 2H), 6.25 (s, 1H), 6.58 (s, 1H), 6.99 (d, *J* = 6.4 Hz, 2H), 7.10-7.20 (m, 3H), 7.28 (d, *J* = 7.6 Hz, 2H), 7.69 (d, *J* = 8.0 Hz, 2H); ^{13}C{^{1}H}-NMR (101 MHz, $CDCl_3$, TMS) δ_C 21.4, 24.1, 55.7, 101.6, 108.6, 109.0, 116.4, 127.7, 128.1, 129.2, 129.5, 129.7, 135.0, 136.8, 139.2, 142.4, 143.5, 146.2, 146.9; IR (neat): ν 3029, 2970, 2921, 1595, 1507, 1489, 1452, 1377, 1329, 1279, 1242, 1205, 1152, 1082, 1028, 966, 873, 819, 719, 697 cm^{-1}; HRMS (ESI-TOF) Calcd for $C_{24}H_{23}NO_4NaS$ $[M+Na]^+$: 444.1240, found: 444.1248.

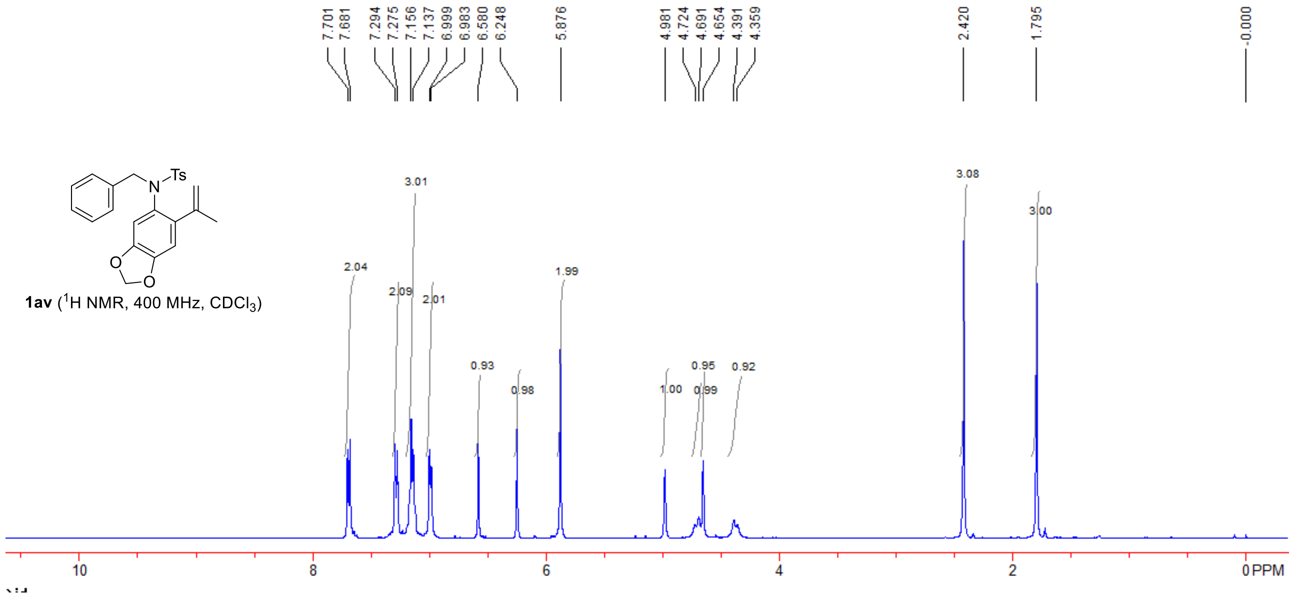
7.701
7.681
7.294
7.275
7.156
7.137
6.999
6.983
6.580
6.248
5.876
4.981
4.724
4.691
4.654
4.391
4.359
2.420
1.795
-0.000
Ts
N
O
O
1av (¹H NMR, 400 MHz, CDCl₃)
2.04
2.09
3.01
2.01
0.93
0.98
1.99
1.00
0.95
0.99
0.92
3.08
3.00
10
8
6
4
2
0 PPM

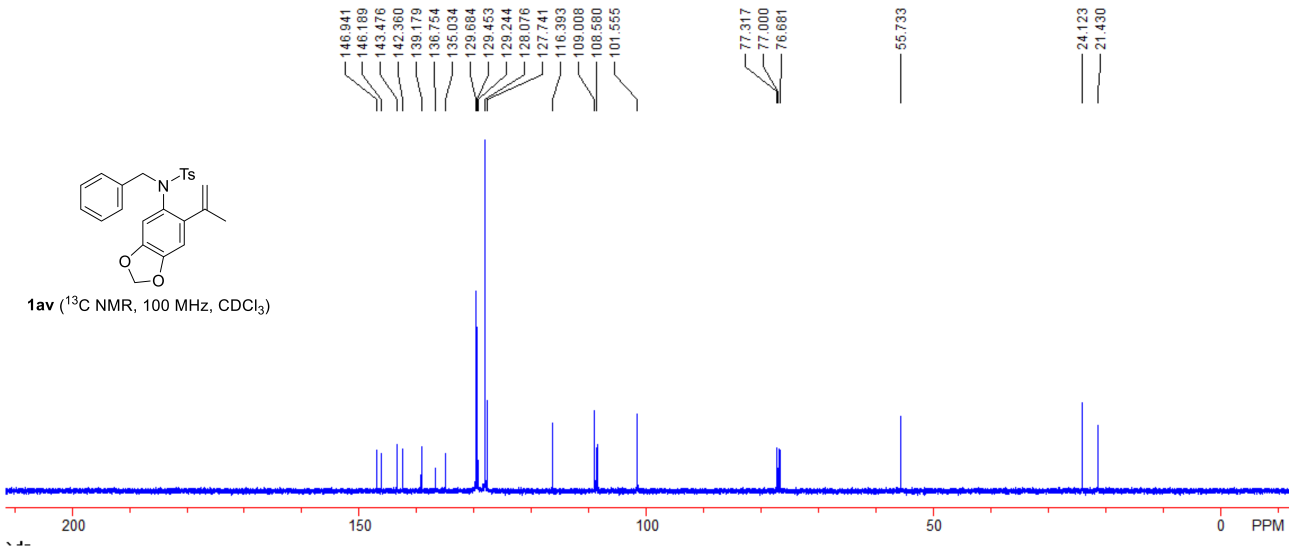
146.941
146.189
143.476
142.360
139.179
136.754
135.034
129.684
129.453
129.244
128.076
127.741
116.393
109.008
108.580
101.555
77.317
77.000
76.681
55.733
24.123
21.430
Ts
N
O
O
1av (¹³C NMR, 100 MHz, CDCl₃)
200
150
100
50
0
PPM

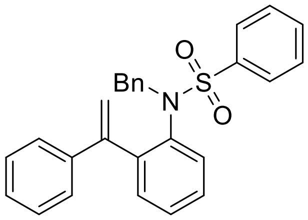

Compound 1aw: Yield: 1957 mg, 87%; a light yellow solid; Mp: 98-100 ^{o}C; Eluent: PE/EA = 10/1. ^{1}H NMR (400 MHz, $CDCl_3$, TMS) δ 4.00-4.60 (m, 2H), 5.08 (s, 1H), 5.61 (s, 1H), 6.84 (d, *J* = 6.8 Hz, 2H), 6.93 (d, *J* = 8.0 Hz, 1H), 7.00-7.13 (m, 4H), 7.15-7.26 (m, 7H), 7.32 (d, *J* = 8.0 Hz, 2H), 7.44 (d, *J* = 7.2 Hz, 1H), 7.62 (d, *J* = 7.2 Hz, 2H); ^{13}C{^{1}H}-NMR (101 MHz, $CDCl_3$, TMS) δ_C 54.5, 117.1, 126.8, 127.5, 127.7, 127.80, 127.90, 127.93, 128.1, 128.5, 129.2, 130.9, 131.9, 132.4, 135.2, 137.0, 140.2, 140.7, 142.2, 145.8; IR (neat): ν 3060, 2987, 2926, 1614, 1487, 1446, 1355, 1321, 1273, 1211, 1176, 1103, 1088, 1047, 994, 880, 807, 784, 712 cm^{-1}; HRMS (ESI-TOF) Calcd for $C_{27}H_{23}NO_2NaS$ $[M+Na]^+$: 448.1342, found: 448.1350.

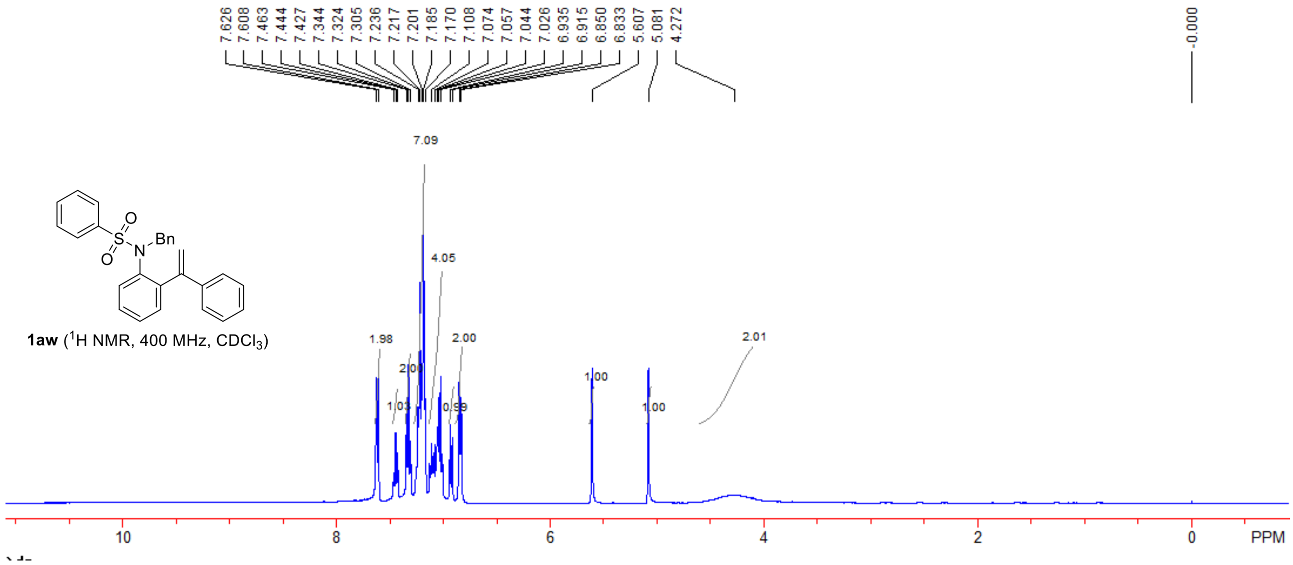

1aw (^{1}H NMR, 400 MHz, $CDCl_3$)

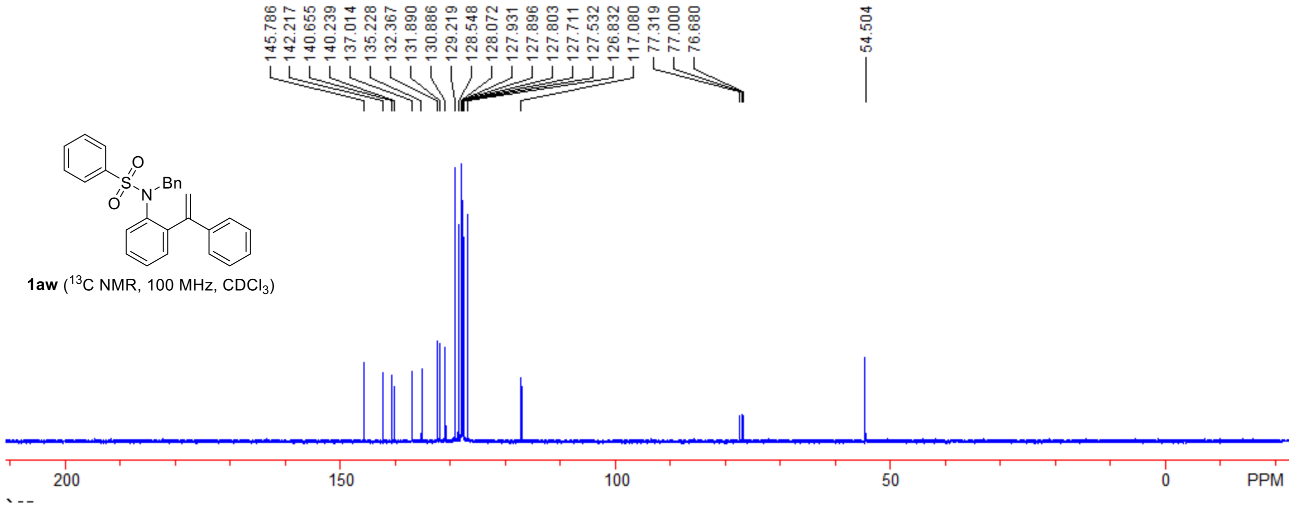

1aw (^{13}C NMR, 100 MHz, $CDCl_3$)

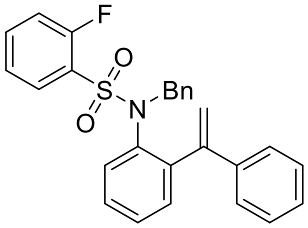

Compound 1ax: Yield: 1879 mg, 90%; a yellow oil; Eluent: PE/EA = 10/1. ^{1}H NMR (400 MHz, $CDCl_3$, TMS) δ 4.00-5.00 (m, 2H), 4.73 (s, 1H), 5.43 (s, 1H), 6.87 (d, *J* = 7.6 Hz, 1H), 6.92-7.00 (m, 2H), 7.03-7.32 (m, 13H), 7.52 (q, *J* = 6.4 Hz, 1H), 7.67 (t, *J* = 7.6 Hz, 1H); ^{13}C{^{1}H}-NMR (101 MHz, $CDCl_3$, TMS) δ_C 54.3 (d, *J* = 3.6 Hz), 116.8, 117.0 (d, *J* = 21.7 Hz), 124.0 (d, *J* = 3.8 Hz), 127.68, 127.72 (d, *J* = 20.6 Hz), 128.1, 128.20, 128.24, 129.3, 131.1, 131.8, 133.4, 134.7 (d, *J* = 8.4 Hz), 135.85, 135.90, 140.4, 142.1, 145.8, 159.1 (d, *J* = 254.8 Hz); ^{19}F NMR (376 MHz, $CDCl_3$) δ -106.0; IR (neat): ν 3059, 3028, 2917, 1617, 1597, 1474, 1447, 1345, 1264, 1211, 1153, 1123, 1073, 1027, 952, 827, 733 cm^{-1}; HRMS (ESI-TOF) Calcd for $C_{27}H_{22}NO_2FNaS$ $[M+Na]^+$: 466.1248, found: 466.1256.

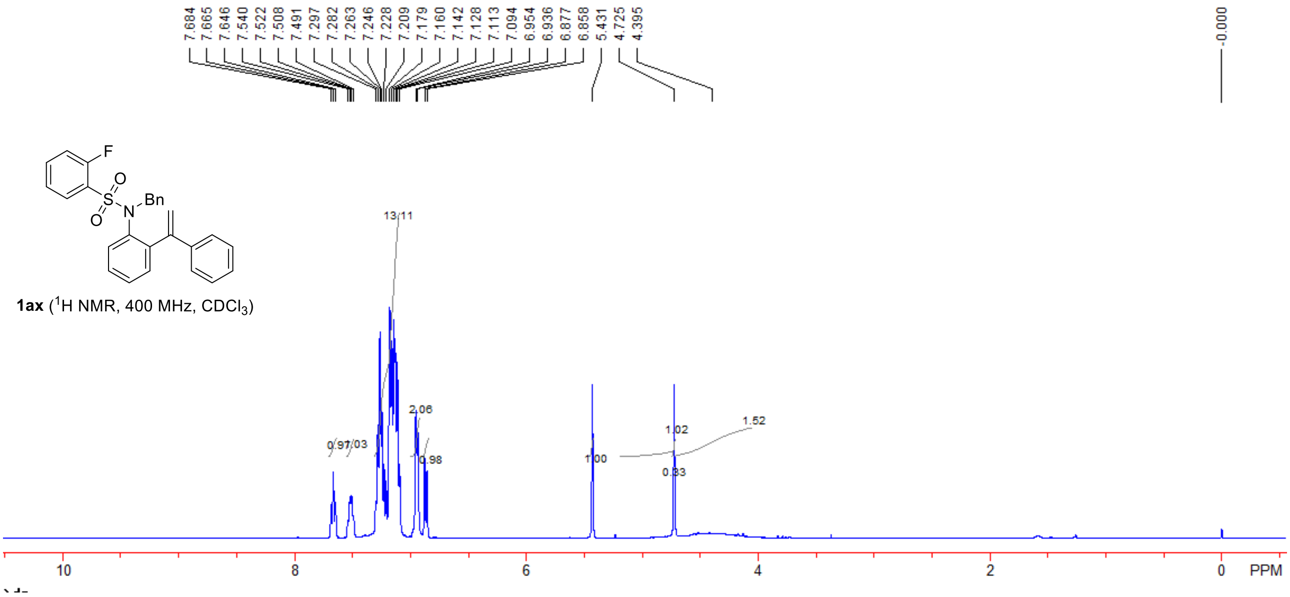

1ax (^{1}H NMR, 400 MHz, $CDCl_3$)

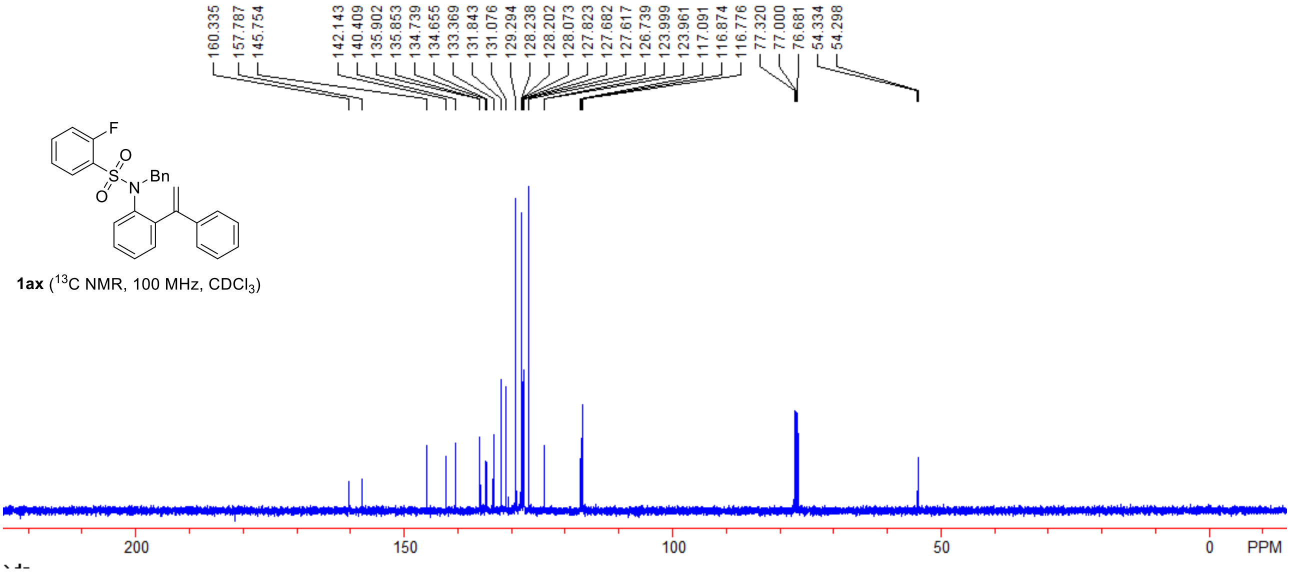

1ax (^{13}C NMR, 100 MHz, $CDCl_3$)

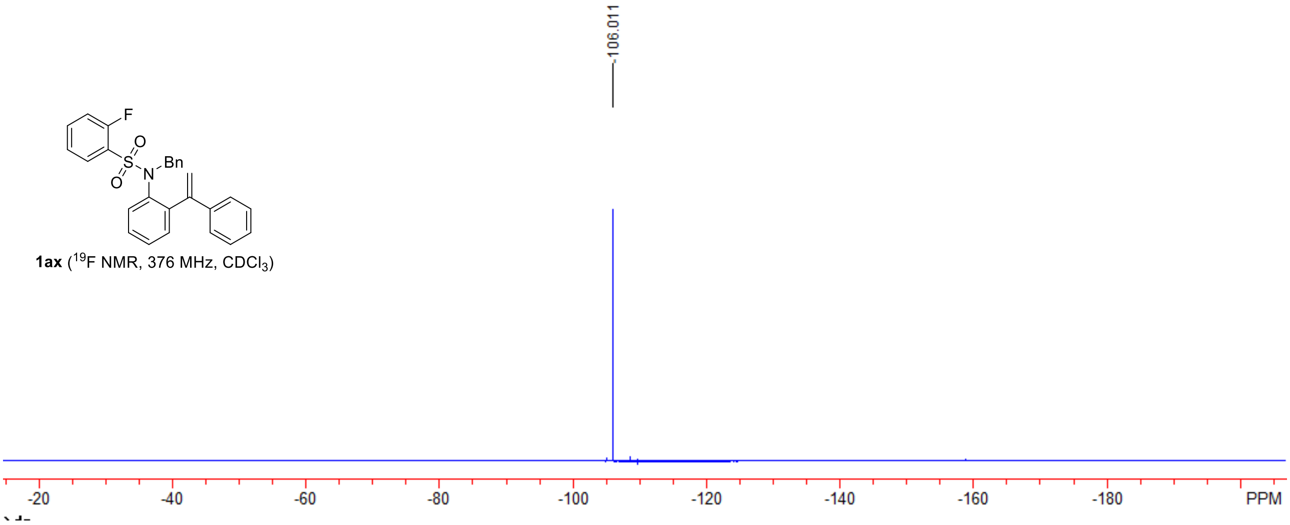

1ax (^{19}F NMR, 376 MHz, $CDCl_3$)

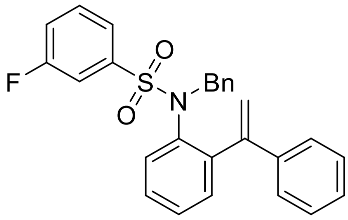

Compound 1ay: Yield: 1702 mg, 80%; a yellow oil; Eluent: PE/EA = 10/1. ^{1}H NMR (400 MHz, $CDCl_3$, TMS) δ 3.80-4.60 (m, 2H), 5.10 (s, 1H), 5.64 (s, 1H), 6.85 (d, *J* = 7.2 Hz, 2H), 6.92 (d, *J* = 8.0 Hz, 1H), 7.02-7.30 (m, 14H), 7.38 (d, *J* = 7.6 Hz, 1H); ^{13}C{^{1}H}-NMR (101 MHz, $CDCl_3$, TMS) δ_C 54.8, 115.1 (d, *J* = 24.3 Hz), 117.1, 119.4 (d, *J* = 21.0 Hz), 123.6 (d, *J* = 3.3 Hz), 126.8, 127.70, 127.72, 127.9, 128.0, 128.17, 128.21, 129.3, 130.2 (d, *J* = 7.6 Hz), 130.9, 132.0, 135.0, 136.9, 140.5, 142.2, 142.4 (d, *J* = 6.2 Hz), 146.0, 161.9 (d, *J* = 249.3 Hz); ^{19}F NMR (376 MHz, $CDCl_3$) δ -109.9; IR (neat): ν 3063, 3029, 2918, 1589, 1494, 1433, 1348, 1269, 1222, 1150, 1083, 910, 862, 766, 697 cm^{-1}; HRMS (ESI-TOF) Calcd for $C_{27}H_{22}NO_2FNaS$ $[M+Na]^+$: 466.1248, found: 466.1257.

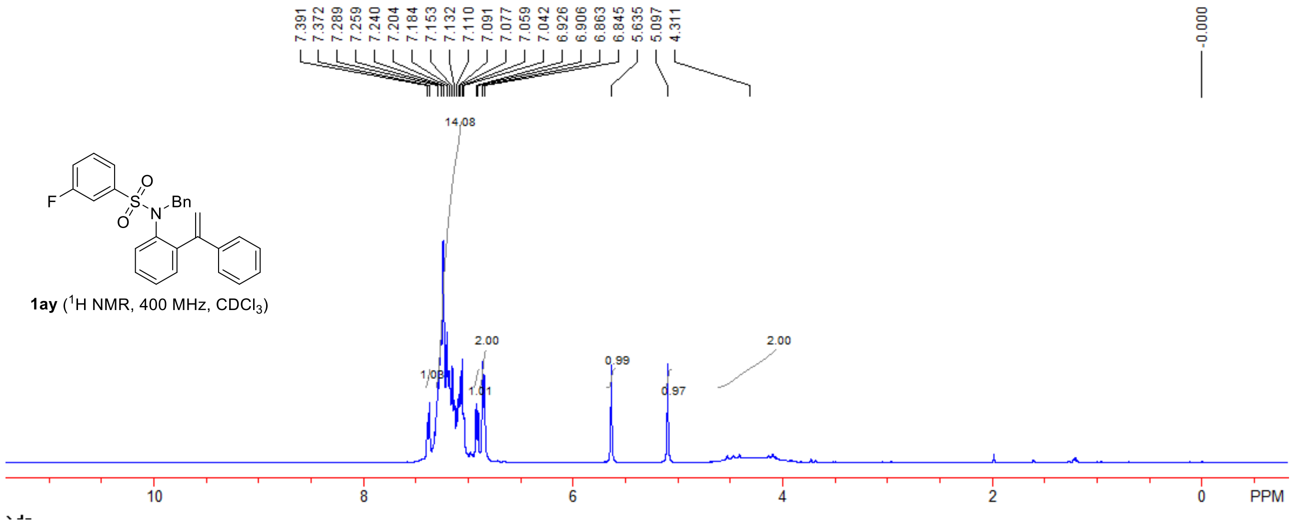

7.391 7.372 7.289 7.259 7.240 7.204 7.184 7.153 7.132 7.110 7.091 7.077 7.059 7.042 6.926 6.906 6.863 6.845 5.635 5.097 4.311
-0.000
14.08
1.08 2.00 1.01 0.99 0.97 2.00
F
O
S
O
N
Bn
1ay (¹H NMR, 400 MHz, CDCl₃)
10 8 6 4 2 0 PPM

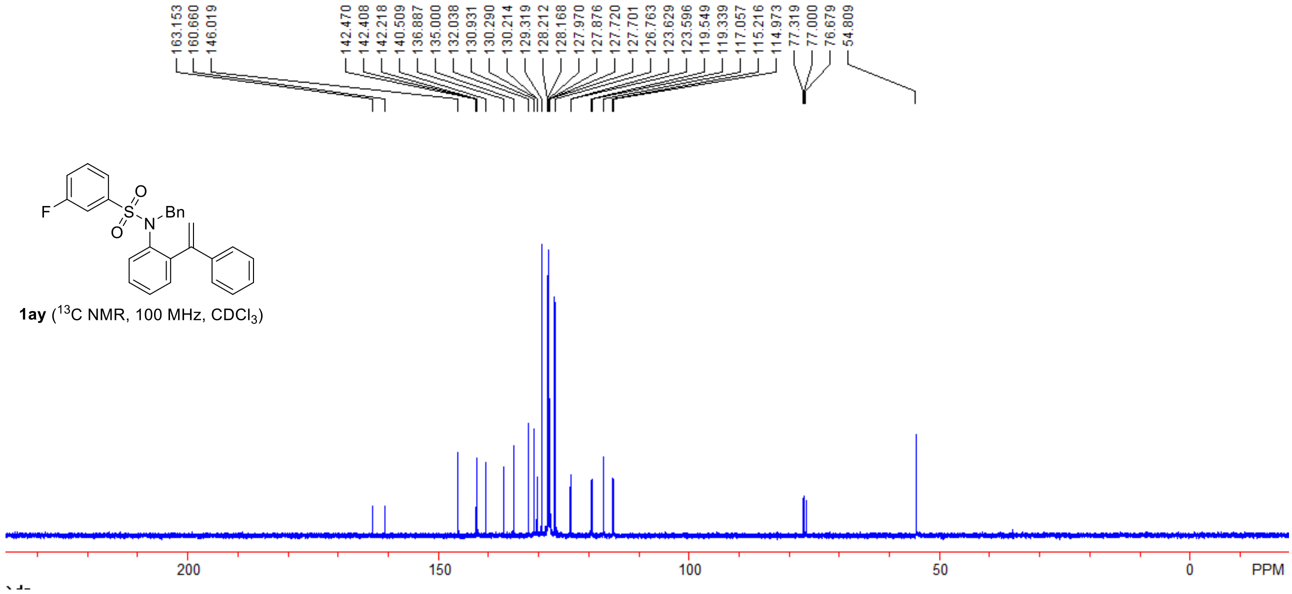

163.153 160.660 146.019 142.470 142.408 142.218 140.509 136.887 135.000 132.038 130.931 130.290 130.214 129.319 128.212 128.168 127.970 127.876 127.720 127.701 126.763 123.629 123.596 119.549 119.339 117.057 115.216 114.973 77.319 77.000 76.679 54.809
F
O
S
O
N
Bn
1ay (¹³C NMR, 100 MHz, CDCl₃)
200 150 100 50 0 PPM

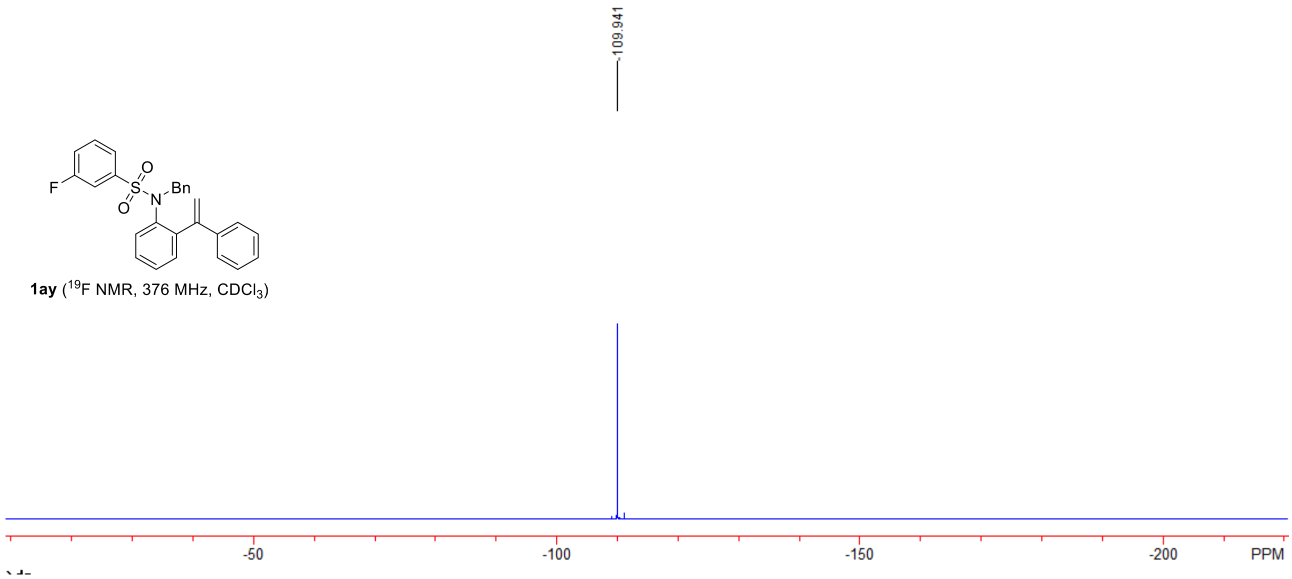

-109.941
F
O
S
O
N
Bn
1ay (¹⁹F NMR, 376 MHz, CDCl₃)
-50 -100 -150 -200 PPM

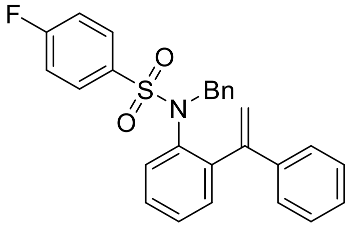

Compound 1az: Yield: 1935 mg, 92%; a yellow oil; Eluent: PE/EA = 10/1. ^{1}H NMR (400 MHz, $CDCl_3$, TMS) δ 3.90-4.60 (m, 2H), 5.09 (s, 1H), 5.64 (s, 1H), 6.86 (d, *J* = 7.2 Hz, 2H), 6.94 (d, *J* = 7.6 Hz, 1H), 7.00 (t, *J* = 8.4 Hz, 2H), 7.03-7.30 (m, 11H), 7.50-7.57 (m, 2H); ^{13}C{^{1}H}-NMR (101 MHz, $CDCl_3$, TMS) δ_C 54.8, 115.6 (d, *J* = 22.4 Hz), 117.0, 126.8, 127.65, 127.66, 127.9, 128.0, 128.13, 128.18, 129.3, 130.6 (d, *J* = 9.3 Hz), 130.8, 132.1, 136.4, (d, *J* = 2.9 Hz), 137.1, 140.6, 142.1, 146.6, 164.7 (d, *J* = 253.6 Hz); ^{19}F NMR (376 MHz, $CDCl_3$) δ -105.4; IR (neat): ν 3062, 3029, 2930, 1590, 1491, 1404, 1344, 1290, 1233, 1151, 1089, 1027, 910, 835, 766, 696 cm^{-1}; HRMS (ESI-TOF) Calcd for $C_{27}H_{22}NO_2FNaS$ $[M+Na]^+$: 466.1248, found: 466.1238.

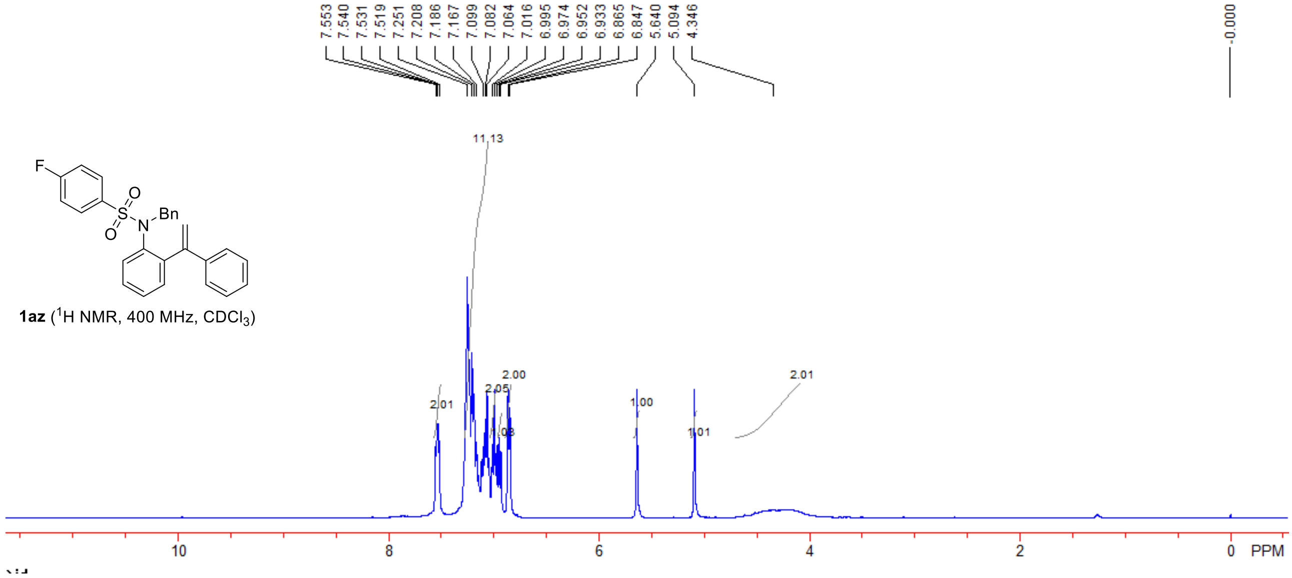

1az (^{1}H NMR, 400 MHz, $CDCl_3$)

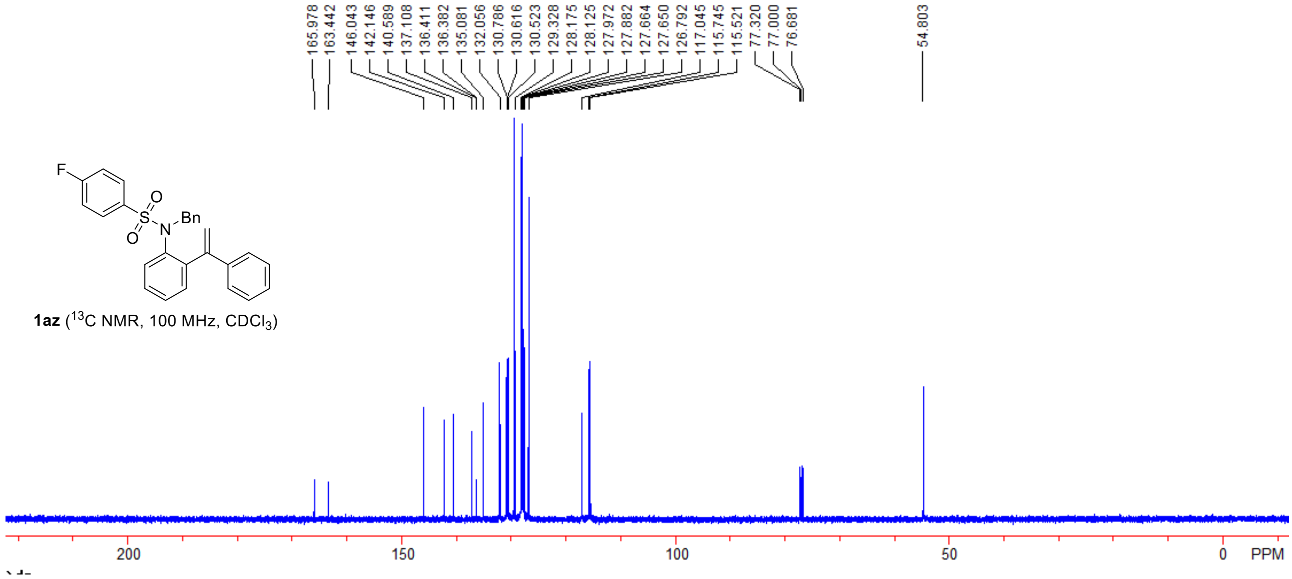

1az (^{13}C NMR, 100 MHz, $CDCl_3$)

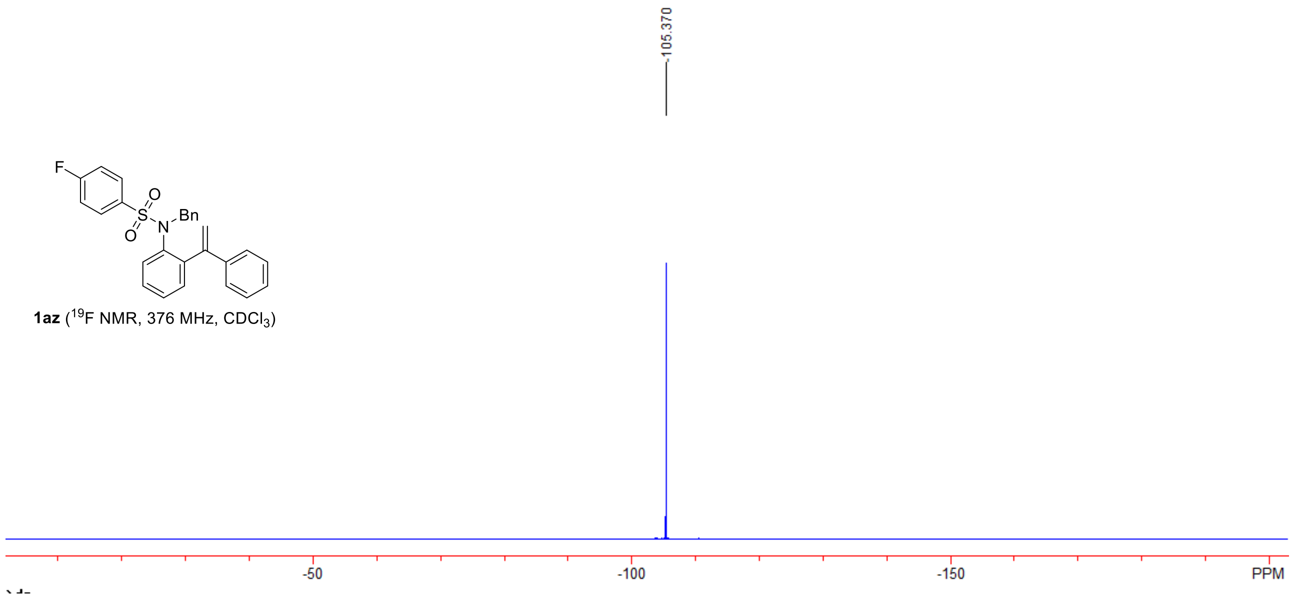

1az (^{19}F NMR, 376 MHz, $CDCl_3$)

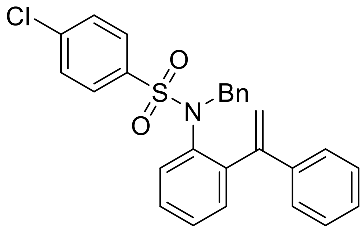

Compound 1aaa: Yield: 1855 mg, 86%; a yellow oil; Eluent: PE/EA = 10/1. ^{1}H NMR (400 MHz, $CDCl_3$, TMS) δ 3.90-4.60 (m, 2H), 5.10 (s, 1H), 5.64 (s, 1H), 6.87 (d, *J* = 8.8 Hz, 2H), 6.93 (d, *J* = 8.0 Hz, 1H), 7.03-7.30 (m, 13H), 7.45 (d, *J* = 8.4 Hz, 2H); ^{13}C{^{1}H}-NMR (101 MHz, $CDCl_3$, TMS) δ_C 54.9, 117.1, 126.8, 127.6, 127.7, 127.9, 128.0, 128.1, 128.2, 128.7, 129.30, 129.33, 130.8, 132.1, 135.0, 137.0, 138.6, 138.9, 140.5, 142.1, 146.0; IR (neat): ν 3060, 3027, 2987, 1583, 1493, 1445, 1346, 1276, 1211, 1158, 1085, 1012, 909, 824, 757, 697 cm^{-1}; HRMS (ESI-TOF) Calcd for $C_{27}H_{22}NO_2NaSCl$ $[M+Na]^+$: 482.0952, found: 482.0961.

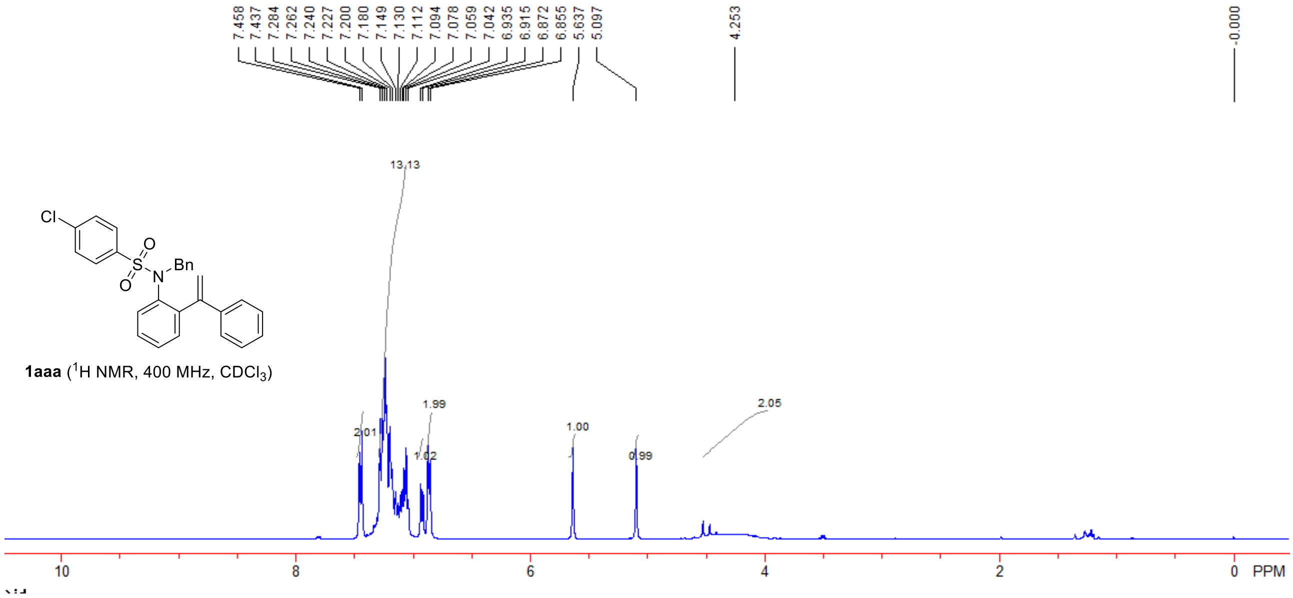

1aaa (^{1}H NMR, 400 MHz, $CDCl_3$)

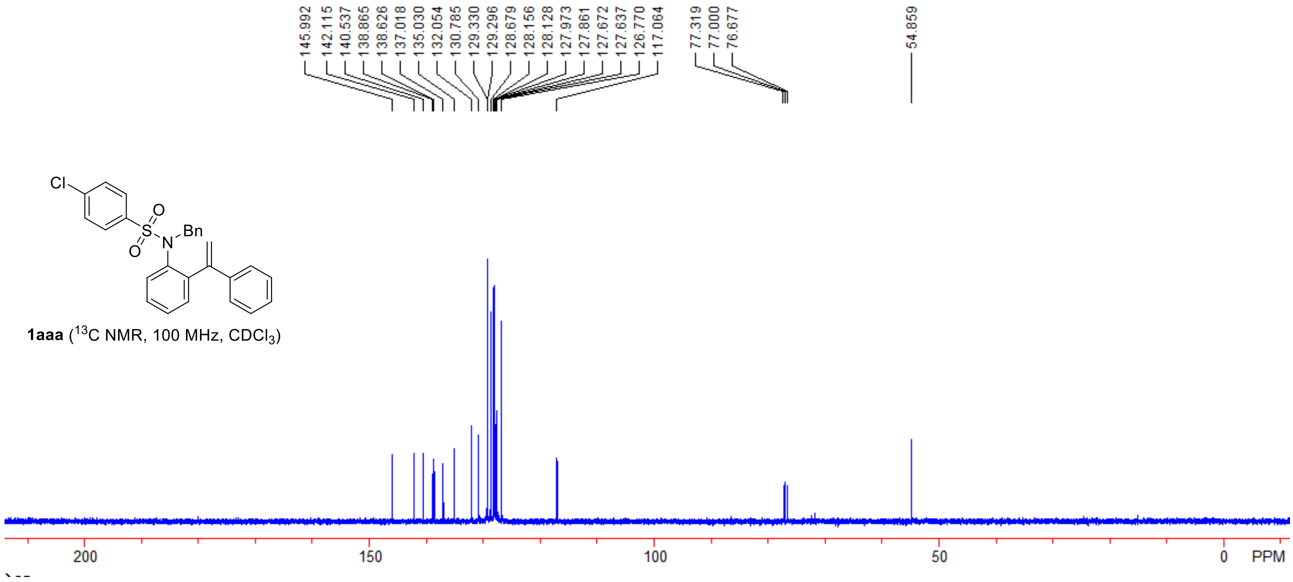

1aaa (^{13}C NMR, 100 MHz, $CDCl_3$)

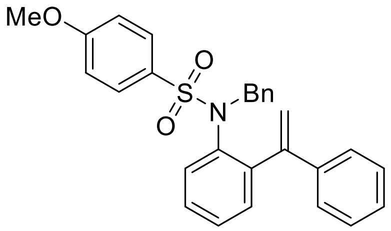

Compound 1aab: Yield: 1867 mg, 83%; a yellow oil; Eluent: PE/EA = 5/1. ^{1}H NMR (400 MHz, $CDCl_3$, TMS) δ 3.76 (s, 3H), 4.00-4.60 (m, 2H), 5.12 (s, 1H), 5.64 (s, 1H), 6.80-6.90 (m, 4H), 6.97 (d, J = 7.6 Hz, 1H), 7.02-7.28 (m, 11H), 7.51 (d, J = 8.8 Hz, 2H); $^{13}C\{^{1}H\}$-NMR (101 MHz, $CDCl_3$, TMS) δ_C 54.6, 55.4, 113.6, 117.0, 126.8, 127.43, 127.45, 127.7, 127.8, 127.9, 128.0, 129.2, 130.0, 130.6, 131.7, 131.9, 135.3, 137.3, 140.7, 142.1, 145.8, 162.6 ; IR (neat): ν 3059, 3029, 2942, 2839, 1594, 1494, 1441, 1341, 1257, 1150, 1110, 1025, 909, 831, 766, 697 cm^{-1}; HRMS (ESI-TOF) Calcd for $C_{28}H_{25}NO_3NaS$ $[M+Na]^+$: 478.1447, found: 478.1460.

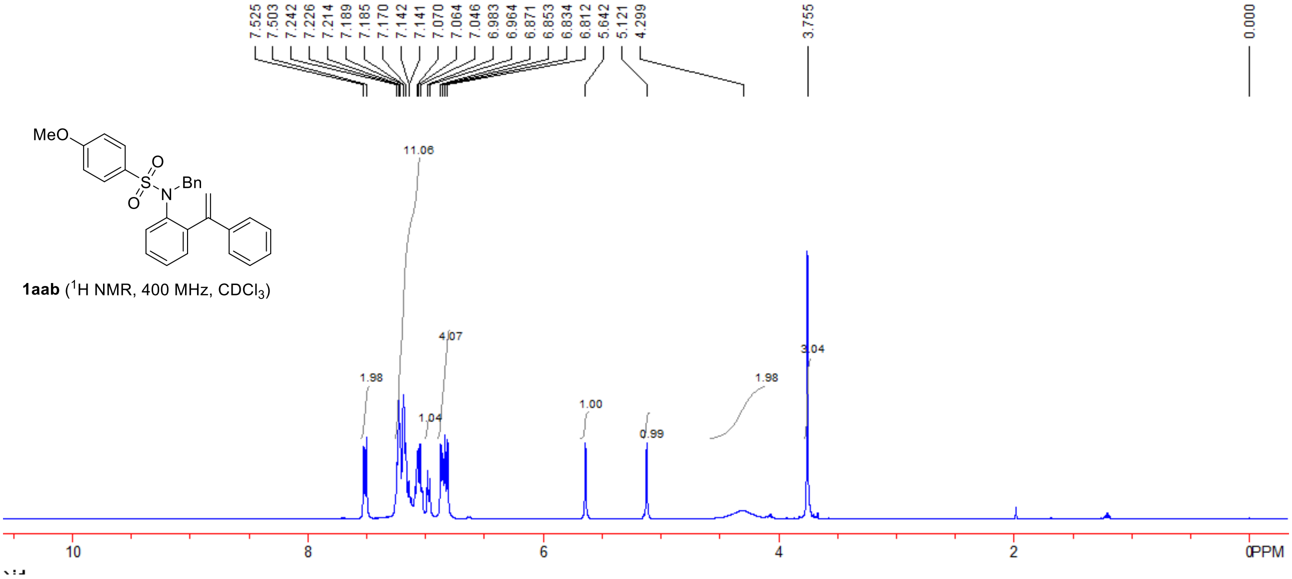

1aab (^{1}H NMR, 400 MHz, $CDCl_3$)

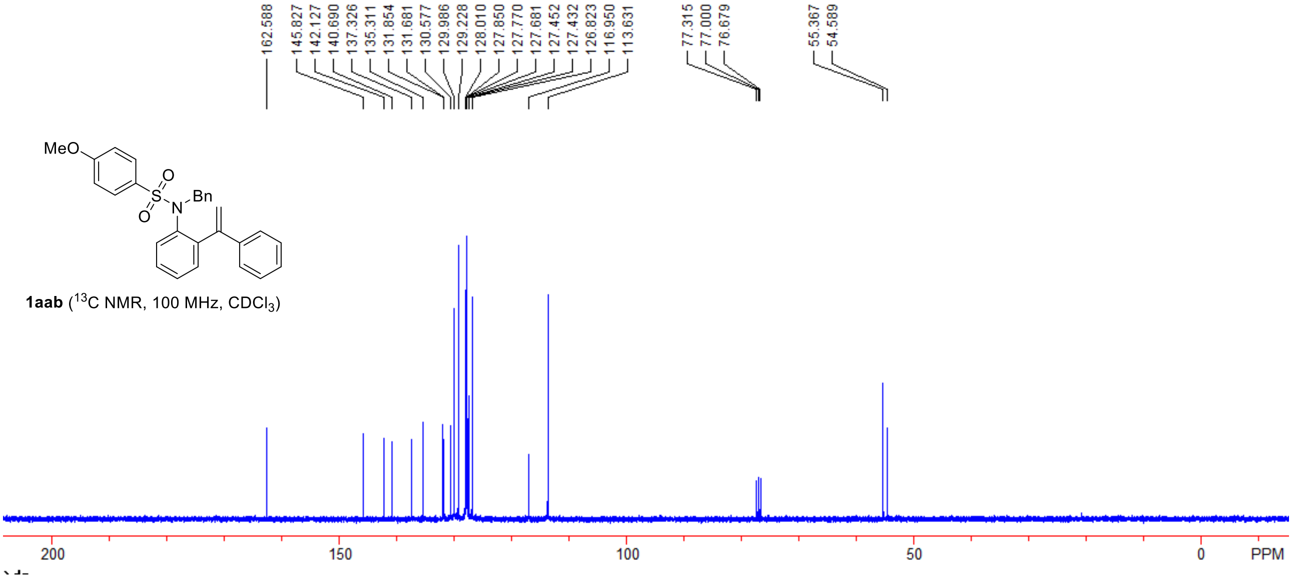

1aab (^{13}C NMR, 100 MHz, $CDCl_3$)

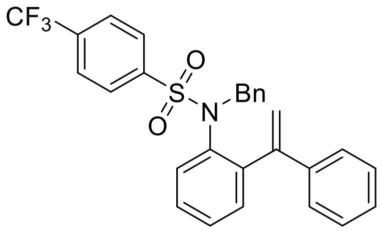

Compound 1aac: Yield: 2082 mg, 85%; a light yellow solid; Mp: 109-111 °C; Eluent: PE/EA = 10/1. ^{1}H NMR (400 MHz, $CDCl_3$, TMS) δ 3.90-4.70 (m, 2H), 5.10 (s, 1H), 5.63 (s, 1H), 6.86 (d, *J* = 6.8 Hz, 2H), 6.94 (d, *J* = 8.0 Hz, 1H), 7.00-7.35 (m, 11H), 7.55 (d, *J* = 8.0 Hz, 2H), 7.64 (d, *J* = 8.0 Hz, 2H); ^{13}C{^{1}H}-NMR (101 MHz, $CDCl_3$, TMS) δ_C 55.0, 117.0, 123.2 (q, *J* = 271.5 Hz), 125.5, (q, *J* =3.6 Hz), 126.7, 127.74, 127.75, 127.97, 127.99, 128.20, 128.28, 128.31, 129.3, 130.9, 132.1, 133.6 (q, *J* = 32.7 Hz), 134.8, 136.9, 140.5, 142.1, 144.0, 146.1; ^{19}F NMR (376 MHz, $CDCl_3$) δ -62.8; IR (neat): ν 3103, 2982, 1608, 1487, 1443, 1374, 1352, 1321, 1201, 1121, 1108, 1061, 990, 880, 770, 697 cm^{-1}; HRMS (ESI-TOF) Calcd for $C_{28}H_{22}NO_2F_3NaS$ $[M+Na]^+$: 516.1216, found: 516.1216.

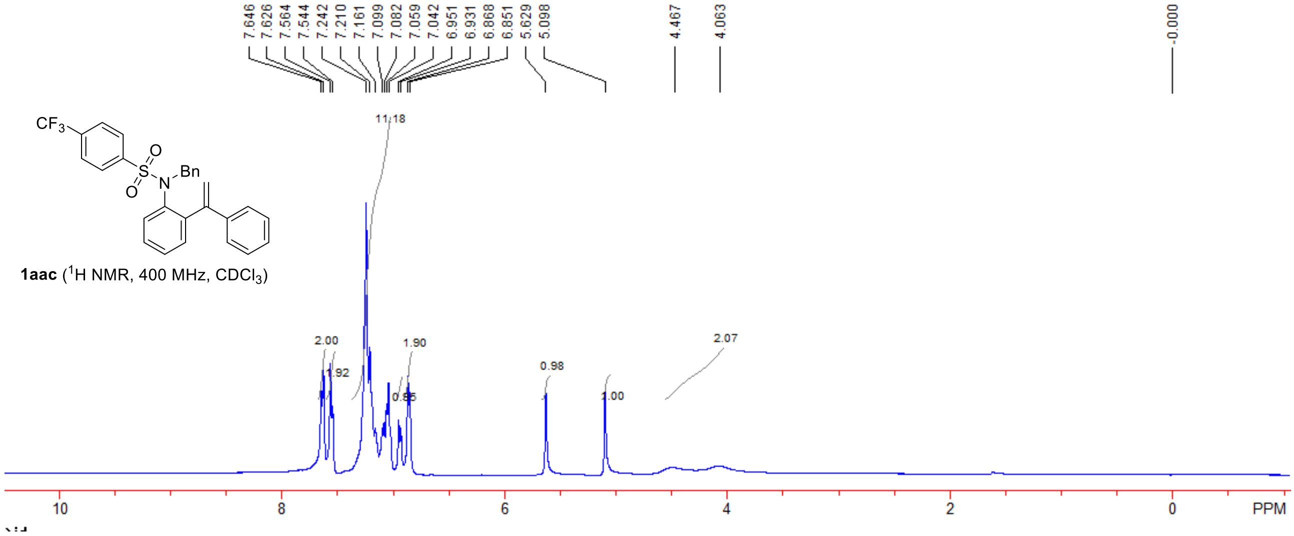

1aac (^{1}H NMR, 400 MHz, $CDCl_3$)

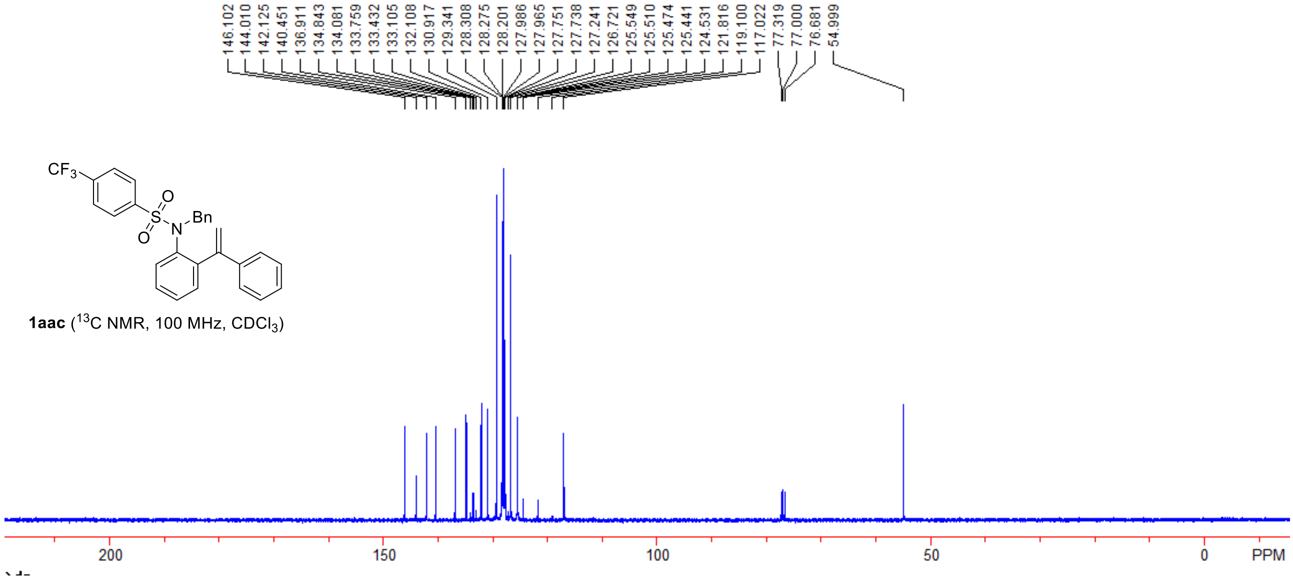

1aac (^{13}C NMR, 100 MHz, $CDCl_3$)

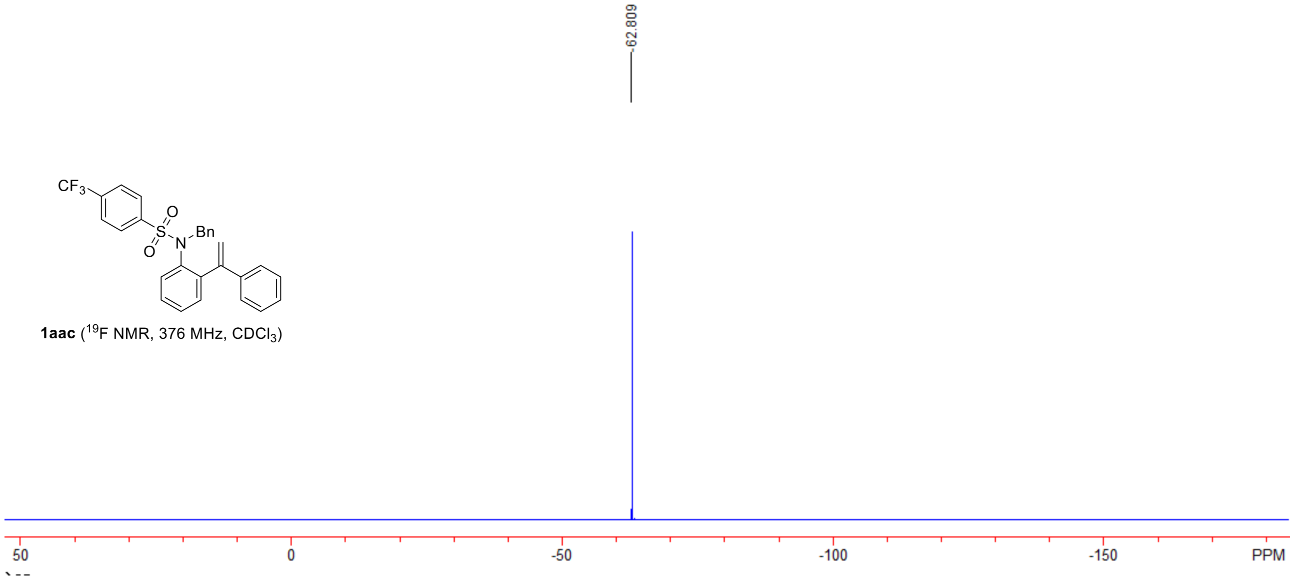

1aac (^{19}F NMR, 376 MHz, $CDCl_3$)

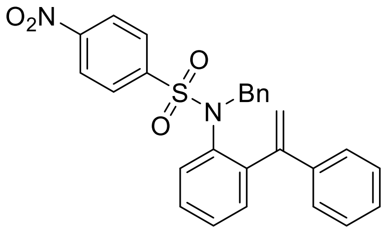

Compound 1aad: Yield: 1567 mg, 78%; a yellow oil; Eluent: PE/EA = 5/1. ^{1}H NMR (400 MHz, $CDCl_3$, TMS) δ 3.90-4.80 (m, 2H), 5.13 (s, 1H), 5.66 (s, 1H), 6.85-6.95 (m, 3H), 7.06 (t, *J* = 7.2 Hz, 2H), 7.10-7.33 (m, 9H), 7.62 (d, *J* = 8.8 Hz, 2H), 8.08 (d, *J* = 8.8 Hz, 2H); ^{13}C{^{1}H}-NMR (101 MHz, $CDCl_3$, TMS) δ_C 55.3, 117.1, 123.5, 126.6, 127.76, 127.85, 128.0, 128.2, 128.4, 128.8, 129.4, 130.8, 132.2, 134.6, 136.7, 140.2, 141.9, 145.97, 146.01, 149.3; IR (neat): ν 3102, 3063, 3029, 2986, 1605, 1526, 1493, 1346, 1309, 1159, 1088, 1027, 910, 852, 767, 738 cm^{-1}; HRMS (ESI-TOF) Calcd for $C_{27}H_{22}N_2O_4NaS$ $[M+Na]^+$: 493.1193, found: 493.1191.

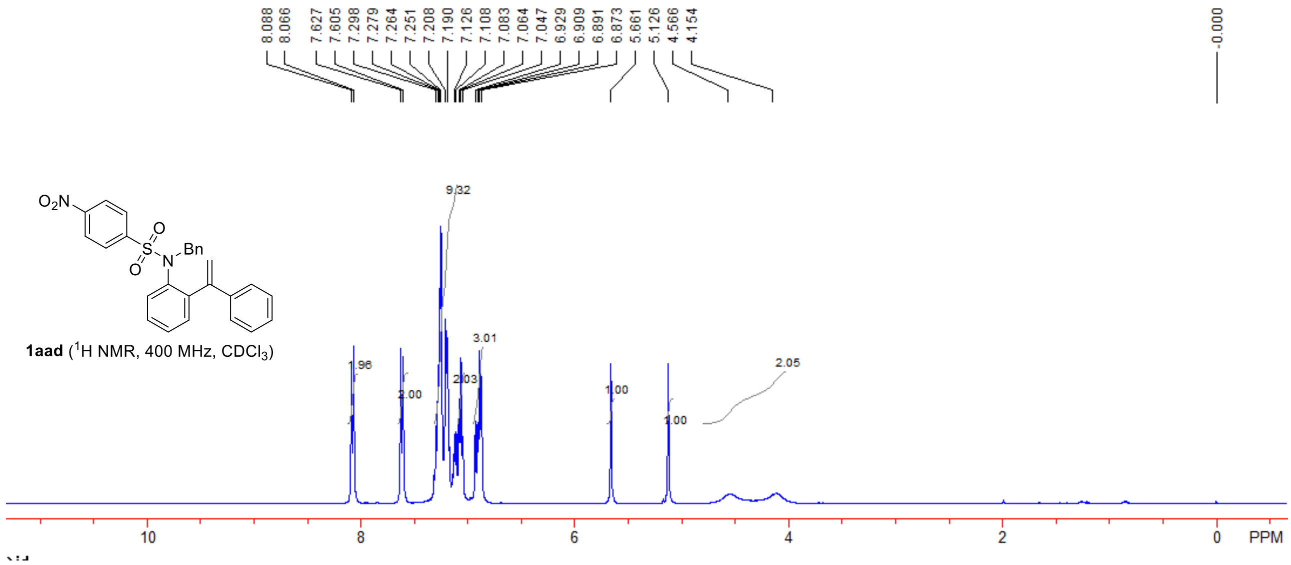

8.088 8.066 7.627 7.605 7.298 7.279 7.264 7.251 7.208 7.190 7.126 7.108 7.083 7.064 7.047 6.929 6.909 6.891 6.873 5.661 5.126 4.566 4.154
-0.000
O_2N
Bn
1aad (^{1}H NMR, 400 MHz, $CDCl_3$)
9.32
3.01
1.96
2.00
2.03
1.00
1.00
2.05
10
8
6
4
2
0
PPM

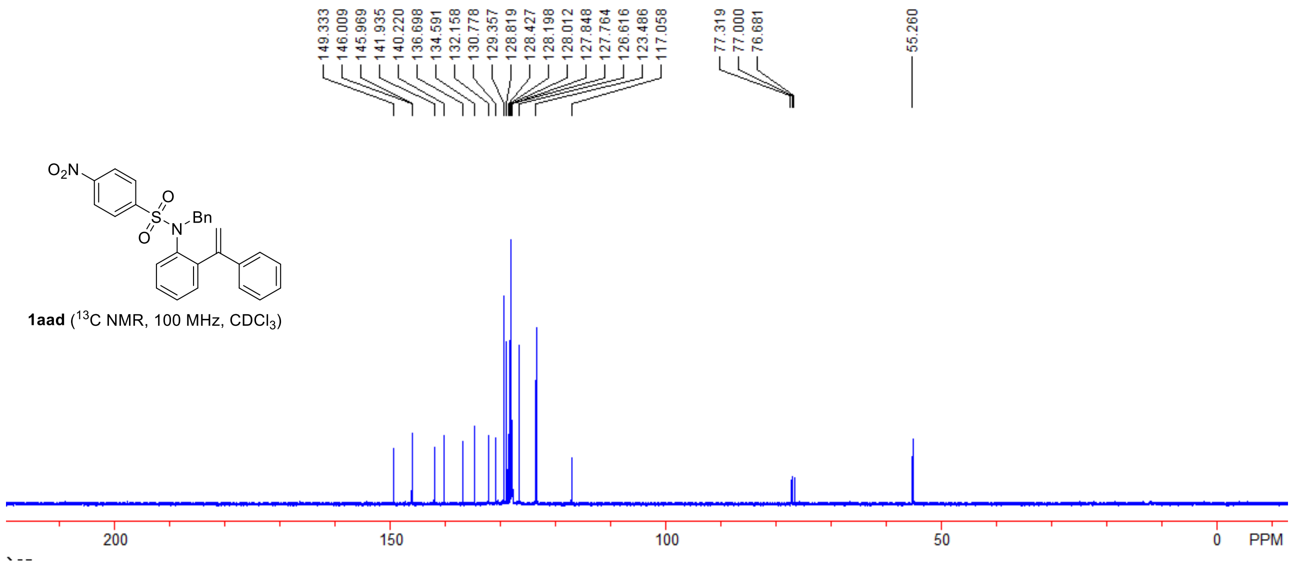

149.333 146.009 145.969 141.935 140.220 136.698 134.591 132.158 130.778 129.357 128.819 128.427 128.198 128.012 127.848 127.764 126.616 123.486 117.058
77.319 77.000 76.681
55.260
O_2N
Bn
1aad (^{13}C NMR, 100 MHz, $CDCl_3$)
200
150
100
50
0
PPM

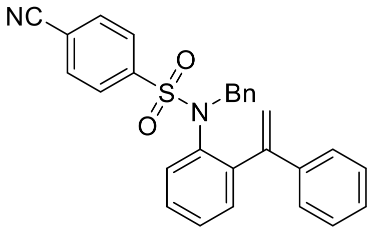

Compound 1aae: Yield: 1315 mg, 52%; a yellow oil; Eluent: PE/EA = 5/1. ^{1}H NMR (400 MHz, $CDCl_3$, TMS) δ 3.80-4.70 (m, 2H), 5.10 (s, 1H), 5.65 (s, 1H), 6.83-6.92 (m, 3H), 7.08 (t, *J* = 7.2 Hz, 2H), 7.13 (d, *J* = 6.8 Hz, 1H), 7.15-7.35 (m, 8H), 7.54-7.58 (m, 4H); ^{13}C{^{1}H}-NMR (101 MHz, $CDCl_3$, TMS) δ_C 55.2, 115.6, 117.1, 117.3, 126.6, 127.78, 127.84, 128.00, 128.02, 128.2, 128.4, 129.3, 130.8, 132.1, 134.6, 136.8, 140.3, 142.0, 144.5, 146.0; IR (neat): ν 3028, 2987, 2231, 1595, 1486, 1445, 1347, 1181, 1157, 1087, 1027, 910, 829, 766, 698 cm^{-1}; HRMS (ESI-TOF) Calcd for $C_{28}H_{22}N_2O_2NaS$ [M+Na]$^+$: 473.1294, found: 473.1303.

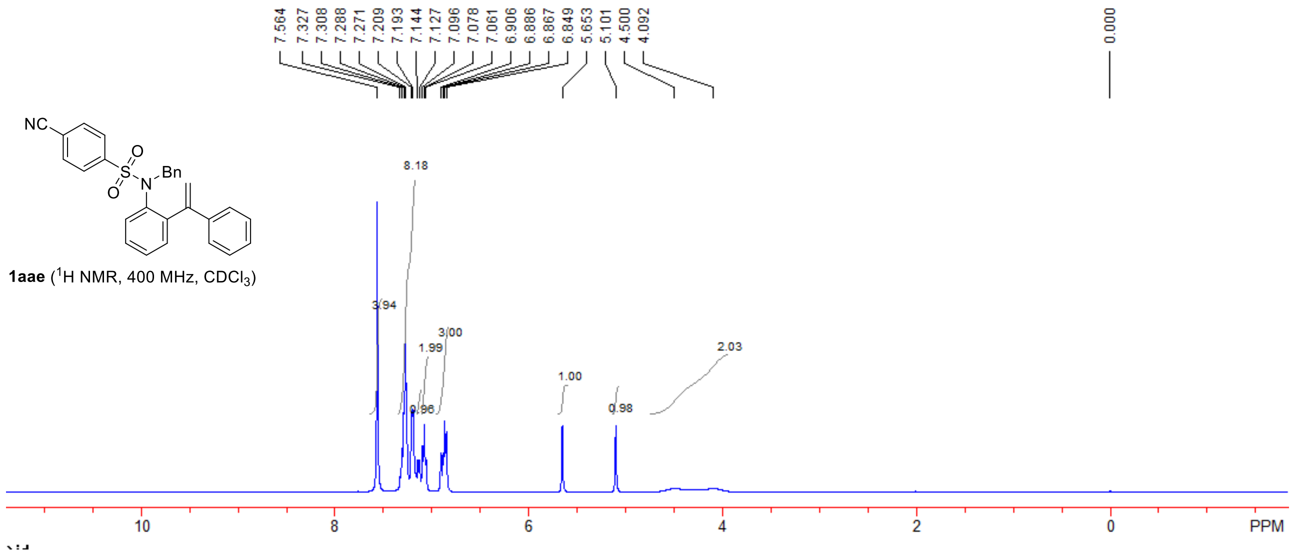

1aae (^{1}H NMR, 400 MHz, $CDCl_3$)

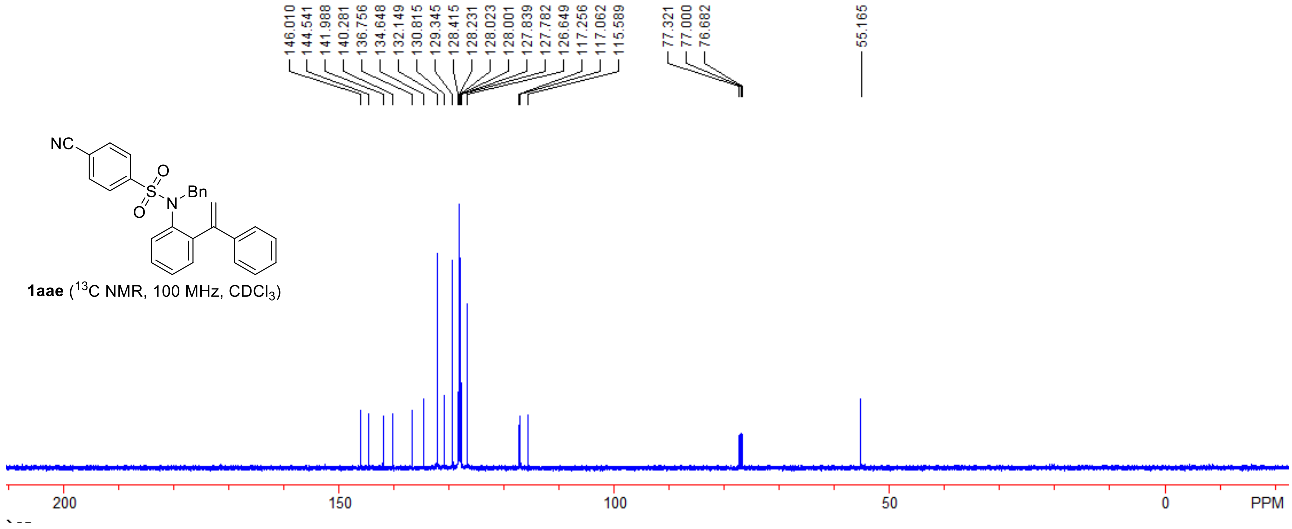

1aae (^{13}C NMR, 100 MHz, $CDCl_3$)

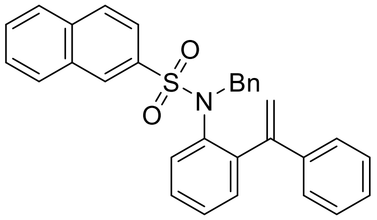

Compound 1aaf: Yield: 1809 mg, 74%; a yellow oil; Eluent: PE/EA = 10/1. ^{1}H NMR (400 MHz, $CDCl_3$, TMS) δ 4.00-4.80 (m, 2H), 5.09 (s, 1H), 5.58 (s, 1H), 6.88 (d, *J* = 7.2 Hz, 2H), 6.96 (d, *J* = 8.0 Hz, 1H), 6.98-7.08 (m, 3H), 7.10-7.28 (m, 8H), 7.48-7.61 (m, 3H), 7.75-7.87 (m, 3H), 8.20 (s, 1H); ^{13}C{^{1}H}-NMR (101 MHz, $CDCl_3$, TMS) δ_C 54.8, 117.1, 123.2, 126.9, 127.2, 127.57, 127.60, 127.64, 127.8, 127.9, 128.0, 128.1, 128.6, 128.7, 129.1, 129.2, 129.3, 131.0, 131.8, 132.0, 134.5, 135.3, 137.2, 137.3, 140.7, 142.3, 145.8; IR (neat): ν 3053, 3026, 2986, 2920, 1596, 1509, 1441, 1361, 1305, 1276, 1212, 1156, 1115, 1085, 1037, 998, 891, 784, 705 cm^{-1}; HRMS (ESI-TOF) Calcd for $C_{31}H_{25}NO_2NaS$ $[M+Na]^+$: 498.1498, found: 498.1504.

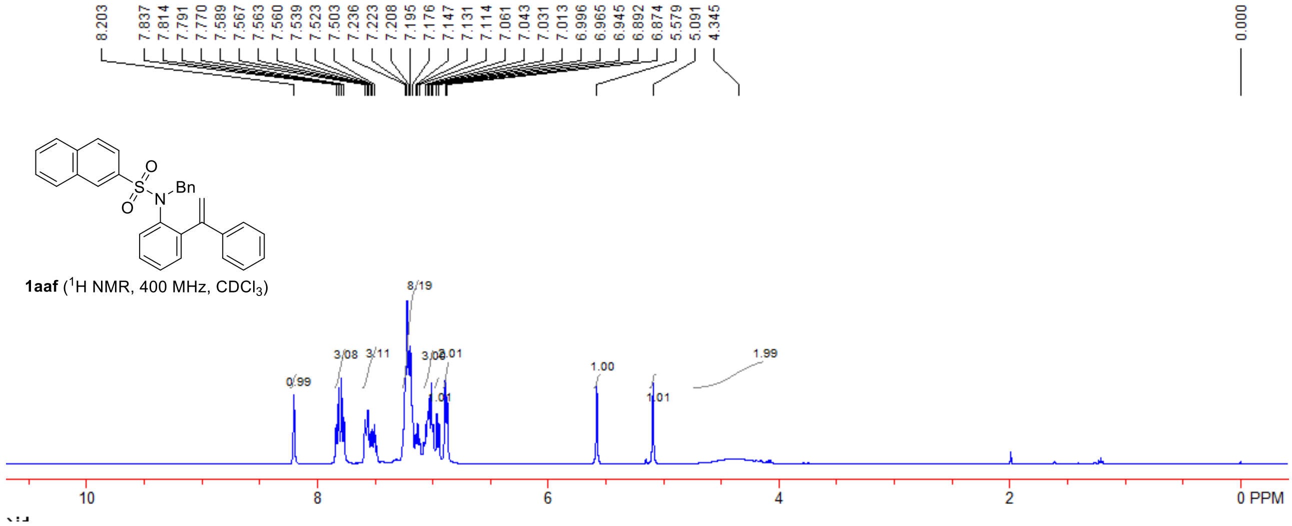

1aaf (^{1}H NMR, 400 MHz, $CDCl_3$)

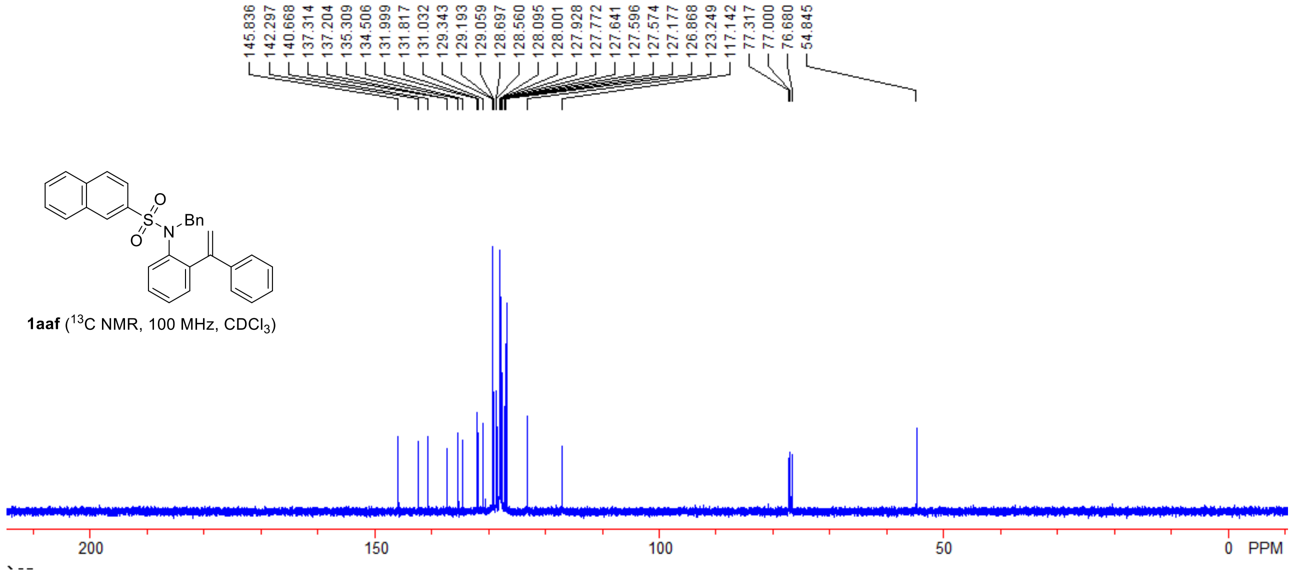

1aaf (^{13}C NMR, 100 MHz, $CDCl_3$)

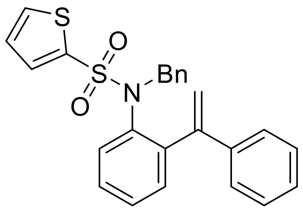

Compound 1aag: Yield: 1518 mg, 60%; a yellow oil; Eluent: PE/EA = 10/1. ^{1}H NMR (400 MHz, $CDCl_3$, TMS) δ 4.00-4.80 (m, 2H), 5.20 (s, 1H), 5.69 (s, 1H), 6.86 (d, *J* = 6.8 Hz, 2H), 6.90-6.97 (m, 2H), 7.02-7.26 (m, 11H), 7.31-7.35 (m, 1H), 7.43-7.48 (m, 1H); ^{13}C{^{1}H}-NMR (101 MHz, $CDCl_3$, TMS) δ_C 54.6, 117.2, 126.9, 127.0, 127.5, 127.65, 127.75, 127.95, 128.02, 128.04, 129.2, 130.3, 131.87, 131.91, 132.7, 134.9, 136.9, 140.6, 141.0, 142.3, 145.6; IR (neat): ν 3059, 3028, 2917, 1597, 1493, 1441, 1403, 1348, 1224, 1150, 1091, 1015, 909, 864, 766, 730 cm^{-1}; HRMS (ESI-TOF) Calcd for $C_{25}H_{21}NO_2NaS_2$ $[M+Na]^+$: 454.0906, found: 454.0911.

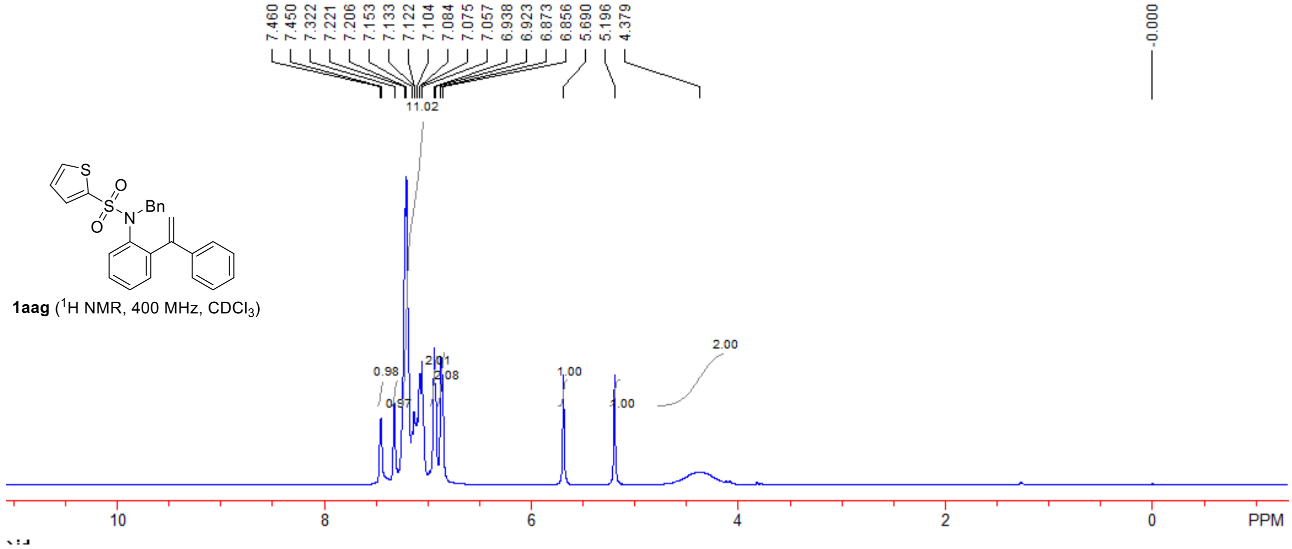

1aag (^{1}H NMR, 400 MHz, $CDCl_3$)

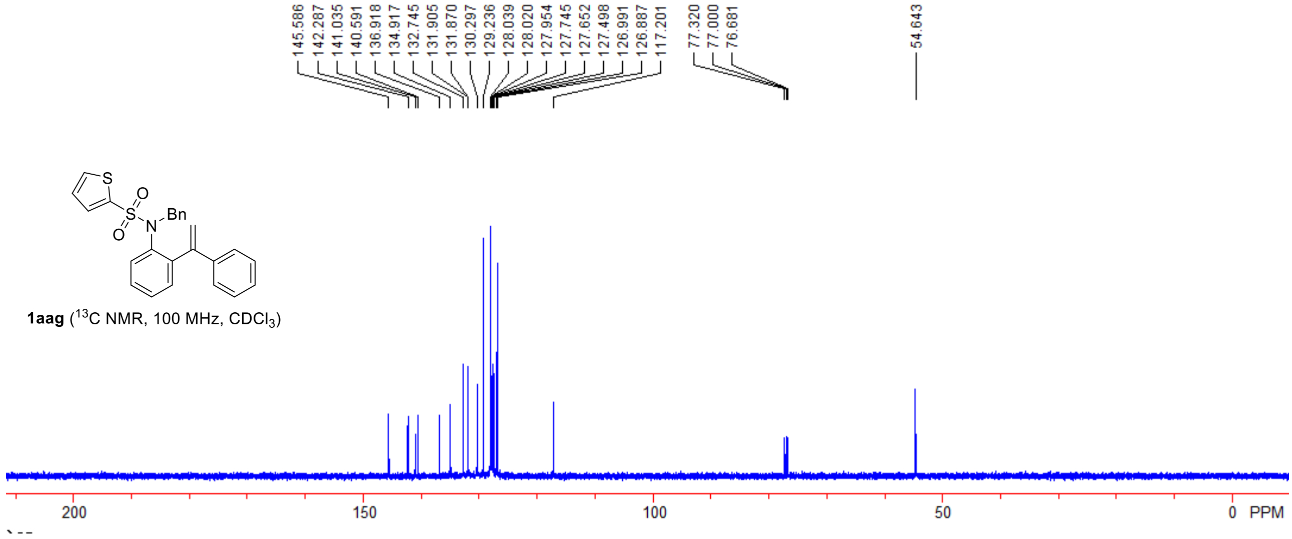

1aag (^{13}C NMR, 100 MHz, $CDCl_3$)

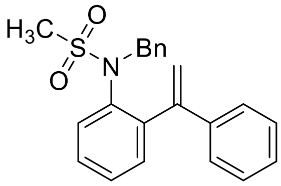

Compound 1aah: Yield: 1015 mg, 90%; a light yellow solid; Mp: 80-82 ^{o}C; Eluent: PE/EA = 10/1. ^{1}H NMR (400 MHz, $CDCl_3$, TMS) δ 2.31 (s, 3H), 4.00-4.60 (m, 2H), 5.35 (s, 1H), 5.78 (s, 1H), 7.04 (d, J = 8.0 Hz, 1H), 7.07-7.13 (m, 2H), 7.17-7.24 (m, 4H), 7.25-7.35 (m, 7H); ^{13}C{^{1}H}-NMR (101 MHz, $CDCl_3$, TMS) δ_C 40.6, 54.3, 117.0, 126.7, 127.86, 127.93, 128.08, 128.18, 128.21, 128.4, 129.5, 131.7, 131.9, 135.4, 137.3, 140.8, 141.3, 147.7; IR (neat): ν 3083, 3064, 3026, 3006, 2940, 1595, 1490, 1445, 1368, 1269, 1201, 1145, 1086, 1027, 964, 866, 785, 712, 699 cm^{-1}; HRMS (ESI-TOF) Calcd for $C_{22}H_{21}NO_2NaS$ $[M+Na]^+$: 386.1185, found: 386.1194.

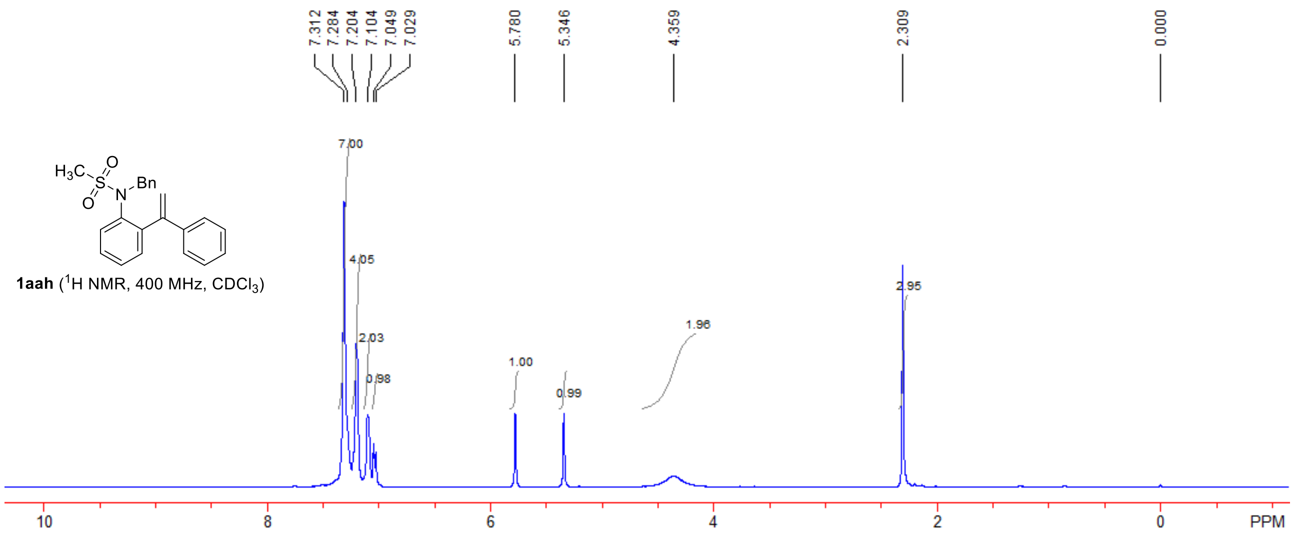

1aah (^{1}H NMR, 400 MHz, $CDCl_3$)

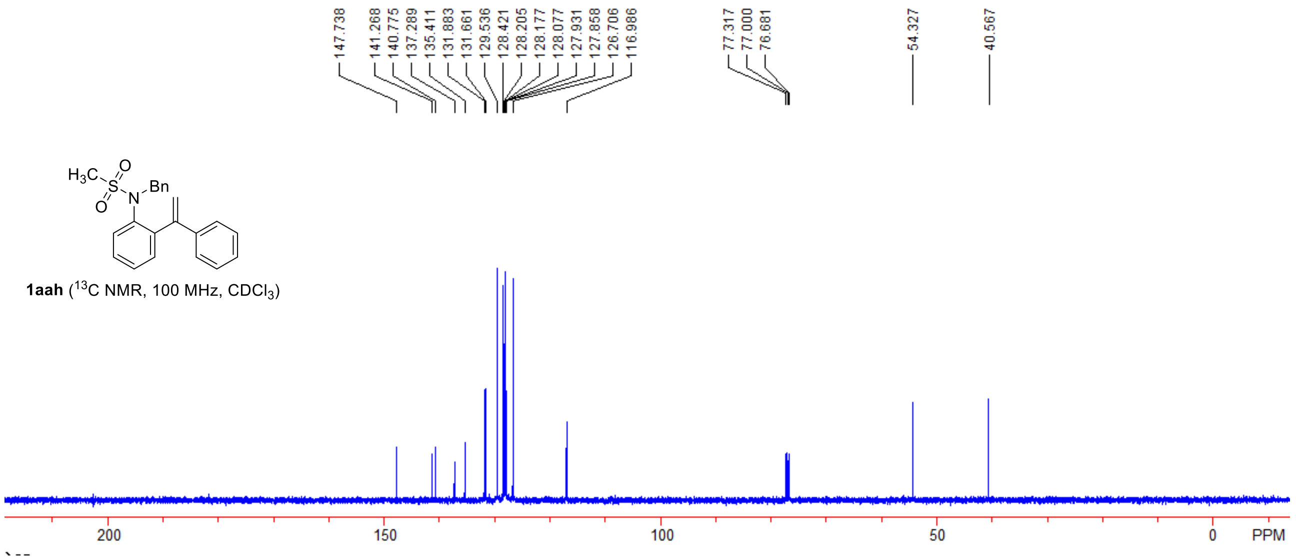

1aah (^{13}C NMR, 100 MHz, $CDCl_3$)

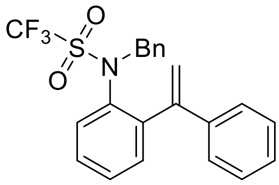

Compound 1aai: Yield: 1244 mg, 67%; a yellow oil; Eluent: PE/EA = 10/1. ^{1}H NMR (400 MHz, $CDCl_3$, TMS) δ 3.90-4.10 (m, 1H), 4.46-4.48 (m, 1H), 5.37 (s, 1H), 5.86 (s, 1H), 6.85 (d, *J* = 7.6 Hz, 1H), 6.95 (d, *J* = 6.4 Hz, 2H), 7.12-7.26 (m, 4H), 7.27-7.40 (m, 7H); ^{13}C{^{1}H}-NMR (101 MHz, $CDCl_3$, TMS) δ_C 55.5, 118.4, 119.8 (q, *J* = 321.6 Hz), 127.1, 128.2, 128.25, 128.39, 128.48, 128.49, 129.2, 129.6, 131.8, 132.3, 133.8, 134.5, 140.2, 142.1, 145.9; ^{19}F NMR (376 MHz, $CDCl_3$) δ -74.2; IR (neat): ν 3063, 3031, 2926, 2859, 1600, 1494, 1389, 1223, 1186, 1139, 1040, 1027, 911, 765, 696 cm^{-1}; HRMS (FI-TOF) Calcd for $C_{22}H_{18}NO_2F_3S$ $[M]^+$: 417.1005, found: 417.1007.

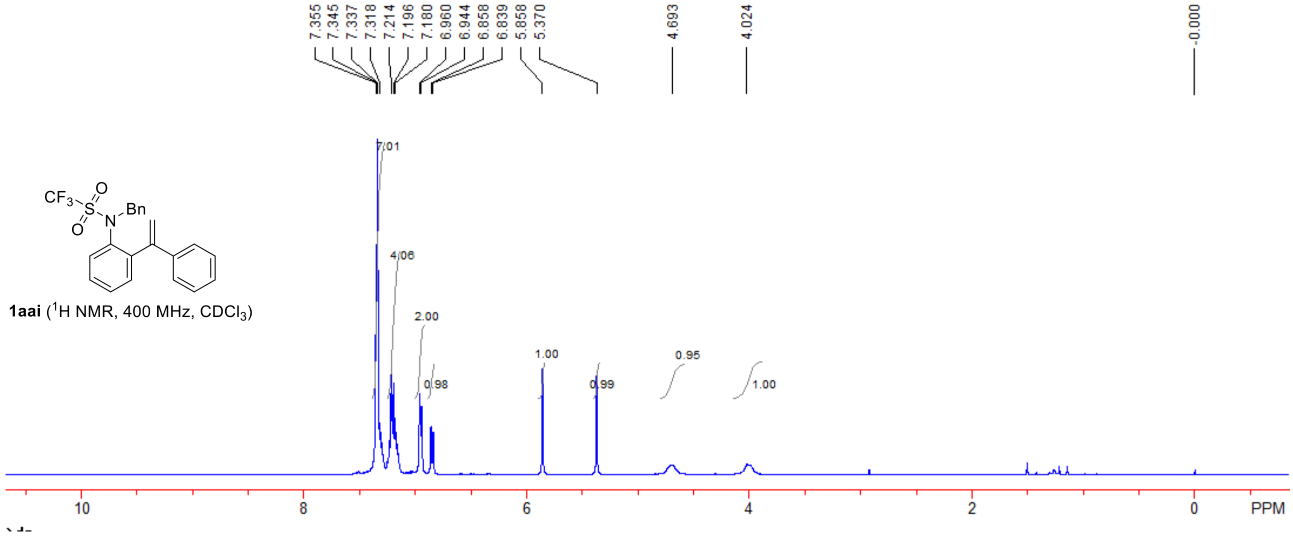

1aai (^{1}H NMR, 400 MHz, $CDCl_3$)

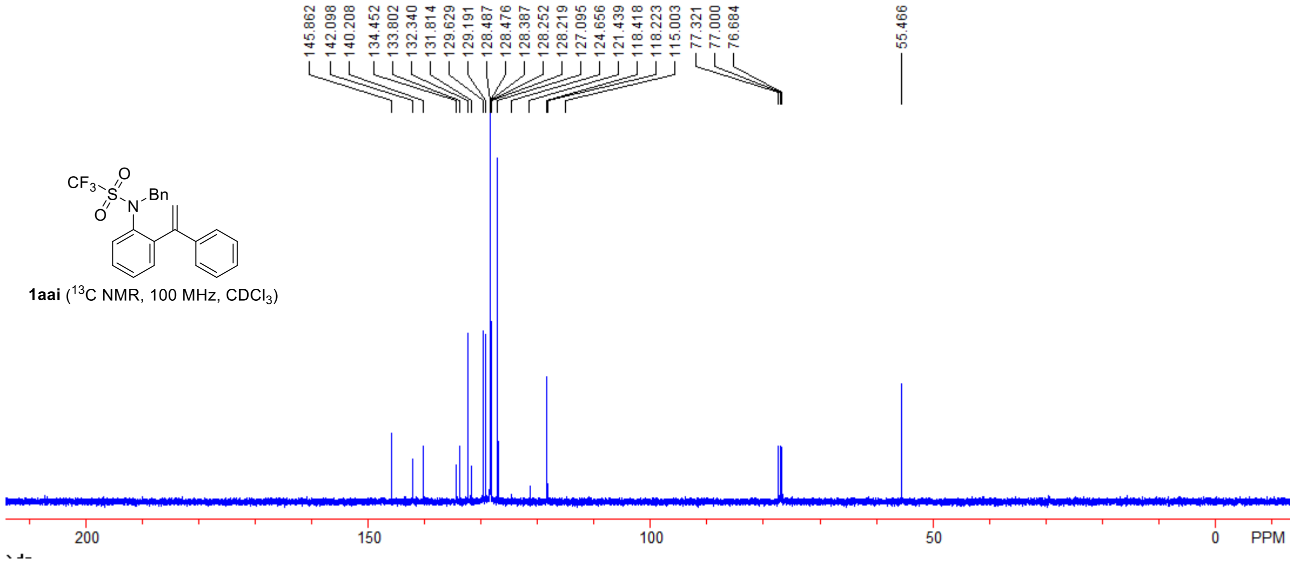

1aai (^{13}C NMR, 100 MHz, $CDCl_3$)

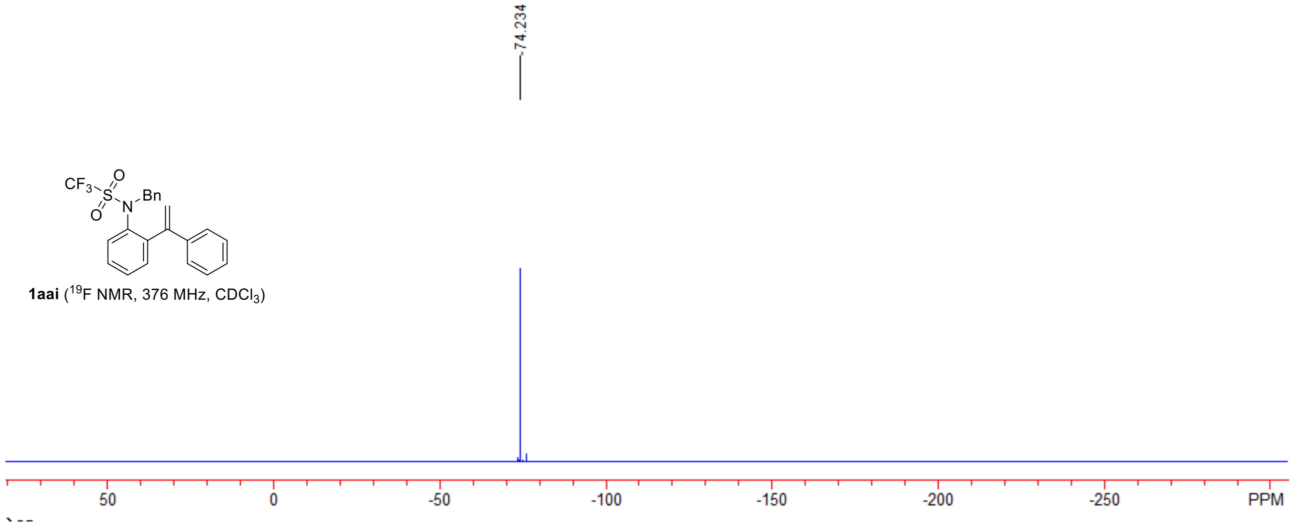

1aai (^{19}F NMR, 376 MHz, $CDCl_3$)

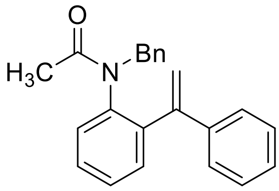

Compound 1aaj: Yield: 1076 mg, 81%; a yellow oil; Eluent: PE/EA = 10/1. ^{1}H NMR (400 MHz, $CDCl_3$, TMS) δ 1.64 (s, 3H), 3.44 (d, *J* = 14.4 Hz, 1H), 5.26 (d, *J* = 14.4 Hz, 1H), 5.32 (s, 1H), 5.65 (s, 1H), 6.65 (d, *J* = 7.6 Hz, 1H), 7.09 (d, *J* = 7.2 Hz, 2H), 7.12-7.22 (m, 4H), 7.24-7.36 (m, 6H), 7.43 (d, *J* = 6.8 Hz, 1H); ^{13}C{^{1}H}-NMR (101 MHz, $CDCl_3$, TMS) δ_C 22.2, 50.4, 117.1, 126.6, 126.9, 127.90, 127.95, 127.98, 128.2, 128.4, 128.6, 130.1, 131.4, 137.4, 139.9, 140.0, 140.5, 148.1, 169.9; IR (neat): ν 3060, 3027, 2931, 1655, 1485, 1448, 1384, 1358, 1319, 1253, 1069, 1027, 907, 766, 699 cm^{-1}; HRMS (ESI-TOF) Calcd for $C_{23}H_{21}NONa$ $[M+Na]^+$: 350.1515, found: 350.1511.

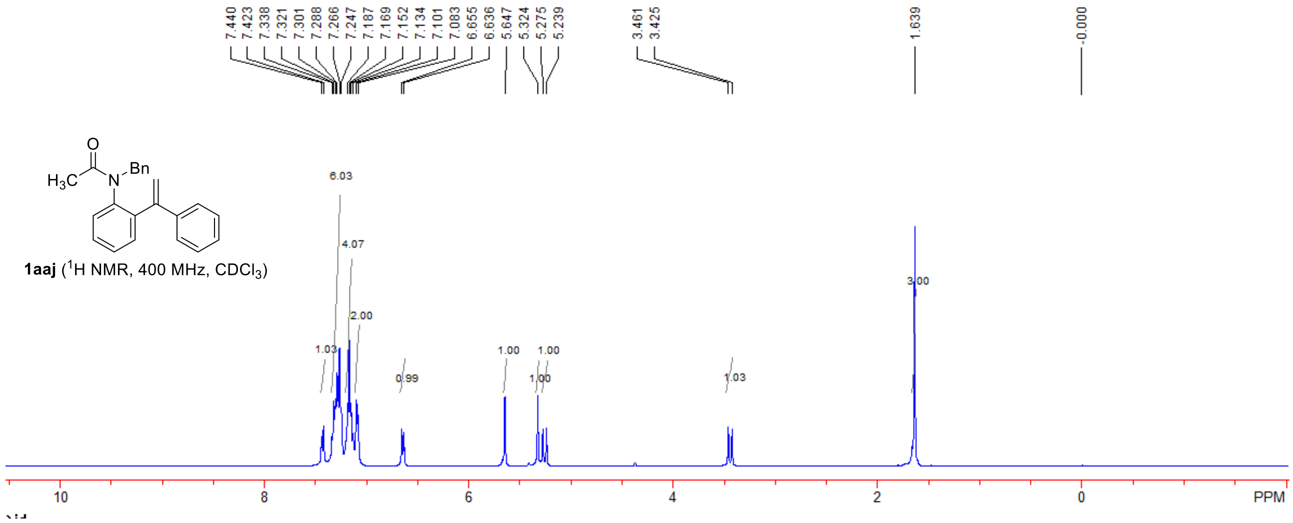

1aaj (^{1}H NMR, 400 MHz, $CDCl_3$)

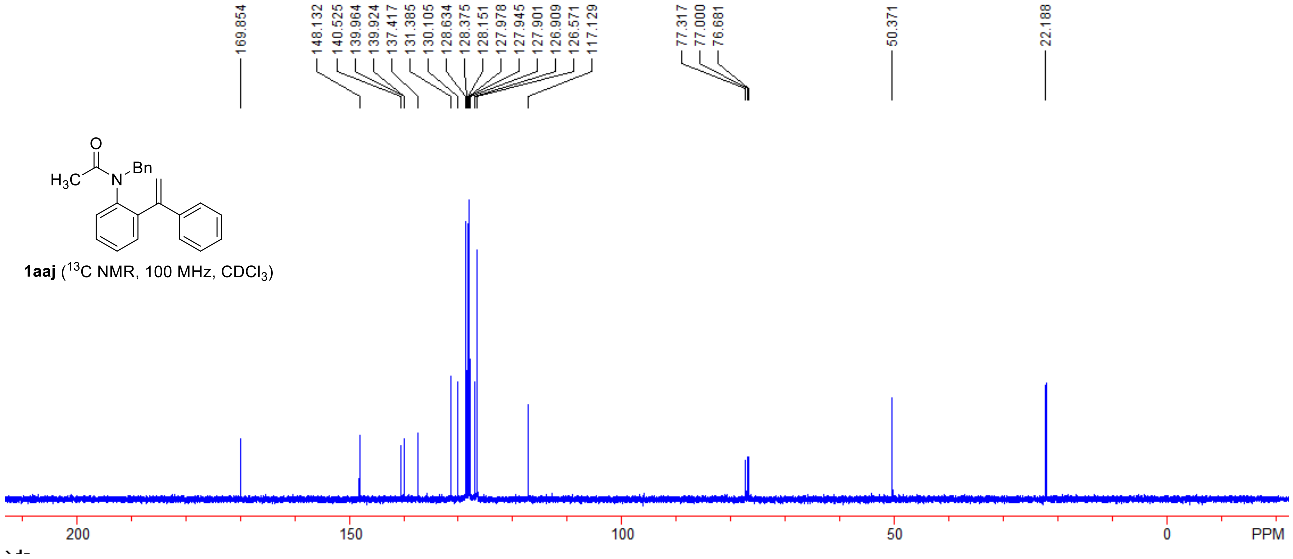

1aaj (^{13}C NMR, 100 MHz, $CDCl_3$)

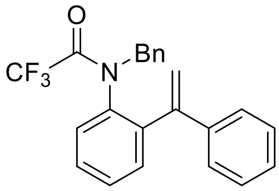

Compound 1aak: Yield: 1116 mg, 83%; a yellow oil; Eluent: PE/EA = 10/1. ^{1}H NMR (400 MHz, $CDCl_3$, TMS) δ 3.58 (d, *J* = 14.0 Hz, 1.00H), 4.26 (d, *J* = 15.6 Hz, 0.15H), 4.85 (d, *J* = 15.6 Hz, 0.15H), 5.21 (d, *J* = 14.4 Hz, 1.00H), 5.35-5.40 (m, 1.15H), 5.72-5.80 (m, 1.15H), 6.66-6.74 (m, 1.15H), 6.96-7.10 (m, 2.30H), 7.11-7.46 (m, 12.65H); ^{13}C{^{1}H}-NMR (101 MHz, $CDCl_3$, TMS) δ_C 52.5, 53.0, 116.2 (q, *J* = 287.0 Hz), 116.9, 118.3, 126.6, 126.9, 127.9, 128.1, 128.16, 128.29, 128.32, 128.35, 128.43, 128.46, 128.53, 129.07, 129.09, 129.95, 129.96, 130.8, 131.72, 131.74, 135.4, 136.5, 139.6, 140.4, 146.8, 147.2, 157.1 (q, *J* =35.3 Hz); ^{19}F NMR (376 MHz, $CDCl_3$) δ -68.1, -67.1; IR (neat): ν 3062, 3030, 2950, 1687, 1487, 1449, 1418, 1357, 1201, 1174, 1146, 1078, 908, 766, 732, 696 cm^{-1}; HRMS (ESI-TOF) Calcd for $C_{23}H_{18}NOF_3Na$ [M+Na]$^+$: 404.1233, found: 404.1229.

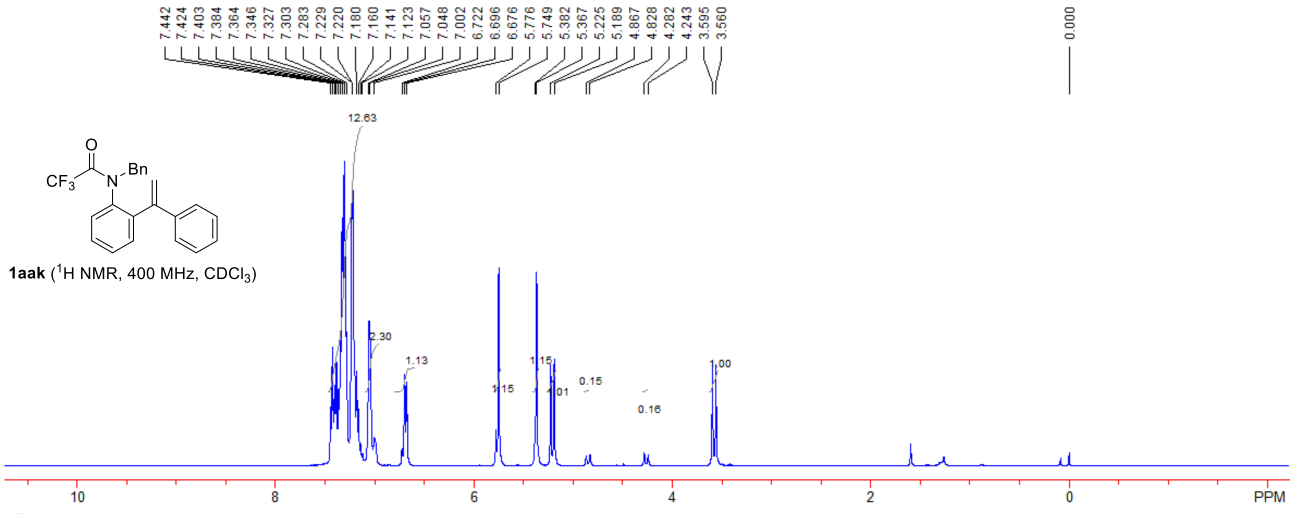

1aak (^{1}H NMR, 400 MHz, $CDCl_3$)

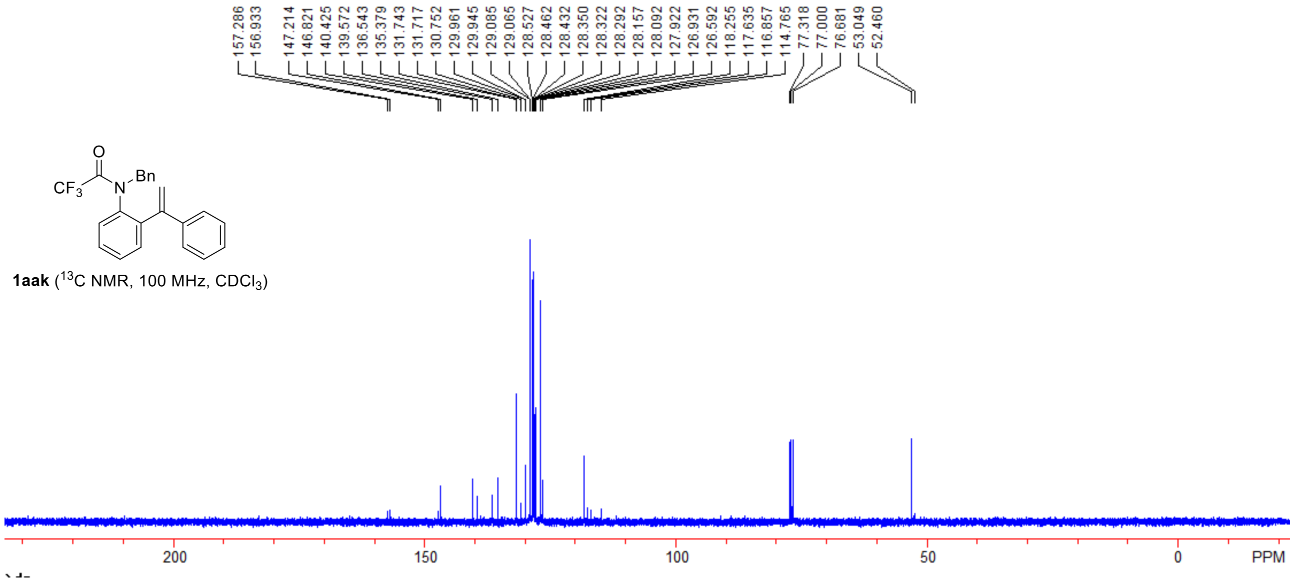

1aak (^{13}C NMR, 100 MHz, $CDCl_3$)

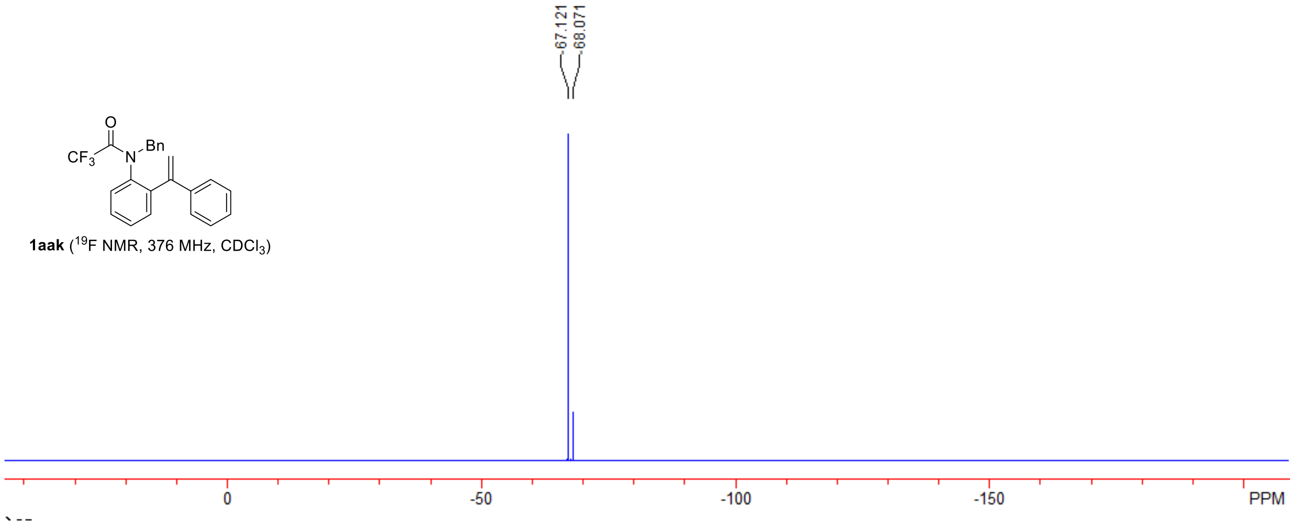

1aak (^{19}F NMR, 376 MHz, $CDCl_3$)

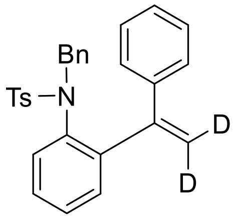

Compound [*D*$_2$]-**1a**: Yield: 2283 mg, 75%, D containing 93%; a yellow oil; Eluent: PE/EA = 30/1. ^{1}H NMR (400 MHz, $CDCl_3$, TMS) δ 2.32 (s, 3H), 4.00-4.60 (m, 2H), 6.86 (d, *J* = 7.2 Hz, 2H), 6.94 (d, *J* = 7.6 Hz, 1H), 7.00-7.09 (m, 3H), 7.10-7.26 (m, 10H), 7.50 (d, *J* = 8.0 Hz, 2H); ^{13}C{^{1}H}-NMR (101 MHz, $CDCl_3$, TMS) δ_C 21.2, 54.5, 126.8, 127.38, 127.42, 127.6, 127.7, 127.81, 127.84, 128.0, 129.1, 129.2, 130.6, 131.8, 135.2, 137.08, 137.13, 140.6, 142.1, 143.1, 145.5; IR (neat): ν 3059, 3028, 2922, 1597, 1492, 1402, 1342, 1212, 1183, 1106, 1042, 912, 855, 814, 766, 725 cm^{-1}; HRMS (ESI-TOF) Calcd for $C_{28}H_{23}NO_2NaSD_2$ $[M+Na]^+$: 464.1624, found: 464.1626.

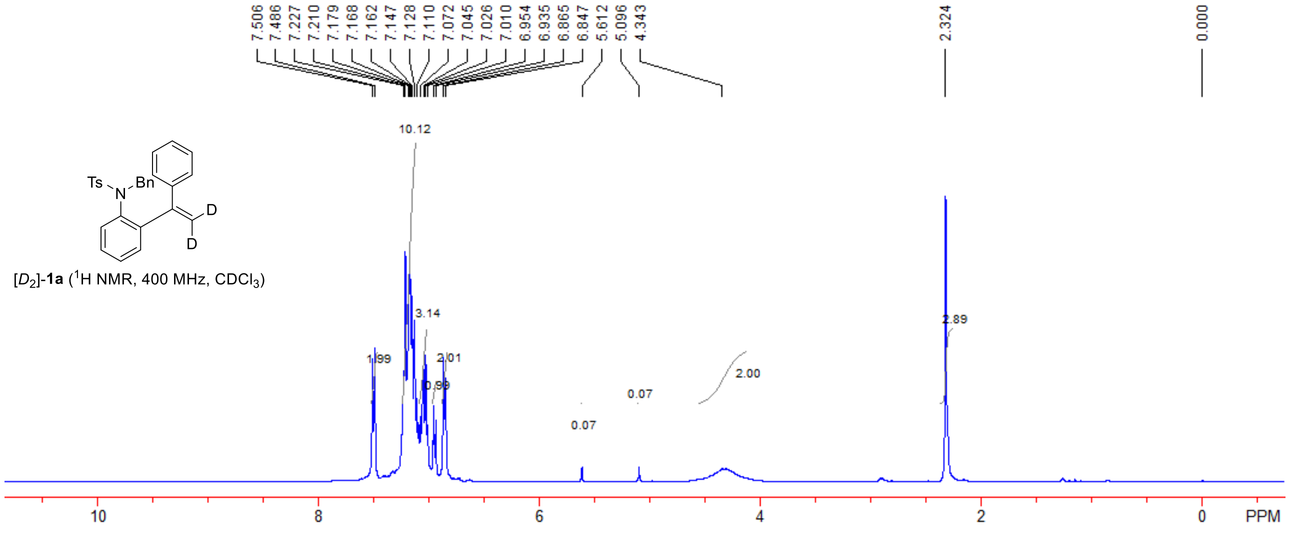

[D_2]-**1a** (^{1}H NMR, 400 MHz, $CDCl_3$)

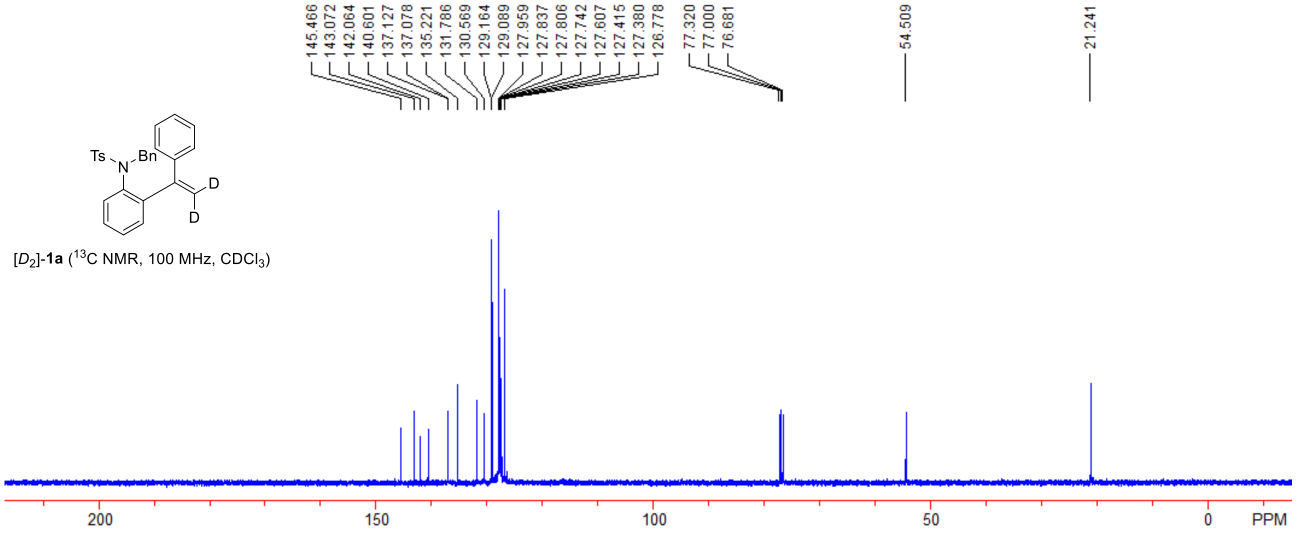

[D_2]-**1a** (^{13}C NMR, 100 MHz, $CDCl_3$)

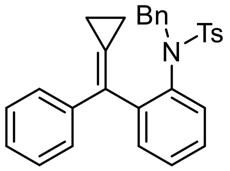

Compound 1aal: Yield: 818 mg, 88%; a yellow solid; Mp: 139-141 °C; Eluent: PE/EA = 10/1. ^{1}H NMR (400 MHz, $CDCl_3$, TMS) δ 7.42–7.37 (m, 2H), 7.37–7.29 (m, 6H), 7.28–7.25 (m, 1H), 7.22–7.16 (m, 1H), 7.14–7.08 (m, 3H), 7.07–7.01 (m, 2H), 6.99–6.92 (m, 1H), 6.78–6.67 (m, 2H), 4.15 (s, 2H), 2.39 (s, 3H), 1.48–1.40 (m, 2H), 1.15–1.00 (m, 2H); ^{13}C{^{1}H}-NMR (101 MHz, $CDCl_3$, TMS) δ_C 142.9, 141.9, 140.6, 138.0, 137.9, 135.6, 132.5, 131.5, 129.4, 129.1, 128.2, 128.0, 127.8, 127.7, 127.4, 127.1, 127.0, 126.9 54.5, 21.5, 5.2, 2.1; IR (neat): ν 2969, 1597, 1493, 1329, 1155, 1091, 856, 814, 720, 696, 653 cm^{-1}; HRMS (ESI-TOF) Calcd for $C_{30}H_{27}NO_2NaS$ $[M+Na]^+$: 488.1655, found: 488.1651.

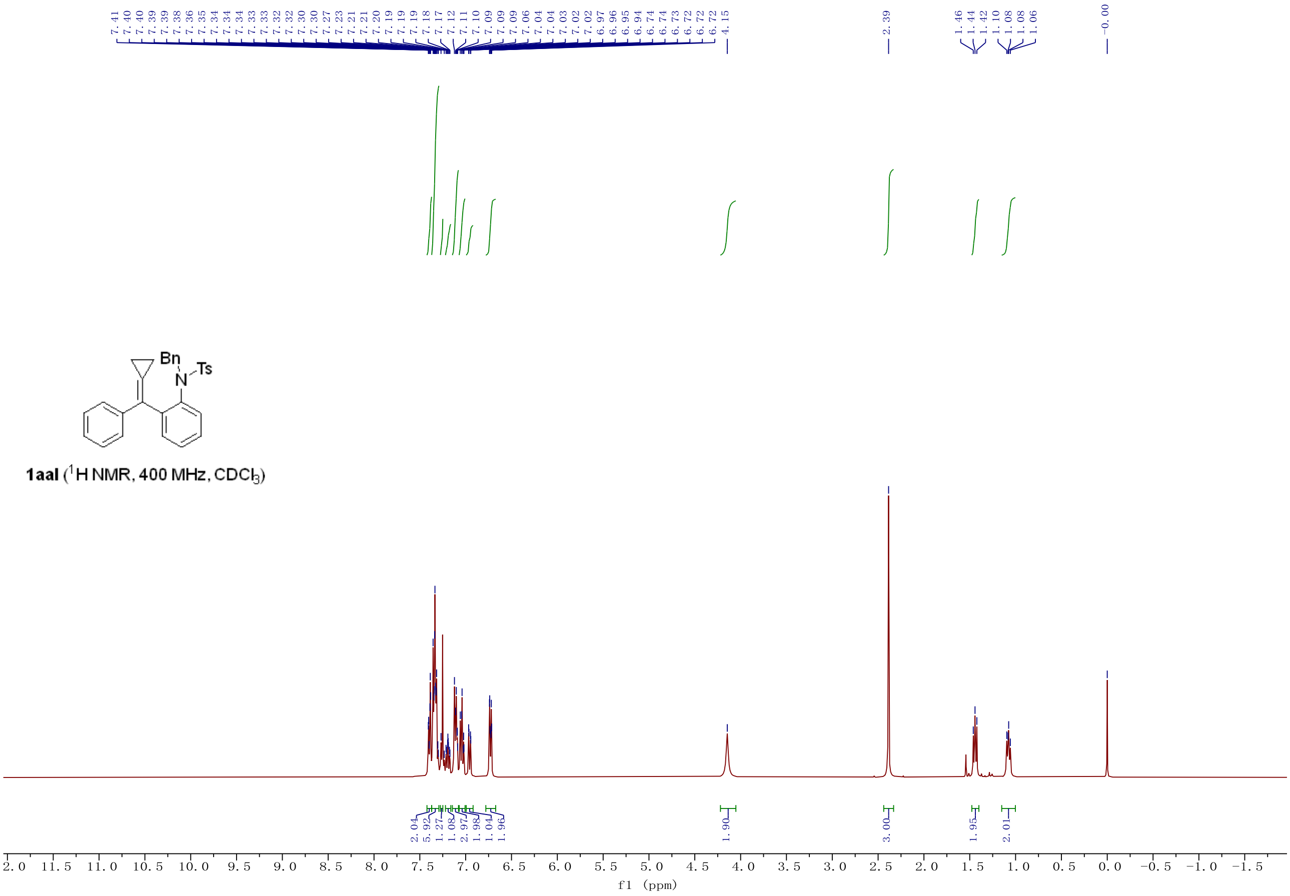

1aal (^{1}H NMR, 400 MHz, $CDCl_3$)

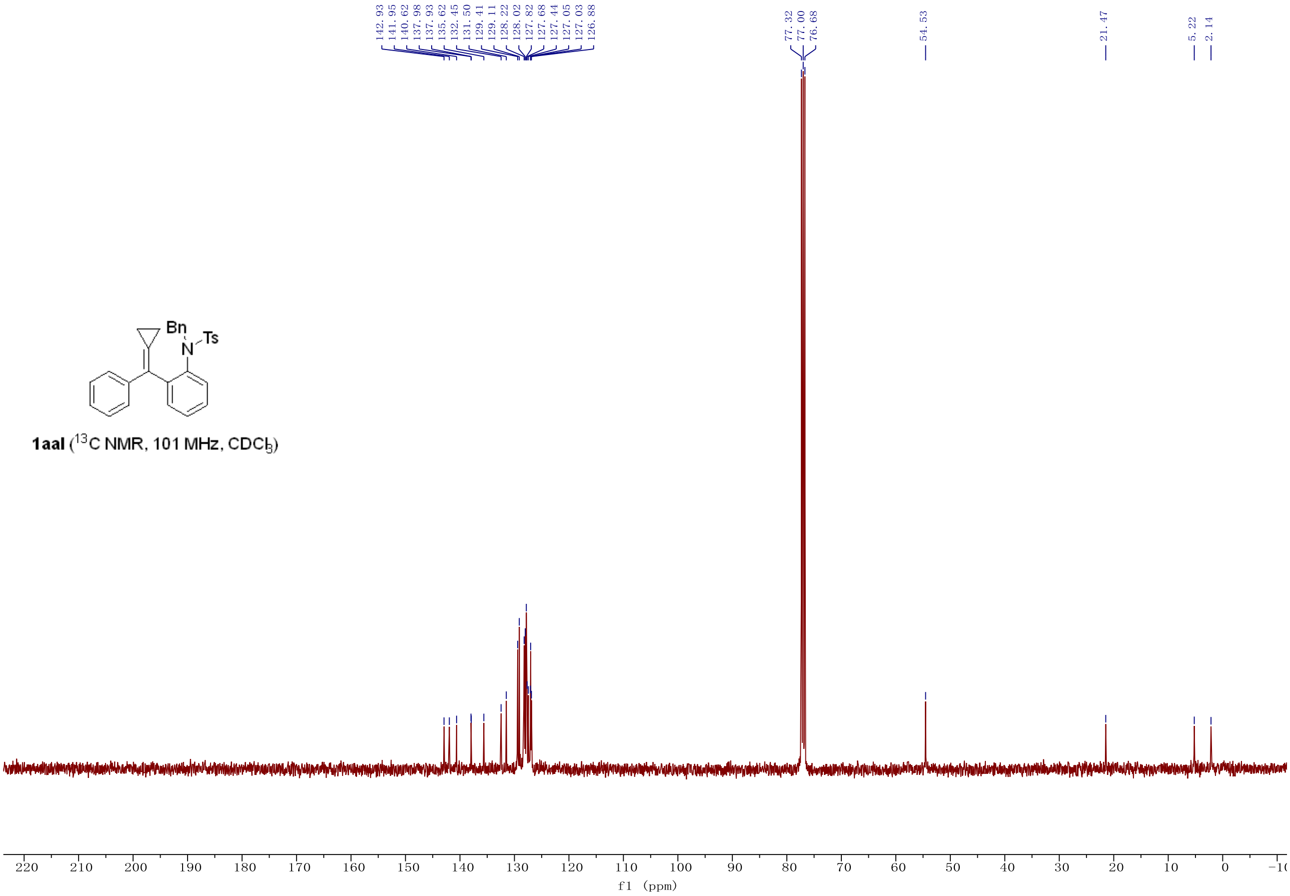

1aal (^{13}C NMR, 101 MHz, $CDCl_3$)

9. Characterization Data of Products.

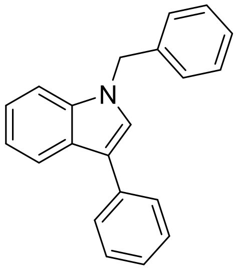

Compound 2a:[11] Yield: 25.5 mg, 90%; a yellow oil; Eluent: PE/EA = 20/1. ^{1}H NMR (400 MHz, $CDCl_3$, TMS) δ 5.30 (s, 2H), 7.14 (d, *J* = 7.2 Hz, 2H), 7.17-7.34 (m, 8H), 7.42 (t, *J* = 7.2 Hz, 2H), 7.66 (d, *J* = 7.6 Hz, 2H), 7.97 (d, *J* = 7.6 Hz, 1H); ^{13}C{^{1}H}-NMR (101 MHz, $CDCl_3$, TMS) δ_C 50.1, 110.0, 117.3, 120.0, 120.1, 122.1, 125.8, 125.9, 126.4, 126.9, 127.3, 127.7, 128.72, 128.78, 135.5, 137.1, 137.2.

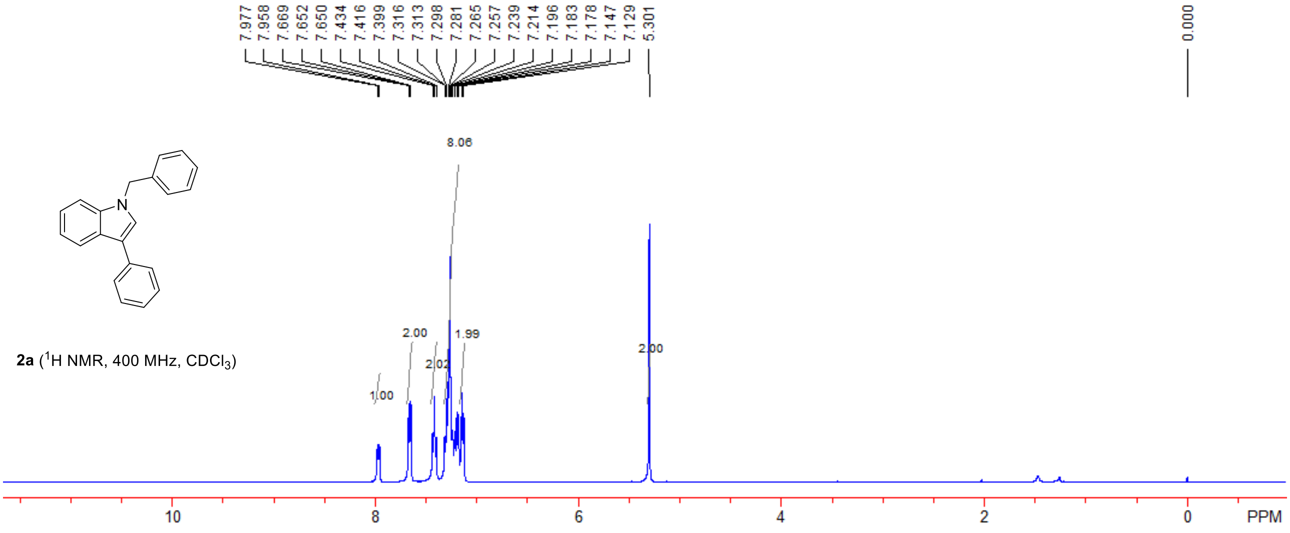

2a (^{1}H NMR, 400 MHz, $CDCl_3$)

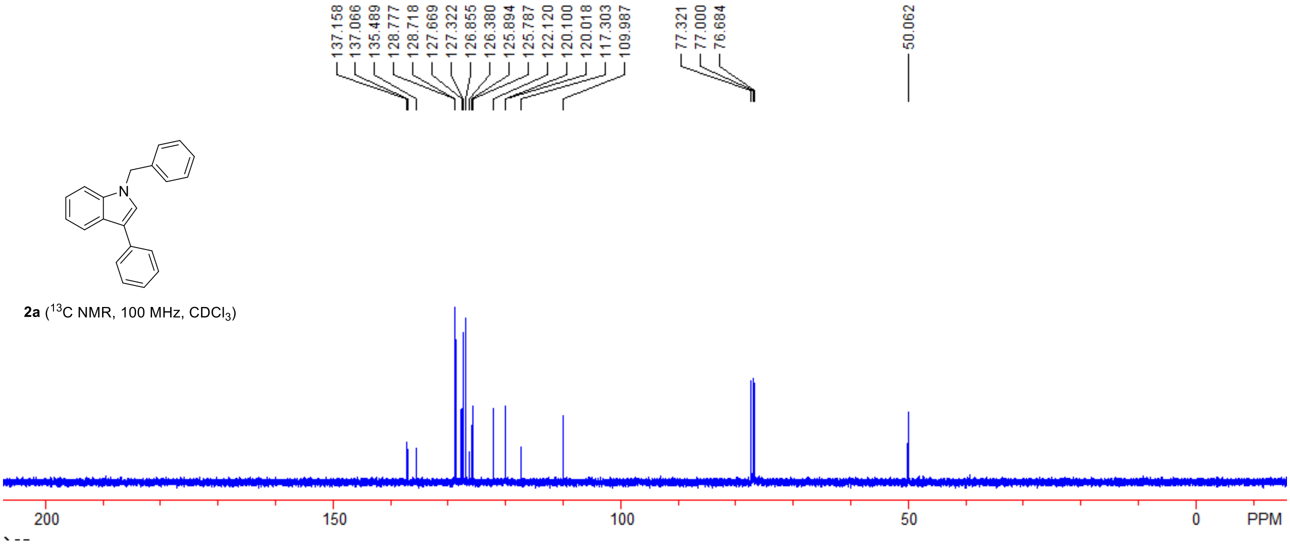

2a (^{13}C NMR, 100 MHz, $CDCl_3$)

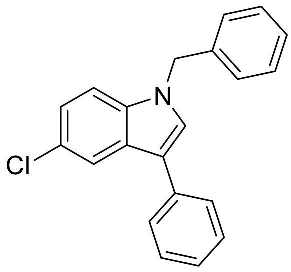

Compound 2c: Yield: 25.7 mg, 81%; a yellow oil; Eluent: PE/EA = 20/1. ^{1}H NMR (400 MHz, $CDCl_3$, TMS) δ 5.27 (s, 2H), 7.08-7.21 (m, 4H), 7.22-7.32 (m, 5H), 7.42 (t, *J* = 7.6 Hz, 2H), 7.59 (d, *J* = 7.6 Hz, 2H),7.89-7.92 (m, 1H); ^{13}C{^{1}H}-NMR (101 MHz, $CDCl_3$, TMS) δ_C 50.3, 111.0, 117.1, 119.5, 122.4, 126.0, 126.1, 126.8, 127.1, 127.3, 127.4, 127.9, 128.8, 128.9, 134.8, 135.4, 136.7; IR (neat): ν 3360, 2959, 2919, 2849, 1632, 1602, 1543, 1494, 1468, 1389, 1352, 1291, 1231, 1174, 1066, 1027, 970, 819, 792, 759, 694 cm^{-1}; HRMS (EI-TOF) Calcd for $C_{21}H_{16}ClN$ $[M]^+$: 317.0966, found: 317.0972.

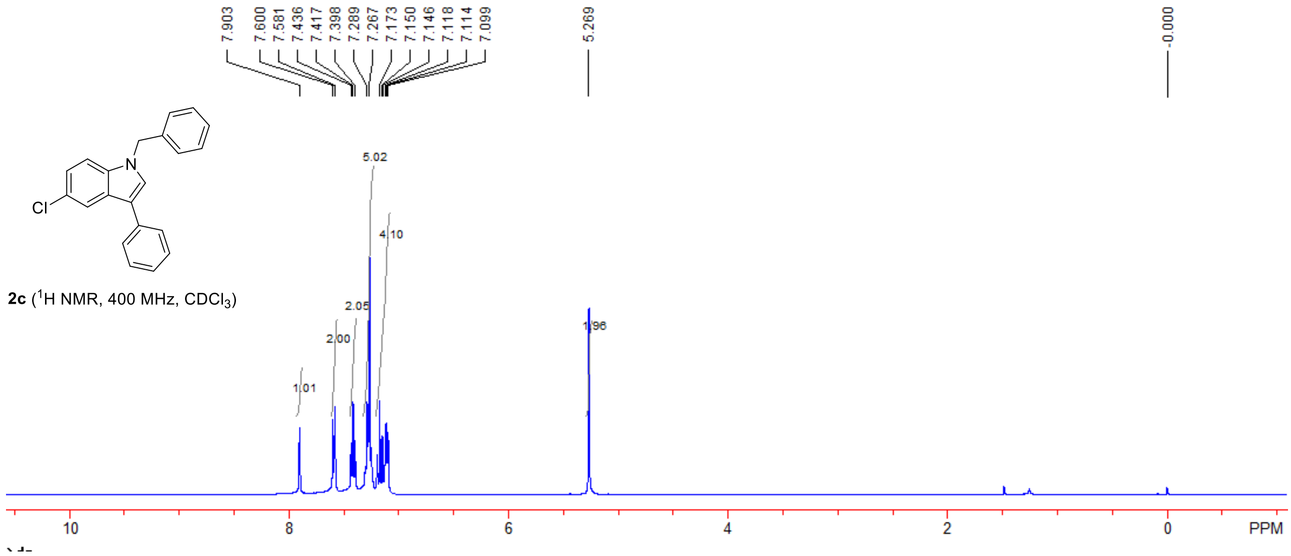

2c (^{1}H NMR, 400 MHz, $CDCl_3$)

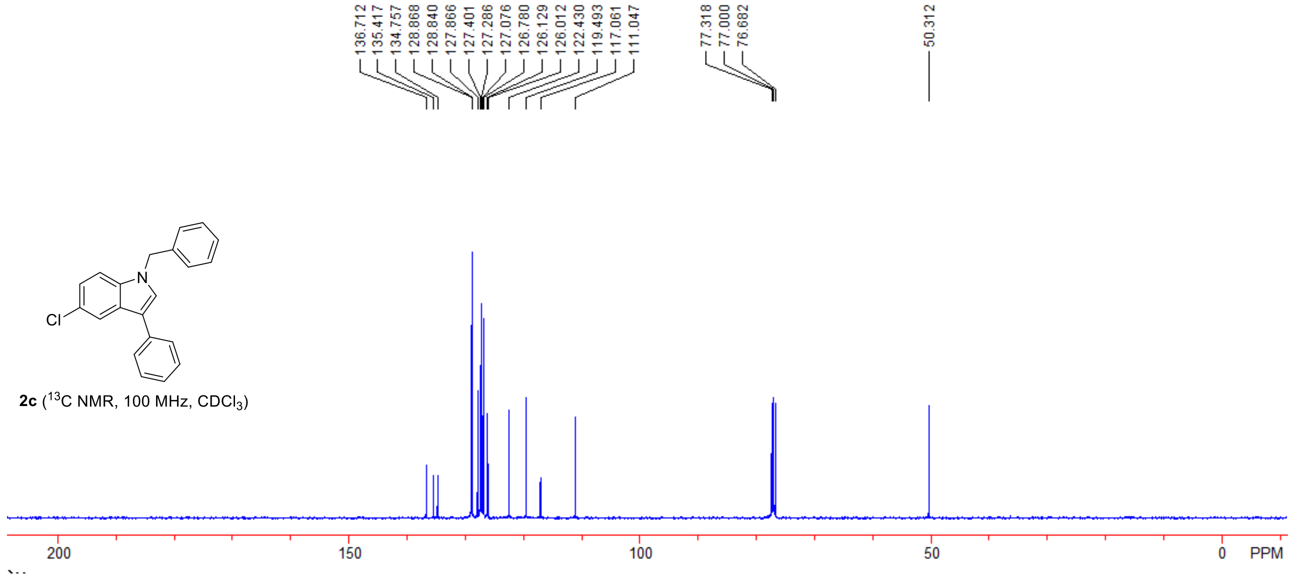

2c (^{13}C NMR, 100 MHz, $CDCl_3$)

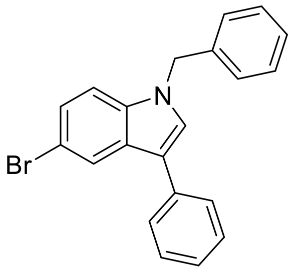

Compound 2d: Yield: 29.2 mg, 81%; a yellow oil; Eluent: PE/EA = 20/1. ^{1}H NMR (400 MHz, $CDCl_3$, TMS) δ 5.20 (s, 2H), 7.04-7.12 (m, 3H), 7.18-7.30 (m, 6H), 7.40 (t, *J* = 7.6 Hz, 2H), 7.57 (d, *J* = 7.6 Hz, 2H), 8.06 (s, 1H); $^{13}C\{^{1}H\}$-NMR (101 MHz, $CDCl_3$, TMS) δ_C 50.2, 111.5, 113.6, 116.9, 122.5, 124.9, 126.1, 126.7, 126.9, 127.3, 127.8, 128.0, 128.8, 134.7, 135.6, 136.6; IR (neat): ν 3057, 3027, 2918, 2850, 1602, 1543, 1494, 1452, 1372, 1309, 1175, 1071, 1027, 969, 867, 790, 731, 694 cm^{-1}; HRMS (EI-TOF) Calcd for $C_{21}H_{16}BrN$ $[M]^{+}$: 361.0461, found: 361.0474.

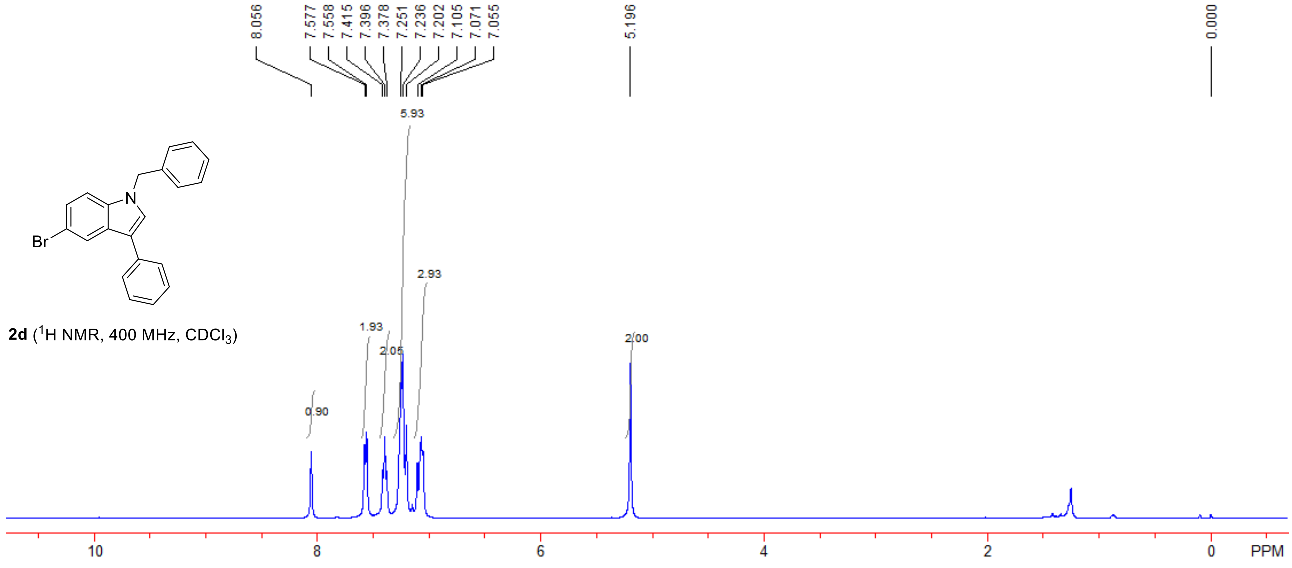

2d (^{1}H NMR, 400 MHz, $CDCl_3$)

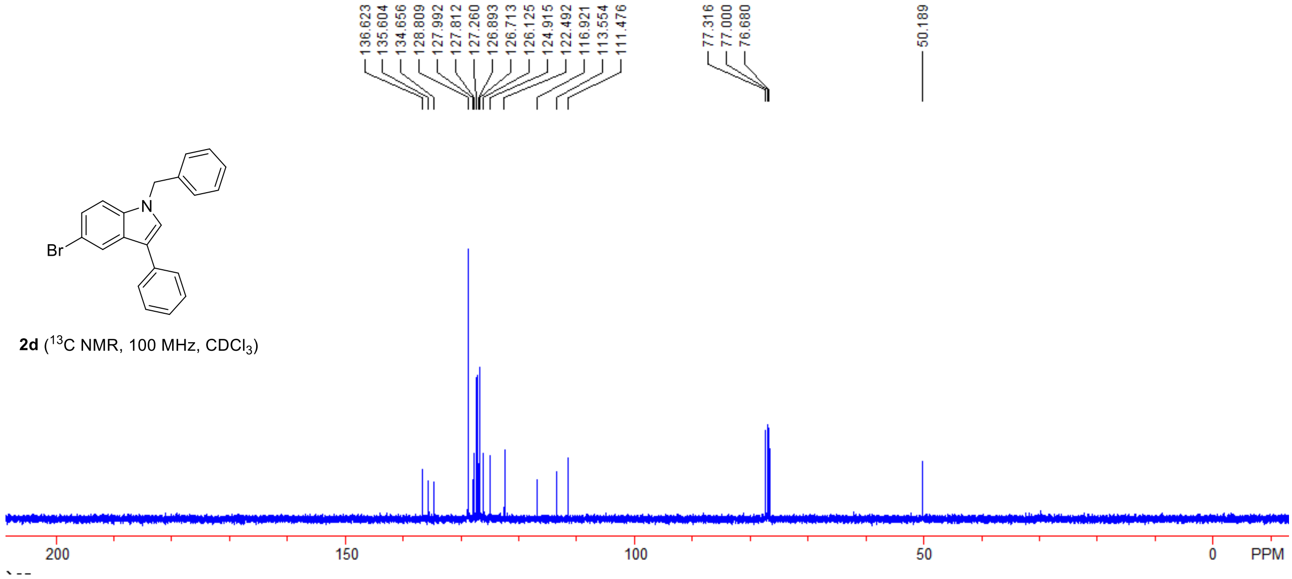

2d (^{13}C NMR, 100 MHz, $CDCl_3$)

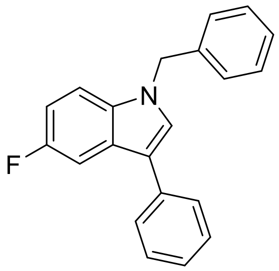

Compound 2e: Yield: 26.5 mg, 88%; a yellow oil; Eluent: PE/EA = 20/1. ^{1}H NMR (400 MHz, $CDCl_3$, TMS) δ 5.27 (s, 2H), 6.98 (dd, J_1 = 8.8 Hz, J_2 = 2.0 Hz, 1H), 7.09-7.14 (m, 2H), 7.15-7.20 (m, 1H), 7.22-7.32 (m, 5H), 7.41 (t, J = 8.0 Hz, 2H), 7.57-7.62 (m, 3H); ^{13}C{^{1}H}-NMR (101 MHz, $CDCl_3$, TMS) δ_C 50.4, 105.0 (d, J = 24.1 Hz), 110.5 (d, J = 26.2 Hz), 110.7 (d, J = 9.6 Hz), 117.2 (d, J = 4.8 Hz), 126.0, 126.6 (d, J = 9.7 Hz), 126.8, 127.1, 127.4, 127.8, 128.8 (d, J = 1.6 Hz), 133.6, 135.0, 136.8, 158.4 (d, J = 233.7 Hz); ^{19}F NMR (376 MHz, $CDCl_3$) δ -124.1; IR (neat): ν 3061, 3028, 2918, 1621, 1601, 1544, 1480, 1441, 1354, 1253, 1176, 1104, 1072, 1001, 965, 871, 778, 735, 694 cm^{-1}; HRMS (EI-TOF) Calcd for $C_{21}H_{16}FN$ $[M]^+$: 301.1261, found: 301.1271.

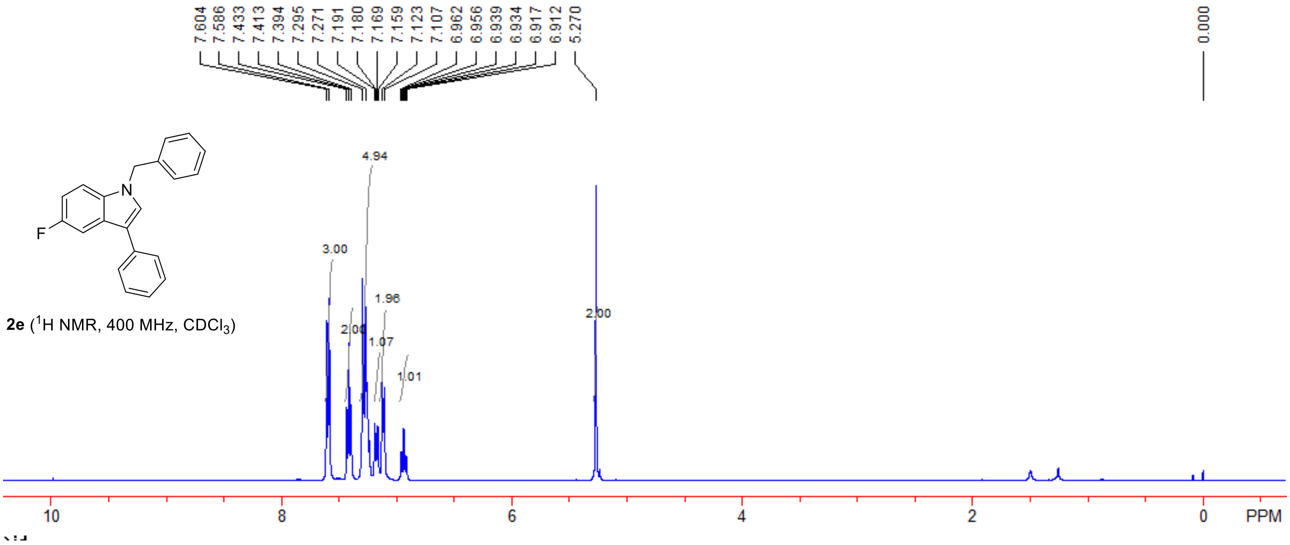

2e (^{1}H NMR, 400 MHz, $CDCl_3$)

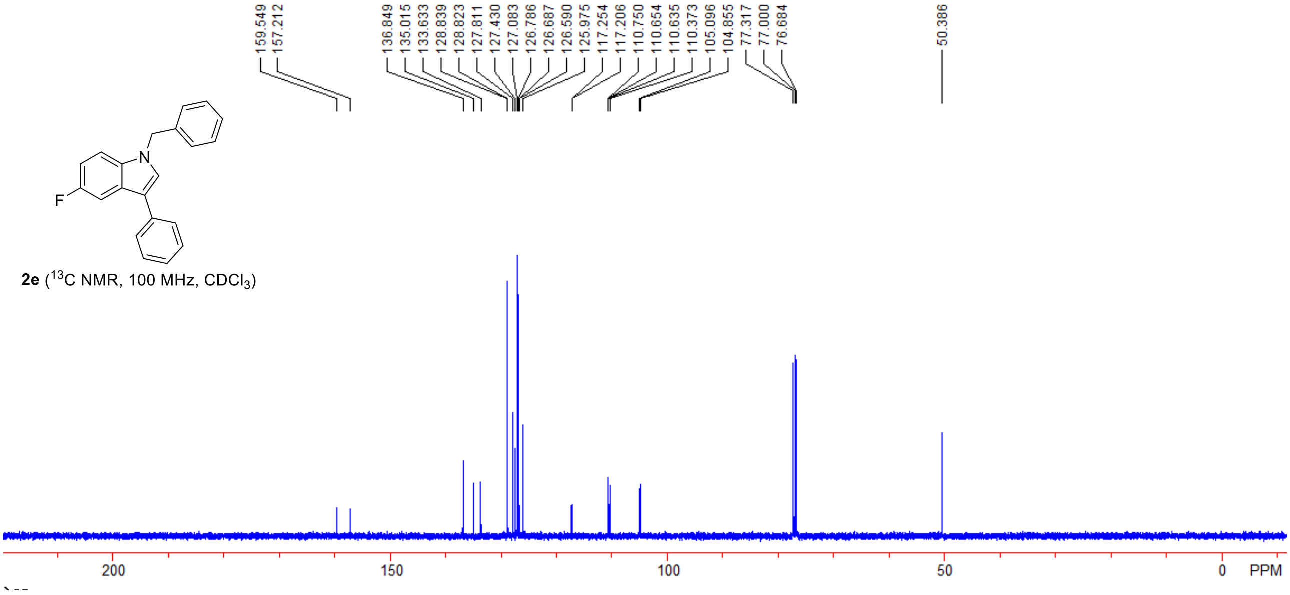

2e (^{13}C NMR, 100 MHz, $CDCl_3$)

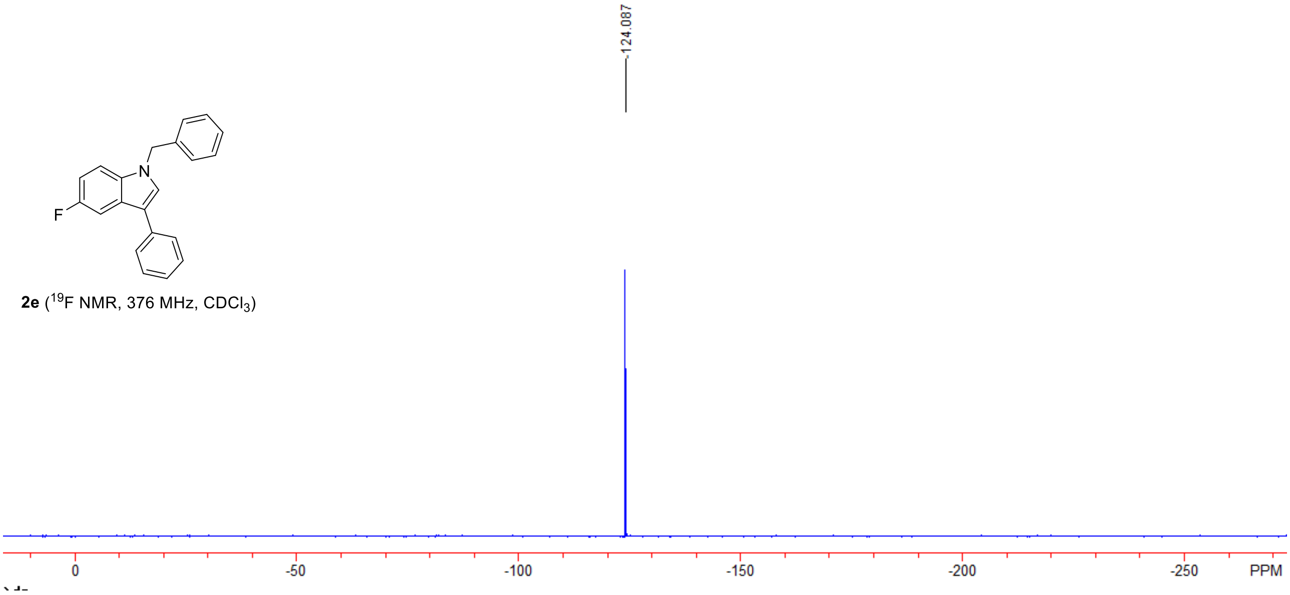

2e (^{19}F NMR, 376 MHz, $CDCl_3$)

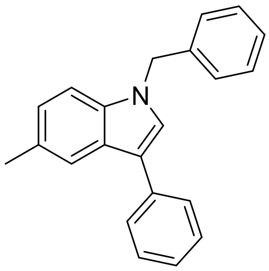

Compound 2f: Yield: 22.3 mg, 75%; a light yellow solid; Mp: 89-91 ºC; Eluent: PE/EA = 20/1. ^{1}H NMR (400 MHz, $CDCl_3$, TMS) δ 2.46 (s, 3H), 5.26 (s, 2H), 7.03 (d, *J* = 7.6 Hz, 1H), 7.12 (d, *J* = 7.2 Hz, 2H), 7.15-7.30 (m, 6H), 7.38-7.46 (m, 2H), 7.62-7.68 (m, 2H), 7.73-7.78 (m, 1H); ^{13}C{^{1}H}-NMR (101 MHz, $CDCl_3$, TMS) δ_C 21.6, 50.1, 109.7, 116.7, 119.6, 123.7, 125.7, 126.0, 126.6, 126.8, 127.3, 127.6, 128.68, 128.73, 129.4, 135.4, 135.7, 137.3; IR (neat): ν 3026, 2918, 2849, 1600, 1541, 1483, 1435, 1378, 1352, 1238, 1197, 1139, 1072, 1027, 863, 791, 761, 738, 696 cm^{-1}; HRMS (EI-TOF) Calcd for $C_{22}H_{19}N$ [M]$^+$: 297.1512, found: 297.1519.

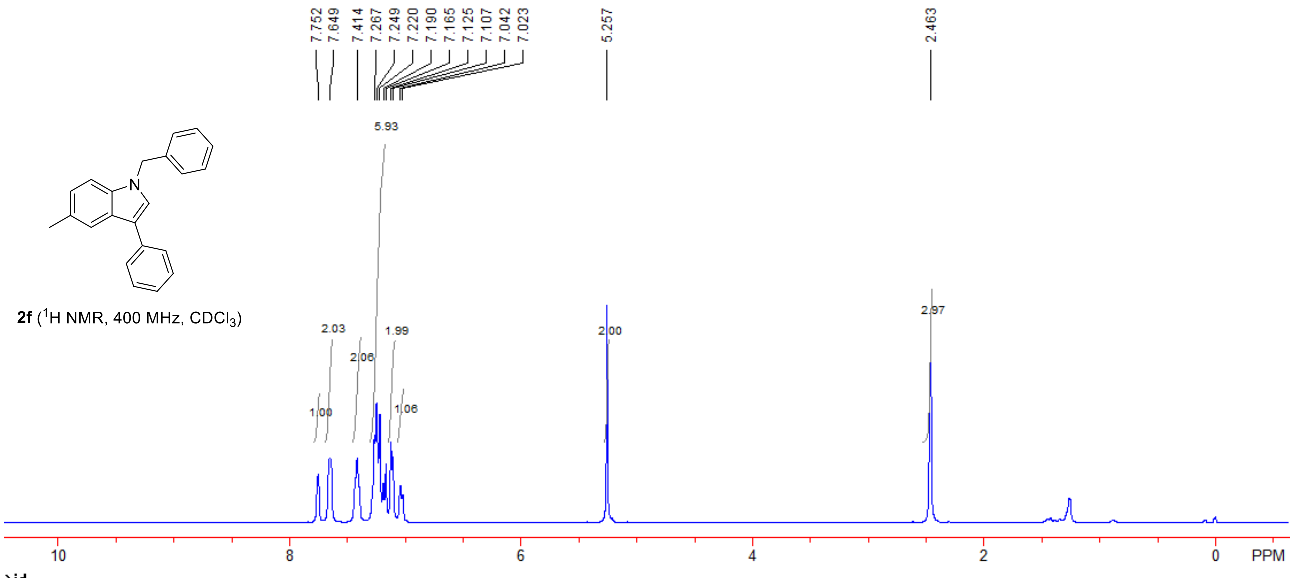

2f (^{1}H NMR, 400 MHz, $CDCl_3$)

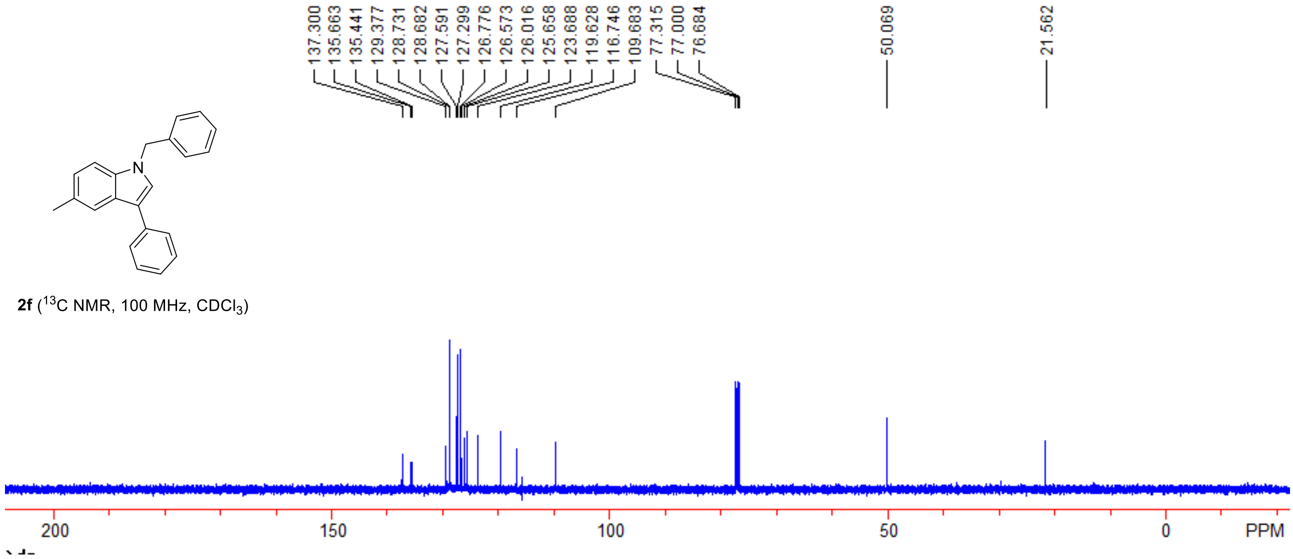

2f (^{13}C NMR, 100 MHz, $CDCl_3$)

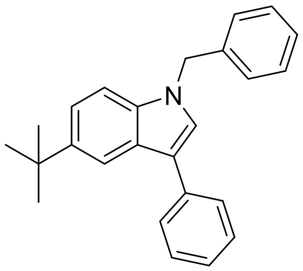

Compound 2g:[12] Yield: 26.8 mg, 79%; a light yellow solid; Mp: 72-74 °C; Eluent: PE/EA = 20/1. ^{1}H NMR (400 MHz, $CDCl_3$, TMS) δ 1.39 (s, 9H), 5.27 (s, 2H), 7.16 (d, *J* = 6.0 Hz, 2H), 7.20-7.34 (m, 7H), 7.43 (t, *J* = 7.2 Hz, 2H), 7.66 (d, *J* = 7.2 Hz, 2H), 7.95 (s, 1H); ^{13}C{^{1}H}-NMR (101 MHz, $CDCl_3$, TMS) δ_C 31.9, 34.7, 50.1, 109.5, 115.6, 117.3, 120.4, 125.7, 126.0, 126.1, 127.0, 127.4, 127.6, 128.7, 135.3, 135.8, 137.3, 143.0.

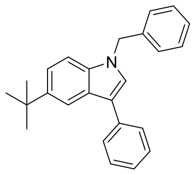

2g (^{1}H NMR, 400 MHz, $CDCl_3$)

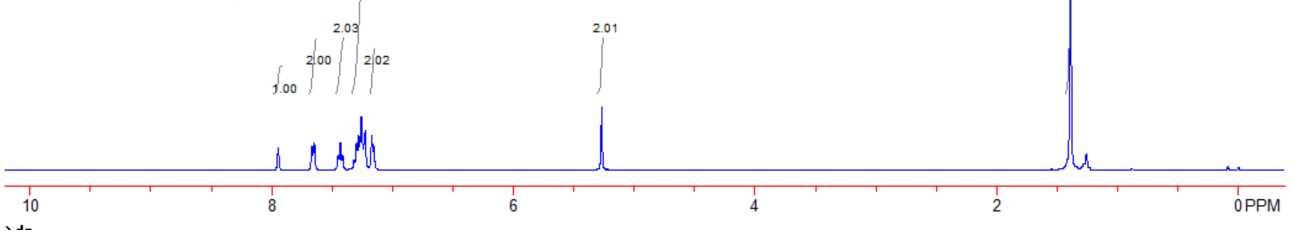

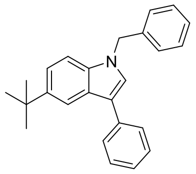

2g (^{13}C NMR, 100 MHz, $CDCl_3$)

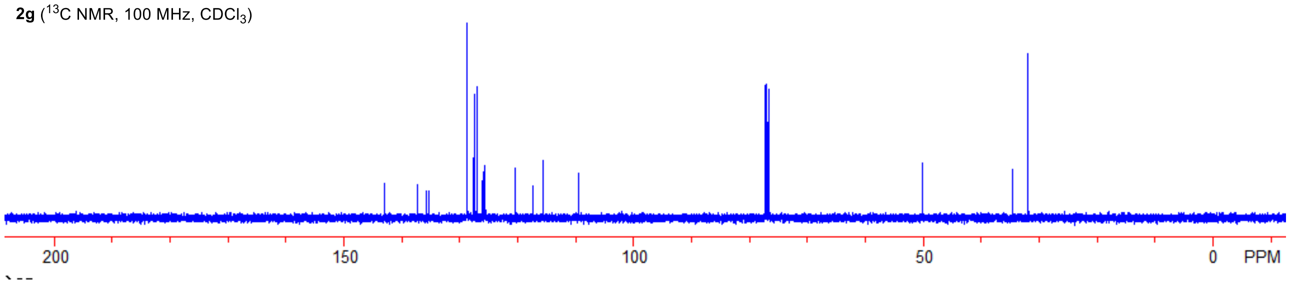

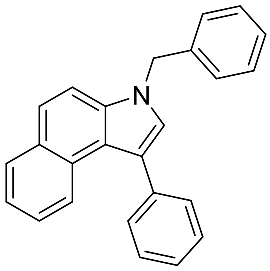

Compound 2h: Yield: 26.3 mg, 79%; a light yellow solid; Mp: 141-143 ^{o}C; Eluent: PE/EA = 20/1. ^{1}H NMR (400 MHz, $CDCl_3$, TMS) δ 5.38 (s, 2H), 7.08 (s, 1H), 7.12 (d, *J* = 6.8 Hz, 2H), 7.21-7.50 (m, 9H), 7.57 (d, *J* = 8.4 Hz, 1H), 7.62 (d, *J* = 6.8 Hz, 2H), 7.85 (d, *J* = 6.8 Hz, 1H), 8.15 (d, *J* = 6.4 Hz, 1H); ^{13}C{^{1}H}-NMR (101 MHz, $CDCl_3$, TMS) δ_C 50.2, 111.4, 120.2, 120.3, 123.2, 123.3, 123.5, 125.4, 125.8, 126.7, 126.8, 127.7, 128.3, 128.7, 128.8, 129.6, 130.1, 133.3, 137.1, 137.3; IR (neat): ν 3061, 3025, 2920, 2850, 1603, 1593, 1540, 1495, 1438, 1359, 1300, 1200, 1160, 1070, 971, 830, 716 cm^{-1}; HRMS (EI-TOF) Calcd for $C_{25}H_{19}N$ $[M]^{+}$: 333.1512, found: 333.1524.

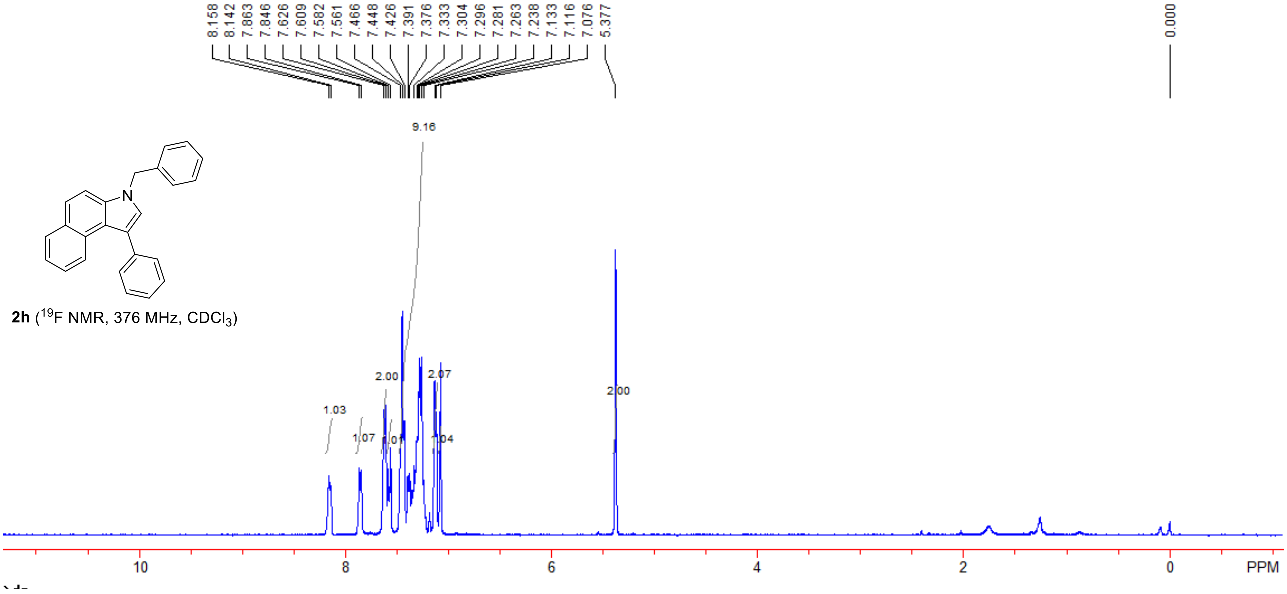

2h (^{19}F NMR, 376 MHz, $CDCl_3$)

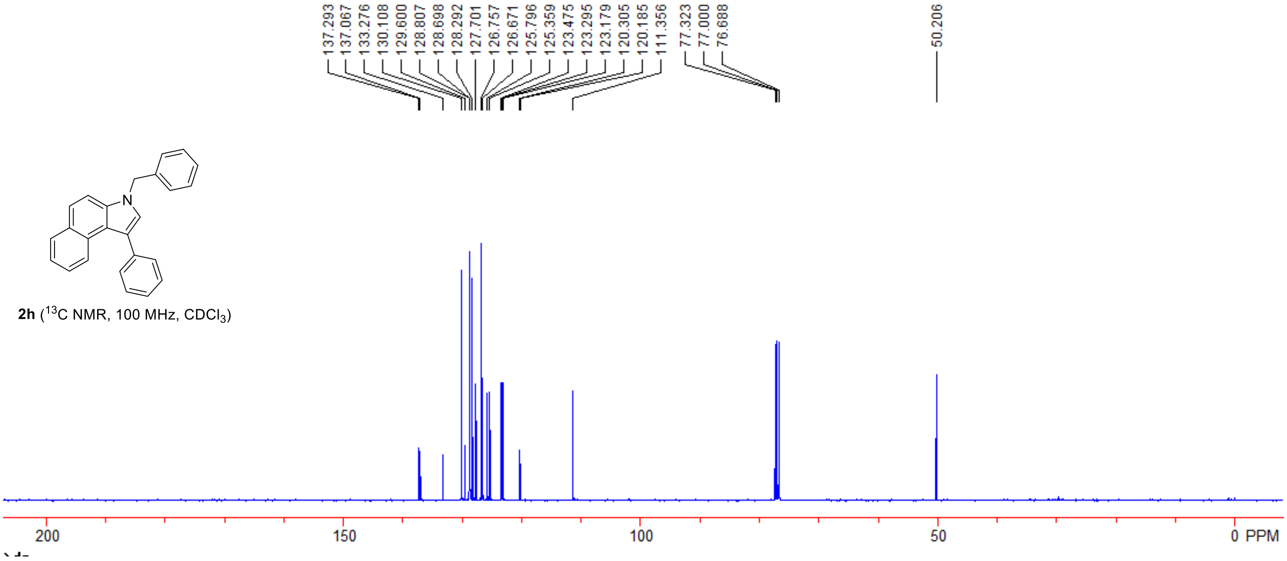

2h (^{13}C NMR, 100 MHz, $CDCl_3$)

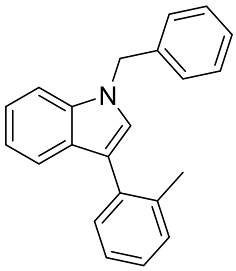

Compound 2i: Yield: 25.3 mg, 85%; a yellow oil; Eluent: PE/EA = 20/1. ^{1}H NMR (400 MHz, $CDCl_3$, TMS) δ 2.33 (s, 3H), 5.28 (s, 2H), 7.05-7.15 (m, 4H), 7.16-7.35 (m, 8H), 7.40-7.50 (m, 1H), 7.51-7.60 (m, 1H); ^{13}C{^{1}H}-NMR (101 MHz, $CDCl_3$, TMS) δ_C 20.8, 50.0, 109.8, 116.5, 119.6, 120.3, 121.8, 125.6, 126.6, 126.7, 126.9, 127.6, 127.8, 128.7, 130.3, 130.8, 134.3, 136.3, 136.6, 137.4; IR (neat): ν 3027, 2919, 1602, 1548, 1463, 1452, 1329, 1174, 937, 769, 724, 695 cm^{-1}; HRMS (ESI-TOF) Calcd for $C_{22}H_{20}N$ $[M+H]^+$: 298.1590, found: 298.1595.

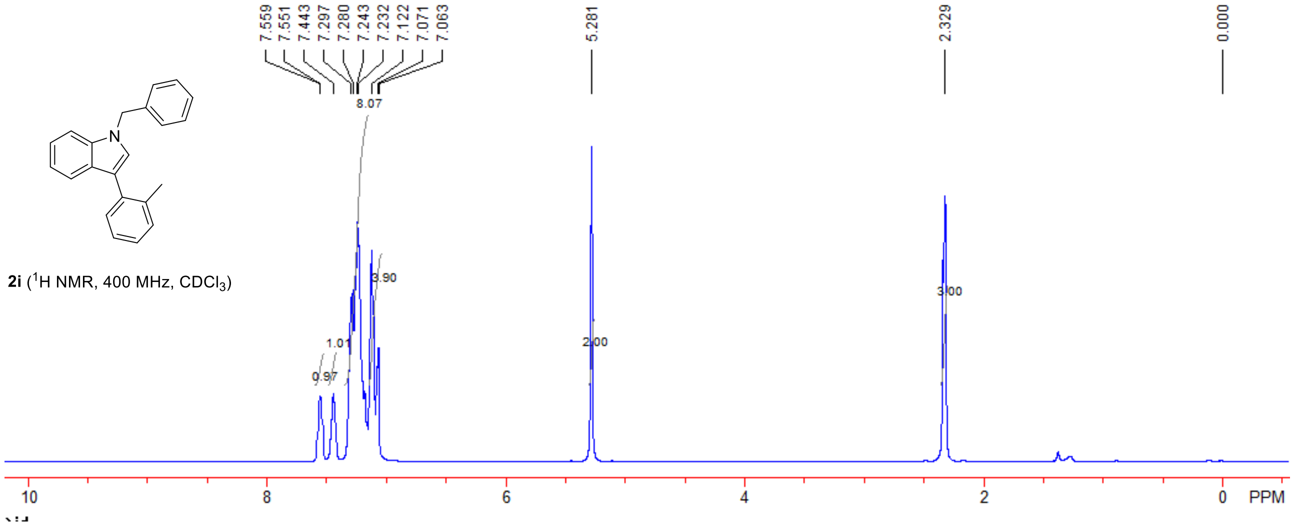
7.559
7.551
7.443
7.297
7.280
7.243
7.232
7.122
7.071
7.063
5.281
2.329
0.000
8.07
3.90
1.01
0.97
2.00
3.00
N
2i (¹H NMR, 400 MHz, CDCl₃)
10
8
6
4
2
0
PPM

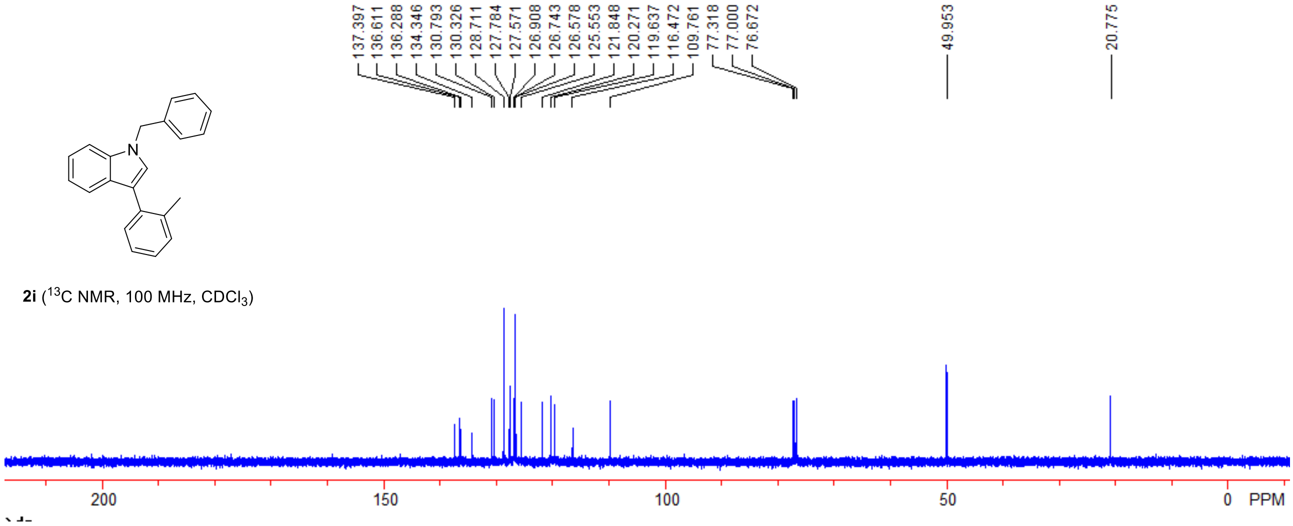
137.397
136.611
136.288
134.346
130.793
130.326
128.711
127.784
127.571
126.908
126.743
126.578
125.553
121.848
120.271
119.637
116.472
109.761
77.318
77.000
76.672
49.953
20.775
N
2i (¹³C NMR, 100 MHz, CDCl₃)
200
150
100
50
0
PPM

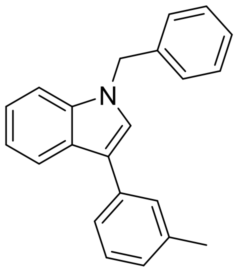

Compound 2j: Yield: 24.7 mg, 83%; a light yellow solid; Mp: 86-88 ºC; Eluent: PE/EA = 20/1. ^{1}H NMR (400 MHz, $CDCl_3$, TMS) δ 2.37 (s, 3H), 5.17 (s, 2H), 7.02-7.08 (m, 3H), 7.13-7.24 (m, 7H), 7.28 (t, *J* = 7.6 Hz, 1H), 7.43-7.48 (m, 2H), 7.94-7.99 (m, 1H); ^{13}C{^{1}H}-NMR (101 MHz, $CDCl_3$, TMS) δ_C 21.5, 49.9, 109.9, 117.3, 120.0, 120.1, 122.0, 124.4, 125.9, 126.4, 126.6, 126.8, 127.6, 128.0, 128.6, 128.7, 135.4, 137.0, 137.2, 138.2; IR (neat): ν 3023, 2916, 1602, 1538, 1494, 1391, 1189, 1072, 959, 830, 779, 729, 694 cm^{-1}; HRMS (EI-TOF) Calcd for $C_{22}H_{19}N$ $[M]^+$: 297.1512, found: 297.1524.

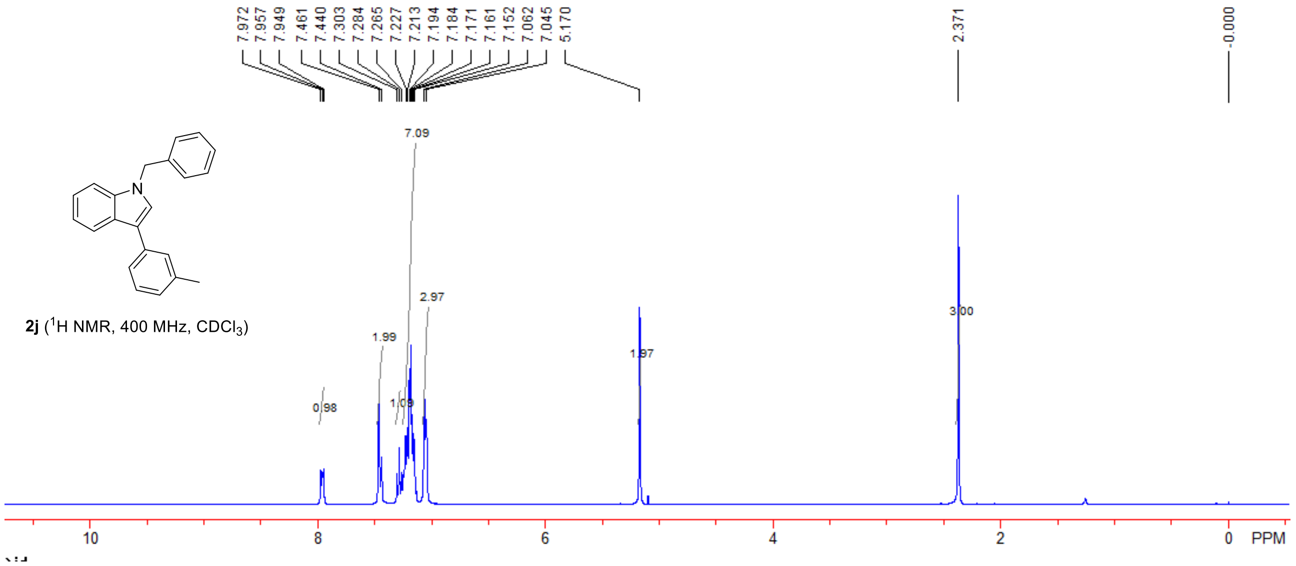

2j (^{1}H NMR, 400 MHz, $CDCl_3$)

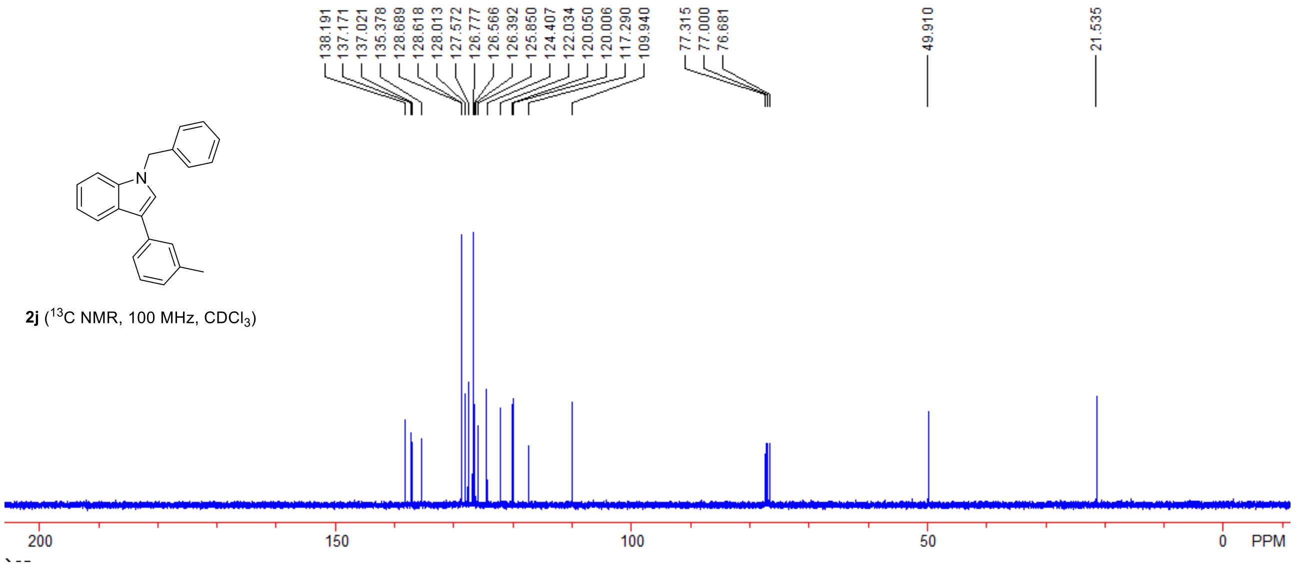

2j (^{13}C NMR, 100 MHz, $CDCl_3$)

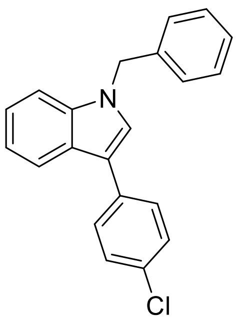

Compound 2k: Yield: 26.0 mg, 82%; a light yellow solid; Mp: 92-94 ºC; Eluent: PE/EA = 20/1. ^{1}H NMR (400 MHz, $CDCl_3$, TMS) δ 5.19 (s, 2H), 7.07 (d, *J* = 6.4 Hz, 2H), 7.12-7.19 (m, 3H), 7.20-7.28 (m, 4H), 7.33 (d, *J* = 8.4 Hz, 2H), 7.52 (d, *J* = 8.4 Hz, 2H), 7.87 (d, *J* = 8.0 Hz, 1H); ^{13}C{^{1}H}-NMR (101 MHz, $CDCl_3$, TMS) δ_C 50.0, 110.1, 116.0, 119.7, 120.3, 122.3, 125.9, 126.1, 126.8, 127.7, 128.4, 128.7, 128.8, 131.3, 134.0, 136.95, 137.02; IR (neat): ν 3027, 2922, 2852, 1593, 1556, 1491, 1467, 1410, 1355, 1296, 1202, 1139, 1020, 969, 827, 763, 716 cm^{-1}; HRMS (EI-TOF) Calcd for $C_{21}H_{16}ClN$ $[M]^+$: 317.0966, found: 317.0976.

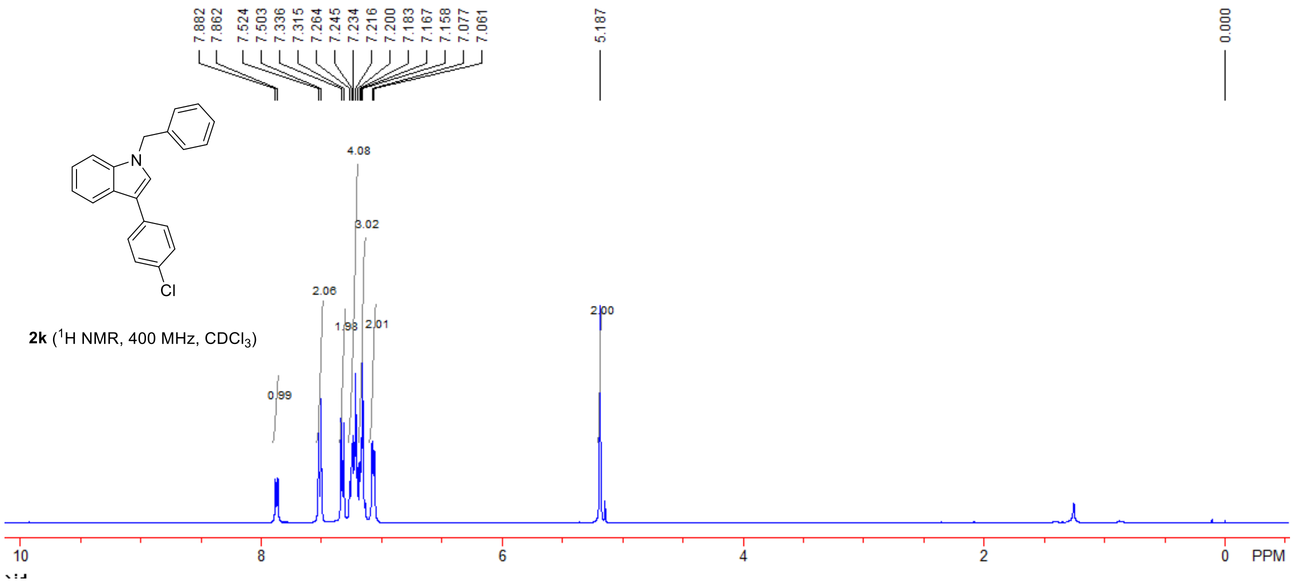

2k (^{1}H NMR, 400 MHz, $CDCl_3$)

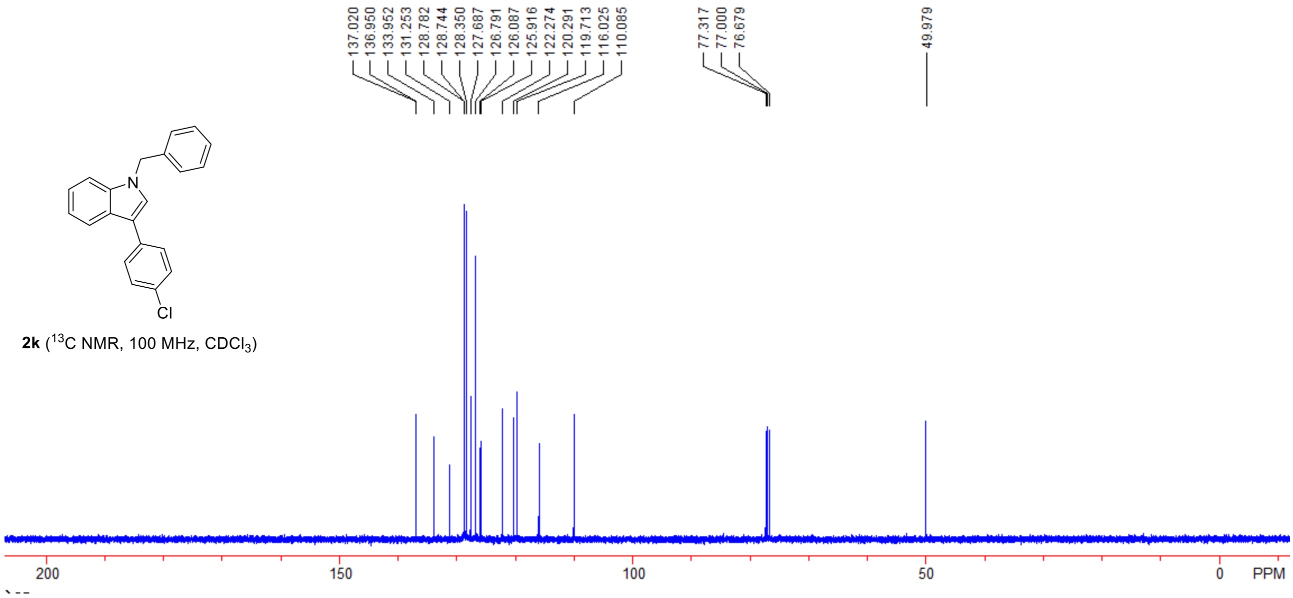

2k (^{13}C NMR, 100 MHz, $CDCl_3$)

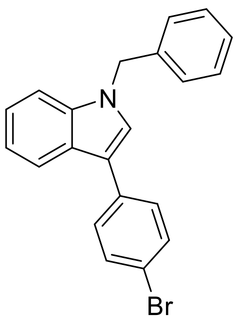

Compound 2l: Yield: 30.0 mg, 83%; a light yellow solid; Mp: 103-105 °C; Eluent: PE/EA = 20/1. ^{1}H NMR (400 MHz, $CDCl_3$, TMS) δ 5.28 (s, 2H), 7.13 (t, *J* = 6.8 Hz, 2H), 7.15-7.33 (m, 7H), 7.47-7.54 (m, 4H), 7.89 (d, *J* = 7.6 Hz, 1H); $^{13}C\{^{1}H\}$-NMR (101 MHz, $CDCl_3$, TMS) δ_C 50.1, 110.1, 116.1, 119.3, 119.7, 120.3, 122.3, 125.9, 126.1, 126.8, 127.7, 128.7, 128.8, 131.7, 134.4, 136.9, 137.1; IR (neat): ν 2988, 2965, 2908, 2885, 1587, 1494, 1441, 1378, 1332, 1264, 1199, 1140, 1099, 1029, 950, 832, 785, 727, 698 cm^{-1}; HRMS (EI-TOF) Calcd for $C_{21}H_{16}BrN$ $[M]^+$: 361.0461, found: 361.0472.

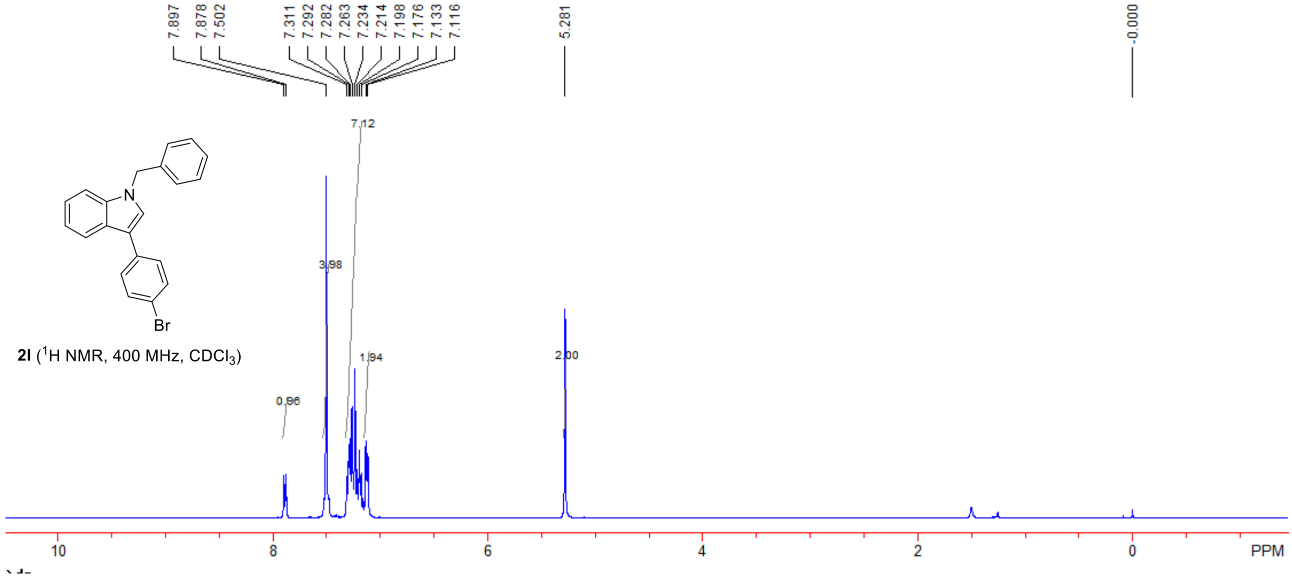

2l (^{1}H NMR, 400 MHz, $CDCl_3$)

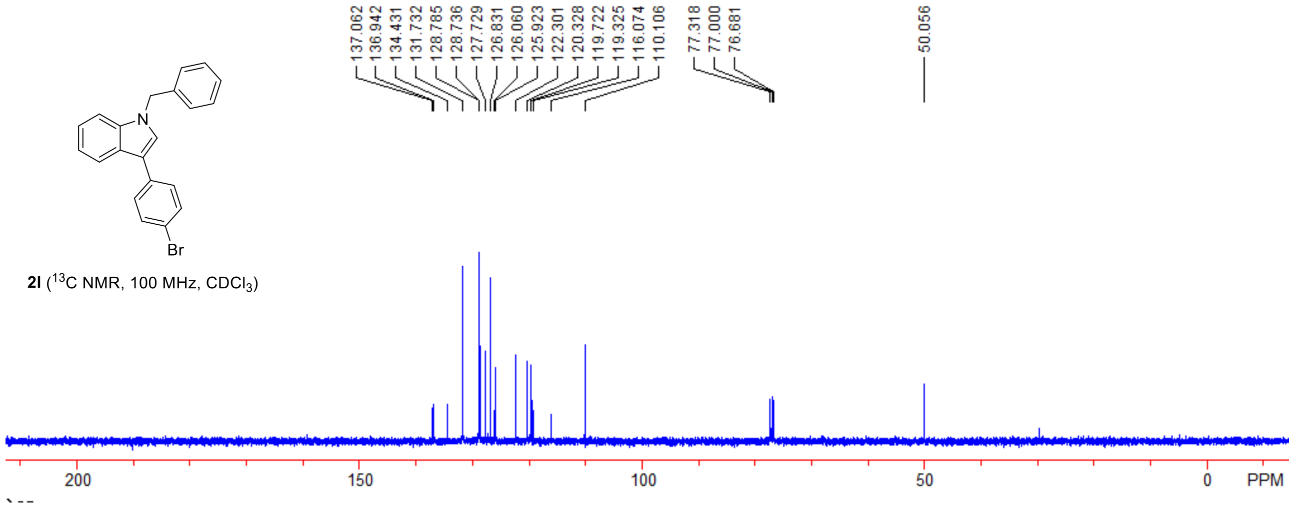

2l (^{13}C NMR, 100 MHz, $CDCl_3$)

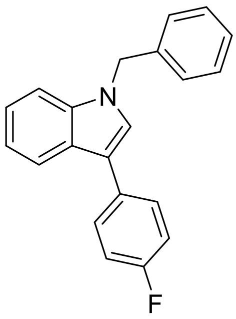

Compound 2m: Yield: 22.9 mg, 76%; a light yellow solid; Mp: 106-108 °C; Eluent: PE/EA = 20/1. ^{1}H NMR (400 MHz, $CDCl_3$, TMS) δ 5.20 (s, 2H), 7.02-7.11 (m, 4H), 7.12-7.29 (m, 7H), 7.50-7.58 (m, 2H), 7.87 (d, J = 6.4 Hz, 1H); ^{13}C{^{1}H}-NMR (101 MHz, $CDCl_3$, TMS) δ_C 50.0, 110.0, 115.5 (d, J = 21.5 Hz), 116.3, 119.7, 120.2, 122.2, 125.7, 126.3, 126.8, 127.7, 128.7 (d, J = 8.9 Hz), 128.8, 131.5 (d, J = 2.9 Hz), 137.0, 137.1, 161.3 (d, J = 242.8 Hz); ^{19}F NMR (376 MHz, $CDCl_3$) δ -116.9; IR (neat): ν 2924, 1543, 1503, 1468, 1436, 1356, 1333, 1226, 1187, 1156, 1097, 1070, 968, 835, 809, 728, 695 cm^{-1}; HRMS (EI-TOF) Calcd for $C_{21}H_{16}FN$ $[M]^+$: 301.1261, found: 301.1270.

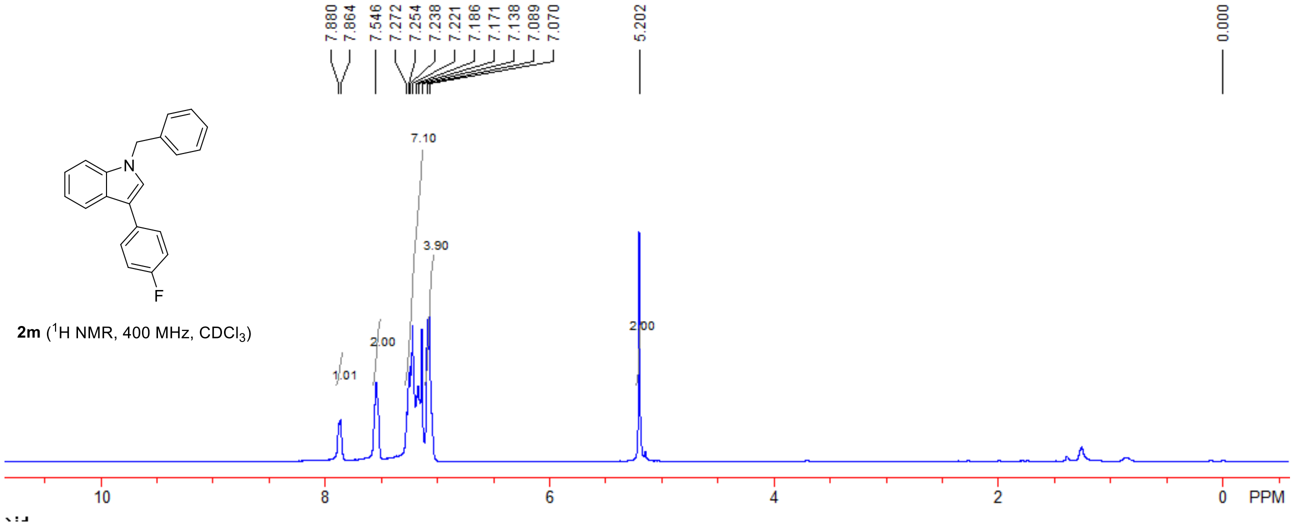

2m (^{1}H NMR, 400 MHz, $CDCl_3$)

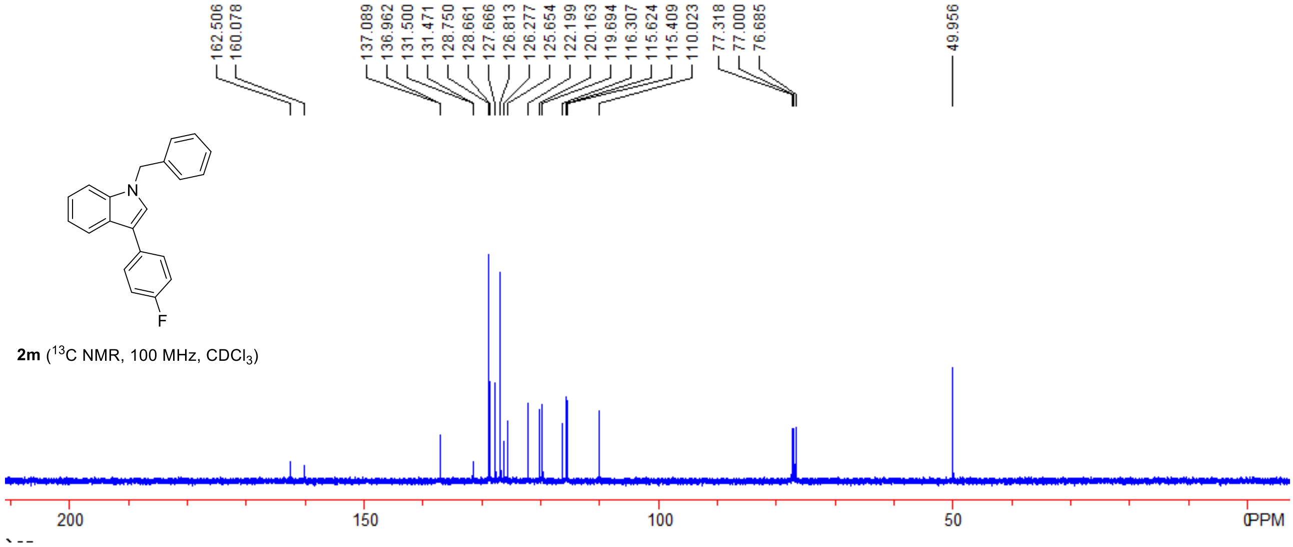

2m (^{13}C NMR, 100 MHz, $CDCl_3$)

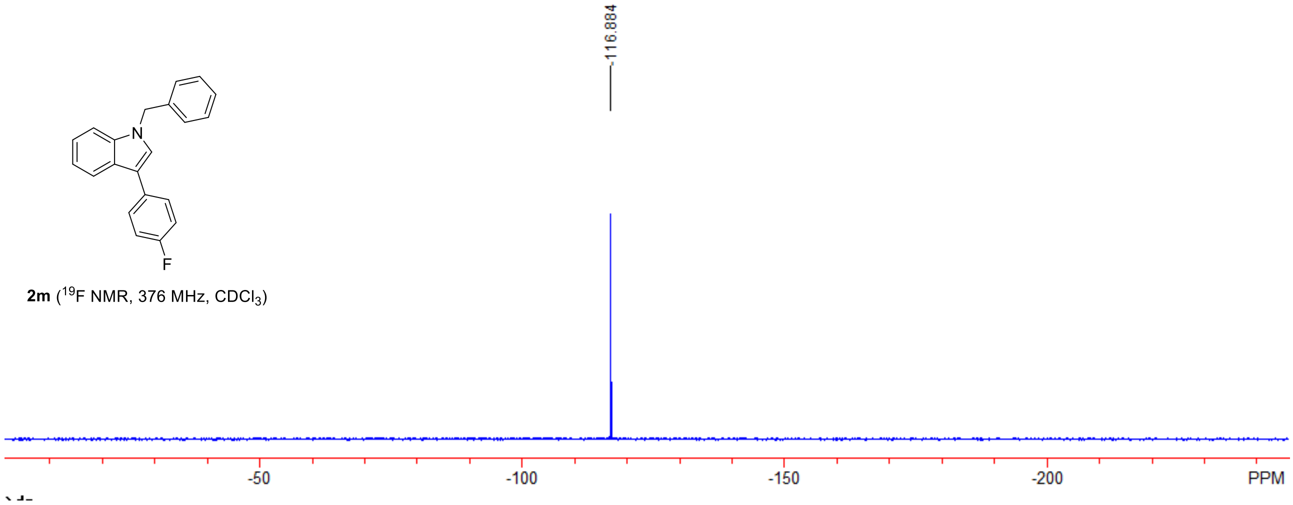

2m (^{19}F NMR, 376 MHz, $CDCl_3$)

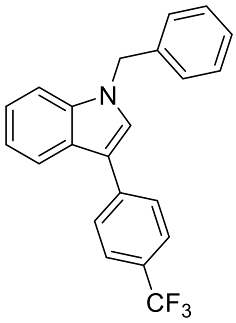

Compound 2n: Yield: 27.4 mg, 78%; a light yellow solid; Mp: 92-94 ºC; Eluent: PE/EA = 20/1. ^{1}H NMR (400 MHz, $CDCl_3$, TMS) δ 5.32 (s, 2H), 7.15 (d, *J* = 6.8 Hz, 2H), 7.19-7.37 (m, 7H), 7.65 (d, *J* = 8.0 Hz, 2H), 7.74 (d, *J* = 8.0 Hz, 2H), 7.94 (d, *J* = 7.6 Hz, 1H); ^{13}C{^{1}H}-NMR (101 MHz, $CDCl_3$, TMS) δ_C 50.2, 110.3, 116.0, 119.8, 120.6, 122.5, 124.5 (q, *J* = 270.3 Hz), 125.7 (q, *J* = 4.0 Hz), 126.1, 126.6, 126.9, 127.1, 127.5 (q, *J* = 32.2 Hz), 127.9, 128.9, 136.8, 137.2, 139.3; ^{19}F NMR (376 MHz, $CDCl_3$) δ -62.1; IR (neat): ν 3028, 2920, 2850, 1614, 1544, 1509, 1495, 1454, 1388, 1324, 1237, 1158, 1104, 1027, 966, 838, 785, 728, 696 cm^{-1}; HRMS (EI-TOF) Calcd for $C_{22}H_{16}F_3N$ $[M]^+$: 351.1229, found: 351.1243.

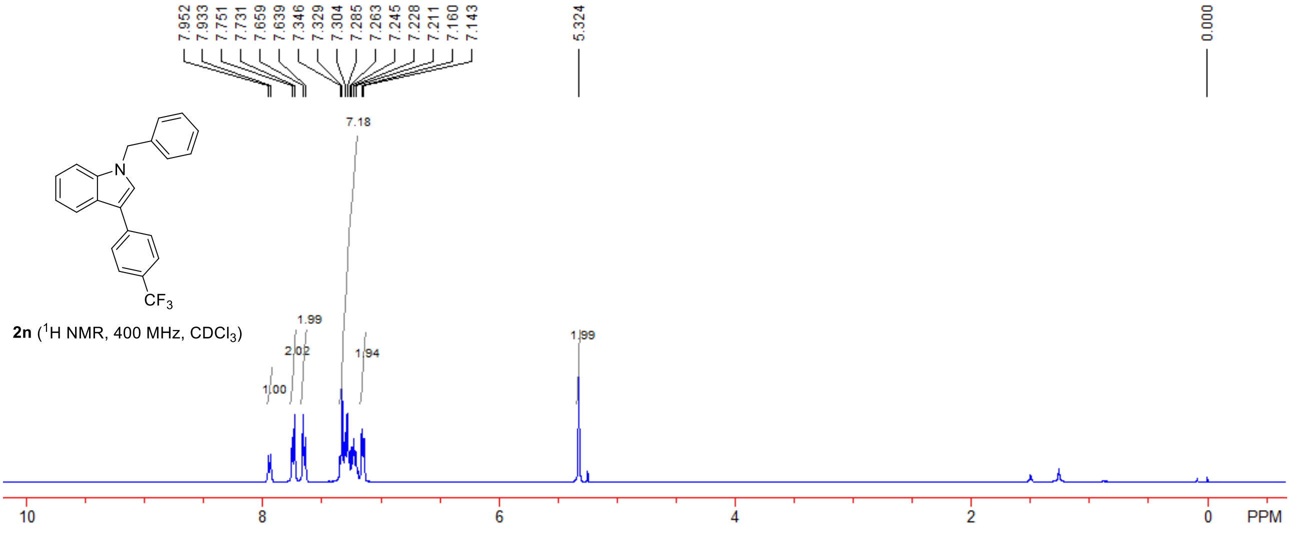

2n (^{1}H NMR, 400 MHz, $CDCl_3$)

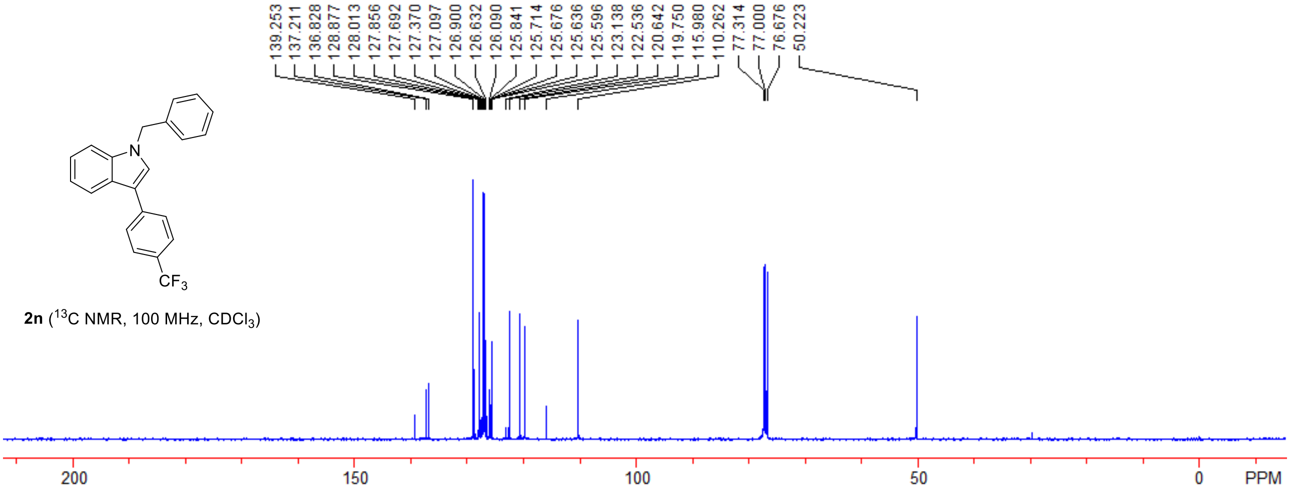

2n (^{13}C NMR, 100 MHz, $CDCl_3$)

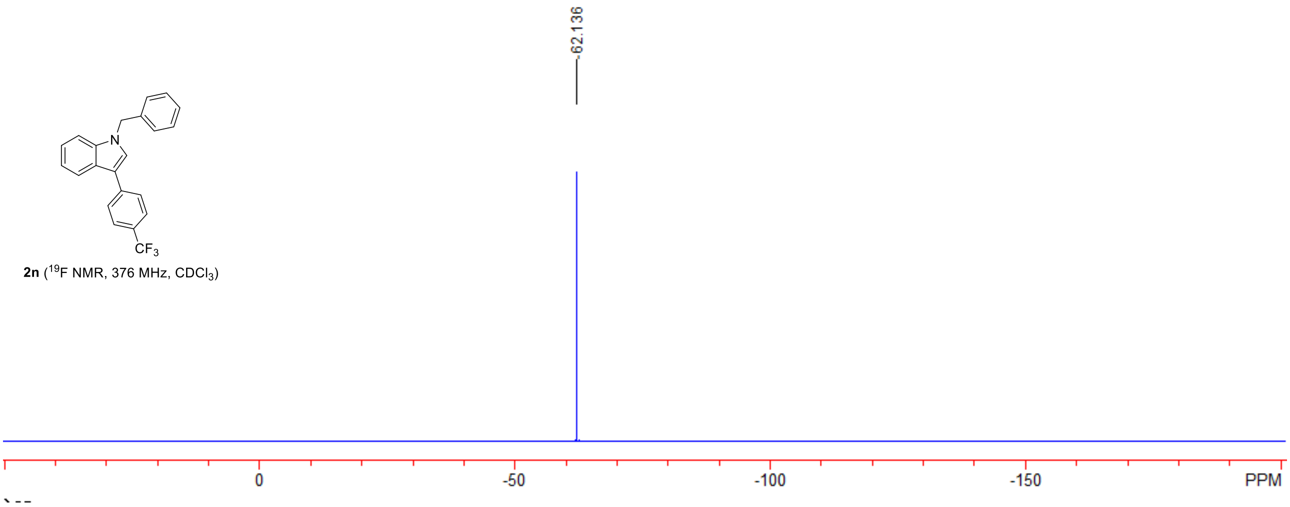

2n (^{19}F NMR, 376 MHz, $CDCl_3$)

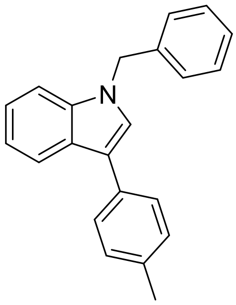

Compound 2o:[13] Yield: 23.8 mg, 80%; a light yellow solid; Mp: 75-77 ^{o}C; Eluent: PE/EA = 20/1. ^{1}H NMR (400 MHz, $CDCl_3$, TMS) δ 2.37 (s, 3H), 5.25 (s, 2H), 7.11 (d, *J* = 6.8 Hz, 2H), 7.15-7.30 (m, 9H), 7.55 (d, *J* = 7.6 Hz, 2H), 7.94 (d, *J* = 8.4 Hz, 1H); ^{13}C{^{1}H}-NMR (101 MHz, $CDCl_3$, TMS) δ_C 21.1, 50.0, 109.9, 117.2, 119.95, 120.03, 122.0, 125.6, 126.4, 126.8, 127.2, 127.6, 128.7, 129.4, 132.5, 135.4, 137.0, 137.2.

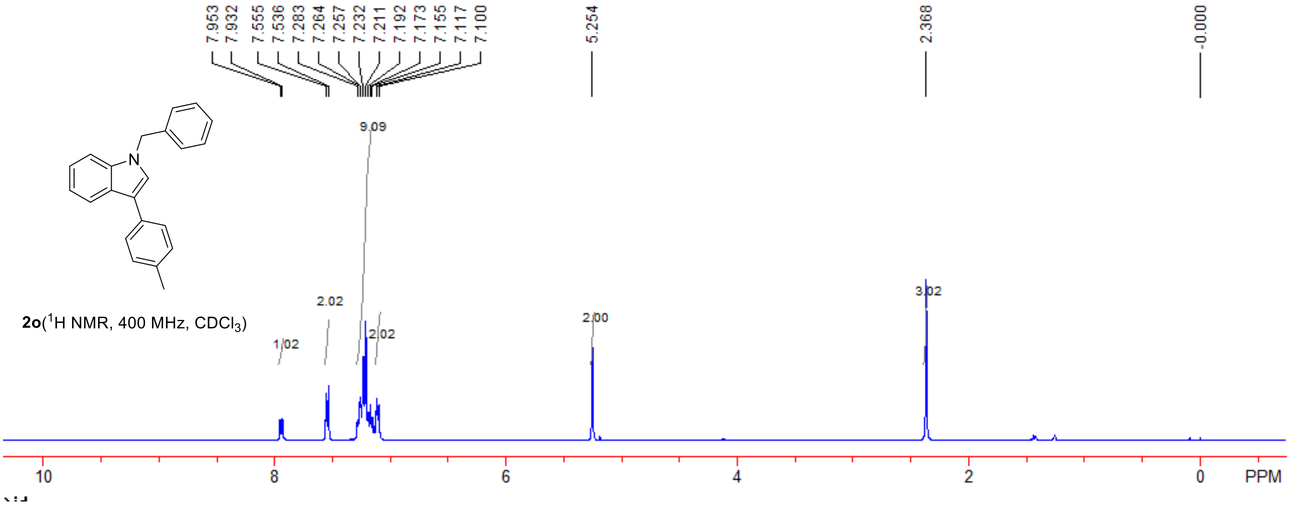

2o(^{1}H NMR, 400 MHz, $CDCl_3$)

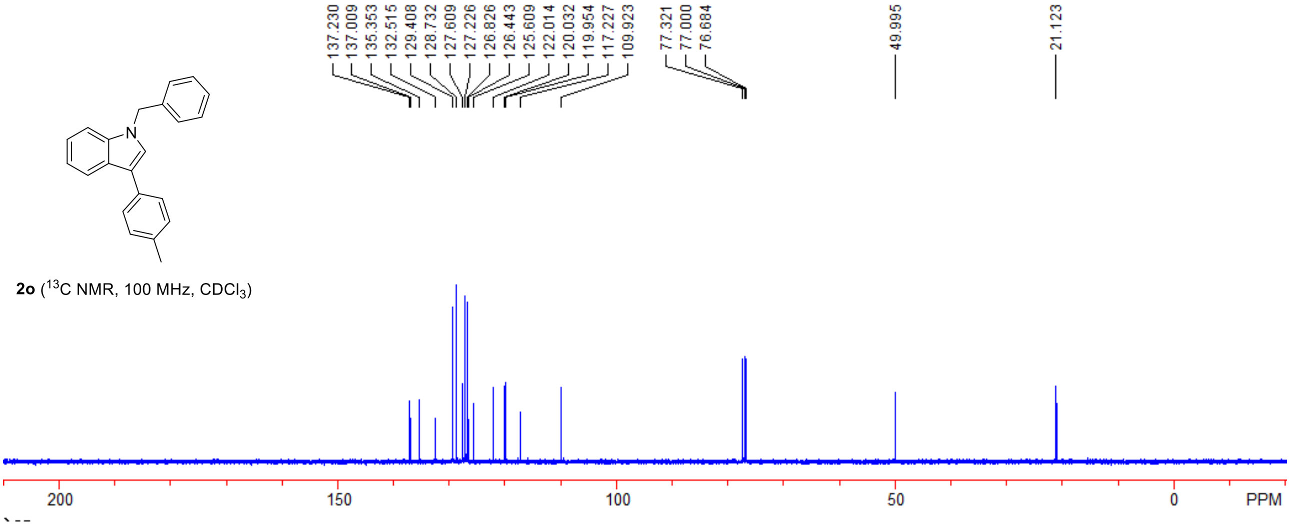

2o (^{13}C NMR, 100 MHz, $CDCl_3$)

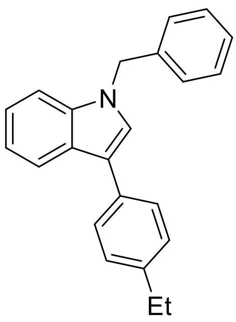

Compound 2p: Yield: 25.8 mg, 83%; a light yellow solid; Mp: 74-76 ºC; Eluent: PE/EA = 20/1. ^{1}H NMR (400 MHz, $CDCl_3$, TMS) δ 1.28 (t, *J* = 7.6 Hz, 3H), 2.69 (q, *J* = 7.6 Hz, 2H), 5.32 (s, 2H), 7.12-7.24 (m, 6H), 7.26-7.34 (m, 5H), 7.59 (d, *J* = 7.6 Hz, 2H), 7.96 (d, *J* = 6.8 Hz, 1H); ^{13}C{^{1}H}-NMR (101 MHz, $CDCl_3$, TMS) δ_C 15.7, 28.6, 50.0, 109.9, 117.3, 119.9, 120.1, 122.0, 125.7, 126.4, 126.8, 127.3, 127.6, 128.2, 128.8, 132.8, 137.0, 137.2, 141.8; IR (neat): ν 3026, 2959, 2923, 1612, 1543, 1467, 1436, 1384, 1332, 1299, 1181, 1070, 1070, 1018, 938, 831, 808, 726, 694 cm^{-1}; HRMS (EI-TOF) Calcd for $C_{23}H_{21}N$ $[M]^+$: 311.1669, found: 311.1676.

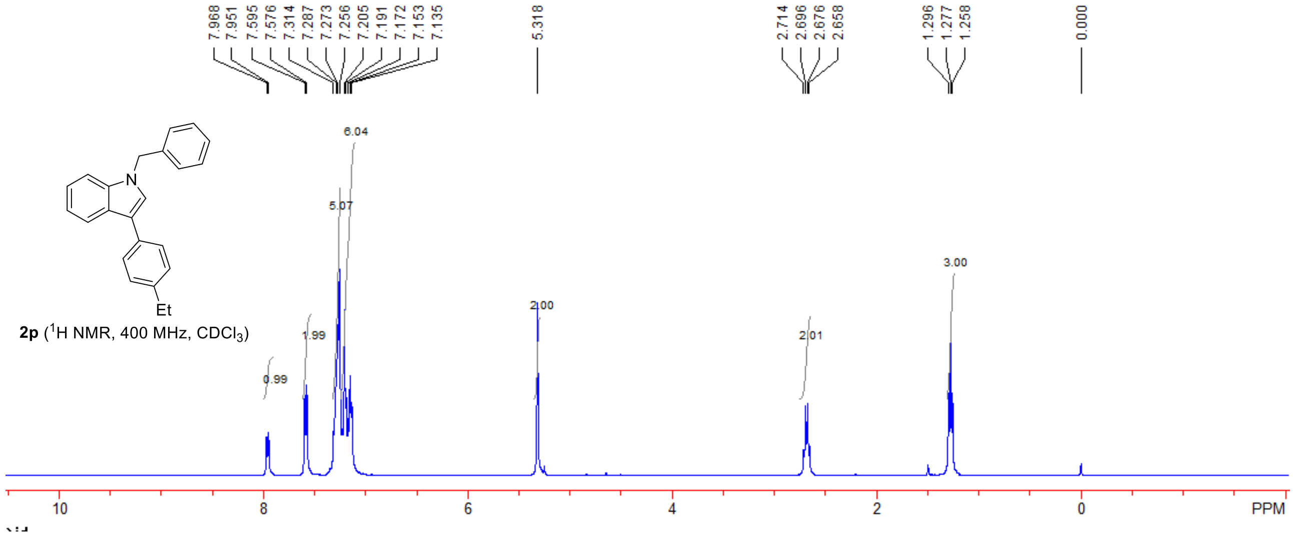

2p (^{1}H NMR, 400 MHz, $CDCl_3$)

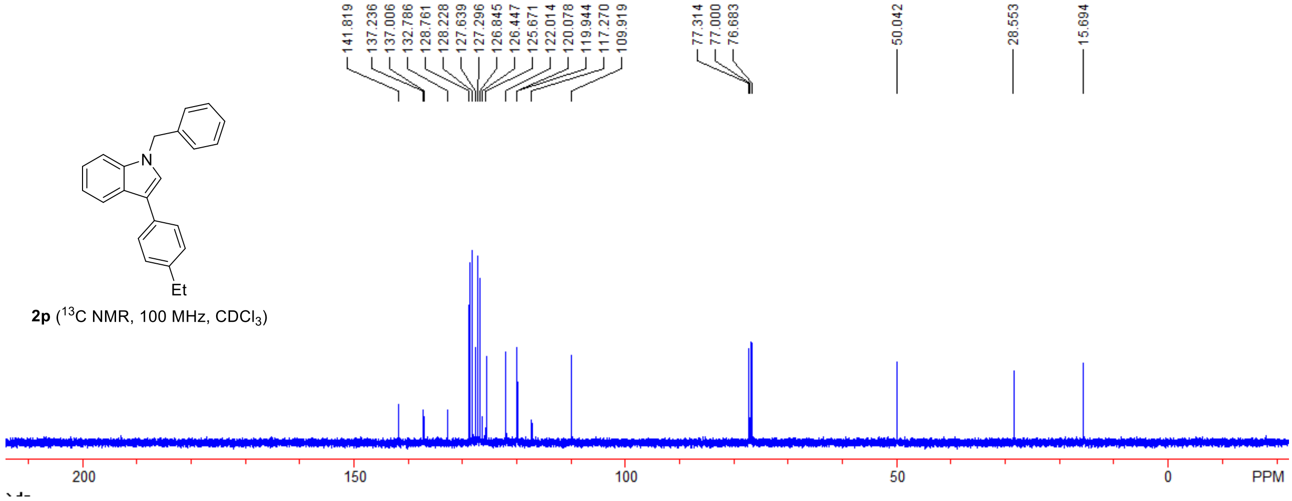

2p (^{13}C NMR, 100 MHz, $CDCl_3$)

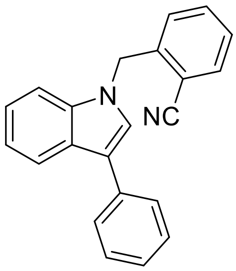

Compound 2s: Yield: 21.3 mg, 69%; a yellow oil; Eluent: PE/EA = 20/1. ^{1}H NMR (400 MHz, $CDCl_3$, TMS) δ 5.51 (s, 2H), 6.86 (d, *J* = 7.6 Hz, 1H), 7.15-7.39 (m, 7H), 7.42 (t, *J* = 7.2 Hz, 2H), 7.66 (d, *J* = 7.6 Hz, 3H), 7.96 (d, *J* = 6.8 Hz, 1H); $^{13}C\{^{1}H\}$-NMR (101 MHz, $CDCl_3$, TMS) δ_C 48.0, 109.7, 110.6, 117.1, 118.0, 120.2, 120.46, 120.53, 125.8, 126.0, 126.4, 127.3, 127.4, 128.1, 128.7, 132.9, 133.3, 135.0, 136.8, 140.9; IR (neat): ν 3026, 2924, 2222, 1600, 1546, 1479, 1450, 1376, 1331, 1235, 1179, 1072, 968, 910, 808, 736, 696 cm^{-1}; HRMS (ESI-TOF) Calcd for $C_{22}H_{17}N_2$ $[M+H]^+$: 309.1386, found: 309.1388.

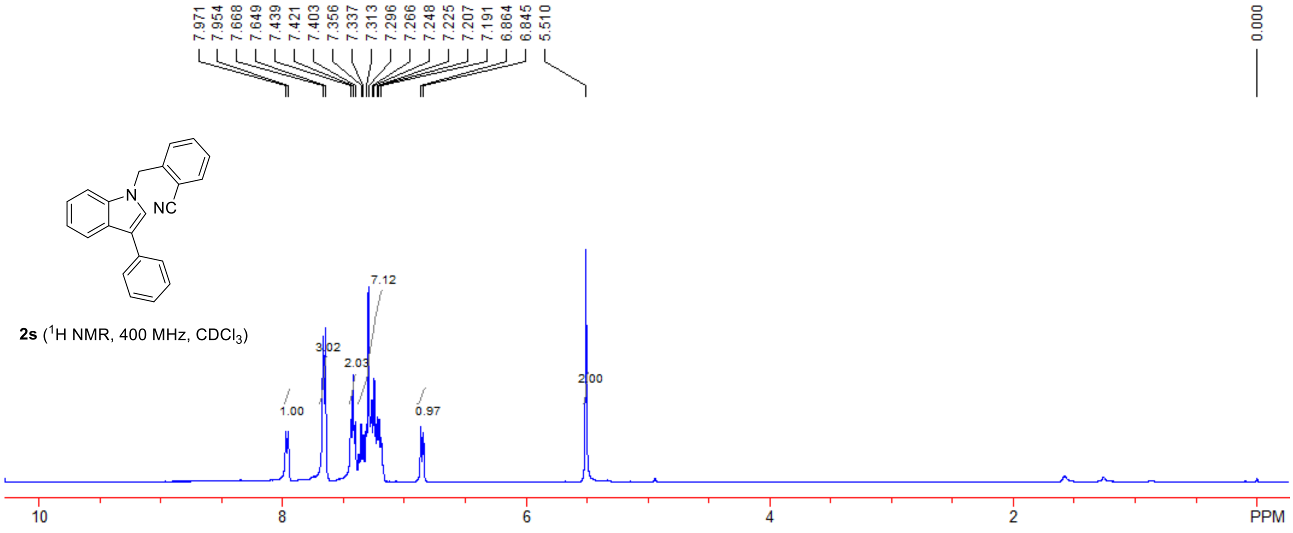

2s (^{1}H NMR, 400 MHz, $CDCl_3$)

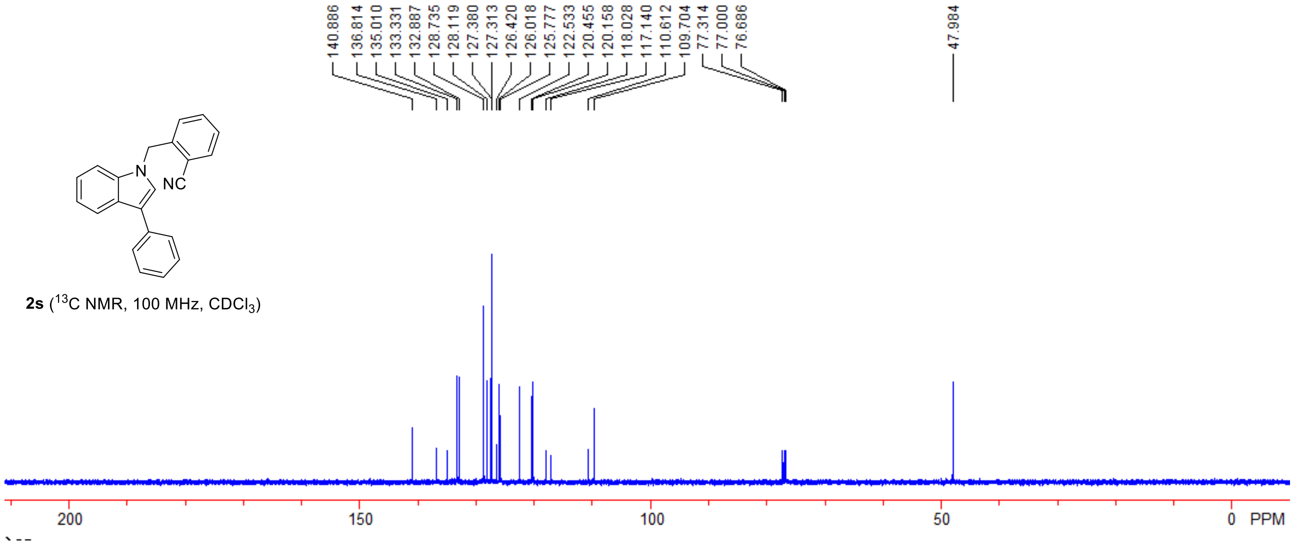

2s (^{13}C NMR, 100 MHz, $CDCl_3$)

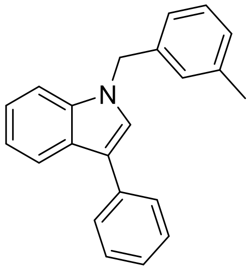

Compound 2t: Yield: 24.4 mg, 82%; a light yellow solid; Mp: 77-79 ºC; Eluent: PE/EA = 20/1. ^{1}H NMR (400 MHz, $CDCl_3$, TMS) δ 2.26 (s, 3H), 5.22 (s, 2H), 6.92 (d, *J* = 7.6 Hz, 1H), 6.97 (s, 1H), 7.04 (d, *J* = 7.6 Hz, 1H), 7.12-7.27 (m, 5H), 7.30 (d, *J* = 7.6 Hz, 1H), 7.40 (t, *J* = 7.6 Hz, 2H), 7.65 (d, *J* = 7.6 Hz, 2H), 7.96 (d, *J* = 7.6 Hz, 1H); ^{13}C{^{1}H}-NMR (101 MHz, $CDCl_3$, TMS) δ_C 21.4, 50.0, 110.0, 117.2, 120.0, 120.1, 122.1, 124.0, 125.7, 125.9, 126.4, 127.3, 127.6, 128.5, 128.66, 128.71, 135.5, 137.08, 137.11, 138.5; IR (neat): ν 3045, 2972, 2923, 1601, 1539, 1491, 1468, 1435, 1374, 1302, 1238, 1088, 1035, 968, 904, 790, 727, 693 cm^{-1}; HRMS (EI-TOF) Calcd for $C_{22}H_{19}N$ $[M]^+$: 297.1512, found: 297.1518.

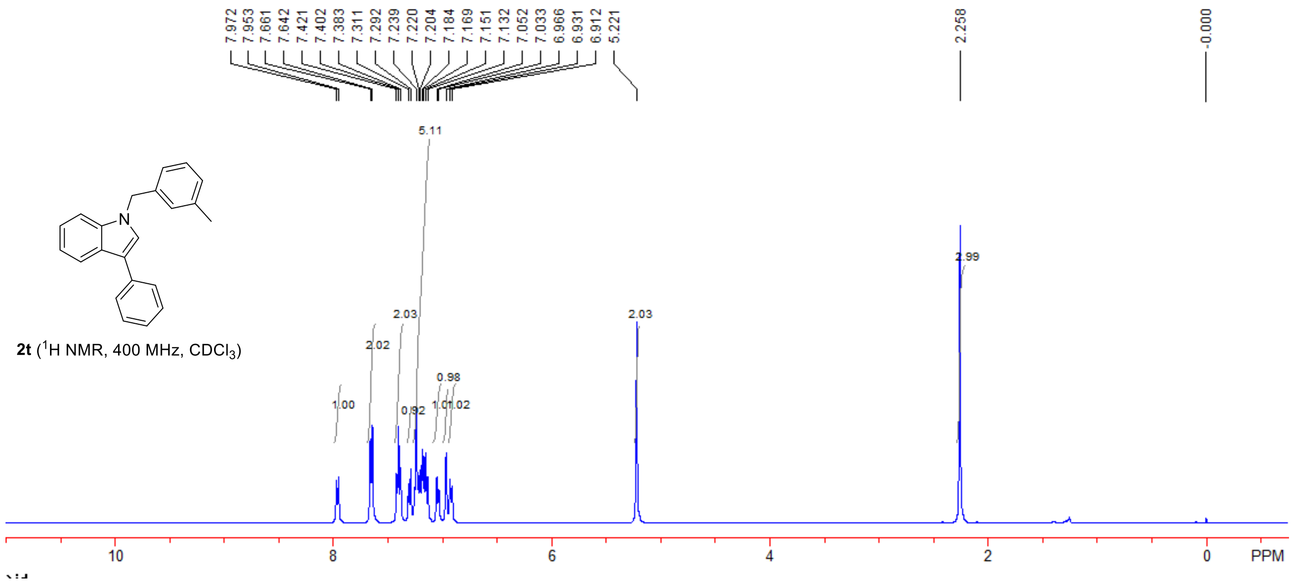

2t (^{1}H NMR, 400 MHz, $CDCl_3$)

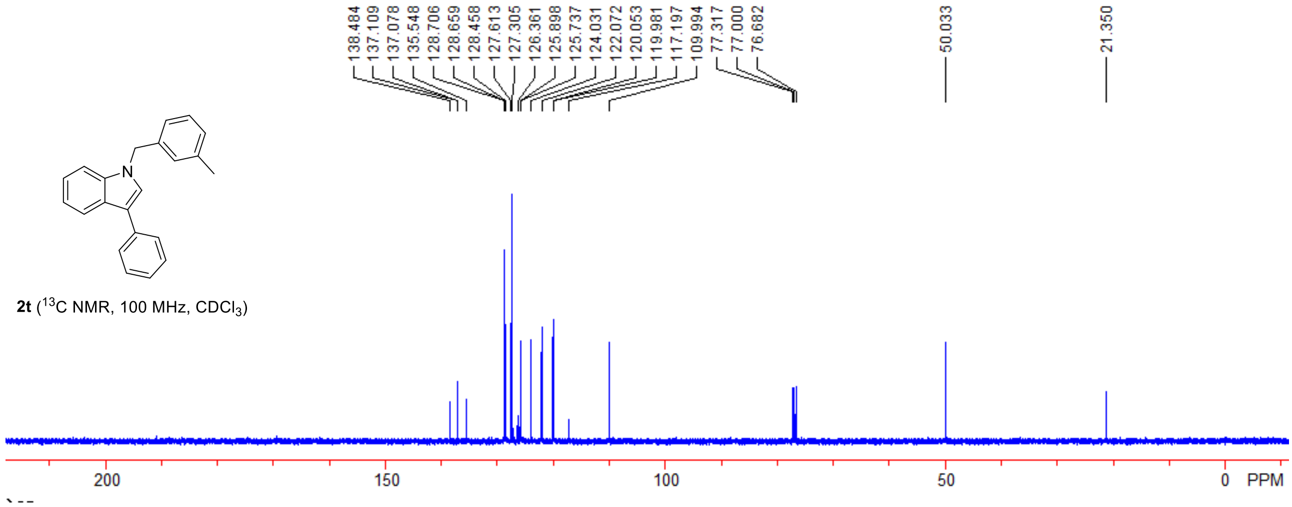

2t (^{13}C NMR, 100 MHz, $CDCl_3$)

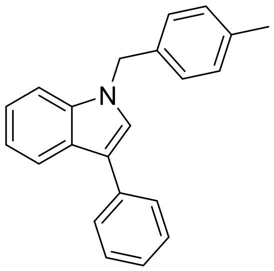

Compound 2u:[12] Yield: 26.4 mg, 89%; a yellow oil; Eluent: PE/EA = 20/1. ^{1}H NMR (400 MHz, $CDCl_3$, TMS) δ 2.25 (s, 3H), 5.15 (s, 2H), 6.98 (d, *J* = 7.6 Hz, 2H), 7.04 (d, *J* = 8.0 Hz, 2H), 7.12-7.29 (m, 5H), 7.38 (t, *J* = 7.6 Hz, 2H), 7.63 (d, *J* = 8.0 Hz, 2H), 7.95 (d, *J* = 6.8 Hz, 1H); ^{13}C{^{1}H}-NMR (101 MHz, $CDCl_3$, TMS) δ_C 21.0, 49.8, 110.0, 117.1, 119.96, 120.03, 122.0, 125.7, 125.8, 126.3, 126.9, 127.3, 128.7, 129.4, 134.1, 135.5, 137.0, 137.3.

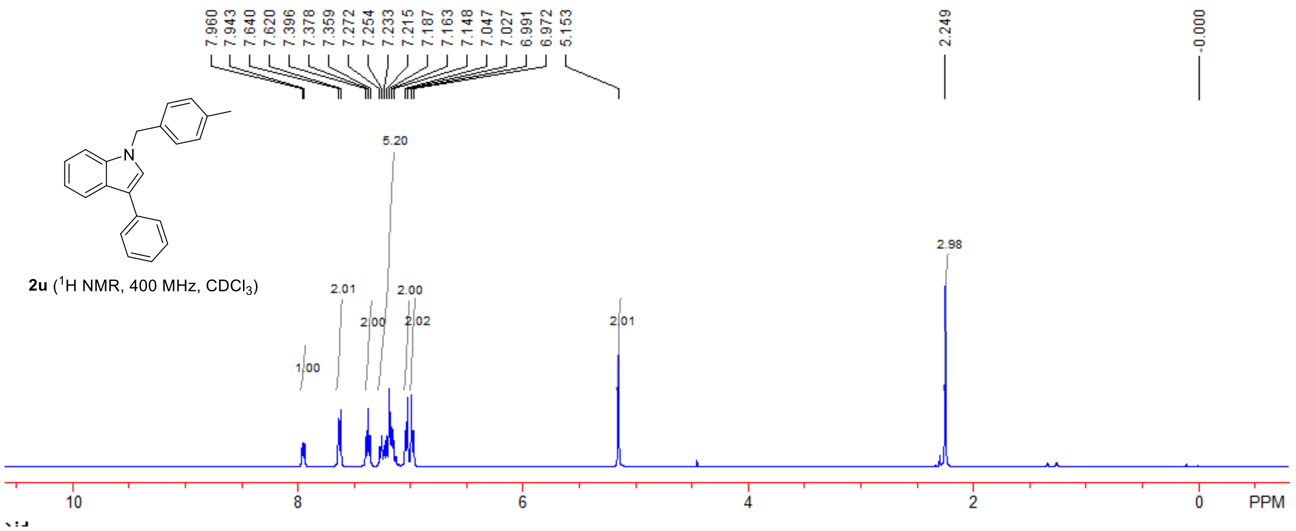

2u (^{1}H NMR, 400 MHz, $CDCl_3$)

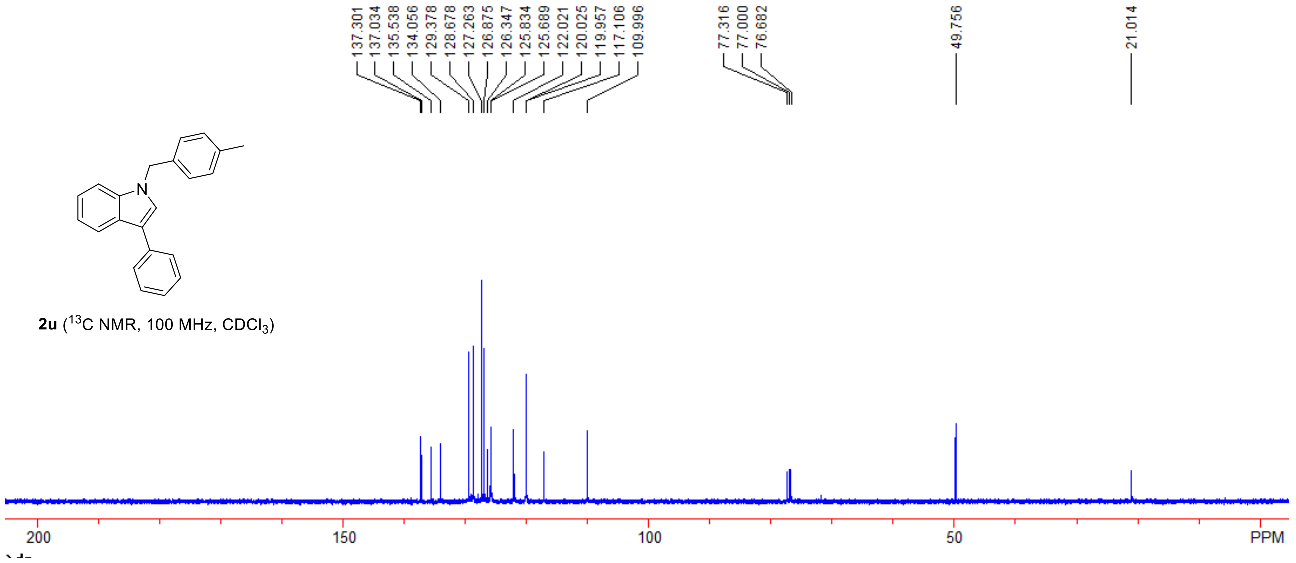

2u (^{13}C NMR, 100 MHz, $CDCl_3$)

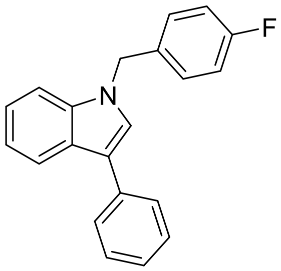

Compound 2v: Yield: 24.4 mg, 81%; a yellow oil; Eluent: PE/EA = 20/1. ^{1}H NMR (400 MHz, $CDCl_3$, TMS) δ 5.28 (s, 2H), 6.97 (t, *J* = 8.8 Hz, 2H), 7.08-7.14 (m, 2H), 7.15-7.30 (m, 5H), 7.43 (t, *J* = 7.2 Hz, 2H), 7.66 (d, *J* = 7.6 Hz, 2H), 7.97 (d, *J* = 8.0 Hz, 1H); $^{13}C\{^{1}H\}$-NMR (101 MHz, $CDCl_3$, TMS) δ_C 49.4, 109.9, 115.7 (d, *J* = 21.5 Hz), 117.5, 120.1, 120.2, 122.2, 125.7, 125.9, 126.5, 127.3, 128.6 (d, *J* = 7.4 Hz), 128.7, 132.9 (d, *J* = 3.2 Hz), 135.4, 136.9, 162.2 (d, *J* = 244.0 Hz); ^{19}F NMR (376 MHz, $CDCl_3$) δ -114.6; IR (neat): ν 3047, 2924, 1601, 1546, 1508, 1464, 1350, 1220, 1177, 1155, 1073, 972, 819, 737, 696 cm^{-1}; HRMS (EI-TOF) Calcd for $C_{21}H_{16}FN$ $[M]^+$: 301.1261, found: 301.1271.

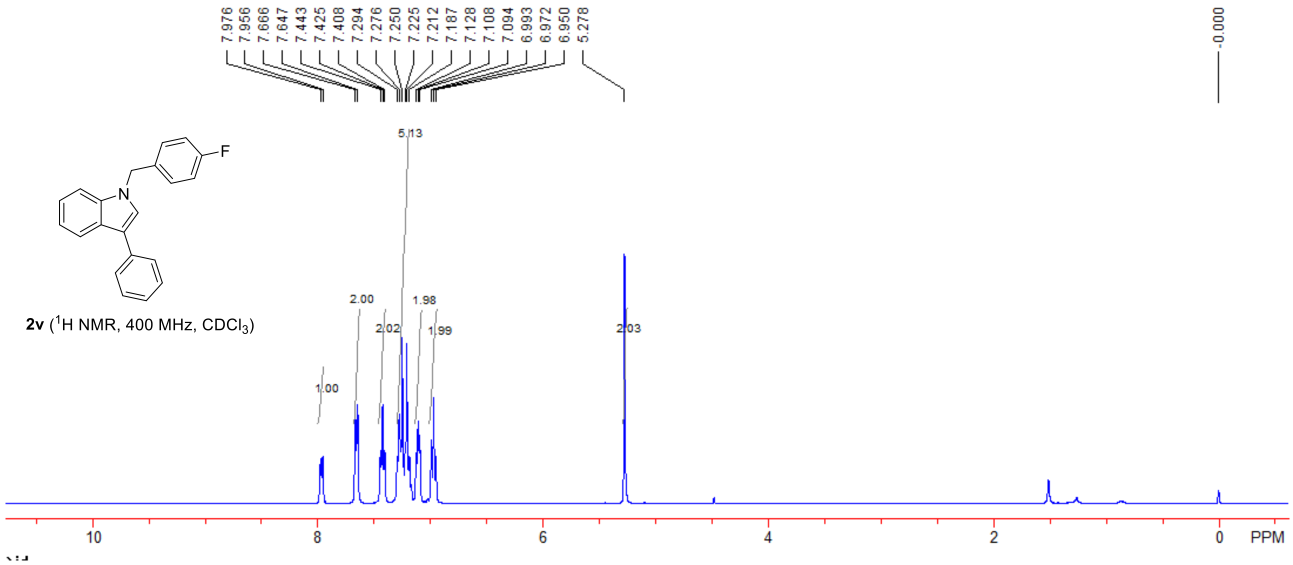

2v (^{1}H NMR, 400 MHz, $CDCl_3$)

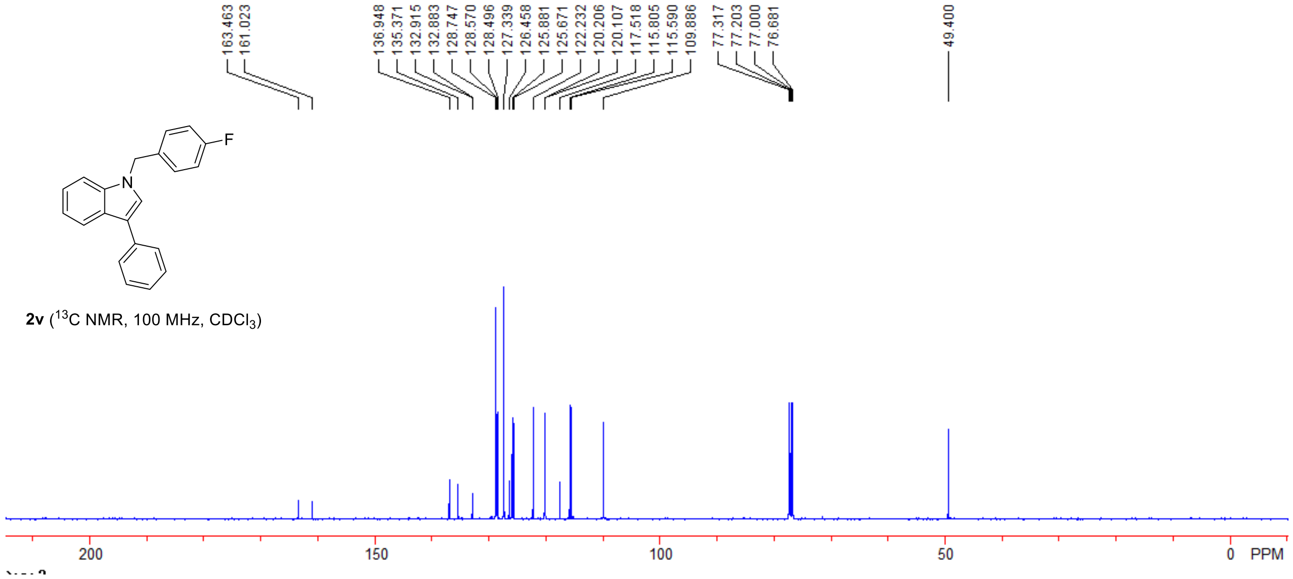

2v (^{13}C NMR, 100 MHz, $CDCl_3$)

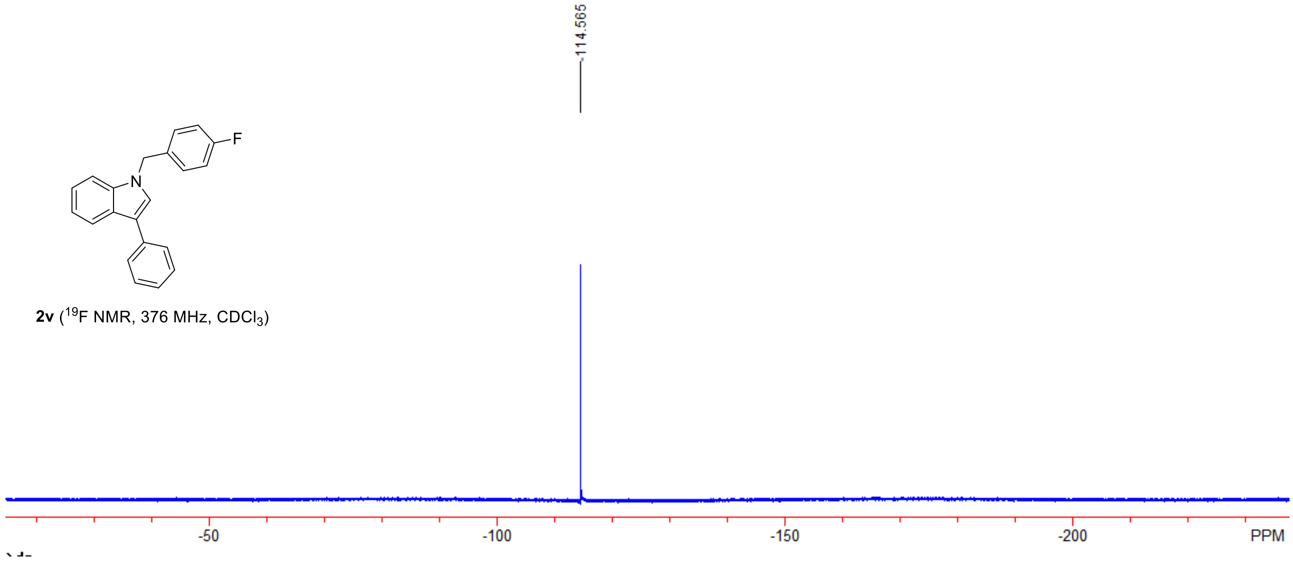

2v (^{19}F NMR, 376 MHz, $CDCl_3$)

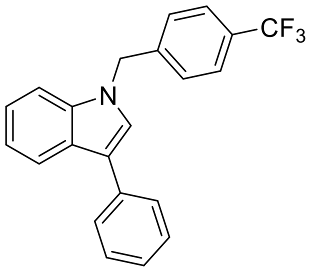

Compound 2w: Yield: 30.5 mg, 87%; a yellow oil; Eluent: PE/EA = 20/1. ^{1}H NMR (400 MHz, $CDCl_3$, TMS) δ 5.36 (s, 2H), 7.16-7.31 (m, 7H), 7.43 (t, *J* = 8.0 Hz, 2H), 7.53 (d, *J* = 8.0 Hz, 2H), 7.66 (d, *J* = 7.2 Hz, 2H), 7.95-8.00 (m, 1H); ^{13}C{^{1}H}-NMR (101 MHz, $CDCl_3$, TMS) δ_C 49.6, 109.8, 117.8, 120.2, 120.4, 122.4, 124.0 (q, *J* = 270.7 Hz), 125.7, 125.8 (q, *J* = 3.8 Hz), 126.0, 126.5, 126.9, 127.4, 128.8, 129.9 (q, *J* = 32.0 Hz), 135.2, 136.9, 141.3; ^{19}F NMR (376 MHz, $CDCl_3$) δ -62.5; IR (neat): ν 3049, 1602, 1546, 1464, 1418, 1320, 1161, 1064, 1016, 936, 819, 738, 697 cm^{-1}; HRMS (EI-TOF) Calcd for $C_{22}H_{16}F_3N$ $[M]^+$: 351.1229, found: 351.1240.

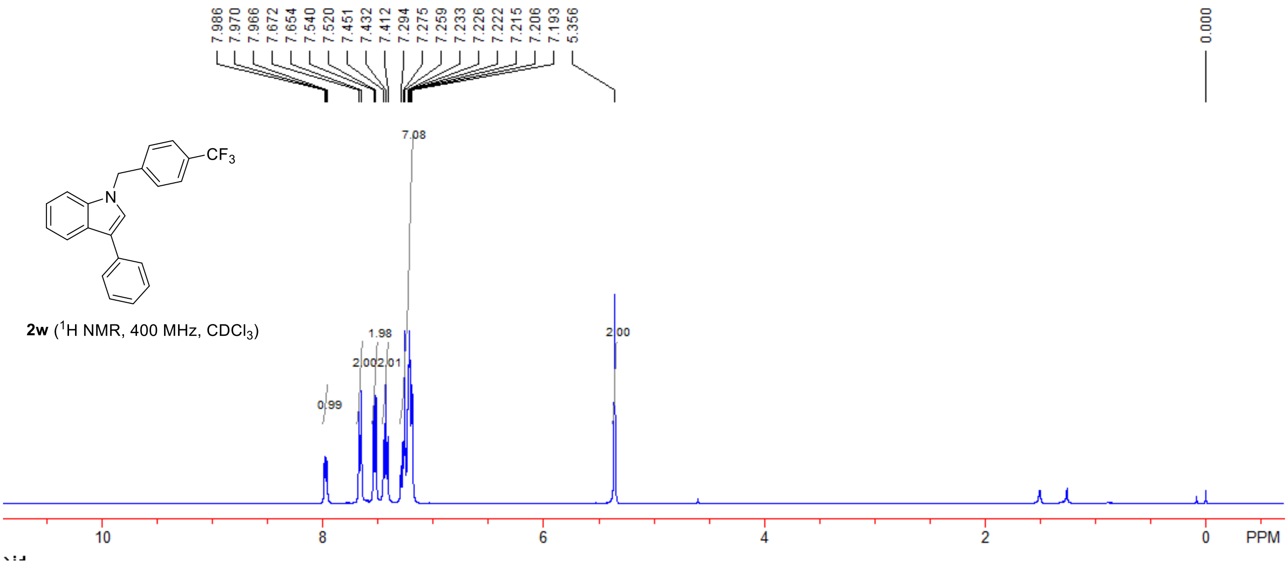

7.986
7.970
7.966
7.672
7.654
7.540
7.520
7.451
7.432
7.412
7.294
7.275
7.259
7.233
7.226
7.222
7.215
7.206
7.193
5.356
0.000
CF3
N
2w (1H NMR, 400 MHz, CDCl3)
7.08
1.98
2.00
2.01
0.99
2.00
10
8
6
4
2
0
PPM

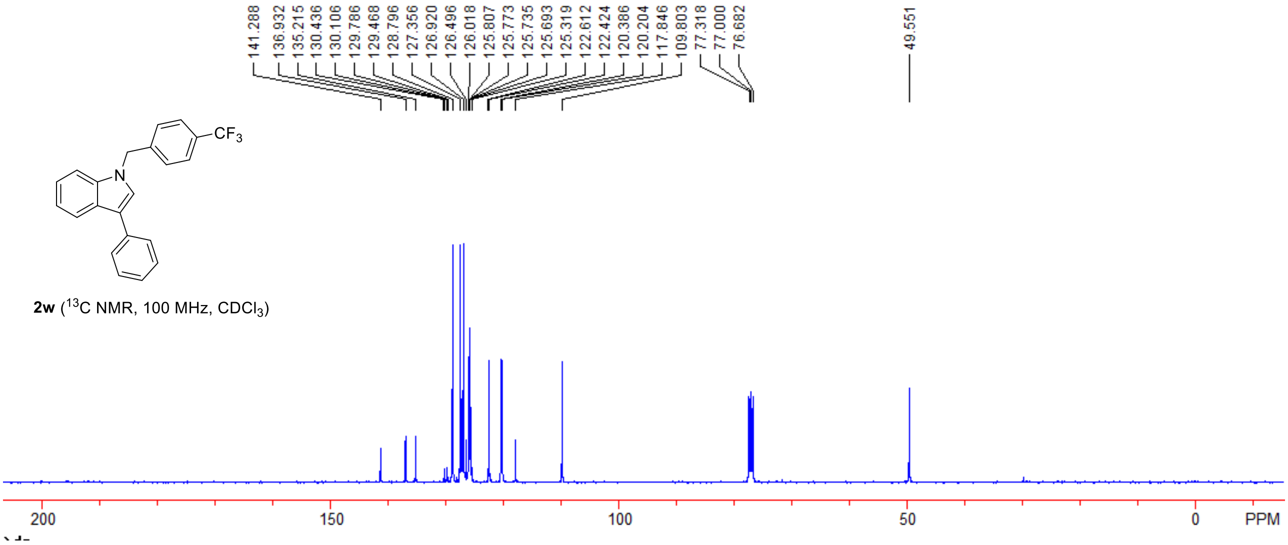

141.288
136.932
135.215
130.436
130.106
129.786
129.468
128.796
127.356
126.920
126.496
126.018
125.807
125.773
125.735
125.693
125.319
122.612
122.424
120.386
120.204
117.846
109.803
77.318
77.000
76.682
49.551
CF3
N
2w (13C NMR, 100 MHz, CDCl3)
200
150
100
50
0
PPM

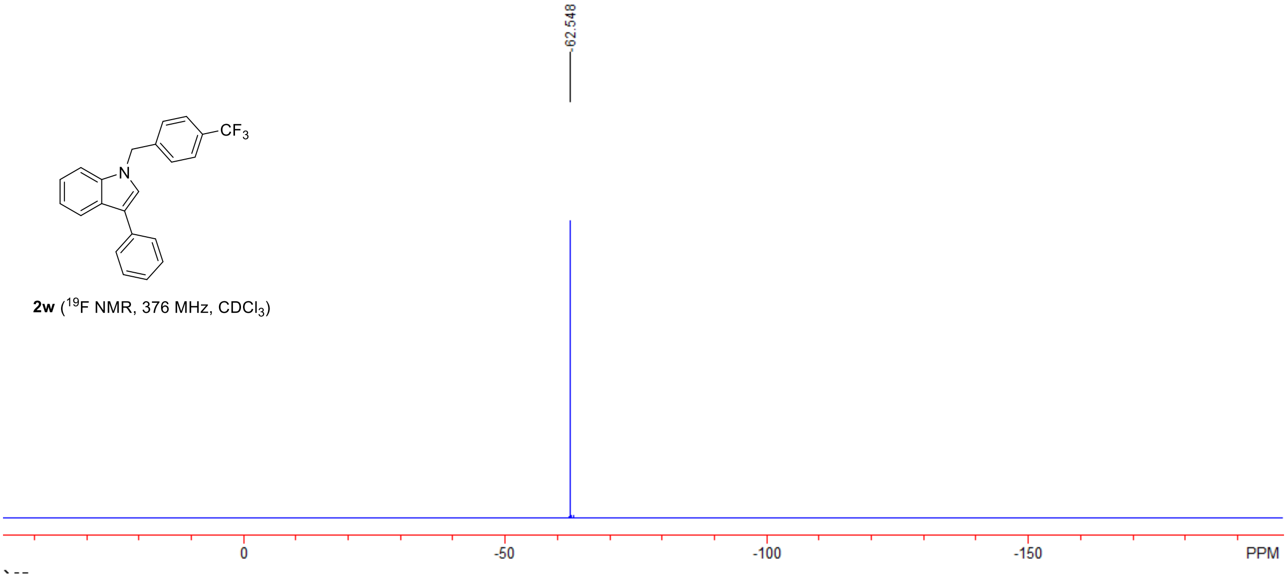

-62.548
CF3
N
2w (19F NMR, 376 MHz, CDCl3)
0
-50
-100
-150
PPM

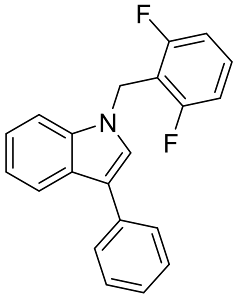

Compound 2x: Yield: 27.1 mg, 85%; a light yellow solid; Mp: 89-91 ºC; Eluent: PE/EA = 20/1. ^{1}H NMR (400 MHz, $CDCl_3$, TMS) δ 5.33 (s, 2H), 6.85 (t, *J* = 8.4 Hz, 2H), 7.13-7.31 (m, 4H), 7.36-7.44 (m, 3H), 7.58 (d, *J* = 8.8 Hz, 1H), 7.63 (d, *J* = 7.6 Hz, 2H), 7.90 (d, *J* = 7.6 Hz, 1H); ^{13}C{^{1}H}-NMR (101 MHz, $CDCl_3$, TMS) δ_C 63.5 (t, *J* = 3.5 Hz), 109.7 (d, *J* = 2.0 Hz), 111.6 (dd, J_1 = 19.4 Hz, J_2 = 6.1 Hz), 113.0 (t, *J* = 19.7 Hz), 117.5, 119.8, 120.1, 122.2, 125.8, 126.2, 127.4, 128.7, 130.2 (t, *J* = 10.4 Hz), 135.4, 136.7, 161.4 (dd, J_1 = 248.7 Hz, J_2 = 7.8 Hz); ^{19}F NMR (376 MHz, $CDCl_3$) δ -113.8; IR (neat): ν 3053, 2920, 2852, 1623, 1598, 1545, 1466, 1390, 1352, 1304, 1231, 1145, 1017, 967, 902, 816, 790, 728, 698 cm^{-1}; HRMS (EI-TOF) Calcd for $C_{21}H_{15}F_2N$ $[M]^+$: 319.1167, found: 319.1174.

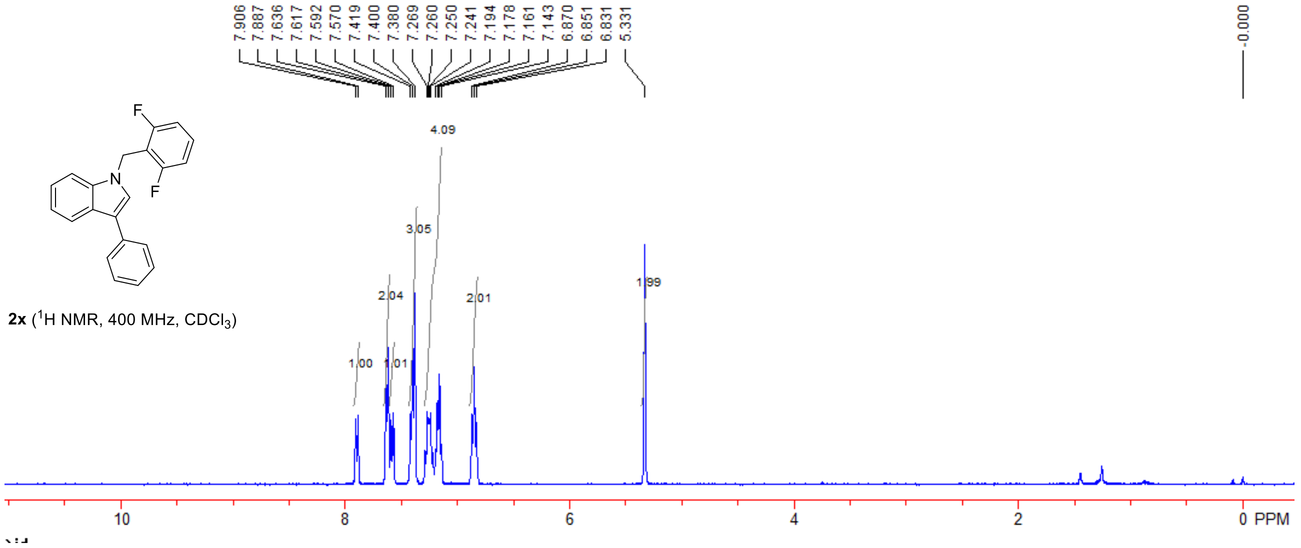

2x (^{1}H NMR, 400 MHz, $CDCl_3$)

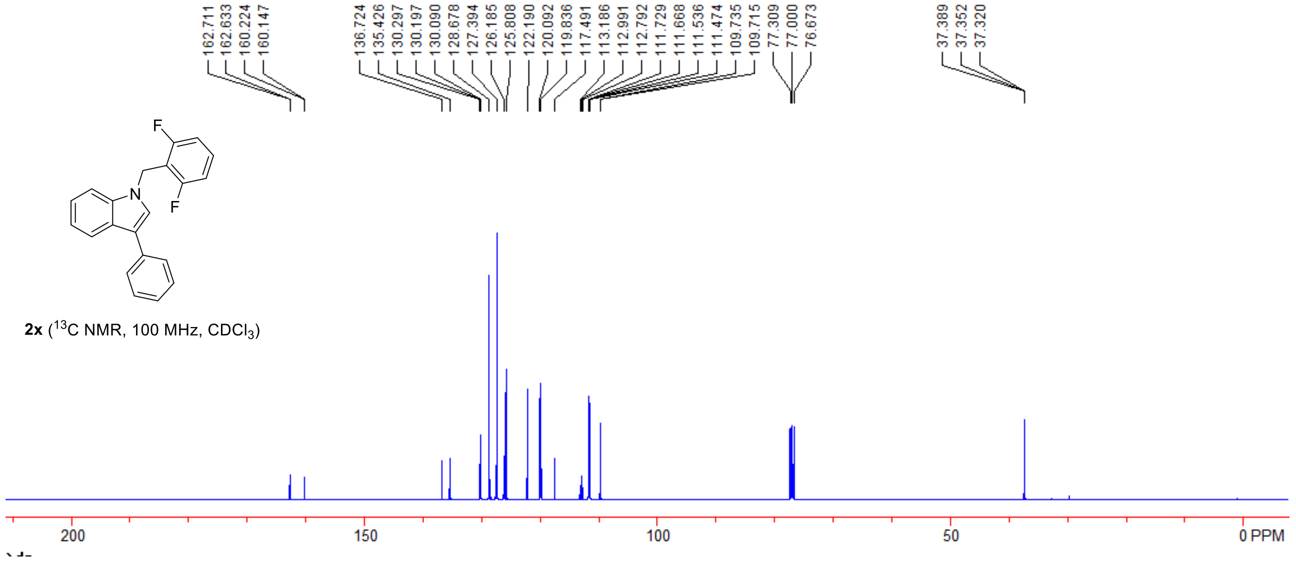

2x (^{13}C NMR, 100 MHz, $CDCl_3$)

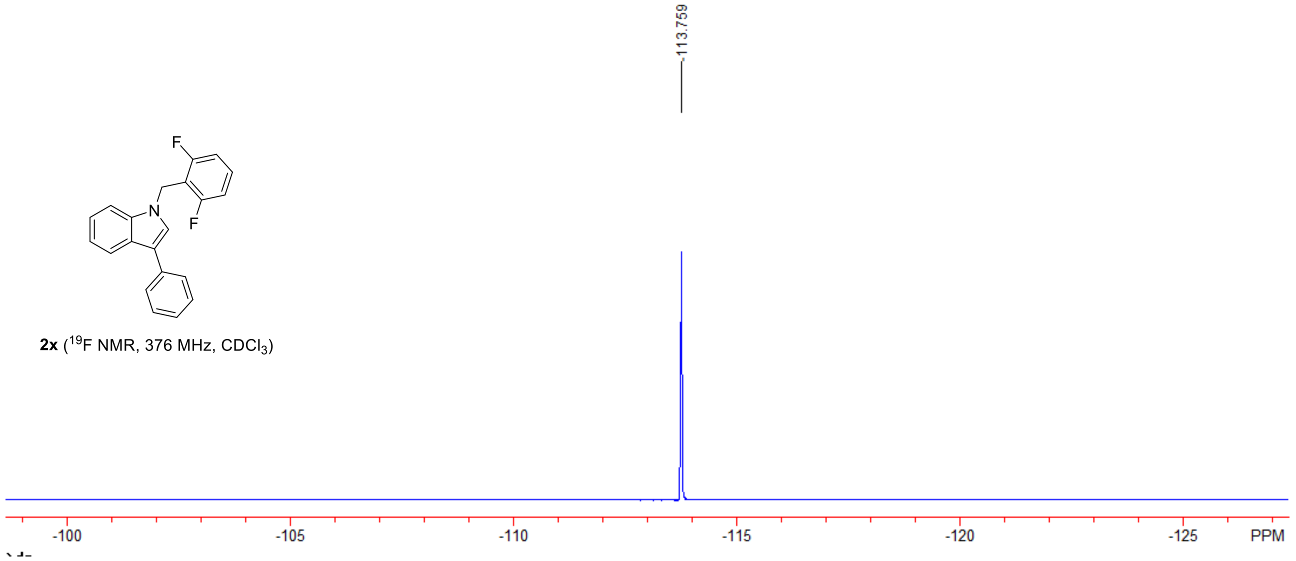

2x (^{19}F NMR, 376 MHz, $CDCl_3$)

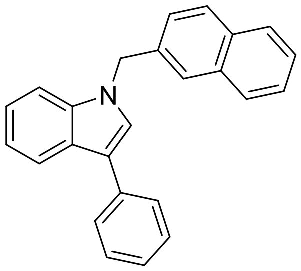

Compound 2y: Yield: 25.3 mg, 76%; a light yellow solid; Mp: 135-137 ^{o}C; Eluent: PE/EA = 20/1. ^{1}H NMR (400 MHz, $CDCl_3$, TMS) δ 5.49 (s, 2H), 7.16-7.23 (m, 2H), 7.28 (d, *J* = 7.6 Hz, 2H), 7.33-7.48 (m, 6H), 7.62 (s, 1H), 7.68 (d, *J* = 7.6 Hz, 2H), 7.72-7.82 (m, 3H), 7.99 (d, *J* = 6.8 Hz, 1H); ^{13}C{^{1}H}-NMR (101 MHz, $CDCl_3$, TMS) δ_C 50.4, 110.0, 117.4, 120.07, 120.15, 120.19, 124.9, 125.7, 125.8, 125.9, 126.1, 126.4, 126.5, 127.4, 127.7, 127.8, 128.7, 132.9, 133.3, 134.6, 135.5, 137.2; IR (neat): ν 3054, 3023, 2922, 2852, 1666, 1599, 1544, 1508, 1466, 1433, 1386, 1334, 1261, 1158, 1014, 972, 910, 815, 769, 698 cm^{-1}; HRMS (FI-TOF) Calcd for $C_{25}H_{19}N$ $[M]^+$: 333.1512, found: 333.1516.

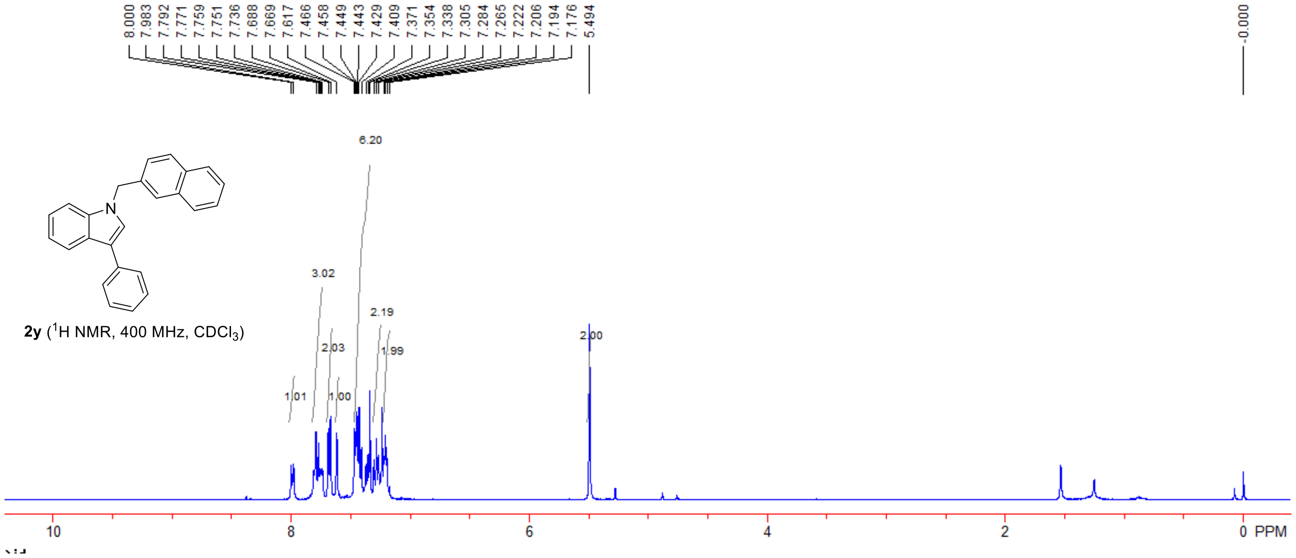

2y (^{1}H NMR, 400 MHz, $CDCl_3$)

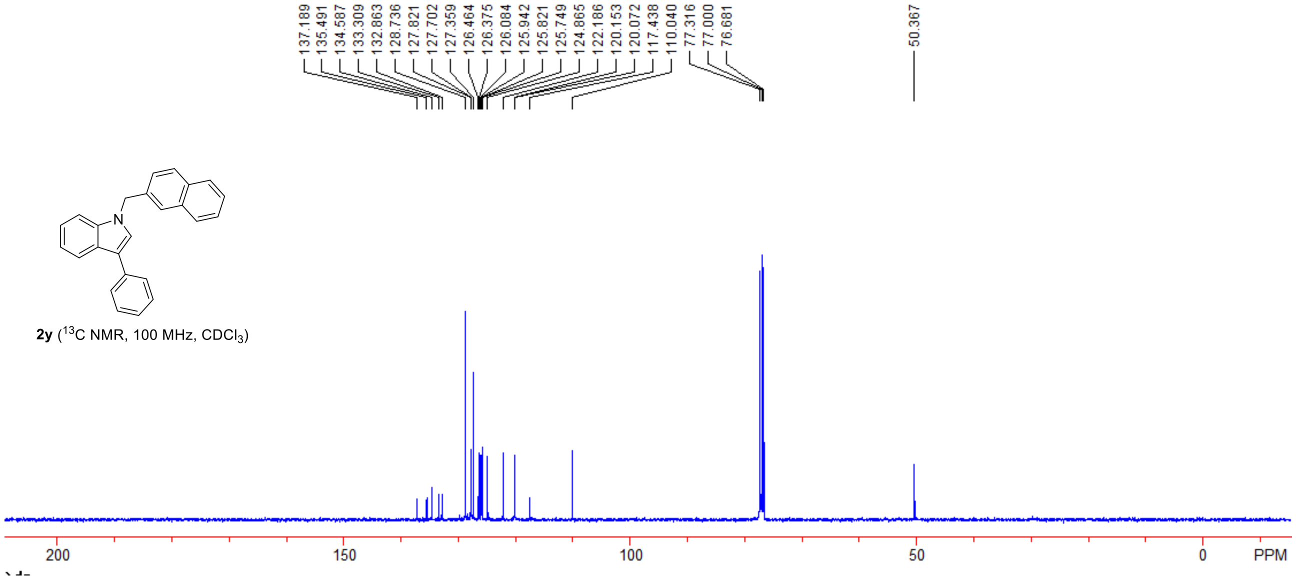

2y (^{13}C NMR, 100 MHz, $CDCl_3$)

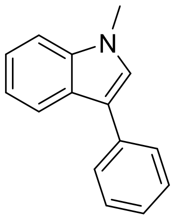

Compound 2z:[14] Yield: 18.4 mg, 89%; a yellow oil; Eluent: PE/EA = 20/1. ^{1}H NMR (400 MHz, $CDCl_3$, TMS) δ 3.70 (s, 3H), 7.12-7.19 (m, 2H), 7.21-7.27 (m, 2H), 7.30 (d, J = 8.4 Hz, 1H), 7.40 (t, J = 8.0 Hz, 2H), 7.63 (d, J = 7.6 Hz, 2H), 7.94 (d, J = 8.0 Hz, 1H); ^{13}C{^{1}H}-NMR (101 MHz, $CDCl_3$, TMS) δ_C 32.7, 109.5, 116.5, 119.8, 121.9, 125.6, 126.1, 126.5, 127.2, 128.7, 135.6, 137.4.

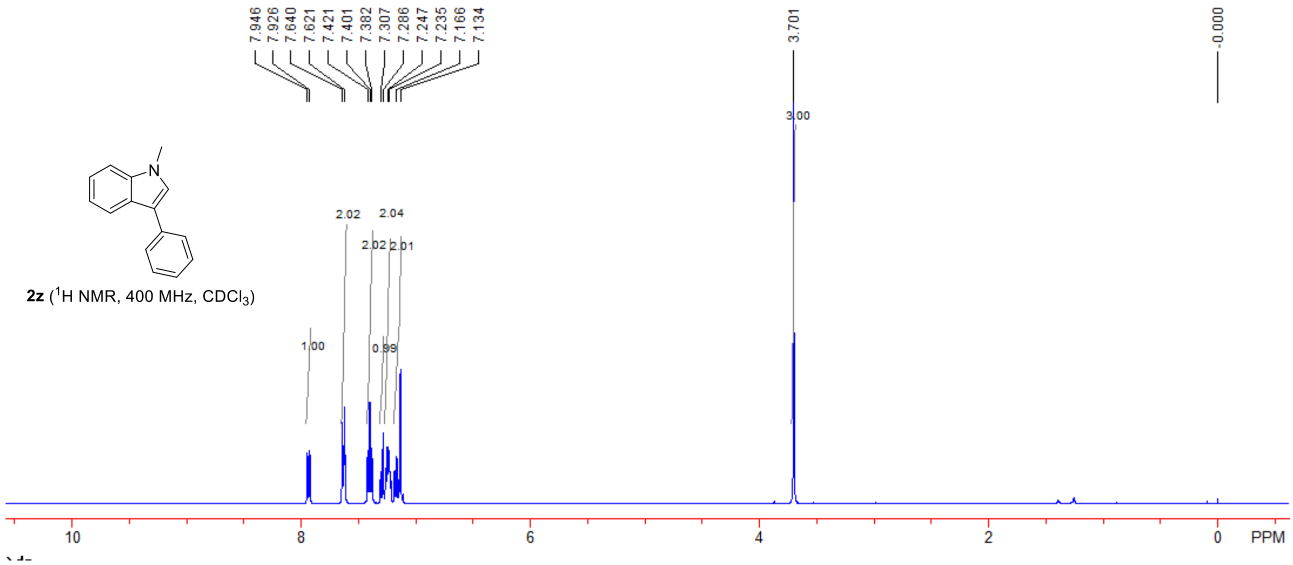

2z (^{1}H NMR, 400 MHz, $CDCl_3$)

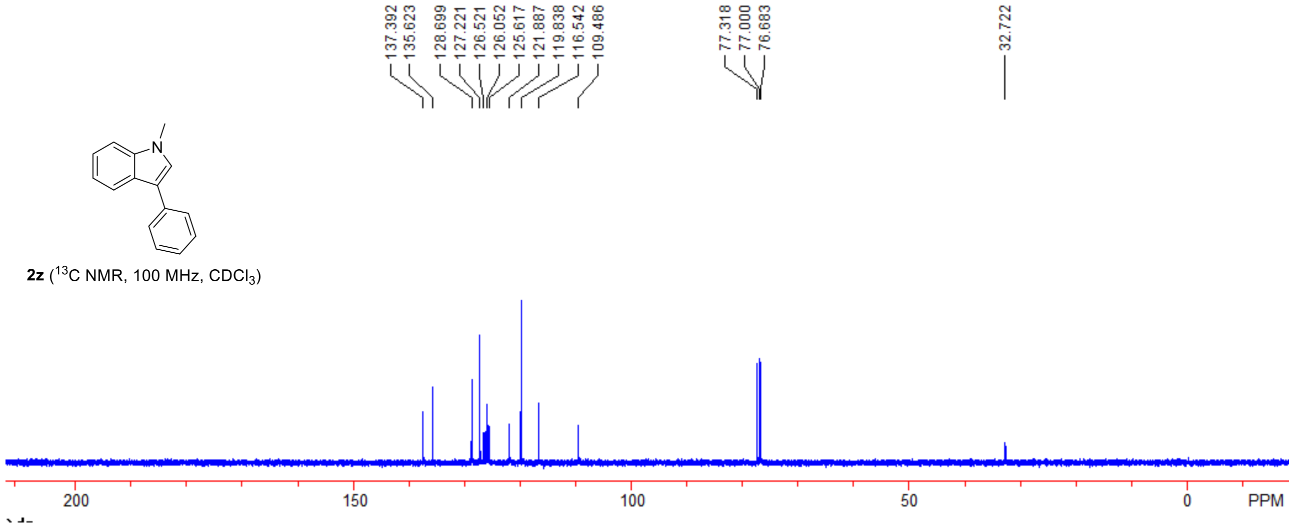

2z (^{13}C NMR, 100 MHz, $CDCl_3$)

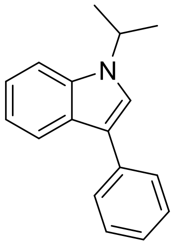

Compound 2aa:[15] Yield: 18.3 mg, 78%; a yellow oil; Eluent: PE/EA = 20/1. ^{1}H NMR (400 MHz, $CDCl_3$, TMS) δ 1.51 (d, *J* = 6.4 Hz, 6H), 4.60-4.70 (m, 1H), 7.16 (t, *J* = 7.2 Hz, 1H), 7.24 (t, *J* = 7.6 Hz, 2H), 7.34-7.45 (m, 4H), 7.66 (d, *J* = 7.2 Hz, 2H), 7.95 (d, *J* = 8.0 Hz, 1H); ^{13}C{^{1}H}-NMR (101 MHz, $CDCl_3$, TMS) δ_C 22.7, 47.0, 109.7, 116.8, 119.8, 119.9, 121.4, 121.6, 125.6, 126.2, 127.3, 128.7, 135.8, 136.3.

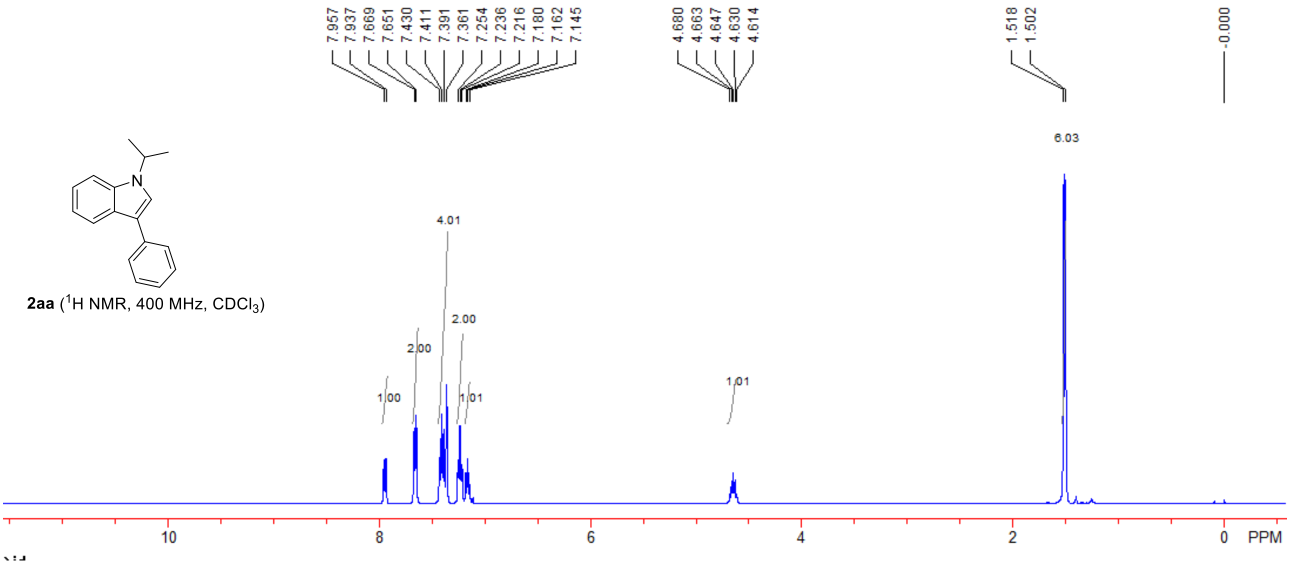

2aa (^{1}H NMR, 400 MHz, $CDCl_3$)

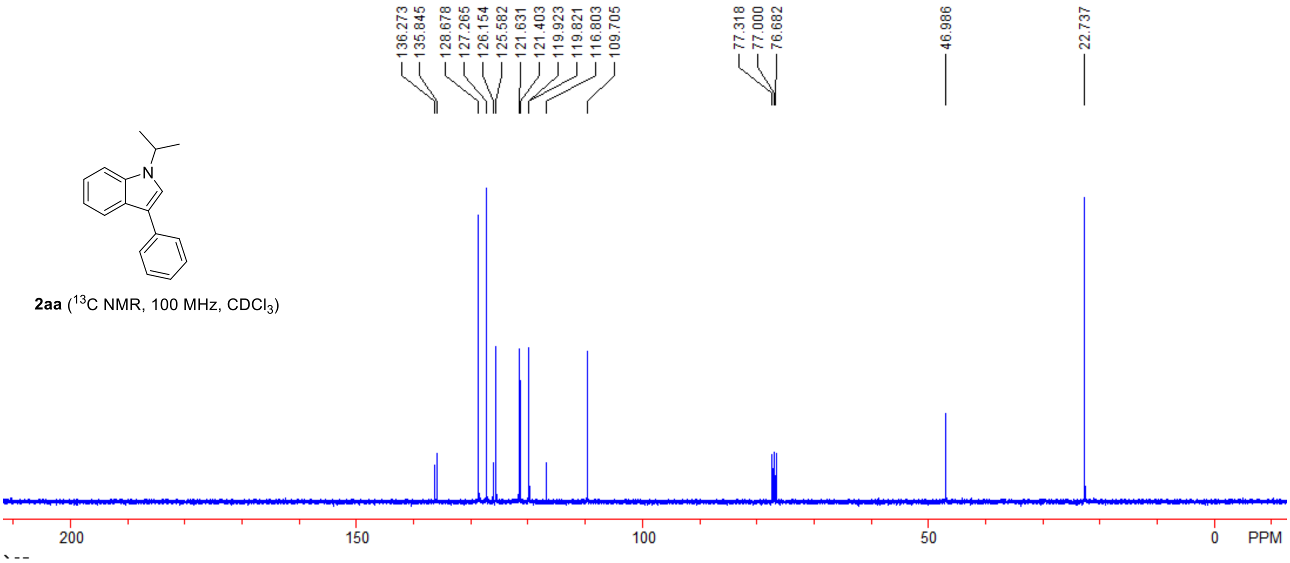

2aa (^{13}C NMR, 100 MHz, $CDCl_3$)

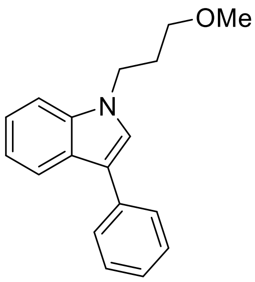

Compound 2ab: Yield: 21.5 mg, 81%; a yellow oil; Eluent: PE/EA = 20/1. ^{1}H NMR (400 MHz, $CDCl_3$, TMS) δ 2.00-2.10 (m, 2H), 3.27 (t, *J* = 5.6 Hz, 2H), 3.30 (s, 3H), 4.23 (t, *J* = 6.8 Hz, 2H), 7.16 (t, *J* = 7.6 Hz, 1H), 7.21-7.28 (m, 3H), 7.35-7.45 (m, 3H), 7.65 (d, *J* = 7.6 Hz, 2H), 7.94 (d, *J* = 7.6 Hz, 1H); ^{13}C{^{1}H}-NMR (101 MHz, $CDCl_3$, TMS) δ_C 30.2, 42.8, 58.6, 68.9, 109.7, 116.7, 119.8, 119.9, 121.8, 125.6, 125.7, 126.2, 127.2, 128.7, 135.7, 136.8; IR (neat): ν 2922, 2873, 1601, 1545, 1478, 1372, 1226, 1113, 1016, 940, 908, 766, 737, 696 cm^{-1}; HRMS (EI-TOF) Calcd for $C_{18}H_{19}NO$ $[M]^+$: 265.1461, found: 265.1465.

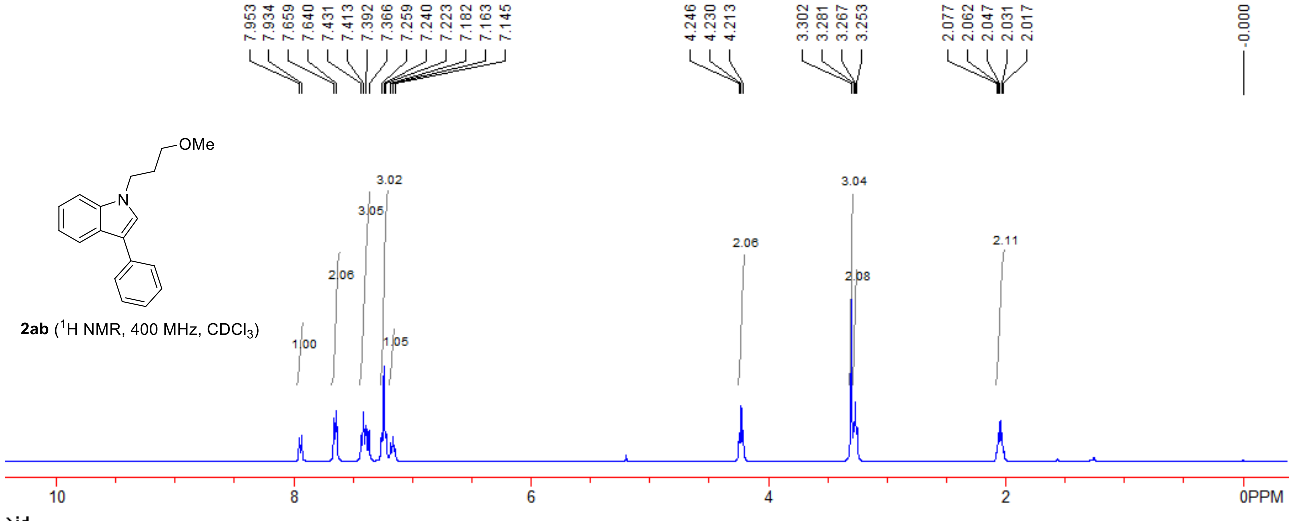

2ab (^{1}H NMR, 400 MHz, $CDCl_3$)

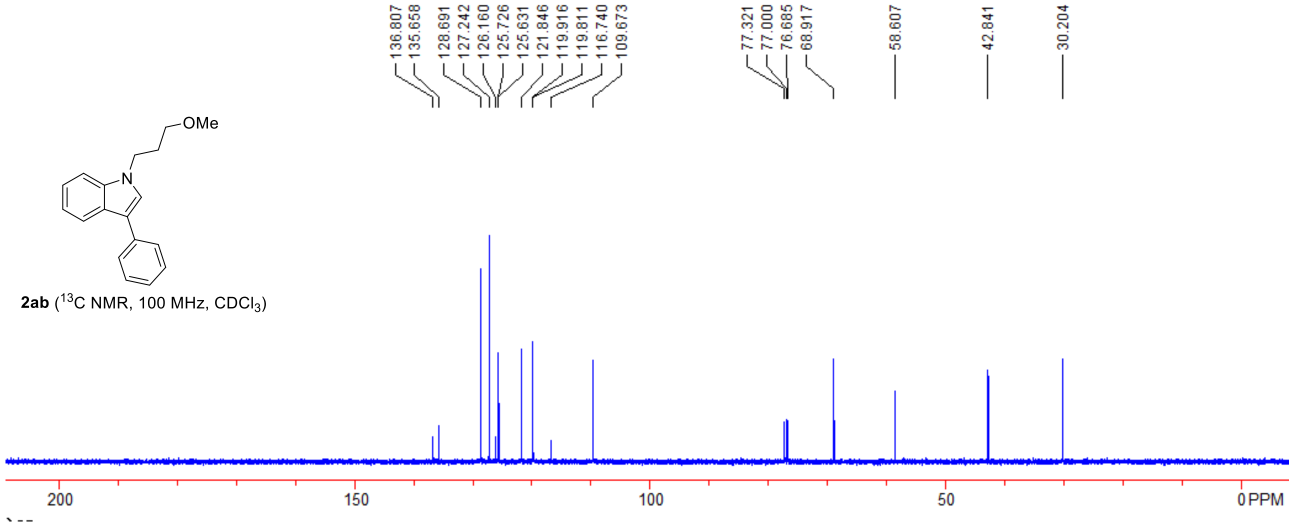

2ab (^{13}C NMR, 100 MHz, $CDCl_3$)

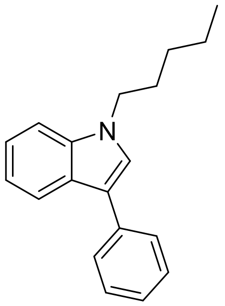

Compound 2ac: Yield: 21.6 mg, 82%; a light yellow solid; Mp: 73-75 °C; Eluent: PE/EA = 20/1. ^{1}H NMR (400 MHz, $CDCl_3$, TMS) δ 0.86 (t, *J* = 6.8 Hz, 3H), 1.20-1.36 (m, 4H), 1.75-1.86 (m, 2H), 4.05 (t, *J* = 7.2 Hz, 2H), 7.16 (t, *J* = 7.6 Hz, 1H), 7.20-7.27 (m, 3H), 7.33 (d, *J* = 8.4 Hz, 1H), 7.40 (t, *J* = 8.0 Hz, 2H), 7.65 (d, *J* = 7.2 Hz, 2H), 7.95 (d, *J* = 7.6 Hz, 1H); ^{13}C{^{1}H}-NMR (101 MHz, $CDCl_3$, TMS) δ_C 13.9, 22.3, 29.1, 29.9, 46.3, 109.7, 116.5, 119.7, 119.9, 121.7, 125.5, 125.6, 126.2, 127.2, 128.7, 135.7, 136.7; IR (neat): ν 3046, 2927, 2857, 1601, 1544, 1465, 1391, 1371, 1333, 1219, 1184, 1016, 940, 810, 765, 735, 696 cm^{-1}; HRMS (EI-TOF) Calcd for $C_{19}H_{21}N$ $[M]^+$: 263.1669, found: 263.1673.

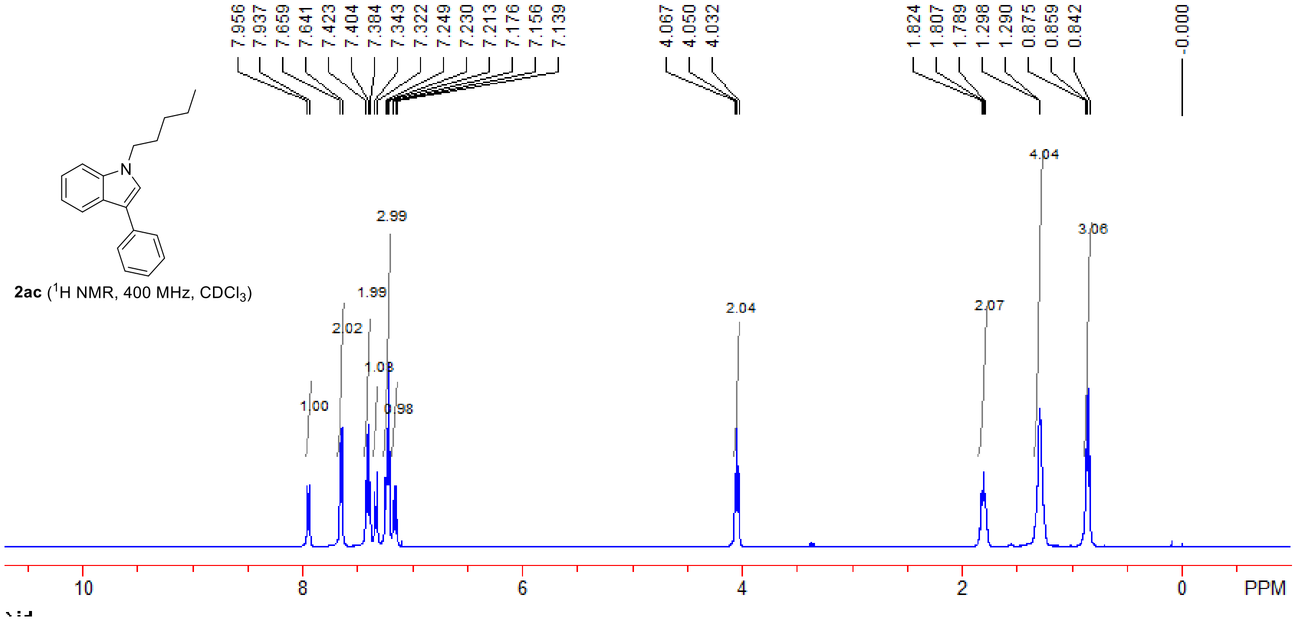

2ac (^{1}H NMR, 400 MHz, $CDCl_3$)

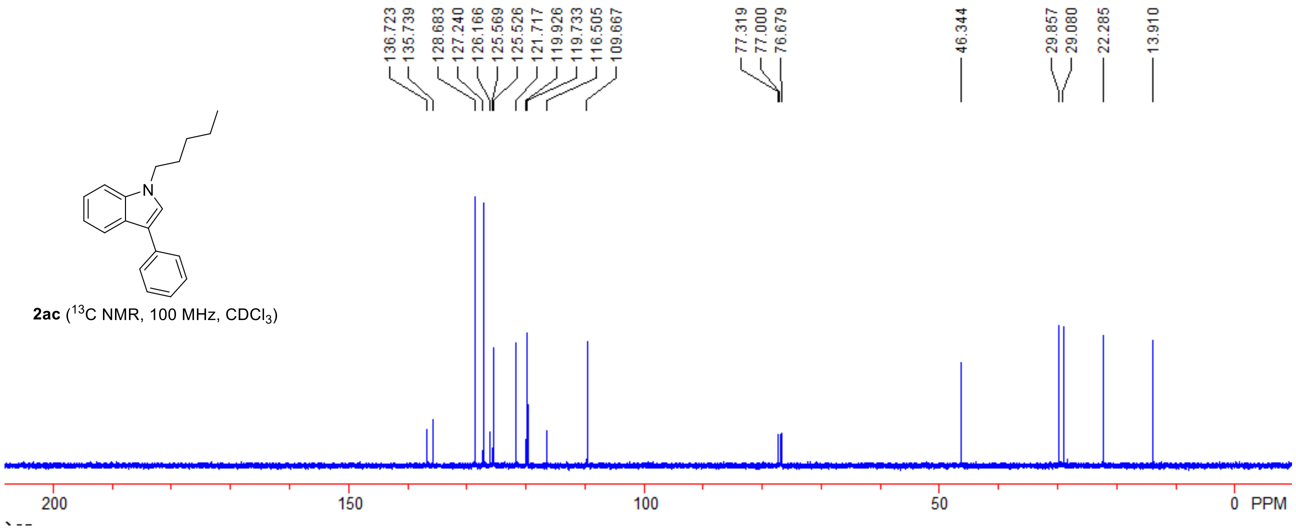

2ac (^{13}C NMR, 100 MHz, $CDCl_3$)

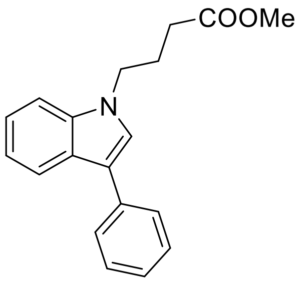

Compound 2ad: Yield: 24.6 mg, 84%; a yellow oil; Eluent: PE/EA = 20/1. ^{1}H NMR (400 MHz, $CDCl_3$, TMS) δ 2.12-2.22 (m, 2H), 2.30 (t, *J* = 6.8 Hz, 2H), 3.64 (s, 3H), 4.19 (t, *J* = 6.8 Hz, 2H), 7.17 (t, *J* = 8.0 Hz, 1H), 7.22-7.29 (m, 3H), 7.37 (d, *J* = 8.0 Hz, 1H), 7.42 (t, *J* = 7.6 Hz, 2H), 7.65 (d, *J* = 7.6 Hz, 2H), 7.94 (d, *J* = 8.0 Hz, 1H); ^{13}C{^{1}H}-NMR (101 MHz, $CDCl_3$, TMS) δ_C 25.3, 30.8, 45.2, 51.6, 109.6, 117.0, 119.9, 120.0, 122.0, 125.4, 125.7, 126.3, 127.3, 128.7, 135.5, 136.7, 173.1; IR (neat): ν 2948, 1731, 1601, 1545, 1465, 1391, 1334, 1196, 1161, 1017, 766, 737, 697 cm^{-1}; HRMS (EI-TOF) Calcd for $C_{19}H_{19}NO_2$ $[M]^+$: 293.1410, found: 293.1414.

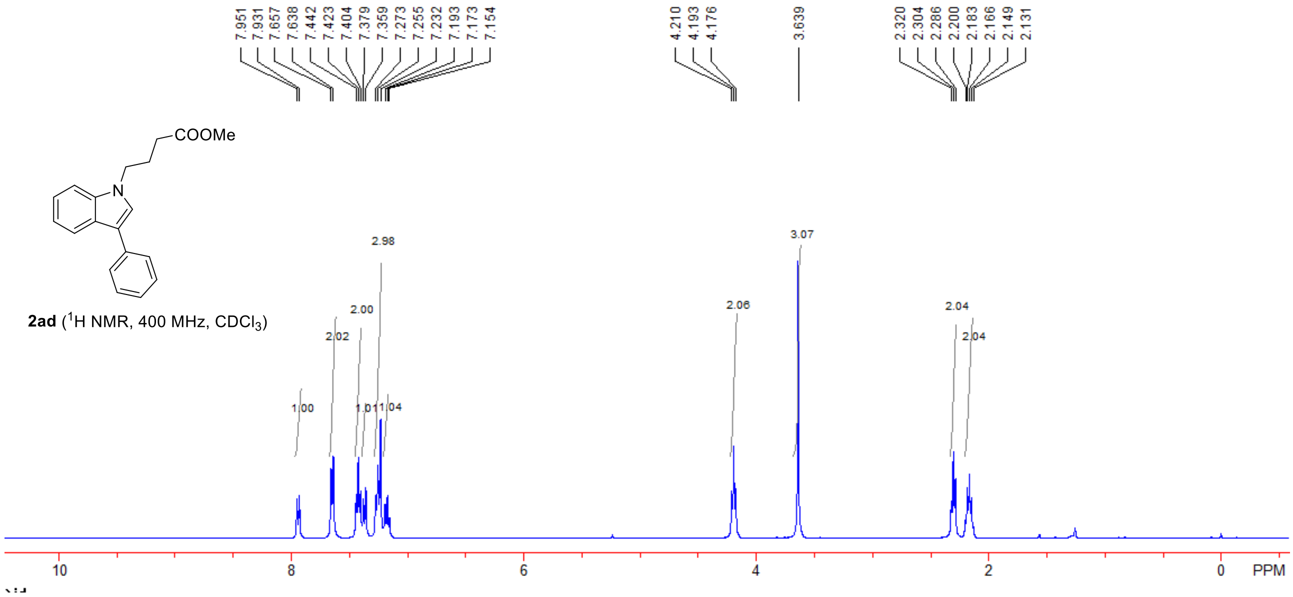

2ad (^{1}H NMR, 400 MHz, $CDCl_3$)

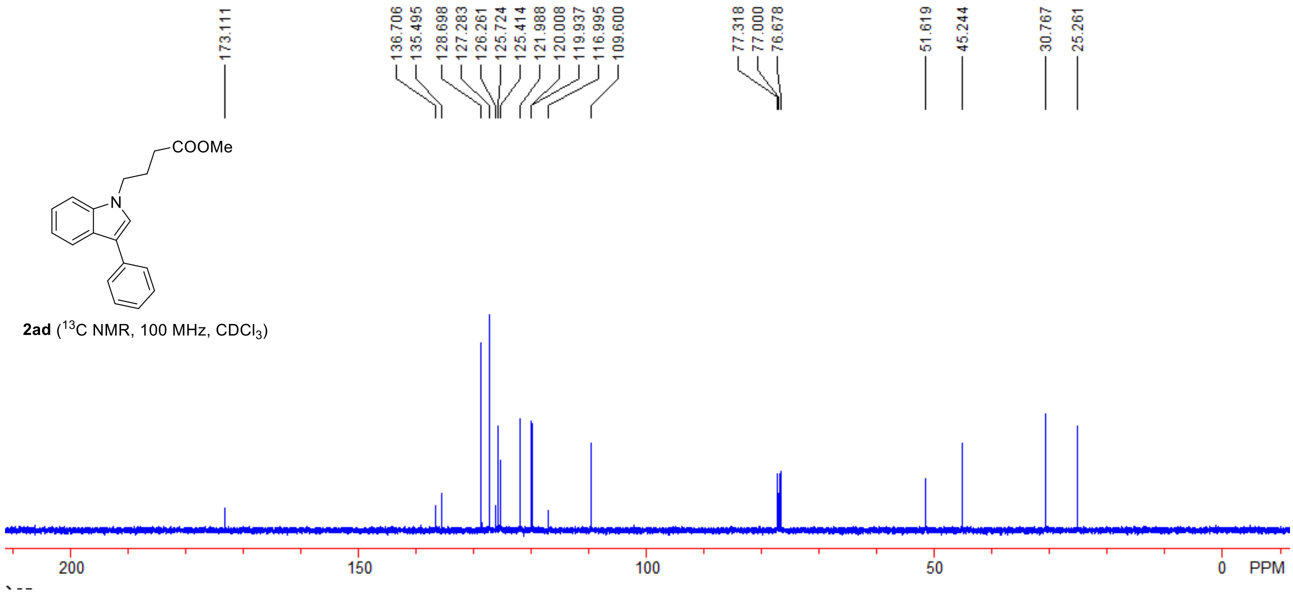

2ad (^{13}C NMR, 100 MHz, $CDCl_3$)

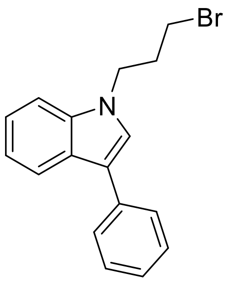

Compound 2ae: Yield: 16.9 mg, 54%; a yellow oil; Eluent: PE/EA = 20/1. ^{1}H NMR (400 MHz, $CDCl_3$, TMS) δ 2.30-2.40 (m, 2H), 3.31 (t, *J* = 6.0 Hz, 2H), 4.34 (t, *J* = 6.4 Hz, 2H), 7.16-7.22 (m, 1H), 7.23-7.32 (m, 3H), 7.37-7.48 (m, 3H), 7.65 (d, *J* = 6.8 Hz, 2H), 7.95 (d, *J* = 7.2 Hz, 1H); $^{13}C\{^1H\}$-NMR (101 MHz, $CDCl_3$, TMS) δ_C 30.5, 32.6, 44.0, 109.6, 117.2, 120.09, 120.12, 122.1, 125.6, 125.8, 126.4, 127.3, 128.8, 135.4, 136.6; IR (neat): ν 3047, 2920, 1600, 1545, 1478, 1390, 1373, 1283, 1262, 1245, 1216, 1072, 1016, 940, 766, 737, 696 cm^{-1}; HRMS (ESI-TOF) Calcd for $C_{17}H_{17}NBr$ $[M+H]^+$: 314.0539, found: 314.0543.

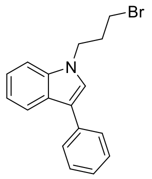

2ae (^{1}H NMR, 400 MHz, $CDCl_3$)

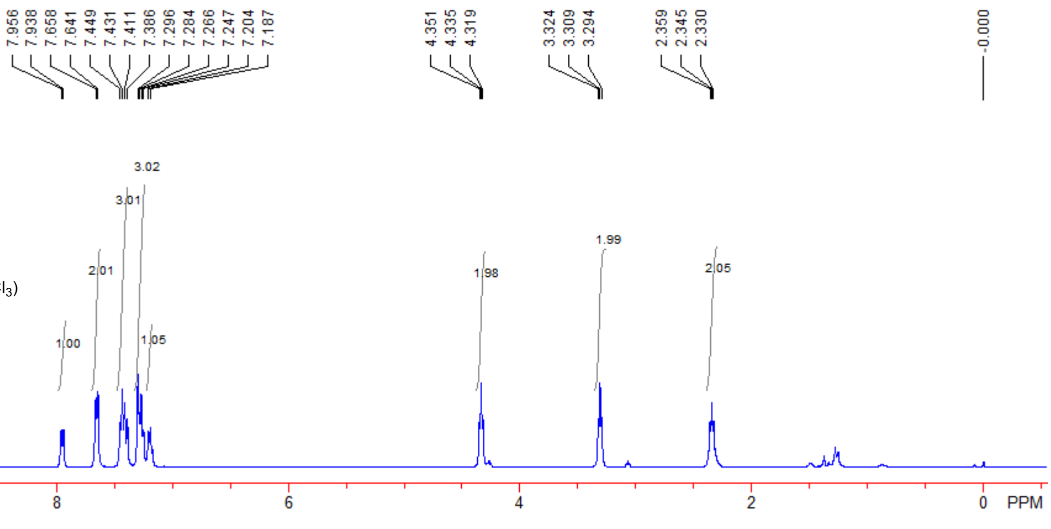

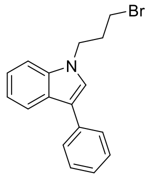

2ae (^{13}C NMR, 100 MHz, $CDCl_3$)

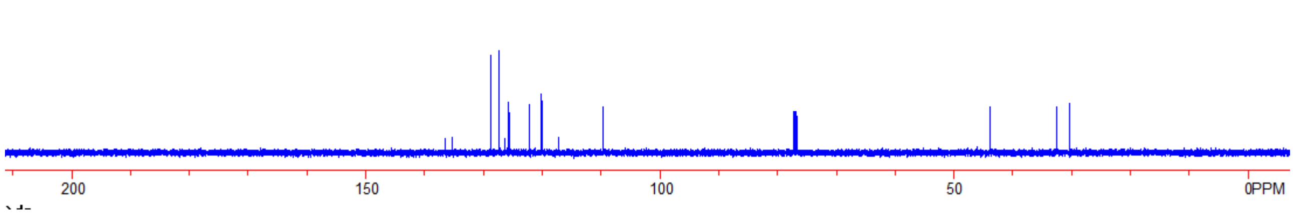

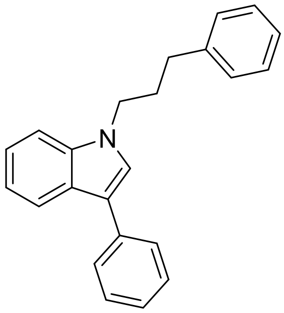

Compound 2af: Yield: 24.0 mg, 77%; a yellow oil; Eluent: PE/EA = 20/1. ^{1}H NMR (400 MHz, $CDCl_3$, TMS) δ 2.11-2.20 (m, 2H), 2.61 (t, J = 7.6 Hz, 2H), 4.08 (t, J = 7.2 Hz, 2H), 7.10-7.30 (m, 10H), 7.41 (t, J = 7.6 Hz, 2H), 7.65 (d, J = 7.6 Hz, 2H), 7.95 (d, J = 8.0 Hz, 1H); ^{13}C{^{1}H}-NMR (101 MHz, $CDCl_3$, TMS) δ_C 31.3, 32.9, 45.6, 109.7, 116.7, 119.8, 120.0, 121.8, 125.4, 125.6, 126.1, 126.2, 127.3, 128.3, 128.4, 128.7, 135.6, 136.7, 140.8; IR (neat): ν 3024, 2917, 2850, 1600, 1544, 1494, 1465, 1391, 1372, 1260, 1162, 1085, 1016, 938, 808, 766, 736, 695 cm^{-1}; HRMS (EI-TOF) Calcd for $C_{23}H_{21}N$ $[M]^+$: 311.1669, found: 311.1680.

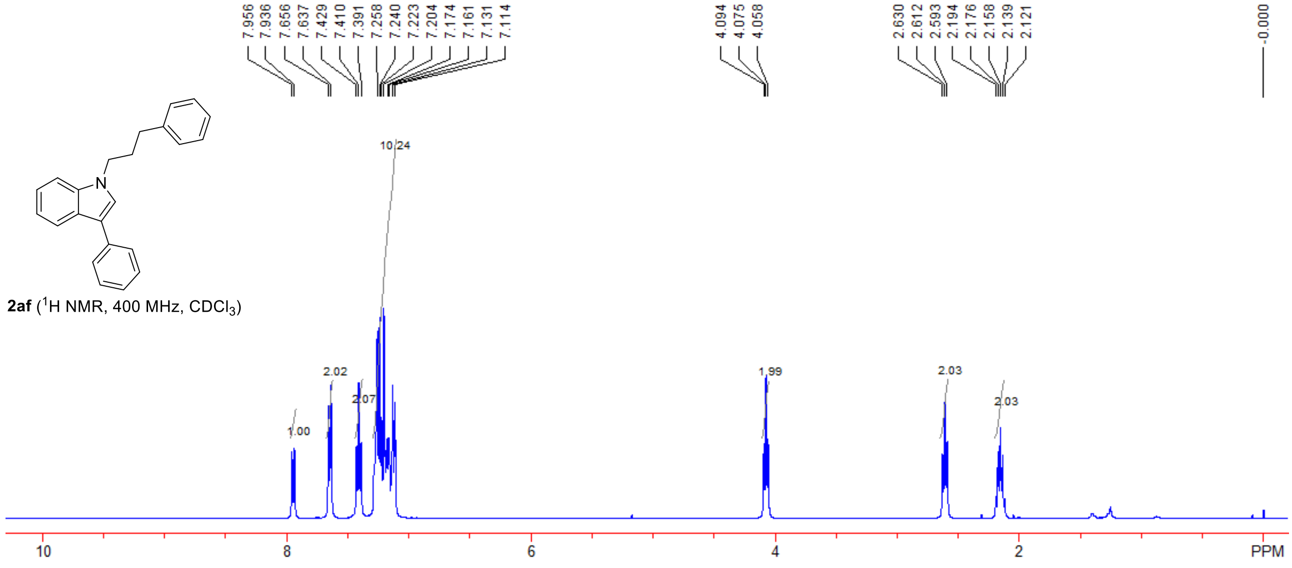

2af (^{1}H NMR, 400 MHz, $CDCl_3$)

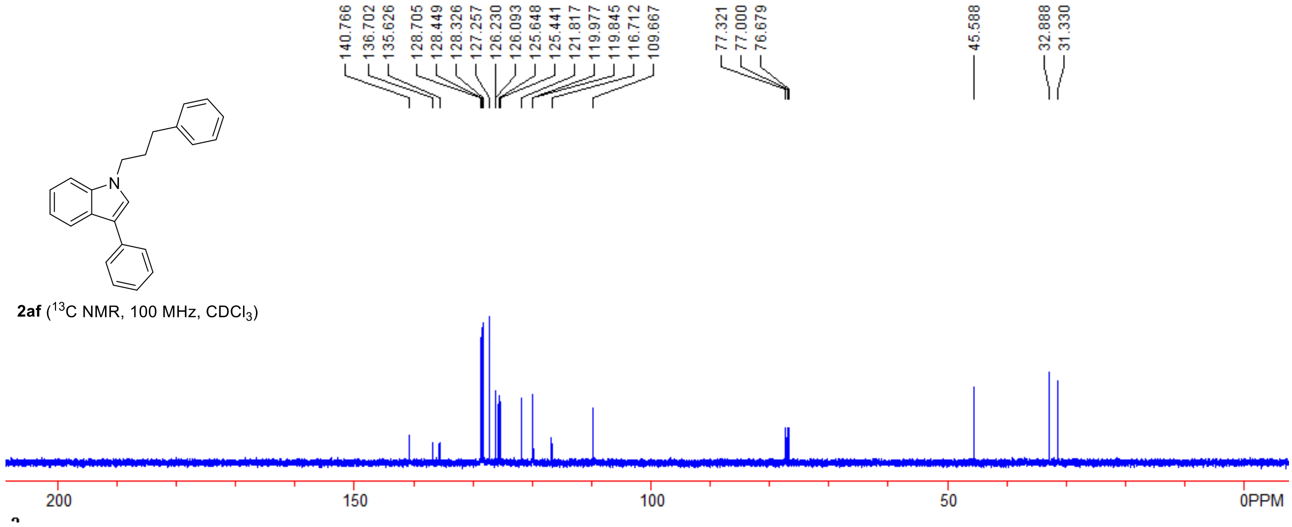

2af (^{13}C NMR, 100 MHz, $CDCl_3$)

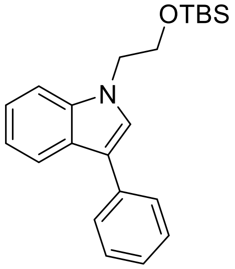

Compound 2ah: Yield: 27.0 mg, 77%; a yellow oil; Eluent: PE/EA = 20/1. ^{1}H NMR (400 MHz, $CDCl_3$, TMS) δ -0.13 (s, 6H), 0.83 (s, 9H), 3.93 (t, *J* = 5.6 Hz, 2H), 4.24 (t, *J* = 5.6 Hz, 2H), 7.13-7.20 (m, 1H), 7.21-7.29 (m, 2H), 7.31 (s, 1H), 7.36 (d, *J* = 7.6 Hz, 1H), 7.42 (t, *J* = 7.6 Hz, 2H), 7.65 (d, *J* = 7.2 Hz, 2H), 7.93 (d, *J* = 7.2 Hz, 1H); ^{13}C{^{1}H}-NMR (101 MHz, $CDCl_3$, TMS) δ_C -5.7, 18.2, 25.8, 48.7, 62.3, 109.6, 116.7, 119.8, 119.9, 121.8, 125.6, 126.3, 126.4, 127.3, 128.7, 135.8, 136.9; IR (neat): ν 2951, 2927, 2855, 1602, 1547, 1464, 1375, 1333, 1253, 1194, 1107, 936, 915, 829, 765, 736, 696 cm^{-1}; HRMS (ESI-TOF) Calcd for $C_{22}H_{30}NOSi$ $[M+H]^+$: 352.2091, found: 352.2088.

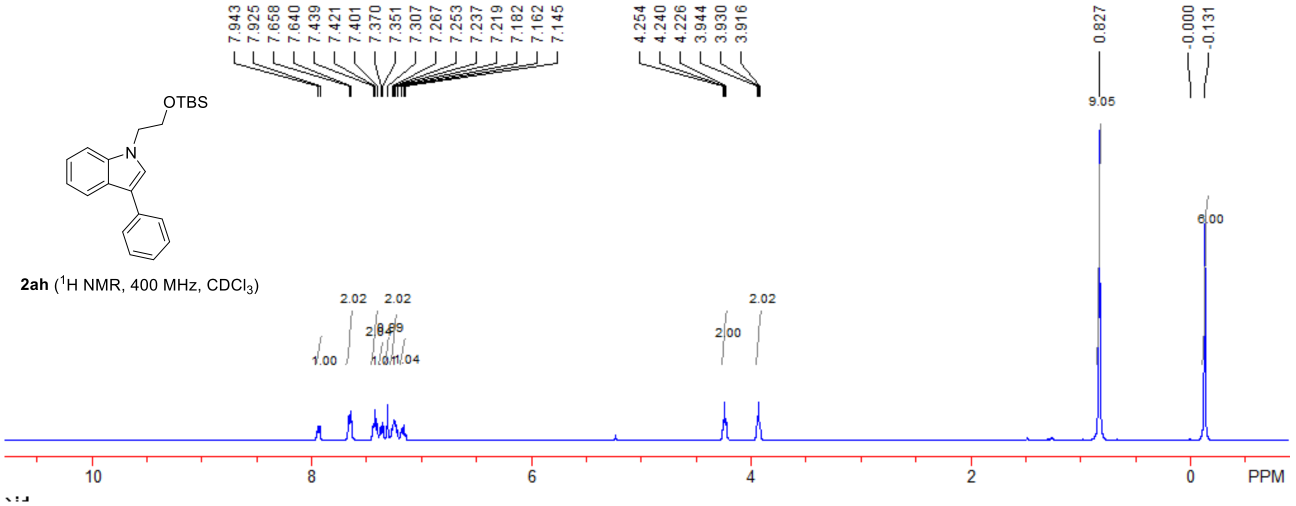

2ah (^{1}H NMR, 400 MHz, $CDCl_3$)

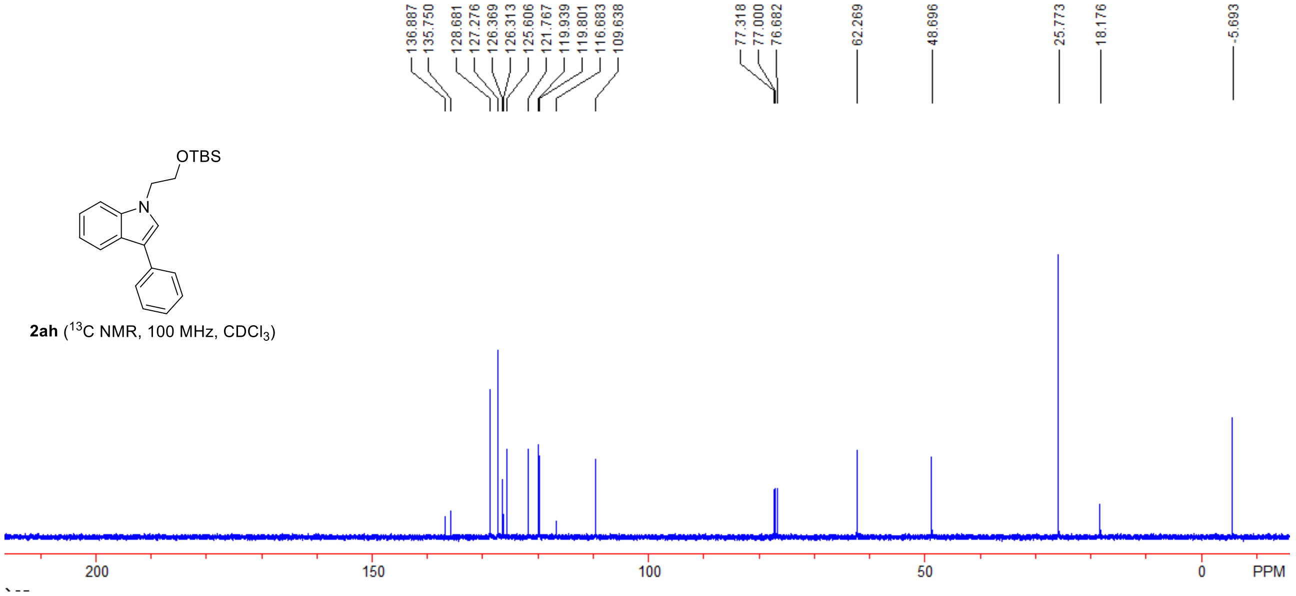

2ah (^{13}C NMR, 100 MHz, $CDCl_3$)

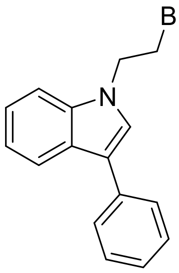

Compound 2ai: Yield: 9.6 mg, 32%; a light yellow solid; Mp: 80-82 ºC; Eluent: PE/EA = 20/1. ^{1}H NMR (400 MHz, $CDCl_3$, TMS) δ 3.58 (t, *J* = 6.8 Hz, 2H), 4.45 (t, *J* = 6.8 Hz, 2H), 7.15-7.35 (m, 5H), 7.42 (t, *J* = 7.2 Hz, 2H), 7.63 (d, *J* = 7.6 Hz, 2H), 7.94 (d, *J* = 8.0 Hz, 1H); ^{13}C{^{1}H}-NMR (101 MHz, $CDCl_3$, TMS) δ_C 29.6, 47.8, 109.2, 117.5, 120.2, 120.3, 122.3, 125.4, 126.0, 126.4, 127.4, 128.8, 135.1, 136.3; IR (neat): ν 3042, 2921, 2849, 1599, 1543, 1478, 1464, 1374, 1328, 1209, 1145, 1072, 1016, 983, 914, 764, 733, 694 cm^{-1}; HRMS (ESI-TOF) Calcd for $C_{16}H_{15}NBr$ $[M+H]^+$: 300.0382, found: 300.0382.

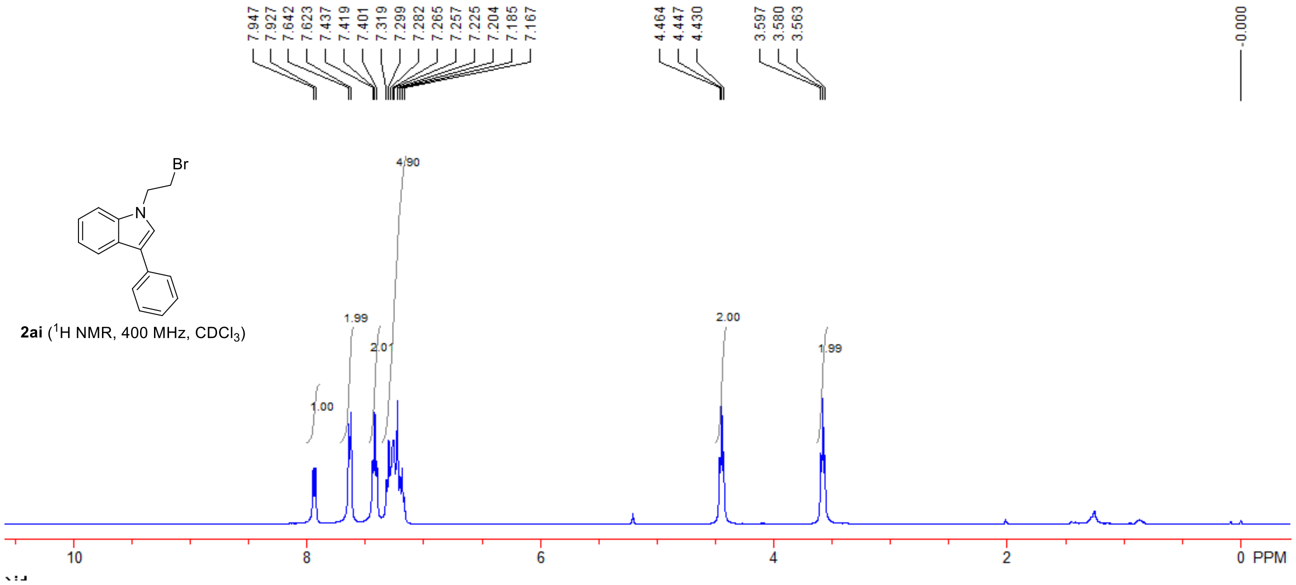

2ai (^{1}H NMR, 400 MHz, $CDCl_3$)

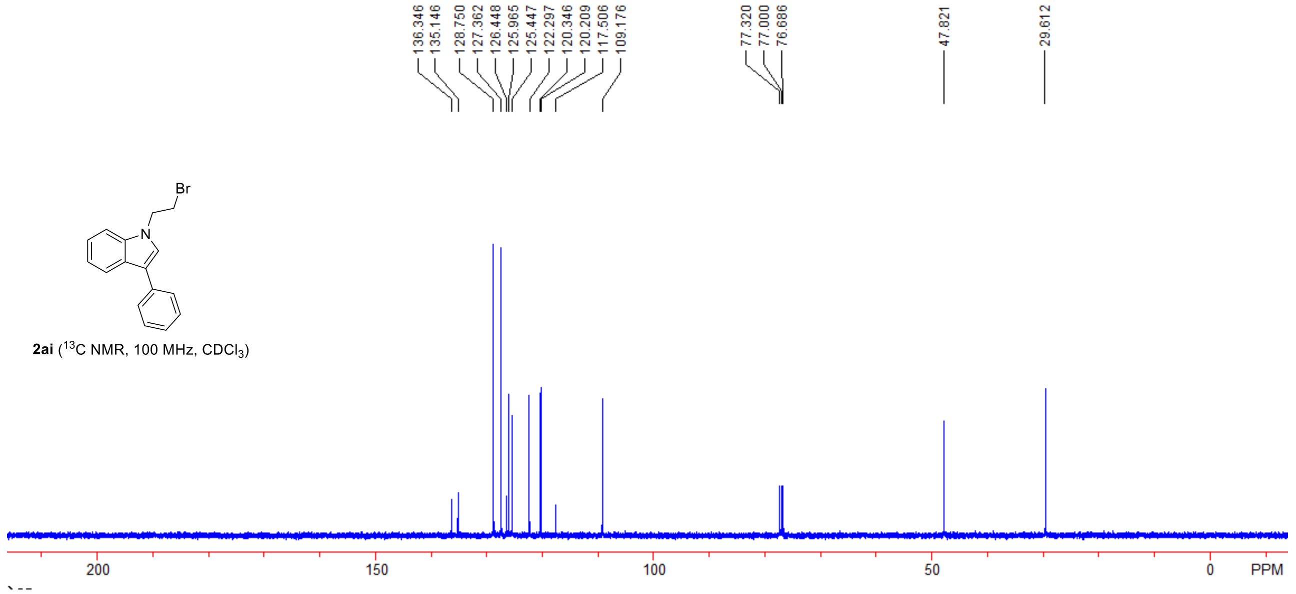

2ai (^{13}C NMR, 100 MHz, $CDCl_3$)

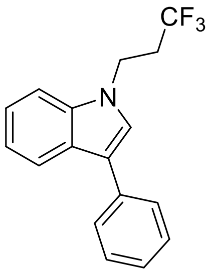

Compound 2aj: Yield: 16.2 mg, 56%; a light yellow solid; Mp: 46-48 ^{o}C; Eluent: PE/EA = 20/1. ^{1}H NMR (400 MHz, $CDCl_3$, TMS) δ 2.45-2.65 (m, 2H), 4.29 (t, *J* = 7.6 Hz, 2H), 7.12 (s, 1H), 7.15-7.21 (m, 1H), 7.22-7.30 (m, 3H), 7.40 (t, *J* = 7.6 Hz, 2H), 7.61 (d, *J* = 7.6 Hz, 2H), 7.93 (d, *J* = 8.0 Hz, 1H); ^{13}C{^{1}H}-NMR (101 MHz, $CDCl_3$, TMS) δ_C 34.3 (q, *J* = 28.3 Hz), 39.4 (q, *J* = 4.1 Hz), 109.0, 117.8, 120.3, 120.4, 122.4, 125.0, 125.7 (q, *J* = 275.7 Hz), 126.0, 126.5, 127.3, 128.8, 135.1, 136.2; ^{19}F NMR (376 MHz, $CDCl_3$) δ -65.4; IR (neat): ν 3052, 2922, 1600, 1545, 1468, 1391, 1336, 1302, 1239, 1199, 1130, 1107, 1069, 988, 848, 764, 736, 696 cm^{-1}; HRMS (FI-TOF) Calcd for $C_{17}H_{14}NF_3$ $[M]^+$: 289.1073, found: 289.1077.

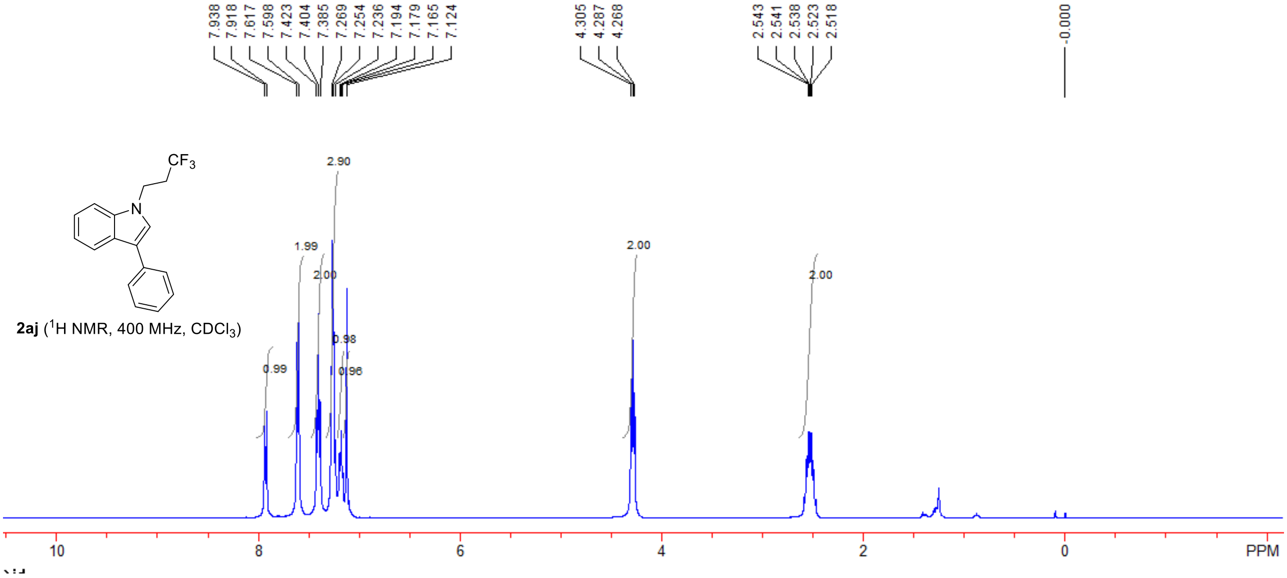

2aj (^{1}H NMR, 400 MHz, $CDCl_3$)

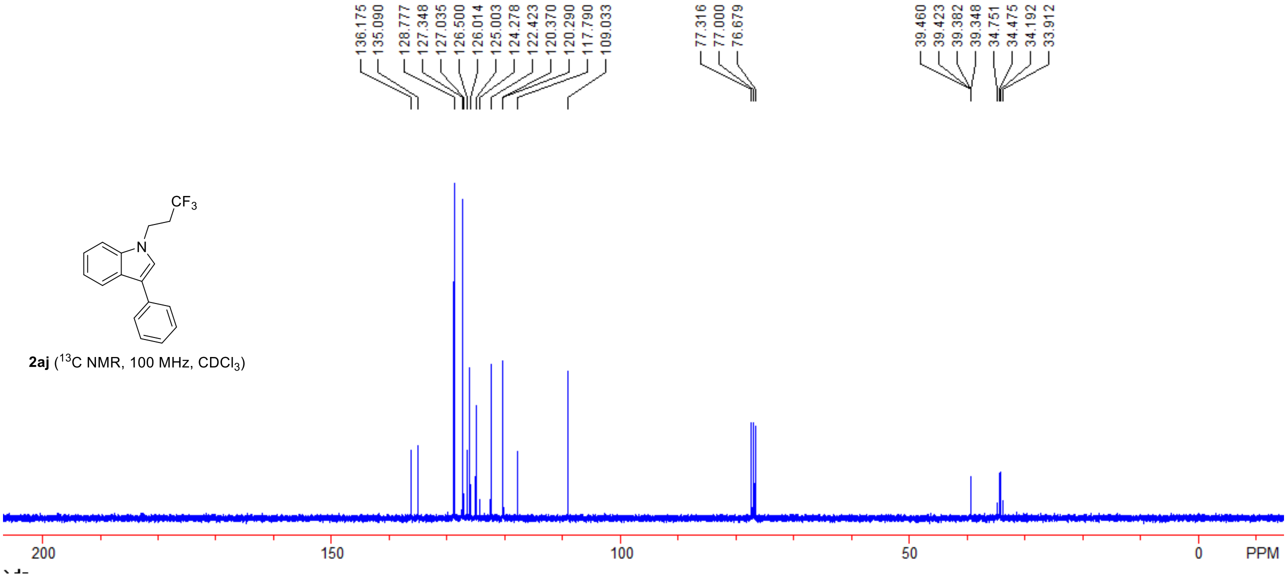

2aj (^{13}C NMR, 100 MHz, $CDCl_3$)

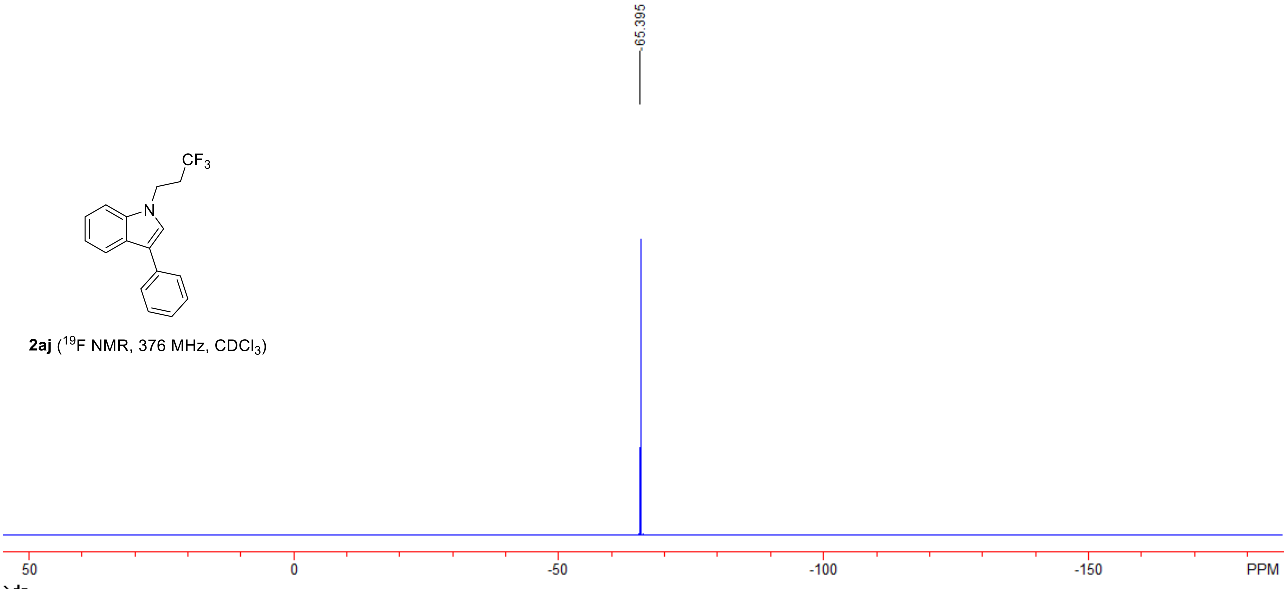

2aj (^{19}F NMR, 376 MHz, $CDCl_3$)

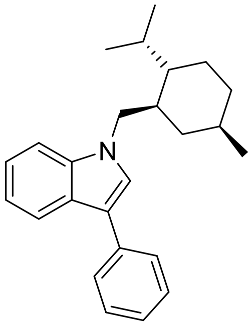

Compound 2am: Yield: 15.9 mg, 46%; a light yellow solid; Mp: 148-150 °C; Eluent: PE/EA = 20/1. ^{1}H NMR (400 MHz, $CDCl_3$, TMS) 0.63 (q, J = 12.0 Hz, 1H), 0.74 (d, J = 6.4 Hz, 3H), 0.79-0.91 (m, 1H), 0.97 (d, J = 6.8 Hz, 3H), 1.01 (d, J = 6.8 Hz, 3H), 1.05-1.22 (m, 3H), 1.39 (d, J = 12.4 Hz, 1H), 1.66-1.76 (m, 2H), 1.83-1.96 (m, 1H), 2.14-2.22 (m, 1H), 3.58-3.67 (m, 1H), 4.52 (dd, J_1 = 14.0 Hz, J_2 = 3.6 Hz, 1H), 7.14-7.30 (m, 4H), 7.36 (d, J = 8.0 Hz, 1H), 7.44 (t, J = 8.0 Hz, 2H), 7.68 (d, J = 7.2 Hz, 2H), 7.96 (d, J = 8.0 Hz, 1H); ^{13}C{^{1}H}-NMR (101MHz, $CDCl_3$, TMS) δ_C 15.6, 21.5, 22.5, 24.1, 26.9, 32.0, 34.9, 39.43, 39.45, 46.2, 50.1, 109.8, 116.3, 119.7, 119.9, 121.7, 125.6, 126.1, 126.6, 127.2, 128.7, 135.7, 137.0; IR (neat): ν 2953, 2916, 2839, 1599, 1545, 1478, 1394, 1260, 1141, 1070, 1001, 921, 821, 735, 696 cm^{-1}; HRMS (ESI-TOF) Calcd for $C_{25}H_{32}N$ $[M+H]^+$: 346.2529, found: 346.2535.

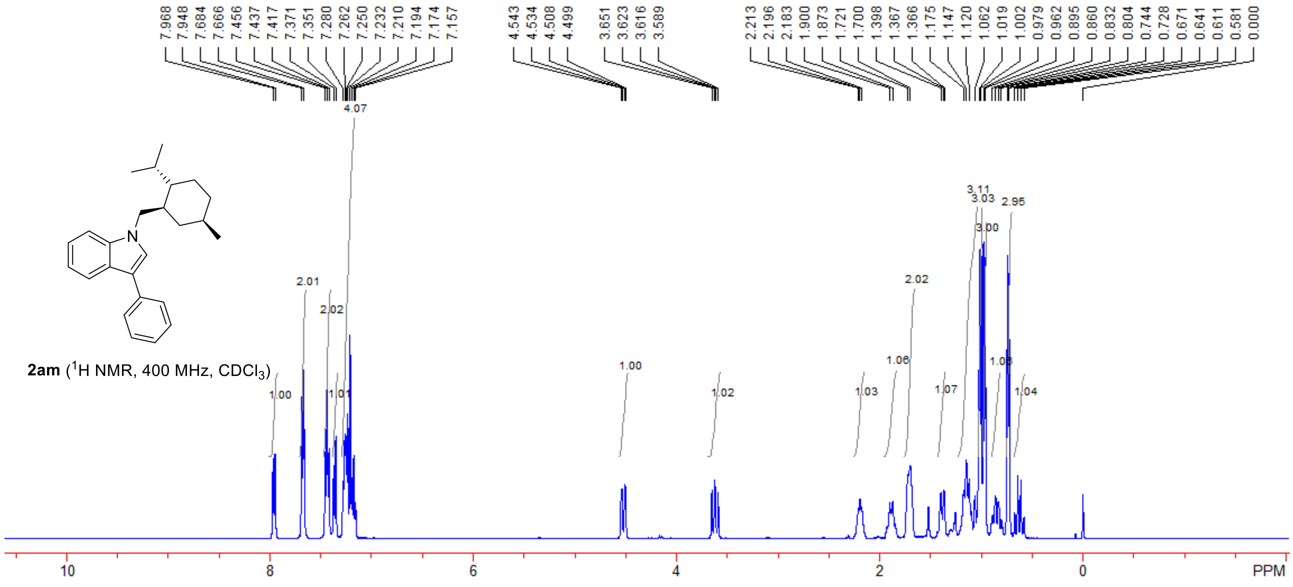

2am (^{1}H NMR, 400 MHz, $CDCl_3$)

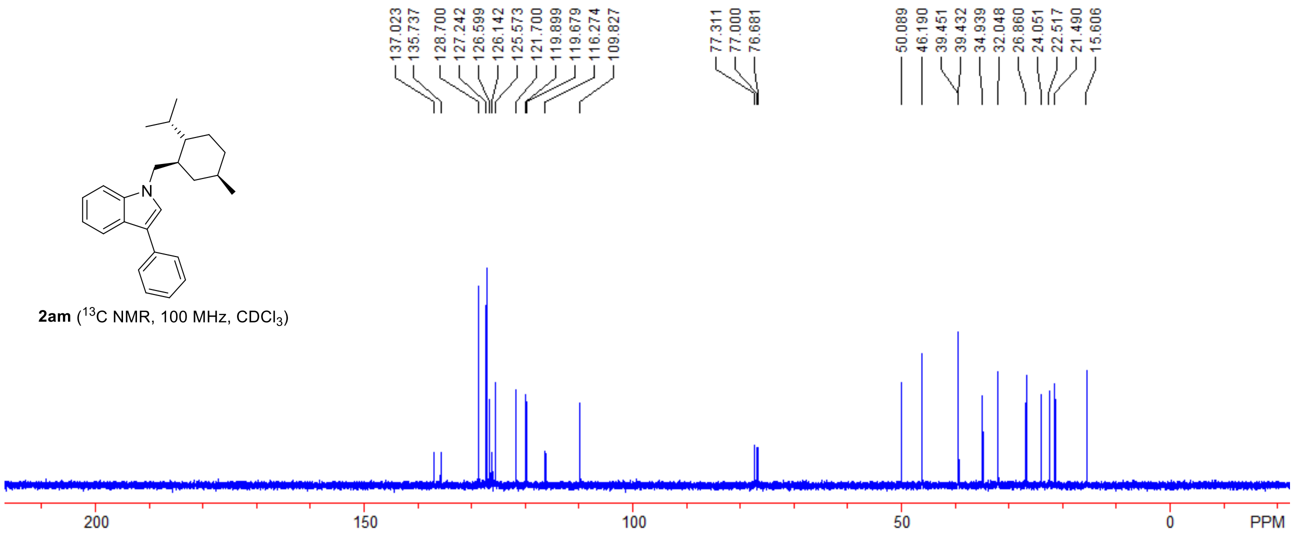

2am (^{13}C NMR, 100 MHz, $CDCl_3$)

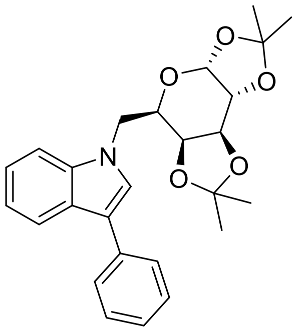

Compound 2an: Yield: 32.6 mg, 75%; a light yellow solid; Mp: 109-111 °C; Eluent: PE/EA = 10/1. ^{1}H NMR (400 MHz, $CDCl_3$, TMS) δ 1.29 (s, 3H), 1.34 (s, 3H), 1.42 (s, 3H), 1.55 (s, 3H), 4.00 (d, *J* = 8.0 Hz, 1H), 4.17-4.50 (m, 4H), 4.53 (d, *J* = 7.6 Hz, 1H), 5.56 (d, *J* = 4.8 Hz, 1H), 7.17 (t, *J* = 7.2 Hz, 1H), 7.26 (t, *J* = 7.2 Hz, 2H), 7.40-7.48 (m, 4H), 7.66 (d, *J* = 7.2 Hz, 2H), 7.94 (d, *J* = 7.6 Hz, 1H); ^{13}C{^{1}H}-NMR (101 MHz, $CDCl_3$, TMS) δ_C 24.4, 24.8, 25.97, 26.01, 45.8, 65.9, 70.46, 70.56, 96.4, 108.7, 109.4, 109.6, 117.0, 119.96, 120.02, 122.0, 125.7, 126.3, 127.3, 128.7, 135.6, 137.0; IR (neat): ν 2988, 2918, 2849, 1644, 1601, 1546, 1468, 1395, 1334, 1252, 1165, 1037, 999, 917, 885, 765, 699 cm^{-1}; HRMS (ESI-TOF) Calcd for $C_{26}H_{30}NO_5$ [M+H]$^{+}$: 436.2119, found: 436.2120.

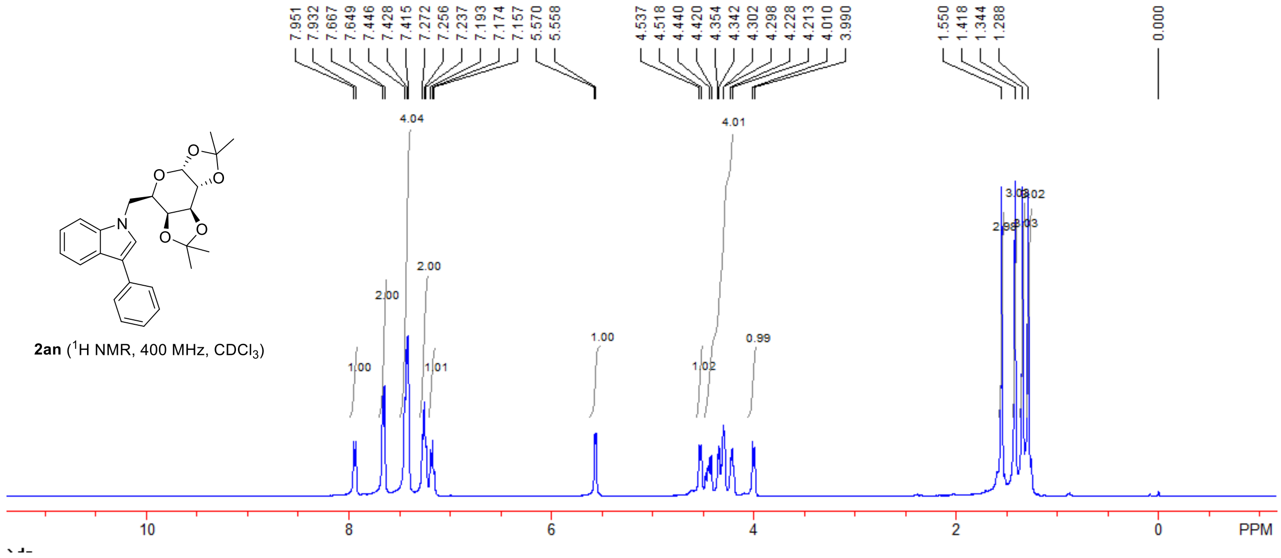

2an (^{1}H NMR, 400 MHz, $CDCl_3$)

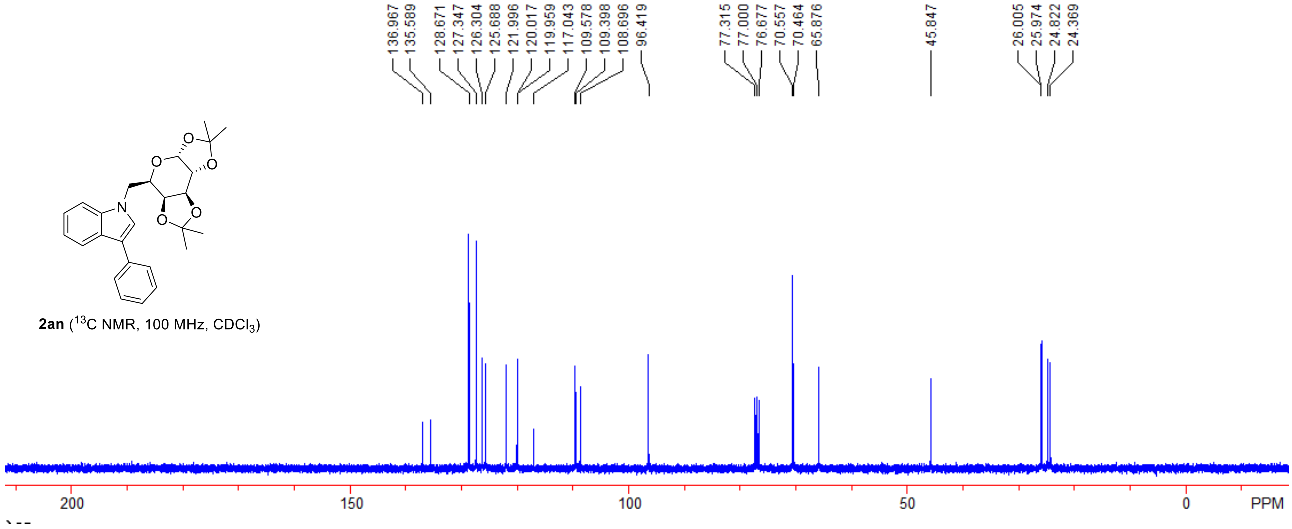

2an (^{13}C NMR, 100 MHz, $CDCl_3$)

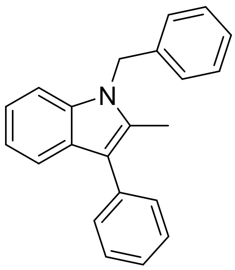

Compound 2ao:[16] Yield: 12.8 mg, 43%; a yellow oil; Eluent: PE/EA = 20/1. ^{1}H NMR (400 MHz, $CDCl_3$, TMS) δ 2.41 (s, 3H), 5.35 (s, 2H), 7.02 (d, *J* = 7.2 Hz, 2H), 7.08-7.20 (m, 2H), 7.21-7.33 (m, 5H), 7.45 (t, *J* = 7.6 Hz, 2H), 7.52 (d, *J* = 6.8 Hz, 2H), 7.71 (d, *J* = 7.2 Hz, 1H); $^{13}C\{^{1}H\}$-NMR (101 MHz, $CDCl_3$, TMS) δ_C 11.0, 46.6, 109.2, 114.6, 118.8, 119.9, 121.4, 125.8, 126.0, 127.2, 127.3, 128.4, 128.8, 129.7, 133.2, 135.6, 136.5, 137.7.

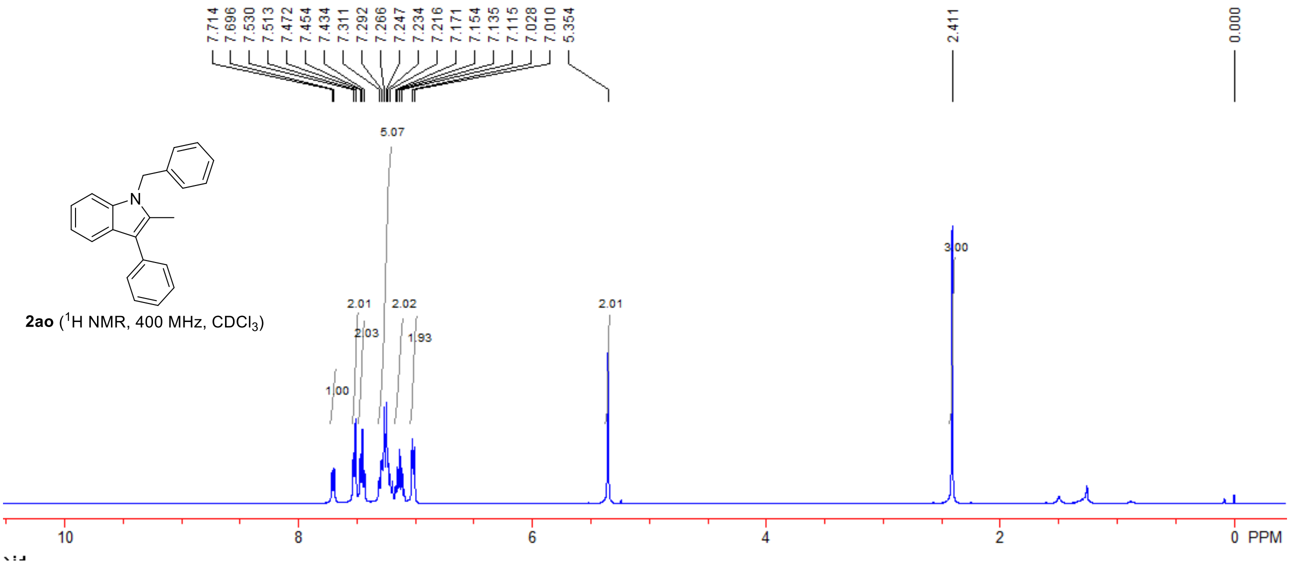

2ao (^{1}H NMR, 400 MHz, $CDCl_3$)

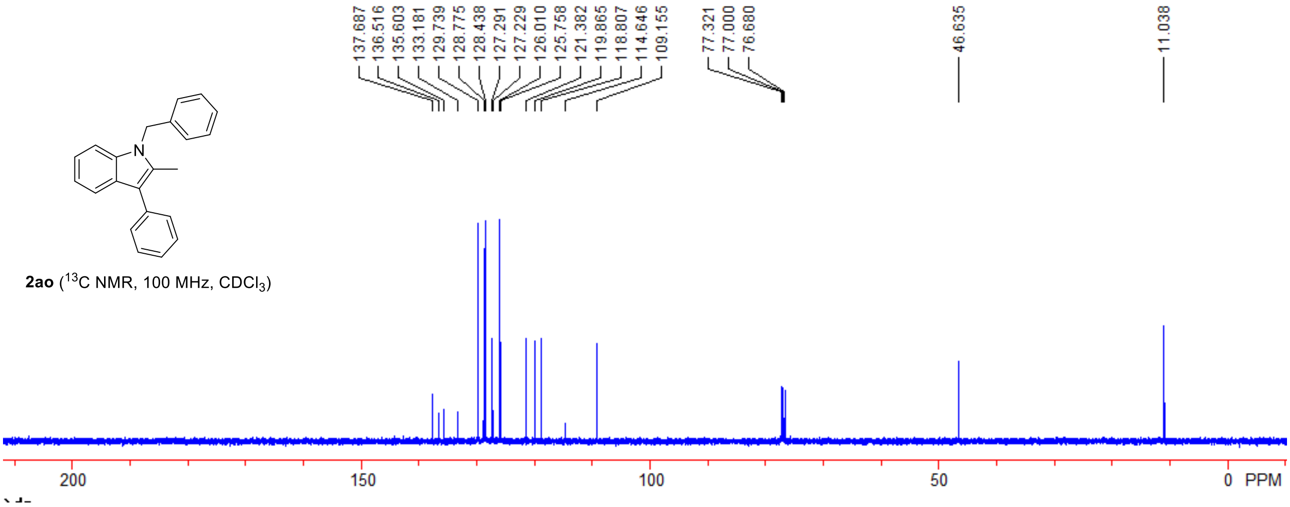

2ao (^{13}C NMR, 100 MHz, $CDCl_3$)

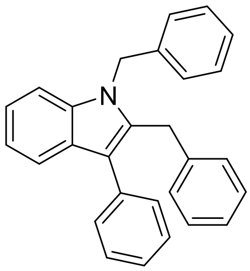

Compound 2ap: Yield: 7.8 mg, 21%; a light yellow solid; Mp: 131-133 °C; Eluent: PE/EA = 20/1. ^{1}H NMR (400 MHz, $CDCl_3$, TMS) δ 4.15 (s, 2H), 5.09 (s, 2H), 6.84-6.92 (m, 2H), 7.03-7.10 (m, 2H), 7.12-7.30 (m, 10H), 7.34-7.43 (m, 2H), 7.50-7.58 (m, 2H), 7.74-7.81 (m, 1H); ^{13}C{^{1}H}-NMR (101 MHz, $CDCl_3$, TMS) δ_C 30.8, 46.7, 109.5, 116.7, 119.4, 120.0, 121.9, 125.9, 126.1, 126.4, 127.2, 127.3, 127.9, 128.6, 128.7, 129.4, 134.4, 135.2, 136.9, 137.7, 138.9; IR (neat): ν 3024, 2915, 1600, 1555, 1494, 1431, 1407, 1306, 1205, 1093, 1001, 936, 874, 772, 716 cm^{-1}; HRMS (EI-TOF) Calcd for $C_{28}H_{23}N$ $[M]^+$: 373.1825, found: 373.1840.

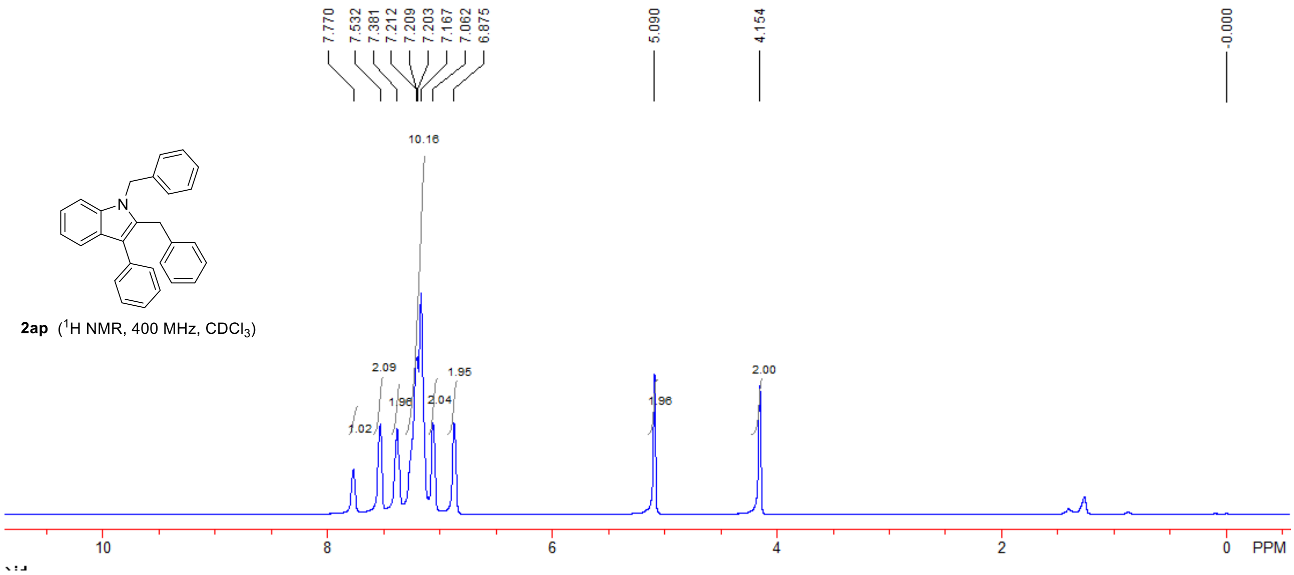

2ap (^{1}H NMR, 400 MHz, $CDCl_3$)

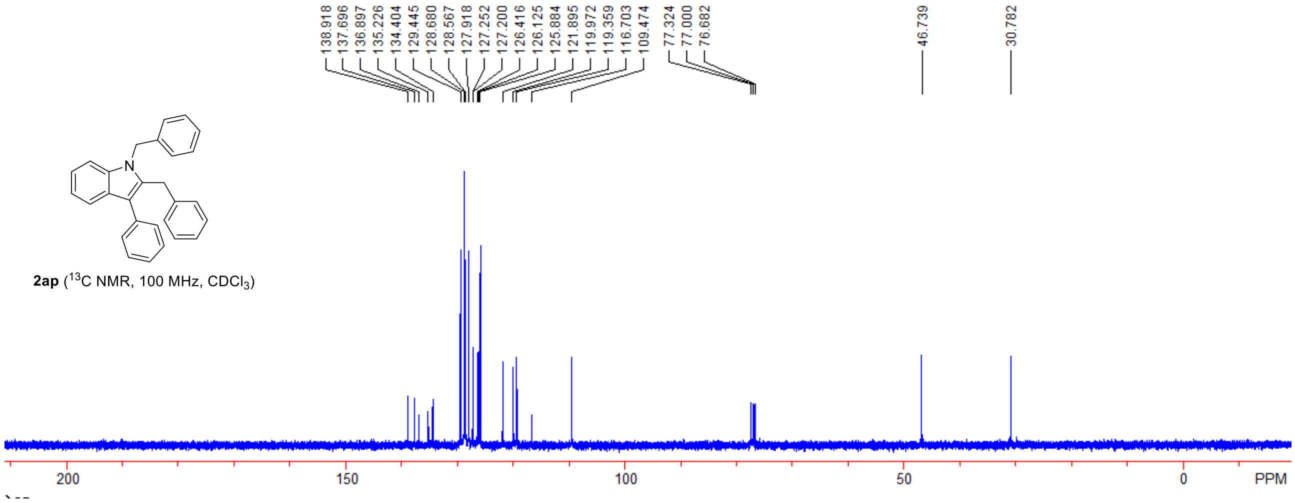

2ap (^{13}C NMR, 100 MHz, $CDCl_3$)

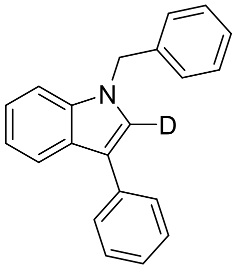

Compound [*D*]-**2a**: Yield: 23.6 mg, 83%, D containing 93%; a light yellow solid; Mp: 65-67 ^{o}C; Eluent: PE/EA = 20/1. ^{1}H NMR (400 MHz, $CDCl_3$, TMS) δ 5.21 (s, 2H), 7.08 (d, *J* = 6.8 Hz, 2H), 7.13-7.29 (m, 7H), 7.39 (t, *J* = 7.6 Hz, 2H), 7.64 (d, *J* = 7.6 Hz, 2H), 7.96 (d, *J* = 6.8 Hz, 1H); ^{13}C{^{1}H}-NMR (101 MHz, $CDCl_3$, TMS) δ_C 49.9, 110.0, 117.1, 120.0, 120.1, 122.1, 125.7, 126.4, 126.8, 127.3, 127.6, 128.70, 128.73, 135.5, 137.0, 137.1; IR (neat): ν 3028, 2922, 1598, 1520, 1464, 1385, 1297, 1069, 940, 890, 770, 749, 727, 694 cm^{-1}; HRMS (DART-LTQFTICR) Calcd for $C_{21}H_{17}DN$ [M+H]$^{+}$: 285.1497, found: 285.1495.

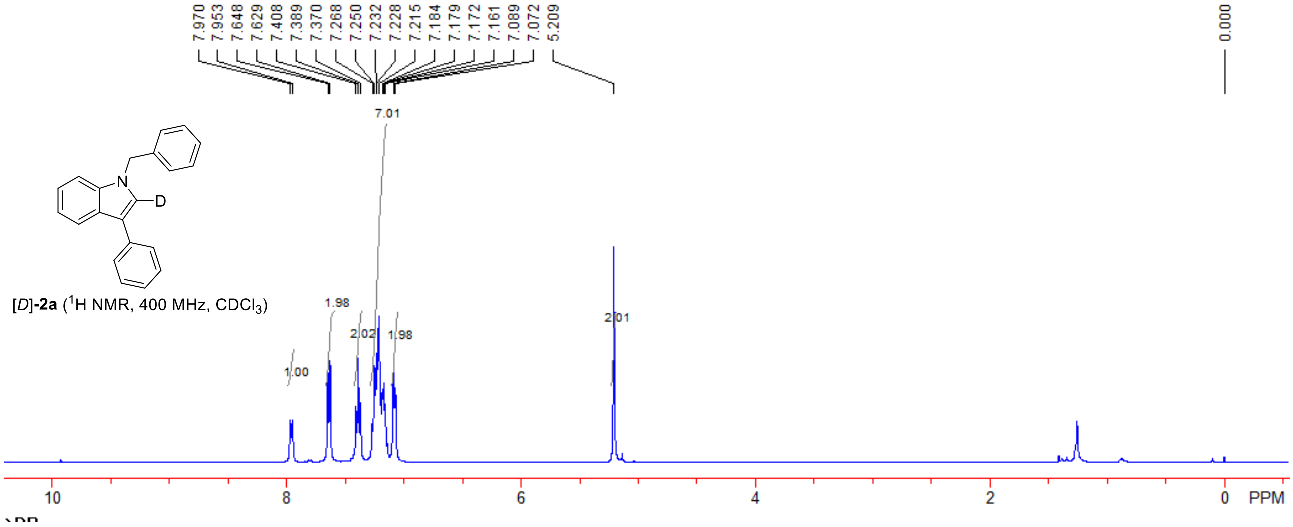

[*D*]-**2a** (^{1}H NMR, 400 MHz, $CDCl_3$)

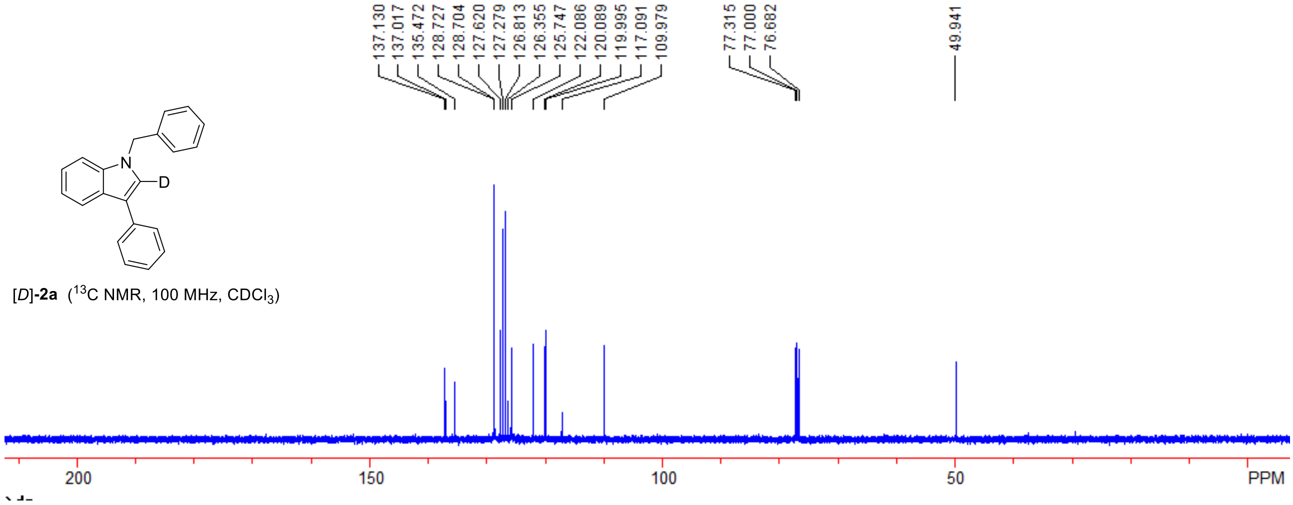

[*D*]-**2a** (^{13}C NMR, 100 MHz, $CDCl_3$)

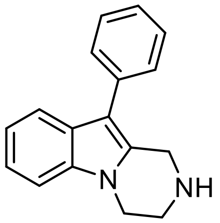

Compound 11a: Yield: 37.7 mg, 76%; a light yellow solid; Mp: 98-100 °C; Eluent: PE/EA = 2/1. ^{1}H NMR (400 MHz, $CDCl_3$, TMS) δ 7.76 (d, *J* = 7.8 Hz, 1H), 7.48–7.39 (m, 4H), 7.32–7.25 (m, 2H), 7.22–7.13 (m, 2H), 4.11 (t, *J* = 5.6 Hz, 2H), 4.01 (s, 2H), 3.36 (s, 1H), 3.15 (t, *J* = 5.6 Hz, 2H); ^{13}C{^{1}H}-NMR (101 MHz, $CDCl_3$, TMS) δ_C 136.1, 135.0, 130.9, 128.8, 128.6, 126.9, 125.7, 121.3, 120.4, 119.1, 111.6, 108.8, 49.5, 48.2, 41.9; IR (neat): ν 3668, 3397, 2970, 1602, 1455, 1320, 1260, 1074, 747, 701 cm^{-1}; HRMS (ESI-TOF) Calcd for $C_{17}H_{17}N_2$ $[M+H]^+$: 249.1386, found: 249.1389.

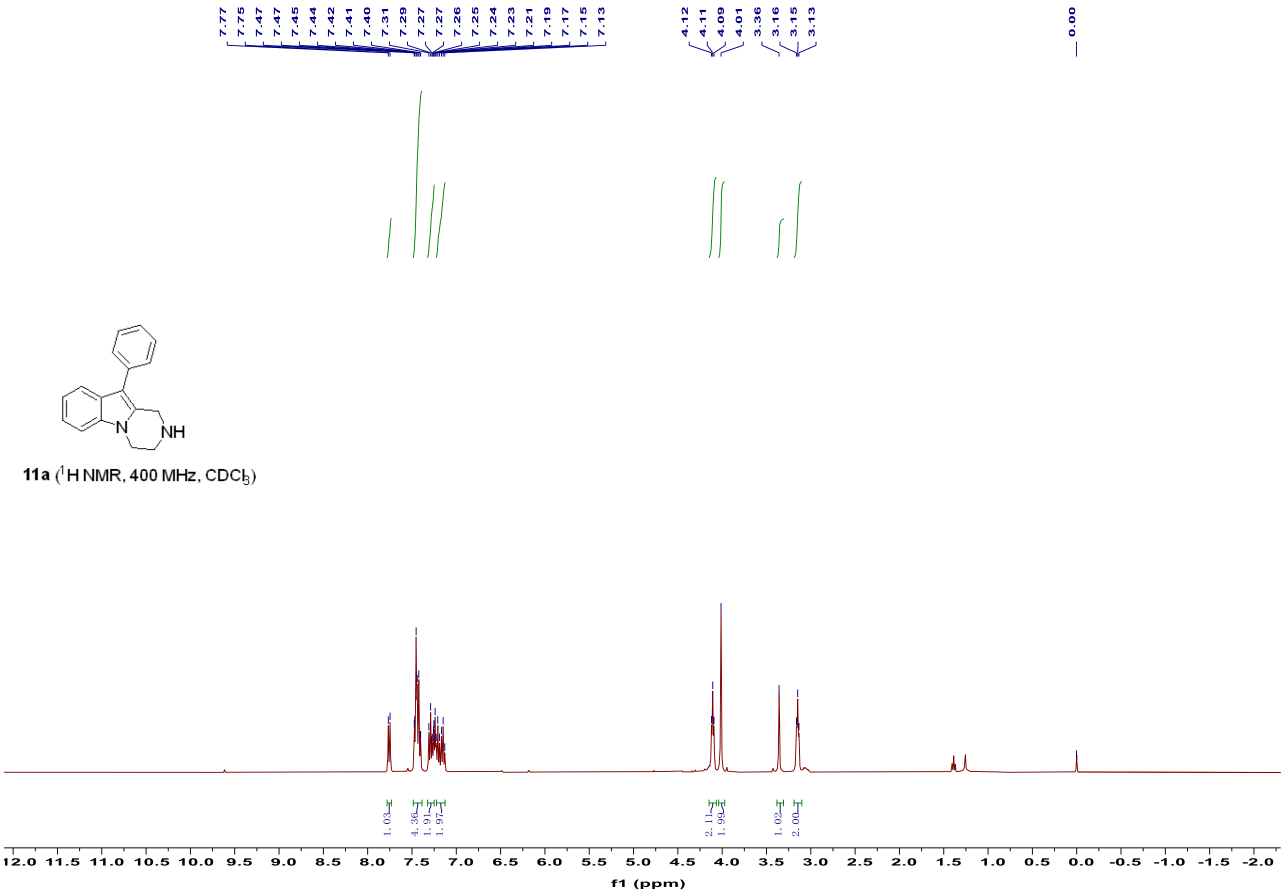

11a (^{1}H NMR, 400 MHz, $CDCl_3$)

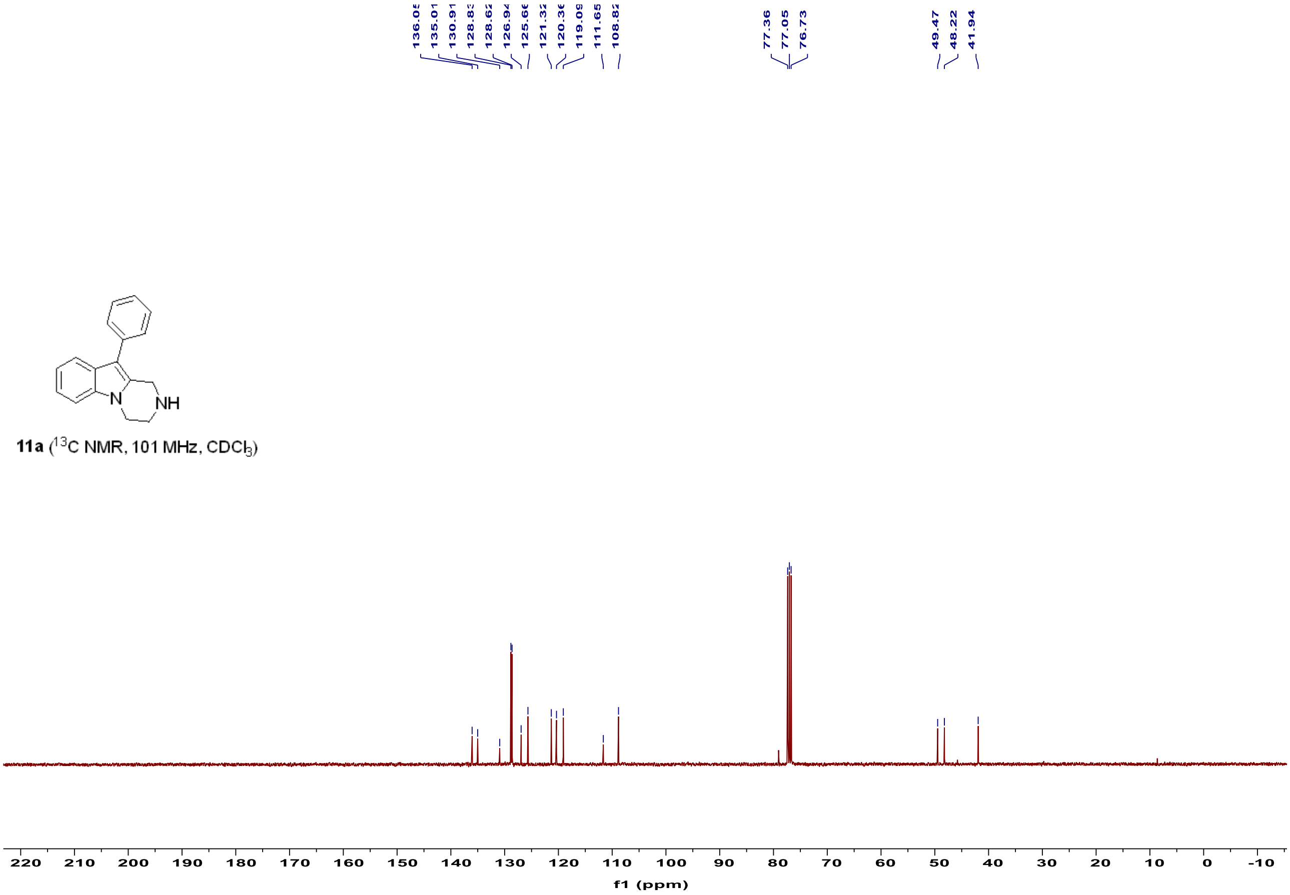

11a (^{13}C NMR, 101 MHz, $CDCl_3$)

10. Computational details

All quantum mechanical calculations have been performed with Gaussian 16. The geometries of all species have been optimized at B3LYP/6-31G(d) level. The subsequent frequency calculations on the stationary points were carried out at the same level of theory to ascertain the nature of the stationary points as minima on the respective potential energy surfaces. The conformational space of flexible systems has first been searched manually and checked by xtb 6.0 program.[17] Thermochemical corrections to 298.15 K have been calculated for all minima from unscaled vibrational frequencies obtained at this same level. The thermochemical corrections have been combined with single-point energies calculated at the SMD/B3LYP/6-311+G(d,p)// B3LYP/6-31G(d) level to yield free energy G_{298} at 298.15 K. The solvent effect was estimated by the IEFPCM method with radii and nonelectrostatic terms for SMD salvation model in acetonitrile ($\varepsilon = 35.688$).

The Gibbs activation energy of SET process is obtained based on Marcus-Hush theory.[18]

Table S4. The total energies, enthalpies and free energies of all species in acetonitrile shown in Scheme 7a.

	E_{tot}	H_{298}	G_{298}
**1a**	-1685.724311	-1685.23542	-1685.327013
**1a**$^{\cdot+}$	-1685.517362	-1685.02848	-1685.120565
**TS1**	-1685.508294	-1685.0199	-1685.109127
**INT1**	-1685.539571	-1685.04901	-1685.138892
**INT2**	-1685.767508	-1685.767508	-1685.767508
**TS2**	-1685.75244	-1685.26924	-1685.361068
**TsH**	-820.2882496	-820.139865	-820.1866468
**2a**	-865.4799819	-865.141256	-865.2064329
**PC**$^{\cdot}$	-1875.859578	-1875.13736	-1875.260156
**PC**	-1875.727313	-1875.00228	-1875.121490
**1n**$^{\cdot+}$	-2022.665044	-2022.16788	-2022.270353
**TS1-1n**	-2022.654569	-2022.1579	-2022.257285
**1q**$^{\cdot+}$	-1800.085154	-1799.56066	-1799.658583
**TS1-1q**	-1800.071513	-1799.54742	-1799.642378

Archive entries

1a


```
1\1\GINC-OMC-1-819\SP\RB3LYP\6-311+G(d,p)\C28H25N1O2S1\SHIMGRP\04-Dec-
 2021\0\\#p scrf=(iefpcm,smd,solvent=acetonitrile) b3lyp/6-311+G(d,p)\\
 YL-freq\\0,1\C,0,-2.068524,3.786283,0.624296\C,0,-0.762822,3.855009,0.
 132837\C,0,0.16732,2.872569,0.469294\C,0,-0.188804,1.799978,1.305598\C
 ,0,-1.514623,1.722612,1.762232\C,0,-2.440373,2.712946,1.435976\H,0,-2.
 790307,4.558672,0.371339\H,0,-0.466946,4.676122,-0.515339\H,0,1.177588
 ,2.920314,0.07379\H,0,-1.815444,0.892124,2.39403\H,0,-3.454775,2.64690
 8,1.821594\C,0,0.837106,0.823547,1.765384\C,0,0.457745,-0.614601,1.964
```

343\C,0,0.124859,-1.038681,3.261068\C,0,0.471078,-1.57531,0.930607\C,0
,-0.201359,-2.36527,3.538131\H,0,0.131934,-0.303511,4.060863\C,0,0.174
405,-2.91615,1.221084\C,0,-0.175982,-3.308022,2.511935\H,0,-0.459002,-
2.660117,4.551821\H,0,0.214273,-3.646022,0.420368\H,0,-0.411746,-4.349
494,2.713236\C,0,2.066574,1.22924,2.114748\H,0,2.818082,0.528119,2.462
256\H,0,2.361323,2.272849,2.055467\N,0,0.878351,-1.182156,-0.405442\C,
0,2.308655,-1.491009,-0.751842\H,0,2.679013,-2.224324,-0.028949\H,0,2.
329956,-1.977913,-1.732109\C,0,3.223106,-0.284172,-0.785994\C,0,4.4122
52,-0.288229,-0.050046\C,0,2.938127,0.815595,-1.606863\C,0,5.299709,0.
788859,-0.117434\H,0,4.648203,-1.141353,0.583126\C,0,3.820382,1.893303
,-1.672119\H,0,2.026521,0.8123,-2.196148\C,0,5.003144,1.885311,-0.9262
94\H,0,6.218456,0.76972,0.46318\H,0,3.590545,2.738754,-2.315978\H,0,5.
690177,2.725728,-0.981754\S,0,-0.169555,-1.617203,-1.705986\O,0,0.3886
62,-0.929171,-2.877889\O,0,-0.371963,-3.074914,-1.772774\C,0,-1.740455
,-0.869129,-1.296375\C,0,-2.001778,0.431964,-1.732043\C,0,-2.727111,-1
.633198,-0.672874\C,0,-3.266945,0.970684,-1.524445\H,0,-1.229991,1.000
137,-2.238153\C,0,-3.985435,-1.070672,-0.464125\H,0,-2.517652,-2.65466
,-0.377061\C,0,-4.275297,0.233095,-0.885594\H,0,-3.474837,1.982138,-1.
862611\H,0,-4.758498,-1.661555,0.020603\C,0,-5.637565,0.841912,-0.6554
54\H,0,-6.356219,0.098062,-0.298505\H,0,-5.587182,1.643602,0.09267\H,0
,-6.036979,1.285645,-1.574735\\Version=ES64L-G16RevA.03\State=1-A\HF=-
1685.7243107\RMSD=1.224e-09\Dipole=-1.3117081,1.0382005,2.1689965\Quad
rupole=17.0078131,-1.6619372,-15.3458759,-2.8060782,2.3483826,-16.5859
383\PG=C01 [X(C28H25N1O2S1)]\\@

1a·+

1\1\GINC-OMC-1-811\SP\UB3LYP\6-311+G(d,p)\C28H25N1O2S1(1+,2)\SHIMGRP\2
3-Nov-2021\0\\#p b3lyp/6-311+G(d,p) scrf=(iefpcm,smd,solvent=acetonitr
ile)\\LQZ-TS1\\1,2\C,0,5.022707,-2.077492,0.119901\C,0,5.25478,-3.2543
47,0.836701\C,0,4.17569,-3.9872,1.343036\C,0,2.873955,-3.543126,1.1424
1\C,0,2.621524,-2.352095,0.429465\C,0,3.720283,-1.634735,-0.08996\C,0,
1.243394,-1.847441,0.253954\C,0,0.891819,-1.133752,-0.993654\C,0,0.273
072,-2.079859,1.180647\C,0,1.277256,-1.678341,-2.231188\C,0,0.800996,-
1.155926,-3.425693\C,0,-0.053094,-0.039591,-3.413441\C,0,-0.420557,0.5
54339,-2.217904\C,0,0.064427,0.042525,-0.992428\N,0,-0.208226,0.687102
,0.219597\C,0,0.89946,1.194994,1.10098\S,0,-1.820642,1.456641,0.537666
\C,0,1.323178,2.58334,0.674386\C,0,0.903644,3.71122,1.398606\C,0,1.335
477,4.98286,1.027113\C,0,2.173797,5.142891,-0.078944\C,0,2.587734,4.02
5204,-0.813056\C,0,2.159947,2.754662,-0.441817\O,0,-1.710854,1.831541,
1.943383\O,0,-2.071647,2.438044,-0.512892\C,0,-2.952378,0.096623,0.376
14\C,0,-3.202774,-0.706454,1.495856\C,0,-4.134137,-1.734108,1.386905\C
,0,-4.826919,-1.966343,0.186849\C,0,-4.561373,-1.133597,-0.91183\C,0,-
3.634306,-0.098643,-0.828925\C,0,-5.862869,-3.057253,0.094629\H,0,5.85
7368,-1.504223,-0.272017\H,0,6.270809,-3.602665,0.99629\H,0,4.352296,-
4.910441,1.886411\H,0,2.042931,-4.135363,1.512758\H,0,3.551597,-0.7155

1,-0.643216\H,0,-0.760422,-1.807911,1.006944\H,0,0.497551,-2.590397,2.
111716\H,0,1.913171,-2.556979,-2.238701\H,0,1.08128,-1.616622,-4.36783
3\H,0,-0.399468,0.388493,-4.349228\H,0,-1.010111,1.461881,-2.215346\H,
0,0.535348,1.181015,2.126856\H,0,1.718173,0.481514,1.014747\H,0,0.2446
81,3.58746,2.252107\H,0,1.017286,5.848625,1.599817\H,0,2.503758,6.1353
52,-0.371561\H,0,3.245246,4.148694,-1.668456\H,0,2.488144,1.889151,-1.
013086\H,0,-2.698672,-0.509605,2.436187\H,0,-4.340395,-2.357045,2.2528
48\H,0,-5.099343,-1.288896,-1.842826\H,0,-3.462304,0.55937,-1.672857\H
,0,-5.629279,-3.891616,0.762715\H,0,-6.849598,-2.673345,0.384637\H,0,-
5.950591,-3.443724,-0.9251\\Version=ES64L-G16RevA.03\State=2-A\HF=-168
5.517362\S2=0.761802\S2-1=0.\S2A=0.750098\RMSD=2.052e-09\Dipole=0.6002
97,-3.4886729,-1.1767134\Quadrupole=13.4625597,-6.6924137,-6.7701459,9
.1252655,-0.9079049,-6.6914338\PG=C01 [X(C28H25N1O2S1)]\\@

TS1

1\1\GINC-OMC-1-801\SP\UB3LYP\6-311+G(d,p)\C28H25N1O2S1(1+,2)\SHIMGRP\2
3-Nov-2021\0\\#p b3lyp/6-311+G(d,p) scrf=(iefpcm,smd,solvent=acetonitr
ile)\\Tilte Card Required\\1,2\C\C,1,1.39960322\C,2,1.40047154,1,120.0
3663439\C,3,1.38705101,2,120.07884049,1,0.0123483,0\C,4,1.41884812,3,1
20.84249689,2,-0.17811761,0\C,1,1.38829325,2,120.23820671,3,-0.0851685
5,0\C,5,1.44815785,4,120.12224872,3,-179.73118389,0\C,7,1.46437628,5,1
24.42956725,4,-165.89685623,0\C,7,1.41080159,5,123.33570972,4,25.75950
864,0\C,8,1.4017232,7,127.28007022,5,41.070046,0\C,10,1.39369726,8,119
.72182123,7,-177.68363516,0\C,11,1.39766923,10,120.25363448,8,1.790894
49,0\C,12,1.39982694,11,121.05629676,10,-0.84739851,0\C,13,1.3930719,1
2,118.69316505,11,-1.35038744,0\N,14,1.45289793,13,124.7589509,12,178.
50476093,0\C,15,1.52801336,14,115.75641591,13,-86.45009403,0\S,15,1.83
933507,14,117.43128534,13,48.46865846,0\C,16,1.50784777,15,115.9772388
6,14,63.68278098,0\C,18,1.40278653,16,120.48301461,15,92.76721891,0\C,
19,1.39385802,18,120.32221656,16,178.05868942,0\C,20,1.39657187,19,120
.12685087,18,-0.13602399,0\C,21,1.39660311,20,119.97187129,19,-0.34322
374,0\C,22,1.3943304,21,119.92420535,20,0.33516832,0\O,17,1.46042429,1
5,101.95095073,14,178.54690653,0\O,17,1.45972136,15,106.84027009,14,-5
1.82564614,0\C,17,1.77876701,15,105.29399498,14,64.04022791,0\C,26,1.4
0049089,17,118.71177984,15,98.70086703,0\C,27,1.39164004,26,118.717867
03,17,176.03809017,0\C,28,1.40441288,27,121.28609417,26,-0.43666493,0\
C,29,1.40440705,28,118.50603053,27,-0.08029257,0\C,30,1.3914253,29,121
.37848696,28,-0.19846104,0\C,29,1.5072853,28,120.74268767,27,-178.2333
6776,0\H,1,1.08566127,6,119.73710666,5,-179.01319971,0\H,2,1.08583554,
1,119.99947118,6,179.88616439,0\H,3,1.08557526,2,120.07788808,1,179.35
500676,0\H,4,1.08583693,3,119.48328446,2,177.63227045,0\H,6,1.08442988
,1,119.58653549,2,178.03404414,0\H,9,1.08272789,7,119.63343832,5,-141.
17591012,0\H,9,1.08781159,7,121.18741239,5,16.19866958,0\H,10,1.085232
98,8,119.72959895,7,4.34299725,0\H,11,1.08556391,10,119.7717591,8,-178
.38093565,0\H,12,1.08574971,11,119.87217555,10,178.45505777,0\H,13,1.0

8232284,12,120.76632737,11,178.52548243,0\H,16,1.09035697,15,105.28179
682,14,-172.36776843,0\H,16,1.09347223,15,103.99262246,14,-58.29358698
,0\H,19,1.08624401,18,119.84607756,16,-1.51362189,0\H,20,1.08584611,19
,119.71669182,18,-179.71595173,0\H,21,1.08614336,20,120.02619406,19,-1
79.70401902,0\H,22,1.08614442,21,120.17107215,20,-178.94725925,0\H,23,
1.08752477,22,119.65744485,21,-178.76466886,0\H,27,1.08488211,26,120.2
9121184,17,-2.51581499,0\H,28,1.08643959,27,119.17943636,26,-179.49784
972,0\H,30,1.08641337,29,119.4720524,28,-179.22374577,0\H,31,1.0838891
7,30,120.64962832,29,-177.85770254,0\H,32,1.0940928,29,111.57681794,28
,-150.99859604,0\H,32,1.09399919,29,111.58891586,28,-29.45947052,0\H,3
2,1.09774179,29,110.37676068,28,89.78425046,0\\Version=ES64L-G16RevA.0
3\State=2-A\HF=-1685.5082936\S2=0.761314\S2-1=0.\S2A=0.750114\RMSD=3.7
85e-09\Dipole=-1.467687,3.0252723,1.6835278\Quadrupole=9.9083635,-12.4
691282,2.5607647,11.1507652,2.5331243,3.7474036\PG=C01 [X(C28H25N1O2S1
)]\\@

INT1

1\1\GINC-OMC-1-802\SP\UB3LYP\6-311+G(d,p)\C28H25N1O2S1(1+,2)\SHIMGRP\2
3-Nov-2021\0\\#p b3lyp/6-311+G(d,p) scrf=(iefpcm,smd,solvent=acetonitr
ile)\\int2\\1,2\C,0,0.242698,2.22692,3.087886\C,0,1.471574,1.565997,2.
936815\C,0,1.798345,0.932165,1.732746\C,0,0.864862,0.982192,0.705688\C
,0,-0.414903,1.586007,0.847572\C,0,-0.699414,2.243676,2.066536\N,0,0.9
94522,0.380625,-0.612047\C,0,-0.245055,0.81875,-1.376251\C,0,-1.169699
,1.42222,-0.351671\S,0,0.948722,-1.673638,-0.454226\C,0,-2.536673,1.73
7531,-0.663615\C,0,-2.956291,1.813971,-2.017562\C,0,-4.269176,2.12422,
-2.347406\C,0,-5.212962,2.356269,-1.341571\C,0,-4.828309,2.26291,0.000
39\C,0,-3.516573,1.957198,0.338548\C,0,2.246895,0.739628,-1.462896\O,0
,1.173823,-2.058427,-1.846752\O,0,1.887616,-1.927368,0.629808\C,0,-0.6
99152,-2.057169,0.047783\C,0,-1.016599,-2.019626,1.41236\C,0,-2.303606
,-2.375337,1.798681\C,0,-3.267813,-2.771462,0.856147\C,0,-2.909899,-2.
803008,-0.502938\C,0,-1.632947,-2.450265,-0.921681\C,0,-4.645976,-3.19
2297,1.294477\C,0,3.623885,0.475491,-0.918149\C,0,4.318748,1.506097,-0
.265136\C,0,5.628082,1.311869,0.171621\C,0,6.26195,0.087433,-0.051155\
C,0,5.58763,-0.935153,-0.721311\C,0,4.278397,-0.741543,-1.159404\H,0,0
.023331,2.738052,4.020494\H,0,2.184568,1.552396,3.754578\H,0,2.742197,
0.41893,1.616663\H,0,-1.627776,2.785276,2.196603\H,0,0.079728,1.54716,
-2.128906\H,0,-0.663907,-0.032873,-1.918123\H,0,-2.238407,1.659327,-2.
818297\H,0,-4.558748,2.192231,-3.391881\H,0,-6.238533,2.600946,-1.6005
98\H,0,-5.560399,2.421253,0.786931\H,0,-3.255543,1.839545,1.383349\H,0
,2.081797,0.203693,-2.399865\H,0,2.11848,1.809797,-1.644562\H,0,-0.270
944,-1.73922,2.147654\H,0,-2.562719,-2.357461,2.853369\H,0,-3.640122,-
3.120471,-1.241761\H,0,-1.35267,-2.508361,-1.967906\H,0,-4.922675,-2.7
35549,2.248909\H,0,-5.402162,-2.927584,0.549245\H,0,-4.687378,-4.28131
6,1.427554\H,0,3.835552,2.467032,-0.10531\H,0,6.156345,2.118228,0.6715
65\H,0,7.284353,-0.063545,0.282646\H,0,6.084389,-1.881177,-0.913992\H,

0,3.77041,-1.534217,-1.699901\\Version=ES64L-G16RevA.03\State=2-A\HF=-1685.5395715\S2=0.77838\S2-1=0.\S2A=0.750722\RMSD=2.012e-09\Dipole=-1.6667847,0.7581056,0.0789834\Quadrupole=16.2419609,-12.5451055,-3.6968554,12.6479931,0.4438761,0.290466\PG=C01 [X(C28H25N1O2S1)]\\@

INT2

1\1\GINC-OMC-1-801\SP\RB3LYP\6-311+G(d,p)\C21H18N1(1+)\SHIMGRP\23-Nov-2021\0\\#p b3lyp/6-311+G(d,p) scrf=(iefpcm,smd,solvent=acetonitrile)\\int3\\1,1\C,0,-2.023586,3.5796,0.655429\C,0,-0.735148,4.085346,0.288175\C,0,0.280941,3.277331,-0.165711\C,0,0.012345,1.890654,-0.280335\C,0,-1.304356,1.363438,0.035681\C,0,-2.310858,2.247525,0.538415\N,0,0.832766,0.8976,-0.653336\C,0,0.111046,-0.365232,-0.628929\C,0,-1.280755,-0.011824,-0.181164\C,0,-2.335036,-0.997291,-0.064523\C,0,-2.021814,-2.344071,0.23269\C,0,-3.02711,-3.295715,0.346712\C,0,-4.362611,-2.932024,0.14542\C,0,-4.688714,-1.610269,-0.173795\C,0,-3.689774,-0.649851,-0.274963\C,0,2.242367,0.993655,-1.07217\C,0,3.123694,-0.035084,-0.393643\C,0,3.702823,-1.067762,-1.13909\C,0,4.524269,-2.011833,-0.518512\C,0,4.766212,-1.931423,0.852824\C,0,4.189684,-0.902953,1.604247\C,0,3.374355,0.042154,0.984195\H,0,-2.767184,4.268826,1.040914\H,0,-0.551349,5.151345,0.387678\H,0,1.24828,3.692263,-0.423237\H,0,-3.273776,1.858426,0.846824\H,0,0.614727,-1.074805,0.039407\H,0,0.096984,-0.819682,-1.629145\H,0,-0.991995,-2.637219,0.412921\H,0,-2.772981,-4.321417,0.594901\H,0,-5.146257,-3.678967,0.22887\H,0,-5.722331,-1.332992,-0.355733\H,0,-3.947984,0.358721,-0.577735\H,0,2.289886,0.878304,-2.161784\H,0,2.575075,2.007321,-0.836071\H,0,3.524263,-1.128406,-2.2104\H,0,4.976054,-2.804347,-1.107647\H,0,5.407395,-2.662744,1.335931\H,0,4.384724,-0.83207,2.67029\H,0,2.939276,0.848025,1.571652\\Version=ES64L-G16RevA.03\State=1-A\HF=-865.9055679\RMSD=9.644e-09\Dipole=-0.6892637,1.0439994,-0.5979358\Quadrupole=6.496685,9.4668289,-15.9635139,0.1329181,-2.5008401,0.6659563\PG=C01 [X(C21H18N1)]\\@

TS2

1\1\GINC-OMC-1-822\SP\RB3LYP\6-311+G(d,p)\C28H25N1O2S1\SHIMGRP\19-Feb-2022\0\\#p b3lyp/6-311+G(d,p) scrf=(iefpcm,smd,solvent=acetonitrile)\\int2\\0,1\C,0,4.601063,2.343794,0.04249\C,0,4.84832,0.962124,-0.205663\C,0,3.838201,0.051995,-0.420997\C,0,2.50031,0.530149,-0.418422\C,0,2.239816,1.937618,-0.21024\C,0,3.318228,2.829483,0.049066\N,0,1.326217,-0.118601,-0.580414\C,0,0.241137,0.817965,-0.42669\C,0,0.853151,2.132241,-0.274993\S,0,-1.794761,-1.068382,2.044431\C,0,0.098713,3.379017,-0.172813\C,0,-1.163941,3.398355,0.45467\C,0,-1.880787,4.588794,0.548768\C,0,-1.368382,5.771126,0.009631\C,0,-0.125193,5.759958,-0.627291\C,0,0.604667,4.577782,-0.715642\C,0,1.015882,-1.5315,-0.886476\O,0,-1.333842,0.402322,1.760797\O,0,-0.736672,-2.08428,1.633551\C,0,-3.087666,-1.297669,0.760025\C,0,-3.27038,-2.558497,0.191145\C,0,-4.293848,-2.758934,-0.735797\C,0,-5.154912,-1.715192,-1.101539\C,0,-4.954973,-0.455906,-0.517877\C,0,-3.935366,-0.245444,0.410435\C,0,-6.288475,-1.945351,-2.0

74353\C,0,2.076583,-2.545064,-0.521111\C,0,2.869195,-3.13986,-1.510758
\C,0,3.837314,-4.087321,-1.171866\C,0,4.016502,-4.450203,0.164292\C,0,
3.213798,-3.876096,1.153636\C,0,2.242709,-2.933206,0.816232\H,0,5.4387
25,3.005643,0.240134\H,0,5.876314,0.609503,-0.210837\H,0,4.061423,-0.9
91718,-0.586453\H,0,3.115986,3.872639,0.266336\H,0,-0.511181,0.727519,
-1.21808\H,0,-0.400595,0.554809,0.558364\H,0,-1.553512,2.489653,0.9052
26\H,0,-2.844392,4.592786,1.050794\H,0,-1.935377,6.695266,0.08174\H,0,
0.273356,6.671984,-1.063283\H,0,1.55152,4.569092,-1.246232\H,0,0.11035
5,-1.761244,-0.309758\H,0,0.786598,-1.594983,-1.958486\H,0,-2.594327,-
3.364284,0.461773\H,0,-4.424955,-3.741014,-1.186203\H,0,-5.605183,0.37
0885,-0.797866\H,0,-3.783084,0.735079,0.852149\H,0,-6.537843,-1.034009
,-2.6295\H,0,-6.039444,-2.726386,-2.800535\H,0,-7.200779,-2.265489,-1.
553155\H,0,2.73074,-2.856706,-2.552341\H,0,4.445815,-4.542405,-1.94900
5\H,0,4.768934,-5.187557,0.431816\H,0,3.335292,-4.173364,2.191861\H,0,
1.582377,-2.516666,1.571581\\Version=ES64L-G16RevA.03\State=1-A\HF=-16
85.7536664\RMSD=6.686e-09\Dipole=1.7175232,1.9972122,-3.2436771\Quadru
pole=15.5554119,4.3722891,-19.9277009,-1.5046153,5.4953449,5.1807557\P
G=C01 [X(C28H25N1O2S1)]\\@

TsH

1\1\GINC-OMC-1-820\SP\RB3LYP\6-311+G(d,p)\C7H8O2S1\SHIMGRP\16-Feb-2022
\0\\#p b3lyp/6-311+G(d,p) geom=check scrf=(iefpcm,smd,solvent=acetonit
rile)\\int1\\0,1\S,0,-2.1413670203,0.197987653,0.5001718518\O,0,-2.612
1906049,-1.1879091228,0.2082148399\O,0,-2.4889086683,1.2292882046,-0.7
88786527\C,0,-0.3394719758,0.1492021191,0.2526934335\C,0,0.2906682991,
-1.0850679717,0.1137680073\C,0,1.678148667,-1.1296756002,-0.029053956\
C,0,2.4456775866,0.0420819645,-0.0192626228\C,0,1.7854430041,1.2728296
613,0.1284080838\C,0,0.4017163726,1.3343789375,0.2688310291\C,0,3.9502
286004,-0.0125906034,-0.139601739\H,0,-0.3103564161,-1.9894564995,0.10
70400364\H,0,2.1727348181,-2.0908103851,-0.1483237348\H,0,2.3647843433
,2.1933030476,0.1301426414\H,0,-0.0990760921,2.2935210042,0.3696268523
\H,0,4.2833263781,-0.958313899,-0.5783068973\H,0,4.3328250818,0.804350
1725,-0.7612095364\H,0,4.4280777475,0.0793587553,0.8446778895\H,0,-2.1
437929311,0.8242677921,-1.6100445217\\Version=ES64L-G16RevA.03\State=1
-A\HF=-820.3161228\RMSD=3.864e-09\Dipole=2.3486104,0.6567008,-0.331131
8\Quadrupole=-2.4554964,0.8809517,1.5745448,-2.9817253,0.3578745,0.748
5925\PG=C01 [X(C7H8O2S1)]\\@

2a

1\1\GINC-OMC-1-820\SP\RB3LYP\6-311+G(d,p)\C21H17N1\SHIMGRP\10-Dec-2021
\0\\#p scrf=(iefpcm,smd,solvent=acetonitrile) b3lyp/6-311+G(d,p)\\CW-D
J-Freq\\0,1\N,0,0.679017,-0.119825,-1.003563\C,0,0.447251,1.162762,-0.
533932\C,0,1.313604,2.259653,-0.462863\C,0,0.797667,3.454708,0.024405\
C,0,-0.549494,3.55647,0.425463\C,0,-1.403632,2.462827,0.359158\C,0,-0.
909387,1.233973,-0.113029\C,0,-1.495223,-0.070857,-0.341342\C,0,-0.491

581,-0.843634,-0.882271\C,0,-2.870143,-0.516531,-0.067237\C,0,-3.56584
9,-0.077476,1.073912\C,0,-4.862715,-0.517018,1.336802\C,0,-5.493821,-1
.413,0.47206\C,0,-4.814542,-1.862377,-0.662401\C,0,-3.52293,-1.414133,
-0.931928\C,0,1.939984,-0.626996,-1.515785\C,0,3.021283,-0.838241,-0.4
65064\C,0,2.705806,-1.261564,0.830989\C,0,3.717094,-1.495459,1.763019\
C,0,5.055914,-1.312428,1.410617\C,0,5.377963,-0.888816,0.1206\C,0,4.36
4605,-0.649826,-0.808983\H,0,2.352785,2.18037,-0.767434\H,0,1.444425,4
.324955,0.09538\H,0,-0.925175,4.508334,0.791012\H,0,-2.443237,2.559732
,0.657027\H,0,-0.513042,-1.885126,-1.174816\H,0,-3.073092,0.592301,1.7
72222\H,0,-5.377954,-0.16512,2.22709\H,0,-6.504316,-1.755493,0.678193\
H,0,-5.297157,-2.554457,-1.348027\H,0,-3.014858,-1.74289,-1.834861\H,0
,2.308856,0.056054,-2.29125\H,0,1.715751,-1.574846,-2.019715\H,0,1.665
425,-1.397068,1.113432\H,0,3.457938,-1.818975,2.767631\H,0,5.842165,-1
.49332,2.138524\H,0,6.416364,-0.735471,-0.161099\H,0,4.620866,-0.31241
9,-1.811502\\Version=ES64L-G16RevA.03\State=1-A\HF=-865.4799819\RMSD=6
.087e-09\Dipole=1.0585731,-0.8204678,-0.6137553\Quadrupole=5.2071772,-
4.9027439,-0.3044333,2.1045142,-5.3107375,4.0031686\PG=C01 [X(C21H17N1
)]\\@

PC·

1\1\GINC-B2167\SP\UB3LYP\6-311+G(d,p)\C36H40B1F4N1(1-,2)\ROOT\20-Jan-2
022\0\\#p geom=check scrf=(iefpcm,smd,solvent=acetonitrile) b3lyp/6-31
1+G(d,p)\\Title Card Required\\-1,2\C,0,-1.78269258,3.6458657207,0.114
7452203\C,0,-0.6654490866,2.8368928125,-0.1485033365\C,0,-0.7471291682
,1.4416837349,-0.2297875745\C,0,-2.0024724215,0.789402775,-0.003573129
6\C,0,-3.1188476423,1.6217636121,0.251742571\C,0,-3.0168903353,3.00613
74924,0.3054016602\C,0,-2.0832175492,-0.6339548472,-0.0336779516\C,0,-
0.8921846592,-1.3950916349,-0.2491107535\C,0,0.3521271006,-0.737878989
,-0.4897633265\C,0,1.5194979004,-1.4874608007,-0.6908706888\H,0,2.4523
634205,-0.9660133263,-0.8370251325\C,0,1.521200862,-2.8848946315,-0.65
8003711\C,0,0.2915252579,-3.5318211299,-0.423521364\C,0,-0.8739220893,
-2.8113511915,-0.2300460993\H,0,0.3028585846,3.298675592,-0.2907996652
\H,0,-4.0826380095,1.1492932014,0.4148596053\H,0,-3.9138454973,3.58254
41473,0.5053954994\H,0,0.2455989881,-4.6157886491,-0.3761329654\H,0,-1
.8078088777,-3.334817692,-0.0483063374\N,0,0.3830840661,0.6695725687,-
0.5262108811\C,0,1.5996475297,1.3425493729,-0.9177909792\C,0,1.6791938
118,1.8950514145,-2.2021376902\C,0,2.679722502,1.4336280116,-0.0395685
635\C,0,2.8421905874,2.5526047306,-2.6000693136\H,0,0.8293889086,1.807
3701359,-2.8734705372\C,0,3.8529609543,2.0713125872,-0.4536671212\H,0,
2.6503929886,0.9636706355,0.9371697589\C,0,3.9298986732,2.6389084565,-
1.7252756622\H,0,2.9034727182,2.9828903449,-3.5971838497\H,0,4.6910609
295,2.0764508051,0.2331182307\H,0,4.843527463,3.1350127725,-2.04436311
26\C,0,2.8233735561,-3.6928363819,-0.7924919533\C,0,3.9868368937,-2.84
84219442,-1.3527695412\C,0,3.2249701878,-4.1940804282,0.617352833\C,0,
2.6124100638,-4.9002851869,-1.7355529303\H,0,3.7261548239,-2.400272254

6,-2.320472531\H,0,4.3016522021,-2.0569856177,-0.666989169\H,0,4.85709
61825,-3.497272615,-1.513054322\H,0,2.4404871552,-4.8249434889,1.05443
08417\H,0,4.1462891344,-4.7893015052,0.5602213116\H,0,3.4045376516,-3.
3414595542,1.2786372296\H,0,3.5488952527,-5.4635705864,-1.8328728904\H
,0,1.8514230878,-5.5967630262,-1.3661900316\H,0,2.3096880728,-4.572610
7306,-2.7380575781\C,0,-1.6045241628,5.1725796133,0.1859881801\C,0,-0.
6115902721,5.5281437987,1.3186310856\C,0,-1.0479209812,5.6958480437,-1
.1601019086\C,0,-2.931418435,5.9027735591,0.4684356677\H,0,-0.98676580
43,5.1807430215,2.2881173184\H,0,0.3683226567,5.0678181127,1.158906917
8\H,0,-0.4670831771,6.6151049489,1.3772870044\H,0,-1.7415814036,5.4750
03039,-1.9801026375\H,0,-0.9018378867,6.7834259086,-1.120087547\H,0,-0
.0851290857,5.2371570354,-1.4063799476\H,0,-2.7583769337,6.9850270736,
0.5079713451\H,0,-3.6746804389,5.7133307308,-0.3148134892\H,0,-3.36461
31701,5.6007615494,1.4288688782\C,0,-3.3918473068,-1.320930355,0.19591
42525\C,0,-4.2288971703,-1.6394368718,-0.8950013145\C,0,-3.7991716328,
-1.6620544784,1.5043902474\C,0,-5.4485551721,-2.284502533,-0.662387727
9\C,0,-5.0261337154,-2.3069140517,1.6954841387\C,0,-5.8682344871,-2.62
4278958,0.6262946645\H,0,-6.0850374285,-2.5292290639,-1.5117810556\H,0
,-5.3289477801,-2.5702410124,2.7080335672\C,0,-2.9194534396,-1.3420542
784,2.6915688482\H,0,-1.9361081648,-1.8169847254,2.5996576657\H,0,-2.7
407446412,-0.2638114733,2.7761204866\H,0,-3.3790569525,-1.6856586223,3
.624389363\C,0,-3.8133760922,-1.294245773,-2.3073065067\H,0,-3.6555737
458,-0.2155959958,-2.4236755667\H,0,-2.868568544,-1.7817590859,-2.5744
048544\H,0,-4.575736959,-1.6077433756,-3.0287318926\C,0,-7.2044869211,
-3.292083349,0.8599183486\H,0,-7.9982898977,-2.5535243079,1.0399791356
\H,0,-7.5107311547,-3.8905285218,-0.0055552738\H,0,-7.1755870692,-3.95
28654933,1.7338053728\B,0,4.9904288763,-0.4530684172,1.8287941202\F,0,
5.7794664735,-1.0919471837,2.7816410246\F,0,5.5958938487,-0.5129112199
,0.5558057884\F,0,4.7972622279,0.9056487979,2.1814909072\F,0,3.7141553
479,-1.0762157983,1.750154325\\Version=ES64L-G16RevA.03\State=2-A\HF=-
1875.8595775\S2=0.769385\S2-1=0.\S2A=0.75032\RMSD=4.791e-09\Dipole=-7.
8513611,1.2452701,-3.3196979\Quadrupole=-45.9727702,29.8487193,16.1240
509,12.6136744,-32.2598247,-1.1653238\PG=C01 [X(C36H40B1F4N1)]\\@

PC

1\1\GINC-B2074\SP\RB3LYP\6-311+G(d,p)\C36H40B1F4N1\ROOT\19-Jan-2022\0\
\#p geom=check scrf=(iefpcm,smd,solvent=acetonitrile) b3lyp/6-311+G(d,
p)\\Title Card Required\\0,1\C,0,1.8972236085,2.8717488699,-0.80488218
5\C,0,2.0153084859,1.4969741238,-0.8438661405\C,0,0.8795037591,0.66329
03607,-0.7890772431\C,0,-0.4293246782,1.2447999313,-0.7195221881\C,0,-
0.5277133705,2.6665746094,-0.8056939716\C,0,0.5901354081,3.4463901181,
-0.8584145298\C,0,-1.550640028,0.4282966286,-0.5127911277\C,0,-1.37520
3397,-0.9746274534,-0.4450795801\C,0,-0.0684910955,-1.5392936778,-0.57
13582245\C,0,0.0962179686,-2.940119109,-0.5007083361\H,0,1.0945911343,
-3.344758489,-0.5897131665\C,0,-0.9767363505,-3.794455727,-0.298329048

4\C,0,-2.273267603,-3.2252869125,-0.1691257263\C,0,-2.4618973513,-1.86
72115603,-0.2363895862\H,0,2.9894012086,1.0378349714,-0.808642269\H,0,
-1.5149400014,3.1139945228,-0.7907897327\H,0,0.4817552622,4.5252680167
,-0.8853058641\H,0,-3.132643346,-3.8642073876,-0.0073130649\H,0,-3.455
208702,-1.446645205,-0.1258933644\N,0,1.0121139478,-0.7056087243,-0.76
13034549\C,0,2.3440694627,-1.2911570014,-0.8100819965\C,0,2.8591669091
,-1.7059806386,-2.0386829265\C,0,3.0646201705,-1.4287128078,0.37646867
15\C,0,4.129146251,-2.2835442377,-2.0795837802\H,0,2.2731249355,-1.578
9315382,-2.9444201152\C,0,4.3344974269,-2.0058469553,0.3177426161\H,0,
2.6388000588,-1.0533836515,1.2996028598\C,0,4.8645440655,-2.4348721389
,-0.9013742092\H,0,4.5416520881,-2.6109189122,-3.0296418445\H,0,4.9114
449236,-2.1100225931,1.2318456428\H,0,5.8541988104,-2.8820475942,-0.93
57207971\C,0,-0.7296066645,-5.3114468593,-0.2081586798\C,0,-0.06548949
35,-5.8026804713,-1.5171342799\C,0,0.2098411832,-5.6019541461,0.987680
6676\C,0,-2.0331227295,-6.1069195639,-0.0023817081\H,0,-0.710727314,-5
.6125428355,-2.3825387065\H,0,0.8954742683,-5.3108429384,-1.6992526429
\H,0,0.118335526,-6.8820479868,-1.4622139676\H,0,-0.2347042948,-5.2620
657407,1.929449724\H,0,0.393634113,-6.6799600805,1.0668354922\H,0,1.17
8562904,-5.1043423983,0.8777269044\H,0,-1.8028400918,-7.1764554525,0.0
494144901\H,0,-2.5366542624,-5.8347927375,0.9319468197\H,0,-2.73789418
88,-5.9622375014,-0.8292797608\C,0,3.1115959775,3.7862710894,-0.621360
418\C,0,2.881825714,4.6044855778,0.6772230333\C,0,4.4169671251,2.98777
39104,-0.4489731521\C,0,3.2483938347,4.735287315,-1.8341411535\H,0,2.0
44926812,5.3041443512,0.577846025\H,0,2.6649406166,3.9344233268,1.5132
703524\H,0,3.7795693936,5.1911935722,0.905431243\H,0,4.6585147106,2.39
35676297,-1.3390493162\H,0,5.2487208959,3.6816926532,-0.2850351547\H,0
,4.3498626929,2.3229194579,0.4178454893\H,0,4.0881188914,5.4221764358,
-1.6763089907\H,0,3.4388279721,4.1752890758,-2.7575962405\H,0,2.349498
2559,5.3428410823,-1.987532038\C,0,-2.9025861141,1.0326100066,-0.30546
96638\C,0,-3.7850130931,1.1971342368,-1.3921514095\C,0,-3.2720755961,1
.4320907102,0.9988108003\C,0,-5.0404367549,1.7659081432,-1.156664512\C
,0,-4.5419094354,1.9913882265,1.1792256111\C,0,-5.4371010039,2.1719129
203,0.1207709394\H,0,-5.7237349577,1.8937427562,-1.9938799239\H,0,-4.8
357168895,2.2953148906,2.1818032998\C,0,-2.33422539,1.2717461682,2.173
1878773\H,0,-1.9366678782,0.2544784227,2.2530712116\H,0,-1.4523207152,
1.9183383844,2.0992116238\H,0,-2.8503297889,1.5089618395,3.1081840792\
C,0,-3.3950783781,0.774937829,-2.7920388315\H,0,-2.4829456252,1.282327
3204,-3.12918371\H,0,-3.2015627677,-0.3031696905,-2.8538475015\H,0,-4.
1903234046,1.0108870492,-3.5051789102\C,0,-6.7868286158,2.8116591296,0
.3485747134\H,0,-6.7161543621,3.9070913847,0.3166673662\H,0,-7.5091619
339,2.5094630925,-0.4169595897\H,0,-7.1968723033,2.5436022024,1.328173
2471\B,0,1.4869976106,1.1427832977,2.5858188463\F,0,1.4982609272,1.095
1206992,3.9655968856\F,0,2.8069668514,1.1440497934,2.0666279227\F,0,0.
8175495759,2.2984721743,2.1177781968\F,0,0.8148662331,-0.0043505897,2.
0562300829\\Version=ES64L-G16RevA.03\State=1-A\HF=-1875.7273153\RMSD=3

.200e-09\Dipole=-1.837792,-2.5039071,-5.1920543\Quadrupole=13.6230767,
14.8551365,-28.4782132,-8.560363,-14.0109051,-7.4795307\PG=C01 [X(C36H
40B1F4N1)]\\@

1n·+

1\1\GINC-OMC-1-810\SP\UB3LYP\6-311+G(d,p)\C29H24F3N1O2S1(1+,2)\SHIMGRP
\16-Feb-2022\0\\#p b3lyp/6-311+G(d,p) geom=check scrf=(iefpcm,smd,solv
ent=acetonitrile)\\int1-pcf3\\1,2\C,0,-4.4592512807,0.1260518725,0.324
0979318\C,0,-5.183167683,-0.8920373175,-0.3008413396\C,0,-4.5160655618
,-1.9995890298,-0.8336283308\C,0,-3.1317345049,-2.0854832049,-0.745682
598\C,0,-2.3836921244,-1.0656141807,-0.1266657373\C,0,-3.0743249394,0.
0350968075,0.4167842069\C,0,-0.9024163486,-1.1295715429,-0.0750094669\
C,0,-0.2077647559,-0.607248239,1.1254055351\C,0,-0.1817354364,-1.71674
7868,-1.0633113207\C,0,-0.6773196663,-0.9707162106,2.3978461106\C,0,0.
0493163393,-0.6820071401,3.5462537283\C,0,1.2664451535,0.0156048421,3.
4525695684\C,0,1.7450444788,0.4298455948,2.2229425351\C,0,1.008260608,
0.1602937442,1.0428270112\N,0,1.4266966438,0.6698157196,-0.1860444632\
C,0,0.5332844818,1.5247264264,-1.0371958979\S,0,3.1985217521,0.7694032
658,-0.6282956451\C,0,0.7000459486,2.9790651139,-0.6521627483\C,0,1.43
25146045,3.8544848585,-1.4711147225\C,0,1.5344085434,5.2031240588,-1.1
377037964\C,0,0.926023924,5.6858427965,0.0242491332\C,0,0.2062400653,4
.8171299236,0.85347426\C,0,0.1005973544,3.4710347079,0.5208953518\O,0,
3.1367421014,1.1531016631,-2.0336266632\O,0,3.863170755,1.5884005566,0
.3789256068\C,0,3.7343232001,-0.9173309317,-0.4961847562\C,0,3.5663857
946,-1.7643421991,-1.599243247\C,0,4.0410746702,-3.0692287157,-1.51751
42193\C,0,4.6895450759,-3.5386674783,-0.3626463805\C,0,4.8532171235,-2
.6581349763,0.7188677984\C,0,4.3867929711,-1.3480796388,0.6634612309\C
,0,5.2351725738,-4.9421246579,-0.3030492985\H,0,-4.9778837063,0.989122
3549,0.7272523829\H,0,-5.0814112144,-2.7918340755,-1.3125078274\H,0,-2
.6276804957,-2.9629244887,-1.13815649\H,0,-2.5235188771,0.8363438093,0
.9004663241\H,0,0.8898458504,-1.8552675133,-0.9859518702\H,0,-0.664164
558,-2.1071946618,-1.9537303532\H,0,-1.5994644175,-1.5372832459,2.4702
810011\H,0,-0.3193671107,-1.0057758403,4.5146140125\H,0,1.818755828,0.
2673622307,4.3527072744\H,0,2.6361647586,1.0407351084,2.1600662147\H,0
,0.7964111327,1.3494321681,-2.0789163607\H,0,-0.4859974051,1.178180099
2,-0.8739385343\H,0,1.9128470271,3.4768376996,-2.3679765089\H,0,2.0886
86294,5.8775000512,-1.7831778609\H,0,1.0137907083,6.7359255452,0.28691
45299\H,0,-0.2713937011,5.1938799108,1.7529016567\H,0,-0.4610064889,2.
80039076,1.1673554109\H,0,3.1008881396,-1.3978266299,-2.508113436\H,0,
3.9222940451,-3.7311186297,-2.3708104684\H,0,5.3667342635,-2.999497904
4,1.613265335\H,0,4.5489355736,-0.6672738872,1.491125984\H,0,4.6455710
292,-5.629601373,-0.9168161541\H,0,6.2651997307,-4.9678540007,-0.68194
3911\H,0,5.2556806552,-5.3228126331,0.7223438031\C,0,-6.6921038789,-0.
8279326348,-0.3452277242\F,0,-7.2297653658,-1.4234819863,0.739748168\F
,0,-7.1296366041,0.4465097447,-0.3657469358\F,0,-7.1786850739,-1.45446

45977,-1.4337770397\\Version=ES64L-G16RevA.03\State=2-A\HF=-2022.66504
45\S2=0.762014\S2-1=0.\S2A=0.750112\RMSD=3.764e-09\Dipole=2.3700569,-2
.5205852,1.6295956\Quadrupole=-19.3999668,14.1912665,5.2087003,-21.454
1111,1.7490241,6.6713878\PG=C01 [X(C29H24F3N1O2S1)]\\@

TS1-1n

1\1\GINC-OMC-1-812\SP\UB3LYP\6-311+G(d,p)\C29H24F3N1O2S1(1+,2)\SHIMGRP
\18-Feb-2022\0\\#p b3lyp/6-311+G(d,p) geom=check scrf=(iefpcm,smd,solv
ent=acetonitrile)\\Tilte Card Required\\1,2\C,0,-4.4353402307,-3.32655
77808,0.1034344196\C,0,-4.3677350293,-4.2717519668,-0.9262245531\C,0,-
3.1882484558,-4.4251695938,-1.6648356889\C,0,-2.0826870679,-3.64131713
35,-1.3723150211\C,0,-2.1265931788,-2.6775134169,-0.3347234785\C,0,-3.
3302569796,-2.5414860145,0.3997383177\C,0,-0.9610312175,-1.8608005875,
-0.0474818581\C,0,-0.7856996996,-1.0818247778,1.1783436058\C,0,0.01994
25057,-1.5525228482,-1.0095628615\C,0,-1.1133157912,-1.4746703698,2.48
40786055\C,0,-0.914943441,-0.5896251725,3.5415672016\C,0,-0.4278151198
,0.6984446116,3.2996528745\C,0,-0.1138776413,1.1209816967,2.0027569728
\C,0,-0.2705299196,0.2222571768,0.949630632\N,0,-0.0196387205,0.532938
1335,-0.4452007623\C,0,-1.1911770711,1.1393546185,-1.2163195742\S,0,1.
5664512568,1.3752913712,-0.8578618743\C,0,-1.6524447301,2.4955828009,-
0.746366099\C,0,-1.1409629956,3.663278438,-1.3322526716\C,0,-1.6001233
934,4.9143403723,-0.9239562006\C,0,-2.5751779328,5.0126521849,0.071062
1126\C,0,-3.0972689526,3.8554853421,0.6536848947\C,0,-2.6385937828,2.6
037774462,0.2457069659\O,0,1.4789994788,1.4848015575,-2.3111913691\O,0
,1.6603678801,2.5419032931,0.0140826232\C,0,2.8402735599,0.2150806207,
-0.41875439\C,0,3.4043037711,-0.5781297578,-1.4261413972\C,0,4.4577668
607,-1.426027059,-1.0981013814\C,0,4.9628911522,-1.489221548,0.2110036
722\C,0,4.3814871462,-0.6717744615,1.1939715938\C,0,3.3308052175,0.189
0114921,0.8919831072\C,0,6.1302617484,-2.3825831547,0.5437415243\H,0,-
5.358951033,-3.1980996609,0.6572843169\H,0,-3.1415486245,-5.1597076126
,-2.4613588196\H,0,-1.1627931101,-3.7944920582,-1.9278953549\H,0,-3.40
5860073,-1.7908532561,1.1784365644\H,0,1.0627767089,-1.5158621667,-0.7
215055008\H,0,-0.1693603172,-1.689712187,-2.0719816662\H,0,-1.48616493
09,-2.4772589412,2.6673927055\H,0,-1.1422477389,-0.9018987244,4.556036
5423\H,0,-0.3007797304,1.3919211491,4.1253706978\H,0,0.2383896378,2.12
76222334,1.8183654705\H,0,-0.8693113664,1.1620030238,-2.2577495451\H,0
,-1.9890003423,0.3987449315,-1.1141679754\H,0,-0.3891994023,3.59195566
47,-2.1130486634\H,0,-1.2015196469,5.811631473,-1.3875922316\H,0,-2.93
51275443,5.988207991,0.3846911513\H,0,-3.8683506153,3.9279408633,1.415
1350914\H,0,-3.0631561069,1.7052018249,0.6873922341\H,0,3.0389463199,-
0.5102900928,-2.4454221794\H,0,4.9065284057,-2.0383382861,-1.875283686
\H,0,4.7687033731,-0.6982859678,2.2086594312\H,0,2.9144891399,0.840354
1906,1.6517791665\H,0,6.0995177013,-2.7136100147,1.5860279343\H,0,6.15
71244604,-3.2664680746,-0.100393472\H,0,7.0761760013,-1.8445536783,0.3
995019059\C,0,-5.553931879,-5.1681517963,-1.2054880539\F,0,-5.58901234

21,-5.544357468,-2.4974013582\F,0,-6.7134472215,-4.5480908486,-0.91647
9194\F,0,-5.4894541198,-6.2859675139,-0.4549159843\\Version=ES64L-G16R
evA.03\State=2-A\HF=-2022.654569\S2=0.761898\S2-1=0.\S2A=0.750127\RMSD
=1.833e-09\Dipole=2.3467984,-1.33462,1.5821673\Quadrupole=10.7896187,-
14.1501914,3.3605727,-22.2250729,-3.7259557,7.4172032\PG=C01 [X(C29H24
F3N1O2S1)]\\@

1q·+

1\1\GINC-B2156\SP\UB3LYP\6-311+G(d,p)\C29H27N1O3S1(1+,2)\ROOT\14-Jan-2
022\0\\#p scrf=(iefpcm,smd,solvent=acetonitrile) b3lyp/6-311+G(d,p)\\i
nt1\\1,2\C,0,-4.850826,-0.070291,0.408228\C,0,-5.533099,-1.090711,-0.2
9545\C,0,-4.799818,-2.158536,-0.860325\C,0,-3.424653,-2.18729,-0.72671
\C,0,-2.717207,-1.171705,-0.032997\C,0,-3.48134,-0.119298,0.542032\C,0
,-1.260729,-1.195348,0.077855\C,0,-0.599691,-0.515592,1.216499\C,0,-0.
480155,-1.863465,-0.829667\C,0,-1.051135,-0.750924,2.528019\C,0,-0.369
302,-0.235094,3.622599\C,0,0.762454,0.565007,3.421651\C,0,1.212454,0.8
48263,2.136207\C,0,0.541528,0.320477,1.020167\N,0,0.924761,0.629745,-0
.31121\C,0,0.033664,1.518136,-1.137728\S,0,2.640939,0.693339,-0.760222
\C,0,0.148767,2.980387,-0.767969\C,0,1.014039,3.827746,-1.478104\C,0,1
.102826,5.179357,-1.148522\C,0,0.336773,5.6977,-0.102414\C,0,-0.525582
,4.860777,0.612906\C,0,-0.615651,3.510628,0.282639\O,0,2.596529,0.9637
83,-2.196329\O,0,3.352443,1.577686,0.16176\C,0,3.197611,-0.97953,-0.49
9683\C,0,3.125579,-1.886797,-1.562849\C,0,3.614663,-3.177052,-1.379125
\C,0,4.182504,-3.574099,-0.157542\C,0,4.249528,-2.636675,0.884719\C,0,
3.770133,-1.339274,0.723786\C,0,4.74659,-4.961875,0.015459\H,0,-5.4331
81,0.744981,0.823998\H,0,-5.304152,-2.959874,-1.38669\H,0,-2.883031,-3
.032315,-1.13854\H,0,-2.975342,0.676675,1.077563\H,0,0.58652,-1.970511
,-0.687022\H,0,-0.907313,-2.337424,-1.707604\H,0,-1.917988,-1.38596,2.
680125\H,0,-0.712615,-0.455368,4.628589\H,0,1.280365,0.997276,4.272657
\H,0,2.053186,1.512431,1.983022\H,0,0.299154,1.346588,-2.180113\H,0,-0
.9825,1.152146,-0.978925\H,0,1.614488,3.42498,-2.288154\H,0,1.769625,5
.827596,-1.709182\H,0,0.408595,6.750416,0.155181\H,0,-1.129775,5.26359
5,1.420729\H,0,-1.295678,2.866695,0.836191\H,0,2.718546,-1.575017,-2.5
19052\H,0,3.571007,-3.883826,-2.203437\H,0,4.698307,-2.92188,1.832288\
H,0,3.859014,-0.613313,1.52383\H,0,4.218341,-5.691532,-0.605821\H,0,5.
803483,-4.986578,-0.280101\H,0,4.692337,-5.291306,1.057461\O,0,-6.8583
58,-0.954067,-0.367857\C,0,-7.647127,-1.927194,-1.069316\H,0,-8.675332
,-1.577737,-0.986084\H,0,-7.354946,-1.975164,-2.123308\H,0,-7.551162,-
2.912798,-0.602369\\Version=ES64L-G16RevA.03\State=2-A\HF=-1800.085154
7\S2=0.764151\S2-1=0.\S2A=0.750085\RMSD=2.833e-09\Dipole=-5.341184,-4.
7301094,0.4357614\Quadrupole=29.1018704,-6.7088234,-22.393047,3.105429
9,12.6234194,12.052934\PG=C01 [X(C29H27N1O3S1)]\\@

TS1-1q

1\1\GINC-B2173\SP\UB3LYP\6-311+G(d,p)\C29H27N1O3S1(1+,2)\ROOT\15-Jan-2

022\0\\#p scrf=(iefpcm,smd,solvent=acetonitrile) geom=check b3lyp/6-31
1+G(d,p)\\Tilte Card Required\\1,2\C,0,-4.2986197223,-3.4851355527,0.5
534749496\C,0,-4.3026474941,-4.4148358232,-0.5115221262\C,0,-3.1758079
235,-4.50911179,-1.3545586461\C,0,-2.0820166196,-3.6936416089,-1.12760
80444\C,0,-2.053570325,-2.7483721983,-0.0669558855\C,0,-3.2045743274,-
2.6792722439,0.7712778483\C,0,-0.8997860926,-1.9178032639,0.142231492\
C,0,-0.6801091966,-1.0681116526,1.3193117768\C,0,0.0366259925,-1.59947
95902,-0.8715937734\C,0,-0.9280482405,-1.3769613746,2.6624808299\C,0,-
0.7217930541,-0.4089386545,3.6455275394\C,0,-0.3008836305,0.875391579,
3.2932966666\C,0,-0.0594480633,1.2119802137,1.9558871363\C,0,-0.218451
8813,0.2301335932,0.9813090399\N,0,-0.0204292924,0.4350783391,-0.44759
05249\C,0,-1.2261798743,0.991631478,-1.2040902298\S,0,1.5314145009,1.2
693512969,-0.9687812766\C,0,-1.7134292752,2.3551259172,-0.7830791691\C
,0,-1.2395544865,3.5096909652,-1.4230124374\C,0,-1.7227948799,4.765408
8483,-1.0588748582\C,0,-2.6870640465,4.8814787737,-0.055267633\C,0,-3.
1733122957,3.7370232907,0.5799341505\C,0,-2.6901240829,2.4805998727,0.
215559478\O,0,1.4100680307,1.2542852076,-2.4246683228\O,0,1.6258129386
,2.5099029117,-0.2049865427\C,0,2.8457586458,0.1797419783,-0.466677541
9\C,0,3.4175691586,-0.6692148943,-1.4221015148\C,0,4.5010768647,-1.460
1523385,-1.0514285252\C,0,5.0274372561,-1.4122034552,0.2494472227\C,0,
4.4367903477,-0.541662438,1.1797667331\C,0,3.3566243917,0.2641331365,0
.8335536402\C,0,6.2255526304,-2.2451228216,0.6277430727\H,0,-5.1826883
423,-3.4180325178,1.1787497614\H,0,-3.1502635726,-5.2232073959,-2.1688
614358\H,0,-1.2092478448,-3.8092717493,-1.7631137616\H,0,-3.2384447911
,-1.9531545583,1.575650729\H,0,1.0928296789,-1.5877496677,-0.631027966
2\H,0,-0.1949194393,-1.7576199569,-1.9228708389\H,0,-1.2504445701,-2.3
768536328,2.9349610483\H,0,-0.8922259195,-0.6566182776,4.6887471961\H,
0,-0.1629865625,1.63020422,4.0614066796\H,0,0.2503519524,2.2128354684,
1.6839280902\H,0,-0.9368536747,0.9760074192,-2.2555173163\H,0,-2.00231
53781,0.2372592672,-1.0463983163\H,0,-0.4966223155,3.4247976386,-2.210
9573557\H,0,-1.3513595248,5.6522085777,-1.5636431967\H,0,-3.0661921491
,5.86036645,0.2237204906\H,0,-3.9350907437,3.8216223573,1.3495626918\H
,0,-3.0855472504,1.5907038941,0.6996528391\H,0,3.0332558891,-0.6883035
921,-2.4364007402\H,0,4.9554555435,-2.115706304,-1.7891120142\H,0,4.83
90043128,-0.4831041021,2.1873157806\H,0,2.9306983872,0.9556474031,1.55
12424596\H,0,6.204399676,-2.5224424455,1.6859857045\H,0,6.284515935,-3
.1600670284,0.0309113716\H,0,7.1523955998,-1.6824360786,0.4564017154\O
,0,-5.4137962739,-5.1514616374,-0.6329043012\C,0,-5.5177477838,-6.1152
24873,-1.6881083455\H,0,-6.5027249192,-6.5661227047,-1.5721360016\H,0,
-5.4461691718,-5.6306066567,-2.6677753142\H,0,-4.7452369008,-6.8855796
983,-1.5904849679\\Version=ES64L-G16RevA.03\State=2-A\HF=-1800.0715125
\S2=0.760044\S2-1=0.\S2A=0.750084\RMSD=5.051e-09\Dipole=-0.043281,-3.9
030606,0.4313444\Quadrupole=12.1701859,-0.1244937,-12.0456922,4.148047
6,6.9333098,18.2818632\PG=C01 [X(C29H27N1O3S1)]\\@

11. X-ray Data

Single crystals suitable for XRD were obtained by vapor diffusion experiment:

Compounds **2k** was separately dissolved in 0.5 mL of dichloromethane and 10.0 mL of n-pentane in three glass vials, which were then placed in sealed glass container. Crystals were obtained in about 4-7 days with the evaporation of the solvent.

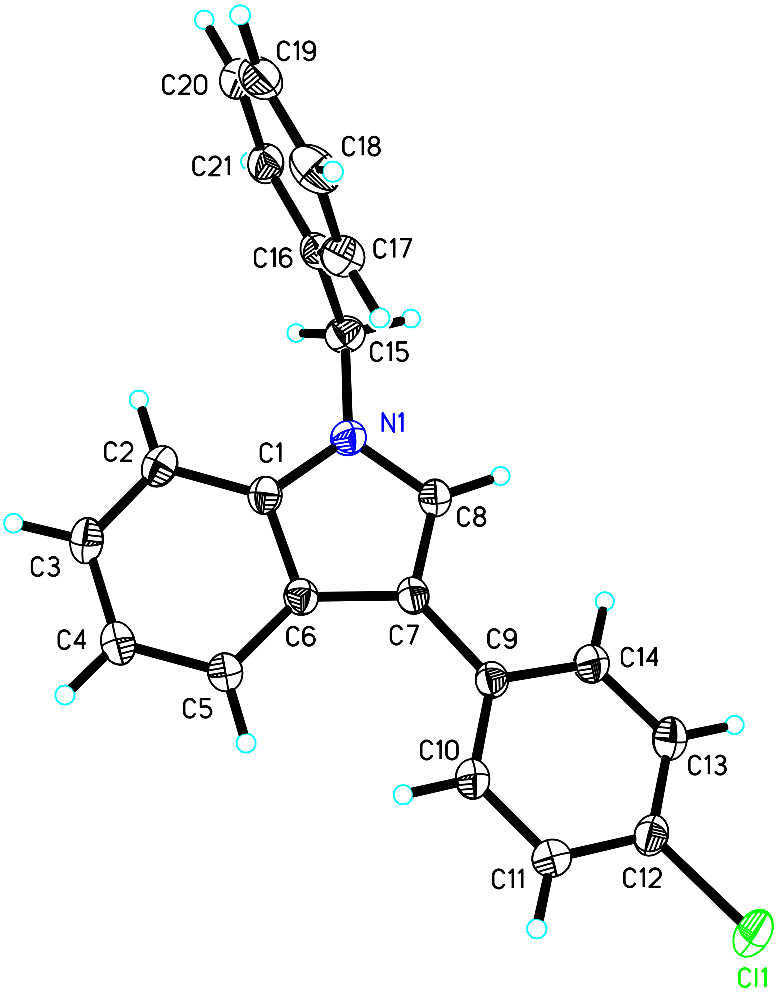

The crystal data of **2k** have been deposited in CCDC with number 2108706. Empirical Formula: $C_{21}H_{16}ClN$; Formula Weight: 317.80; Crystal Color, Habit: colorless; Crystal Dimensions: 0.20 x 0.160 x 0.120 mm^3; Crystal System: Monoclinic; Lattice Parameters: a = 10.7317(3)Å, b = 5.5110(2)Å, c = 27.4878(9)Å, α = 90°, β = 99.3610(10)°, γ = 90°, V = 1604.05(9)$Å^3$; Space group: P 21/n; Z = 4; D_{calc} = 1.316 g/cm^3; F_{000} = 664; Final R indices [I>2sigma(I)] R1 = 0.0630, wR2 = 0.1706; ; the thermal ellipsoids are set at a 30% probability level.

12. References

1) Chatupheeraphat, A.; Rueping, M. & Magre, M. *Org. Lett.* **2019**, *21*, 9153-9157.

2) Li, Q.; Yu, L.; Wei, Y. & Shi, M. *J. Org. Chem.* **2019**, *84*, 9282-9296.

3) Fang, B.; Hou, J.; Tian, J.; Yu, W. & Chang, J. *Org. Biomol. Chem.* **2020**, *18*, 3312-3323.

4) Zhang, Z.; Kvasovs, N.; Dubrovina, A. & Gevorgyan, V. *Angew. Chem. Int. Ed.* **2022**, *61*, e202110924.

5) Ortgies, S. & Breder, A. *Org. Lett.* **2015**, *17*, 2748-2751.

6) Trân, K.; Hauwe, R. V. D.; Sainsily, X.; Couvineau, P.; Côté, J.; Simard, L.; Echevarria, M.; Murza, A.; Serre, A.; Théroux, L.; Saibi, S.; Haroune, L.; Longpré, J. -M.; Lesur, O.; Auger-Messier, M.; Spino, C.; Bouvier, M.; Sarret, P.; Ballet, S. & Marsault, É. *J. Med.Chem.* **2021**, *64*, 5345-5364.

7) Sun, W.; Chen, C.; Qi, Y.; Zhao, J.; Bao, Y. & Zhu, B. *Org. Biomol. Chem.* **2019**, *17*, 8358-8363.

8) Hill, J.; Hettikankanamalage, A. A. & Crich, D. *J. Am. Chem. Soc.* **2020**, *142*, 14820-14825.

9) Jang, Y. H. & Youn, S. W. *Org. Lett.* **2014**, *16*, 3720-3723.

10) Liu, J.; Wei, Y. & Shi, M. *Angew. Chem. Int. Ed.* **2021**, *60*, 12053-12059.

11) Guo, Z. -Q.; Xu, H.; Wang, X.; Wang, Z. -Y.; Ma, B. & Dai, H. -X. *Chem. Commun.* **2021**, *57*, 9716-9719.

12) Ghorai, J.; Reddy, A. C. S. & Anbarasan, P. *Chem. Asian J.* **2018**, *13*, 2499-2504.

13) Barluenga, J.; Fernández, M. A.; Aznar, F.; Valdés, C. *Chem. Eur. J.* **2005**, *11*, 2276-2283.

14) Bering, L.; Paulussen, F. M. & Antonchick, A. P. *Org. Lett.* **2018**, *20*, 1978-1981.

15) Ghorai, J.; Reddy, A. C. S. & Anbarasan, P. *Chem. Eur. J.* **2016**, *22*, 16042-16046.

16) Dou, X.; Yao, W.; Wen S. & Lu, Y. *Chem. Commun.* **2014**, *50*, 9469-9472.

17) Grimme, xtb 6.0; Mulliken Center for Theoretical Chemistry, University of Bonn, **2019**; available upon request via e-mail to xtb@thch.uni-bonn.de.

18) Marcus, R. A. *J. Chem. Phys.* **1957**, *26*, 872-876.
